# Generalization of navigation memory in honeybees

**DOI:** 10.3389/fnbeh.2023.1070957

**Published:** 2023-03-06

**Authors:** Eric Bullinger, Uwe Greggers, Randolf Menzel

**Affiliations:** ^1^Institut für Automatisierungstechnik, Otto-von-Guericke-Universität Magdeburg, Magdeburg, Germany; ^2^Neurobiologie, Freie Universität Berlin, Berlin, Germany

**Keywords:** landmark learning, orientation flight, observational learning, matching landmarks, random search, elongated ground structures

## Abstract

Flying insects like the honeybee learn multiple features of the environment for efficient navigation. Here we introduce a novel paradigm in the natural habitat, and ask whether the memory of such features is generalized to novel test conditions. Foraging bees from colonies located in 5 different home areas were tested in a common area for their search flights. The home areas differed in the arrangements of rising natural objects or their lack, and in the existence or lack of elongated ground structures. The test area resembled partly or not at all the layout of landmarks in the respective home areas. In particular, the test area lacked rising objects. The search flights were tracked with harmonic radar and quantified by multiples procedures, extracting their differences on an individual basis. Random search as the only guide for searching was excluded by two model calculations. The frequencies of directions of flight sectors differed from both model calculations and between the home areas in a graded fashion. Densities of search flight fixes were used to create heat maps and classified by a partial least squares regression analysis. Classification was performed with a support vector machine in order to account for optimal hyperplanes. A rank order of well separated clusters was found that partly resemble the graded differences between the ground structures of the home areas and the test area. The guiding effect of elongated ground structures was quantified with respect to the sequence, angle and distance from these ground structures. We conclude that foragers generalize their specific landscape memory in a graded way to the landscape features in the test area, and argue that both the existence and absences of landmarks are taken into account. The conclusion is discussed in the context of the learning and generalization process in an insect, the honeybee, with an emphasis on exploratory learning in the context of navigation.

## 1. Introduction

Successful navigation requires forming a lasting memory of the locations and identities of significant objects in the environment in relation to each other and a compass. Multiple perceptual systems are involved in probing the world during navigation, and vision is usually the most important sense in further reaching navigation. Recognizing and storing the spatial relations of objects requires reference systems of two kinds, egocentric and environmental (or allocentric). Egocentric references include view point memories, path integration (or dead-reckoning) and body relations to a geocentric reference like the sun compass (Wehner et al., 1996; Collett and Rees, 1997; Collett et al., 2003). Environmental (allocentric) references structure memory such that the spatial relations between egocentric and geocentric references as well as the spatial relations between identified objects are stored. The level of integration between egocentric and allocentric references in insects, and particularly in honeybees, is under debate (Collett and Graham, 2004). The underlying neural processes may be conceptualized as the activation of multiple isolated *ad-hoc* procedures or as the retrieval of a concise navigation memory. In the latter case, generalization tests may provide us with hints about the level of integration across the multiple neural processes involved. Support for this view comes from multiple observations in test conditions in which close visual cues at a feeding site were systematically changed both during training and testing in order to uncover higher order memory processing (Giurfa, 2003, 2015). In these experiments, bees were asked whether they generalize across learned cues that can be discriminated but contain hidden parameters that binds them to categories, i.e., learning of bilateral symmetry (Giurfa et al., 1995), matching-to-sample (Giurfa et al., 2001).

**Figure 1:**
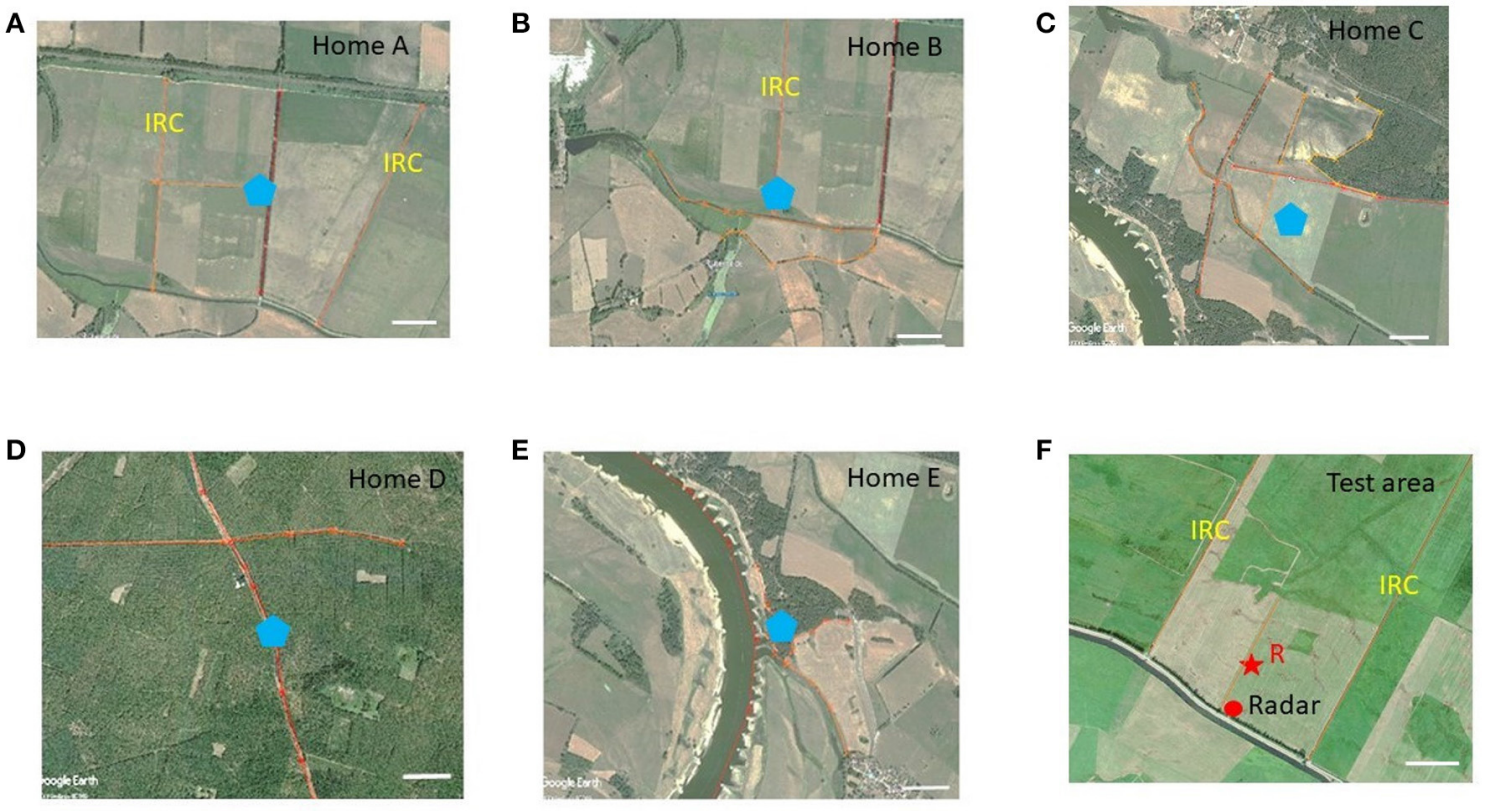
Areal views of the test area **(F)** and the 5 home areas **(A–E)**. The release site R in the test area is marked with a red star, and the location of the radar with a red dot. The locations of the hives in the five home areas are marked with blue pentagons. The elongated ground structures in the test and home areas are highlighted with orange lines. The scale in each subfigure corresponds to 200 m. IRC, irrigation channels. Notice that the ground structures in the test area are more similar to home areas **(A, B)** than to **(C–E)**, and that home areas **(D, E)** are highly different from the test area and from each other. Most importantly, the home areas differ from each other and the test area not only in the ground structures, but also in the panorama and the distribution of rising objects.

Landscape memory acquired during exploratory orientation flights and foraging flights in honeybees has been characterized so far mostly by isolating perceptual and procedures components (e.g., path integration, matching of visual images), and were mostly tested separately often even not over dimensions of natural navigation. Here we apply a different approach by characterizing the partial use of the acquired landscape memory in the home area in a novel test area that differs in its landscape structure more or less, a procedure called generalization in learning theory (Blough, 1975; Kehoe, 2008). Animals from a colony located at a different site (its home area) are transported into a test area that partially resembles landscape features on the ground but differs drastically with respect to rising objects both close by and at the horizon (Figure 1, see also Section 4). Five different home areas were chosen, and all animals were released at the same place in the test area. Four home areas were so far away from the test area that no test bees ever came close to the test area. One home area was located 1.6 km away from the test area, and indeed a few of these animals managed to fly back to their home area. These few animals were not included in our analyzes.

All animals were experienced foragers having calibrated their sky compass and visual odometer, and learned the multiple landscape features for successful homing. The learning flights of the test animals were not recorded due to technical limitations, but we are safe to assume that their intensive exploration and foraging activity prior to testing established a strong navigation memory (Capaldi et al., 2000; Menzel et al., 2005; Degen et al., 2015, 2018). It is known that foragers transported into an unknown area return multiple times to the release site (Dyer, 1996; Menzel et al., 1998), possibly applying search routines that include random components and possibly also innate guidance components with respect to compass cues and landscape features. Random search patterns have been well analyzed in the desert ant *Cataglyphis* (Wehner and Srinivasan, 1981), but were not addressed yet for the honeybee in the dimension of natural navigation. Thus, we expect that animals from different home areas will perform search flights with multiple returns to the release site with random components directions and distances explored but possibly also some structured flights related to landscape features. Most importantly, the search flight patterns of animals from different homes areas would not differ if only these processes guide them. However, if the navigation memory acquired in their home area can at least be partially generalized to the features experienced in the test area, their behavior will differ from each other. We argue that the generalization process may motivate them to explore some landscape features in the test area more intensively. The local density of exploration may thus reflect a generalization effect that may support the notion of a concise navigation memory.

The navigation memory established in the 5 home areas will differ due to the layout of the respective landmarks, potentially resulting in different search patterns in the same test area (Figure 1). Thus, a similarity measure based on the search patterns may reflect components of the navigation memory. We hypothesize that generalization indicates a form of navigation memory based predominantly on salient elongated landmarks. Matching of stored views of the panorama with views in the test area should play no or little role in our experiments because of the drastic difference of panorama between the home areas and the test area for four of the five home areas. The same will apply to localized rising landmarks because there were none such landmarks in the test area. Ground structures, however, may influence their search flights. In the test area, the animals may thus identify features preferentially on the ground that partially resemble features they had learned in their home area, and thus they may generalize between such features. Reduced generalization may also depend on the lack of landscape features the animals has learned in the home area. These hidden effects of generalization need to be kept in mind when trying to relate physical characters of the home area with those of the test area.

First, we shall examine whether the search flights follow a random search strategy running two mathematical models. After showing that random flight alone cannot explain the search behavior, we find that the search flights differ between animals from the different home areas. We next asked about the impact of the elongated ground structures in the test area. Finally, we quantify the differences in search strategy of animals from the different home areas and compare the effects by analyzing the differences between the elongated ground structures of the home areas with that of the test area.

## 2. Results

### 2.1. Analytical procedure

**Figure 2:**
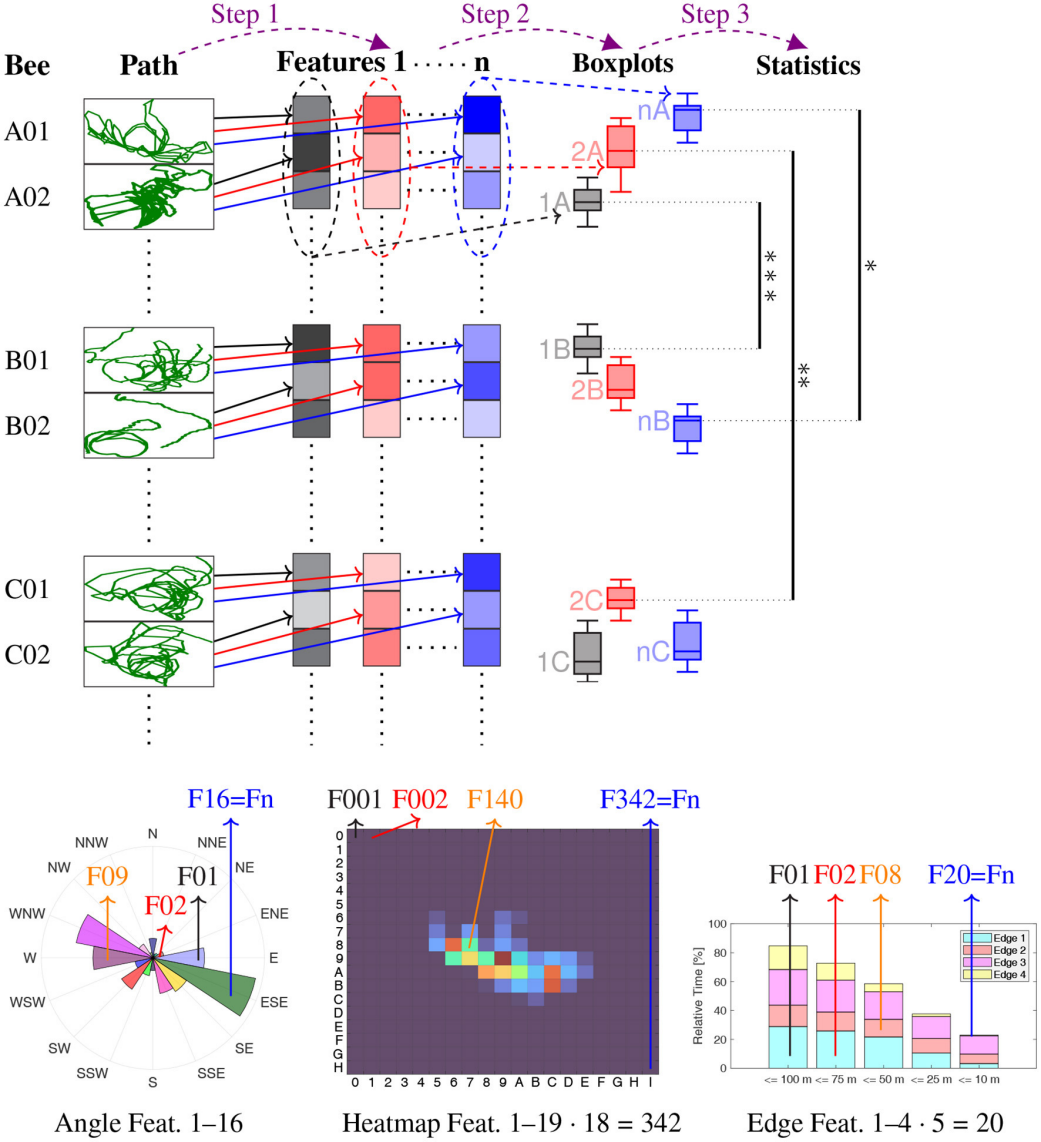
Illustration of the analysis procedure. Step 1: The flight path of each bee is used to calculate a number of features (the black, red and blue boxes), compressed in vertical direction for illustration purposes. These features are Directional analysis Relative time spent in each of 16 cardinal directions, relative to the release site → *n* = 16 features, Heatmap analysis Relative time spent in 19·18 = 342 squares of 100 m times 100 m → *n* = 342 features, Heatmap analysis with PLS Partial least square projections of the 342 heatmap squares → *n* = 3 features, Analysis of edges Relative time spent near each edge, in five distance ranges (4·5 = 20 features), for each of these also the relative time spent in one of six angle ranges (additionally 4·5·6 = 120 features), thus in total → *n* = 140 features. Step 2: The distribution of each feature within the bees of each of the seven groups (A–E, R, S) is visualized by boxplots (same color as feature, e.g., 1A stands for Feature 1 of all bees in Group A). Step 3: The feature distributions are statistically compared using measure of effect analyzes. Bottom row Some of the features of Step 1, at the example of Bee A01: angular histogram of the directional analysis **(left)**, heatmap **(middle)**, and time spent near edges **(right)**. Significance levels: ^***^ Δmes ≥ 0.4, ^**^ Δmes ≥ 0.3, ^*^ Δmes ≥ 0.2.

**Figure 3:**
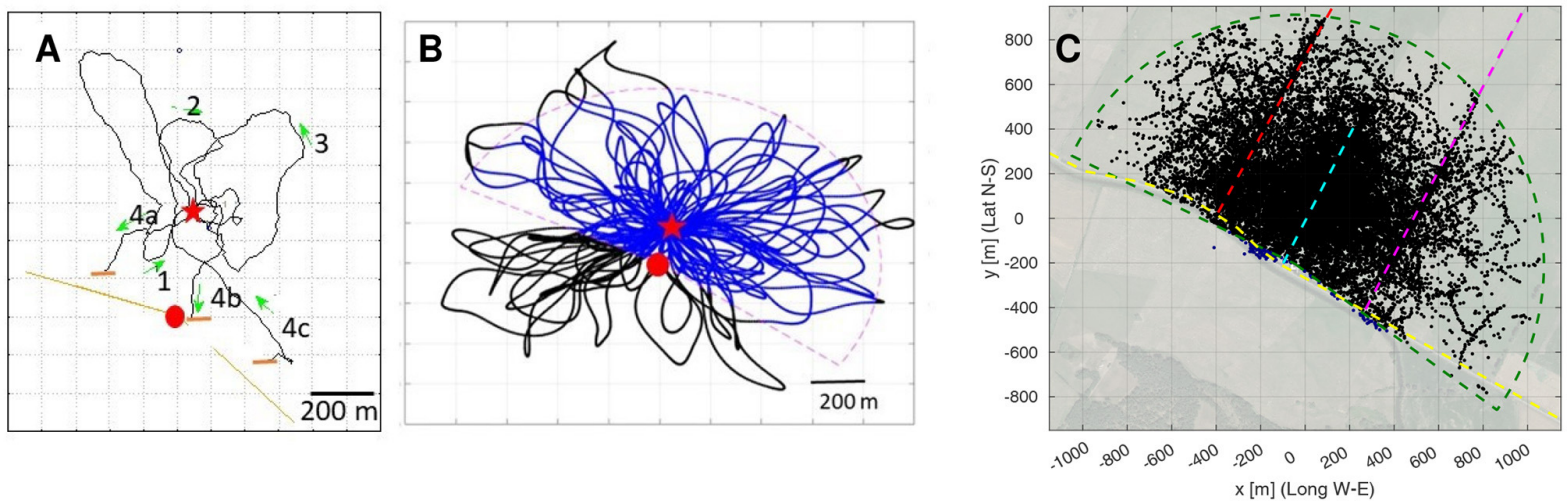
The structure of search flights. **(A)** Example of a bee's search flight (Bee 05 from Home Area E). **(B)** Example of a simulated bee's flight. Two models were run, Model S with search loops in all directions (black and blue trajectories) and Model R in which fixes outside the radar range (dashed line) were excluded (only blue parts of the trajectories). **(C)** All fixes of all search flights plotted together with the radar range(green dashed line) with the release site at the origin. Fixes in the range in black, in blue the approx. 0.45 % outside that range (in proximity of the radar, south of the radar range). The dashed line (red, cyan, magenta and yellow) highlight the edges (elongated ground structures) of the test site. Red dot: radar site, Red star: release site, Green arrows: direction of flight.

The statistical analysis proceeds as follows (see Figure 2 for an illustration). First, specific features are calculated individually for each bee. Examples are the relative time spent in one of 16 cardinal directions (Section 2.2), heat map analysis (Section 2.3), or time spent near a ground structure (Section 2.4). The distribution of a specific feature among all bees of a group, visualized as boxplot, is compared between any two groups to identify statistically distinctive properties of the flight paths. A mathematical model generating random flight paths (see Section 2.2) was developed and utilized to study the randomness of the observed bees (Figure 3B).

### 2.2. Directional analysis of model-based vs. real search patterns

The basic search strategy of all animals released at the unexpected and unknown site in the test area consisted of multiple returns to the release site via multiple loops ranging over different distances and in different directions (Figure 3A). No systematic sequences of growing distances and changing directions were apparent. One may assume, therefore, that the bees just performed random search flights. We tested this question by running two models of random search. Our model calculations assumed multiple returns to the release site with randomly directed loops of increasing size (Figure 3B). These search paths were generated based on a modified version of the ant model by Wehner and Srinivasan (1981) (see Section Methods for the details). The S model bees include the sector not covered by the radar (radar blanking), while the R bees paths are identical to the S bees, but excluding the fixes in the radar blanking (Figure 3B). Figure 3C shows all fixes of all real bees together with the assumed sector covered by the radar that captures over 99.5% of the fixes.

**Figure 4:**
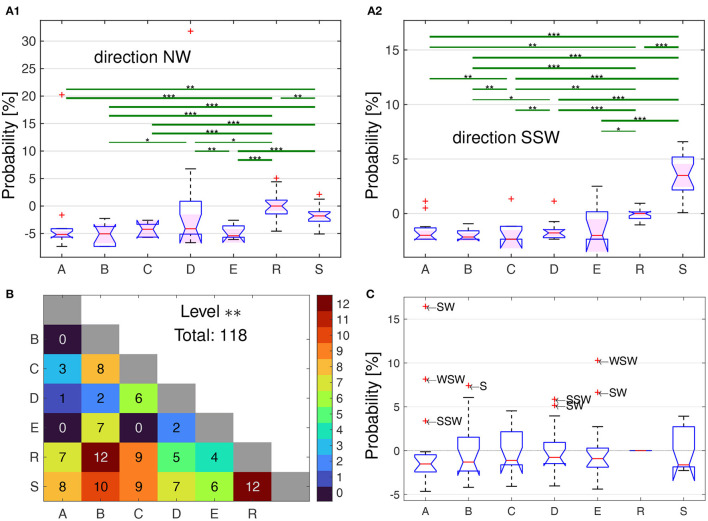
Relative proportions of flight directions for the bees from the five home areas (A–E) and the simulated bees in the models R and S. **(A1**, **A2)** Statistical analyzes of the directional frequencies, at the example of directions NW **(A1)** and SSW **(A2)**. The boxplots show the distribution of the relative time each bee within its group spent in the sector NW or SSW, respectively, additively normalized by the median of the R bees, i.e., 0 % means the same probability as the median of the R bees, 5 % means 5 % more time in that sector. The ordinates are scaled differently due to two extreme outliers in the NW-direction. Significant differences between groups of bees are shown by the green lines, with * corresponding the a difference of Measures of Effect Size based on Cohen's U3 test for two samples (Δmes) of at least 0.2, while ** for Δmes≥0.3, and *** for Δmes≥0.4 up to the maximal difference of 0.5. Two examples are shown here, with the full set of results in Supplementary Data Sheet S2. **(B)** Total number of significant differences among the 16 directions for each pair of groups, with Δmes≥0.3 (which corresponds to **), the number is also color coded according to the colorbar on the right. In total, there are 118 cases where a direction can be used to used to discriminate two groups with Δmes≥0.3, i.e., significance level **. **(C)** Boxplots of the distribution of the median relative time the bees of each group spent in each of the 16-wind compass directions [see **(A1)** and **(A2)** for two examples], additively normalized by the corresponding value of the R bees. Thus, all probabilities are zero for the R bees, while e.g., a positive value of 5 % correspond to directions in which at least half of the bees of a group spent 5 % more time than half of the R bees in that direction. All outliers (+) are labeled with the abbreviated direction they represent.

If bees from the 5 different home areas (see Figure 1 for the layout of the landmarks) would apply a random search strategy only, they should explore the area around the release site about equally frequently and no differences would be expected from the model R or S simulated bees. In a first step we compared the relative number of fixes in 16 angular sectors around the release site by normalizing it in each sector to that of the simulated bees of model R on the level of the individual bees from the 5 home areas and those of the modeled “bees” in model S. We chose the results of model R because it takes into account the bias of no fixes in the area not scanned by the radar, the radar blanking sector. Figure 4 shows the relative proportions of flight directions for the bees from the five home areas (A–E) and the simulated bees in the models R and S. These relative distributions taken together for all distances were statistically analyzed using the Measure of Effect Size (mes) based on Cohen's U3 test for two samples (Cohen, 1988). Figure 4, Supplementary Data Sheet S2 contains all directions as well as statistical analyzes with the Kruskal-Wallis test.

Significant differences according to the measures of effect size as expressed by Δmes were specified for the three threshold values ≥0.1, 0.2, and 0.3, and these values are given in Figures 4A1, A2 for two examples (NW and SSW) of the 16 directional sectors together with the relative frequencies per bee of the directions for the 5 groups of test bees and the “bees” of the two models, R and S. The full set of the results of all 16 directional sectors are given in Supplementary Data Sheet S2. Although the radar blanking implies a dominance of flights into the N–E sector, several bee groups spent more time in south-western direction as compared to the R bees (Figure 4C). The bees from the different home areas differed in their relative directional distributions both in comparison with that of the models and between the 5 test groups as indicated by the pairwise measure of effect size (Figure 4B). The respective numbers of statistically significant cases for the measures of effect size are much more frequent with a total number of 183 including the two models, and 31 between the 5 groups of test bees only, where significant means *p* ≤ 0.01 or Δmes ≥0.2. The measures of effect size shows significant differences between 18 out of 21 pairs, the exception here are A vs. B, A vs. C, and C vs. E (Figure 4B).

### 2.3. Quantifying the generalization effect on the basis of the spatial distribution of radar fixes

**Figure 5:**
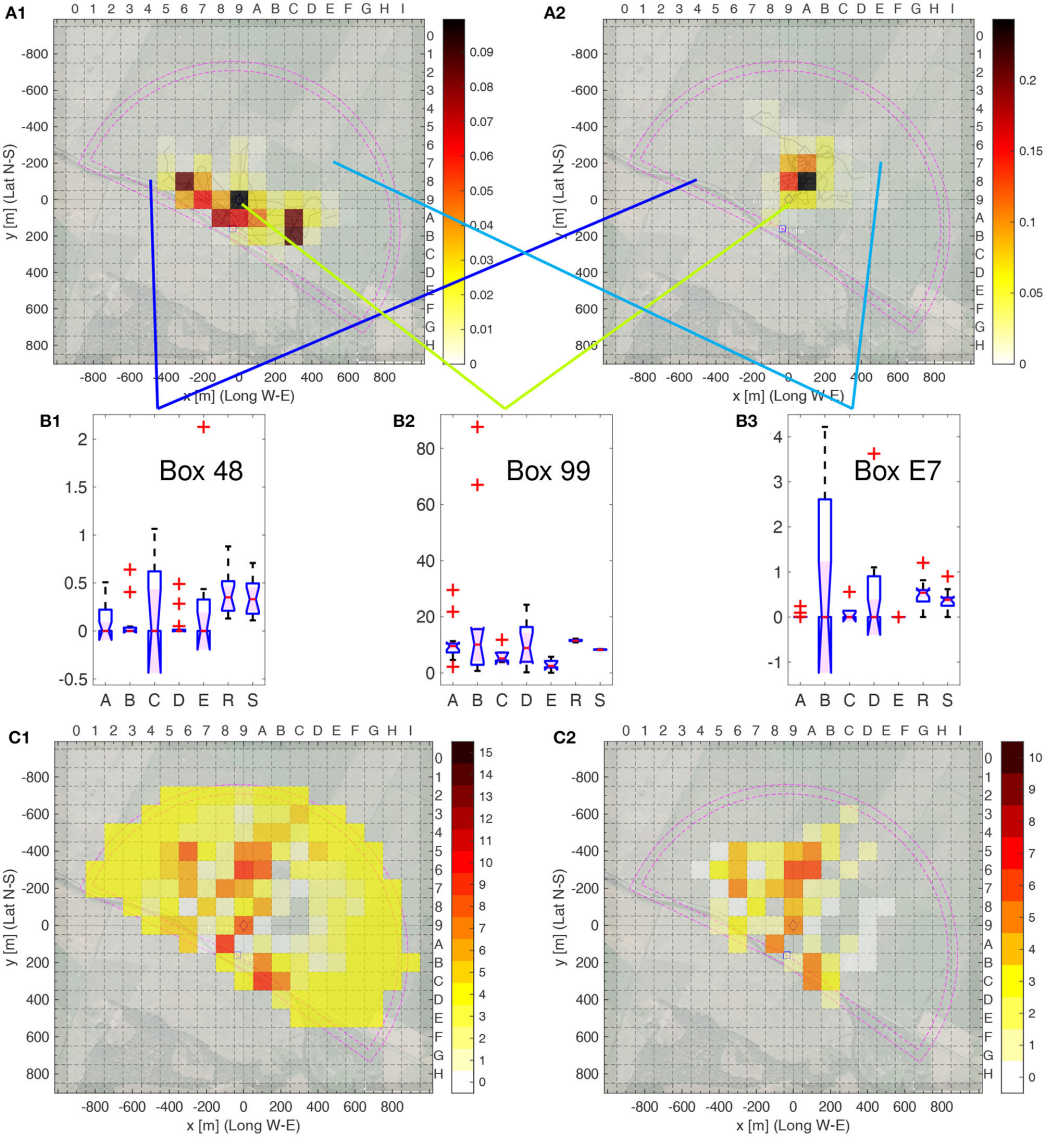
Heatmap analysis of individual bees. **(A1, A2)** Heat map examples. **(A1)** Bee A01, bee number 1 from area A; **(A2)** Bee R01, bee number 1 from model R. The respective flight trajectories are given in pale lines. The relative number per box of fixes of the smoothed trajectories is color coded as indicated in the color bar to the right; the sum over all boxes is one. The inner purple lines indicate the area covered by the radar, the outer ones cover an extra distance of 50 m from the radar, which corresponds to the size of the smoothing filter. **(B1–B3)** Boxplots of the relative frequencies of fixes of each bee being in one of the three selected boxes of the total of 342 boxes as indicated by the blue, green and cyan lines. **(B1)** Box 48, **(B2)** Box 99, and **(B3)** E7. **(C1, C2)** Number of group pairs with Δ_mes_≥0.3, i.e., significance level **. **(C1)** All pairs of bee groups A to E plus R, i.e., 15 in total; **(C1)** all pairs of bee groups A to E only, i.e., 10 in total. The colorbars to the right gives the color code for the number of pairs. Significance levels: ^**^ Δ_mes_≥0.3.

We constructed a 19 × 18 matrix of equally sized tiles (100 × 100 m, numbered 0–I in W–E and 0–H in N–S direction, with Tile 99 centered at the release site). Then, each bee's flight path fixes were interpolated and a smoothing filter applied, as described in Section 4.3.2, resulting in a spatially discretized heat map per bee, see Figures 5A1, A2 for two examples. Further results of this analyzes are given in Supplementary Data Sheet S5 (significance of each one of the 19·18 = 342 tiles, as in Figures 5B1–B3), Supplementary Data Sheet S5 (pairwise comparison), and Supplementary Data Sheet S5 (comparison of one group to all others).

Figures 5B1–B3 show three example of the distribution of the heat map value, were the boxplots show the distribution of the frequency of the filtered fixes in one heat map box, sorted by group, for the others, see Supplementary Data Sheet S4. Several results are obvious. First, the homogeneity varies by group and by box, e.g., compare the very homogeneous Groups B and D in Box 48, Groups R and S in Box 99 as well as Group A and E in Box E7 (small interquartile range, i.e., height of the box), to the heterogeneity of Group C in Box 48 or Group B in Box E7 (large interquartile rage). For each box and each pair of groups, we calculated the Δmes, see Supplementary Data Sheet S5. Figures 5C1, C2 show the number of groups that can be distinguished using a single heat map box, when setting as threshold Δmes≥0.2, i.e., significance level **. In both cases, model group S was ignored as those bees can easily be distinguished as they are the only ones with fixes outside of the radar range. Figure 5C2 only compares the real bees among themselves. Several boxes allow for discriminating many pairs of groups, for example Box 96 and A6, but many other boxes are useful for discrimination of a few pairs (yellow colored boxes). No clear pattern is evident. When including Group R in the comparison, as shown in Figure 5C1, many more boxes are useful for discrimination of the groups, i.e., allowing the discrimination of Group R to the real bees, Groups A–E. Here, boxes further away from the release site, situated in Box 99, are more informative then when comparing only bees in Groups A–E. We conclude that the spatial distribution of fixes differs between the five home areas and cannot be explained by random flights or equal guidance of all test animals by landscape features of the test area.

#### 2.3.1. Partial least squares regression analysis

To quantify the generalization effect, we applied a variant of principle component analysis (PCA), a partial least squares regression (PLS) analysis (see the Method/Computation, Section 4.3.2), to the heat map data with spatial discretization as shown in the previous section, aiming to quantify a similarity-difference gradient of the unknown guidance parameters.

PLS estimates a linear model fitting the predictor X, here the sampled spatial distribution data (342 dimensions) for each of the 66 bees, and to the observation data Y (here the seven-dimensional, binary selector of the home area of each of the 66 bees). In comparison to PCA, PLS's advantages are that it can deal with correlations in the rows of the predictor matrix and that it takes Y directly into account, while PCA only works on X. Similarly to PCA, the PLS's principal components can be used to obtain a reduced order linear model. We then analyzed the three most relevant components for each bee. We performed three different PLS analyzes. The first included all seven groups (five test and both model groups R and S). Its three main components explained 91.4 % of the variance. The second analysis ignored the S group, and there the three main components explained 90.6 % of the variance, while the third analysis, which ignored all test bees, explained 87.8 %.

**Figure 6:**
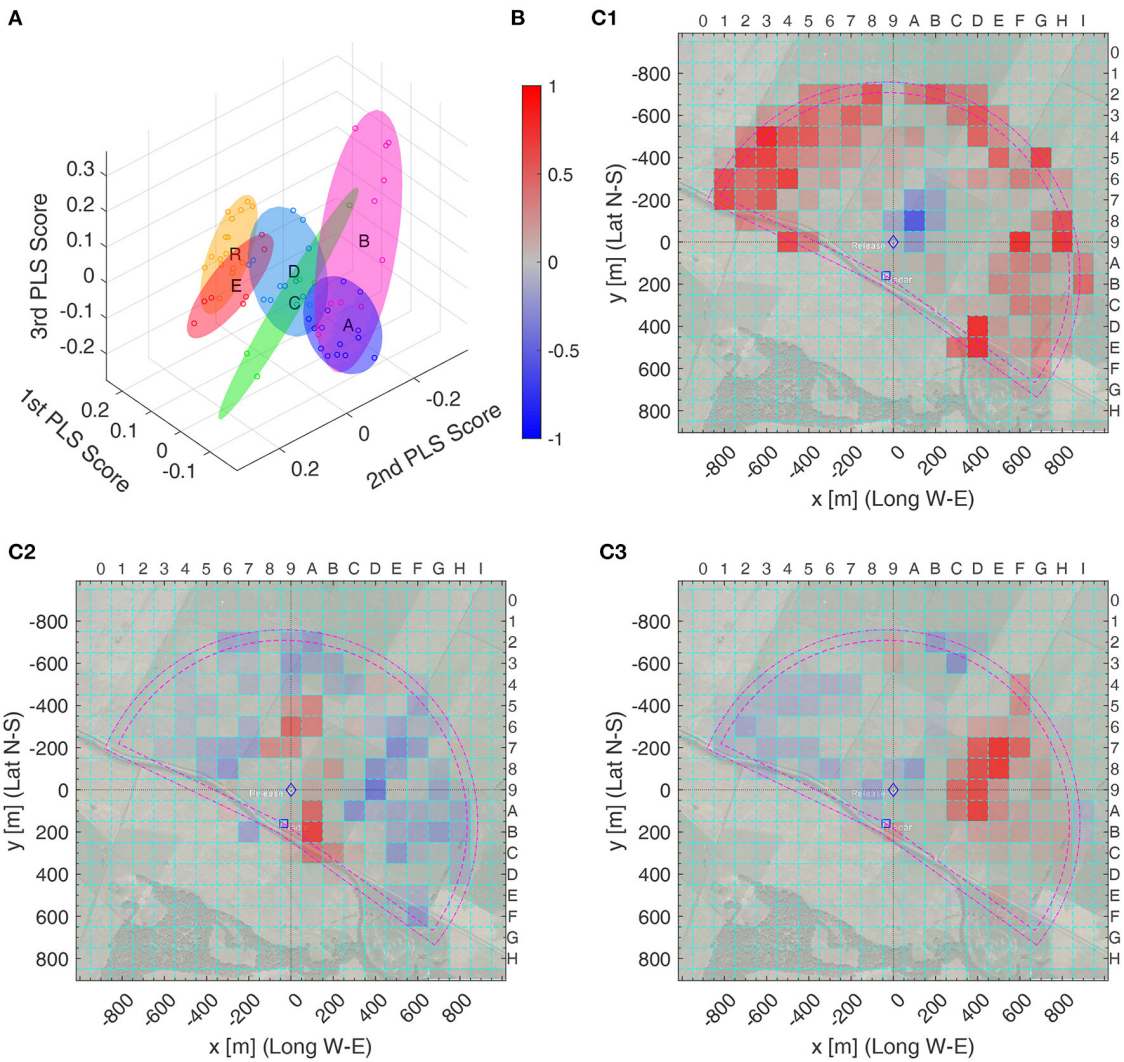
PLS analysis of the heat maps of radar fixes of all test and model groups. **(A)** 3D plot of the top three PLS scores for each bee's heat map (circles), together with minimum volume ellipsoids covering all PLS scores of one group. Color code of the ellipsoids and circles: A, B, C, D, E, and R. **(B)** Colorbar of the PLS support vectors values, normalized to the interval [−1, +1], for **(C1–C3)**. **(C1**–**C3)** Top three PLS support vectors, with color coding as in **(B)**: **(C1)** first, **(C2)** second, and **(C3)** third PLS support vector. Left and bottom label: distance from the release site, ♢, in meter relative to the Release Site; top and right: x and y label of the spatial discretization boxes. For example, PLS Support Vector 1 is mostly positive (red) outside of the radar range (magenta half circles, inner one: true radar range, outer one: increased range due to smoothing filter) and all bees in Group **S** have a higher positive value for the corresponding PLS score than all other bees, see **(A)**, Thus, the bees in Group **S** spent a higher proportion of their time outside of the radar range than the bees A–E and R, as for them this time could not be measured.

The top three PLS scores can be visualized as points in 3D (Figure 6A) and their support vectors as heat maps (Figures 6C1–C3). Figure 6A additionally shows the minimum volume ellipsoids that contain the 3D PLS scores of each bee in a group. Supplementary Data Sheet S8 contains files in the X3D-format, which allows for rotating and zooming the 3D representation. The dominant support vector, Figure 6C1 resembles Figure 5C1, the region away from the release site seems very important. The second PLS vector, Figure 6C1, also looks somehow similar to Figure 6C2 and thus appears to be more important to discriminate among the test groups A–E.

**Figure 7:**
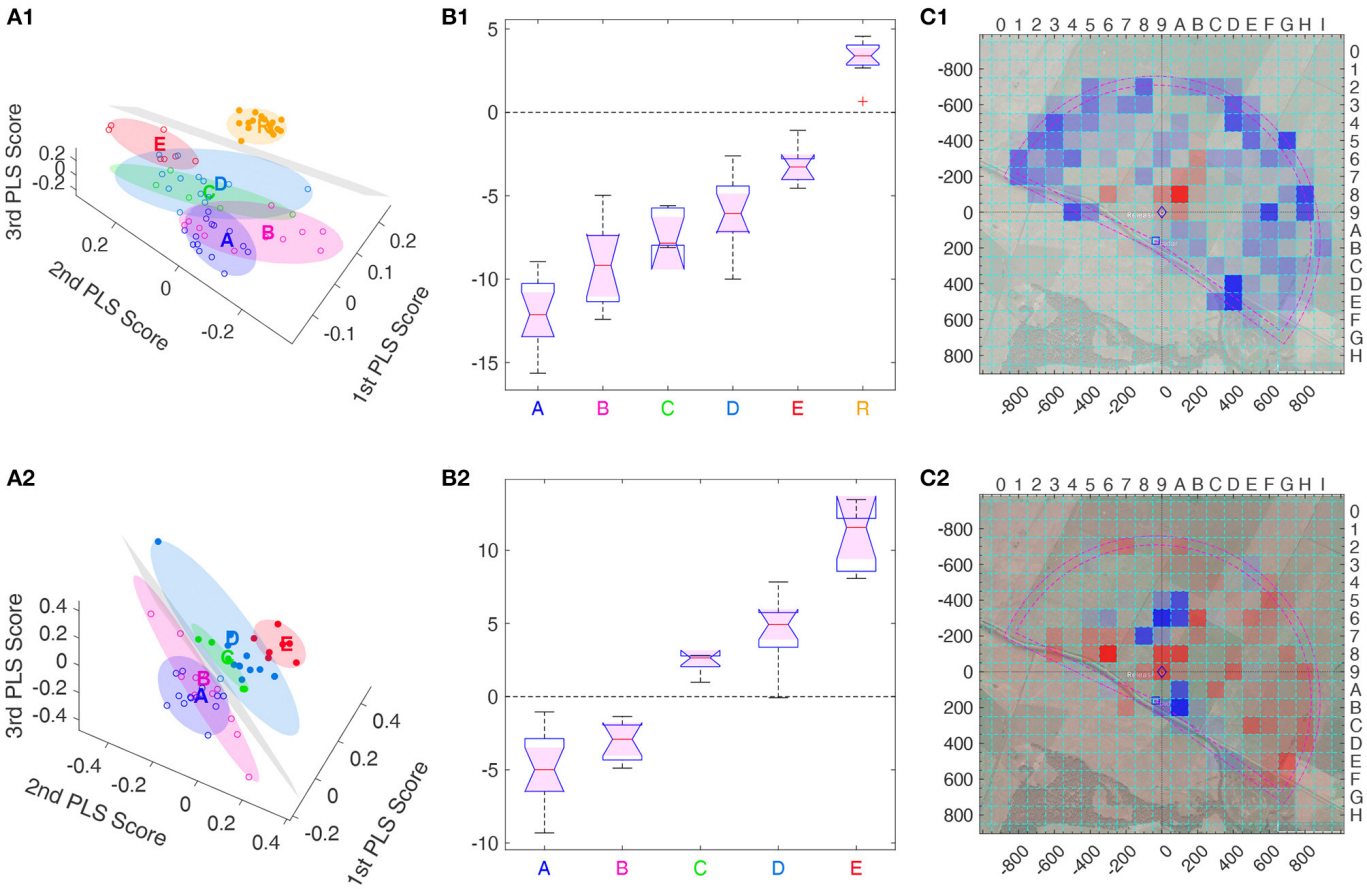
Separatrices, obtained using support vector machines (SVM), of the three main PLS components. **(A1–C1)** (first row) Hyperplane separating R bees from all other groups, with PLS that ignored S group. **(A2–C2)** (second row) Hyperplane separating the A and B group from the C, D, and E groups, in the space of the three dominant PLS support vectors where both R and S group were ignored. **(A1, A2)** (first column) 3D view of each bee's heatmap projected onto the three main PLS support vectors (° or •), together with a minimum volume ellipsoid containing all bees of one group (color code for points and ellipsoids: A, B, C, D, E, and model group R) as well as the separating hyperplane (in gray). All bees on one side (positive side) of the hyperplane are marked •, those on the other side (negative side) by °. **(B1, B2)** (second column) Distribution of the signed distance of each bee to the separating hyperplane within each group, with the separatrix marked by the dashed line at distance zero. Positive distance corresponds to points above the hyperplane and are denoted by • in the 3D views **(A1, A2)**, while points below have a negative distance and are marked by °. In both cases, the distance to the separatrix allows for discriminating each pair of groups with significance level ***, i.e., a Δmes≥0.4, except for the pair C vs. D in **(B1)** with **, due to Δmes = 0.35. **(C1, C2)** (third column) Separating hyperplanes viewed as heat maps, color coded as in Figure 6B. Significance levels: ^***^ Δmes ≥ 0.4, ^**^ Δmes ≥ 0.3.

As could be expected, model S is well separated from all other groups as only its bees fly in the radar blank (see Supplementary Data Sheet S8), but also the R model bees differ from the real bees (Figure 6A). The latter is even better visible when viewing this from a different angle as in Figure 7A1. Here, a hyperplane, shown in gray, was obtained with a support vector machine (SVM) classification, to separate Group R from Groups A–E. In this 3D diagramm, each bee (dot) has a signed distance from the hyperplane, with positive distance on one side (filled dots) and negative distance (empty dots) on the other. Figure 7B1 shows the distribution of these distances among the bees of each group, with zero being the separatrix, the separating hyperplane. Intriguingly, the distance to this separatrix orders the bee groups alphabetically and the corresponding mes statistics show very significant differences between R bees and each other group, but also between any two of the test bee groups: a Δmes≥0.4, i.e., a significance level *** for all pairs, except for the pair C vs. D having only a Δmes = 0.35, thus still a significance level **. The separatrix itself can also be visualized as a heat map, see Figure 7C1. Thus, the separatrix balances boxes away from the release site (in blue) to some boxes closer the release site (in red), and positive distances correlate to blue boxes in that visualization.

The PLS analysis can also be performed only on real bees, see second line Figure 7. Here, we show a hyperplane separating Group A and B from Groups C–E. This analysis allows for separating all bee groups with significance level ***, thus also C from D, even though the separatrix was not chosen to perform this task. The heat map view of the separatrix shows a more intricate pattern, whose structure is not obvious (Figure 7C2). Combined with the distance information, this reveals that E bees searched more to the north and south of the Release, and less in several other spots as e.g., in the south-east, see the blue colored boxes in Figure 7B2.

The previous analyzes completely ignored the landscape of the test area. The next section studies how the bee flight paths correlate to the dominant landscape structures of the test area.

### 2.4. The guiding effect of edges

**Figure 8:**
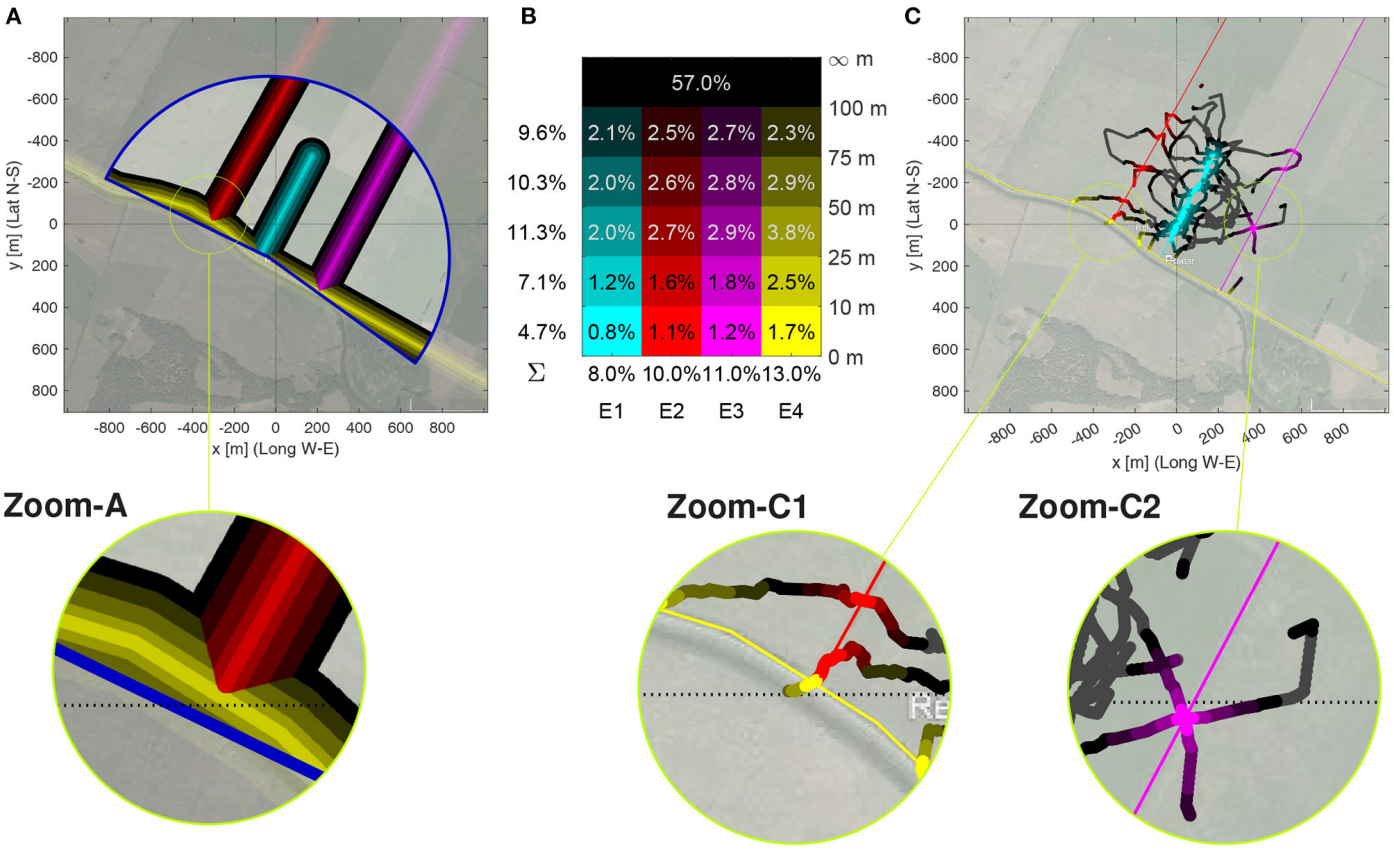
Discrimination analysis based on time and flight angle near edges. **(A)** Pixels within the five distance ranges of 0 to 10 m, 10 to 25, 50 to 75 m, and 75 to 100 m (color intensity coded) to one of the four edges (color coded). Color coding of closest edge and distance to edge as in **(B)**. The radar range is the blue bordered circular sector. Latitudidal and longitudinal coordinates are relative to the Release Site, Relative surface within the radar range covered by the distance ranges (rows) to each edge (columns E1–E4). The sums of the relative surface near each edge is shown below, the sums for each distance range on the left. Further away than 100 m from any edge are 57 % of the pixels. This figure also serves as legend for the color coding of the 4 × 5 distance ranges. **(C)** Flight path of Bee A05 with color-coding as in **(B)** of path segments when closer than 100 m to an edge. The full path is plotted as a black line.

Next, we focused on the relative time each bee spent near one of the four edges, and then on the bees direction of flight relative to the edge direction when close to these edges. Figure 3C seems to show an accumulation of fixes along the three edges in southwest–northeast direction. To study this quantitatively, the radar pixels of each bee were individually interpolated (see Section Methods), and for each resampled flight pixel, the closest edge in a Euclidean distance sense was determined (Figure 8A). The radar coverage was then partitioned in 21 zones: five distances ranges at most 10, 25, 50, or 100 m from their closest edge, plus the rest, see Figure 8B for the relative coverage of each range. Figure 8C exemplarily shows the path of Bee A05, color-coded according to these distance ranges; for the other bees, see Supplementary Data Sheet S11.

**Figure 9:**
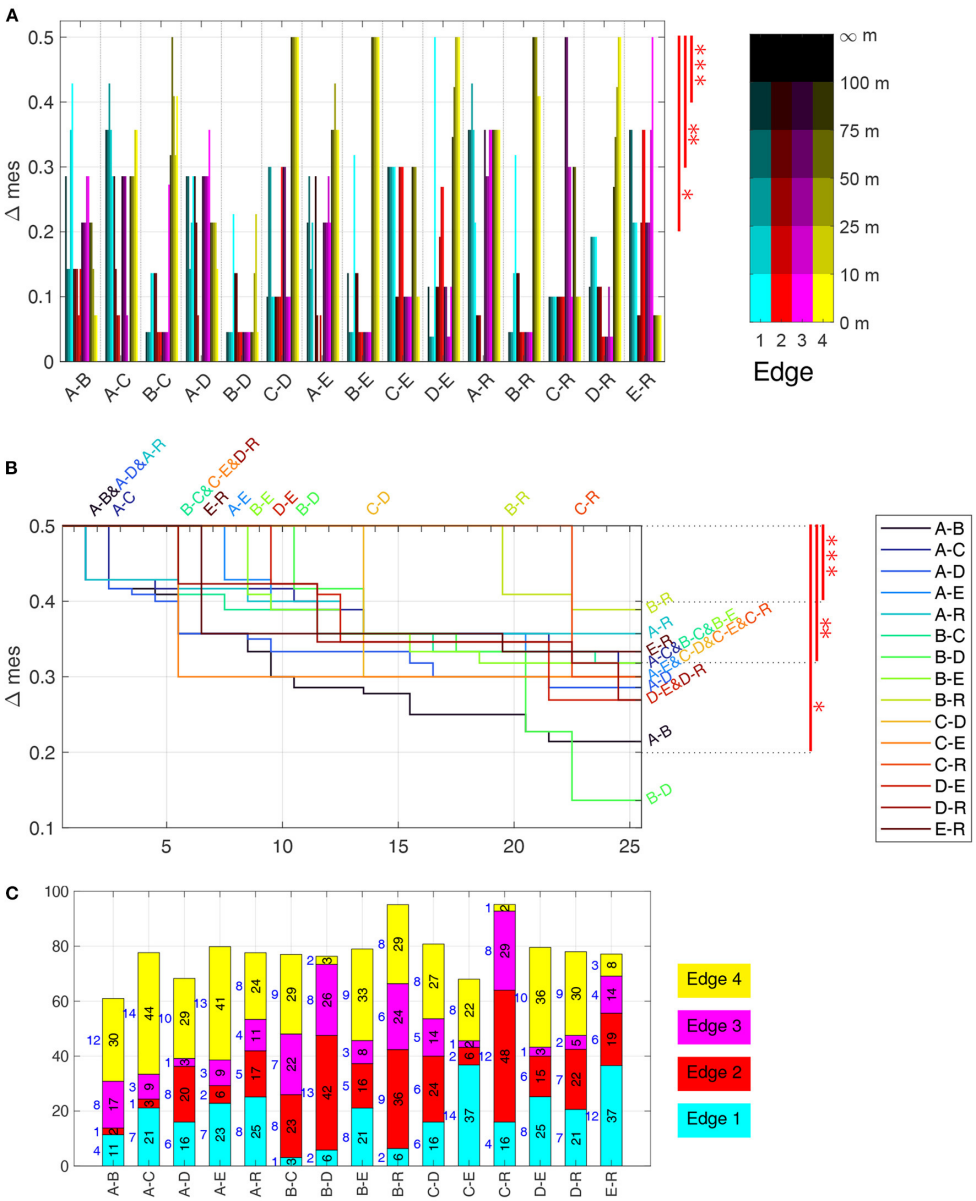
Discrimination analysis based on time and flight angle near edges. **(A)** Δmes for the time spent in each of 20 vicinities of the edges, sorted by pair of groups. The three significance levels are shown in red on the right. For example, Groups C–D can be discriminated very well, while B–D cannot using only time near edges. **(B)** Δmes for the 25 best discrimination measures for each pair of bee groups, combining time near edges as well as angles. Δmes values (y-axis), sorted in decreasing order, with index 1 to 25 on the x-axis. As not all lines are visible everywhere, start and end are specified above and to the right, respectively. The three significance levels are shown in red on the right. For example, Groups C–D can be discriminated extremely well with 13 measures at the maximal Δmes, but also B–D with 11 measures, which are all flight anle measurements, see **(A)**. **(C)** Number (in blue on the left of each bar) and sum (black and height of bar) of best Δmes values per group pair and edge of all discriminants shown in **(B)**. The height is scaled to 100 %, which corresponds to 12.5, i.e., 25 times the maximal Δmes of 0.5. Significance levels: ^***^ Δmes ≥ 0.4, ^**^ Δmes ≥ 0.3, ^*^ Δmes ≥ 0.2.

The distribution of the relative time a bee spent in a specific distance range to an edge shows a large heterogeneity among the test bees, while the model bees are much more homogeneous. Bees A differ from all others with respect to the time spent near Edge 1, the edge passing close to the release site, see blue bars in pairs that include group A such as A–B, or A–R in Figure 9A. As this figure only highlights the zones that allow for discrimination, the information which bees flew more in a zone can be found in Supplementary Data Sheet S12. Edge 3 is the opposite, bees from home area A spent significantly less time there than bees from other hives and also less than the model bees R. The latter also differ from Bees C and E as the model bees spent more time close to Edge 3. Edge 2 is useful for discriminating Bees E, as these bees spent significantly less time there as bees from hive C and D as well as the model bees R. Interestingly, there is a high heterogeneity among bees A, B, and C with respect to the amount of time spent near Edge 2. Edge 4, the bushes behind the radar, give quite some discrimination information, even though the bees flew relatively rarely there. The mes test shows significant differences between 11 out of 15 pairs. Only the pairs of bees A–B, C–E, C–R, and E–R show similar closeness. Overall, using all four edges allows for discriminating any pair of bee group, as for any pairs there is at least one bar above 0.2 in Figure 9A. This clearly indicates that bees from different home areas use edges differently in their search flights.

Figure 9 summarizes the mes-statistics for the pairwise discrimination using all 20 time near edges and the 120 flight angle near edges. The Δmes of all 20 time near edges for each pair of bee groups (Figure 9A) shows that Edge 4 is a very good discriminant for many pairs, i.e., B–C, C–D, A–E, B–E, D–E, B–R, and D–R, while Edge 3 discriminates well A–D and E–R, and Edge 1 the pairs A–B, A–C, and A–R. C–R can best be discriminated by Edge 3, especially by the distance range 75 m to 100 m. For C–E, the edges 1, 2, and 4 are equally discriminating with several Δmes = 0.3. Only B–D can only be discriminated at *, with only two features (close to Edge 1 and 4) showing an Δmes>0.2. To summarize, the near edge measurements allow in almost all cases for very good discrimination (of at least Δmes = 0.43, often even equal to the maximally attainable Δmes = 0.5) (Figure 9B). Only the pairs of bees A–D, B–D and C–E have lower values, with B–D the lowest at 0.23. The time spent near Edge 2 and 3 showed much less significant differences than near Edge 1 and 4 (Supplementary Data Sheet S12).

Often, the Δmes of a pair of group at various distances to one of the edge are similar or even equal, as e.g., for Edge 4 in C–D, B–E (Figure 9A). This is, however, not always the case as Edge 1 in B–E and D–E shows where the fixes close than 10 m have a discrimination of Δmes = 0.5 while all ranges further away from Edge 1 do not show a significant discrimination. In some cases, the ranges close to an edge show higher discrimination value, e.g., Edge 3 in E–R or the already mentioned case Edge 1 in B–E and D–E. In others, the ranges further away from the Edge are more discriminatory than the closer ones, see for example Edge 4 in C–E, Edge 3 in C–R, Edge 1 and E–R. These result clearly indicates a specificity of the home area groups with respect to the guidance effect of the edges. An interesting result of the edge analysis in Figure 9A uncovered that the statistically relevant zone is not only close to the edges, but also beyond the distance bees were shown to see them, i.e., approximately 30 m (Menzel et al., 2019).

Next, we studied the flight angle relative to the nearest edge. Within each distance range as above, we analyzed the frequency of the flight angle relative to the edge, discretized in six ranges of 15° width, i.e., 0° to 15° up to 75° to 90°. The 120 statistics for each bee, four edges à five distance ranges à six angle ranges, can be found in Supplementary Data Sheet S12. The flight angles allow for an even better discrimination, as each pair has at least one Δmes = 0.5, most pairs have several test cases maximally discriminating between them (see Figure 9B), which shows the best Δmes for any distance and angle feature. For any pair of two bee groups, more than 20 test cases exist having a Δmes>0.2, thus showing a significant difference. Also, all pairs can be discriminated with at least five test cases having a Δmes≥0.4. Two pairs are particularly distinctive: both B and C bees have 19 or more test cases that distinguishes them from the model bees R, at a Δmes = 0.5. This shows that their search strategies are very different from random searches and from equal guidance by any of the edges. While the other test bees can also be well discriminated from the R group, the number of test cases highly discriminating them from the R group is much lower. This is a notable result as the pairs A and B as well C and D seemed to behave similarly in the spatial distribution analysis of fixes (PLS analysis Figure 6, separatrices Figure 7).

Figure 9C highlights which edges are present in the top 25 discriminating test cases shown in Figure 9B. The bar heights are proportional to the cumulated Δmes per pair while the colored bar with the number to their left show how many of the 25 test cases are near one of the four edges, with Edge 1 in cyan at the bottom and Edge 4 in yellow at the top. All edges are present for each pair, however differences are clearly visible. For example, Edge 1 is not important for discriminating the pairs B–C and B–D, but plays a significant role for discriminating E from C, D, and R as well as A from C, E and R. Edge 2 and 3 are extremely important for the pairs B–D and C–R, and also much for B–C and E–R. Edge 4 plays a significant role for most pairs, with notable exception of the pairs B–D, C–R, and E–R.

## 3. Discussion

We have applied here generalization tests to characterize navigational memory taking advantage of the fact that honeybees are central place foragers that return frequently to their nest, the hive. Foraging bees were collected from hives located in different country sites, their explored home area. They were trained to a close feeding site, collected when leaving the feeder and transported to a common test site where they were released at the same release site. The home areas were selected such that they differed more or less from the test area. In particular, the home areas provided a more or less rich panorama, and the test area lacked a prominent panorama. The test area provided mostly elongated ground structures (called edges here) of rather simple geometry, and the home areas differed in a graded way in this respect. If the test bees would not, at least partially, generalize their site specific navigation memory, they are expected to apply a stereotypical search strategy and no differences would appear between bees from different home areas. Inexperienced bees or bees unfamiliar with a landscape perform multiple exploratory loops in different directions and over different distances (Capaldi et al., 2000; Menzel et al., 2005; Degen et al., 2015, 2018). Experienced bees released in an unexplored area also perform rather regular loops of search flights that have a number of common features. Bees fly in different directions over different distances during the outbound flight components and return to the release site multiple times (Menzel and Greggers, 2015). Thus, the lack of memorized guiding cues is thus likely to induce random search possibly similar to those of the well-studied desert ant *Cataglyphis* (Wehner and Srinivasan, 1981). In addition, stereotypical flights according to innate responses to landscape features may be apparent. In both cases no differences between bees of the different home areas are expected. Two models simulating random components of distance and direction were run, one that included all directions around the release site (Model S) and one whose fixes in the unscanned sector of the radar were excluded (Model R). Although random components certainly contribute to the search strategy of the test bees random search as the only or dominant strategy and the application of common strategies in response to landscape features and compass directions can be rejected on the result of multiple analyzes.

The results of our analyzes can be summarized as follows. The distribution of flight directions differs significantly between test bees and the random models as well as between bees from different home areas (Figure 4). The spatial distribution of flight fixes quantified by heat maps reveal significant differences between any two groups of test and model bees at specific heat map quadrants (Figure 5). A unified view was obtained by a partial least squares regression (PLS) analysis that uncovered structural differences in the flight fixes' spatial distribution. The three dominant PLS support vectors explain approx. 90 % of the heat map data variance. As PLS support vectors as well as hyperplanes separating the three dominant PLS support vectors of bee groups can be plotted as heat maps, the PLS analysis is also a highly informative way of visualizing these differences (Figures 6, 7). These hyperplanes separate bee groups very well, especially the model bees R from all test bees, but also all test bee groups from each other with very high significance. Most importantly, a gradient of similarity can be derived from these two hyperplanes (Figures 7B1, B2), with the appearance of three supergroups among the test bees: Groups A and B, C and D, as well as E (Figure 7B2). The analyzes did not take into consideration the edges of the test area. From a view point of existence or absence of edges and structured panorama, D and E home areas differ more strongly than A and B homes areas from the test area, and home area C lies in between (Figure 1). A and B home areas are characterized by elongated ground structures but in different ways (irrigation channel, rows of trees, compass direction). These differences are reflected in the preference of Edge 1 by A bees, less guidance by Edge 2 in B bees, and no attraction to Edge 4 for Groups A, B, and D. Their respective home areas were characterized by further distant panorama, border lines of agricultural fields and segments of elongated ground structures that differed quite considerably from those in the test area. The edges in the test area impact the search flights of the bees in each group differently (Figure 9). Quantifying the bee's proximity to the ground structures revealed that the bees of each groups differed significantly from each other in their flying behavior, both with respect to the time spent close to each edge (Figure 9A), as well as to the angle they flew to or away from these edges (Figure 9B). This effect was not only visible in close proximity to an edge, but also in the range of 75 m to 100 m, where the bees probably cannot see the edge. Groups B and C differed most from the random bees, see Figure 9B. Groups B differed mostly around Edge 2 to 4 from the model bees, for Group C only near Edges 2 and 3 as well as 1, but not Edge 4. A summary of the discrimination of bee groups using the edges is not as simple as for the heat maps. Nevertheless, it is obvious from Figure 9 that restricting the bee data to the proximity of the edges is sufficient to highly discriminate among any two pair of bee groups. This requires all four edges as for any edge there are pairs best discriminated using that edge, and other pairs, where a specific edge does not show discriminatory information.

Taken together, sole guidance by a random search strategy and the effect of stereotypical potentially innate guiding factors can be rejected. The heat map PLS analysis (Figures 6, 7) supports the conclusion that a similarity gradient based on the elongated ground structures guided the search flights. Bees from home area E that lacked any similarity with the test area behaved most closely to the modeled R random bees. Bees from home areas A and B behaved most different from modeled R random bees, and were close to each other. Bees from home area C and D were in between and were also rather close to each other. A detailed analysis of the effect of the edges on the different bee groups uncovered that the edges impacted the bees, both with respect to the time spent near an edge, as well as to their flight directions.

The latter finding is important because elongated ground structures might be attractive innately to bees, and flight directions might be bound to directions of elongated ground structures. Indeed, elongated ground structures are frequently used for guidance in honeybees (Menzel et al., 2019) and bumble bees (Brebner et al., 2021). These structures need to be learned (discussion in Menzel et al., 2019). Elongated ground structures like irrigation channels, rows of bushes or trees, edges of forest and a river bank characterized the home areas differently. Two irrigation channels and a borderline between two grasslands were the only elongated ground structure in the test area. A row of bushes and a parallel running small road as well as a parallel creek were in the south of the radar. Most of the flights beyond the row of bushes was, however, occluded by the radar blanking sector. The skyline of the horizon was even over most of the test area covered by the searching bees within the radar range (≤ 2° visual angle). This was not the case in any of the 5 home areas. Thus, the effect of panorama matching was not addressed in our experiments, but the lack of any panorama cue in the test area may have impacted the search flights of the test bees differently. Piloting toward a beacon can also be excluded because the only beacon in the test area was the radar antenna that was not approached. Preferred compass directions can also be excluded for both the earth magnetic field and the sun compass. In both cases such preferred directions would have to be detected in all 5 experimental groups. This applies also for the possibility that searching bees would prefer a constant angle to the sun azimuth because the experiments were performed between a constant time window (12:00 a.m. and 3 p.m.). We thus conclude that the home area specific effects indicate a generalization effect of navigation memory acquired in the respective home area.

Elongated ground structures are characterized by unique features for flying animals that make them most suitable as reference objects in mid-range navigation. They keep a stable relationship to a compass direction (von Frisch and Lindauer, 1954; Dyer and Gould, 1983), they are polarized in relation to the goal (leading to, leading away) and in relation to other localized landmarks, they may be identical to elongated landmarks at the goal, thus following it will lead the animal to the goal, they provide potentially a network of extended landmark features that characterize locations uniquely. These alignment effects has been well studied in human navigation (McNamara et al., 2003). In laboratory mammals, the alignment effect requires a functional hippocampus, possibly via boundary vector cells (Barry et al., 2006). Robots were found to use line-like landmarks for efficient navigation (Se et al., 2005; Furlan et al., 2007). Most importantly, flying animals identify such extended ground structures in a map-like aerial view making them highly attractive as guiding structures. It is thus not surprising that both bats and birds use linear landmarks for navigation (Heithaus et al., 1975; Biro et al., 2004; Lipp et al., 2004; Geva-Sagiv et al., 2015). ased on the data reported here we conclude that elongated ground structures are also salient components of the honeybees' navigation memory.

We have used here a generalization test procedure to explore the properties of landscape memory. We deal with immediate specific transfer in the navigation context, the ability of an animal to respond to partially learned and novel stimuli. Based on the rich literature about navigation in bees (see Introduction) we believe that the existence or absence of panorama and local cues, the kind of panorama and local cues as well as the existence, appearance and compass directions of edges are very well learned and discriminated by bees. However, this was not verified for the cues involved in the experiments reported here. In particular the learning and discrimination of isolated cues involved was not studied. Such experiments under natural conditions and within the dimensions of natural navigation training procedures are close to impossible because environmental features cannot be moved around systematically, separated or freely combined. This is unfortunate because learning about a stimulus or combinations of stimuli influences strongly how the behavior is generalized to other stimuli. In laboratory experiments different training procedures are used to distinguish between the animal's ability to discriminate, and subsequently generalize to other stimuli (Blough, 1975; Kehoe, 2008). Still, generalization tests are highly informative because it is reasonable to assume that self-training and exploratory learning under natural conditions will be rather similar and close to optimal across different animals of the same colony that are exposed to the same environment, and animals of different home areas will have different navigation memories. The ability to generalize (rather than to discriminate/not discriminate) is thus a key component of cognition bound to phenomena like selective attention, expectation, categorization, similarity judgments and saving (Rescorla, 1992; Blaisdell, 2008; Zentall et al., 2008). “Generalization occurs in learning, and is essential for deriving knowledge from experiences and for skills of all kinds. It is the basis of predicting future situations from past experience and for drawing analogies” (Gregory and Zangwill, 1987 p. 284). Thus, generalization speaks to the cognitive dimensions of using memory for solving a problem.

We conclude from the analyzes above that bees from the different home areas were guided in the test area by a generalization effect of their navigational memory. Although we have focused on the elongated ground structures the parameters of guidance are not as obvious. We argued that the generalization process may motivate the bees to explore some landscape features in the test area more intensively, and indeed the local density of exploration reflects a graded generalization effect to landscape features in the test area (elongated ground structures). However, the lack of learned features will certainly also play an important role. Not existing landscape features in the test area (structured horizon, rising objects, their spatial distribution and other features) cannot be explored. The amount of guiding effect of such structures in their home area will potentially add to generalization by hidden dissimilarity effects.

The level of cognition reached by the navigating bee has to be studied along two lines of questions, (1) do bees expect landscape features such that they travel novel short cuts and freely change their states of motivation, cognitive properties assigned to a cognitive map (Tolman, 1948), (2) do bees compose their multiple experiences and thus multiple memories about the pattern of landscape such that they form a spatial schema, a high order cognitive process well documented for rodents and humans (Richards et al., 2014; Farzanfar et al., 2023). The first line of question has been addressed multiple times in honeybee navigation and strong evidence has been found in favor of a cognitive map (Wang et al., 2022; Menzel, 2023). The procedure of a generalization test as presented here will speak to the second line of question if multiple repetitions of pairs of home areas and test areas are tested helping to uncover categorization and reference to generalize spatial representations.

## 4. Methods

The experiments were carried out close to the village Klein Lüben (Brandenburg, Germany). The area is characterized by open grass land, agricultural land, forest, creeks, irrigation channels, small roads and the river Elbe. The harmonic radar for flight tracking in the test area was placed at the coordinates 52°58′31.14′′N 11°50′11.35′′E. The test bees were released at the Site R (Figure 1F 52°58′36.37′′N 11°50′13.37′′E). Five colonies of *Apis mellifera carnica* were positioned in five different areas (home sites Figures 1A–E) that differed with respect to the respective landscape structures. Hive A was located at 52°59′07.94′′N 11°49′05.34′′E, 1.64 km NW of Release Site R, and Hive B at 52°58′53.18′′N 11°48′30.87′′E, 2.1 km E of R. Both areas consisted of predominantly agricultural fields, a road, and irrigation channels. Hive A was close to a row of high rising poplar trees, and Hive B close to isolated low trees. The home area around Hive C (52°57′22.23′′N 11°51′42.29′′E, 2.89 km SW of R) was characterized predominantly by open meadows with irrigation channels and an edge of a small forest in the N. Hive D (53°00′08.98′′N 11°52′17.75′′E, 2.21 km NE of R) was located in the middle of a large forest with a SE–NW stretching forest aisle. Hive E (52°58′46.02′′N 11°46′15.86′′E) was placed at the bank of the river Elbe overlooking a country side with scattered trees. The distance between Hive E and the radar location was 4.55 km. The river was between 350 and 450 m wide during the years 2009 and 2010. In 2011 the river flooding lead to a width of > 10.00 m of open water. Figure 1 gives an impression about the similarity/difference of the elongated ground structures in the test area and the 5 home areas. Notice that the layout of the ground structures in the test area was more similar to that in home areas A and B than to C–F and that home areas E and F were very different between each other and the test area.

The experiments were carried out in the summers (July to September) of the years 2009, 2010 and 2011. The colonies were placed at the respective sites at least 4 weeks before the experiments started, ensuring that only foragers were tested that were familiar with the home area and had not experienced any other area. Foragers of each colony were trained to a feeding site very close to the respective hive (< 7 m). Single foragers were then collected at the feeder after they had sucked their fill, transported in a dark box to the test area within < 30 min, equipped with a transponder for tracking their flight with harmonic radar and released at the release site R in the test area (Figure 1F). All test bees from the 5 colonies were released at the same respective site R. The test area was characterized by two irrigation channels (IRC) NW and NE of the release site R. They ran parallel to each other at a distance of 611 m stretching from SW to NE. In addition, a borderline between two grasslands mown at different times ran parallel to the IRC, roughly in the middle of the large and otherwise rather evenly structured grassland. Release site R was located close to this borderline. Additionally, several rather weak ground structures characterized the test area: lines in the grass running parallel to the borderline originated from mowing of grass, patches of grass growing at different height, and a dip in the ground running through most of the test area from SW to NE contrasting grassland with different grass species. The test area's border running SW–SE was characterized by a row of bushes (at the closest 30 m SE of the radar station) running parallel to a small road and a creek. There were no trees or other high rising structures within 1 km of the radar other than the radar station itself (height 6 m) and the row of bushes behind the radar. The home areas of the 5 hives were selected to either resemble partially the elongated ground structures in the test area (e.g., the two IRC, the border between two grasslands and the row of bushes) or to be different in various respect (rising close or/and far landmarks) from the landscape structure of the test area. The search flights of the bees were tracked from the radar fixes collected every 3 s with a customs made program that converted the fixes from the circular display to a Cartesian map (Cheeseman et al., 2012). Finally, the fixes were imported intoMatlab and overlaid for visualization purposes to test site's Google map. The map was also used to quantify the landscape features in the test area.

The release site in the test area was chosen such that most of the search behavior could be monitored as indicated by the finding that during most of a test flights the respective bee was seen on the radar screen. Between 30 and 50% of the bees (depending on home area) left the scanned area for short periods. Most of them returned to the release site. No information was available where the bees from the different home areas had foraged. No spots of dense forage (e.g., flowering tree or bush) were found within the natural range (up to 2 km radius) of the respective hives during the experimental periods (July, August, September). We, therefore, assume that bees foraged on widely scattered flowers, and thus the individual bees from the different hives may have foraged in different directions and over different areas from their respective hive. The colonies were regularly inspected and ample food store (pollen and nectar) was found in all cases. The test area was the most even and spacious pastry we could find (and where the farmers allowed us to work). The flat and horizontal level lacked any obstacle besides the row of bushes, an ideal condition for harmonic radar tracking. The skyline was flat within ≤ 2° visual angle around the release site over a large proportion of the area within which the test bees performed their search flights (Menzel et al., 2005). This applies to all of the area scanned by the radar besides the area up to 200m NE from the row of bushes behind the radar.

We tested a total number of 50 bees leading to one flight trajectory each. Each animal was released only once, because no animal could be recaptured. The numbers of tested animals (*N*) and the total number of radar fixes (*n*) were for the different colonies: home area A *N* = 14, *n*= 6.098, home area B *N* = 11, *n*= 4128, home area C *N* = 5, *n*= 1.722, home area D *N* = 13, *n* = 4.611, home area E *N* = 7, *n*= 2.496.

### 4.1. Radar tracking

Tracking bees with a harmonic radar was achieved as described in (Cheeseman et al., 2012). We used a system with a sending unit consisting of a 9.4 GHz radar transceiver (Raytheon Marine GmbH, Kiel, NSC 2525/7 XU) combined with a parabolic antenna providing approximately 44 dBi. The transponder fixed to the thorax of the bee consisted of a dipole antenna with a Low Barrier Schottky Diode HSCH-5340 of centered inductivity. The second harmonic component of the signal (18.8 GHz) was the target for the radar. The receiving unit consisted of an 18.8 GHz parabolic antenna, with a low-noise pre-amplifier directly coupled to a mixer (18.8 GHz oscillator), and a downstream amplifier with a 90 MHz ZF-Filter. A 60 MHz ZF-Signal was used for signal recognition. The transponder had a weight of 10.5 mg and a length of 12 mm. We used a silver or gold wire with a diameter of 0.3 mm and a loop inductance of 1.3 nH. The range of the harmonic radar was 900 m. Several limitations of the applied methods and the test conditions need to be mentioned. The range of the radar was limited to 900 m radius and did not scan through an angle of 150° to the SW due to the switch-off of the radar beam (radar blanking) and a row of bushes along a small road. Therefore, data about the searching behavior were available only within a limited sector (210°).

### 4.2. Random bees

Based on a modified version of the ant model by Wehner and Srinivasan (1981), we developed a model of an artificial bee that explores the surrounding of its release site with a flight pattern that is independent of the landscape. Starting at the release in a random direction, the “bees” fly 10.000 steps of unit length with heading changes from step to step as specified by random numbers generated as the arctangent of a normal distribution (zero mean, standard deviation 0.25) and filtered by a tenth-order low-pass digital Butterworth filter with normalized cut-off frequency 0.05. Whenever the distance to the release site is larger than 1+*k*/10 unit lengths, with *k* = 1, 2, … the index of the flight loop, the heading angle is rotated by an extra 180°. This rotation is also added when the simulated bees comes closer than 0.1 unit length to the release site. This raw path is then smoothed by a moving average filter of length 25. Finally, comparing 1.000 simulated paths to those of all real bee, the unit length was fitted to 2.22 m to achieve the same median distance from the release site as in the real flight data. The model bees are labeled S when their path include the sector not covered by the radar (radar blanking), and labeled R when only considering the circle segment covered by the radar, Figure 3C for an example.

One thousand random bees were simulated for quantifying the scaling, but in the analyzes, only 16 random bees (with and without radar blanking) were used to have a similar number as test bees per group.

### 4.3. Computations

The calculations were performed, except noted otherwise, with Matlab (R2020b) and its toolboxes “Statistics and Machine Learning” and “Image Processing.” The radar fixes were available to Matlab in radar-based Cartesian coordinates. They were then transformed to release-site coordinates, both in Cartesian as well as polar coordinates.

#### 4.3.1. Statistical approach

Figure 2 illustrates the procedure used in this study. The data were analyzed using the Kruskal-Wallis test (only in the appendices) and the Measure of Effect Size (mes) based on Cohen's U3 test for two samples (Cohen, 1988) as implemented by Hentschke and Stüttgen (2011). Classical significance tests, e.g., null hypothesis significance testing, depend on sample size, while effect size does not. This mes compares two populations, A and B, and returns the percentage of population A that lies above the median of population B. Thus, the mes always lies in the interval [0, 1] as 100 % = 1, and mes = 0.5 corresponds to equal medians. The further away a mes is from 0.5, the more significant the two populations differ. This difference is denoted by Δ_mes_ where Δ_mes_ = |mes − 0.5|. According to Hentschke and Stüttgen (2011), mes is able to uncover important aspects of the data that standard null hypothesis significance testing does not make visible.

For determining significant differences between pairwise groups, we applied Kruskal-Wallis significance tests (Kruskal and Wallis, 1952) (only shown in the appendices) and the Measures of Effect Size (mes) based on Cohen's U3 test (Cohen, 1988), the latter using the toolbox “Measures of Effect Size” by Hentschke and Stüttgen, Version 1.6.1 (Hentschke and Stüttgen, 2011).

#### 4.3.2. Heat map and edge analyzes

For the heat map and edge analyzes, the fixes were resampled by linear interpolation to obtain 10 additional points equally distanced between fixes. The heat maps were obtain by first smoothing the resampled data points using a 2D moving average filter of kernel size 50 × 50 m, thus smoothing them up to a chessboard distance of 50 m. For the edge analysis, the Euclidean distance from each landscape feature to each of the resampled fixes was calculated in order to determine the closest landscape feature, the distance to it as well as the angle between the closest landscape feature and the flight direction. These data was then statistically analyzed. The partial least square regression was calculated by Matlab's plsregress, the minimum volume ellipsoids were obtained by MinVolEllipse (on Matlab Central, by Moshtagh, 2009). For obtaining the separating hyperplanes, called separatrices, these ellipsoids were resampled with 441 points, the optimal separatrix was calculated using Matlab's fitcsvm with linear kernel functions, which is using a support vector machine approach. The distance between ellipsoids was obtained with the help of the Ellipsoid Toolbox (Kurzhanskiy and Varaiya, 2006), which uses YALMIP (Löfberg, 2004). Further functions from Matlab Central: maxdistcolor by Cobeldick (2020), distinguishable_colors by Holy (2023), plotBarStackGroups by Bollig (2011).

#### 4.3.3. Boxplots

Boxplots are used to illustrate distributions, and are particularly useful for non-Gaussian ones. The red line depicts the median, the second quartile, while the blue box goes from the first to the third quartile, i.e., covers half the data. The box height is called inter quartile range, IQR. The notch on each box, extending $1.57/\sqrt{n}\text{IQR}$ above and below the median, with *n* the number of data points, gives the 95 % confidence interval for the median (McGill et al., 1978). The lines up and down, called whiskers, extend from the box up to the last data points that is not further than 1.5 IQR from the first or the third quartile. The data points further away are outliers and marked by a plus sign.

## Data availability statement

The datasets presented in this study can be found in online repositories. The names of the repository/repositories and accession number(s) can be found below: https://osf.io/ygbde/.

## Author contributions

UG and RM: conceptualization. EB: statistical analyzes. EB and RM: writing. All authors contributed to the article and approved the submitted version.

## Supplementary material

Supplementary Data Sheet S1 Primary data.

Supplementary Data Sheet S2 Directional analysis.

Supplementary Data Sheet S3 Circle statistics.

Supplementary Data Sheet S4 Heat maps.

Supplementary Data Sheet S5 Significance analyses of heat map tiles.

Supplementary Data Sheet S6 Pairwise comparison of bee groups wrt. heat maps.

Supplementary Data Sheet S7 Significant tiles for comparing one bee group to all others.

Supplementary Data Sheet S8 PLS result in 3D.

Supplementary Data Sheet S9 PLS results as heat maps.

Supplementary Data Sheet S10 PLS separatices as heat maps.

Supplementary Data Sheet S11 Flight paths near edges.

Supplementary Data Sheet S12 Significance analyses of time and flight angle near edges for all bee groups.
