# Supplementary material for: Generalization of navigation memory in honeybees
Source: Front Behav Neurosci. 2023 Mar 6;17:1070957. doi: 10.3389/fnbeh.2023.1070957 (PMC10025308; doi:10.3389/fnbeh.2023.1070957)
Supplement: Supplementary Data Sheet S2 — Directional analysis. [file Data_Sheet_2.pdf]

Generalization of Navigation Memory in Honeybees

Supplement Data Sheet 02: Directional Analysis

Eric Bullinger*
Otto-von-Guericke-Universität Magdeburg
Institut für Automatisierungstechnik
Universitätsplatz 2, 39106 Magdeburg, Germany
eric.bullinger@ovgu.de

Uwe Greggers & Randolf Menzel*
Freie Universität Berlin
Neurobiologie
Königin Luisestr. 1 -3, 14195 Berlin, Germany
menzel@neurobiologie.fu-berlin.de

14 February 2023

- Direction W: p. 2
- Direction WSW: p. 2
- Direction SW: p. 3
- Direction SSW: p. 3
- Direction S: p. 4
- Direction SSE: p. 4
- Direction SE: p. 5
- Direction ESE: p. 5
- Direction E: p. 6
- Direction ENE: p. 6
- Direction NE: p. 7
- Direction NNE: p. 7
- Direction N: p. 8
- Direction NNW: p. 8
- Direction NW: p. 9
- Direction WNW: p. 9

*corresponding author

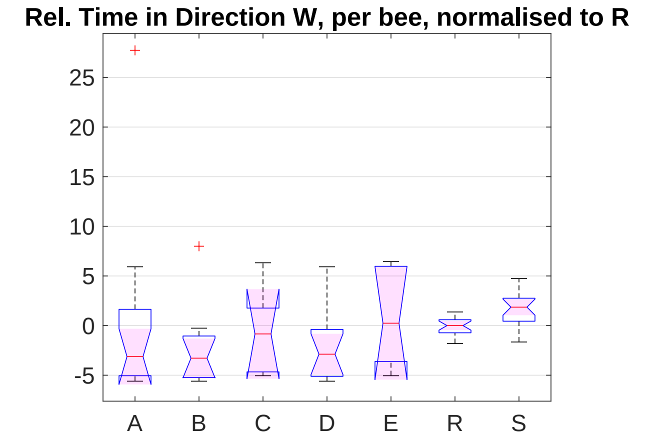
Rel. Time in Direction W, per bee, normalised to R
25
20
15
10
5
0
-5
A B C D E R S

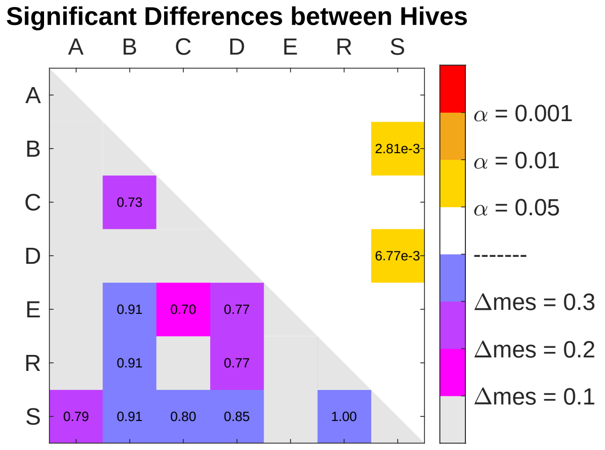
Significant Differences between Hives
A B C D E R S
2.81e-3
0.73
6.77e-3
0.91 0.70 0.77
0.91 0.77
0.79 0.91 0.80 0.85 1.00
α = 0.001
α = 0.01
α = 0.05

Δmes = 0.3
Δmes = 0.2
Δmes = 0.1

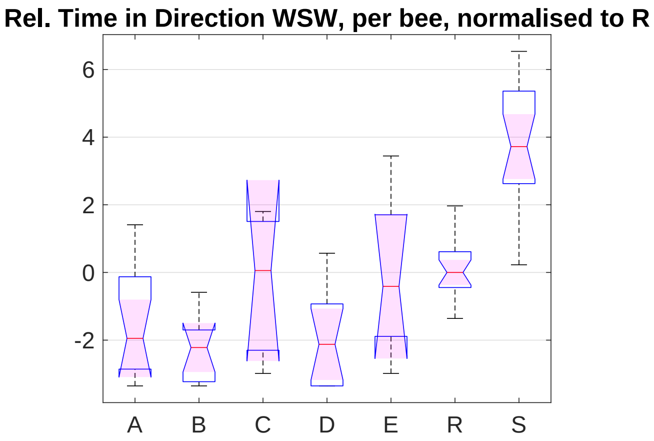
Rel. Time in Direction WSW, per bee, normalised to R
6
4
2
0
-2
A B C D E R S

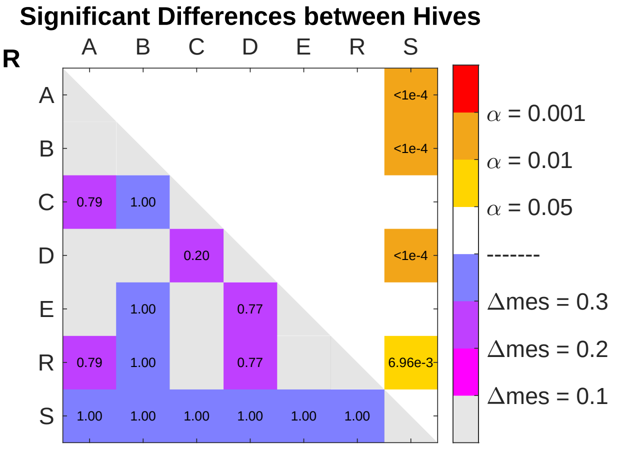
Significant Differences between Hives
A B C D E R S
<1e-4
<1e-4
0.79 1.00
0.20 <1e-4
1.00 0.77
0.79 1.00 0.77 6.96e-3
1.00 1.00 1.00 1.00 1.00 1.00
α = 0.001
α = 0.01
α = 0.05

Δmes = 0.3
Δmes = 0.2
Δmes = 0.1

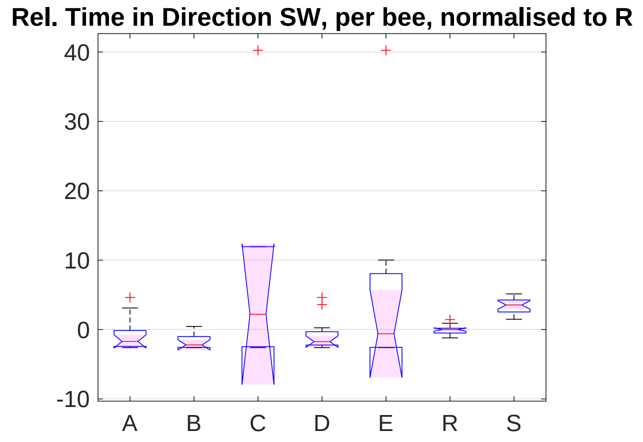
Rel. Time in Direction SW, per bee, normalised to R
40
30
20
10
0
-10
A B C D E R S

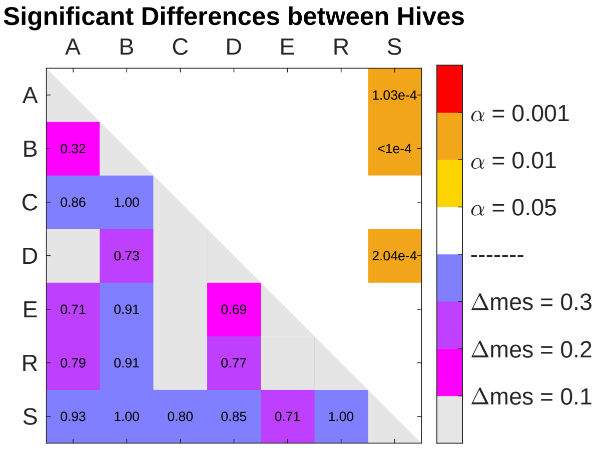
Significant Differences between Hives
A B C D E R S
1.03e-4
0.32 <1e-4
0.86 1.00
0.73 2.04e-4
0.71 0.91 0.69
0.79 0.91 0.77
0.93 1.00 0.80 0.85 0.71 1.00
α = 0.001
α = 0.01
α = 0.05

Δmes = 0.3
Δmes = 0.2
Δmes = 0.1

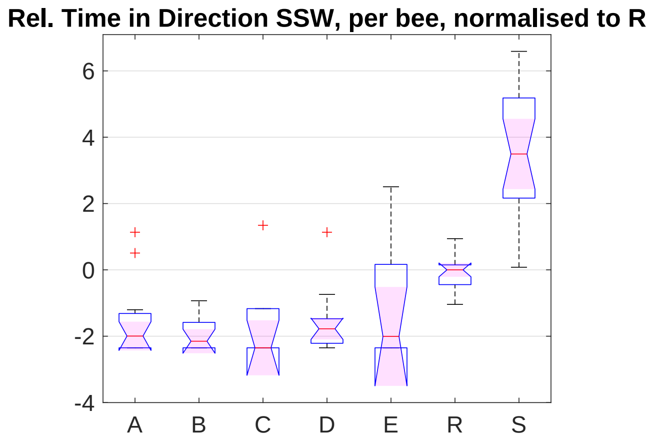
Rel. Time in Direction SSW, per bee, normalised to R
6
4
2
0
-2
-4
A B C D E R S

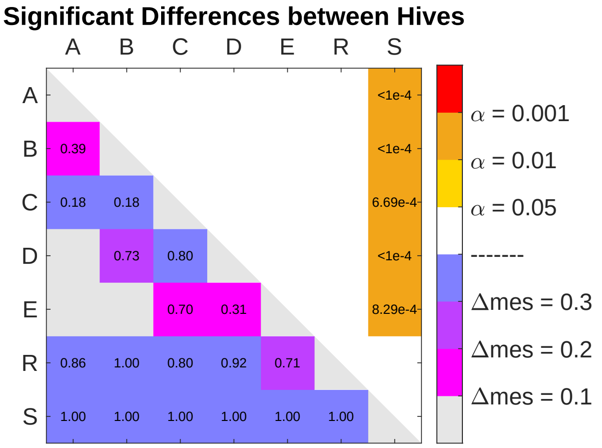
Significant Differences between Hives
A B C D E R S
<1e-4
0.39 <1e-4
0.18 0.18 6.69e-4
0.73 0.80 <1e-4
0.70 0.31 8.29e-4
0.86 1.00 0.80 0.92 0.71
1.00 1.00 1.00 1.00 1.00 1.00
α = 0.001
α = 0.01
α = 0.05

Δmes = 0.3
Δmes = 0.2
Δmes = 0.1

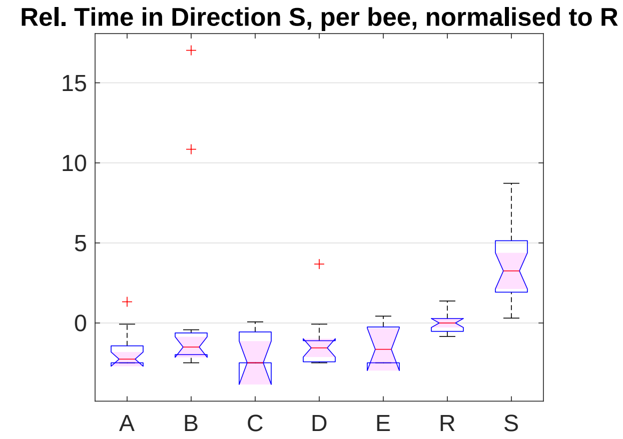
Rel. Time in Direction S, per bee, normalised to R
15
10
5
0
A B C D E R S

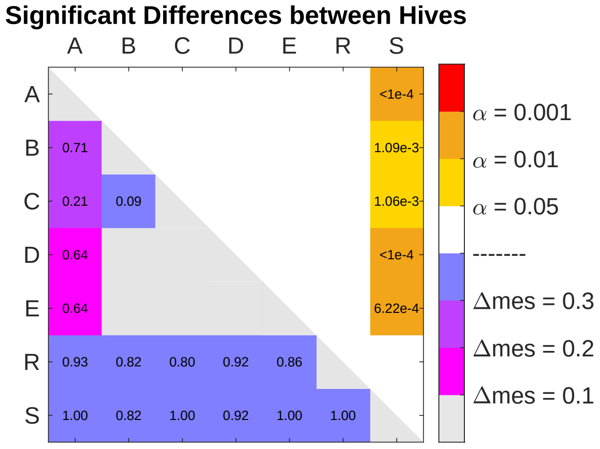
Significant Differences between Hives
A B C D E R S
<1e-4
0.71
1.09e-3
0.21 0.09
1.06e-3
0.64
<1e-4
0.64
6.22e-4
0.93 0.82 0.80 0.92 0.86
1.00 0.82 1.00 0.92 1.00 1.00
α = 0.001
α = 0.01
α = 0.05

Δmes = 0.3
Δmes = 0.2
Δmes = 0.1

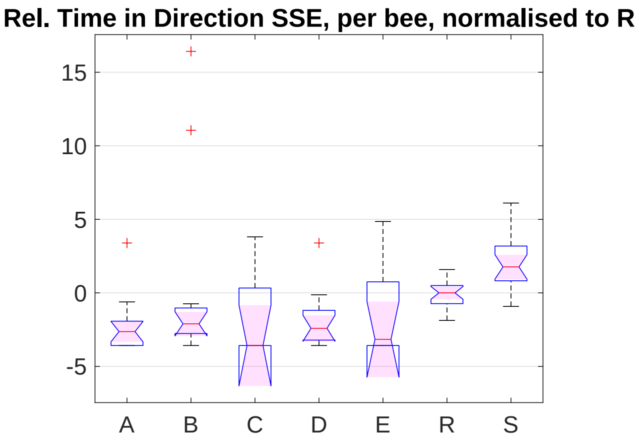
Rel. Time in Direction SSE, per bee, normalised to R
15
10
5
0
-5
A B C D E R S

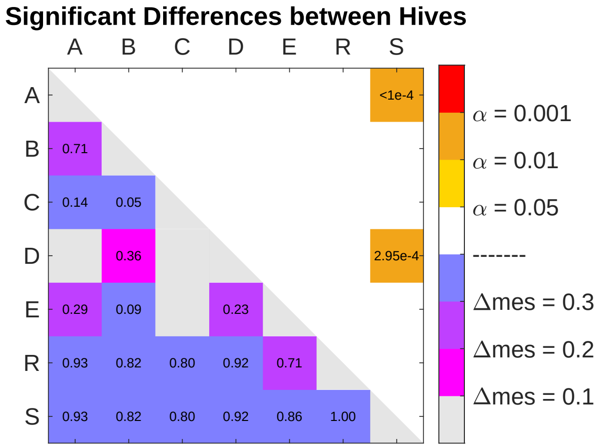
Significant Differences between Hives
A B C D E R S
<1e-4
0.71
0.14 0.05
0.36
2.95e-4
0.29 0.09 0.23
0.93 0.82 0.80 0.92 0.71
0.93 0.82 0.80 0.92 0.86 1.00
α = 0.001
α = 0.01
α = 0.05

Δmes = 0.3
Δmes = 0.2
Δmes = 0.1

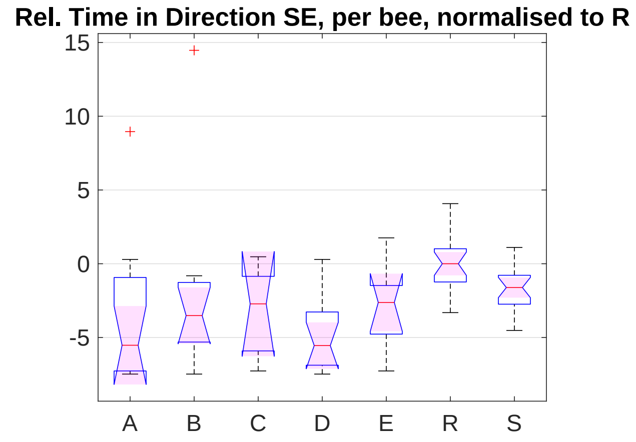
Rel. Time in Direction SE, per bee, normalised to R
15
10
5
0
-5
A B C D E R S

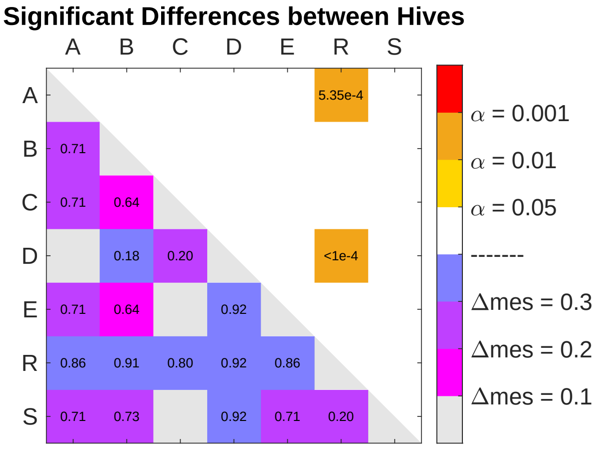
Significant Differences between Hives
A B C D E R S
5.35e-4
0.71
0.71 0.64
0.18 0.20 <1e-4
0.71 0.64 0.92
0.86 0.91 0.80 0.92 0.86
0.71 0.73 0.92 0.71 0.20
α = 0.001
α = 0.01
α = 0.05

Δmes = 0.3
Δmes = 0.2
Δmes = 0.1

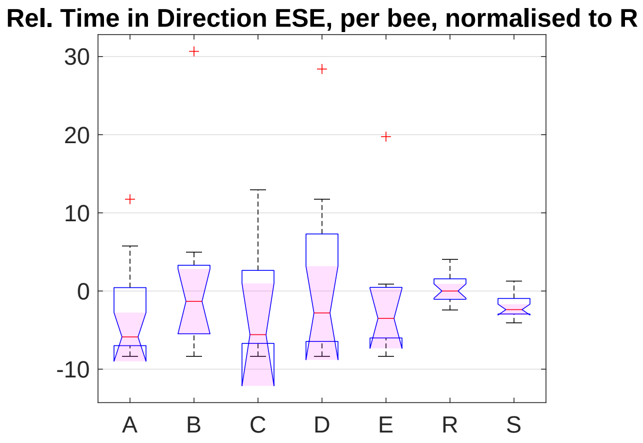
Rel. Time in Direction ESE, per bee, normalised to R
30
20
10
0
-10
A B C D E R S

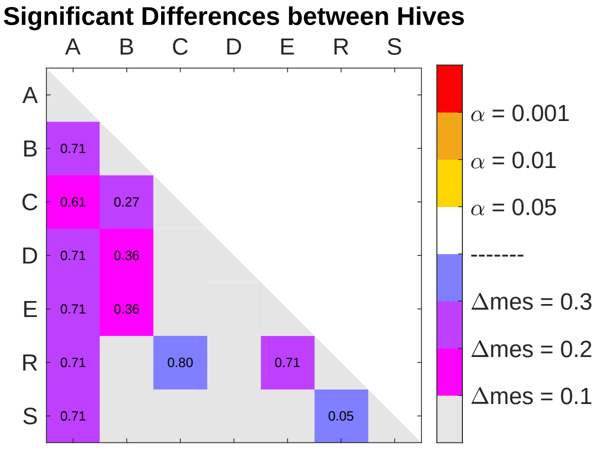
Significant Differences between Hives
A B C D E R S
0.71
0.61 0.27
0.71 0.36
0.71 0.36
0.71 0.80 0.71
0.71 0.05
α = 0.001
α = 0.01
α = 0.05

Δmes = 0.3
Δmes = 0.2
Δmes = 0.1

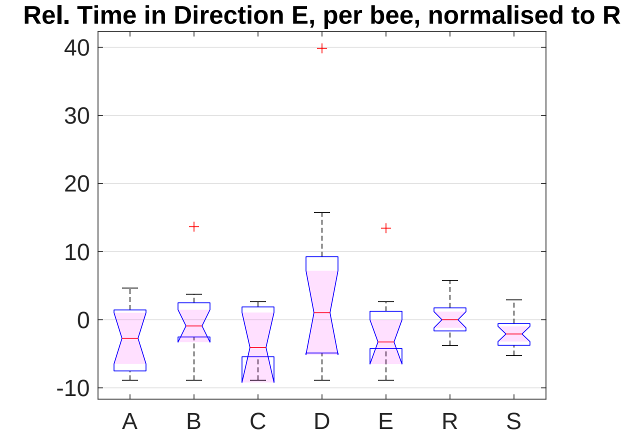
Rel. Time in Direction E, per bee, normalised to R
40
30
20
10
0
-10
A
B
C
D
E
R
S

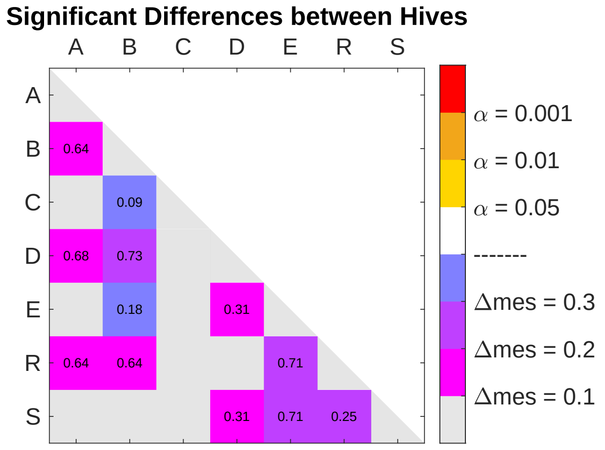
Significant Differences between Hives
A B C D E R S
A
B
C
D
E
R
S
0.64
0.09
0.68 0.73
0.18 0.31
0.64 0.64 0.71
0.31 0.71 0.25
α = 0.001
α = 0.01
α = 0.05

Δmes = 0.3
Δmes = 0.2
Δmes = 0.1

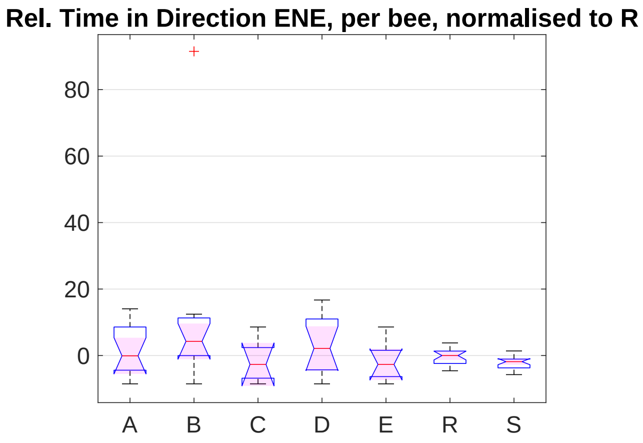
Rel. Time in Direction ENE, per bee, normalised to R
80
60
40
20
0
A
B
C
D
E
R
S

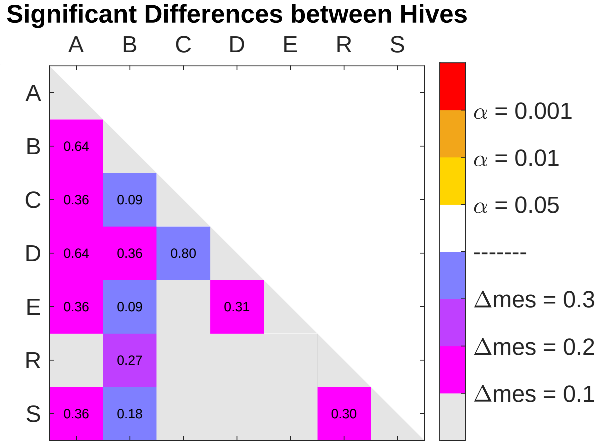
Significant Differences between Hives
A B C D E R S
A
B
C
D
E
R
S
0.64
0.36 0.09
0.64 0.36 0.80
0.36 0.09 0.31
0.27
0.36 0.18 0.30
α = 0.001
α = 0.01
α = 0.05

Δmes = 0.3
Δmes = 0.2
Δmes = 0.1

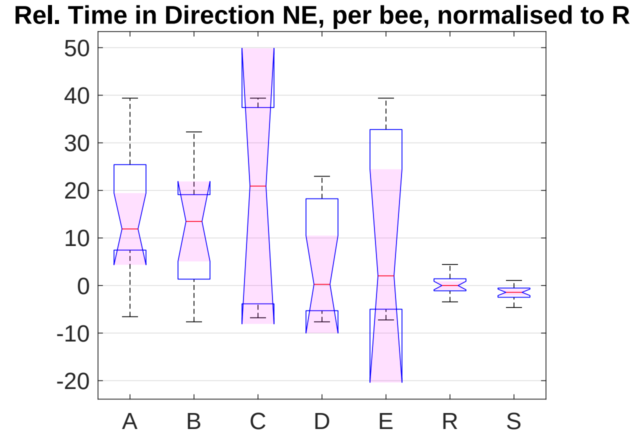
Rel. Time in Direction NE, per bee, normalised to R
50
40
30
20
10
0
-10
-20
A B C D E R S

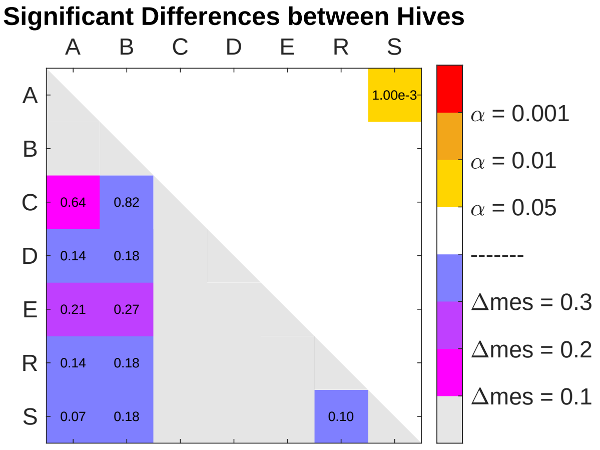
Significant Differences between Hives
A B C D E R S
1.00e-3
0.64 0.82
0.14 0.18
0.21 0.27
0.14 0.18
0.07 0.18 0.10
α = 0.001
α = 0.01
α = 0.05

Δmes = 0.3
Δmes = 0.2
Δmes = 0.1

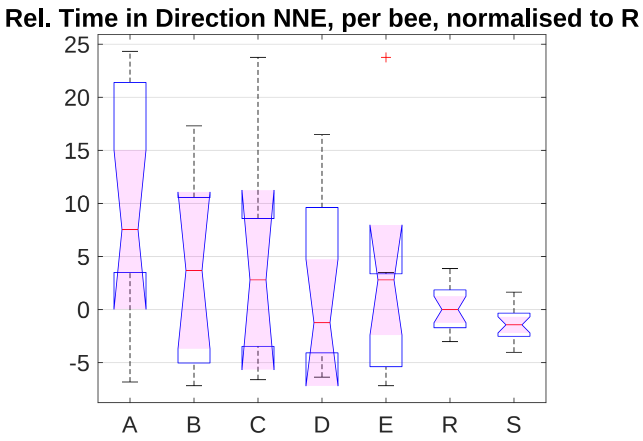
Rel. Time in Direction NNE, per bee, normalised to R
25
20
15
10
5
0
-5
A B C D E R S

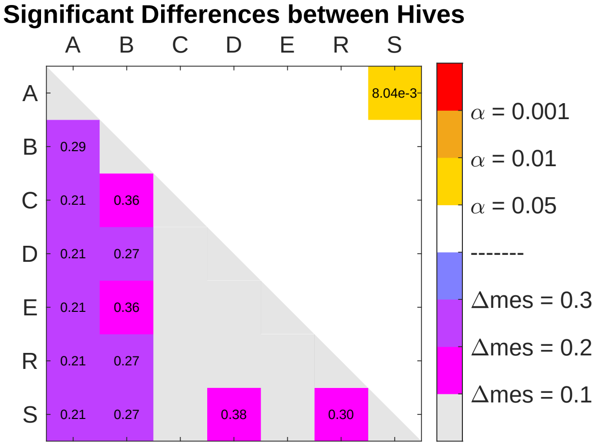
Significant Differences between Hives
A B C D E R S
8.04e-3
0.29
0.21 0.36
0.21 0.27
0.21 0.36
0.21 0.27
0.21 0.27 0.38 0.30
α = 0.001
α = 0.01
α = 0.05

Δmes = 0.3
Δmes = 0.2
Δmes = 0.1

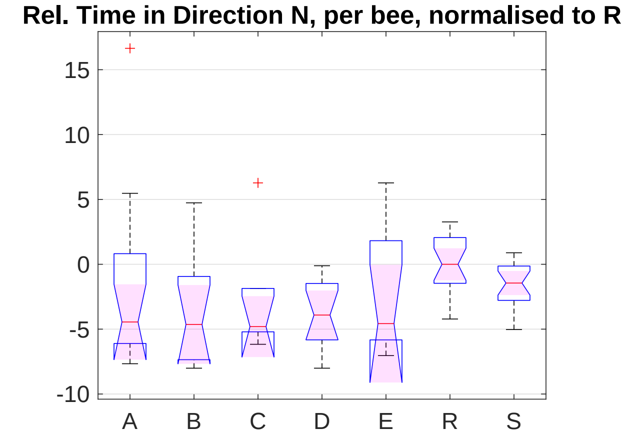
Rel. Time in Direction N, per bee, normalised to R
15
10
5
0
-5
-10
A B C D E R S

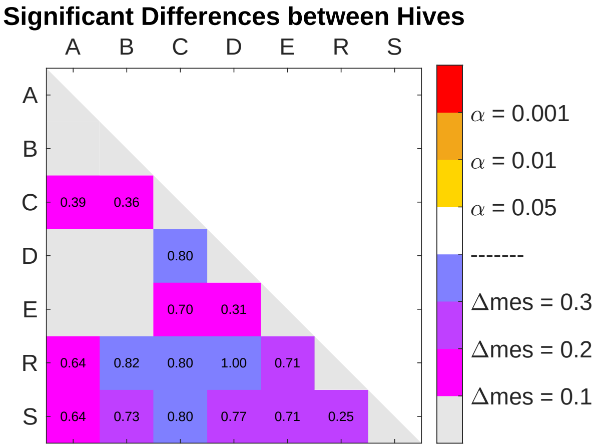
Significant Differences between Hives
A B C D E R S
0.39 0.36
0.80
0.70 0.31
0.64 0.82 0.80 1.00 0.71
0.64 0.73 0.80 0.77 0.71 0.25
α = 0.001
α = 0.01
α = 0.05

Δmes = 0.3
Δmes = 0.2
Δmes = 0.1

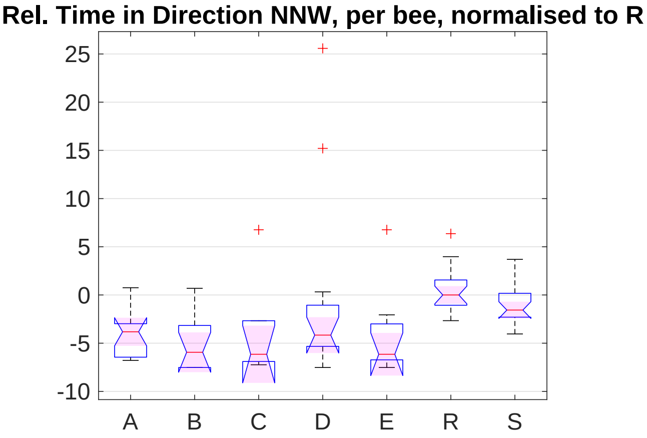
Rel. Time in Direction NNW, per bee, normalised to R
25
20
15
10
5
0
-5
-10
A B C D E R S

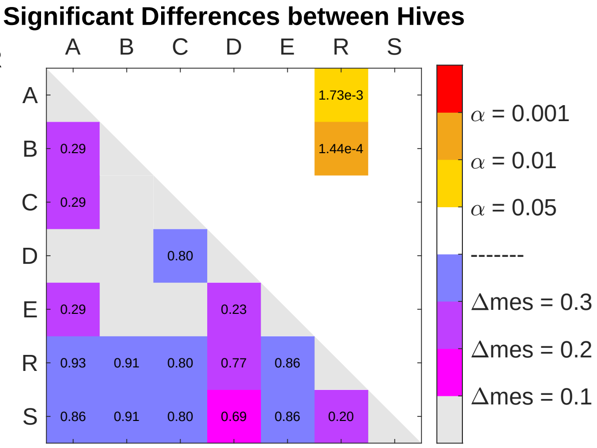
Significant Differences between Hives
A B C D E R S
1.73e-3
0.29 1.44e-4
0.29
0.80
0.29 0.23
0.93 0.91 0.80 0.77 0.86
0.86 0.91 0.80 0.69 0.86 0.20
α = 0.001
α = 0.01
α = 0.05

Δmes = 0.3
Δmes = 0.2
Δmes = 0.1

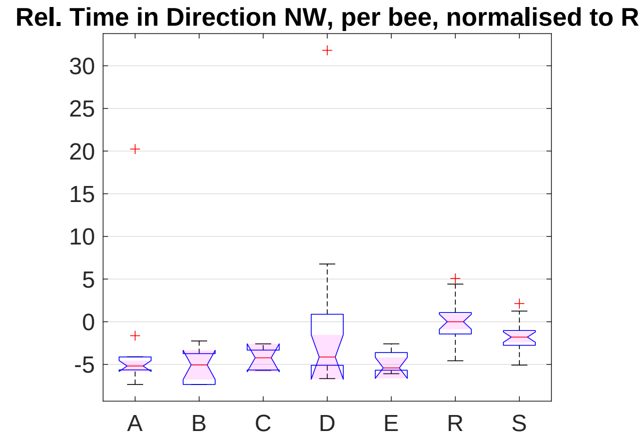
Rel. Time in Direction NW, per bee, normalised to R
30
25
20
15
10
5
0
-5
A B C D E R S

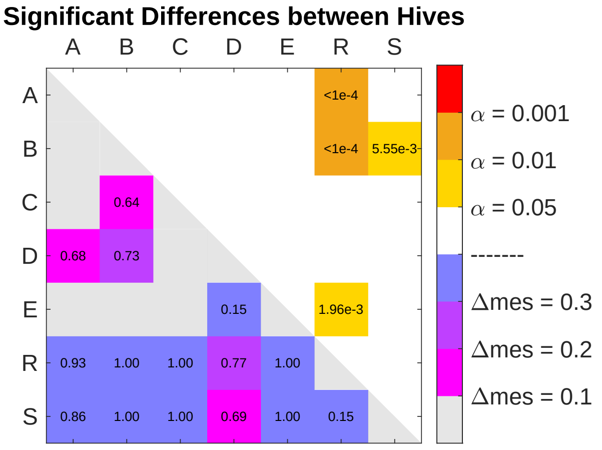
Significant Differences between Hives
A B C D E R S
<1e-4
<1e-4
5.55e-3
0.64
0.68
0.73
0.15
1.96e-3
0.93
1.00
1.00
0.77
1.00
0.86
1.00
1.00
0.69
1.00
0.15
α = 0.001
α = 0.01
α = 0.05

Δmes = 0.3
Δmes = 0.2
Δmes = 0.1

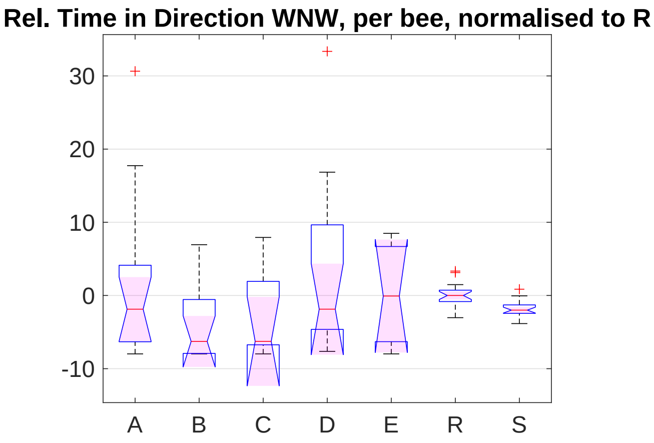
Rel. Time in Direction WNW, per bee, normalised to R
30
20
10
0
-10
A B C D E R S

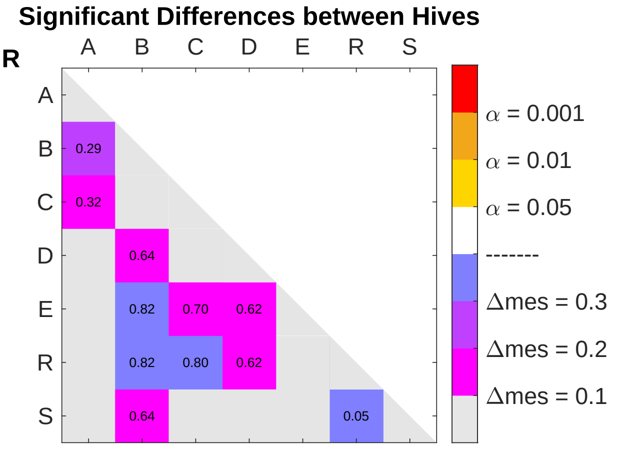
Significant Differences between Hives
A B C D E R S
0.29
0.32
0.64
0.82
0.70
0.62
0.82
0.80
0.62
0.64
0.05
α = 0.001
α = 0.01
α = 0.05

Δmes = 0.3
Δmes = 0.2
Δmes = 0.1
