# Supplementary material for: Generalization of navigation memory in honeybees
Source: Front Behav Neurosci. 2023 Mar 6;17:1070957. doi: 10.3389/fnbeh.2023.1070957 (PMC10025308; doi:10.3389/fnbeh.2023.1070957)

GENERALIZATION OF NAVIGATION MEMORY IN HONEYBEES

SUPPLEMENT DATA SHEET 03: CIRCLE STATISTICS

Eric Bullinger*
Otto-von-Guericke-Universität Magdeburg
Institut für Automatisierungstechnik
Universitätsplatz 2, 39106 Magdeburg, Germany
eric.bullinger@ovgu.de

Uwe Greggers & Randolf Menzel*
Freie Universität Berlin
Neurobiologie
Königin Luisestr. 1 -3, 14195 Berlin, Germany
menzel@neurobiologie.fu-berlin.de

14 February 2023

- Bee A01: p. 2
- Bee A02: p. 3
- Bee A03: p. 4
- Bee A04: p. 5
- Bee A05: p. 6
- Bee A06: p. 7
- Bee A07: p. 8
- Bee A08: p. 9
- Bee A09: p. 10
- Bee A10: p. 11
- Bee A11: p. 12
- Bee A12: p. 13
- Bee A13: p. 14
- Bee A14: p. 15
- Bee B01: p. 16
- Bee B02: p. 17
- Bee B03: p. 18
- Bee B04: p. 19
- Bee B05: p. 20
- Bee B06: p. 21
- Bee B07: p. 22
- Bee B08: p. 23
- Bee B09: p. 24
- Bee B10: p. 25
- Bee B11: p. 26
- Bee C01: p. 27
- Bee C02: p. 28
- Bee C03: p. 29
- Bee C04: p. 30
- Bee C05: p. 31
- Bee D01: p. 32
- Bee D02: p. 33
- Bee D03: p. 34
- Bee D04: p. 35
- Bee D05: p. 36
- Bee D06: p. 37
- Bee D07: p. 38
- Bee D08: p. 39
- Bee D09: p. 40
- Bee D10: p. 41
- Bee D11: p. 42
- Bee D12: p. 43
- Bee D13: p. 44
- Bee E01: p. 45
- Bee E02: p. 46
- Bee E03: p. 47
- Bee E04: p. 48
- Bee E05: p. 49
- Bee E06: p. 50
- Bee E07: p. 51
- Bee R01: p. 52
- Bee R02: p. 53
- Bee R03: p. 54
- Bee R04: p. 55
- Bee R05: p. 56
- Bee R06: p. 57
- Bee R07: p. 58
- Bee R08: p. 59
- Bee R09: p. 60
- Bee R10: p. 61
- Bee R11: p. 62
- Bee R12: p. 63
- Bee R13: p. 64
- Bee R14: p. 65
- Bee R15: p. 66
- Bee R16: p. 67
- Bee S01: p. 68
- Bee S02: p. 69
- Bee S03: p. 70
- Bee S04: p. 71
- Bee S05: p. 72
- Bee S06: p. 73
- Bee S07: p. 74
- Bee S08: p. 75
- Bee S09: p. 76
- Bee S10: p. 77
- Bee S11: p. 78
- Bee S12: p. 79
- Bee S13: p. 80
- Bee S14: p. 81
- Bee S15: p. 82
- Bee S16: p. 83

*corresponding author

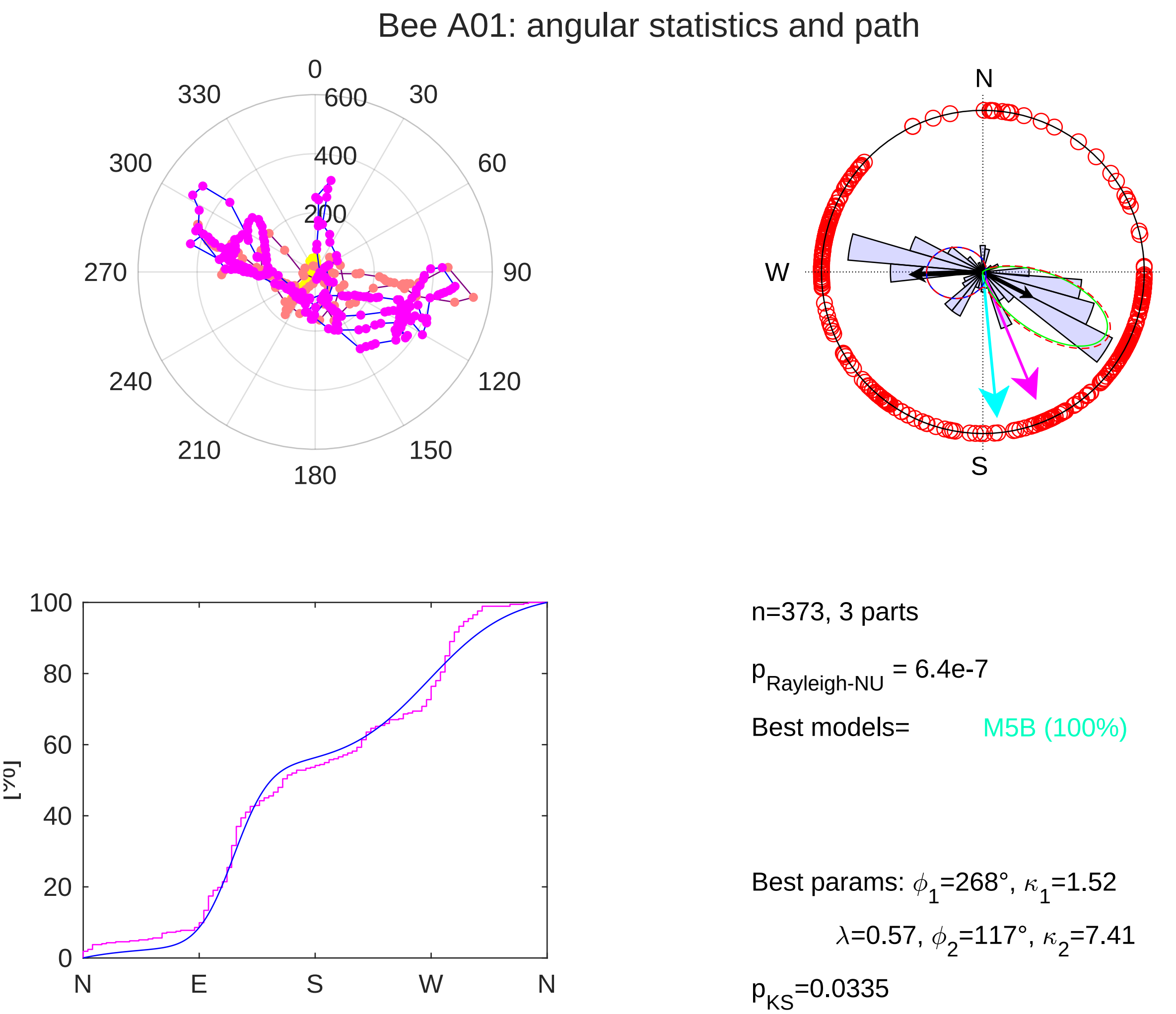
Bee A01: angular statistics and path
0
30
60
90
120
150
180
210
240
270
300
330
200
400
600
N
E
S
W
100
80
60
40
20
0
[%]
N E S W N
n=373, 3 parts
p_Rayleigh-NU = 6.4e-7
Best models= M5B (100%)
Best params: φ1=268°, κ1=1.52
λ=0.57, φ2=117°, κ2=7.41
p_KS=0.0335

Bee A02: angular statistics and path

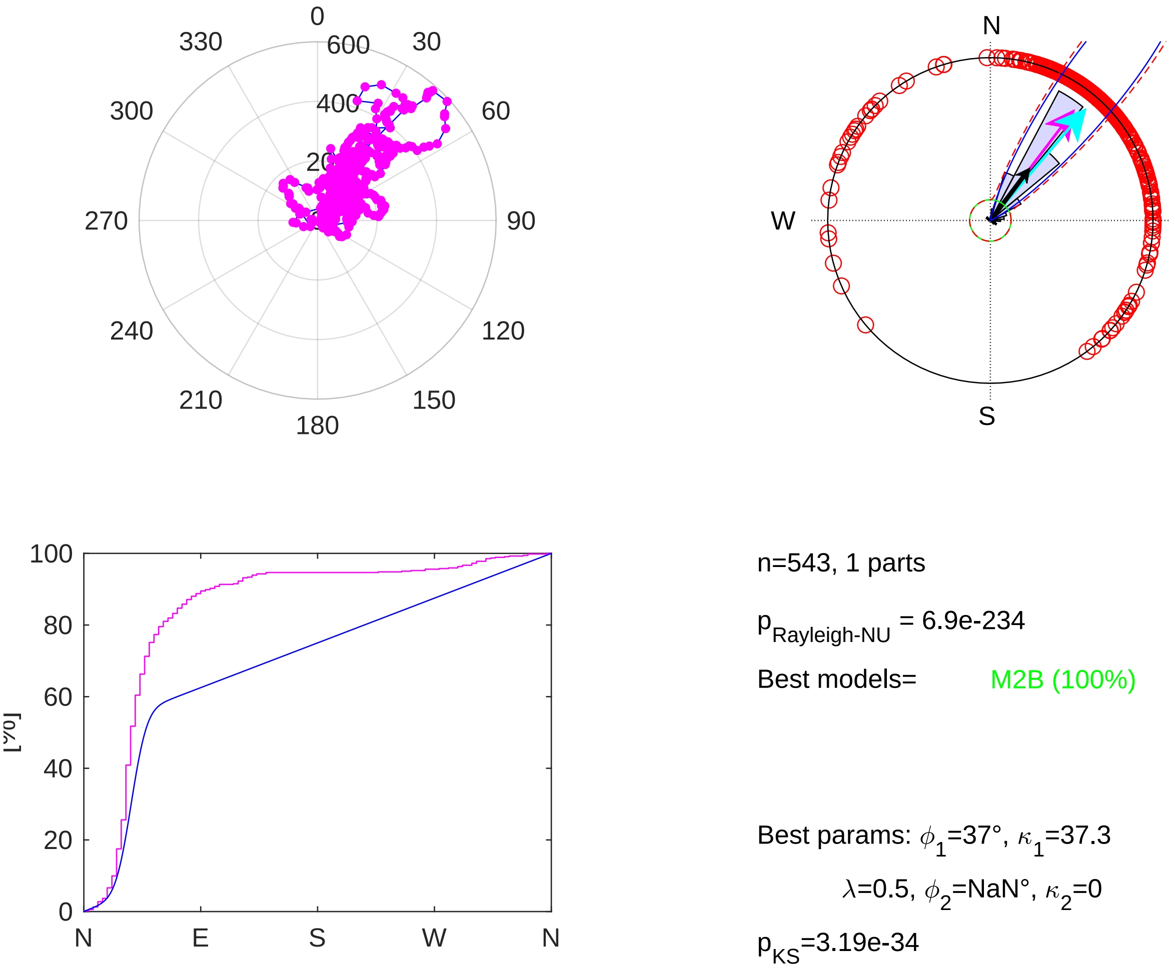

n=543, 1 parts

$p_{Rayleigh\text{-}NU}$ = 6.9e-234

Best models= M2B (100%)

Best params: ϕ_1=37°, κ_1=37.3

λ=0.5, ϕ_2=NaN°, κ_2=0

p_{KS}=3.19e-34

Bee A03: angular statistics and path

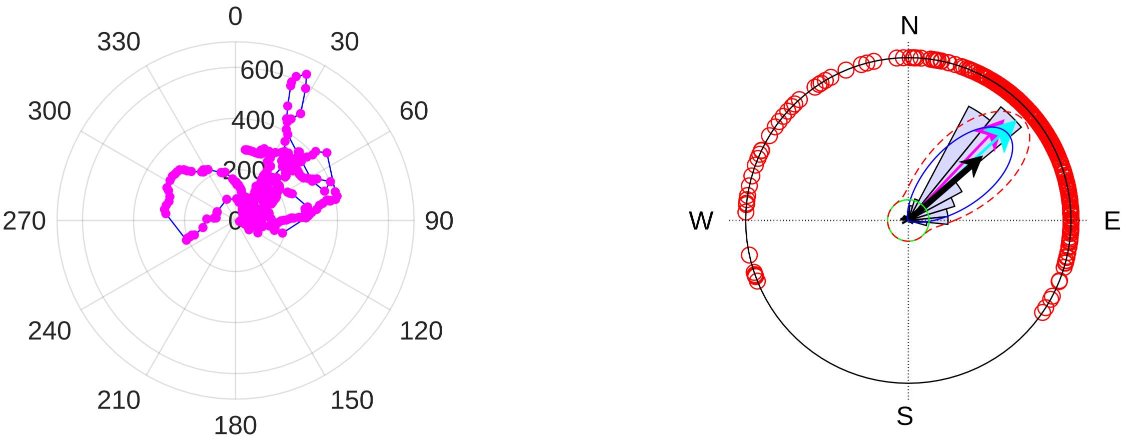

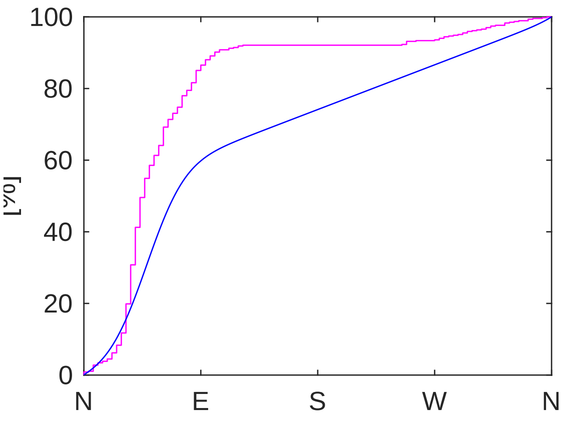

n=468, 1 parts

$p_{Rayleigh\text{-}NU}$ = 3e-179

Best models= M2C (100%)

Best params: ϕ_1=49°, κ_1=6.74

λ=0.75, ϕ_2=NaN°, κ_2=0

p_{KS}=6.64e-31

Bee A04: angular statistics and path

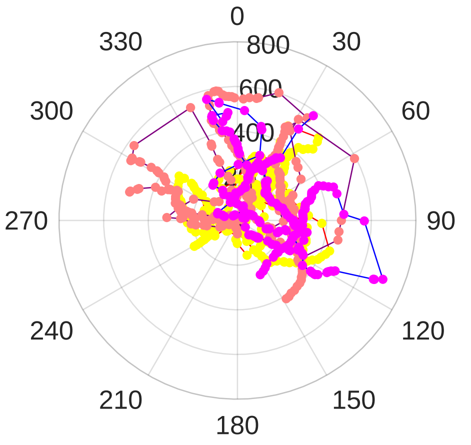

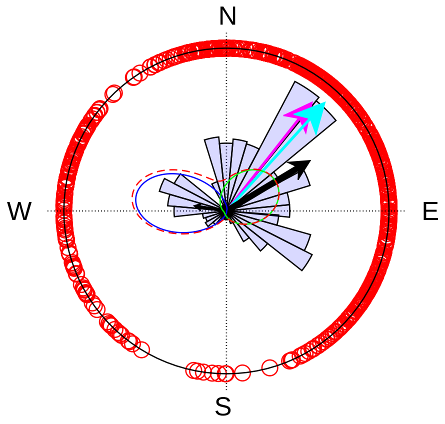

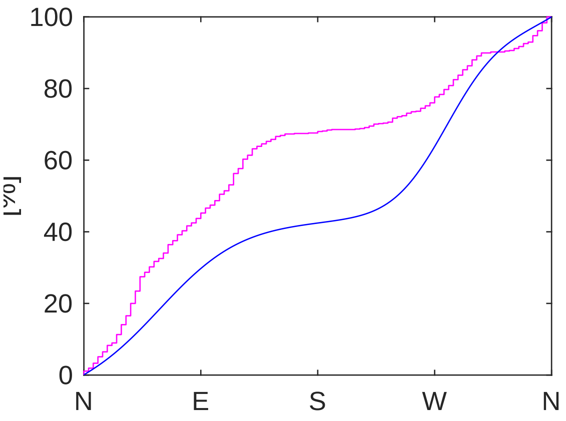

n=725, 3 parts

$p_{Rayleigh\text{-}NU}$ = 7.1e-50

Best models= M5B (100%)

Best params: ϕ_1=279°, κ_1=3.41

λ=0.25, ϕ_2=59°, κ_2=1.54

p_{KS}=8.47e-44

Bee A05: angular statistics and path

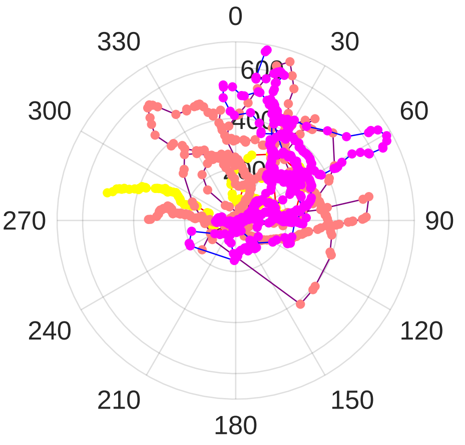

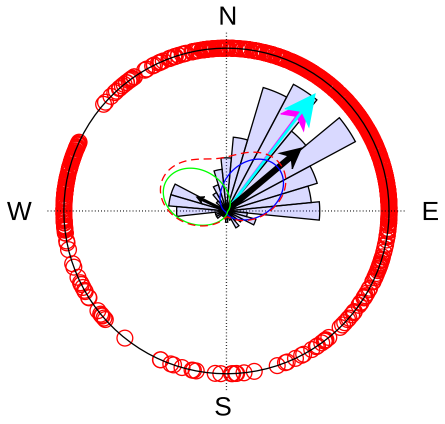

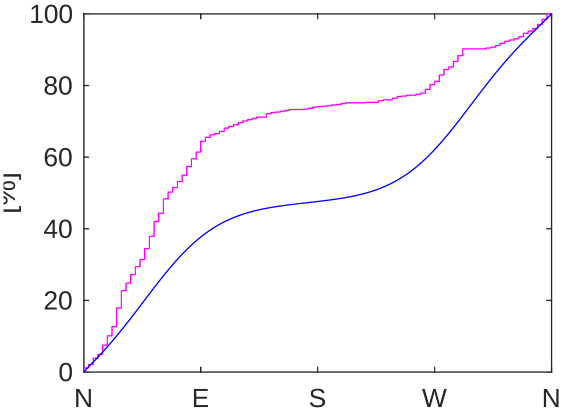

n=850, 3 parts

$p_{Rayleigh\text{-}NU}$ = 5.7e-121

Best models= M5A (100%)

Best params: ϕ_1=50°, κ_1=2.01

λ=0.75, ϕ_2=295°, κ_2=2.01

p_{KS}=2.01e-52

Bee A06: angular statistics and path

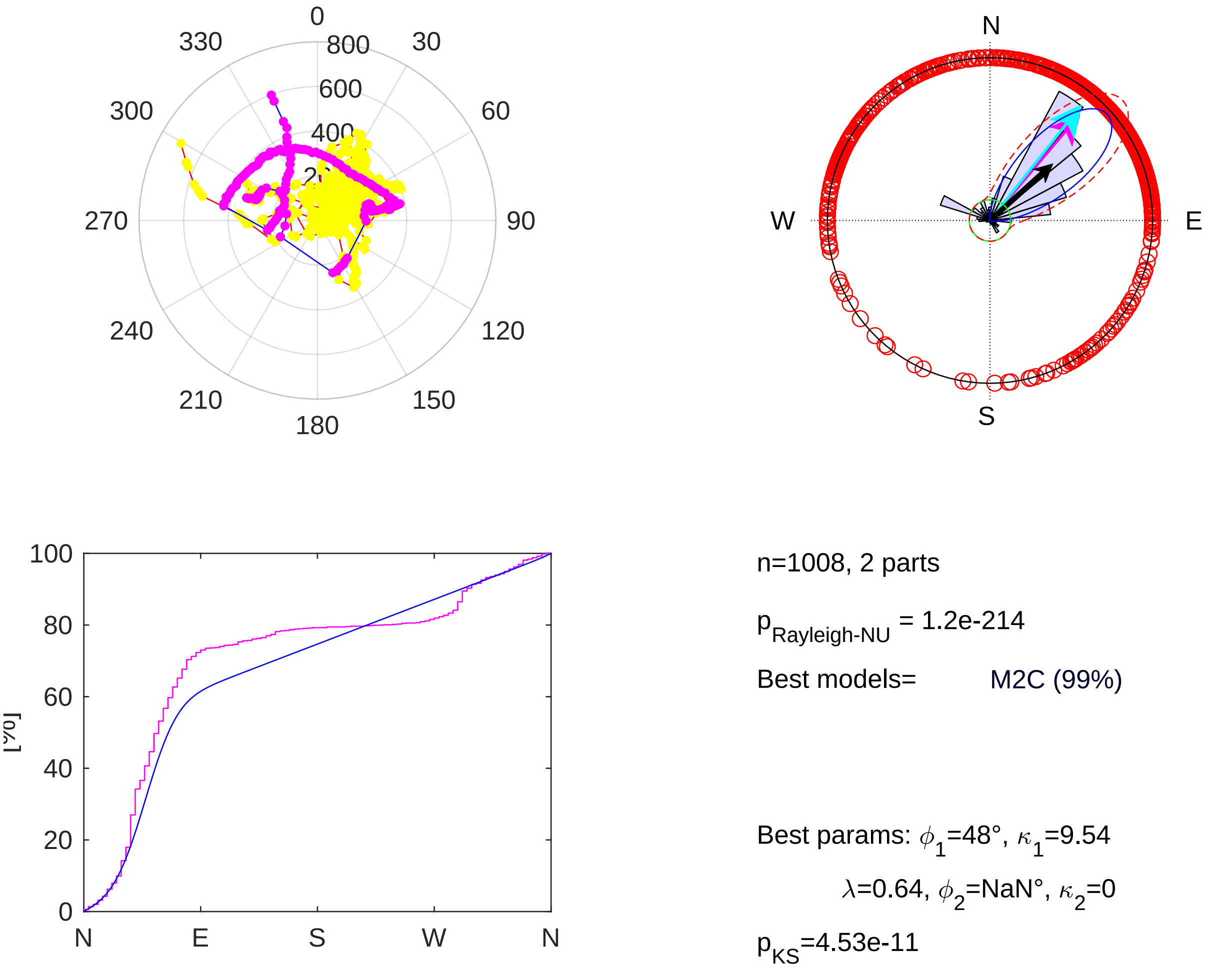

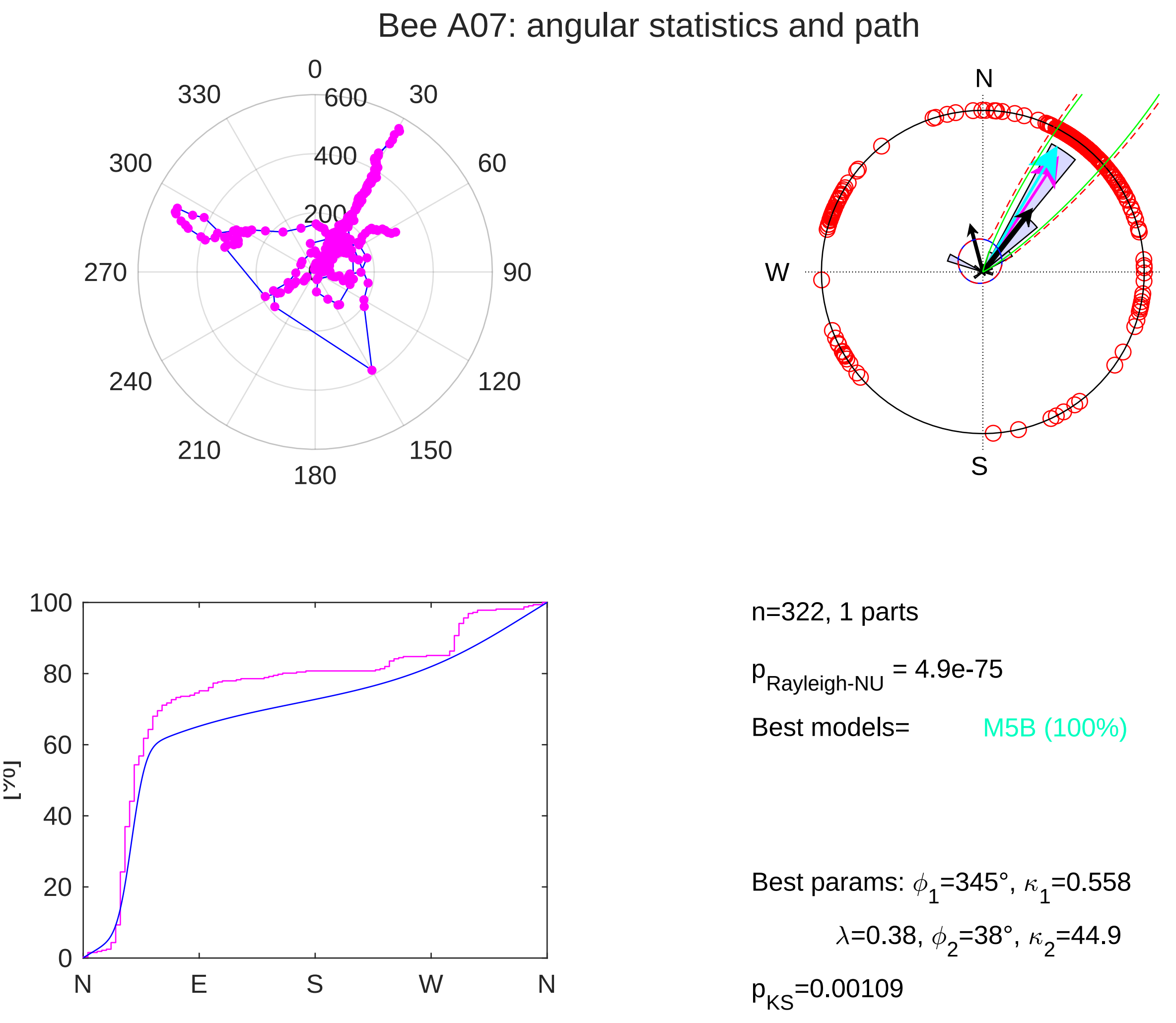
Bee A07: angular statistics and path
0
30
60
90
120
150
180
210
240
270
300
330
200
400
600
N
E
S
W
100
80
60
40
20
0
[%]
N
E
S
W
N
n=322, 1 parts
$p_{Rayleigh\text{-}NU}$ = 4.9e-75
Best models= M5B (100%)
Best params: ϕ_1=345°, κ_1=0.558
λ=0.38, ϕ_2=38°, κ_2=44.9
p_{KS}=0.00109

Bee A08: angular statistics and path

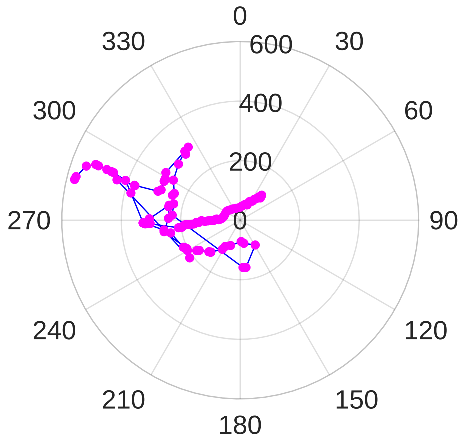

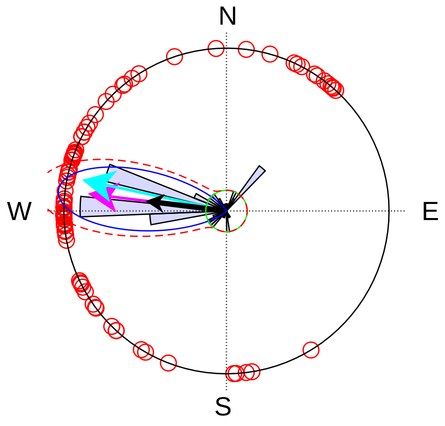

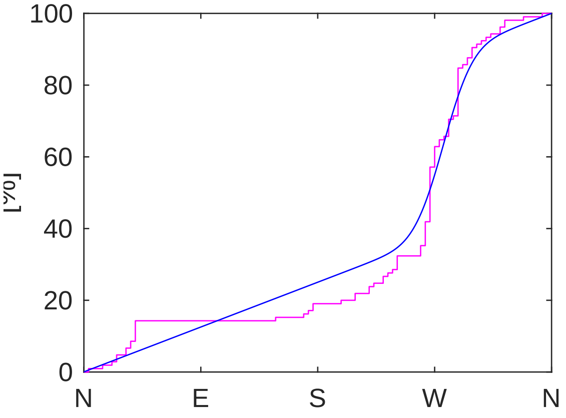

n=105, 1 parts

$p_{Rayleigh\text{-}NU}$ = 2.5e-22

Best models= M2C (53%)
M2B (42%)

Best params: ϕ_1=277°, κ_1=10.9

λ=0.62, ϕ_2=NaN°, κ_2=0

p_{KS}=0.0574

Bee A09: angular statistics and path

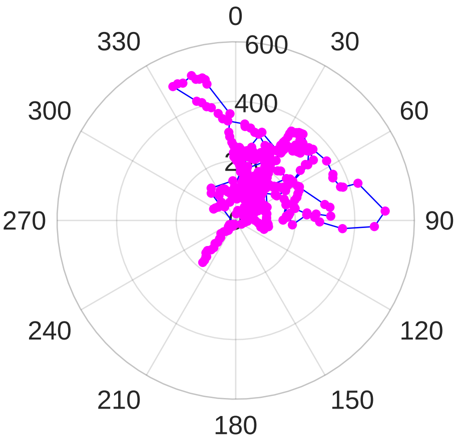

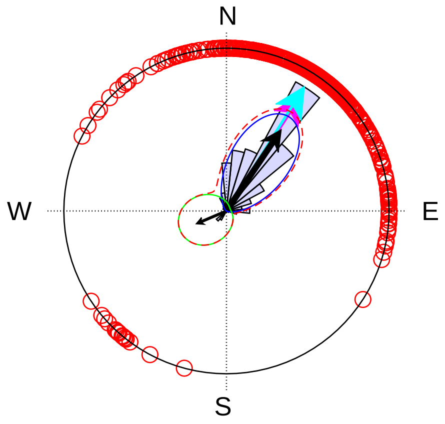

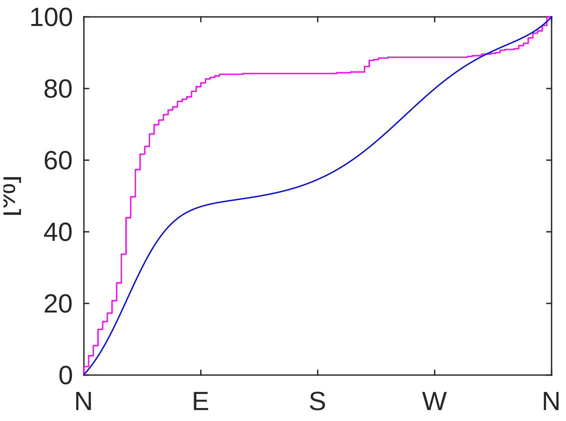

n=462, 1 parts

$p_{Rayleigh\text{-}NU}$ = 9e-160

Best models= M5B (100%)

Best params: ϕ_1=34°, κ_1=5.01

λ=0.75, ϕ_2=247°, κ_2=1.24

p_{KS}=1.19e-51

Bee A10: angular statistics and path

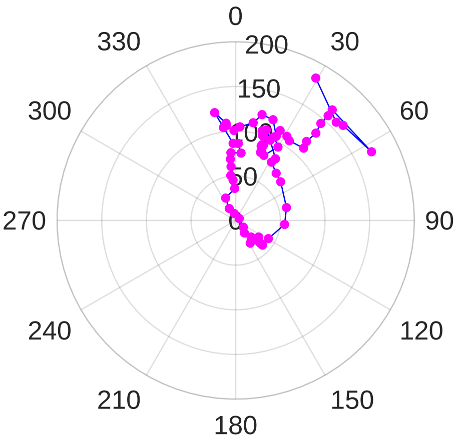

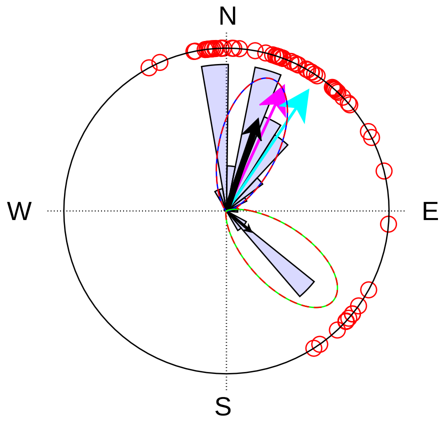

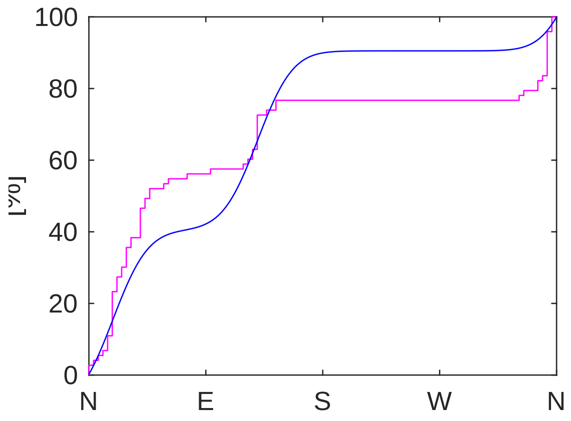

n=73, 1 parts

$p_{Rayleigh-NU}$ = 6.3e-18

Best models= M5A (100%)

Best params: ϕ_1=19°, κ_1=7.48

λ=0.75, ϕ_2=130°, κ_2=7.48

p_{KS}=0.0524

Bee A11: angular statistics and path

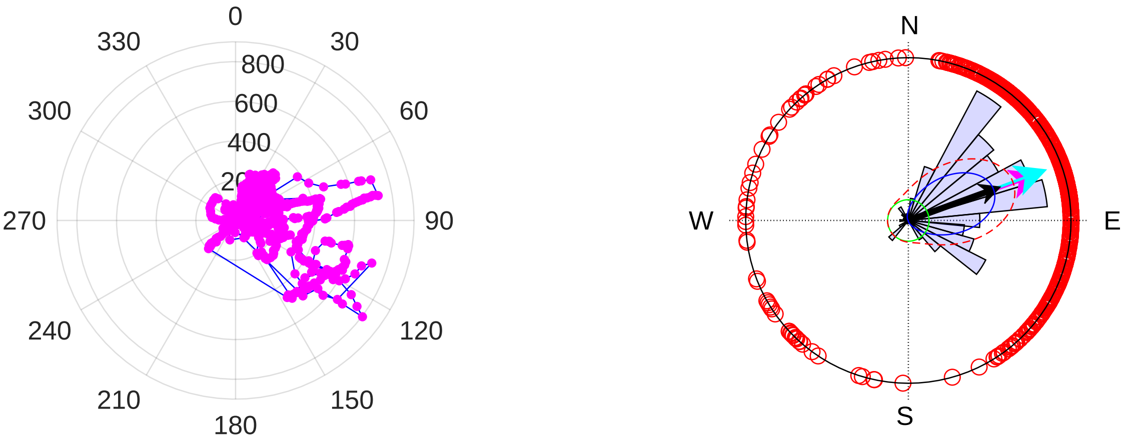

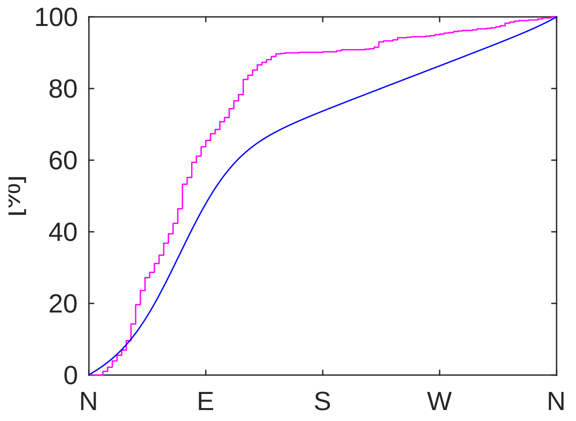

n=687, 1 parts

$p_{Rayleigh\text{-}NU}$ = 1.1e-165

Best models= M2C (100%)

Best params: ϕ_1=70°, κ_1=3.35

λ=0.75, ϕ_2=NaN°, κ_2=0

p_{KS}=6.48e-28

Bee A12: angular statistics and path

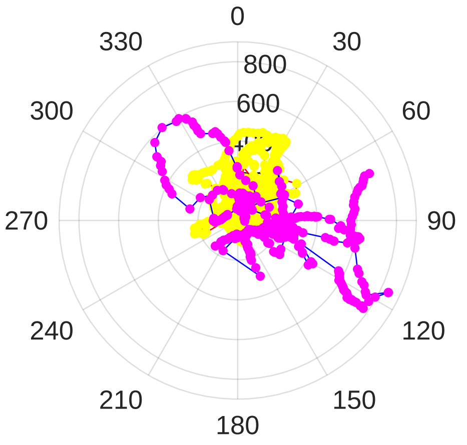

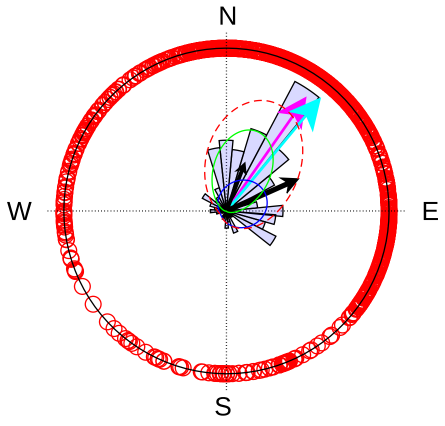

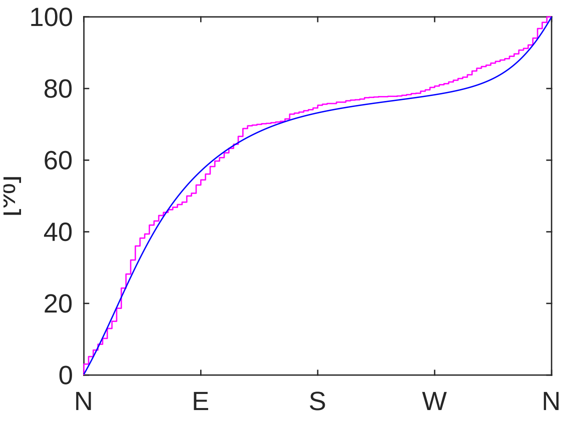

n=1046, 2 parts

$p_{Rayleigh\text{-}NU}$ = 1.7e-138

Best models= M5B (100%)

Best params: ϕ_1=66°, κ_1=0.919

λ=0.6, ϕ_2=21°, κ_2=2.99

p_{KS}=0.0069

Bee A13: angular statistics and path

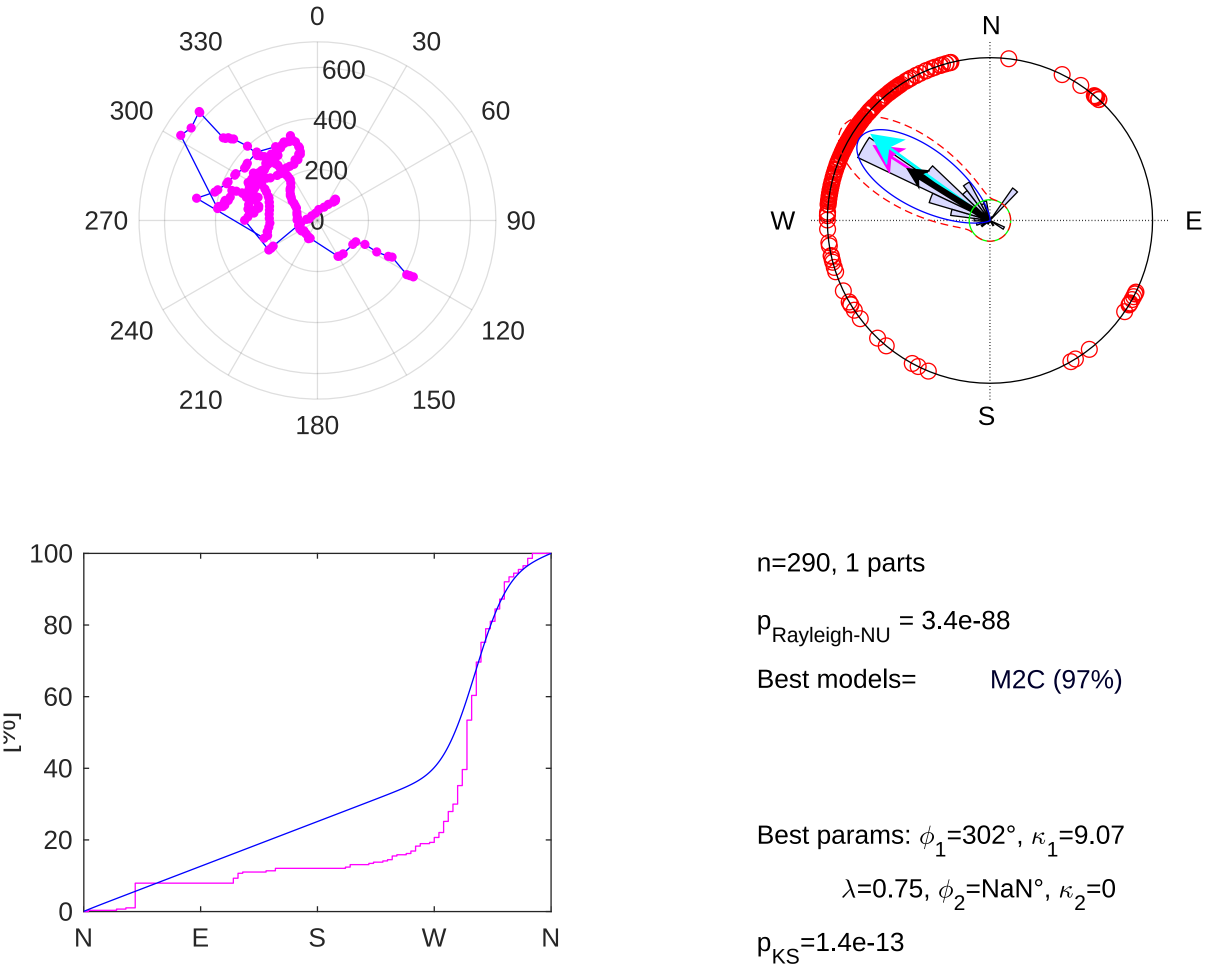

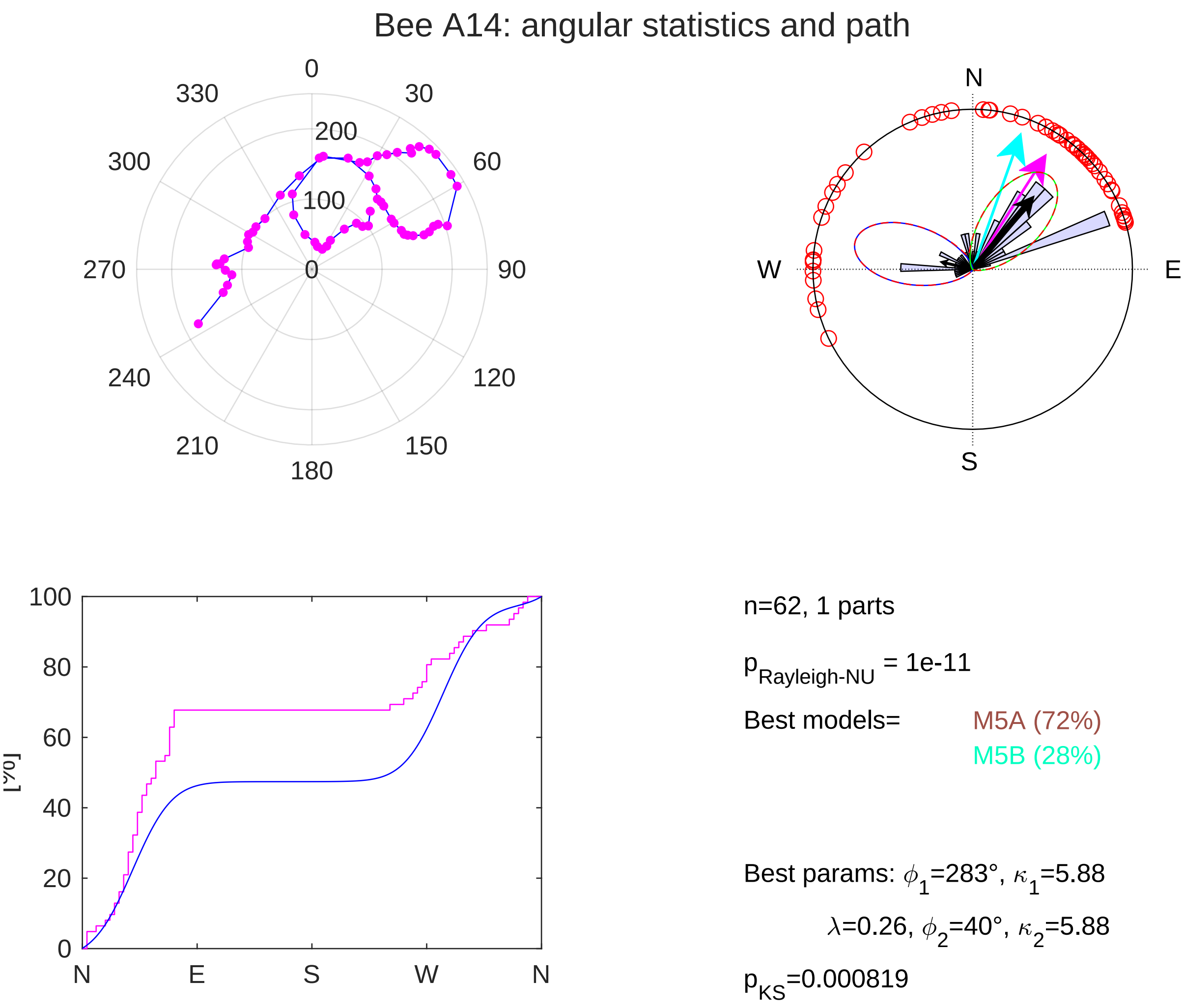
Bee A14: angular statistics and path
0
30
60
90
120
150
180
210
240
270
300
330
200
100
0
N
E
S
W
100
80
60
40
20
0
[%]
N
E
S
W
N
n=62, 1 parts
p_Rayleigh-NU = 1e-11
Best models=
M5A (72%)
M5B (28%)
Best params: φ_1=283°, κ_1=5.88
λ=0.26, φ_2=40°, κ_2=5.88
p_KS=0.000819

Bee B01: angular statistics and path

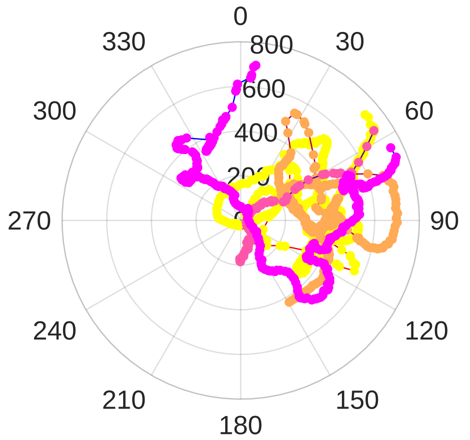

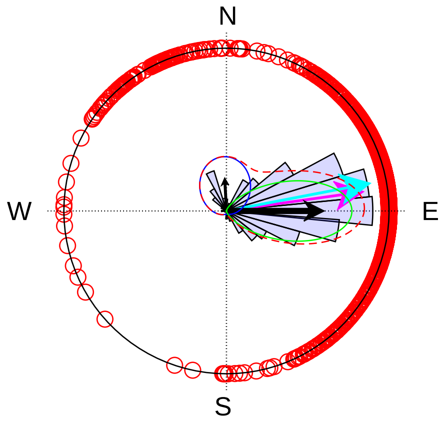

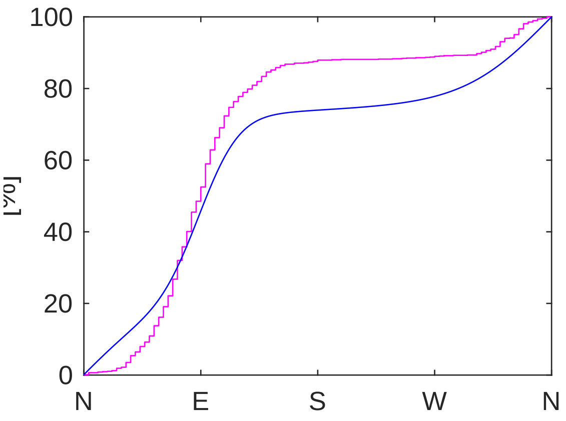

n=1053, 4 parts

$p_{Rayleigh\text{-}NU}$ = 1.9e-276

Best models= M5B (100%)

Best params: ϕ_1=357°, κ_1=1.41

λ=0.25, ϕ_2=90°, κ_2=6.11

p_{KS}=2.59e-18

Bee B02: angular statistics and path

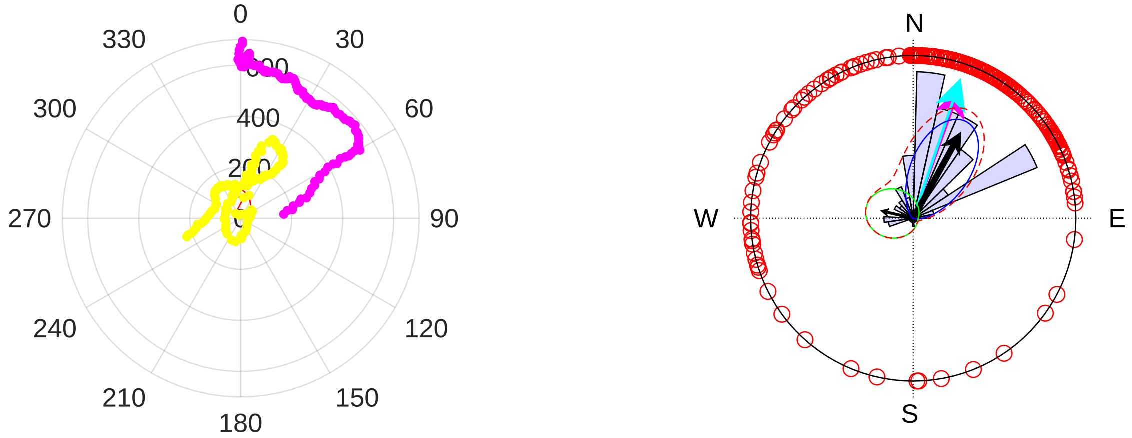

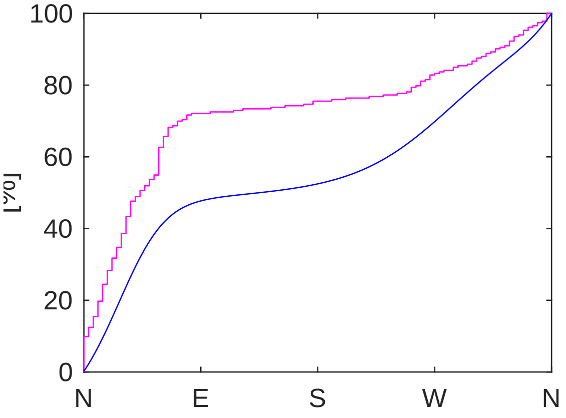

n=233, 2 parts

$p_{\text{Rayleigh-NU}}$ = 1.7e-53

Best models= M5B (100%)

Best params: ϕ_1=29°, κ_1=4.78

λ=0.75, ϕ_2=283°, κ_2=1.15

p_{KS}=3.19e-13

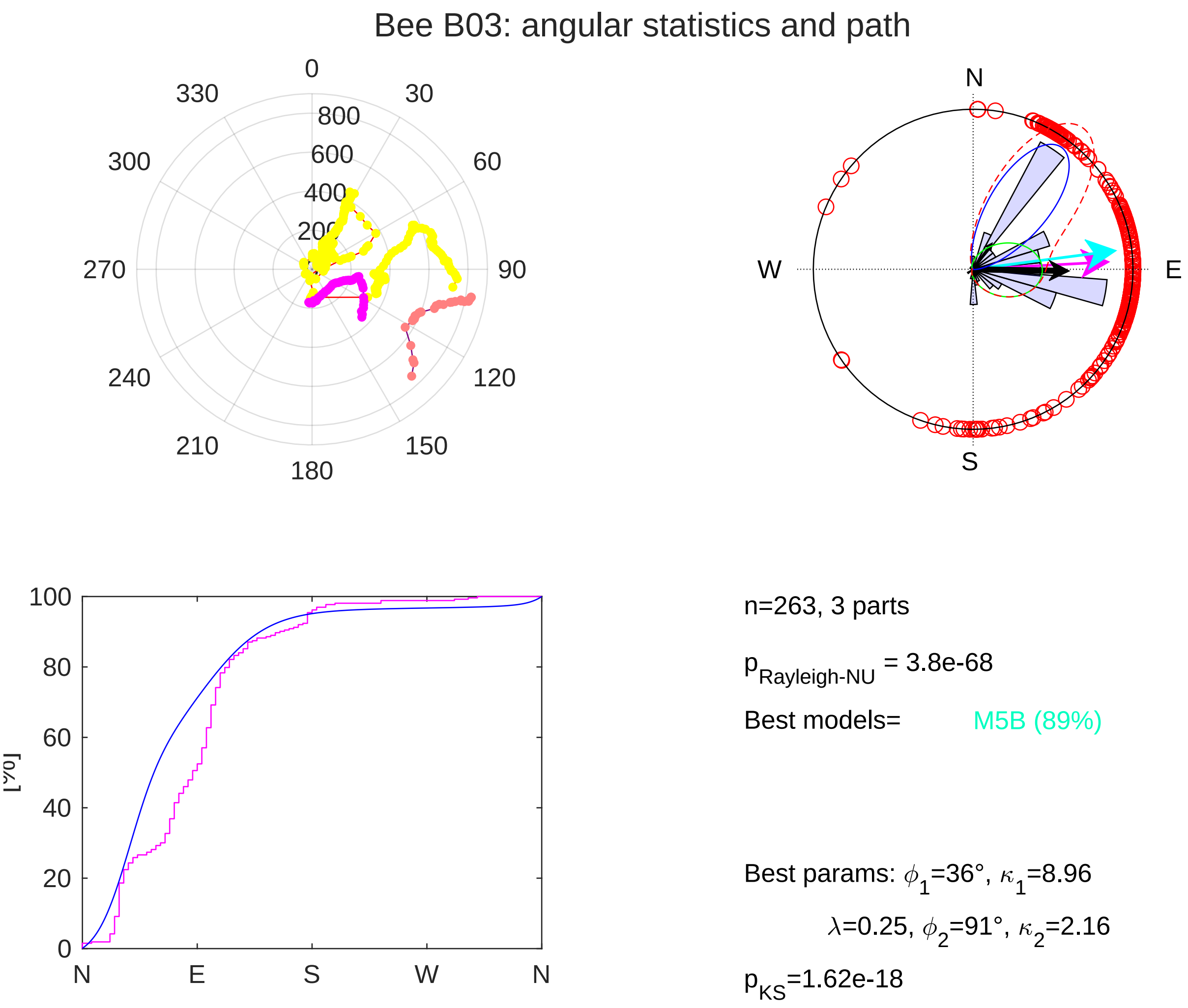
Bee B03: angular statistics and path
0
30
60
90
120
150
180
210
240
270
300
330
800
600
400
200
N
E
S
W
100
80
60
40
20
0
[%]
N
E
S
W
N
n=263, 3 parts
p Rayleigh-NU = 3.8e-68
Best models= M5B (89%)
Best params: ϕ1=36°, κ1=8.96
λ=0.25, ϕ2=91°, κ2=2.16
pKS=1.62e-18

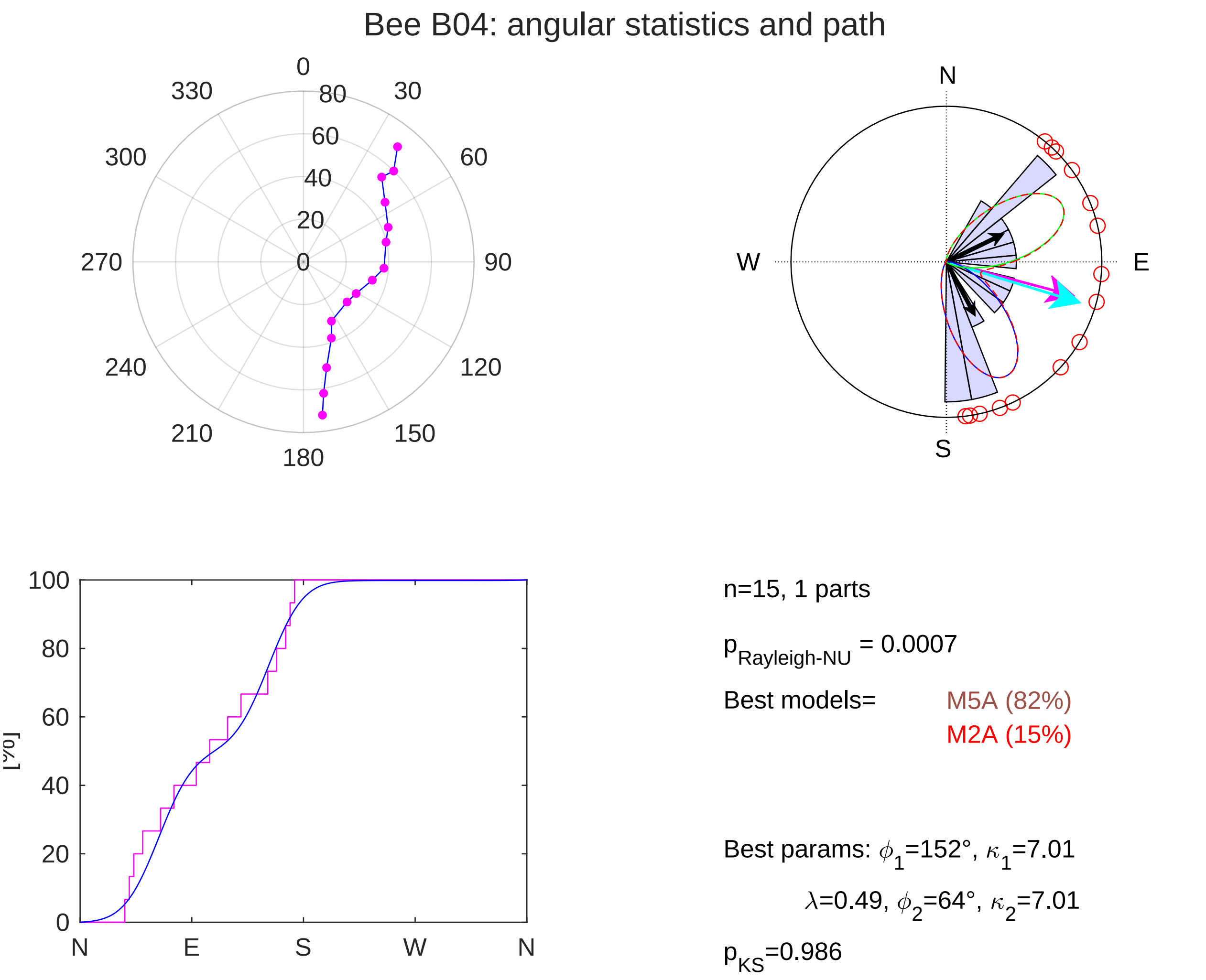
Bee B04: angular statistics and path
0
30
60
90
120
150
180
210
240
270
300
330
80
60
40
20
0
N
E
S
W
100
80
60
40
20
0
[%]
N
E
S
W
N
n=15, 1 parts
p$_{Rayleigh-NU}$ = 0.0007
Best models=
M5A (82%)
M2A (15%)
Best params: ϕ_1=152°, κ_1=7.01
λ=0.49, ϕ_2=64°, κ_2=7.01
p$_{KS}$=0.986

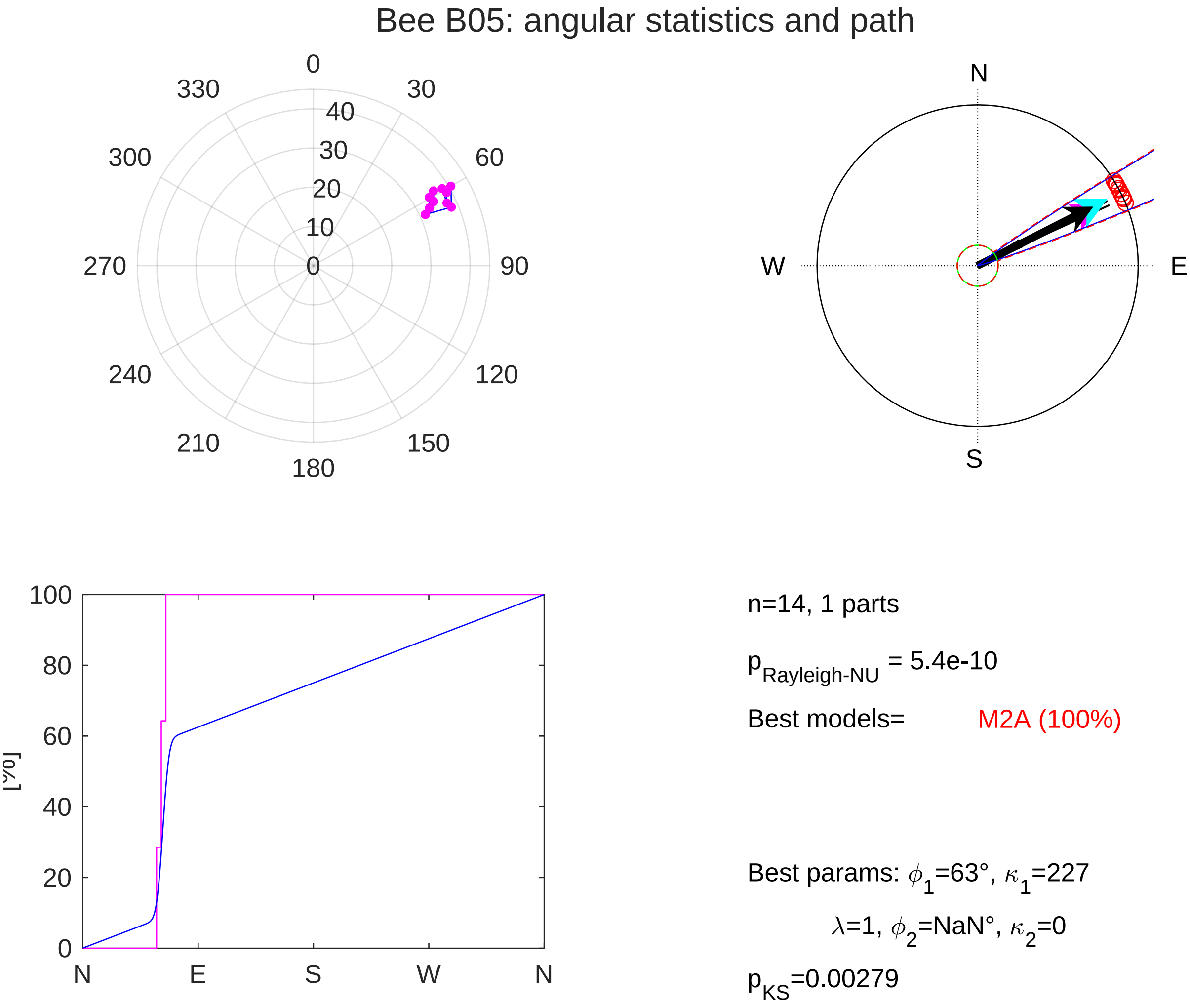
Bee B05: angular statistics and path
0
30
60
90
120
150
180
210
240
270
300
330
10
20
30
40
N
E
S
W
100
80
60
40
20
0
[%]
N
E
S
W
N
n=14, 1 parts
$p_{Rayleigh-NU}$ = 5.4e-10
Best models= M2A (100%)
Best params: ϕ_1=63°, κ_1=227
λ=1, ϕ_2=NaN°, κ_2=0
p_{KS}=0.00279

Bee B06: angular statistics and path

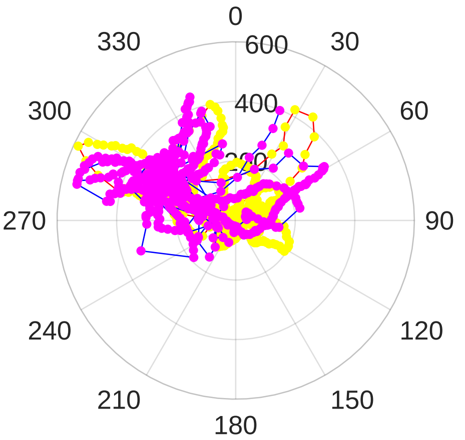

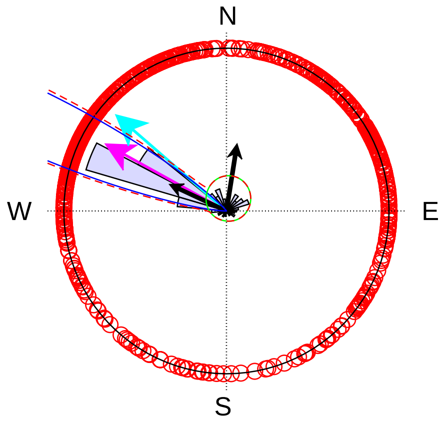

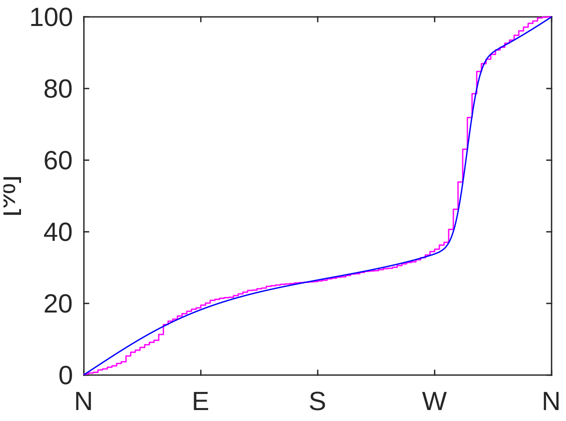

n=1050, 2 parts

$p_{Rayleigh\text{-}NU}$ = 1e-144

Best models= M5B (100%)

Best params: ϕ_1=295°, κ_1=54.2

λ=0.48, ϕ_2=9°, κ_2=0.634

p_{KS}=0.0713

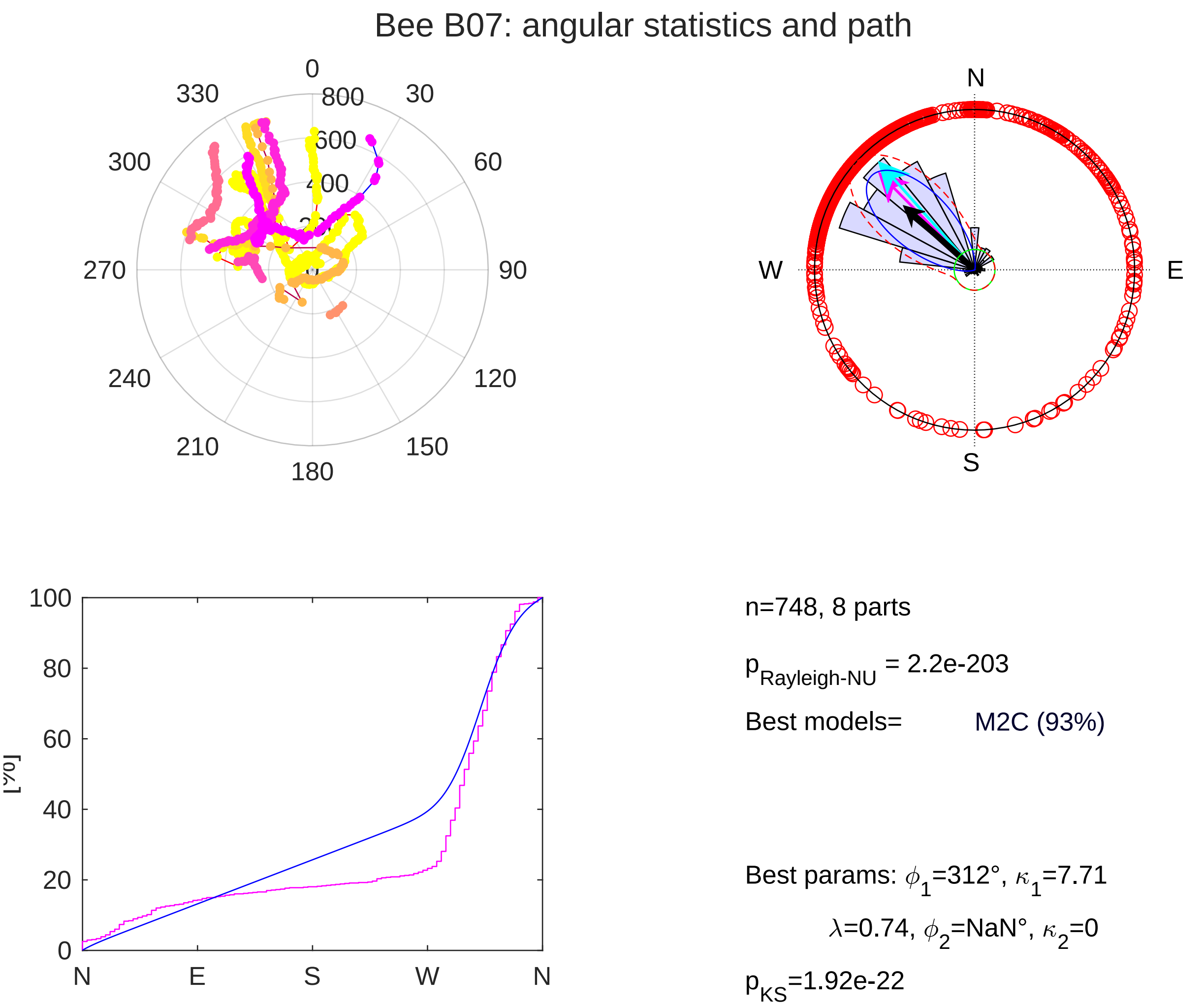
Bee B07: angular statistics and path
0
30
60
90
120
150
180
210
240
270
300
330
800
600
400
N
E
S
W
100
80
60
40
20
0
[%]
N
E
S
W
N
n=748, 8 parts
$p_{Rayleigh\text{-}NU}$ = 2.2e-203
Best models= M2C (93%)
Best params: ϕ_1=312°, κ_1=7.71
λ=0.74, ϕ_2=NaN°, κ_2=0
p_{KS}=1.92e-22

Bee B08: angular statistics and path

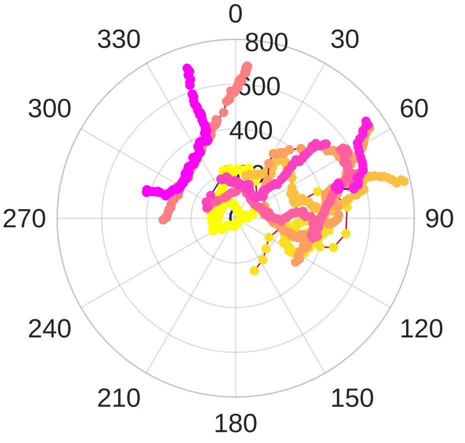

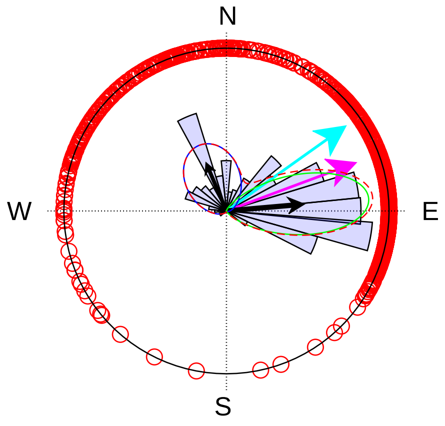

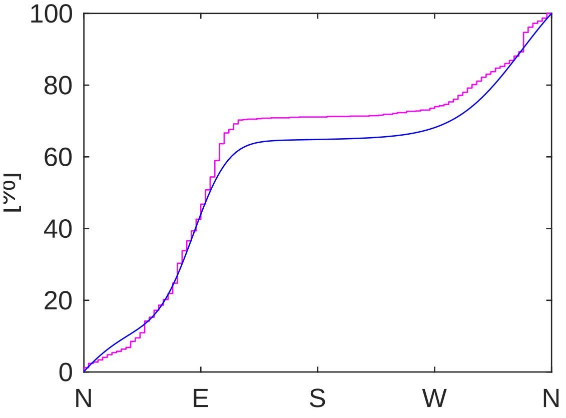

n=831, 9 parts

$p_{Rayleigh\text{-}NU}$ = 4.9e-117

Best models= M5B (100%)

Best params: ϕ_1=337°, κ_1=2.19

λ=0.4, ϕ_2=85°, κ_2=7.83

p_{KS}=5.36e-05

Bee B09: angular statistics and path

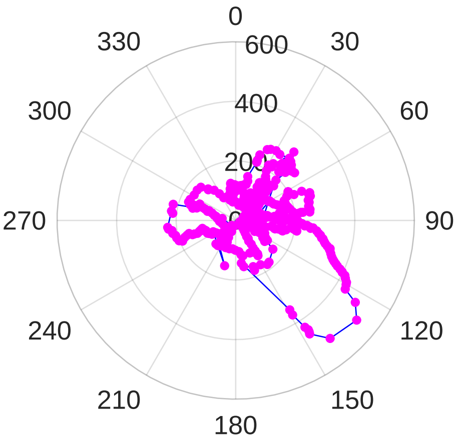

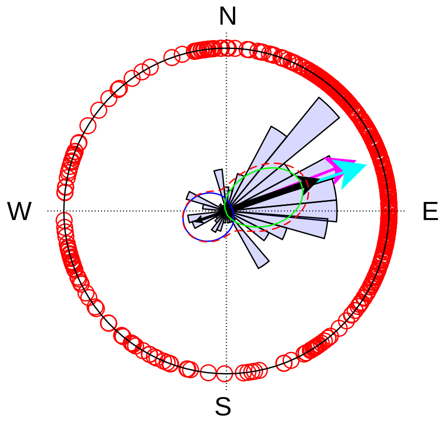

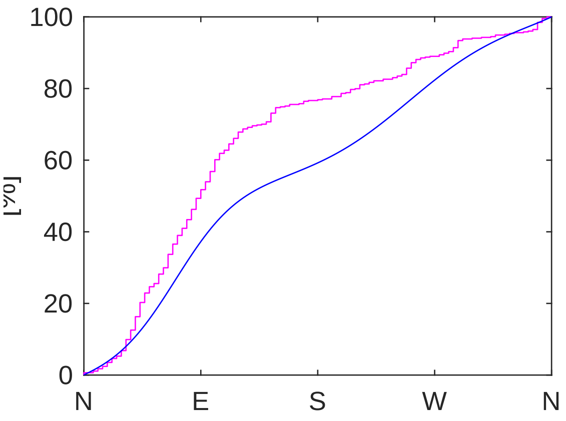

n=454, 1 parts

$p_{Rayleigh\text{-}NU}$ = 3.5e-47

Best models= M4B (91%)

Best params: ϕ_1=251°, κ_1=1.01

λ=0.25, ϕ_2=71°, κ_2=2.65

p_{KS}=2.46e-16

Bee B10: angular statistics and path

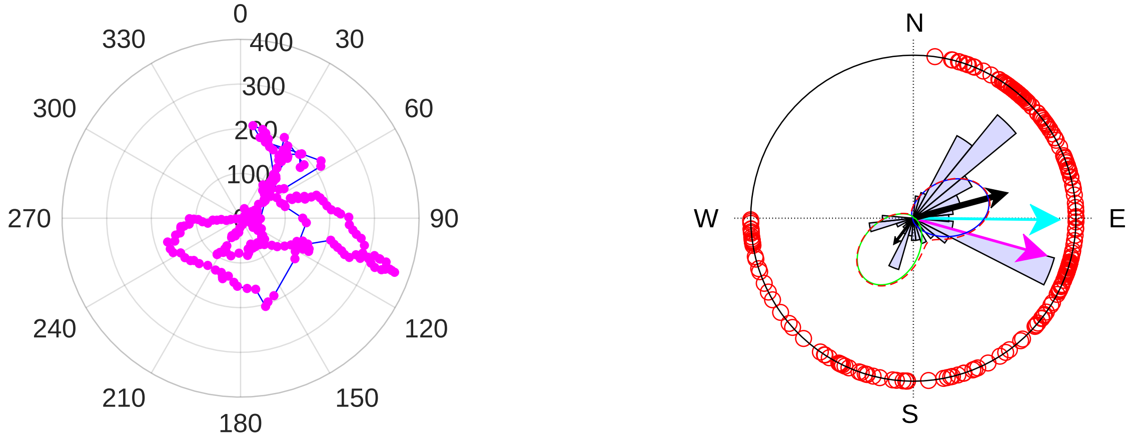

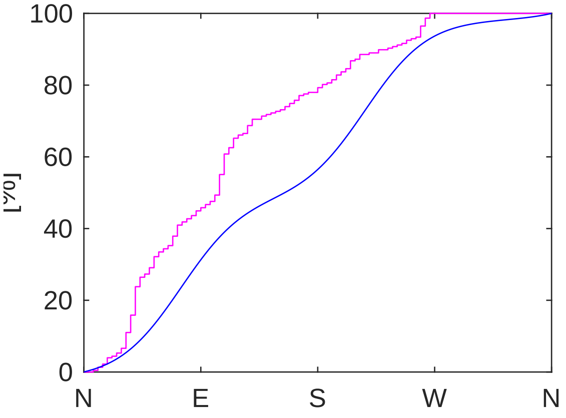

n=227, 1 parts

$p_{Rayleigh-NU}$ = 1.2e-20

Best models= M5A (100%)

Best params: ϕ_1=75°, κ_1=2.55

λ=0.75, ϕ_2=217°, κ_2=2.55

p_{KS}=7.63e-13

Bee B11: angular statistics and path

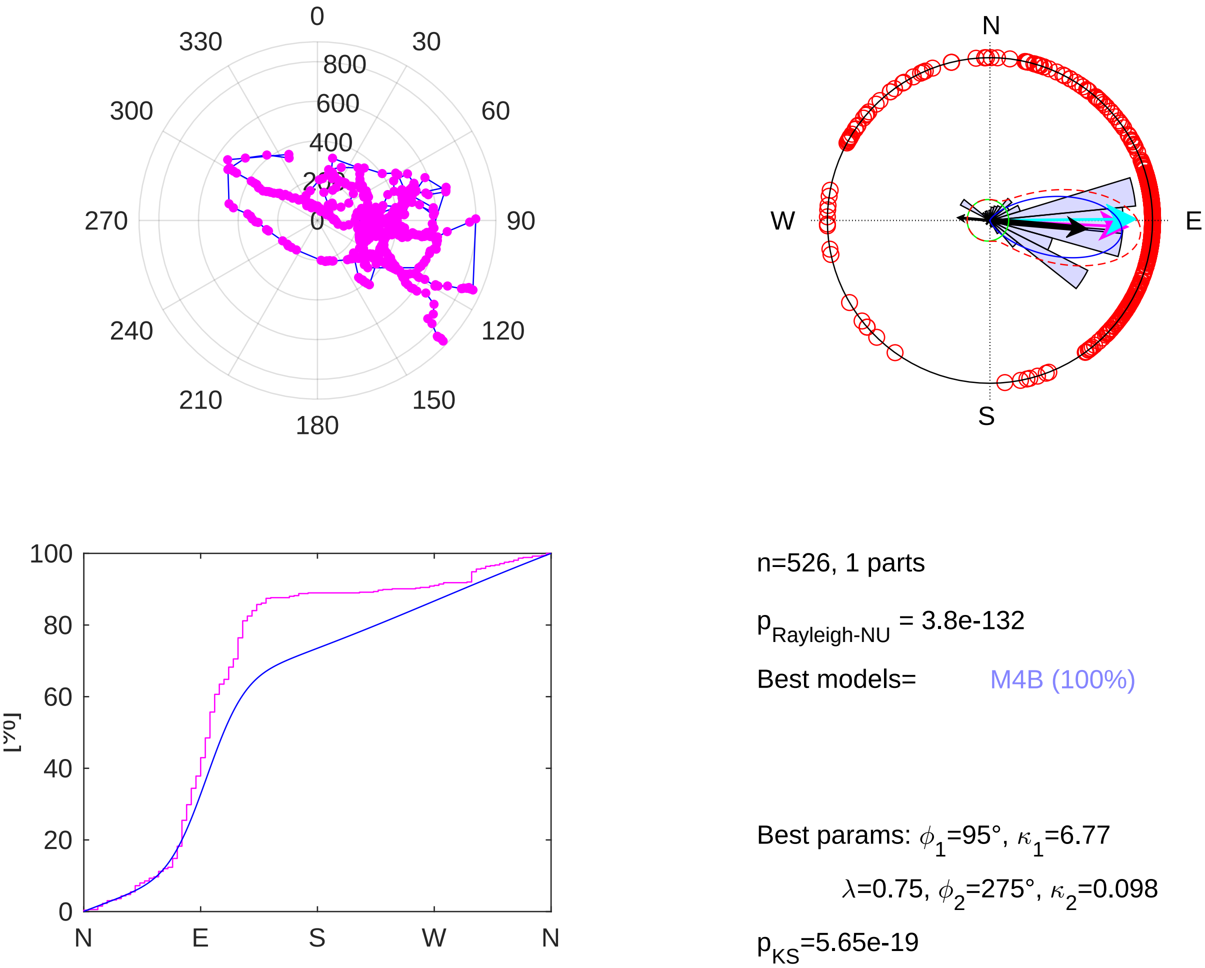

n=526, 1 parts

$p_{Rayleigh\text{-}NU}$ = 3.8e-132

Best models= M4B (100%)

Best params: ϕ_1=95°, κ_1=6.77

λ=0.75, ϕ_2=275°, κ_2=0.098

p_{KS}=5.65e-19

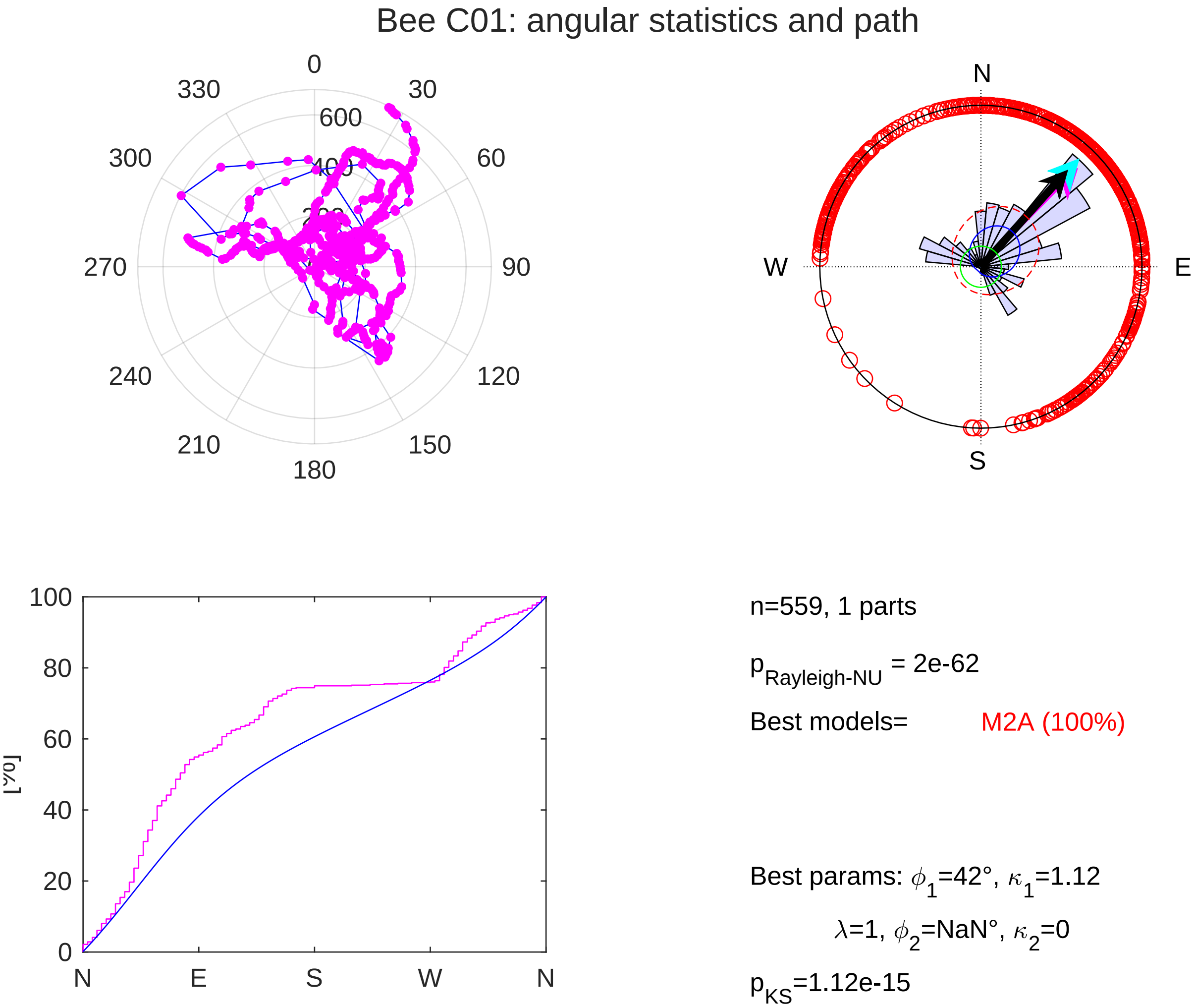
Bee C01: angular statistics and path
0
30
60
90
120
150
180
210
240
270
300
330
600
N
E
S
W
100
80
60
40
20
0
[%]
n=559, 1 parts
p$_{Rayleigh-NU}$ = 2e-62
Best models= M2A (100%)
Best params: ϕ_1=42°, κ_1=1.12
λ=1, ϕ_2=NaN°, κ_2=0
p$_{KS}$=1.12e-15

Bee C02: angular statistics and path

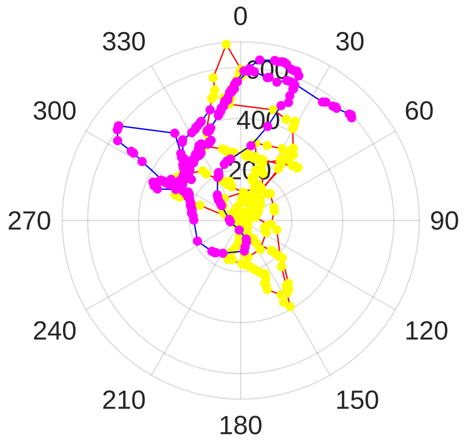

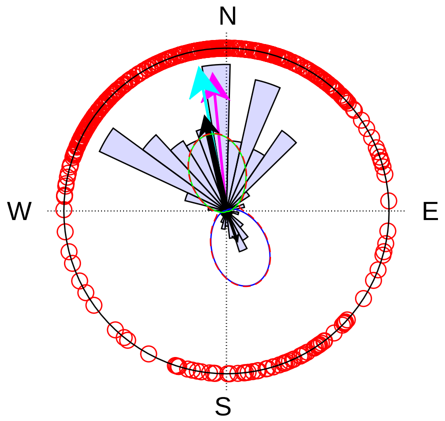

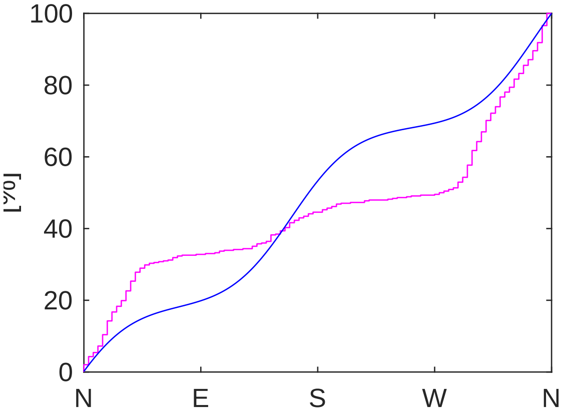

n=442, 2 parts

$p_{Rayleigh\text{-}NU}$ = 7.1e-59

Best models= M5A (53%)
M4A (39%)

Best params: ϕ_1=160°, κ_1=2.6
λ=0.25, ϕ_2=347°, κ_2=2.6

p_{KS}=1.67e-16

Bee C03: angular statistics and path

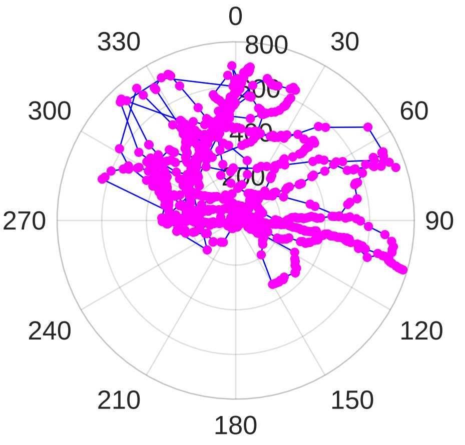

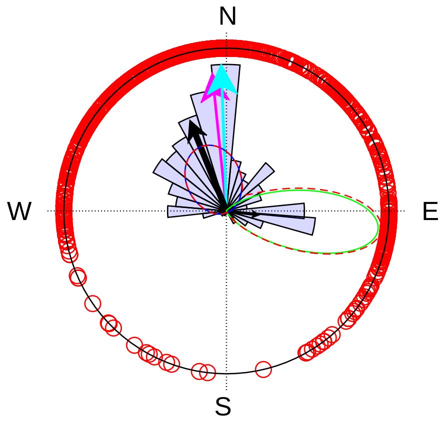

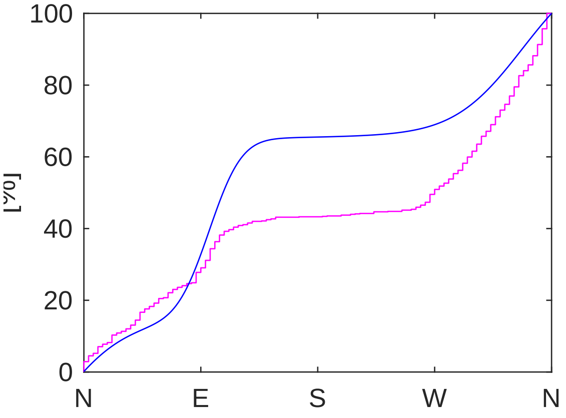

n=864, 1 parts

$p_{Rayleigh\text{-}NU}$ = 6.8e-98

Best models= M5B (100%)

Best params: ϕ_1=338°, κ_1=2.1

λ=0.75, ϕ_2=97°, κ_2=8.92

p_{KS}=1.15e-38

Bee C04: angular statistics and path

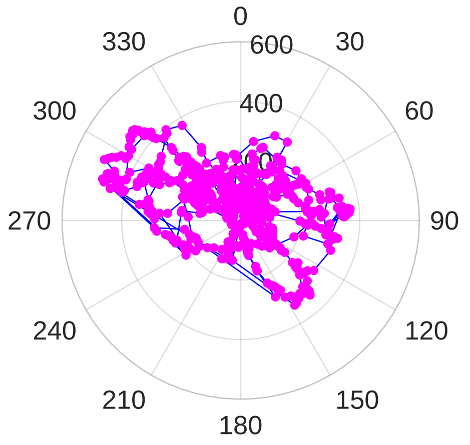

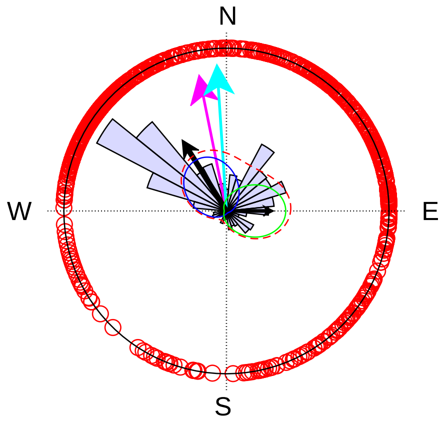

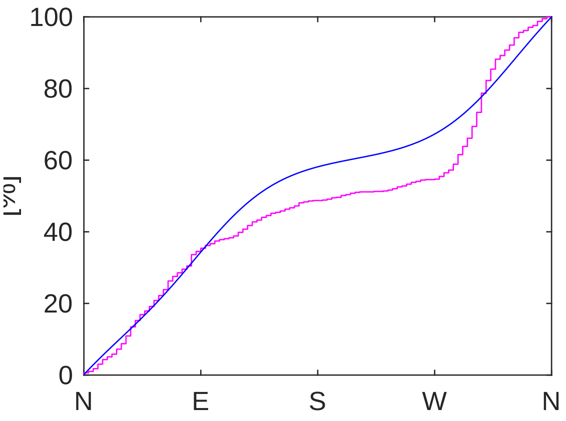

n=788, 1 parts

$p_{Rayleigh-NU}$ = 1.3e-49

Best models= M5A (100%)

Best params: ϕ_1=328°, κ_1=1.63

λ=0.64, ϕ_2=90°, κ_2=1.63

p_{KS}=2.57e-13

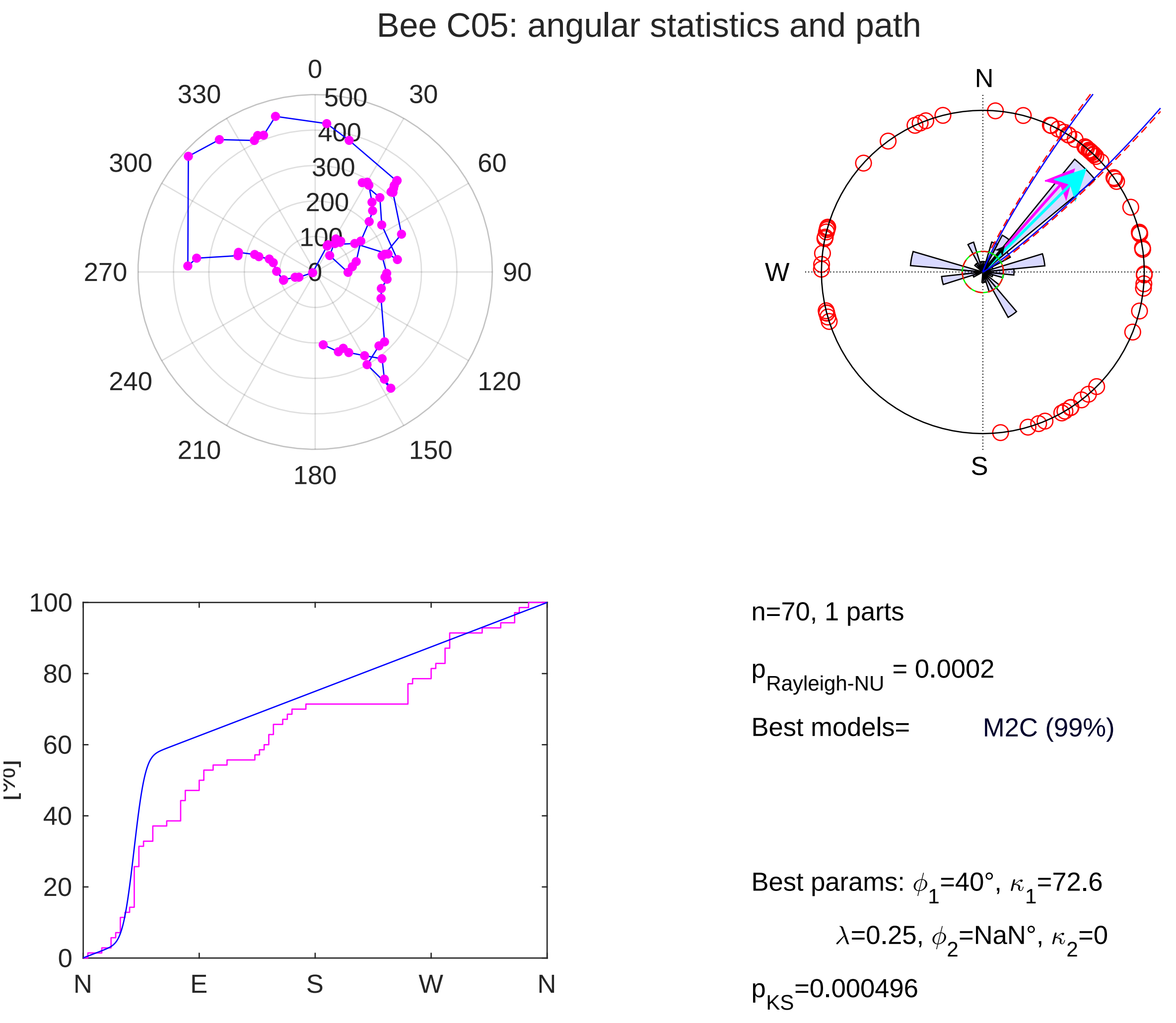
Bee C05: angular statistics and path
0
30
60
90
120
150
180
210
240
270
300
330
500
400
300
200
100
0
N
E
S
W
100
80
60
40
20
0
[%]
N
E
S
W
N
n=70, 1 parts
$p_{Rayleigh\text{-}NU}$ = 0.0002
Best models= M2C (99%)
Best params: ϕ_1=40°, κ_1=72.6
λ=0.25, ϕ_2=NaN°, κ_2=0
p_{KS}=0.000496

Bee D01: angular statistics and path

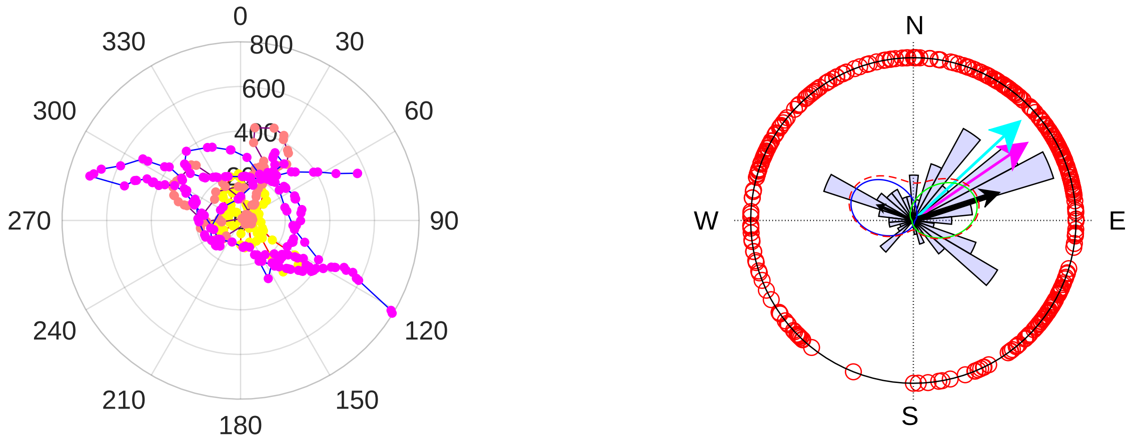

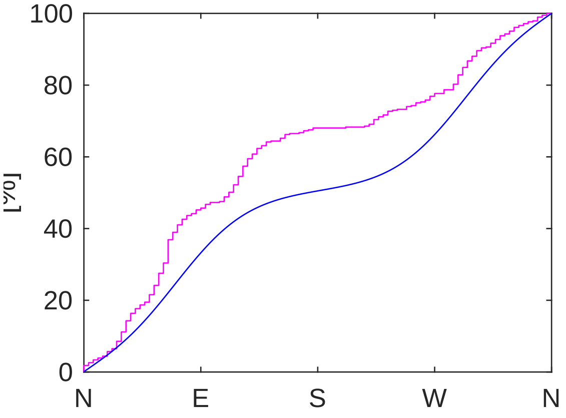

n=385, 3 parts

$p_{Rayleigh\text{-}NU}$ = 7.2e-21

Best models= M5A (99%)

Best params: ϕ_1=293°, κ_1=1.94

λ=0.3, ϕ_2=72°, κ_2=1.94

p_{KS}=7.93e-11

Bee D02: angular statistics and path

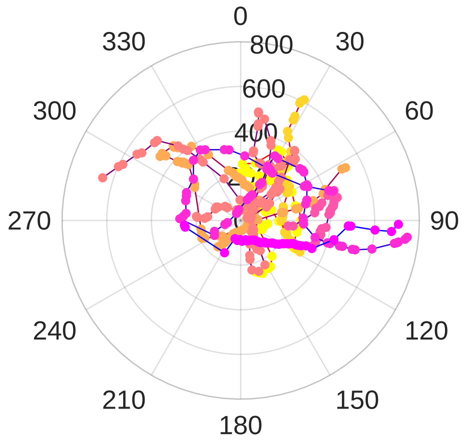

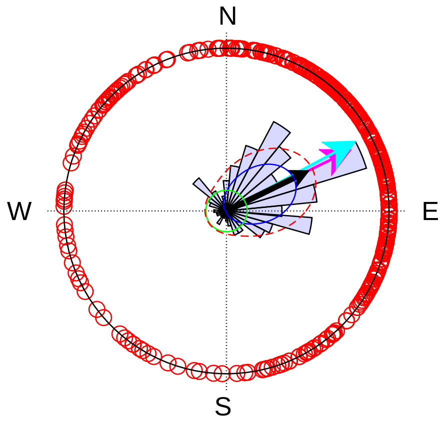

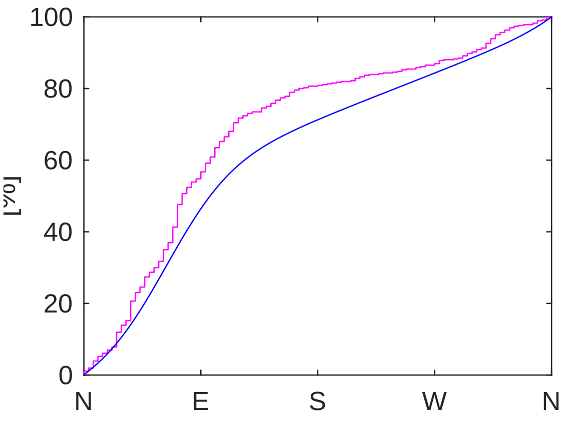

n=460, 7 parts

$p_{Rayleigh\text{-}NU}$ = 1.6e-62

Best models= M2C (98%)

Best params: ϕ_1=64°, κ_1=2.4

λ=0.7, ϕ_2=NaN°, κ_2=0

p_{KS}=2.38e-06

Bee D03: angular statistics and path

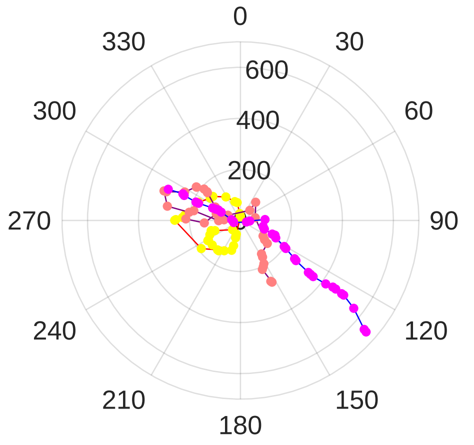

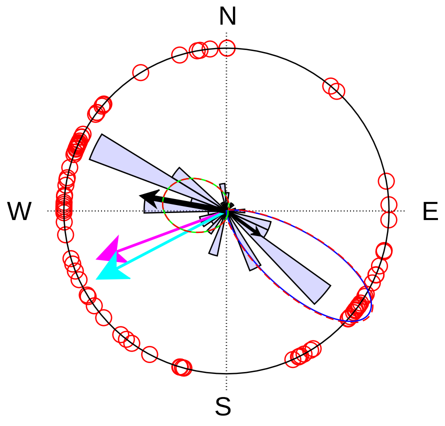

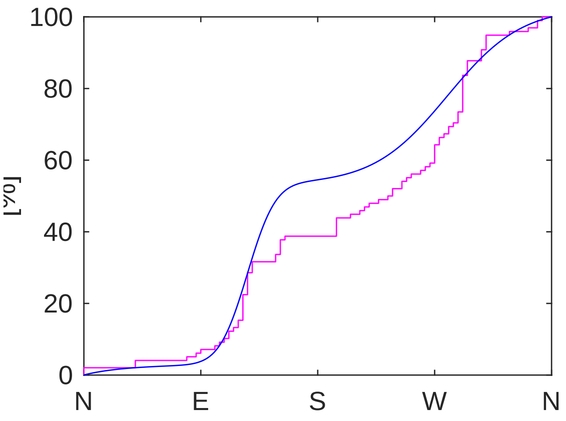

n=98, 3 parts

$p_{Rayleigh\text{-}NU}$ = 0.0079

Best models= M5B (98%)

Best params: ϕ_1=126°, κ_1=11.7

λ=0.33, ϕ_2=280°, κ_2=1.87

p_{KS}=0.00603

Bee D04: angular statistics and path

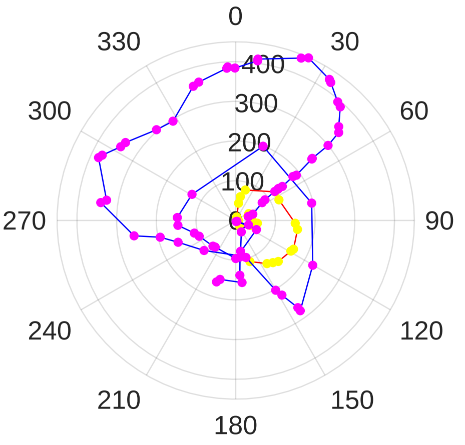

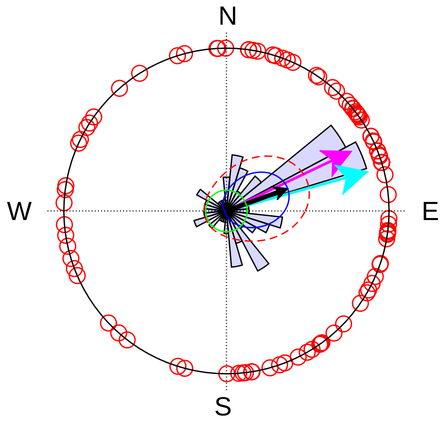

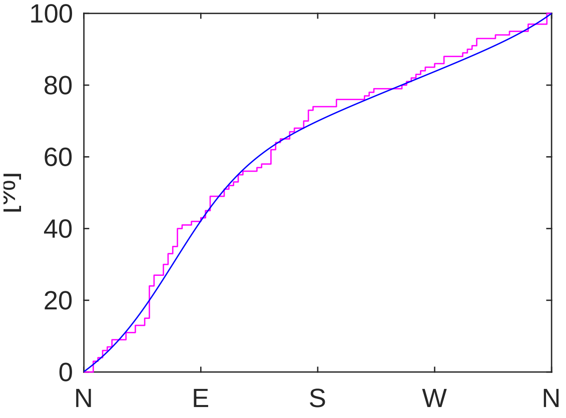

n=100, 2 parts

$p_{Rayleigh\text{-}NU}$ = 9.2e-6

Best models= M2B (46%)
M2A (21%)
M3B (17%)

Best params: ϕ_1=70°, κ_1=1.91

λ=0.5, ϕ_2=NaN°, κ_2=0

p_{KS}=0.605

Bee D05: angular statistics and path

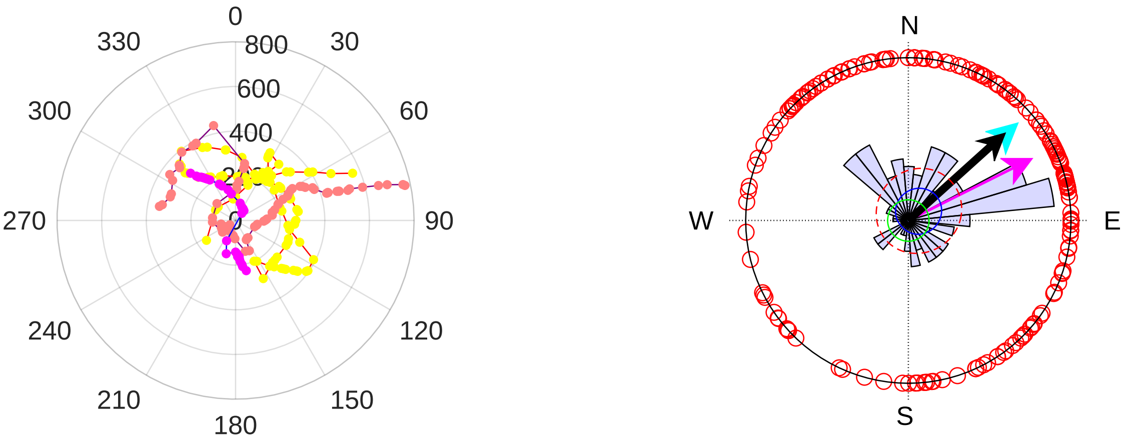

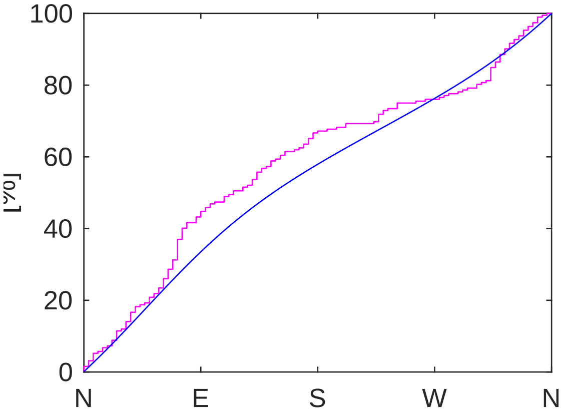

n=192, 3 parts

$p_{Rayleigh\text{-}NU}$ = 3.5e-10

Best models= M2A (60%)
M2C (14%)
M5A (12%)
M2B (10%)

Best params: ϕ_1=48°, κ_1=0.705

λ=1, ϕ_2=NaN°, κ_2=0

p_{KS}=0.0176

Bee D06: angular statistics and path

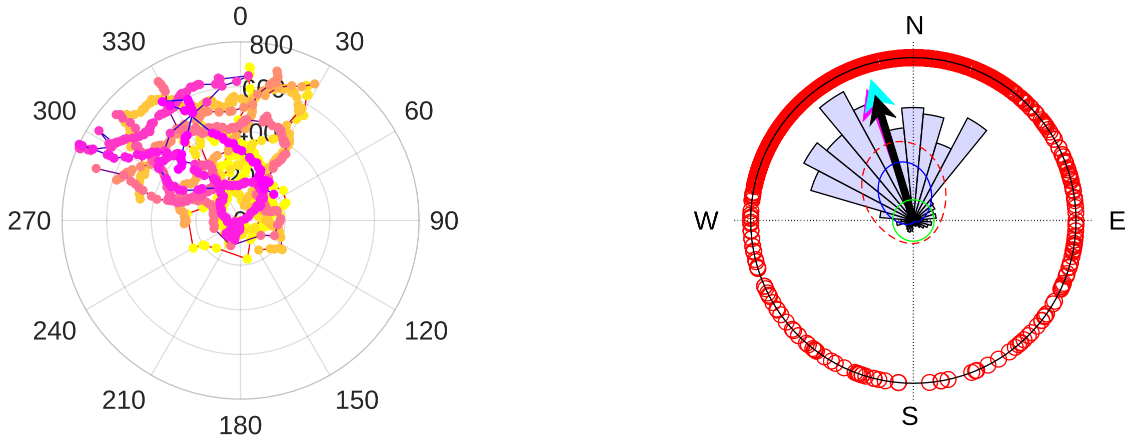

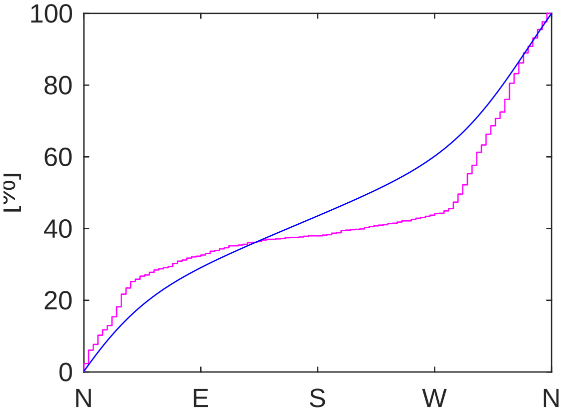

n=935, 10 parts

$p_{\text{Rayleigh-NU}}$ = 3.1e-185

Best models= M2A (99%)

Best params: ϕ_1=343°, κ_1=1.67

λ=1, ϕ_2=NaN°, κ_2=0

p_{KS}=8.19e-30

Bee D07: angular statistics and path

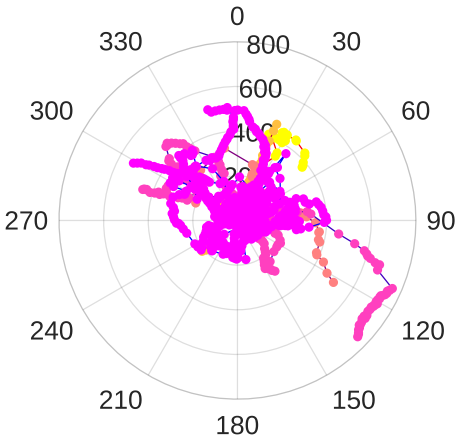

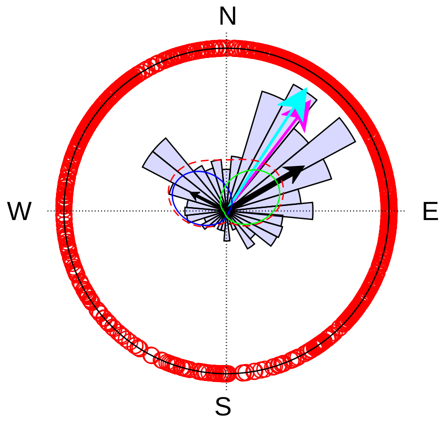

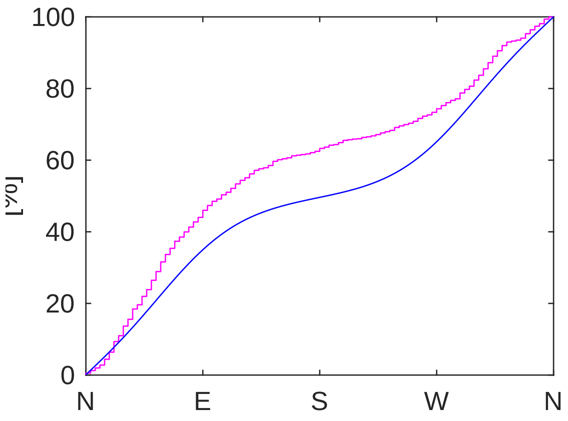

n=1111, 5 parts

$p_{Rayleigh\text{-}NU}$ = 1.7e-62

Best models= M5A (100%)

Best params: ϕ_1=297°, κ_1=1.57

λ=0.31, ϕ_2=60°, κ_2=1.57

p_{KS}=7.64e-20

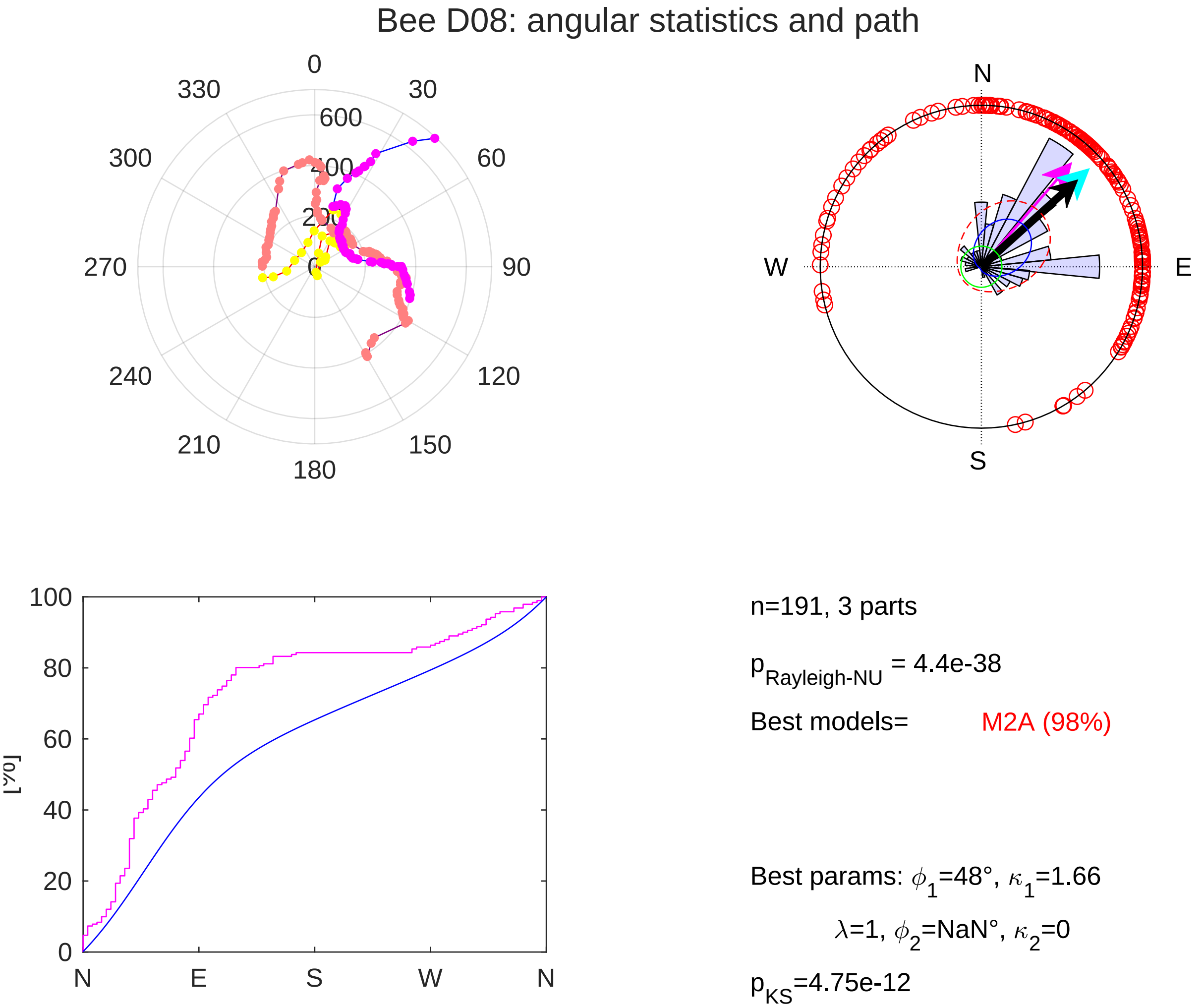
Bee D08: angular statistics and path
0
30
60
90
120
150
180
210
240
270
300
330
600
400
200
0
N
E
S
W
100
80
60
40
20
0
[%]
N
E
S
W
N
n=191, 3 parts
$p_{Rayleigh-NU}$ = 4.4e-38
Best models= M2A (98%)
Best params: ϕ_1=48°, κ_1=1.66
λ=1, ϕ_2=NaN°, κ_2=0
p_{KS}=4.75e-12

Bee D09: angular statistics and path

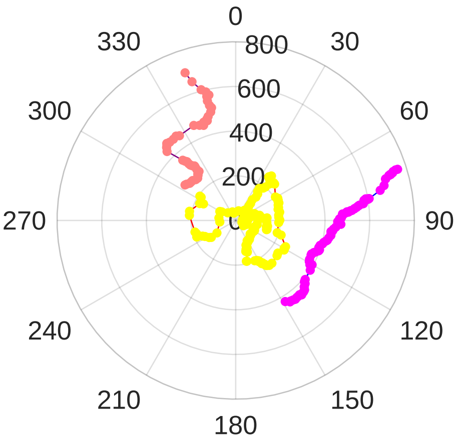

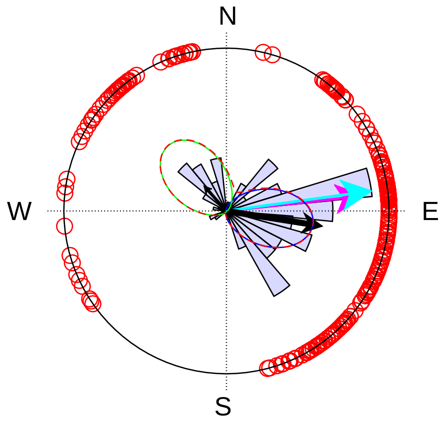

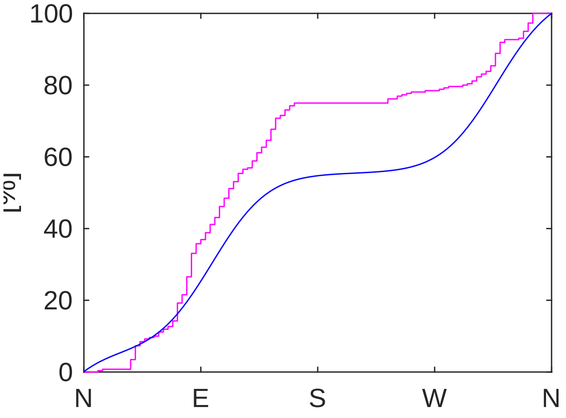

n=260, 3 parts

$p_{Rayleigh\text{-}NU}$ = 2.3e-26

Best models= M5A (71%)
M5B (29%)

Best params: ϕ_1=99°, κ_1=3.13
λ=0.74, ϕ_2=318°, κ_2=3.13

p_{KS}=1.02e-10

Bee D10: angular statistics and path

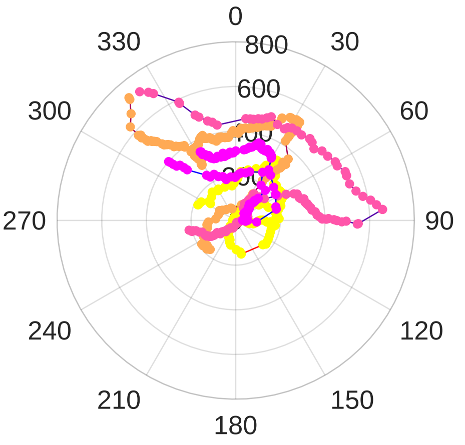

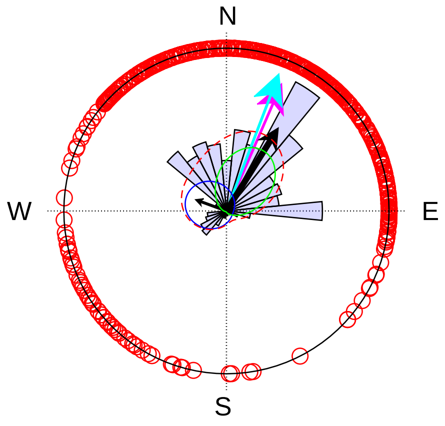

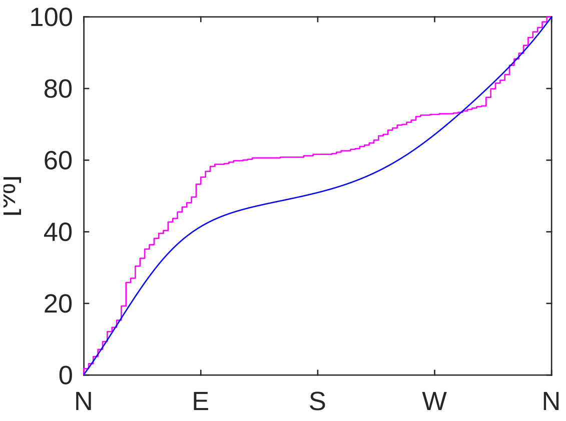

n=503, 4 parts

$p_{Rayleigh-NU}$ = 2e-66

Best models= M5B (100%)

Best params: ϕ_1=290°, κ_1=0.919

λ=0.25, ϕ_2=32°, κ_2=2.17

p_{KS}=3.07e-10

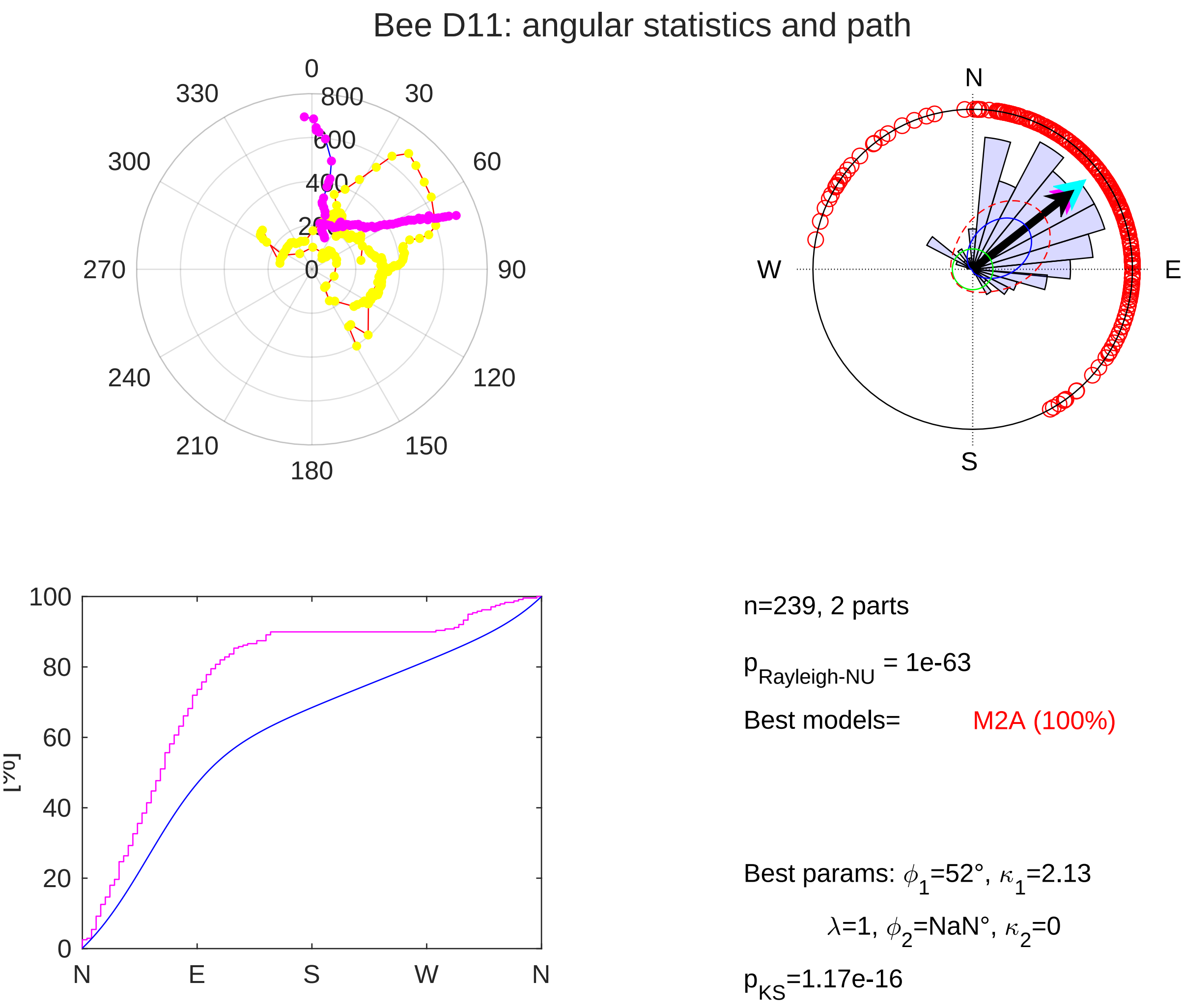
Bee D11: angular statistics and path
0
30
60
90
120
150
180
210
240
270
300
330
800
600
400
200
0
N
E
S
W
100
80
60
40
20
0
[%]
N
E
S
W
N
n=239, 2 parts
p$_{Rayleigh-NU}$ = 1e-63
Best models= M2A (100%)
Best params: ϕ_1=52°, κ_1=2.13
λ=1, ϕ_2=NaN°, κ_2=0
p$_{KS}$=1.17e-16

Bee D12: angular statistics and path

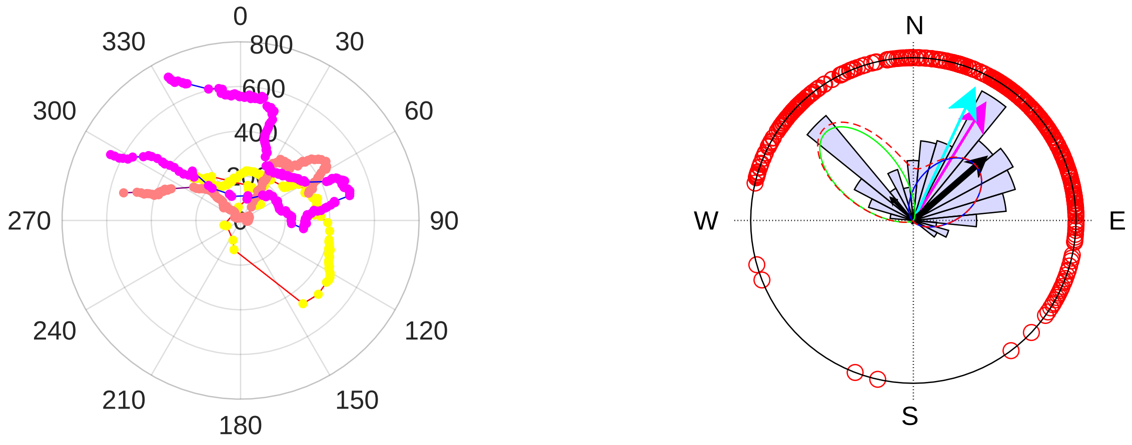

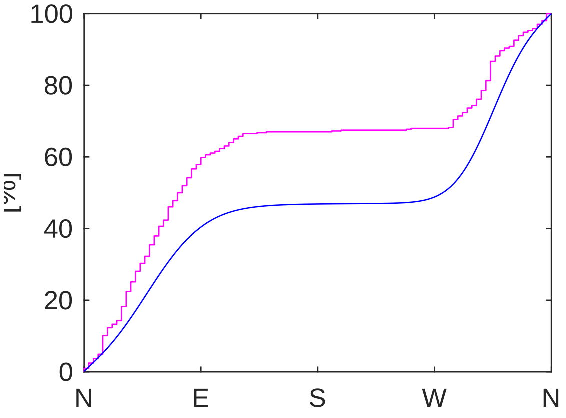

n=406, 3 parts

$p_{Rayleigh-NU}$ = 2.6e-76

Best models= M5B (100%)

Best params: ϕ_1=49°, κ_1=2.87

λ=0.75, ϕ_2=315°, κ_2=5.98

p_{KS}=7.91e-16

Bee D13: angular statistics and path

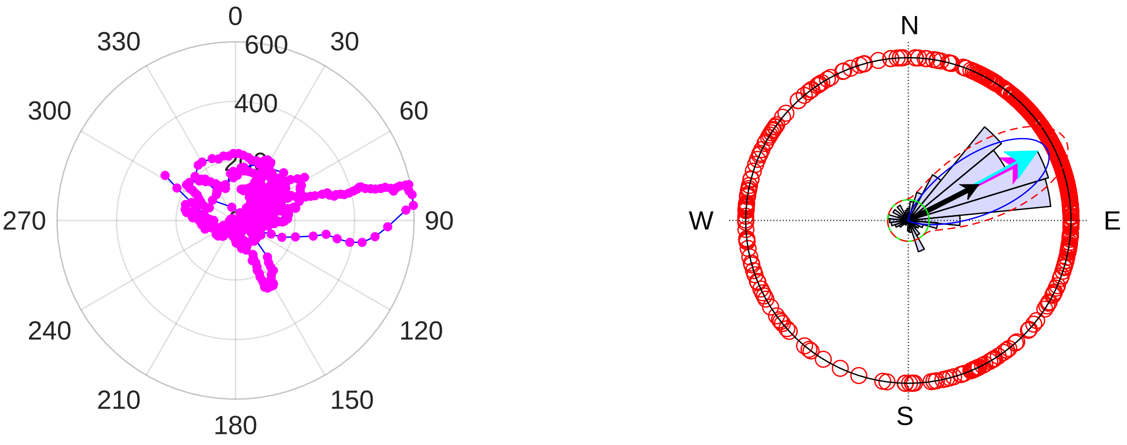

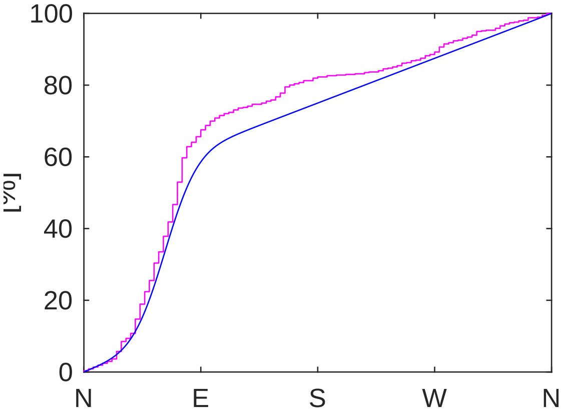

n=576, 1 parts

$p_{Rayleigh\text{-}NU}$ = 1.3e-91

Best models= M2C (74%)
M4B (26%)

Best params: ϕ_1=63°, κ_1=9.28
λ=0.6, ϕ_2=NaN°, κ_2=0

p_{KS}=0.000152

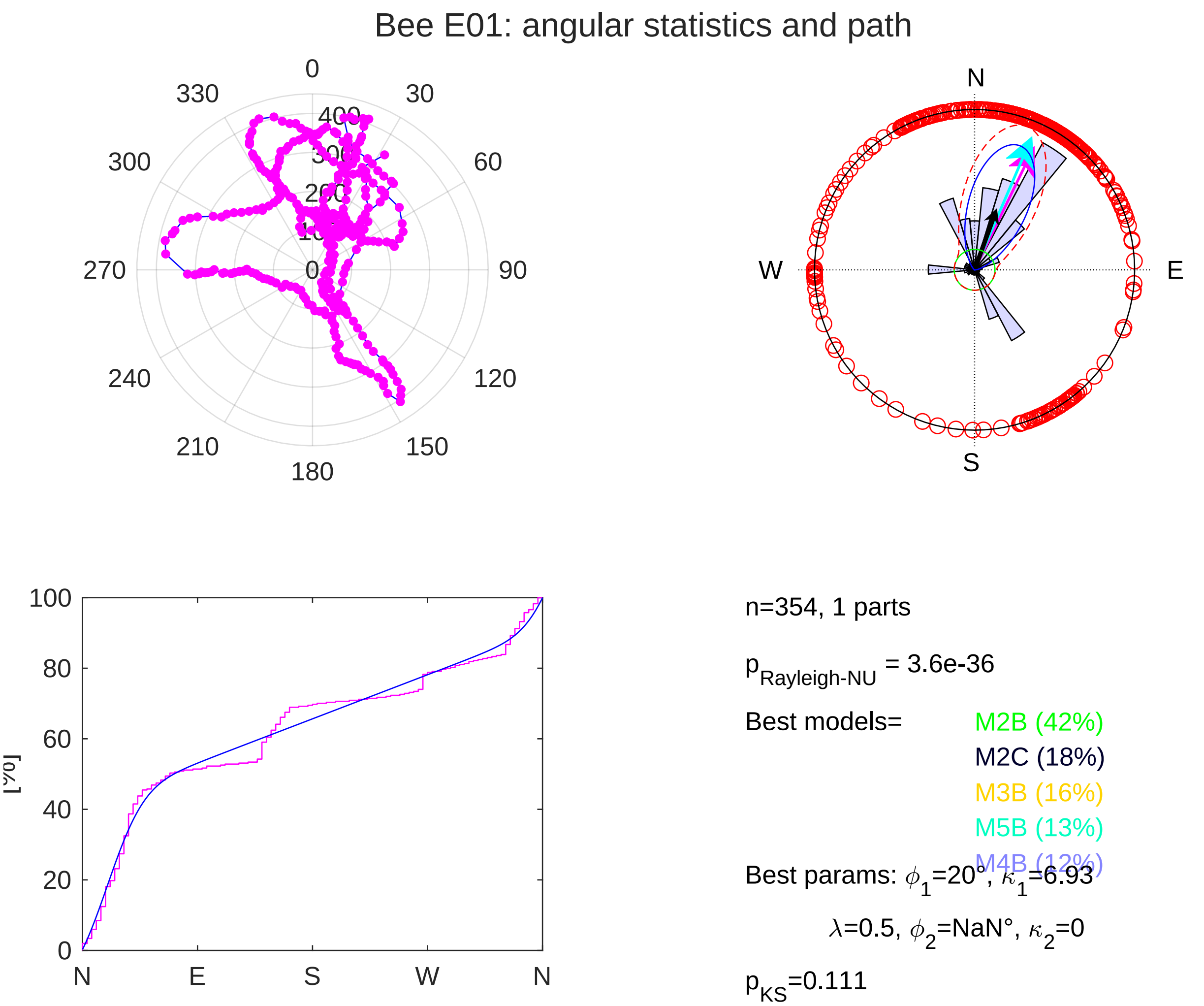

Bee E01: angular statistics and path
0
30
60
90
120
150
180
210
240
270
300
330
N
E
S
W
100
80
60
40
20
0
[%]
n=354, 1 parts
p_Rayleigh-NU = 3.6e-36
Best models=
M2B (42%)
M2C (18%)
M3B (16%)
M5B (13%)
M4B (12%)
Best params: φ_1=20°, κ_1=6.93
λ=0.5, φ_2=NaN°, κ_2=0
p_KS=0.111

Bee E02: angular statistics and path

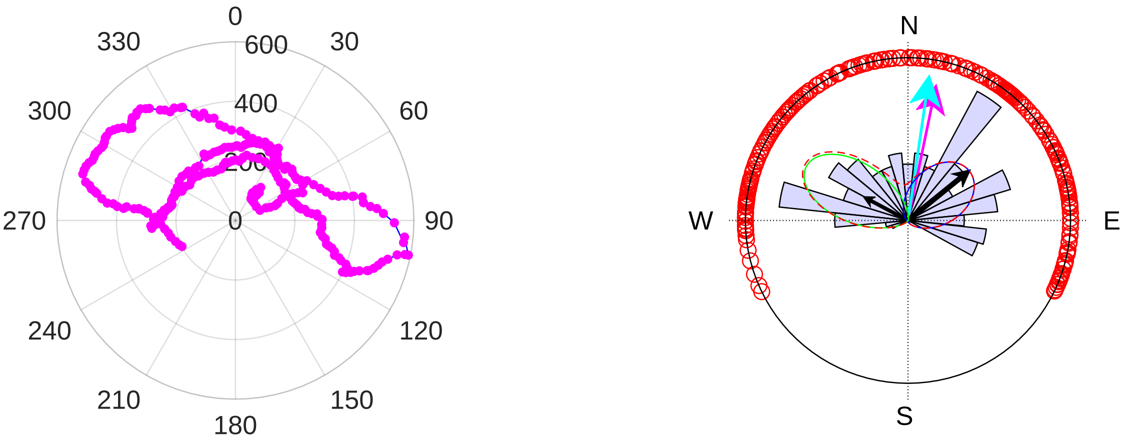

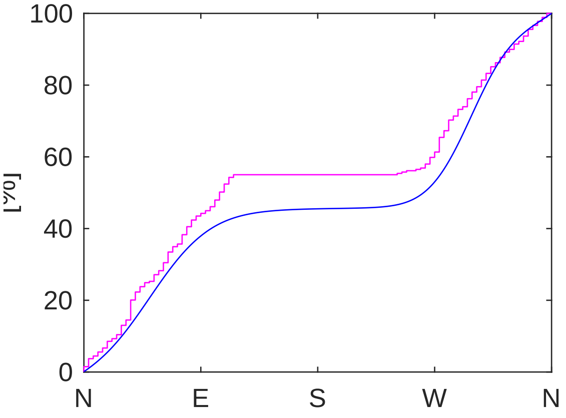

n=269, 1 parts

$p_{Rayleigh\text{-}NU}$ = 1.4e-27

Best models= M5B (67%)
M5A (33%)

Best params: ϕ_1=51°, κ_1=2.62
λ=0.61, ϕ_2=298°, κ_2=5.18

p_{KS}=0.000758

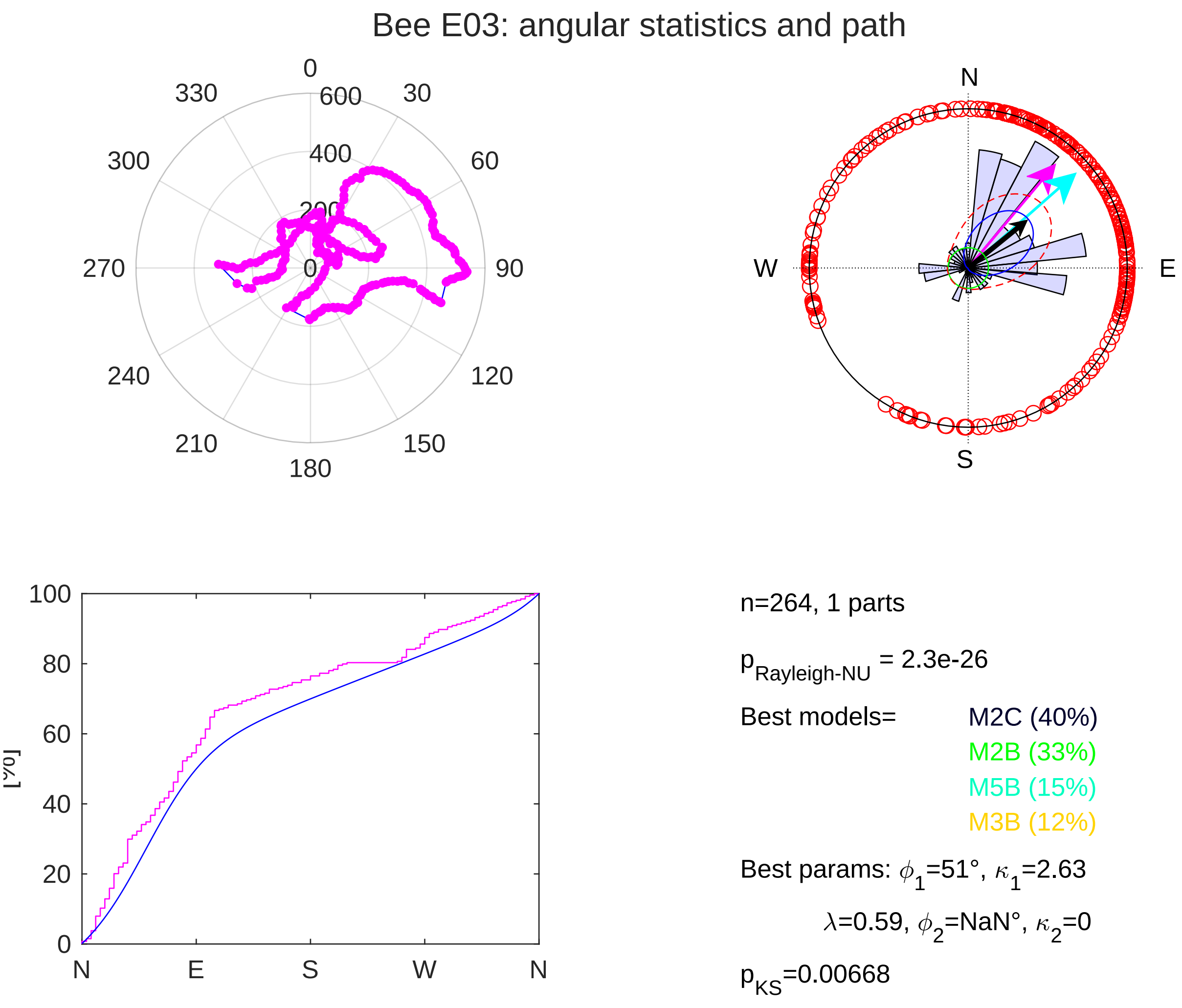
Bee E03: angular statistics and path
0
30
60
90
120
150
180
210
240
270
300
330
600
400
200
0
N
E
S
W
100
80
60
40
20
0
[%]
N
E
S
W
N
n=264, 1 parts
p$_{Rayleigh-NU}$ = 2.3e-26
Best models=
M2C (40%)
M2B (33%)
M5B (15%)
M3B (12%)
Best params: ϕ_1=51°, κ_1=2.63
λ=0.59, ϕ_2=NaN°, κ_2=0
p$_{KS}$=0.00668

Bee E04: angular statistics and path

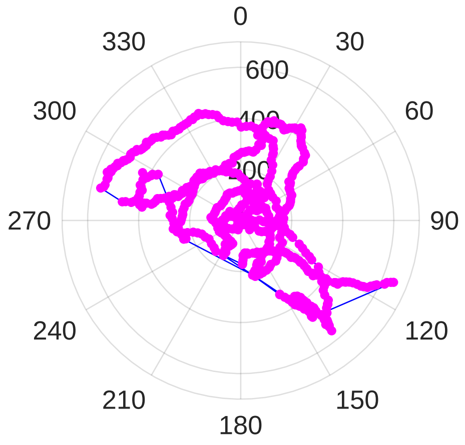

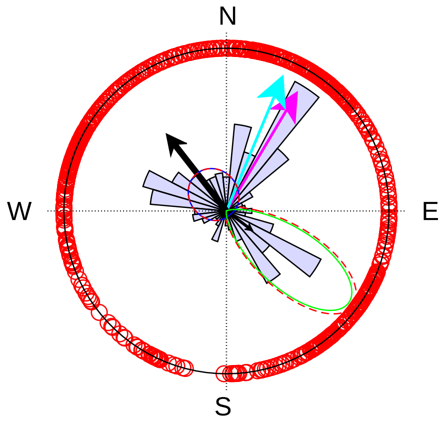

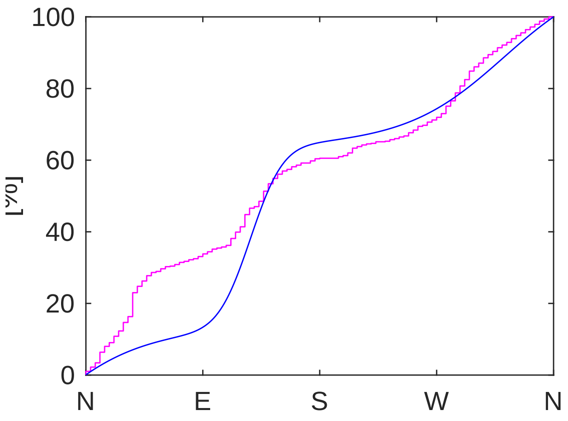

n=674, 1 parts

$p_{Rayleigh\text{-}NU}$ = 1.7e-12

Best models= M5B (94%)

Best params: ϕ_1=322°, κ_1=1.13

λ=0.75, ϕ_2=127°, κ_2=8.93

p_{KS}=8.1e-25

Bee E05: angular statistics and path

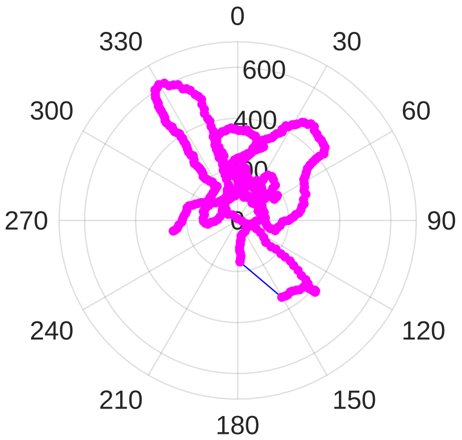

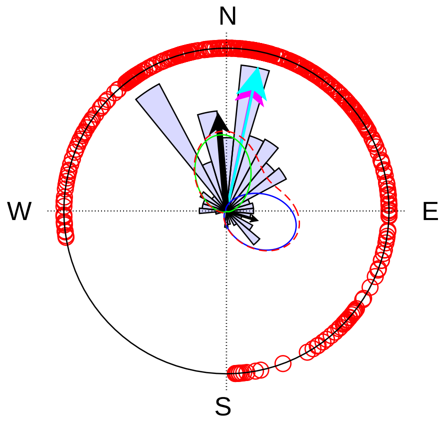

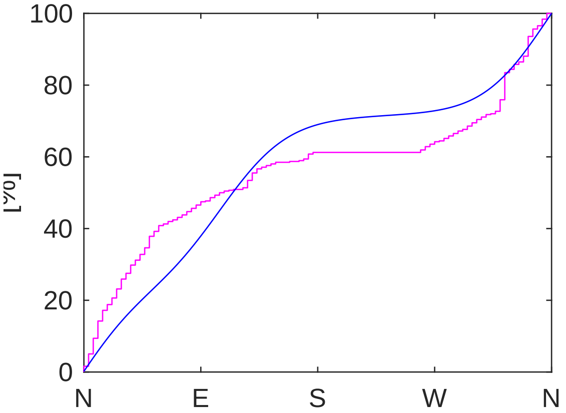

n=436, 1 parts

$p_{Rayleigh\text{-}NU}$ = 1.1e-67

Best models= M5B (100%)

Best params: ϕ_1=107°, κ_1=2.24

λ=0.25, ϕ_2=355°, κ_2=2.48

p_{KS}=5.33e-09

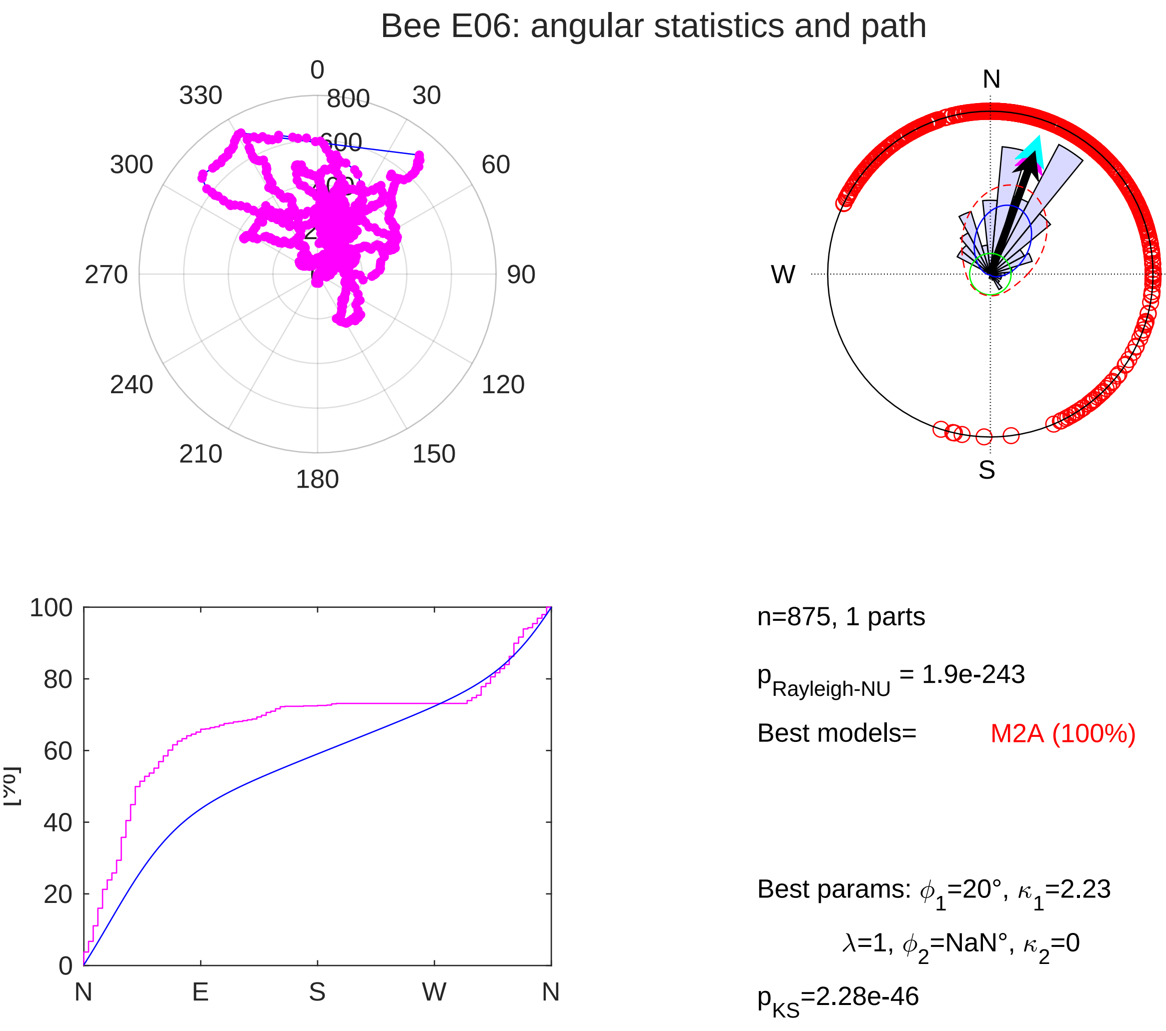
Bee E06: angular statistics and path
0
30
60
90
120
150
180
210
240
270
300
330
800
600
N
E
S
W
100
80
60
40
20
0
[%]
N
E
S
W
N
n=875, 1 parts
$p_{Rayleigh-NU}$ = 1.9e-243
Best models= M2A (100%)
Best params: ϕ_1=20°, κ_1=2.23
λ=1, ϕ_2=NaN°, κ_2=0
p_{KS}=2.28e-46

Bee E07: angular statistics and path

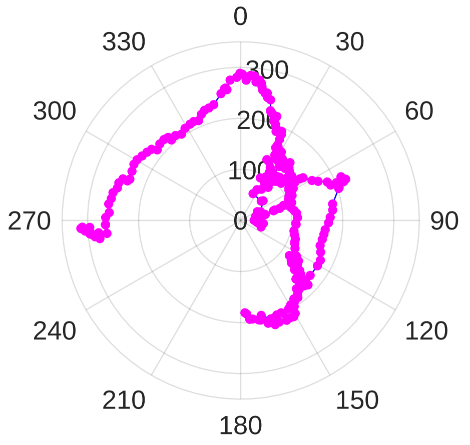

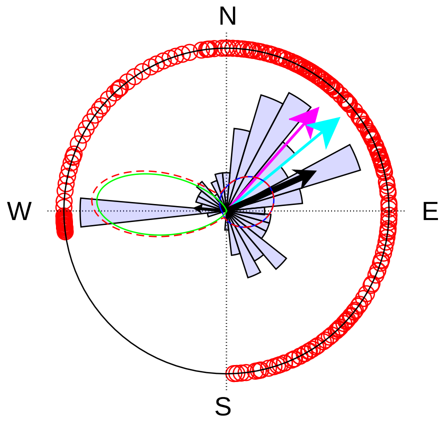

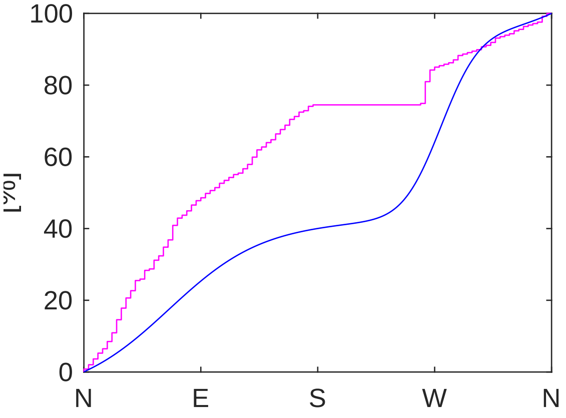

n=247, 1 parts

$p_{Rayleigh\text{-}NU}$ = 8.3e-16

Best models= M5B (80%)

Best params: ϕ_1=66°, κ_1=1.22

λ=0.75, ϕ_2=275°, κ_2=6.54

p_{KS}=1.65e-26

Bee R01: angular statistics and path

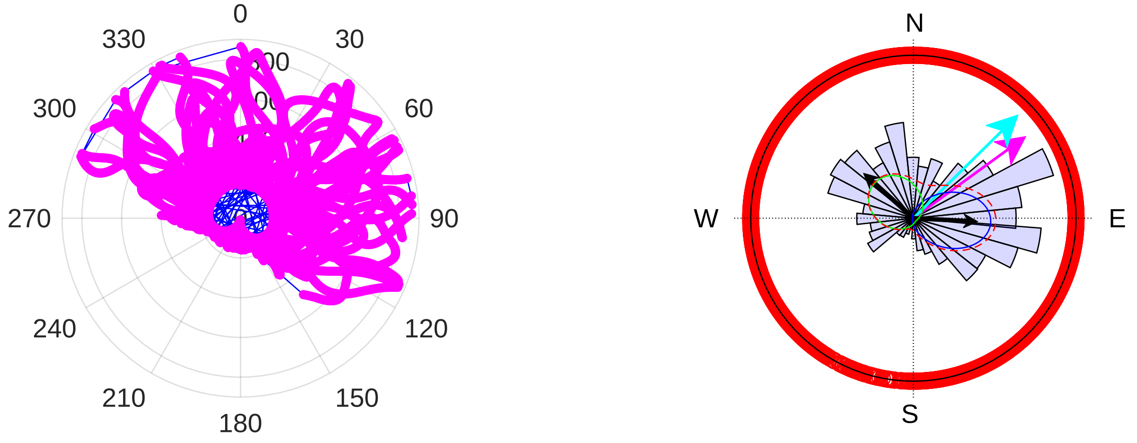

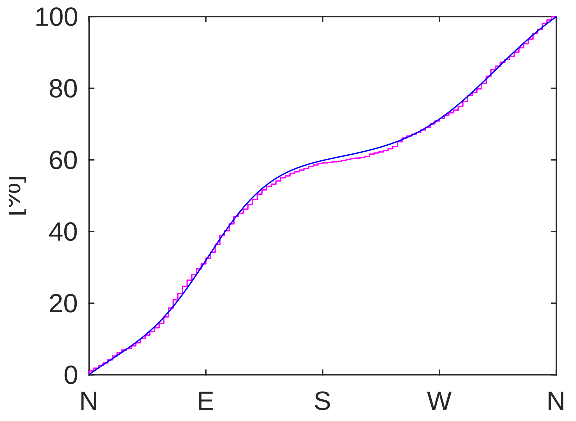

n=7487, 1 parts

$p_{Rayleigh\text{-}NU}$ = 1.4e-183

Best models= M5B (100%)

Best params: ϕ_1=93°, κ_1=2.54

λ=0.49, ϕ_2=312°, κ_2=1.32

p_{KS}=0.00387

Bee R02: angular statistics and path

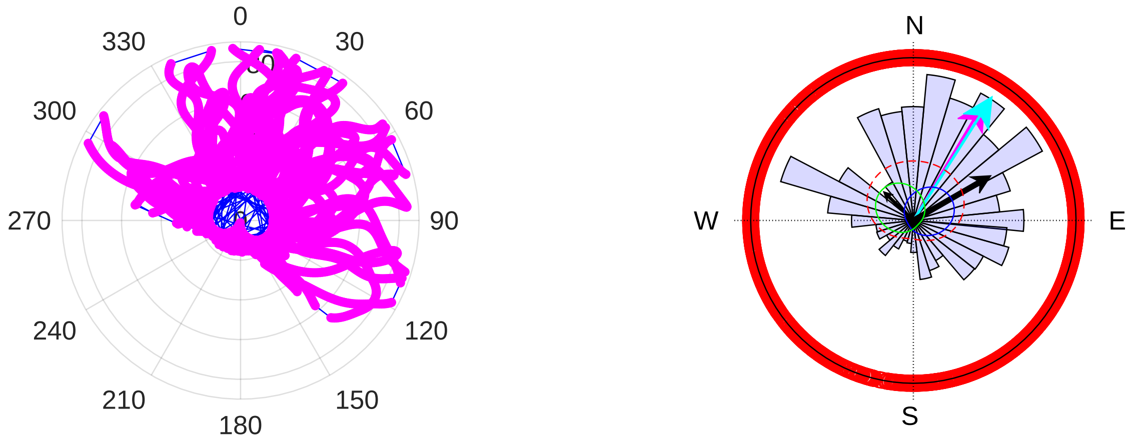

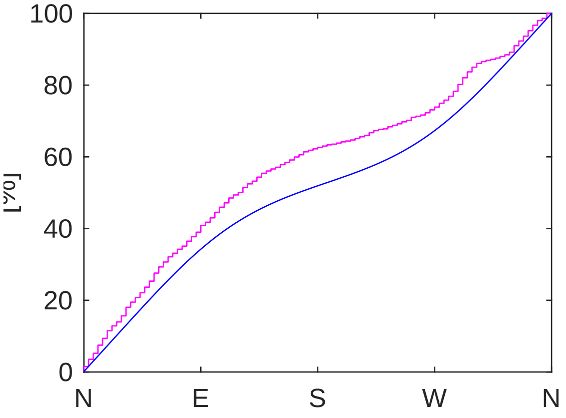

n=7091, 1 parts

$p_{Rayleigh\text{-}NU}$ = 1.1e-270

Best models= M5A (100%)

Best params: ϕ_1=60°, κ_1=0.971

λ=0.69, ϕ_2=314°, κ_2=0.971

p_{KS}=2.15e-67

Bee R03: angular statistics and path

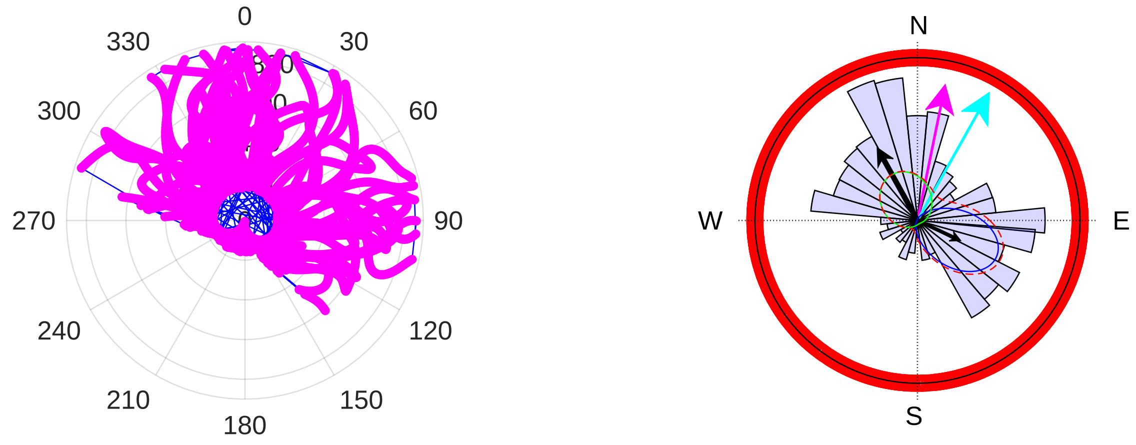

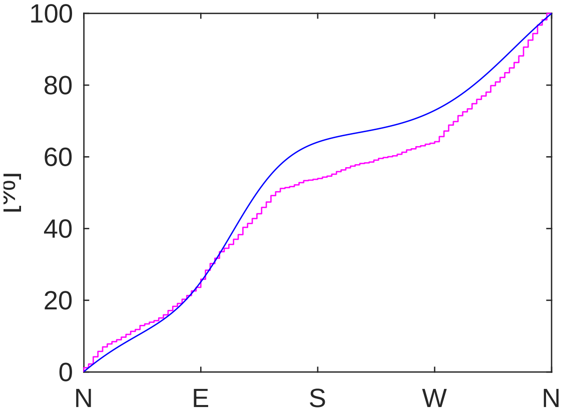

n=7171, 1 parts

$p_{Rayleigh\text{-}NU}$ = 1.3e-137

Best models= M5B (100%)

Best params: ϕ_1=115°, κ_1=3.11

λ=0.36, ϕ_2=331°, κ_2=1.32

p_{KS}=4.04e-71

Bee R04: angular statistics and path

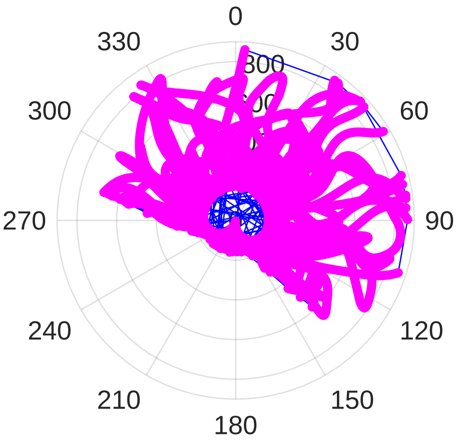

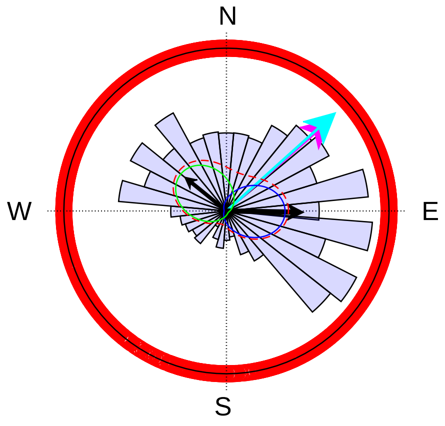

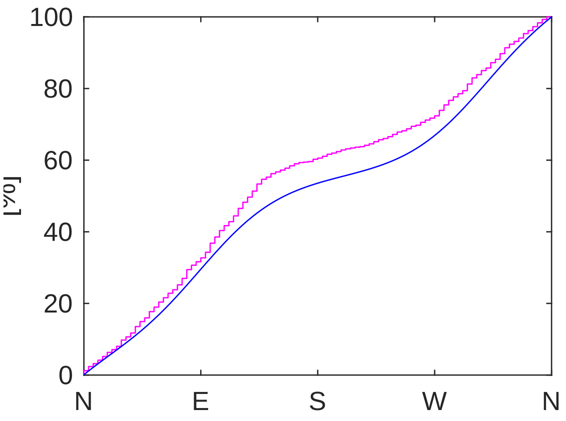

n=7185, 1 parts

$p_{Rayleigh\text{-}NU}$ = 9.8e-167

Best models= M5A (100%)

Best params: ϕ_1=91°, κ_1=1.6

λ=0.59, ϕ_2=309°, κ_2=1.6

p_{KS}=3.34e-37

Bee R05: angular statistics and path

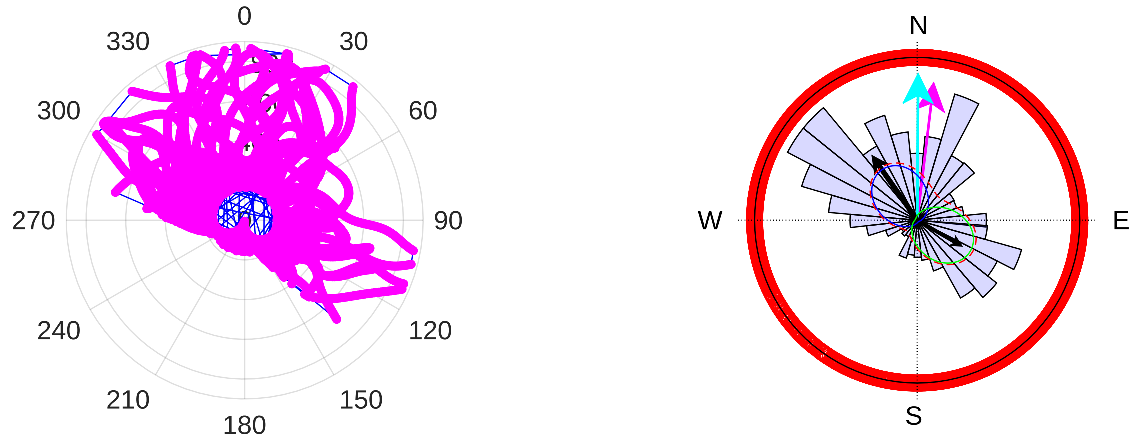

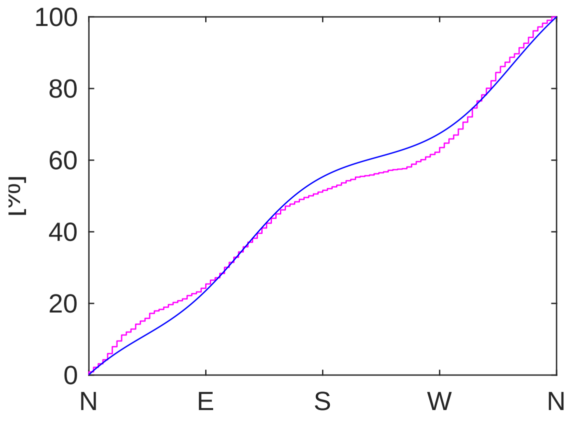

n=7039, 1 parts

$p_{Rayleigh\text{-}NU}$ = 1.2e-109

Best models= M5A (100%)

Best params: ϕ_1=325°, κ_1=1.73

λ=0.6, ϕ_2=120°, κ_2=1.73

p_{KS}=2.08e-20

Bee R06: angular statistics and path

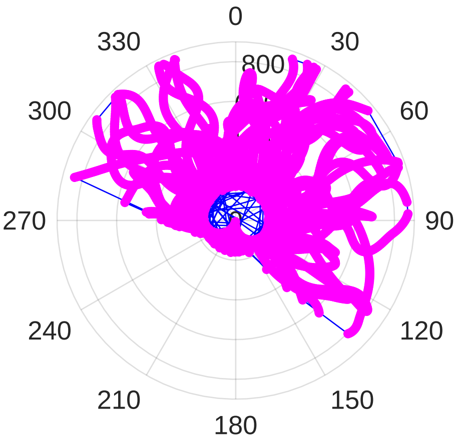

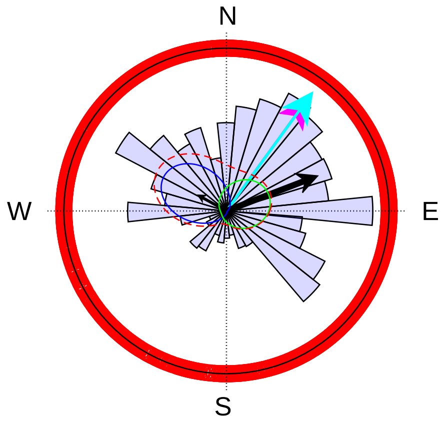

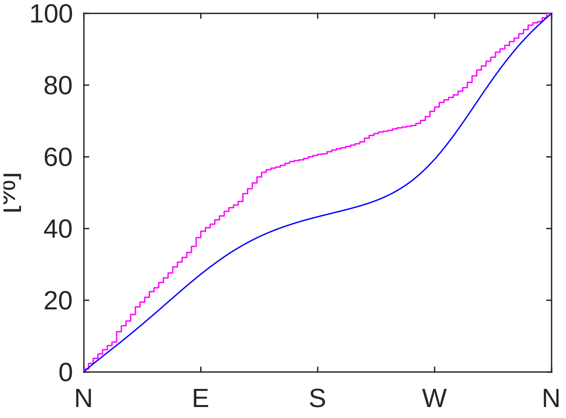

n=7174, 1 parts

$p_{Rayleigh\text{-}NU}$ = 1.5e-224

Best models= M5B (100%)

Best params: ϕ_1=299°, κ_1=2.22

λ=0.25, ϕ_2=69°, κ_2=1.03

p_{KS}=5.31e-216

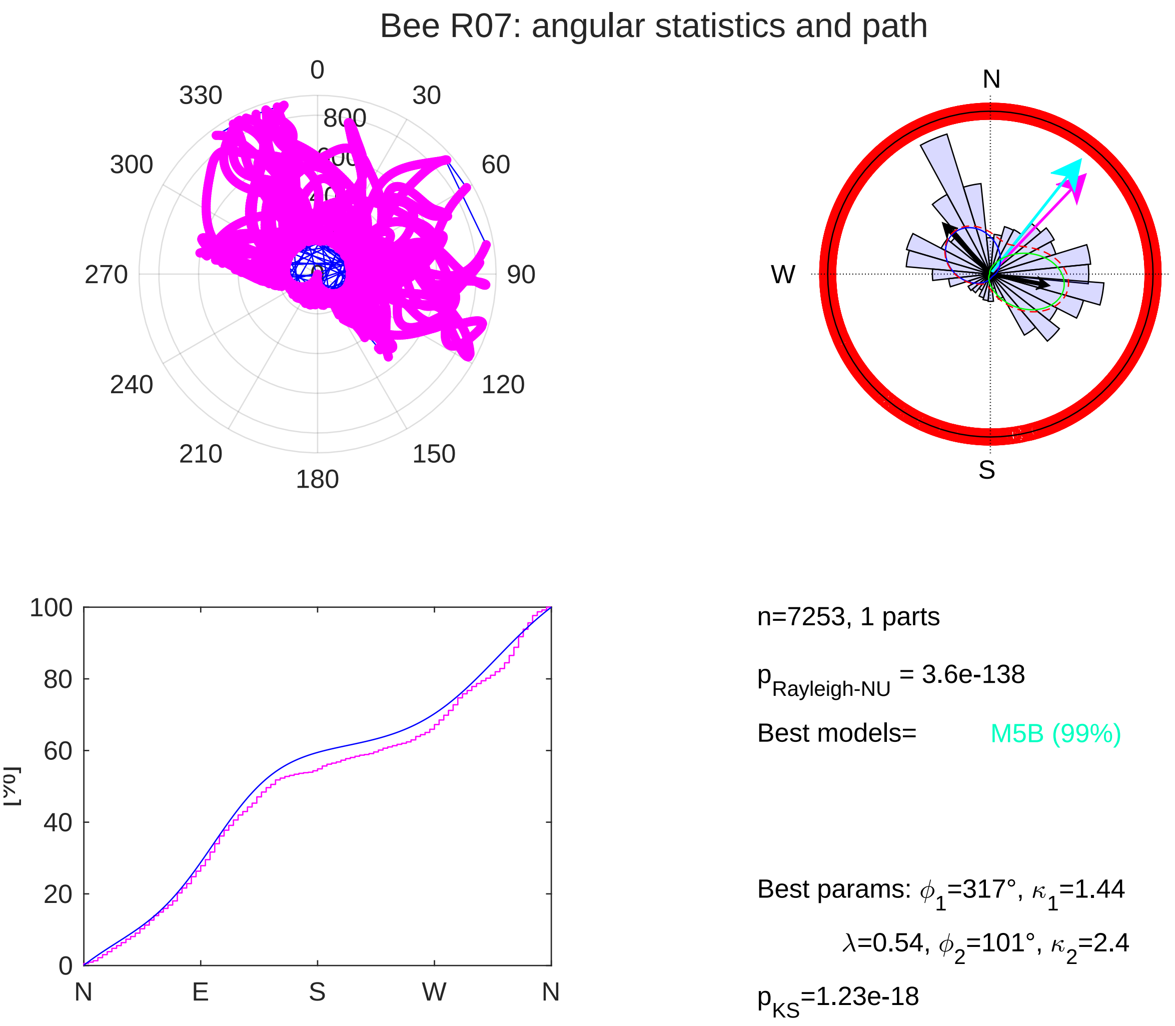
Bee R07: angular statistics and path
0
30
60
90
120
150
180
210
240
270
300
330
800
N
E
S
W
100
80
60
40
20
0
[%]
N
E
S
W
N
n=7253, 1 parts
$p_{Rayleigh\text{-}NU}$ = 3.6e-138
Best models= M5B (99%)
Best params: ϕ_1=317°, κ_1=1.44
λ=0.54, ϕ_2=101°, κ_2=2.4
p_{KS}=1.23e-18

Bee R08: angular statistics and path

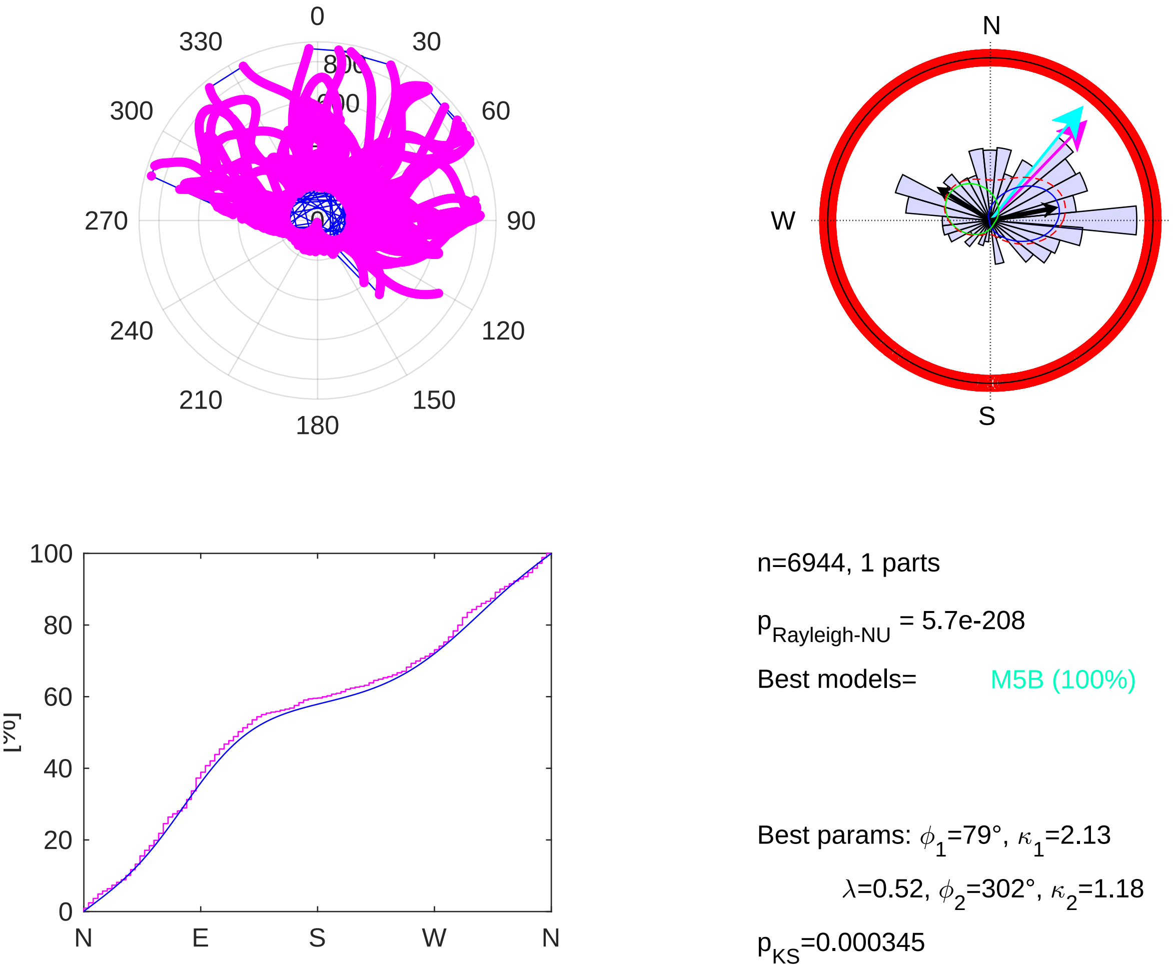

n=6944, 1 parts

$p_{Rayleigh\text{-}NU}$ = 5.7e-208

Best models= M5B (100%)

Best params: ϕ_1=79°, κ_1=2.13

λ=0.52, ϕ_2=302°, κ_2=1.18

p_{KS}=0.000345

Bee R09: angular statistics and path

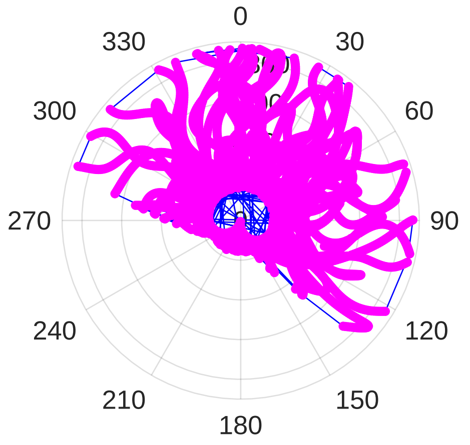

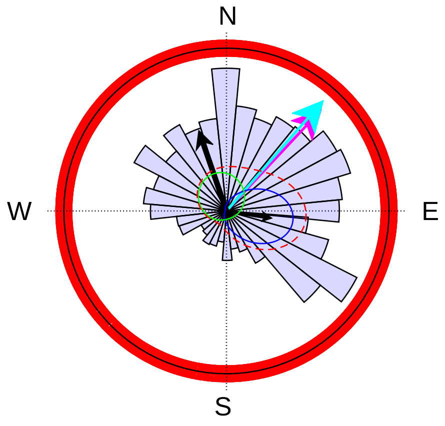

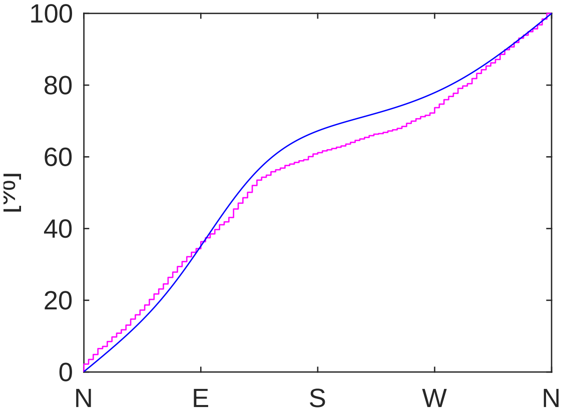

n=6967, 1 parts

$p_{Rayleigh-NU}$ = 1.4e-191

Best models= M5B (83%)
M5A (17%)

Best params: ϕ_1=99°, κ_1=2

λ=0.35, ϕ_2=341°, κ_2=0.791

p_{KS}=5.53e-30

Bee R10: angular statistics and path

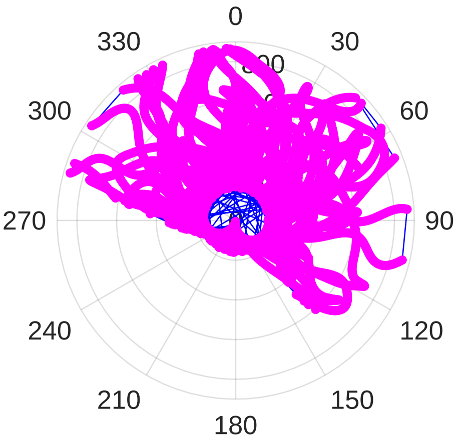

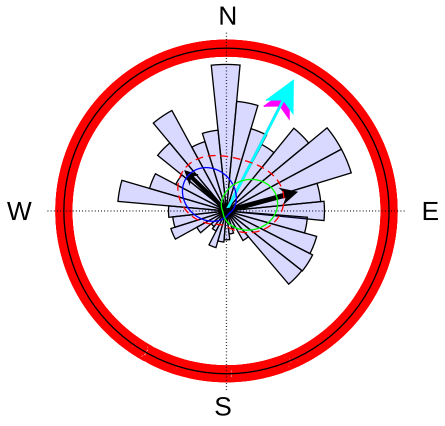

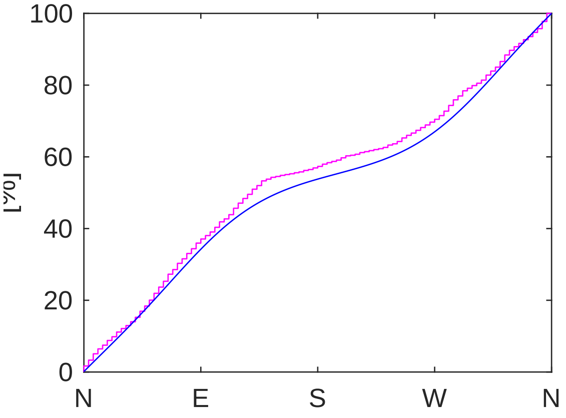

n=7150, 1 parts

$p_{Rayleigh\text{-}NU}$ = 5.6e-242

Best models= M5A (100%)

Best params: ϕ_1=314°, κ_1=1.32

λ=0.44, ϕ_2=75°, κ_2=1.32

p_{KS}=1.09e-15

Bee R11: angular statistics and path

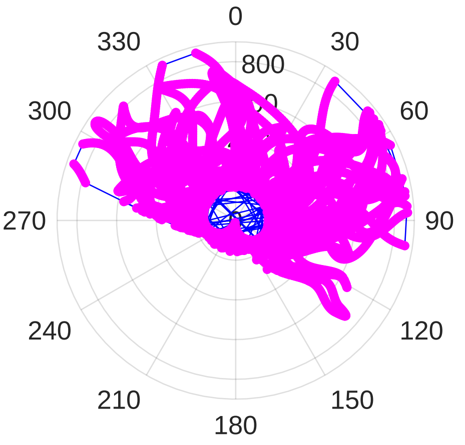

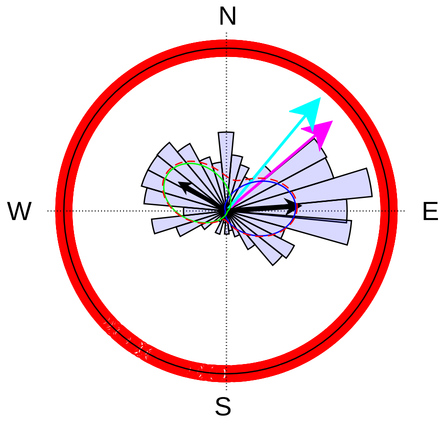

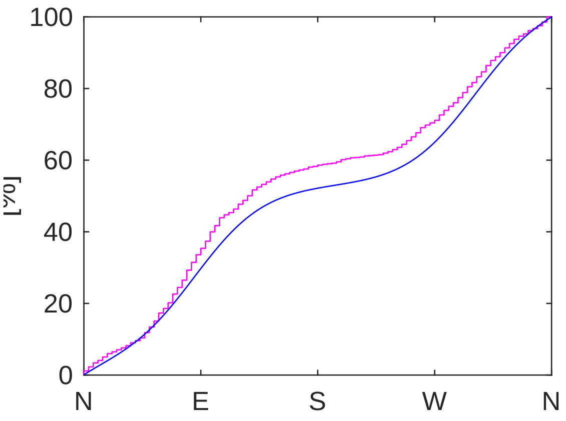

n=7575, 1 parts

$p_{Rayleigh\text{-}NU}$ = 1.4e-189

Best models= M5A (99%)

Best params: ϕ_1=86°, κ_1=2.11

λ=0.57, ϕ_2=301°, κ_2=2.11

p_{KS}=3.35e-30

Bee R12: angular statistics and path

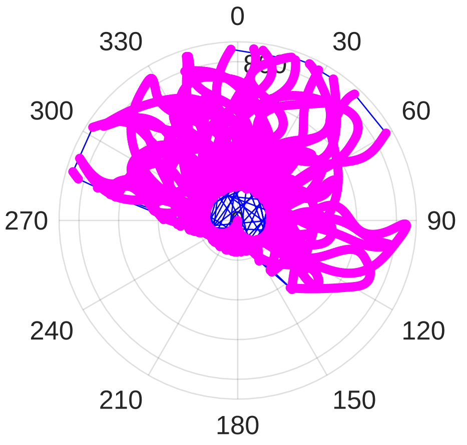

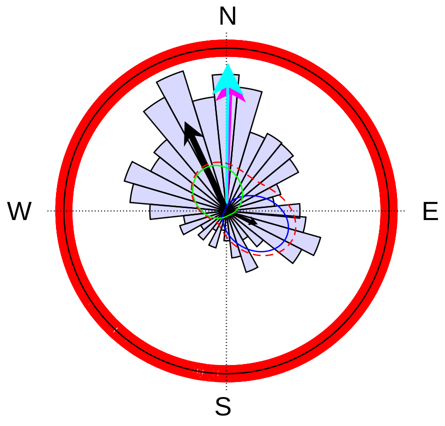

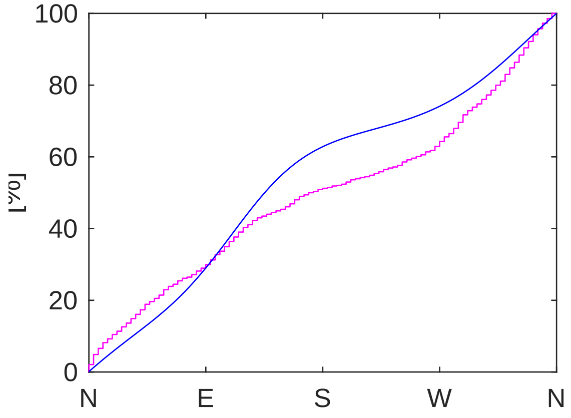

n=6863, 1 parts

$p_{Rayleigh\text{-}NU}$ = 1.5e-221

Best models= M5B (100%)

Best params: ϕ_1=113°, κ_1=1.92

λ=0.25, ϕ_2=335°, κ_2=1.14

p_{KS}=7.61e-103

Bee R13: angular statistics and path

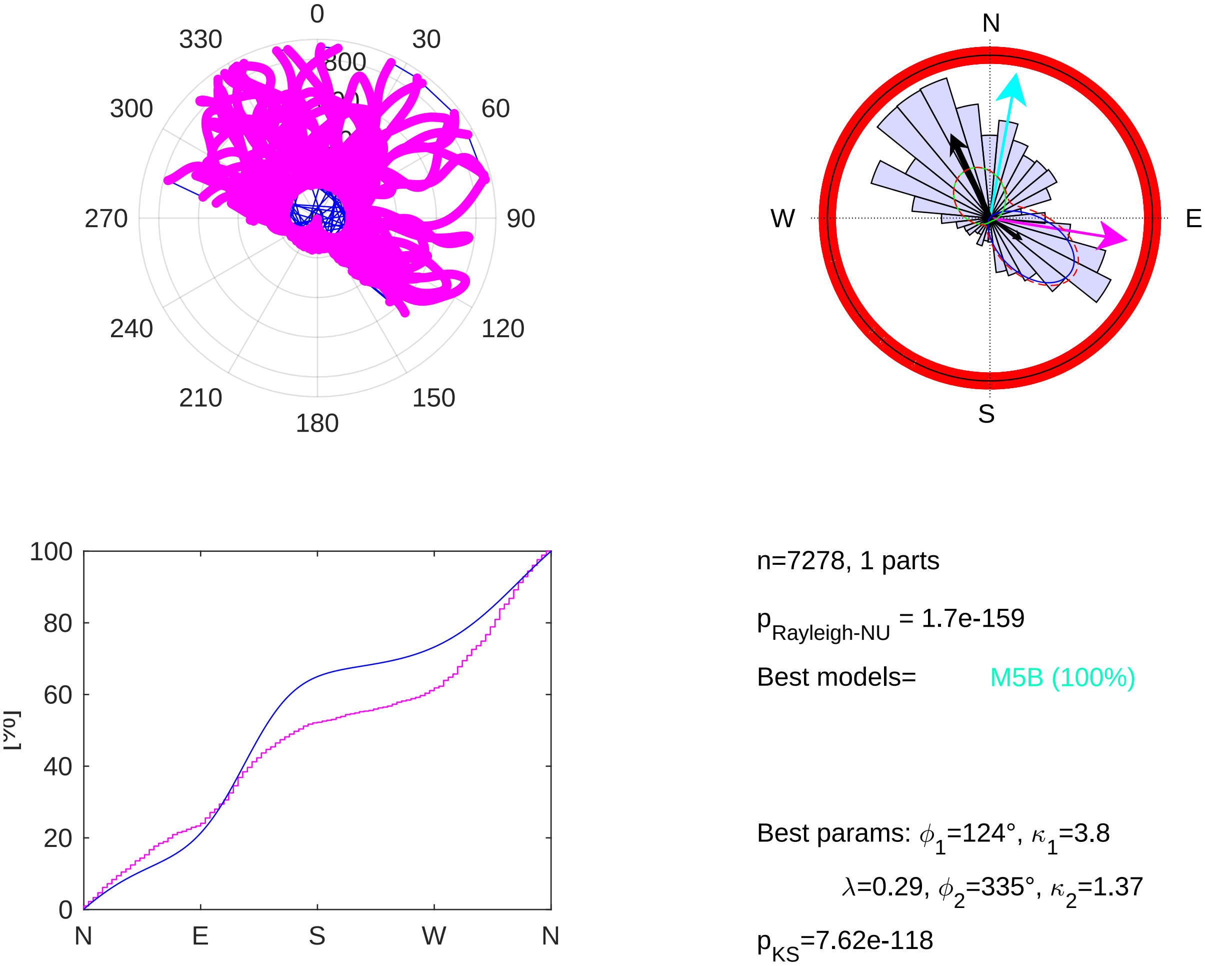

Bee R14: angular statistics and path

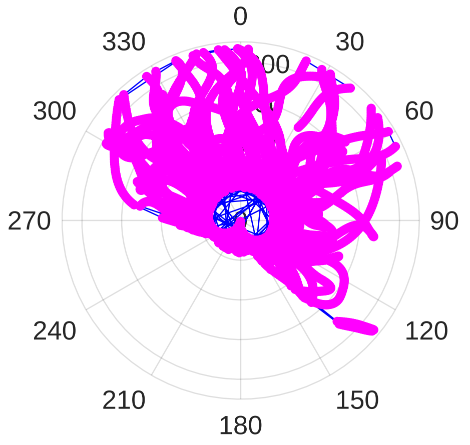

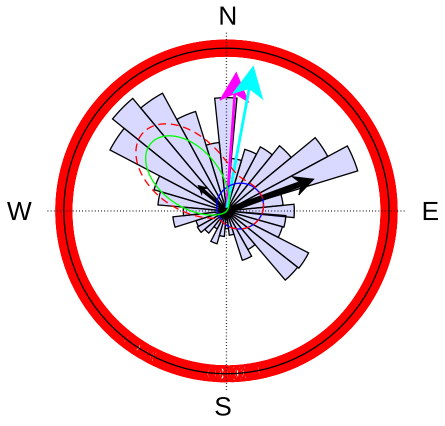

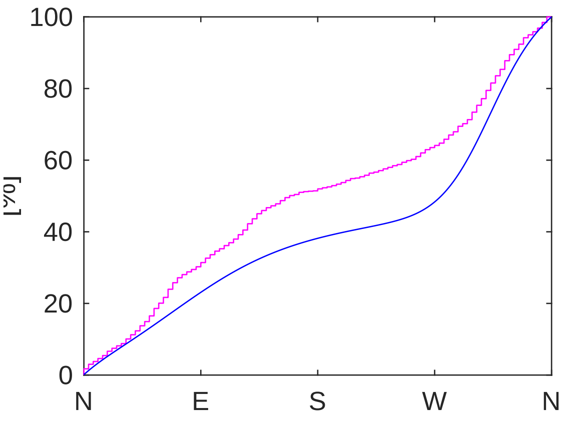

n=7233, 1 parts

$p_{Rayleigh-NU}$ = 5.6e-211

Best models= M5B (100%)

Best params: ϕ_1=70°, κ_1=0.738

λ=0.71, ϕ_2=312°, κ_2=4.1

p_{KS}=1.46e-156

Bee R15: angular statistics and path

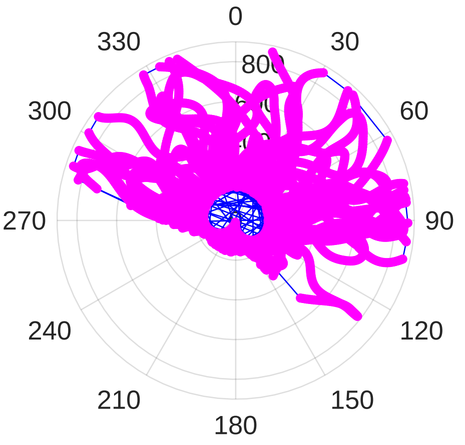

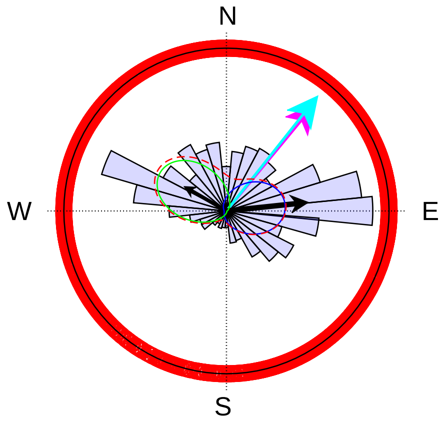

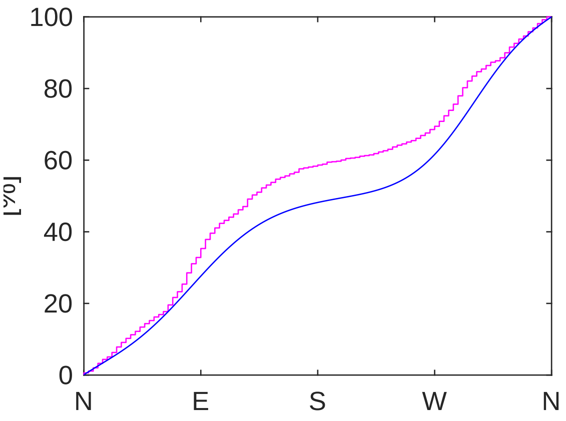

n=7345, 1 parts

$p_{Rayleigh\text{-}NU}$ = 2.5e-176

Best models= M5B (97%)

Best params: ϕ_1=84°, κ_1=1.62

λ=0.63, ϕ_2=300°, κ_2=2.49

p_{KS}=1.56e-71

Bee R16: angular statistics and path

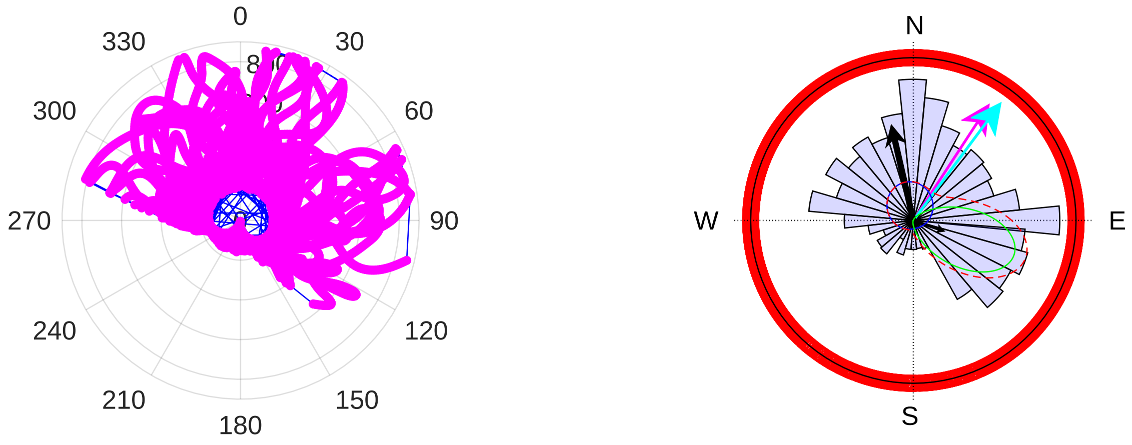

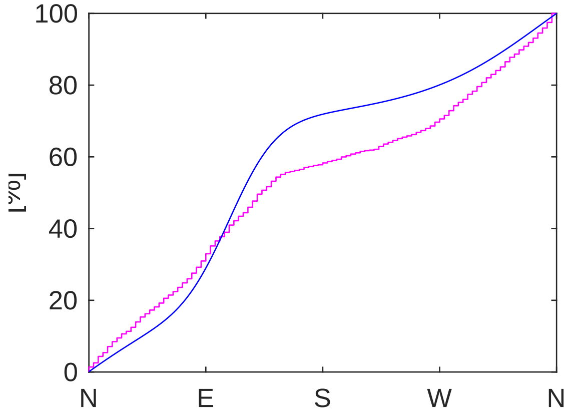

n=7178, 1 parts

$p_{Rayleigh-NU}$ = 2.9e-187

Best models= M5B (100%)

Best params: ϕ_1=347°, κ_1=0.782

λ=0.75, ϕ_2=109°, κ_2=4.48

p_{KS}=1.82e-125

Bee S01: angular statistics and path

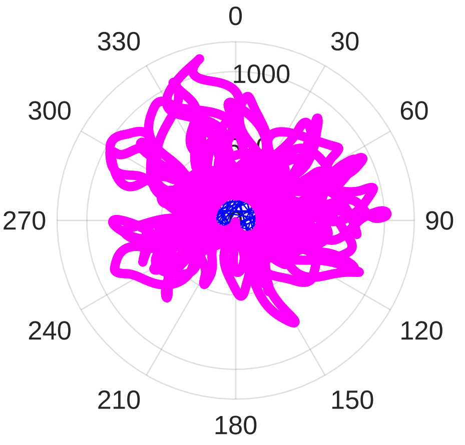

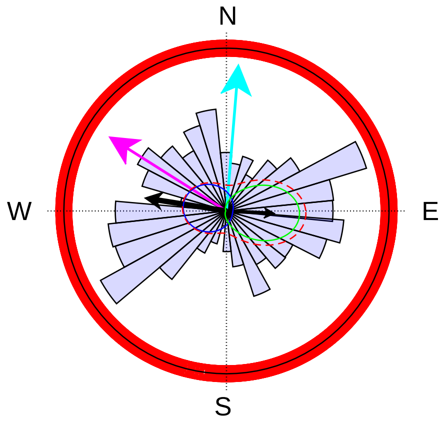

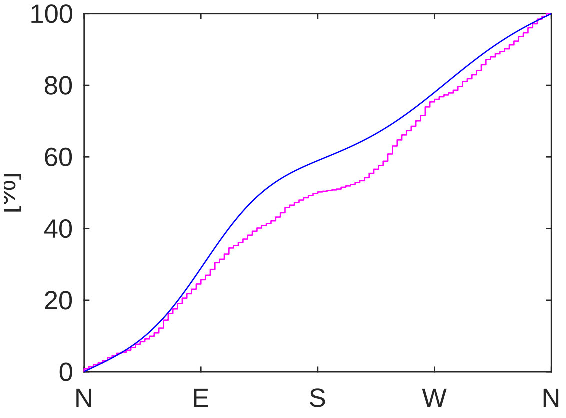

n=10002, 1 parts

$p_{Rayleigh\text{-}NU}$ = 0.00017

Best models= M5B (100%)

Best params: ϕ_1=279°, κ_1=0.961

λ=0.63, ϕ_2=93°, κ_2=2.31

p_{KS}=3.1e-114

Bee S02: angular statistics and path

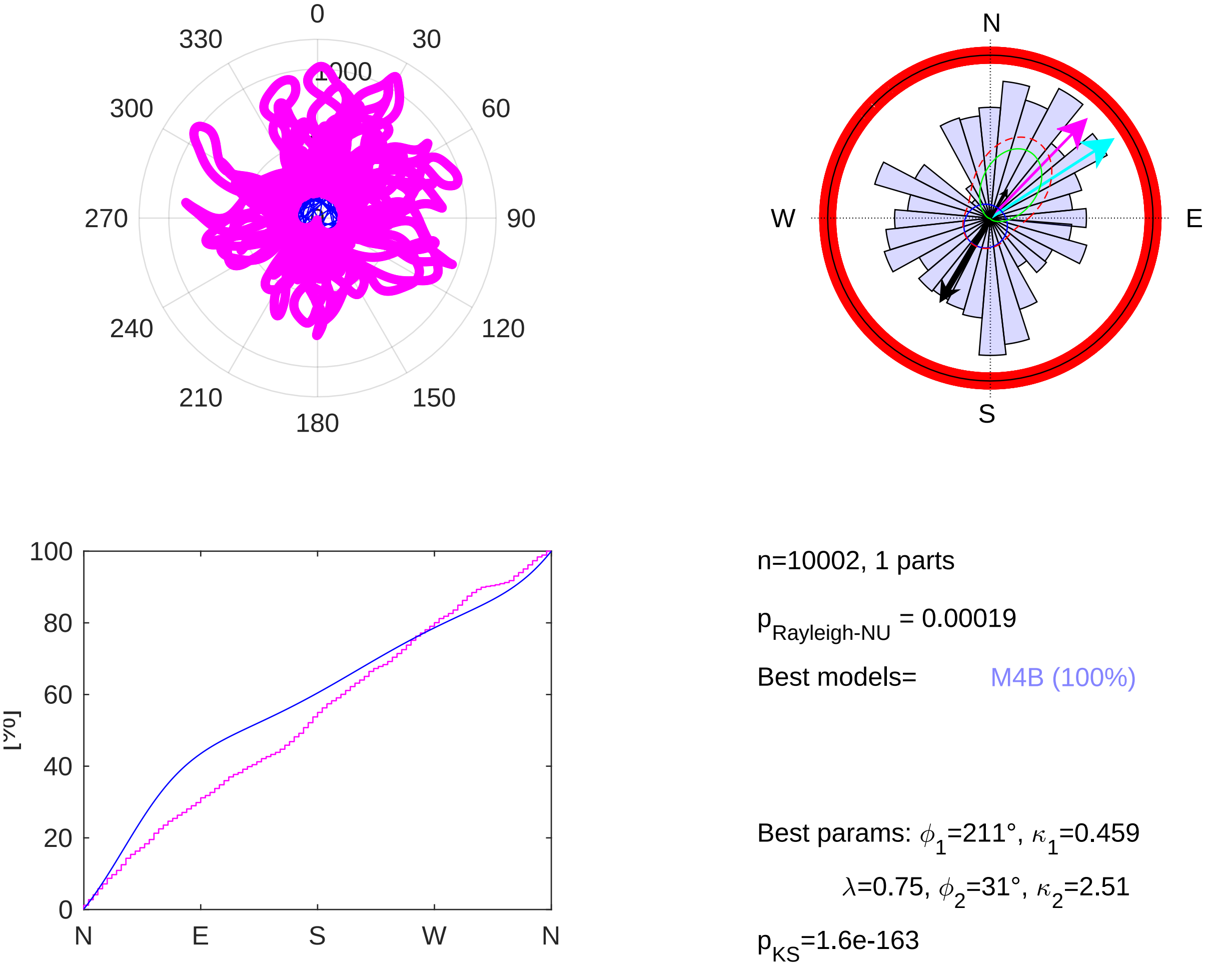

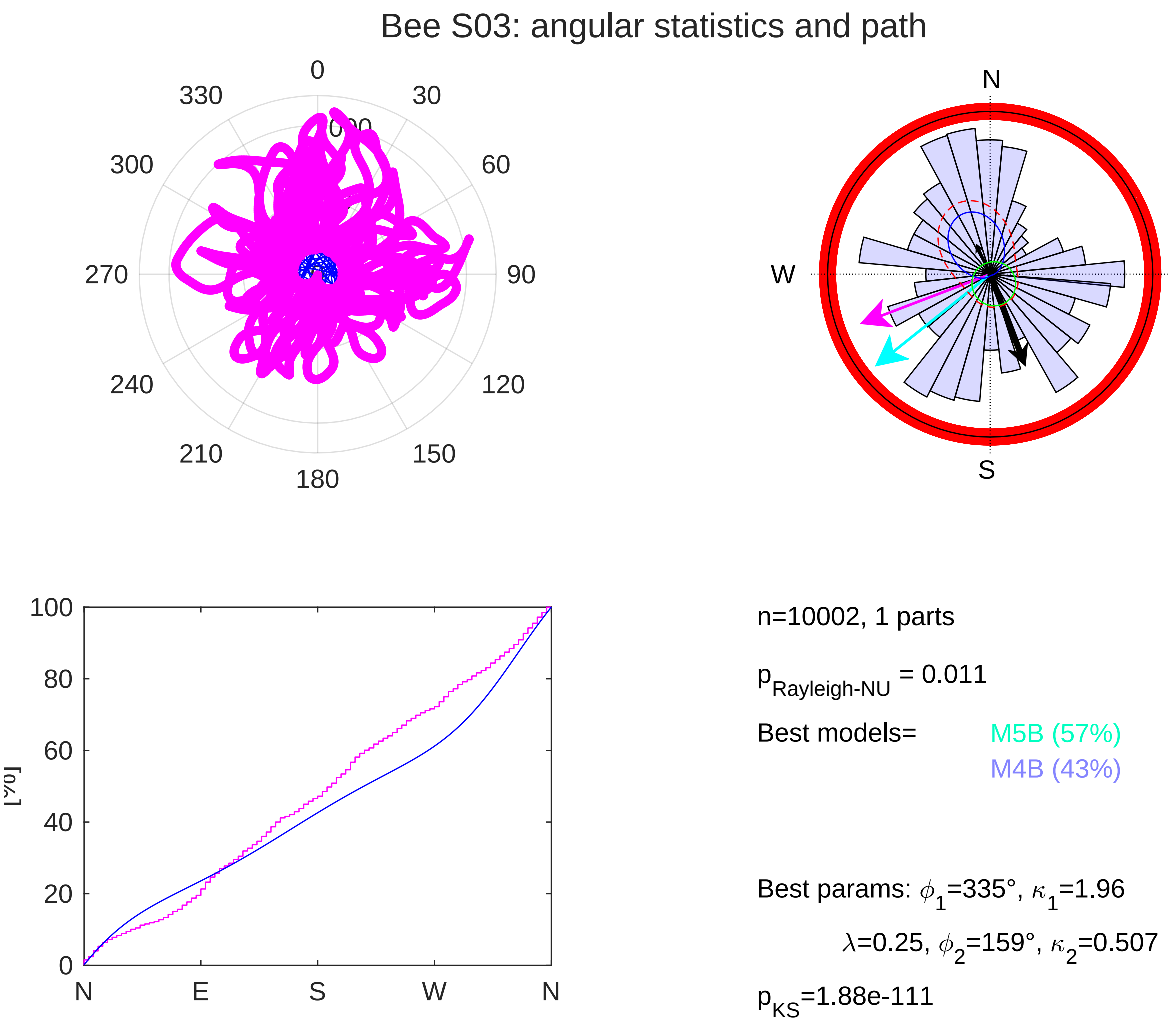
Bee S03: angular statistics and path
0
30
60
90
120
150
180
210
240
270
300
330
N
E
S
W
100
80
60
40
20
0
[%]
N
E
S
W
N
n=10002, 1 parts
$p_{Rayleigh\text{-}NU}$ = 0.011
Best models=
M5B (57%)
M4B (43%)
Best params: ϕ_1=335°, κ_1=1.96
λ=0.25, ϕ_2=159°, κ_2=0.507
p_{KS}=1.88e-111

Bee S04: angular statistics and path

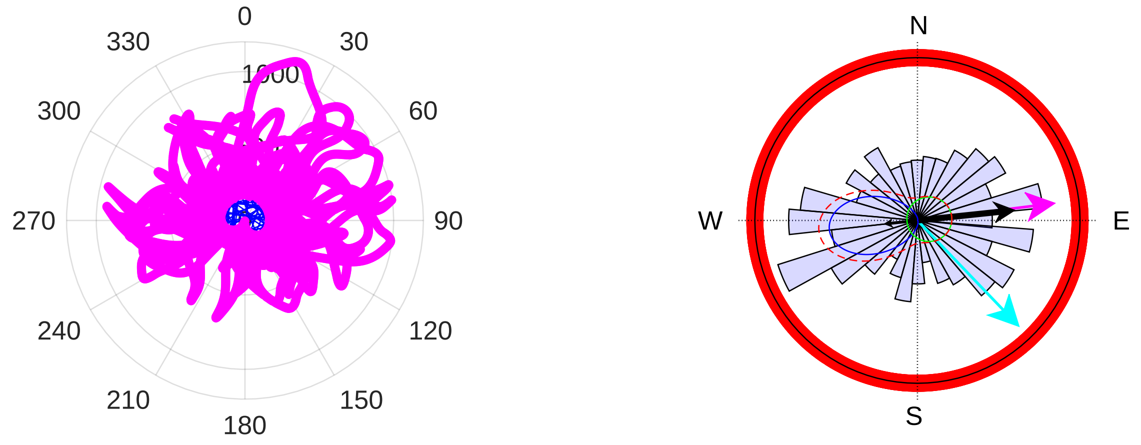

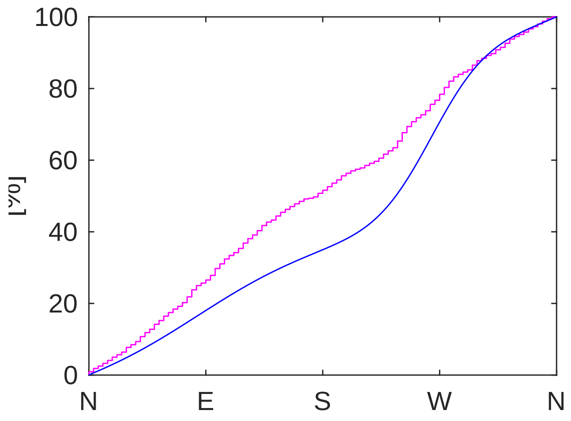

n=10002, 1 parts

$p_{Rayleigh-NU}$ = 0.32

Best models= M4B (100%)

Best params: ϕ_1=264°, κ_1=3.21

λ=0.25, ϕ_2=84°, κ_2=0.593

p_{KS}=1.41e-275

Bee S05: angular statistics and path

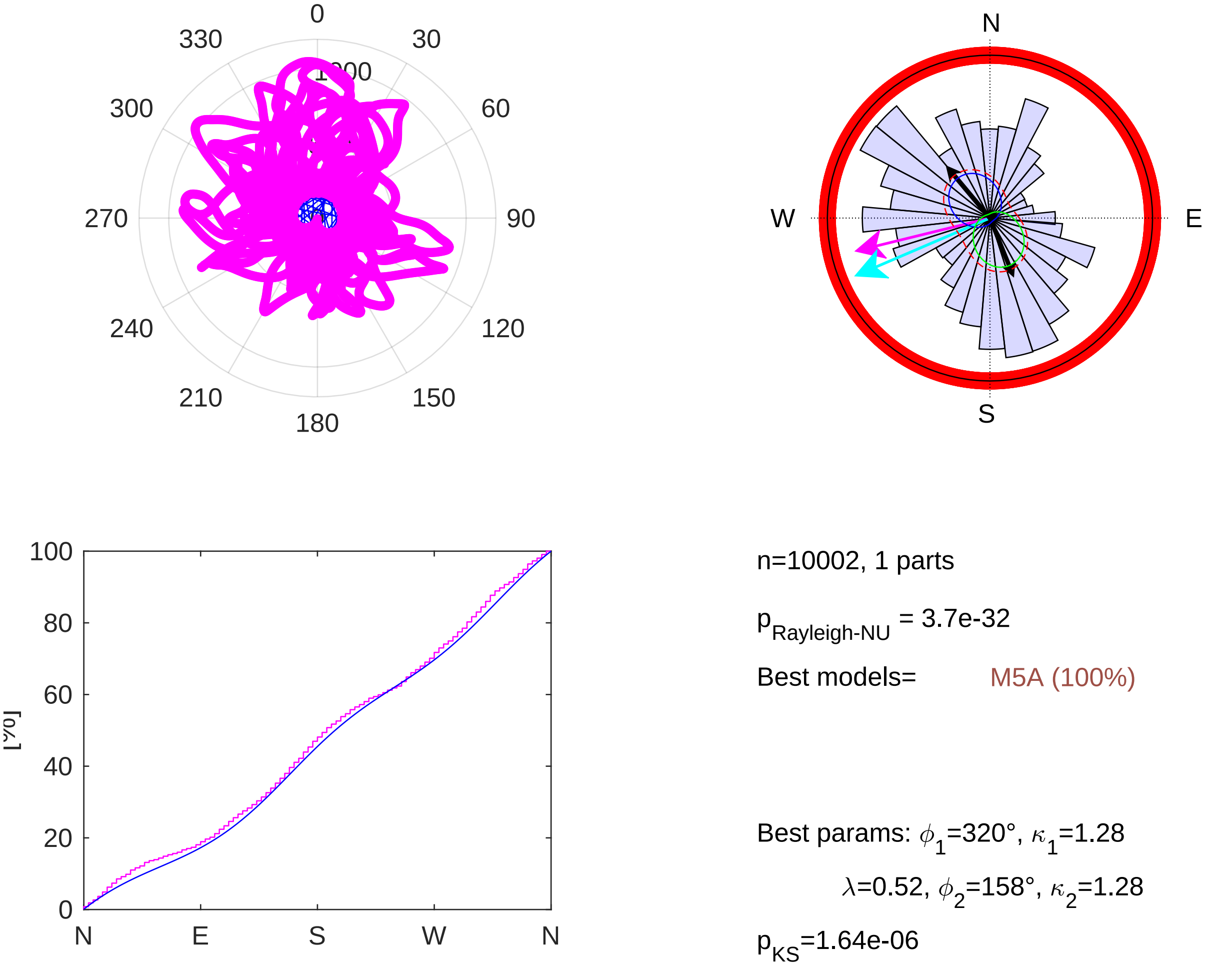

n=10002, 1 parts

$p_{Rayleigh\text{-}NU}$ = 3.7e-32

Best models= M5A (100%)

Best params: ϕ_1=320°, κ_1=1.28

λ=0.52, ϕ_2=158°, κ_2=1.28

p_{KS}=1.64e-06

Bee S06: angular statistics and path

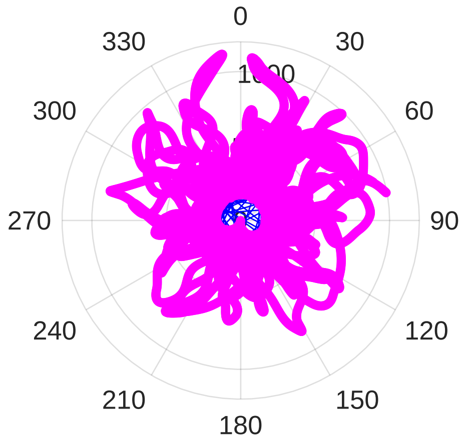

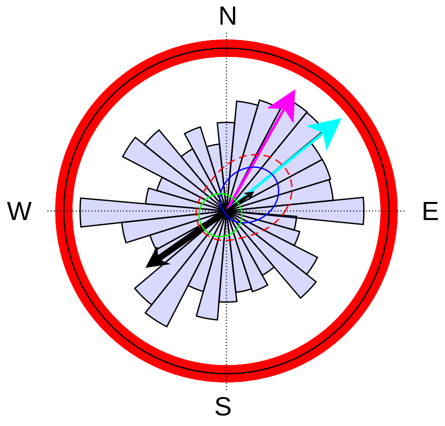

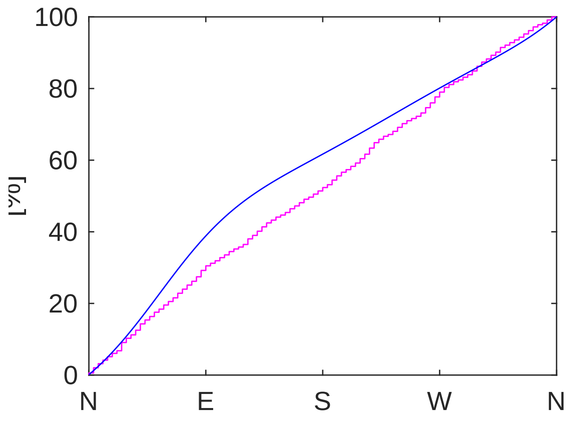

n=10002, 1 parts

$p_{Rayleigh\text{-}NU}$ = 0.0073

Best models= M4B (64%)
M5B (25%)

Best params: ϕ_1=55°, κ_1=1.6

λ=0.25, ϕ_2=235°, κ_2=0.363

p_{KS}=5.35e-146

Bee S07: angular statistics and path

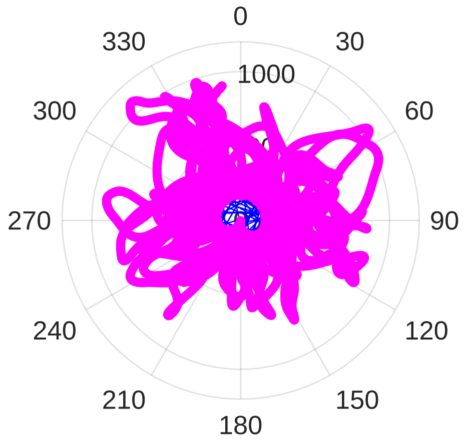

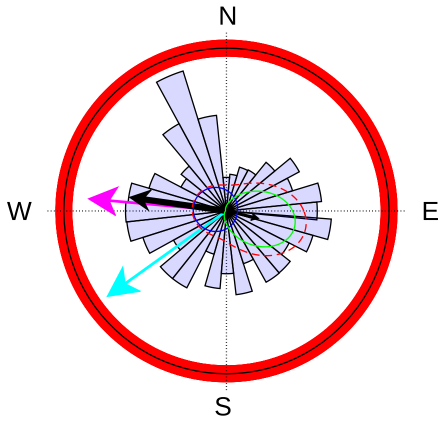

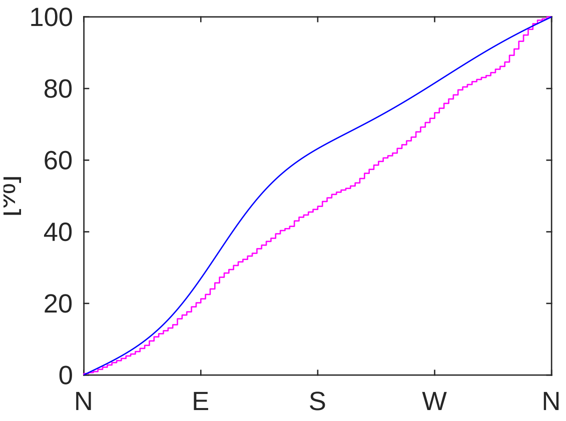

n=10002, 1 parts

$p_{Rayleigh\text{-}NU}$ = 0.0018

Best models= M5B (95%)

Best params: ϕ_1=278°, κ_1=0.55

λ=0.75, ϕ_2=103°, κ_2=2.14

p_{KS}=2.46e-267

Bee S08: angular statistics and path

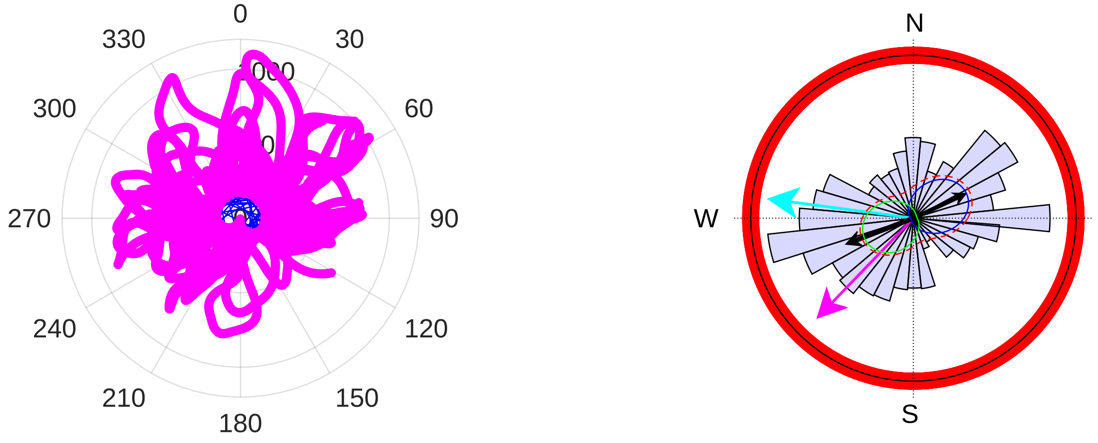

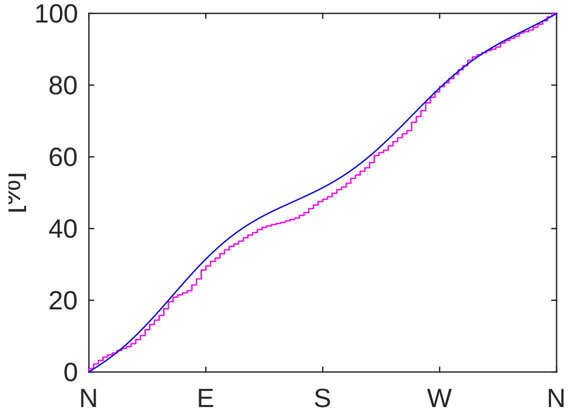

n=10002, 1 parts

$p_{Rayleigh-NU}$ = 1.2e-6

Best models= M5B (91%)

Best params: ϕ_1=65°, κ_1=1.59

λ=0.45, ϕ_2=249°, κ_2=1.35

p_{KS}=6.35e-26

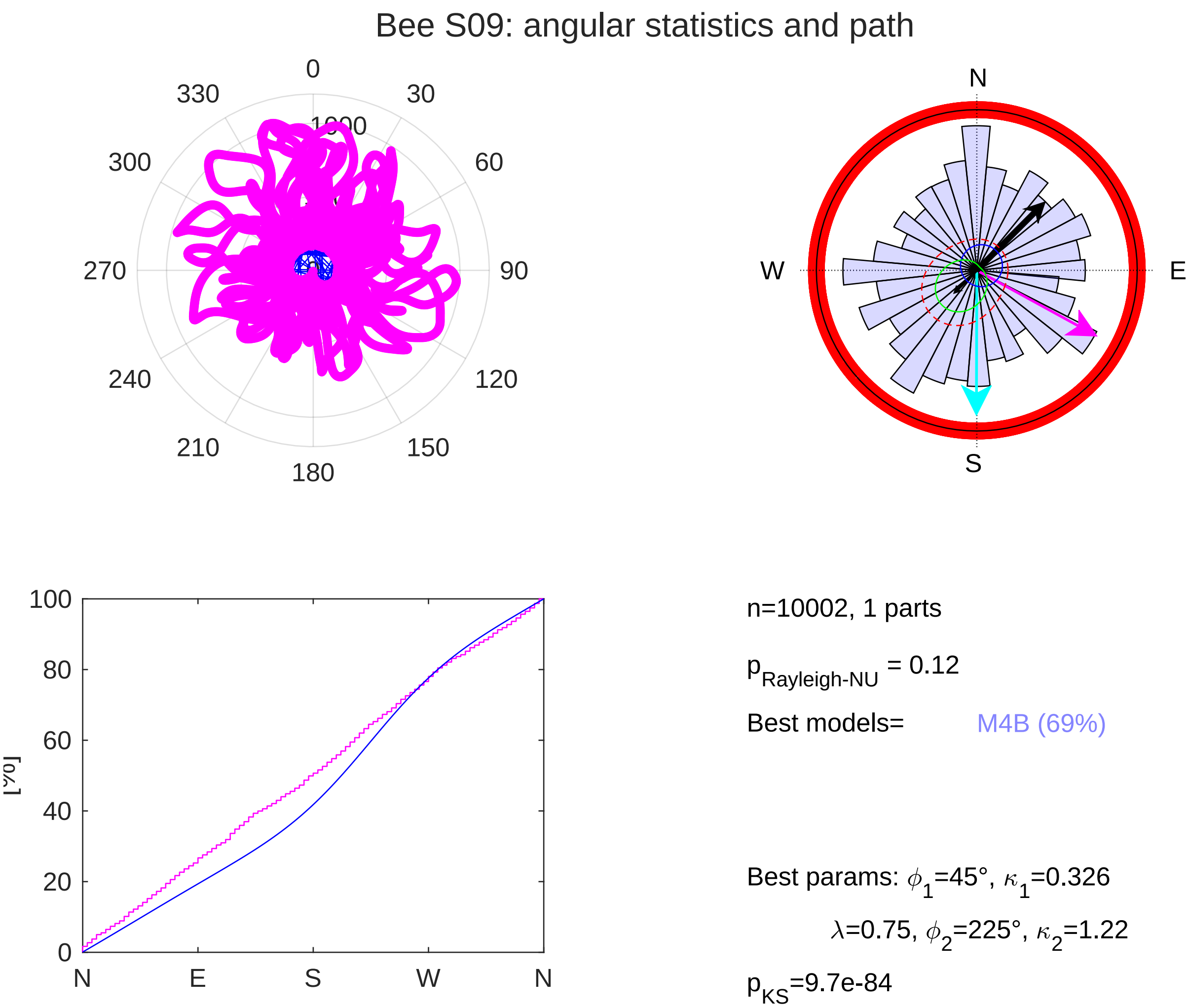
Bee S09: angular statistics and path
0
30
60
90
120
150
180
210
240
270
300
330
1000
N
E
S
W
100
80
60
40
20
0
[%]
N
E
S
W
N
n=10002, 1 parts
$p_{Rayleigh\text{-}NU}$ = 0.12
Best models=
M4B (69%)
Best params: ϕ_1=45°, κ_1=0.326
λ=0.75, ϕ_2=225°, κ_2=1.22
p_{KS}=9.7e-84

Bee S10: angular statistics and path

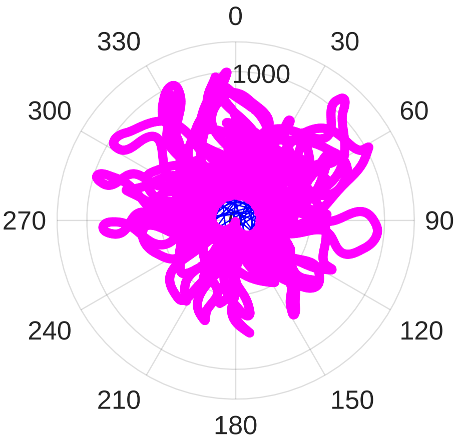

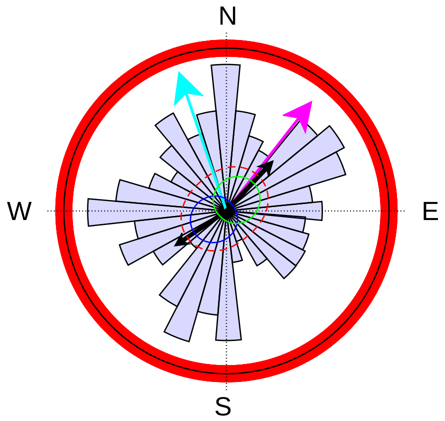

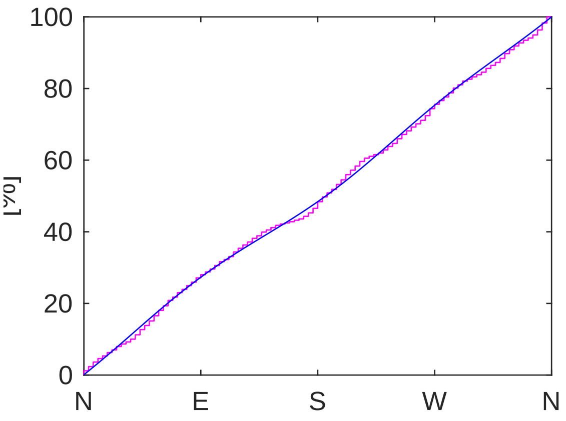

n=10002, 1 parts

$p_{Rayleigh-NU}$ = 4.1e-9

Best models= M5A (66%)
M5B (34%)

Best params: ϕ_1=236°, κ_1=0.775
λ=0.48, ϕ_2=43°, κ_2=0.775

p_{KS}=3.02e-05

Bee S11: angular statistics and path

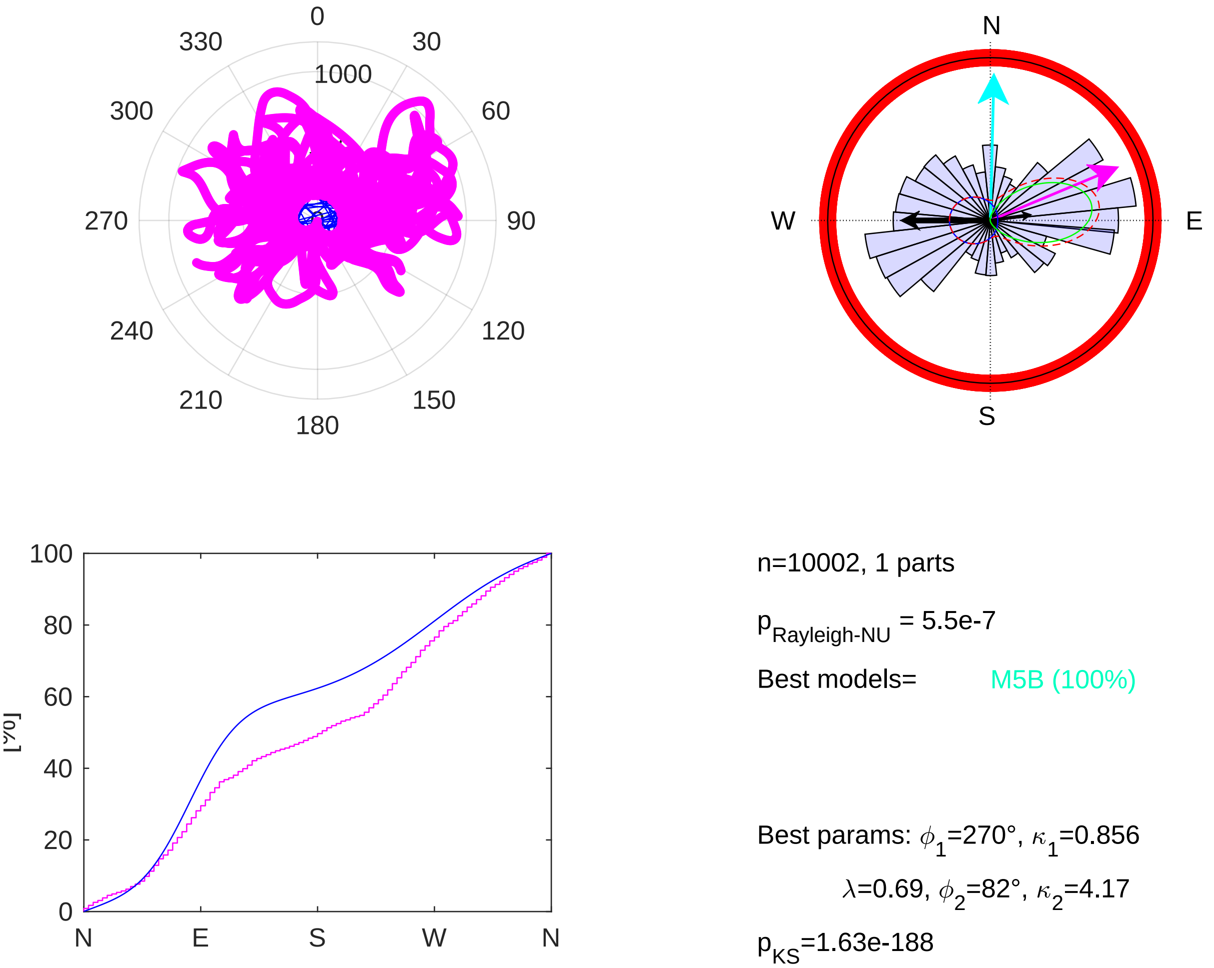

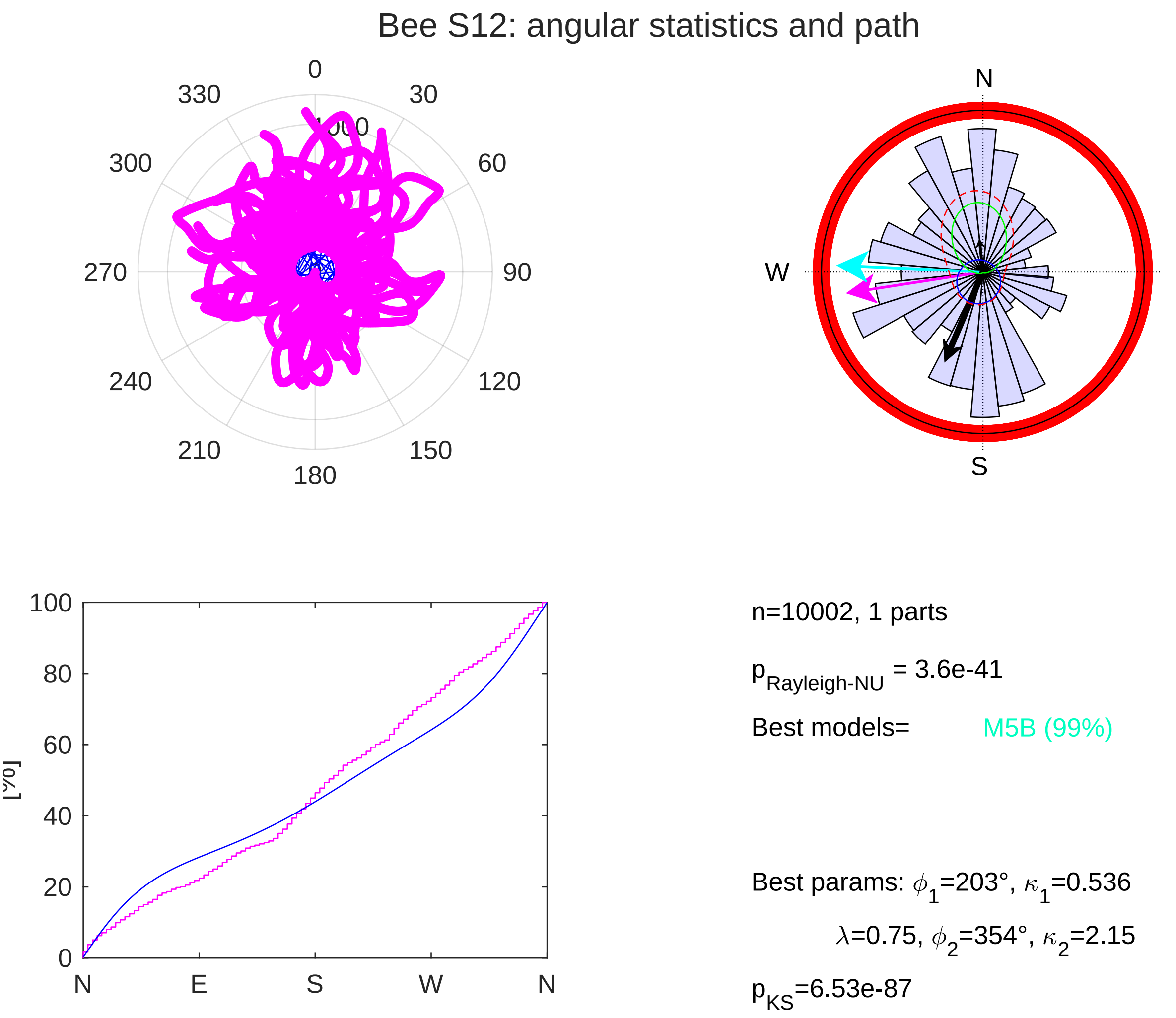
Bee S12: angular statistics and path
0
30
60
90
120
150
180
210
240
270
300
330
N
E
S
W
100
80
60
40
20
0
[%]
N E S W N
n=10002, 1 parts
$p_{Rayleigh-NU}$ = 3.6e-41
Best models= M5B (99%)
Best params: ϕ_1=203°, κ_1=0.536
λ=0.75, ϕ_2=354°, κ_2=2.15
p_{KS}=6.53e-87

Bee S13: angular statistics and path

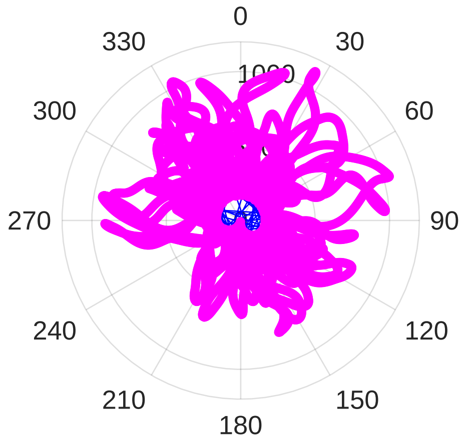

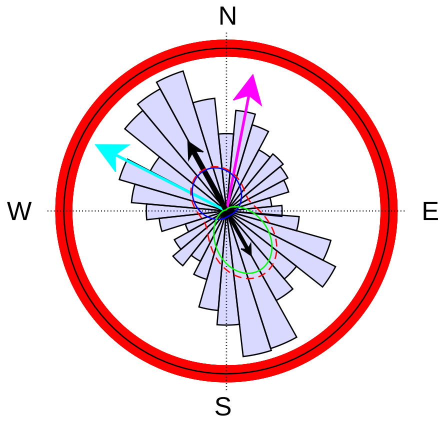

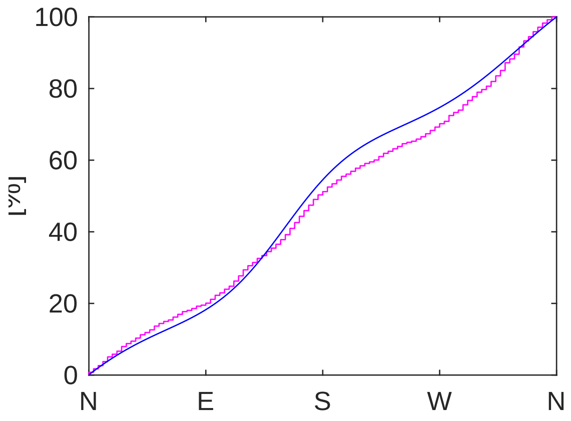

n=10002, 1 parts

$p_{Rayleigh\text{-}NU}$ = 0.22

Best models= M4B (59%)
M5B (40%)

Best params: ϕ_1=331°, κ_1=1.06
λ=0.61, ϕ_2=151°, κ_2=2.04

p_{KS}=3.57e-37

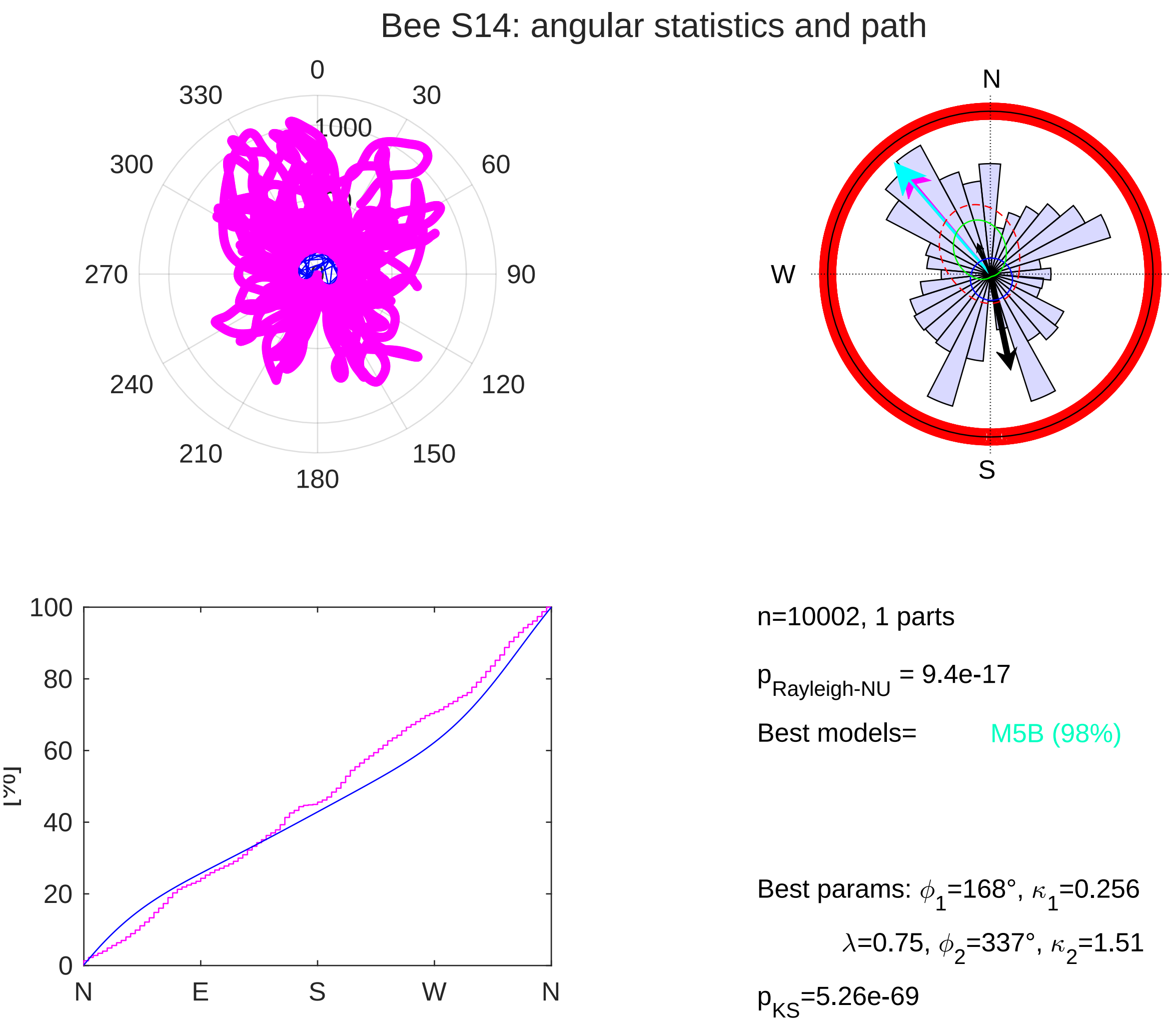
Bee S14: angular statistics and path
0
30
60
90
120
150
180
210
240
270
300
330
1000
N
E
S
W
100
80
60
40
20
0
[%]
N
E
S
W
N
n=10002, 1 parts
$p_{Rayleigh-NU}$ = 9.4e-17
Best models=
M5B (98%)
Best params: ϕ_1=168°, κ_1=0.256
λ=0.75, ϕ_2=337°, κ_2=1.51
p_{KS}=5.26e-69

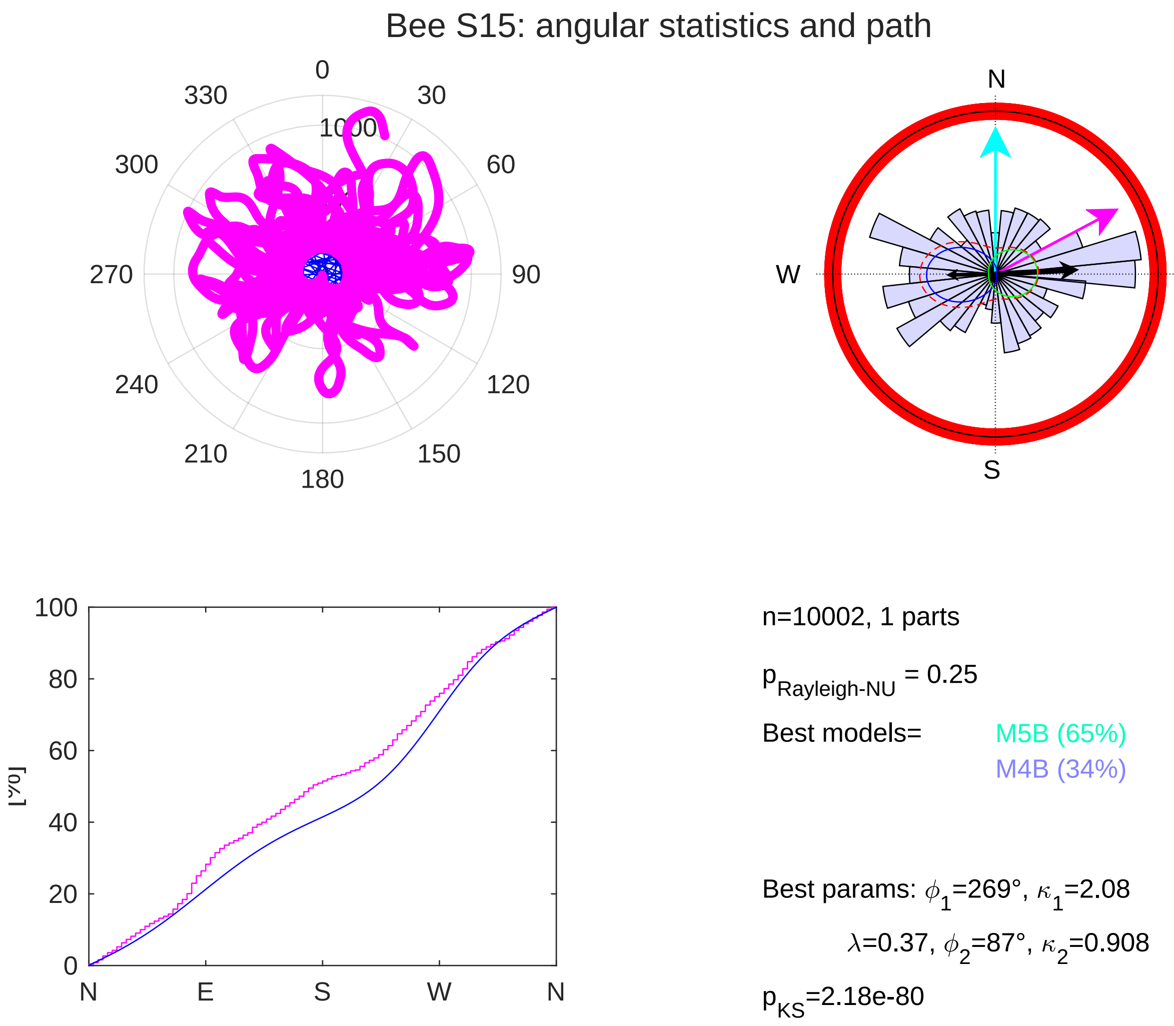
Bee S15: angular statistics and path
0
30
60
90
120
150
180
210
240
270
300
330
1000
N
E
S
W
100
80
60
40
20
0
[%]
N
E
S
W
N
n=10002, 1 parts
$p_{Rayleigh\text{-}NU}$ = 0.25
Best models=
M5B (65%)
M4B (34%)
Best params: ϕ_1=269°, κ_1=2.08
λ=0.37, ϕ_2=87°, κ_2=0.908
p_{KS}=2.18e-80

Bee S16: angular statistics and path

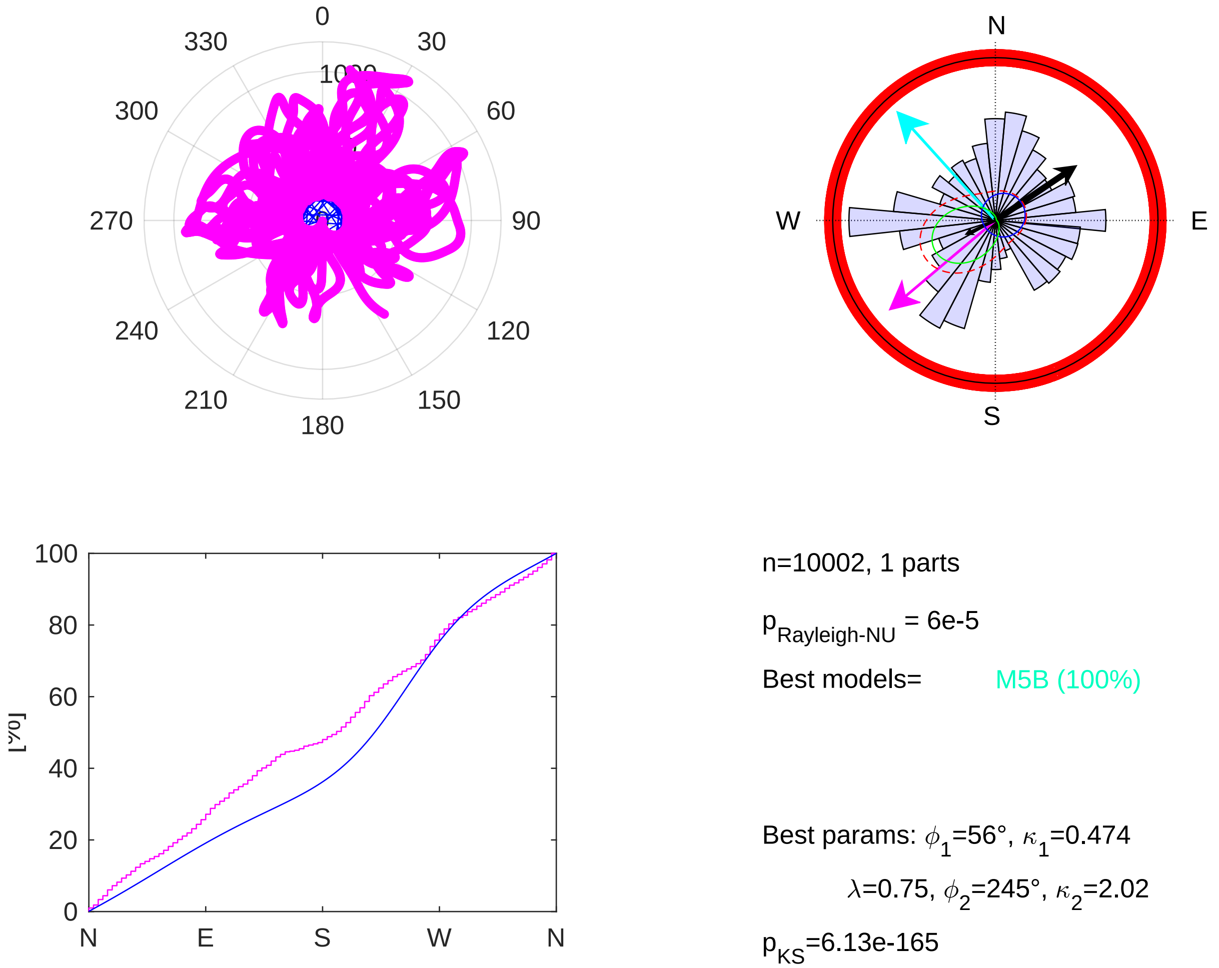

Supplement: Supplementary Data Sheet S3 — Circle statistics. [file Data_Sheet_3.pdf]
