# Supplementary material for: Generalization of navigation memory in honeybees
Source: Front Behav Neurosci. 2023 Mar 6;17:1070957. doi: 10.3389/fnbeh.2023.1070957 (PMC10025308; doi:10.3389/fnbeh.2023.1070957)
Supplement: Supplementary Data Sheet S4 — Heat maps. [file Data_Sheet_4.pdf]

Generalization of Navigation Memory in Honeybees

Supplement Data Sheet 04: Heat Maps

Eric Bullinger*
Otto-von-Guericke-Universität Magdeburg
Institut für Automatisierungstechnik
Universitätsplatz 2, 39106 Magdeburg, Germany
eric.bullinger@ovgu.de

Uwe Greggers & Randolf Menzel*
Freie Universität Berlin
Neurobiologie
Königin Luisestr. 1 -3, 14195 Berlin, Germany
menzel@neurobiologie.fu-berlin.de

14 February 2023

- Bee A01: p. 2
- Bee A02: p. 2
- Bee A03: p. 2
- Bee A04: p. 2
- Bee A05: p. 2
- Bee A06: p. 2
- Bee A07: p. 3
- Bee A08: p. 3
- Bee A09: p. 3
- Bee A10: p. 3
- Bee A11: p. 3
- Bee A12: p. 3
- Bee A13: p. 4
- Bee A14: p. 4
- Bee B01: p. 5
- Bee B02: p. 5
- Bee B03: p. 5
- Bee B04: p. 5
- Bee B05: p. 5
- Bee B06: p. 5
- Bee B07: p. 6
- Bee B08: p. 6
- Bee B09: p. 6
- Bee B10: p. 6
- Bee B11: p. 6
- Bee C01: p. 7
- Bee C02: p. 7
- Bee C03: p. 7
- Bee C04: p. 7
- Bee C05: p. 7
- Bee D01: p. 8
- Bee D02: p. 8
- Bee D03: p. 8
- Bee D04: p. 8
- Bee D05: p. 8
- Bee D06: p. 8
- Bee D07: p. 9
- Bee D08: p. 9
- Bee D09: p. 9
- Bee D10: p. 9
- Bee D11: p. 9
- Bee D12: p. 9
- Bee D13: p. 10
- Bee E01: p. 11
- Bee E02: p. 11
- Bee E03: p. 11
- Bee E04: p. 11
- Bee E05: p. 11
- Bee E06: p. 11
- Bee E07: p. 12
- Bee R01: p. 13
- Bee R02: p. 13
- Bee R03: p. 13
- Bee R04: p. 13
- Bee R05: p. 13
- Bee R06: p. 13
- Bee R07: p. 14
- Bee R08: p. 14
- Bee R09: p. 14
- Bee R10: p. 14
- Bee R11: p. 14
- Bee R12: p. 14
- Bee R13: p. 15
- Bee R14: p. 15
- Bee R15: p. 15
- Bee R16: p. 15
- Bee S01: p. 16
- Bee S02: p. 16
- Bee S03: p. 16
- Bee S04: p. 16
- Bee S05: p. 16
- Bee S06: p. 16
- Bee S07: p. 17
- Bee S08: p. 17
- Bee S09: p. 17
- Bee S10: p. 17
- Bee S11: p. 17
- Bee S12: p. 17
- Bee S13: p. 18
- Bee S14: p. 18
- Bee S15: p. 18
- Bee S16: p. 18

*corresponding author

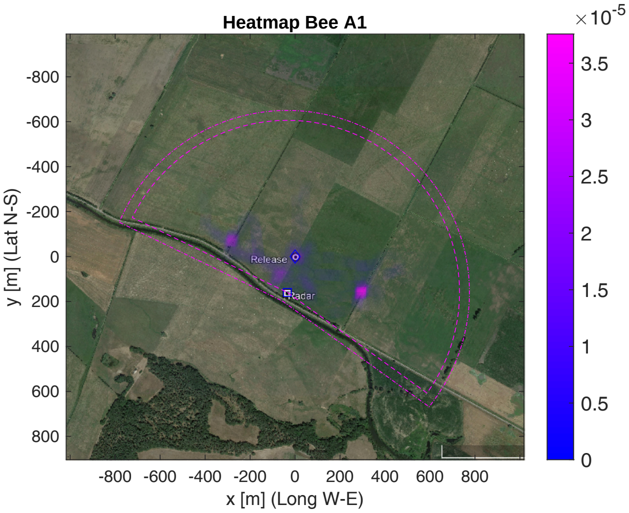
Heatmap Bee A1
×10-5
0
0.5
1
1.5
2
2.5
3
3.5
Release
Radar
y [m] (Lat N-S)
x [m] (Long W-E)
-800
-600
-400
-200
0
200
400
600
800

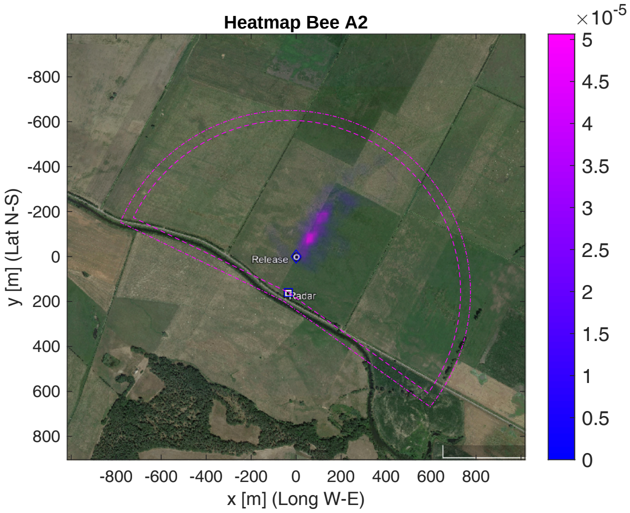
Heatmap Bee A2
×10-5
0
0.5
1
1.5
2
2.5
3
3.5
4
4.5
5
Release
Radar
y [m] (Lat N-S)
x [m] (Long W-E)
-800
-600
-400
-200
0
200
400
600
800

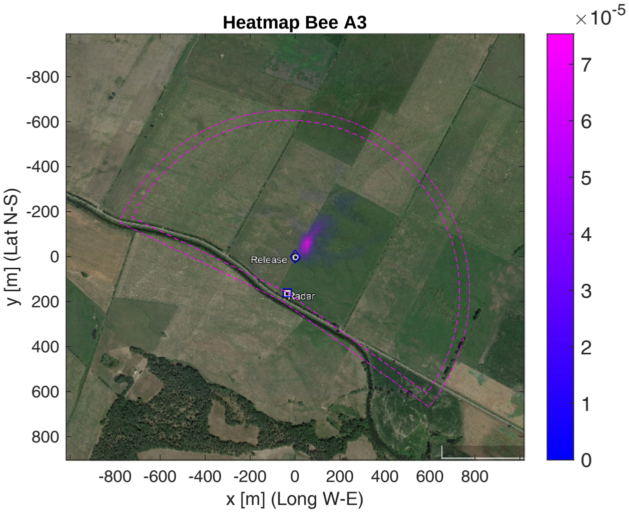
Heatmap Bee A3
×10-5
0
1
2
3
4
5
6
7
Release
Radar
y [m] (Lat N-S)
x [m] (Long W-E)
-800
-600
-400
-200
0
200
400
600
800

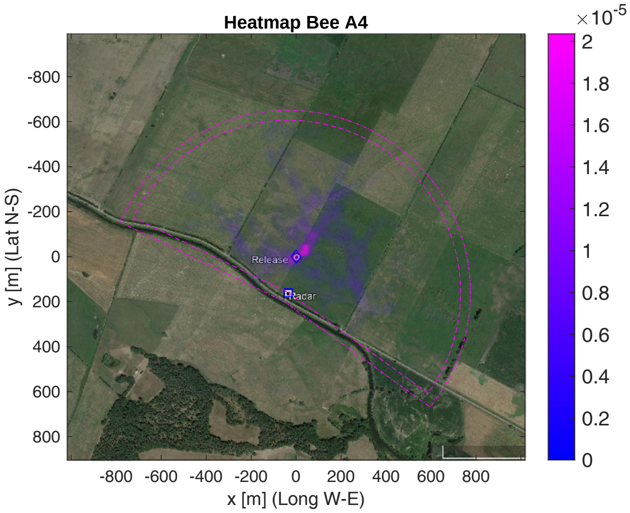
Heatmap Bee A4
×10-5
0
0.2
0.4
0.6
0.8
1
1.2
1.4
1.6
1.8
2
Release
Radar
y [m] (Lat N-S)
x [m] (Long W-E)
-800
-600
-400
-200
0
200
400
600
800

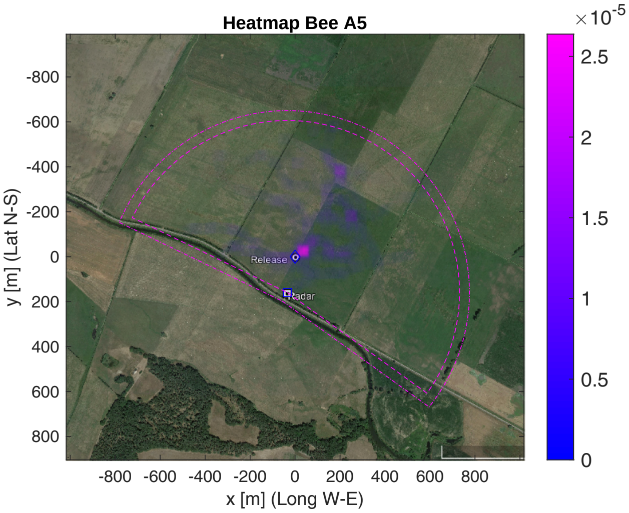
Heatmap Bee A5
×10-5
0
0.5
1
1.5
2
2.5
Release
Radar
y [m] (Lat N-S)
x [m] (Long W-E)
-800
-600
-400
-200
0
200
400
600
800

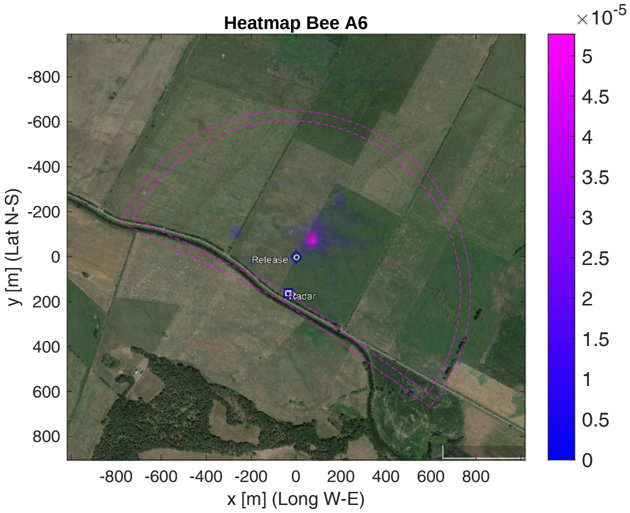
Heatmap Bee A6
×10-5
0
0.5
1
1.5
2
2.5
3
3.5
4
4.5
5
Release
Radar
y [m] (Lat N-S)
x [m] (Long W-E)
-800
-600
-400
-200
0
200
400
600
800

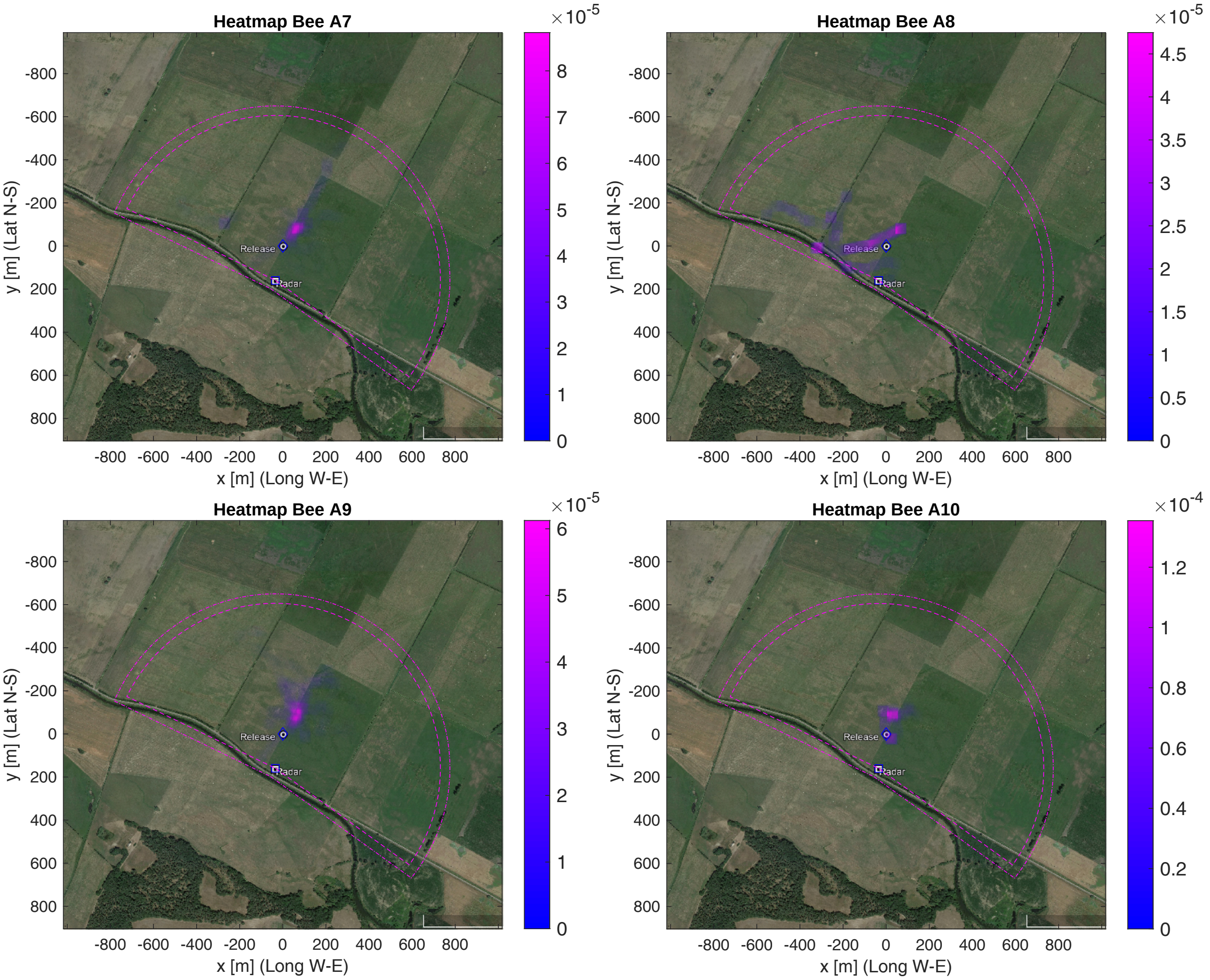
Heatmap Bee A7
×10-5
y [m] (Lat N-S)
x [m] (Long W-E)
Release
Radar
Heatmap Bee A8
×10-5
y [m] (Lat N-S)
x [m] (Long W-E)
Release
Radar
Heatmap Bee A9
×10-5
y [m] (Lat N-S)
x [m] (Long W-E)
Release
Radar
Heatmap Bee A10
×10-4
y [m] (Lat N-S)
x [m] (Long W-E)
Release
Radar

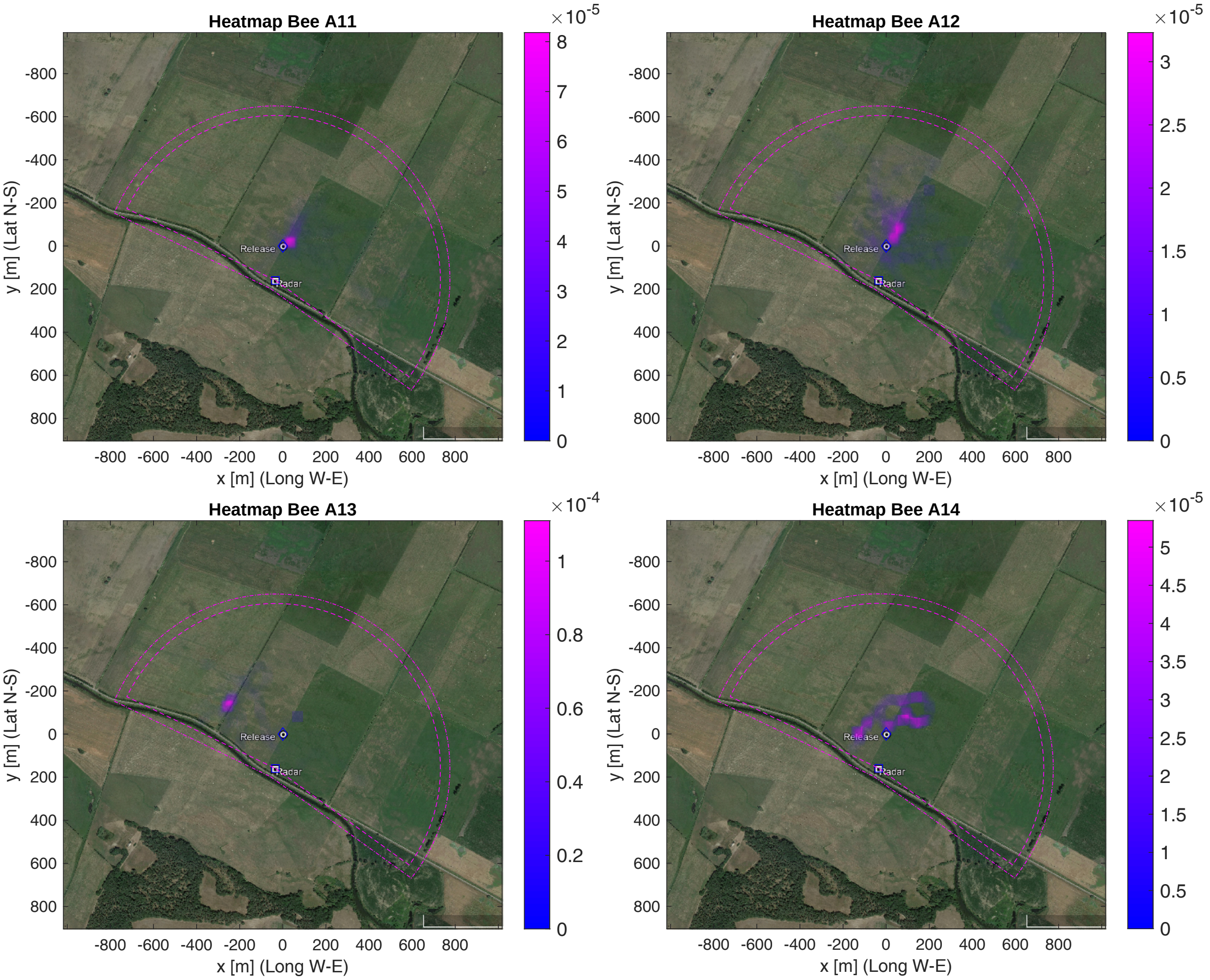
Heatmap Bee A11
×10-5
Heatmap Bee A12
×10-5
Heatmap Bee A13
×10-4
Heatmap Bee A14
×10-5
Release
Radar
y [m] (Lat N-S)
x [m] (Long W-E)

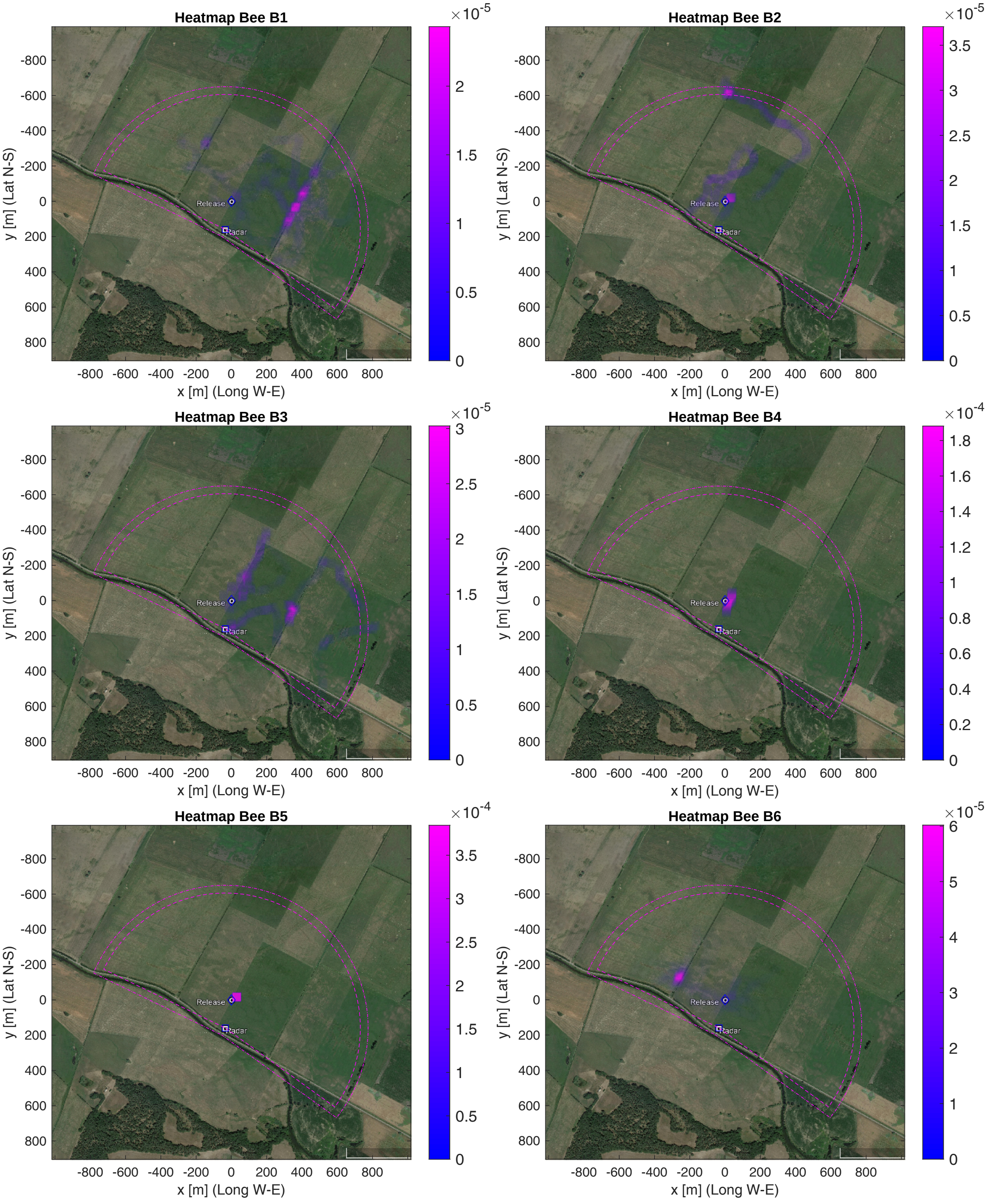
Heatmap Bee B1
×10^-5
y [m] (Lat N-S)
x [m] (Long W-E)
Release
Radar
Heatmap Bee B2
×10^-5
Heatmap Bee B3
×10^-5
Heatmap Bee B4
×10^-4
Heatmap Bee B5
×10^-4
Heatmap Bee B6
×10^-5

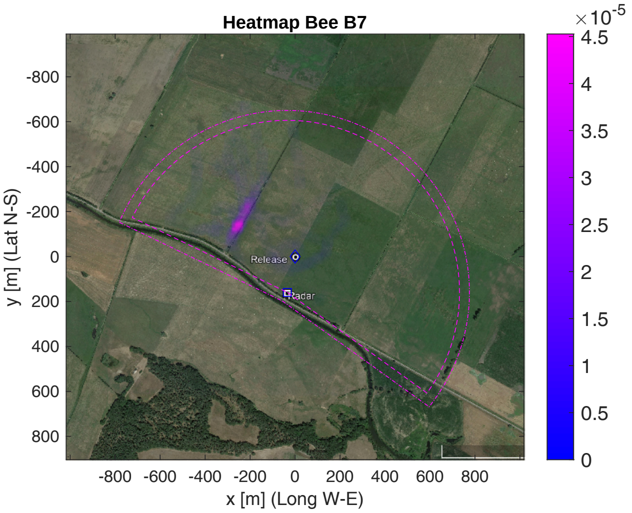
Heatmap Bee B7
×10-5
4.5
4
3.5
3
2.5
2
1.5
1
0.5
0
Release
Radar
y [m] (Lat N-S)
-800 -600 -400 -200 0 200 400 600 800
x [m] (Long W-E)
-800 -600 -400 -200 0 200 400 600 800

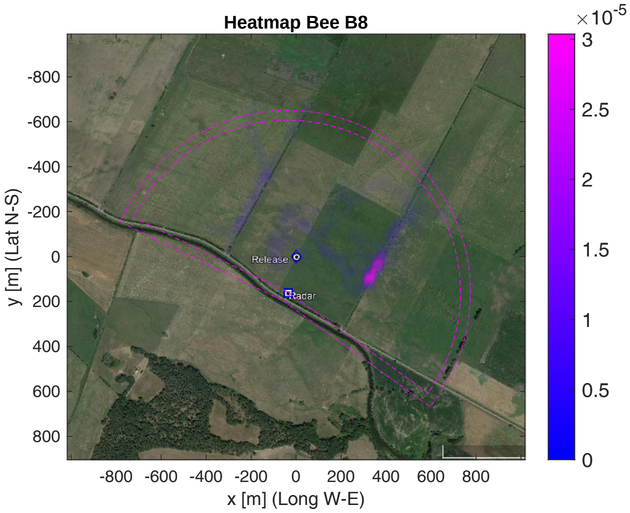
Heatmap Bee B8
×10-5
3
2.5
2
1.5
1
0.5
0
Release
Radar
y [m] (Lat N-S)
-800 -600 -400 -200 0 200 400 600 800
x [m] (Long W-E)
-800 -600 -400 -200 0 200 400 600 800

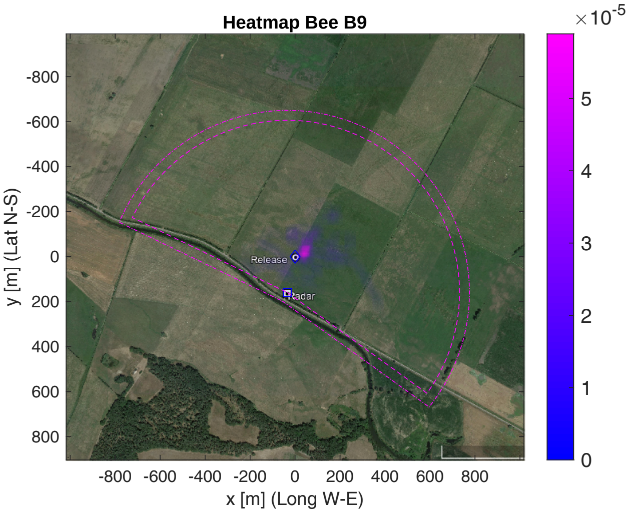
Heatmap Bee B9
×10-5
5
4
3
2
1
0
Release
Radar
y [m] (Lat N-S)
-800 -600 -400 -200 0 200 400 600 800
x [m] (Long W-E)
-800 -600 -400 -200 0 200 400 600 800

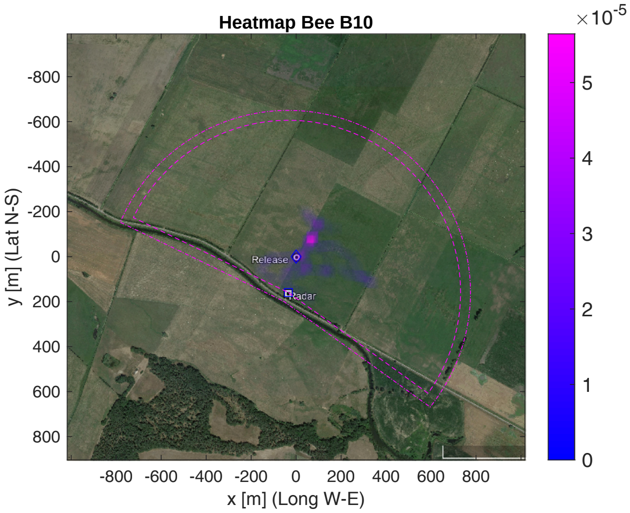
Heatmap Bee B10
×10-5
5
4
3
2
1
0
Release
Radar
y [m] (Lat N-S)
-800 -600 -400 -200 0 200 400 600 800
x [m] (Long W-E)
-800 -600 -400 -200 0 200 400 600 800

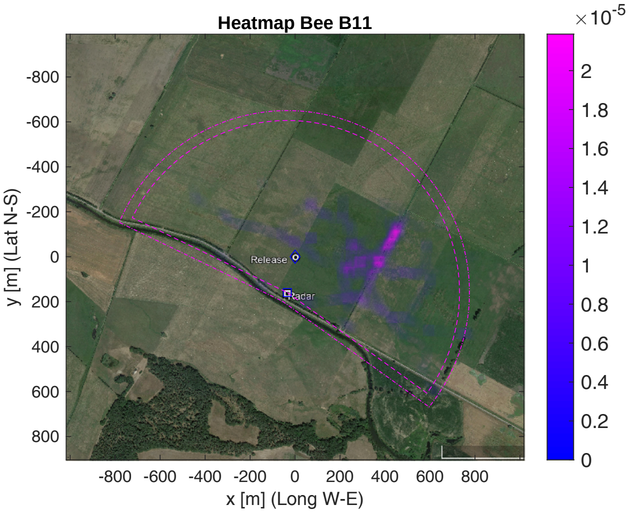
Heatmap Bee B11
×10-5
2
1.8
1.6
1.4
1.2
1
0.8
0.6
0.4
0.2
0
Release
Radar
y [m] (Lat N-S)
-800 -600 -400 -200 0 200 400 600 800
x [m] (Long W-E)
-800 -600 -400 -200 0 200 400 600 800

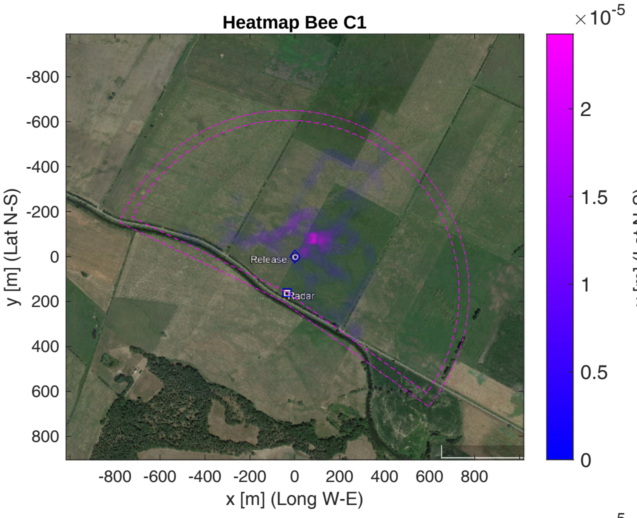
Heatmap Bee C1
×10-5
2
1.5
1
0.5
0
-800
-600
-400
-200
0
200
400
600
800
y [m] (Lat N-S)
Release
Radar
-800 -600 -400 -200 0 200 400 600 800
x [m] (Long W-E)

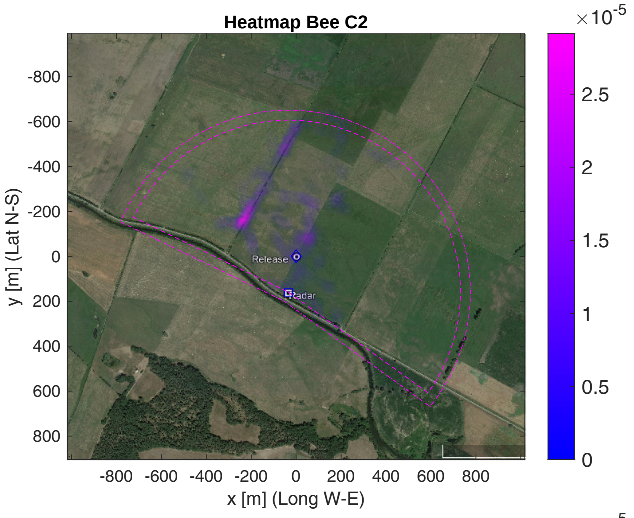
Heatmap Bee C2
×10-5
2.5
2
1.5
1
0.5
0
-800
-600
-400
-200
0
200
400
600
800
y [m] (Lat N-S)
Release
Radar
-800 -600 -400 -200 0 200 400 600 800
x [m] (Long W-E)

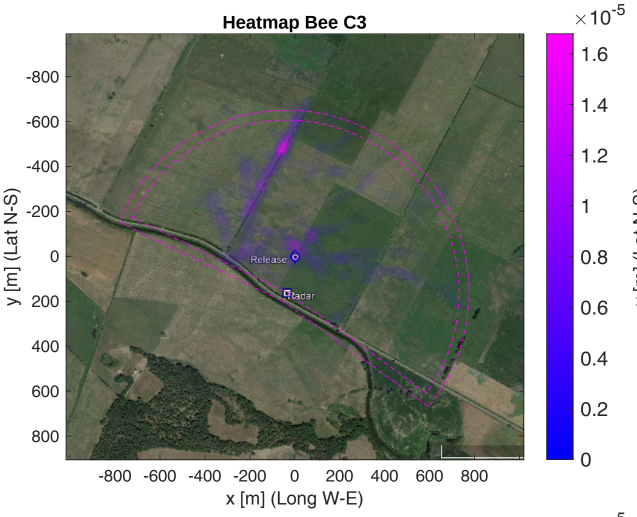
Heatmap Bee C3
×10-5
1.6
1.4
1.2
1
0.8
0.6
0.4
0.2
0
-800
-600
-400
-200
0
200
400
600
800
y [m] (Lat N-S)
Release
Radar
-800 -600 -400 -200 0 200 400 600 800
x [m] (Long W-E)

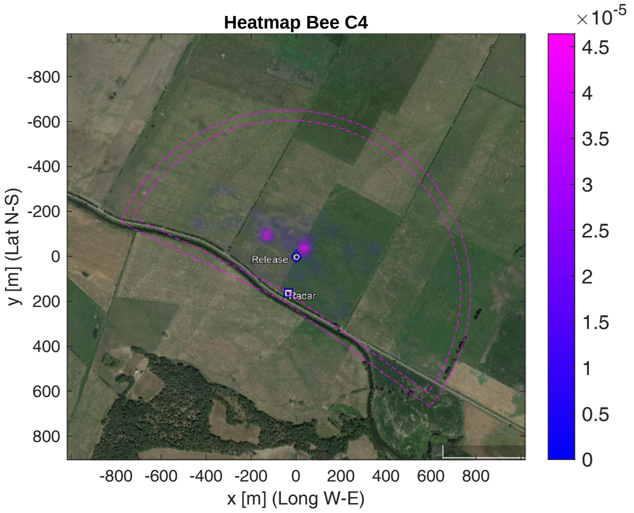
Heatmap Bee C4
×10-5
4.5
4
3.5
3
2.5
2
1.5
1
0.5
0
-800
-600
-400
-200
0
200
400
600
800
y [m] (Lat N-S)
Release
Radar
-800 -600 -400 -200 0 200 400 600 800
x [m] (Long W-E)

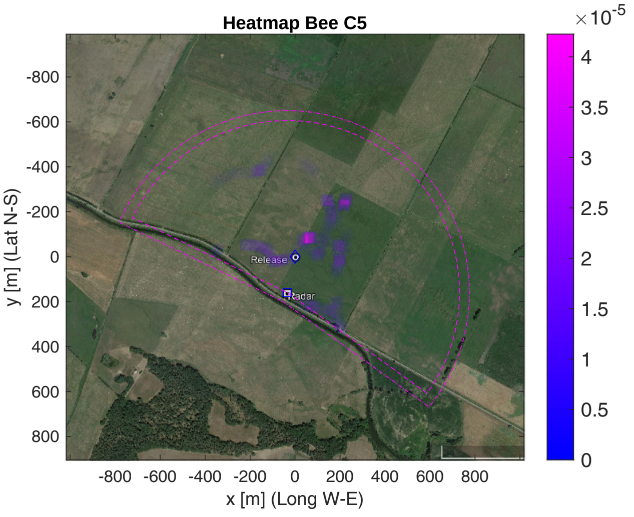
Heatmap Bee C5
×10-5
4
3.5
3
2.5
2
1.5
1
0.5
0
-800
-600
-400
-200
0
200
400
600
800
y [m] (Lat N-S)
Release
Radar
-800 -600 -400 -200 0 200 400 600 800
x [m] (Long W-E)

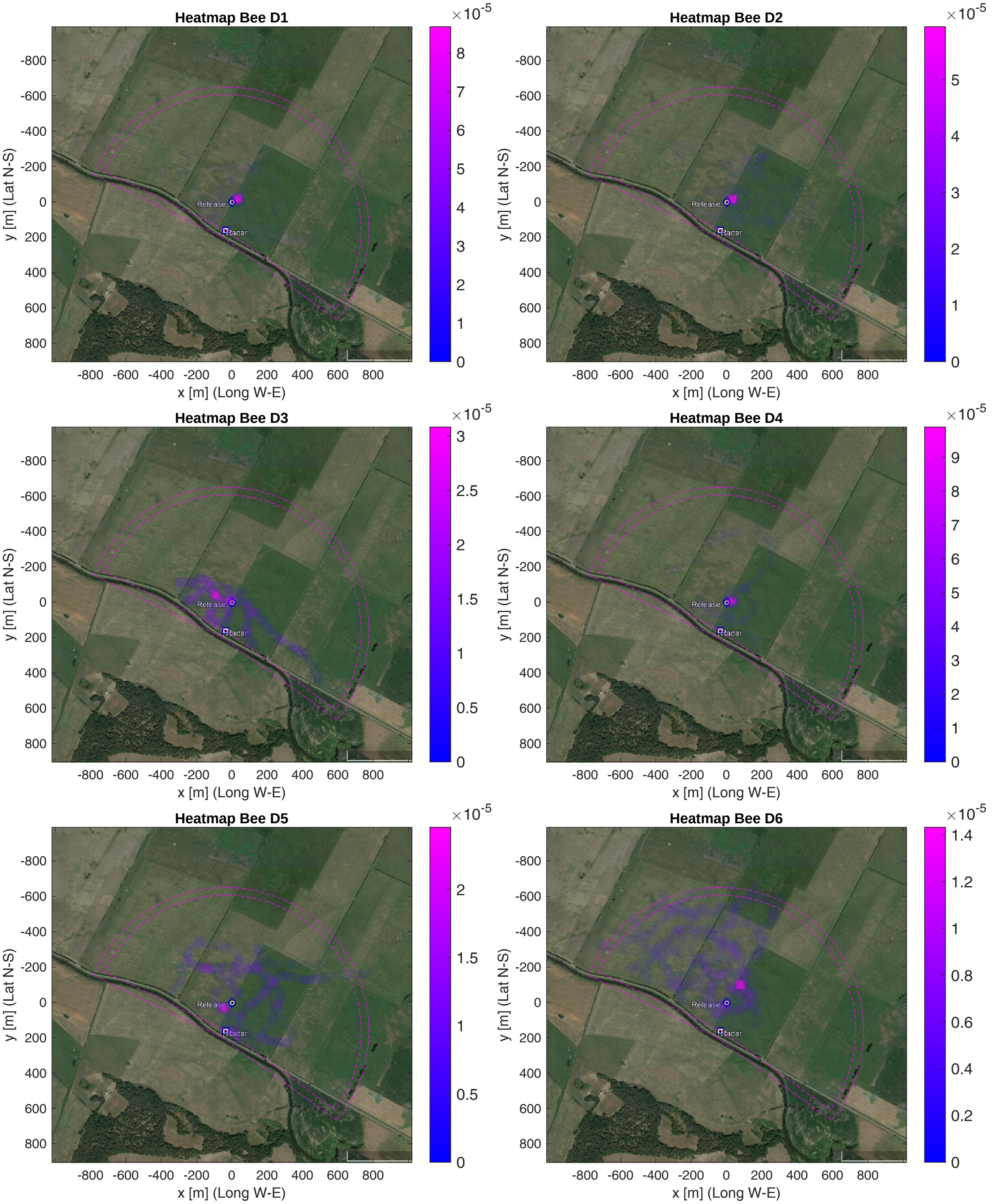
Heatmap Bee D1
×10^-5
Heatmap Bee D2
Heatmap Bee D3
Heatmap Bee D4
Heatmap Bee D5
Heatmap Bee D6
Release
Radar
y [m] (Lat N-S)
x [m] (Long W-E)
-800 -600 -400 -200 0 200 400 600 800

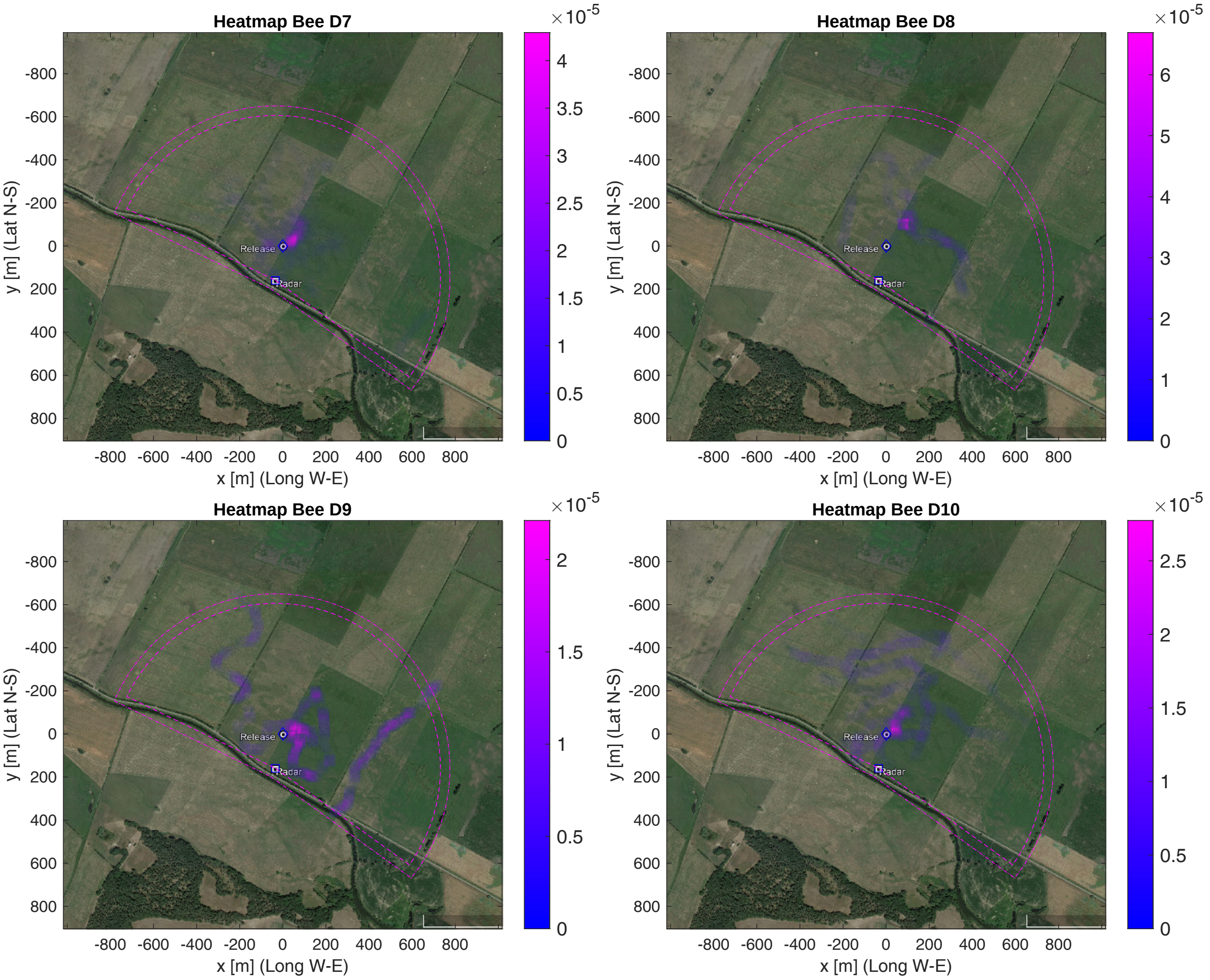
Heatmap Bee D7
Heatmap Bee D8
Heatmap Bee D9
Heatmap Bee D10
Release
Radar
y [m] (Lat N-S)
x [m] (Long W-E)
-800 -600 -400 -200 0 200 400 600 800
$\times 10^{-5}$

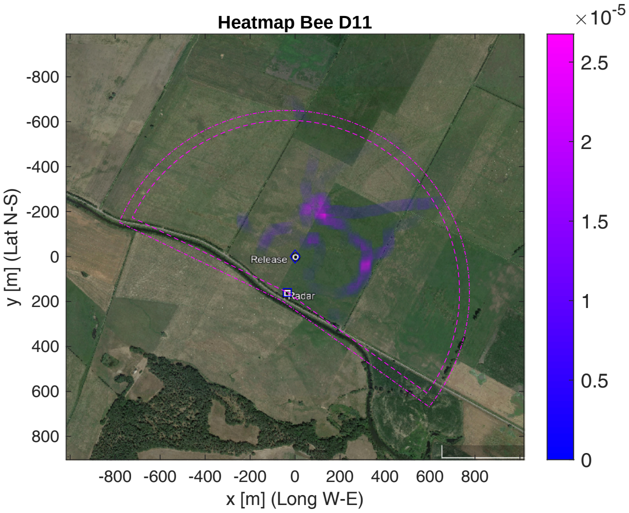
Heatmap Bee D11
×10-5
2.5
2
1.5
1
0.5
0
Release
Radar
-800 -600 -400 -200 0 200 400 600 800
y [m] (Lat N-S)
-800 -600 -400 -200 0 200 400 600 800
x [m] (Long W-E)

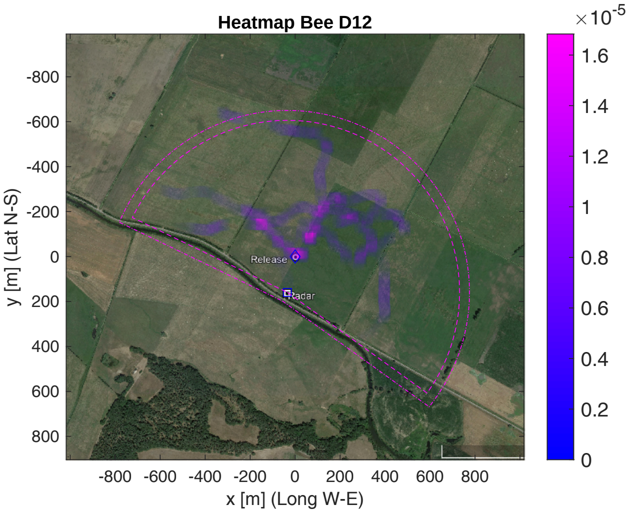
Heatmap Bee D12
×10-5
1.6
1.4
1.2
1
0.8
0.6
0.4
0.2
0
Release
Radar
-800 -600 -400 -200 0 200 400 600 800
y [m] (Lat N-S)
-800 -600 -400 -200 0 200 400 600 800
x [m] (Long W-E)

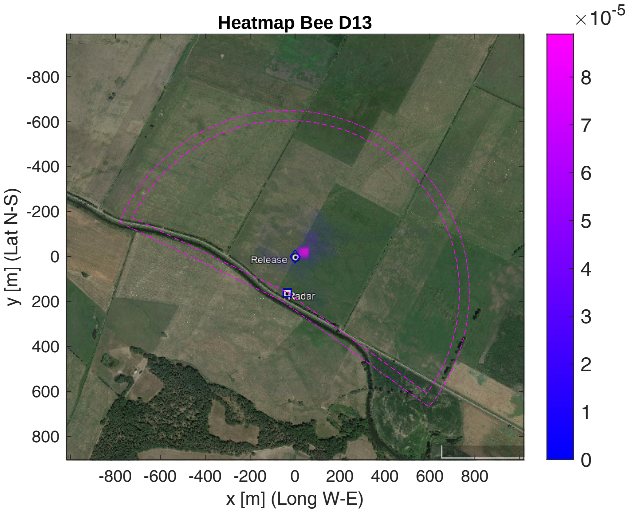
Heatmap Bee D13
×10-5
8
7
6
5
4
3
2
1
0
Release
Radar
-800 -600 -400 -200 0 200 400 600 800
y [m] (Lat N-S)
-800 -600 -400 -200 0 200 400 600 800
x [m] (Long W-E)

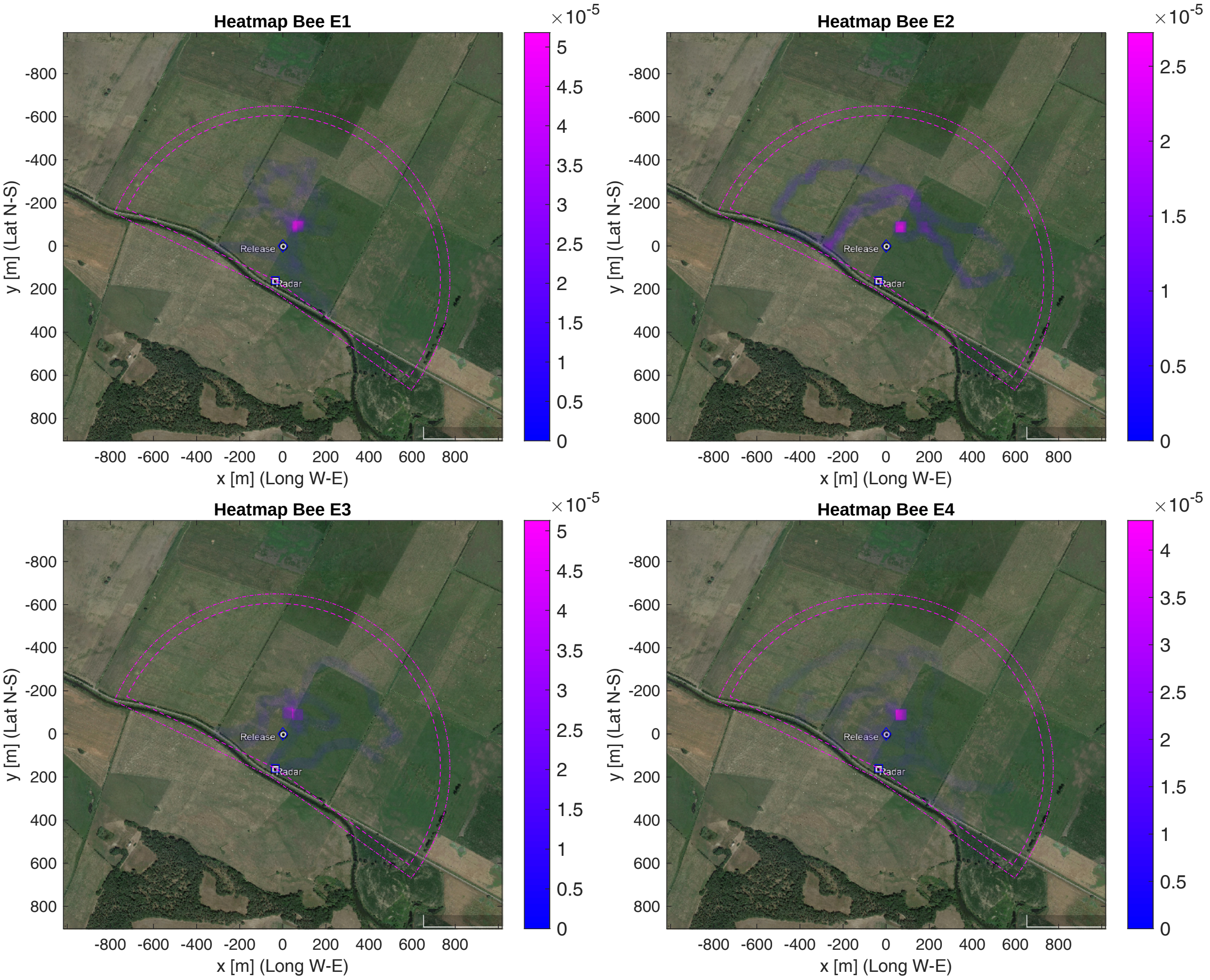
Heatmap Bee E1
Heatmap Bee E2
Heatmap Bee E3
Heatmap Bee E4
y [m] (Lat N-S)
x [m] (Long W-E)
-800 -600 -400 -200 0 200 400 600 800
Release
Radar
×10^-5
0 0.5 1 1.5 2 2.5 3 3.5 4 4.5 5

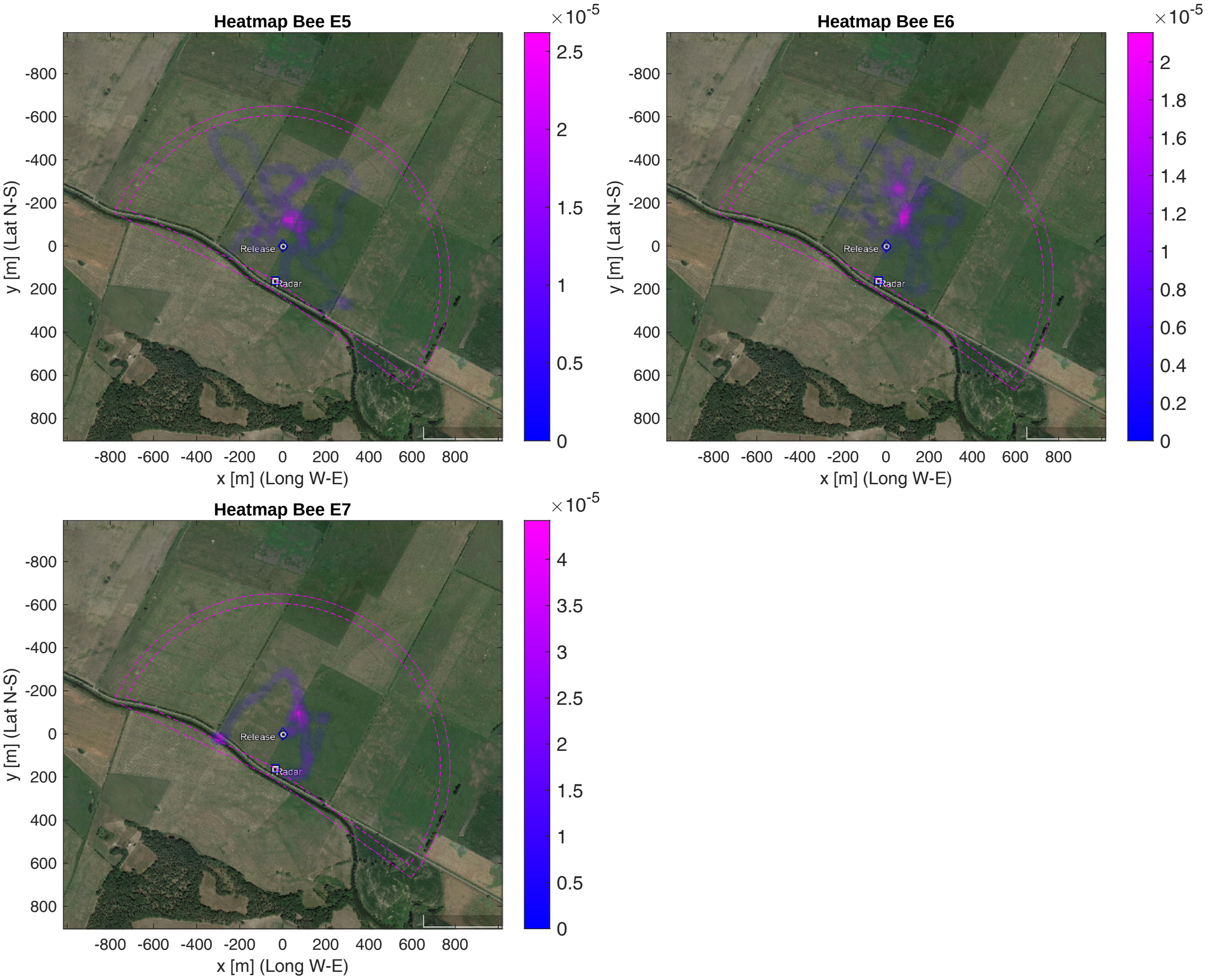
Heatmap Bee E5
×10-5
2.5
2
1.5
1
0.5
0
Release
Radar
y [m] (Lat N-S)
x [m] (Long W-E)
-800 -600 -400 -200 0 200 400 600 800
Heatmap Bee E6
×10-5
2
1.8
1.6
1.4
1.2
1
0.8
0.6
0.4
0.2
0
Release
Radar
y [m] (Lat N-S)
x [m] (Long W-E)
Heatmap Bee E7
×10-5
4
3.5
3
2.5
2
1.5
1
0.5
0
Release
Radar
y [m] (Lat N-S)
x [m] (Long W-E)

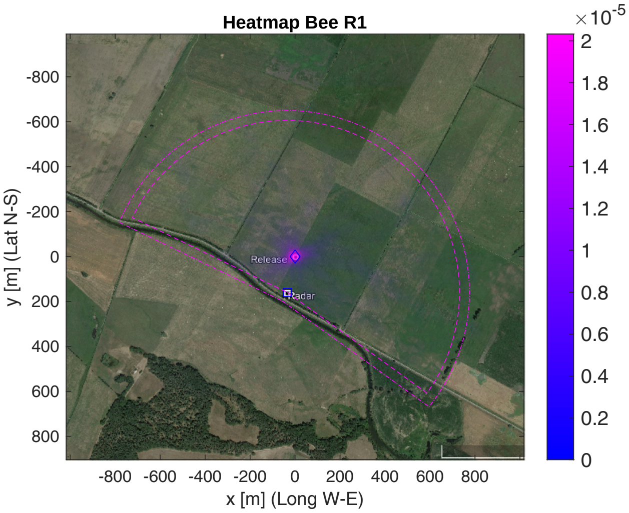
Heatmap Bee R1
×10^-5
2
1.8
1.6
1.4
1.2
1
0.8
0.6
0.4
0.2
0
-800
-600
-400
-200
0
200
400
600
800
y [m] (Lat N-S)
x [m] (Long W-E)
Release
Radar

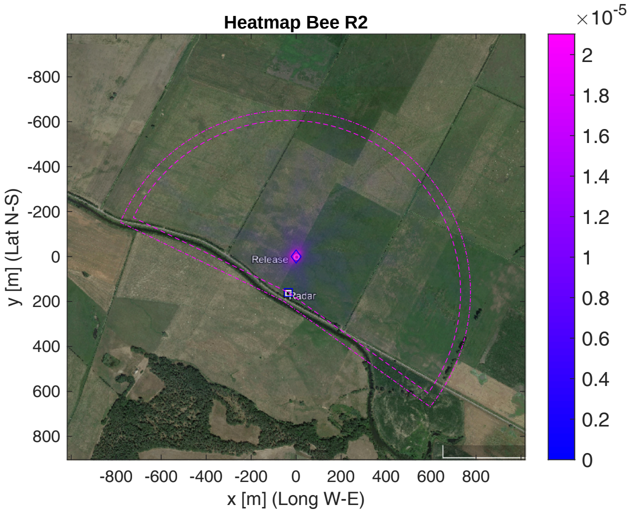
Heatmap Bee R2
×10^-5
2
1.8
1.6
1.4
1.2
1
0.8
0.6
0.4
0.2
0
-800
-600
-400
-200
0
200
400
600
800
y [m] (Lat N-S)
x [m] (Long W-E)
Release
Radar

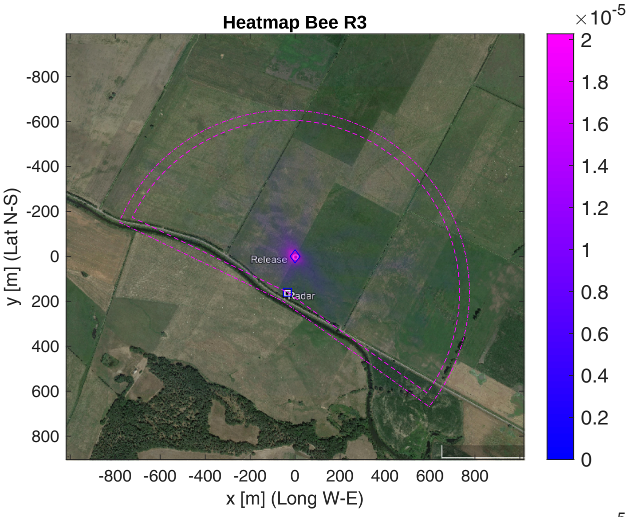
Heatmap Bee R3
×10^-5
2
1.8
1.6
1.4
1.2
1
0.8
0.6
0.4
0.2
0
-800
-600
-400
-200
0
200
400
600
800
y [m] (Lat N-S)
x [m] (Long W-E)
Release
Radar

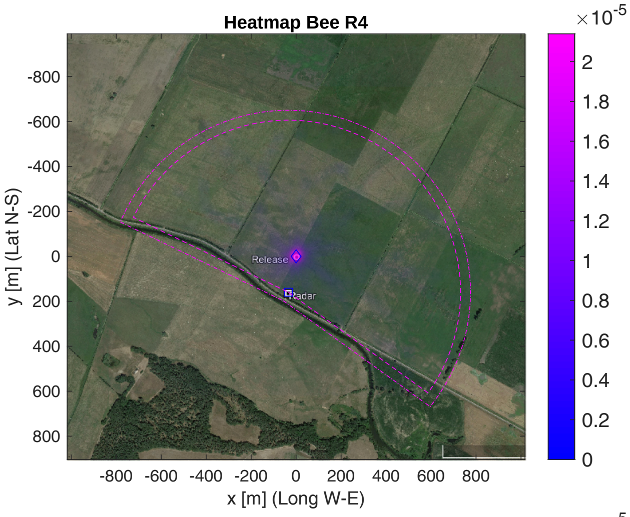
Heatmap Bee R4
×10^-5
2
1.8
1.6
1.4
1.2
1
0.8
0.6
0.4
0.2
0
-800
-600
-400
-200
0
200
400
600
800
y [m] (Lat N-S)
x [m] (Long W-E)
Release
Radar

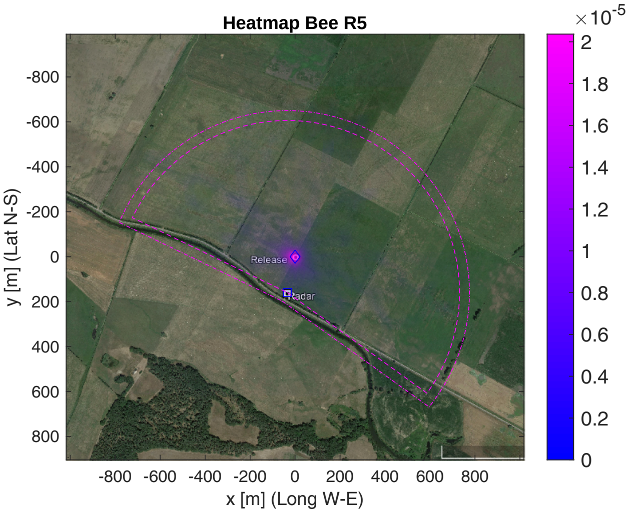
Heatmap Bee R5
×10^-5
2
1.8
1.6
1.4
1.2
1
0.8
0.6
0.4
0.2
0
-800
-600
-400
-200
0
200
400
600
800
y [m] (Lat N-S)
x [m] (Long W-E)
Release
Radar

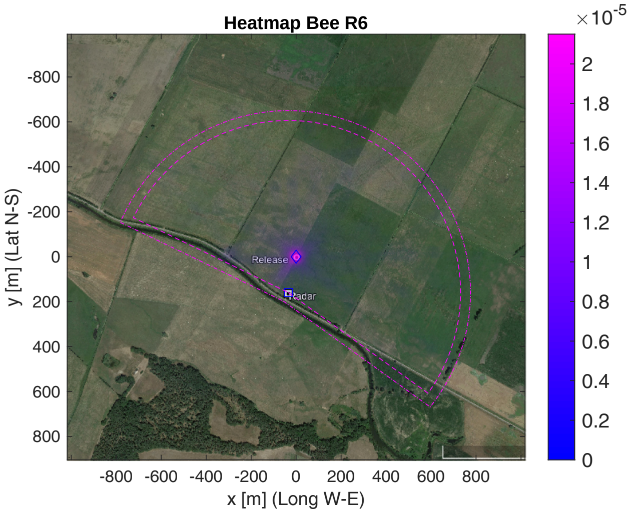
Heatmap Bee R6
×10^-5
2
1.8
1.6
1.4
1.2
1
0.8
0.6
0.4
0.2
0
-800
-600
-400
-200
0
200
400
600
800
y [m] (Lat N-S)
x [m] (Long W-E)
Release
Radar

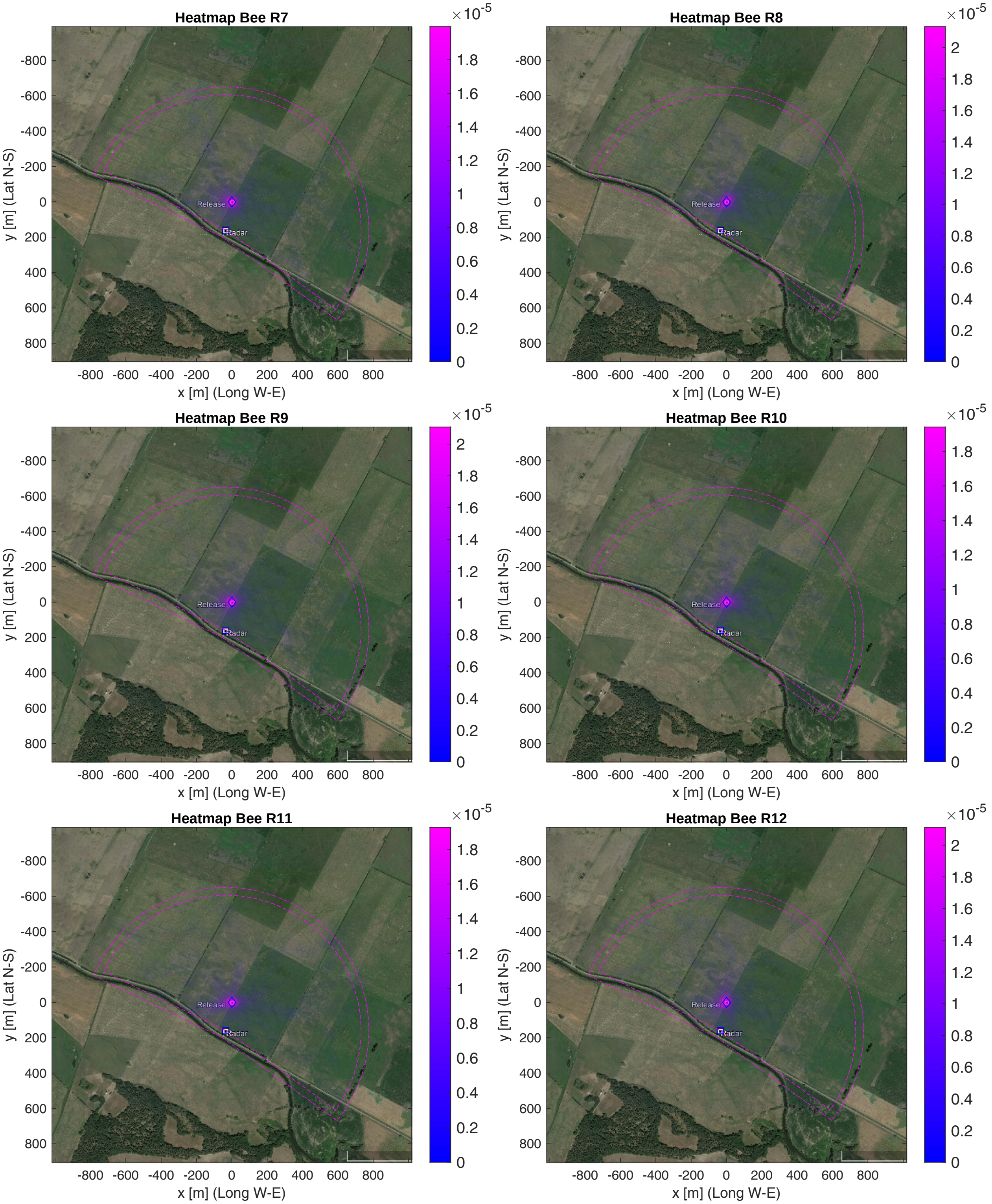
Heatmap Bee R7
Heatmap Bee R8
Heatmap Bee R9
Heatmap Bee R10
Heatmap Bee R11
Heatmap Bee R12
$\times 10^{-5}$
y [m] (Lat N-S)
x [m] (Long W-E)
Release
Radar

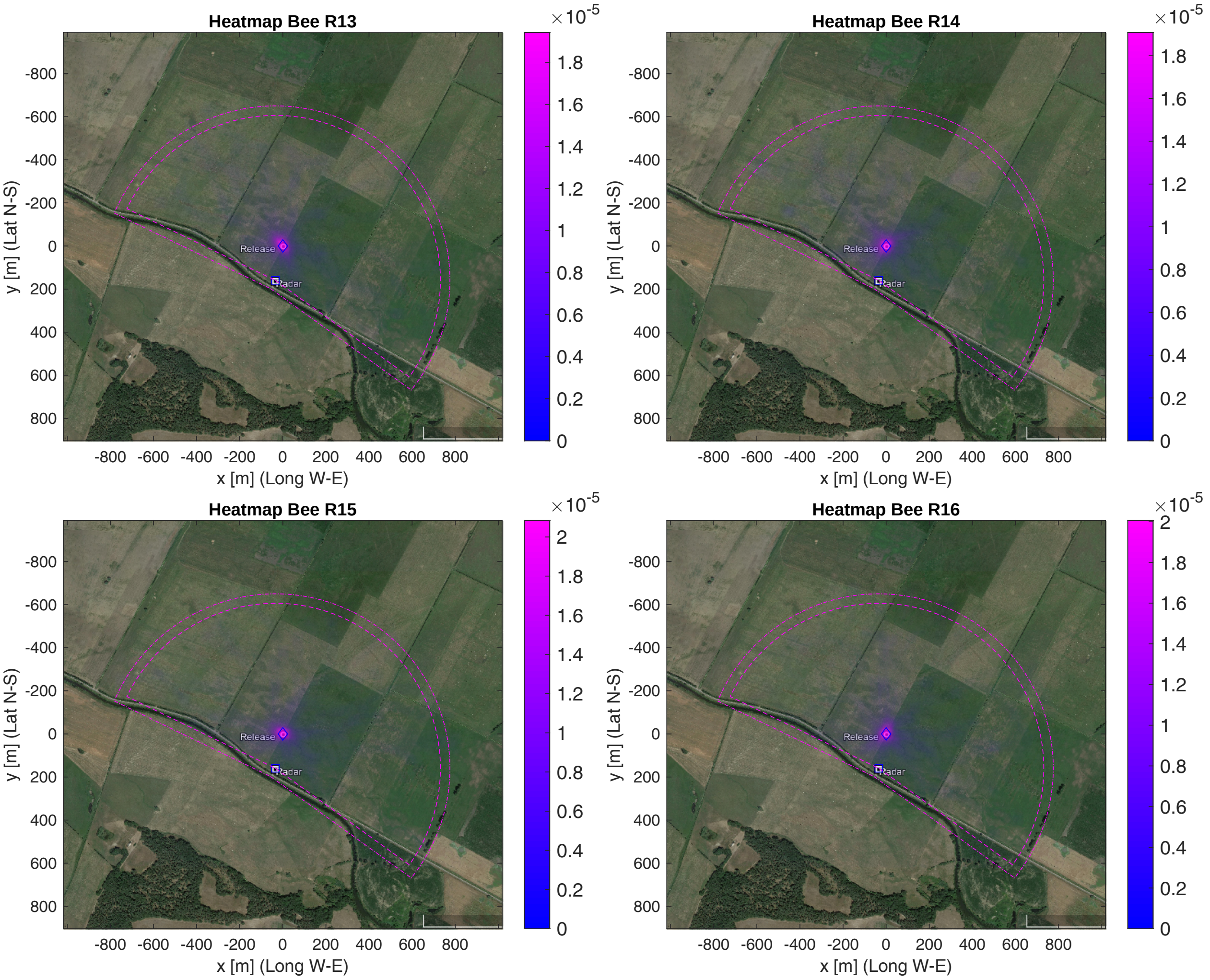
Heatmap Bee R13
Heatmap Bee R14
Heatmap Bee R15
Heatmap Bee R16
×10-5
Release
Radar
y [m] (Lat N-S)
x [m] (Long W-E)
-800 -600 -400 -200 0 200 400 600 800
0 0.2 0.4 0.6 0.8 1 1.2 1.4 1.6 1.8 2

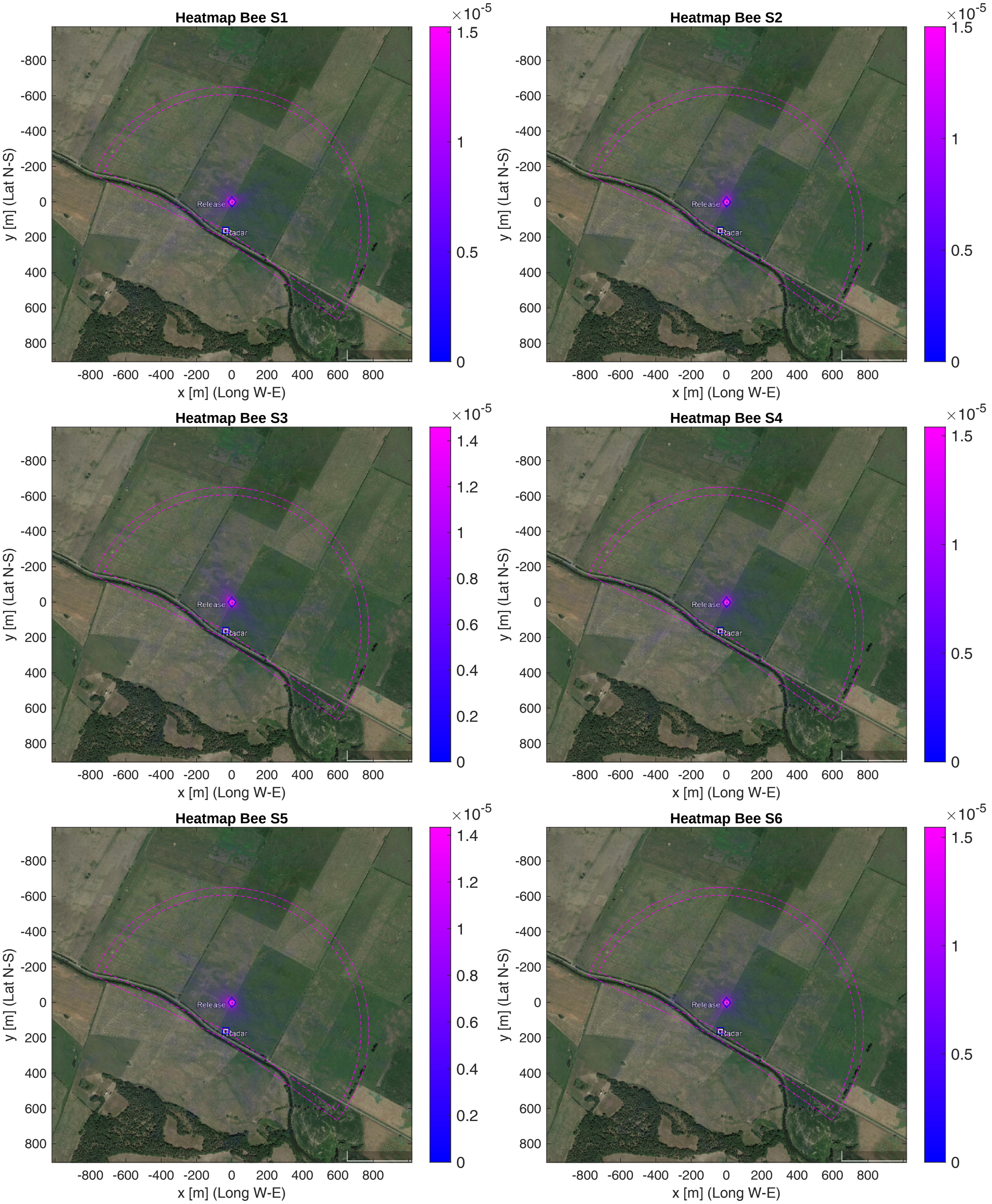
Heatmap Bee S1
Heatmap Bee S2
Heatmap Bee S3
Heatmap Bee S4
Heatmap Bee S5
Heatmap Bee S6
Release
Radar
y [m] (Lat N-S)
x [m] (Long W-E)
-800 -600 -400 -200 0 200 400 600 800
$\times 10^{-5}$
1.5
1
0.5
0
1.4
1.2
0.8
0.6
0.4
0.2

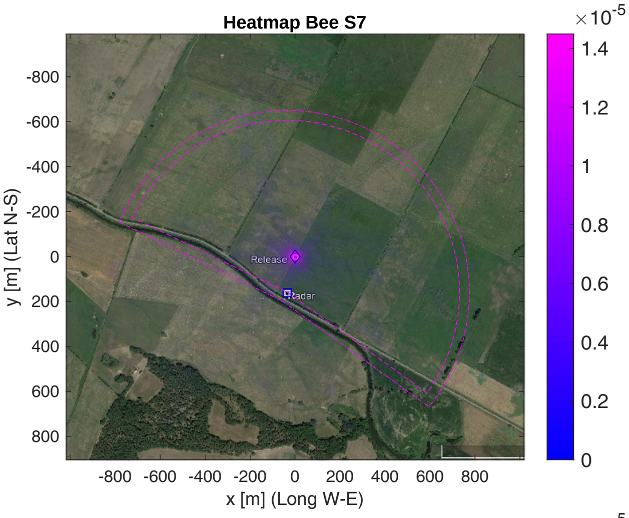
Heatmap Bee S7
×10^-5
y [m] (Lat N-S)
x [m] (Long W-E)
Release
Radar

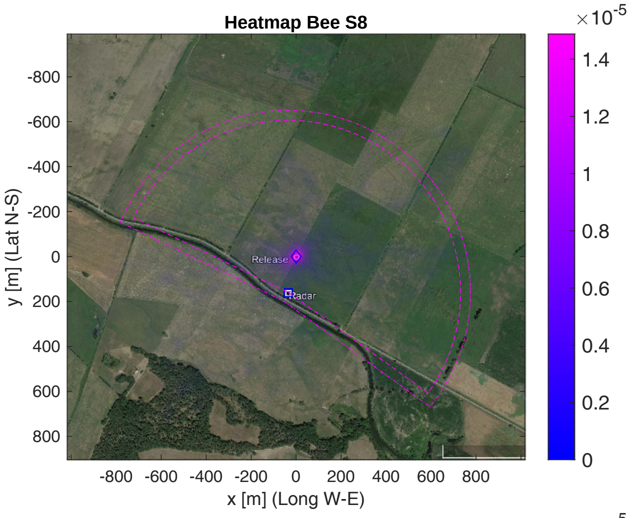
Heatmap Bee S8
×10^-5
y [m] (Lat N-S)
x [m] (Long W-E)
Release
Radar

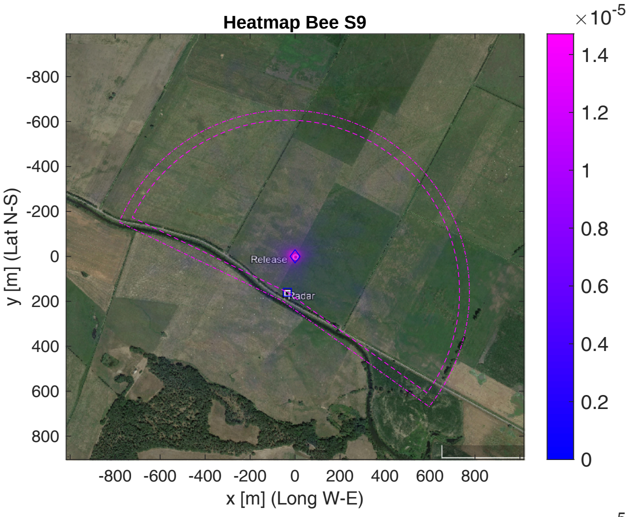
Heatmap Bee S9
×10^-5
y [m] (Lat N-S)
x [m] (Long W-E)
Release
Radar

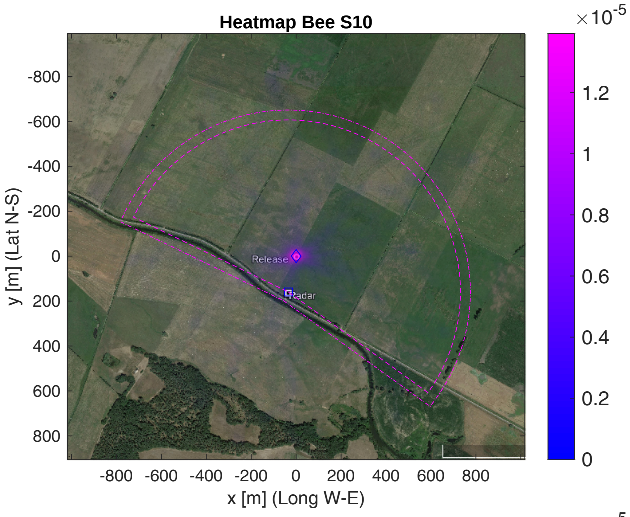
Heatmap Bee S10
×10^-5
y [m] (Lat N-S)
x [m] (Long W-E)
Release
Radar

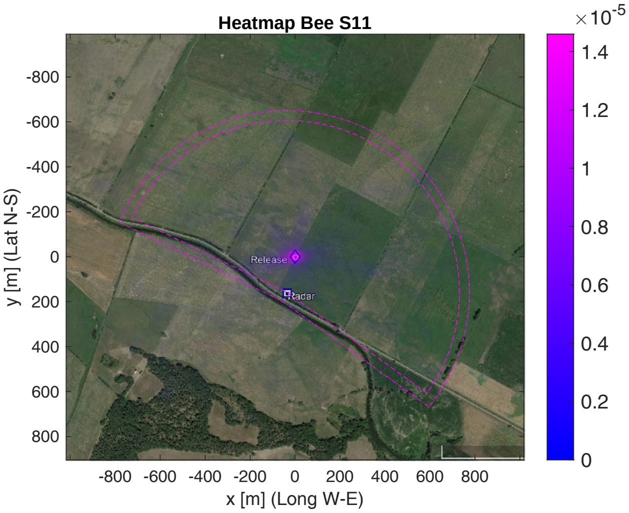
Heatmap Bee S11
×10^-5
y [m] (Lat N-S)
x [m] (Long W-E)
Release
Radar

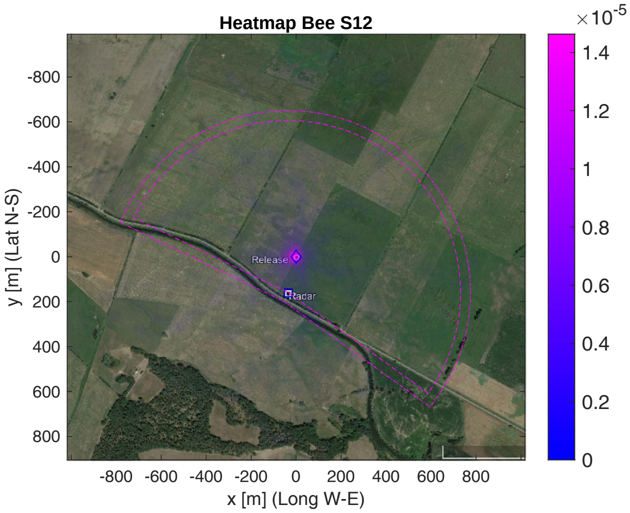
Heatmap Bee S12
×10^-5
y [m] (Lat N-S)
x [m] (Long W-E)
Release
Radar

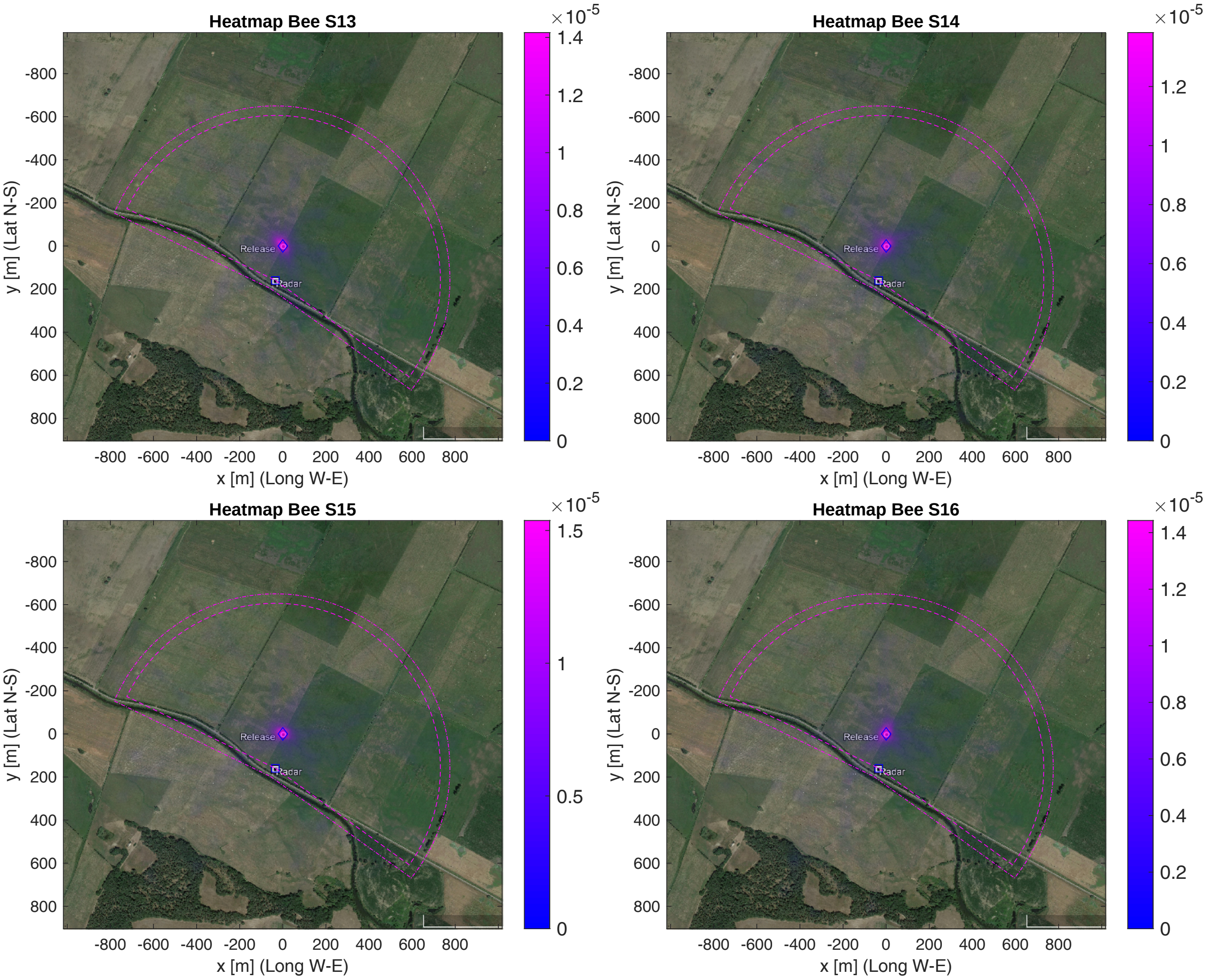
Heatmap Bee S13
Heatmap Bee S14
Heatmap Bee S15
Heatmap Bee S16
×10-5
1.4
1.2
1
0.8
0.6
0.4
0.2
0
1.5
0.5
Release
Radar
y [m] (Lat N-S)
x [m] (Long W-E)
-800
-600
-400
-200
0
200
400
600
800
