# Supplementary material for: Generalization of navigation memory in honeybees
Source: Front Behav Neurosci. 2023 Mar 6;17:1070957. doi: 10.3389/fnbeh.2023.1070957 (PMC10025308; doi:10.3389/fnbeh.2023.1070957)

# GENERALIZATION OF NAVIGATION MEMORY IN HONEYBEES

SUPPLEMENT DS 05: SIGNIFICANCE ANALYSES OF HEAT MAP TILES

**Eric Bullinger***
Otto-von-Guericke-Universität Magdeburg
Institut für Automatisierungstechnik
Universitätsplatz 2, 39106 Magdeburg, Germany
eric.bullinger@ovgu.de

**Uwe Greggers & Randolf Menzel***
Freie Universität Berlin
Neurobiologie
Königin Luisestr. 1 -3, 14195 Berlin, Germany
menzel@neurobiologie.fu-berlin.de

14 February 2023

- Tile 00: p. 4
- Tile 01: p. 4
- Tile 02: p. 5
- Tile 03: p. 5
- Tile 04: p. 6
- Tile 05: p. 6
- Tile 06: p. 7
- Tile 07: p. 7
- Tile 08: p. 8
- Tile 09: p. 8
- Tile 0A: p. 9
- Tile 0B: p. 9
- Tile 0C: p. 10
- Tile 0D: p. 10
- Tile 0E: p. 11
- Tile 0F: p. 11
- Tile 0G: p. 12
- Tile 0H: p. 12
- Tile 10: p. 13
- Tile 11: p. 13
- Tile 12: p. 14
- Tile 13: p. 14
- Tile 14: p. 15
- Tile 15: p. 15
- Tile 16: p. 16
- Tile 17: p. 16
- Tile 18: p. 17
- Tile 19: p. 17
- Tile 1A: p. 18
- Tile 1B: p. 18
- Tile 1C: p. 19
- Tile 1D: p. 19
- Tile 1E: p. 20
- Tile 1F: p. 20
- Tile 1G: p. 21
- Tile 1H: p. 21
- Tile 20: p. 22
- Tile 21: p. 22
- Tile 22: p. 23
- Tile 23: p. 23
- Tile 24: p. 24
- Tile 25: p. 24
- Tile 26: p. 25
- Tile 27: p. 25
- Tile 28: p. 26
- Tile 29: p. 26
- Tile 2A: p. 27
- Tile 2B: p. 27
- Tile 2C: p. 28
- Tile 2D: p. 28
- Tile 2E: p. 29
- Tile 2F: p. 29
- Tile 2G: p. 30
- Tile 2H: p. 30
- Tile 30: p. 31
- Tile 31: p. 31
- Tile 32: p. 32
- Tile 33: p. 32
- Tile 34: p. 33
- Tile 35: p. 33
- Tile 36: p. 34
- Tile 37: p. 34
- Tile 38: p. 35
- Tile 39: p. 35
- Tile 3A: p. 36
- Tile 3B: p. 36
- Tile 3C: p. 37
- Tile 3D: p. 37
- Tile 3E: p. 38
- Tile 3F: p. 38
- Tile 3G: p. 39
- Tile 3H: p. 39
- Tile 40: p. 40
- Tile 41: p. 40
- Tile 42: p. 41
- Tile 43: p. 41
- Tile 44: p. 42
- Tile 45: p. 42
- Tile 46: p. 43
- Tile 47: p. 43
- Tile 48: p. 44
- Tile 49: p. 44
- Tile 4A: p. 45
- Tile 4B: p. 45
- Tile 4C: p. 46
- Tile 4D: p. 46
- Tile 4E: p. 47
- Tile 4F: p. 47
- Tile 4G: p. 48
- Tile 4H: p. 48
- Tile 50: p. 49
- Tile 51: p. 49
- Tile 52: p. 50
- Tile 53: p. 50
- Tile 54: p. 51
- Tile 55: p. 51
- Tile 56: p. 52
- Tile 57: p. 52
- Tile 58: p. 53
- Tile 59: p. 53
- Tile 5A: p. 54
- Tile 5B: p. 54
- Tile 5C: p. 55
- Tile 5D: p. 55
- Tile 5E: p. 56
- Tile 5F: p. 56
- Tile 5G: p. 57
- Tile 5H: p. 57
- Tile 60: p. 58
- Tile 61: p. 58
- Tile 62: p. 59
- Tile 63: p. 59

*corresponding author

- Tile 64: p. 60
- Tile 65: p. 60
- Tile 66: p. 61
- Tile 67: p. 61
- Tile 68: p. 62
- Tile 69: p. 62
- Tile 6A: p. 63
- Tile 6B: p. 63
- Tile 6C: p. 64
- Tile 6D: p. 64
- Tile 6E: p. 65
- Tile 6F: p. 65
- Tile 6G: p. 66
- Tile 6H: p. 66
- Tile 70: p. 67
- Tile 71: p. 67
- Tile 72: p. 68
- Tile 73: p. 68
- Tile 74: p. 69
- Tile 75: p. 69
- Tile 76: p. 70
- Tile 77: p. 70
- Tile 78: p. 71
- Tile 79: p. 71
- Tile 7A: p. 72
- Tile 7B: p. 72
- Tile 7C: p. 73
- Tile 7D: p. 73
- Tile 7E: p. 74
- Tile 7F: p. 74
- Tile 7G: p. 75
- Tile 7H: p. 75
- Tile 80: p. 76
- Tile 81: p. 76
- Tile 82: p. 77
- Tile 83: p. 77
- Tile 84: p. 78
- Tile 85: p. 78
- Tile 86: p. 79
- Tile 87: p. 79
- Tile 88: p. 80
- Tile 89: p. 80
- Tile 8A: p. 81
- Tile 8B: p. 81
- Tile 8C: p. 82
- Tile 8D: p. 82
- Tile 8E: p. 83
- Tile 8F: p. 83
- Tile 8G: p. 84
- Tile 8H: p. 84
- Tile 90: p. 85
- Tile 91: p. 85
- Tile 92: p. 86
- Tile 93: p. 86
- Tile 94: p. 87
- Tile 95: p. 87
- Tile 96: p. 88
- Tile 97: p. 88
- Tile 98: p. 89
- Tile 99: p. 89
- Tile 9A: p. 90
- Tile 9B: p. 90
- Tile 9C: p. 91
- Tile 9D: p. 91
- Tile 9E: p. 92
- Tile 9F: p. 92
- Tile 9G: p. 93
- Tile 9H: p. 93
- Tile A0: p. 94
- Tile A1: p. 94
- Tile A2: p. 95
- Tile A3: p. 95
- Tile A4: p. 96
- Tile A5: p. 96
- Tile A6: p. 97
- Tile A7: p. 97
- Tile A8: p. 98
- Tile A9: p. 98
- Tile AA: p. 99
- Tile AB: p. 99
- Tile AC: p. 100
- Tile AD: p. 100
- Tile AE: p. 101
- Tile AF: p. 101
- Tile AG: p. 102
- Tile AH: p. 102
- Tile B0: p. 103
- Tile B1: p. 103
- Tile B2: p. 104
- Tile B3: p. 104
- Tile B4: p. 105
- Tile B5: p. 105
- Tile B6: p. 106
- Tile B7: p. 106
- Tile B8: p. 107
- Tile B9: p. 107
- Tile BA: p. 108
- Tile BB: p. 108
- Tile BC: p. 109
- Tile BD: p. 109
- Tile BE: p. 110
- Tile BF: p. 110
- Tile BG: p. 111
- Tile BH: p. 111
- Tile C0: p. 112
- Tile C1: p. 112
- Tile C2: p. 113
- Tile C3: p. 113
- Tile C4: p. 114
- Tile C5: p. 114
- Tile C6: p. 115
- Tile C7: p. 115
- Tile C8: p. 116
- Tile C9: p. 116
- Tile CA: p. 117
- Tile CB: p. 117
- Tile CC: p. 118
- Tile CD: p. 118
- Tile CE: p. 119
- Tile CF: p. 119
- Tile CG: p. 120
- Tile CH: p. 120
- Tile D0: p. 121
- Tile D1: p. 121
- Tile D2: p. 122
- Tile D3: p. 122
- Tile D4: p. 123
- Tile D5: p. 123
- Tile D6: p. 124
- Tile D7: p. 124
- Tile D8: p. 125
- Tile D9: p. 125
- Tile DA: p. 126
- Tile DB: p. 126
- Tile DC: p. 127
- Tile DD: p. 127
- Tile DE: p. 128
- Tile DF: p. 128
- Tile DG: p. 129
- Tile DH: p. 129
- Tile E0: p. 130
- Tile E1: p. 130
- Tile E2: p. 131
- Tile E3: p. 131
- Tile E4: p. 132
- Tile E5: p. 132
- Tile E6: p. 133
- Tile E7: p. 133
- Tile E8: p. 134
- Tile E9: p. 134
- Tile EA: p. 135
- Tile EB: p. 135
- Tile EC: p. 136
- Tile ED: p. 136
- Tile EE: p. 137
- Tile EF: p. 137
- Tile EG: p. 138
- Tile EH: p. 138
- Tile F0: p. 139
- Tile F1: p. 139
- Tile F2: p. 140
- Tile F3: p. 140
- Tile F4: p. 141
- Tile F5: p. 141
- Tile F6: p. 142
- Tile F7: p. 142
- Tile F8: p. 143
- Tile F9: p. 143
- Tile FA: p. 144
- Tile FB: p. 144
- Tile FC: p. 145
- Tile FD: p. 145
- Tile FE: p. 146
- Tile FF: p. 146
- Tile FG: p. 147
- Tile FH: p. 147
- Tile G0: p. 148
- Tile G1: p. 148

- Tile G2: p. 149
- Tile G3: p. 149
- Tile G4: p. 150
- Tile G5: p. 150
- Tile G6: p. 151
- Tile G7: p. 151
- Tile G8: p. 152
- Tile G9: p. 152
- Tile GA: p. 153
- Tile GB: p. 153
- Tile GC: p. 154
- Tile GD: p. 154
- Tile GE: p. 155
- Tile GF: p. 155
- Tile GG: p. 156
- Tile GH: p. 156
- Tile H0: p. 157
- Tile H1: p. 157
- Tile H2: p. 158
- Tile H3: p. 158
- Tile H4: p. 159
- Tile H5: p. 159
- Tile H6: p. 160
- Tile H7: p. 160
- Tile H8: p. 161
- Tile H9: p. 161
- Tile HA: p. 162
- Tile HB: p. 162
- Tile HC: p. 163
- Tile HD: p. 163
- Tile HE: p. 164
- Tile HF: p. 164
- Tile HG: p. 165
- Tile HH: p. 165
- Tile I0: p. 166
- Tile I1: p. 166
- Tile I2: p. 167
- Tile I3: p. 167
- Tile I4: p. 168
- Tile I5: p. 168
- Tile I6: p. 169
- Tile I7: p. 169
- Tile I8: p. 170
- Tile I9: p. 170
- Tile IA: p. 171
- Tile IB: p. 171
- Tile IC: p. 172
- Tile ID: p. 172
- Tile IE: p. 173
- Tile IF: p. 173
- Tile IG: p. 174
- Tile IH: p. 174

**Heatmap Analysis of Box 00**

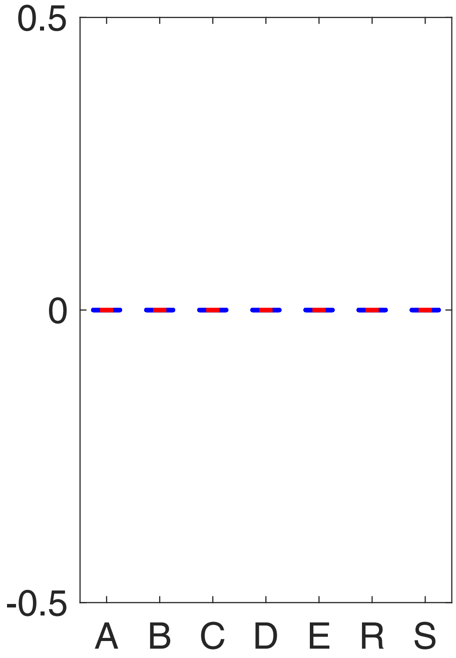


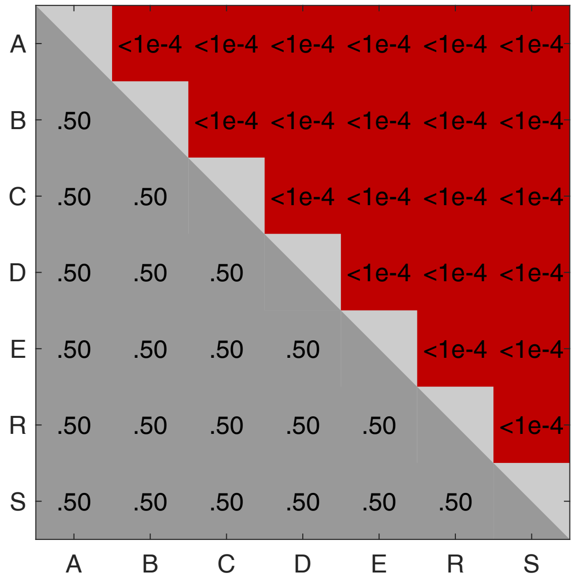


**Heatmap Analysis of Box 01**

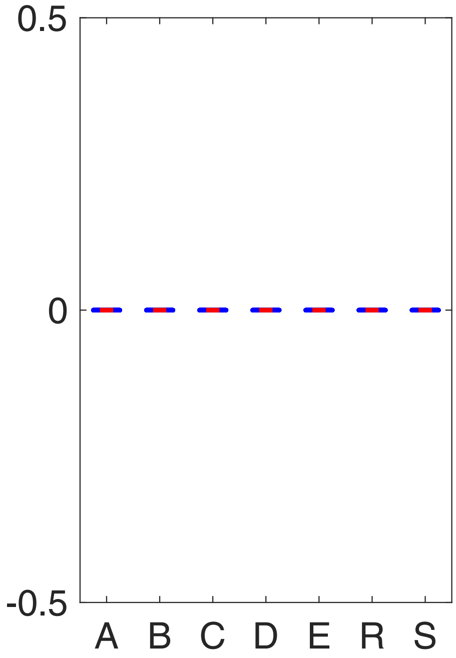


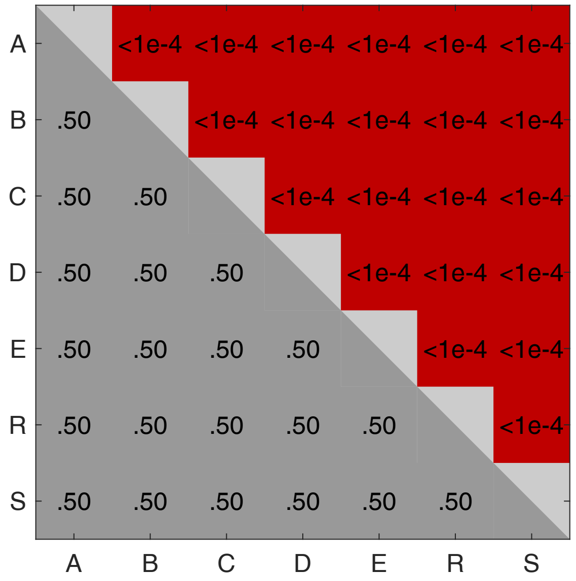

**Heatmap Analysis of Box 02**

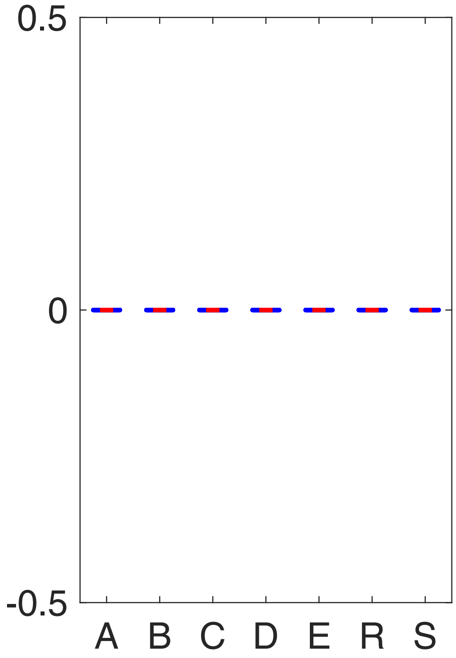


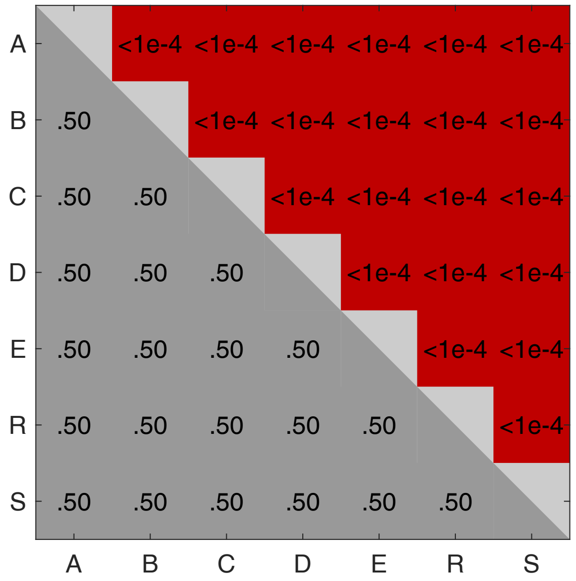


**Heatmap Analysis of Box 03**

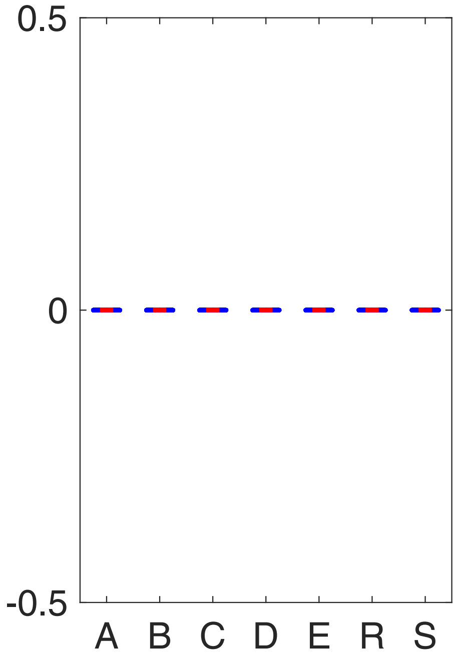


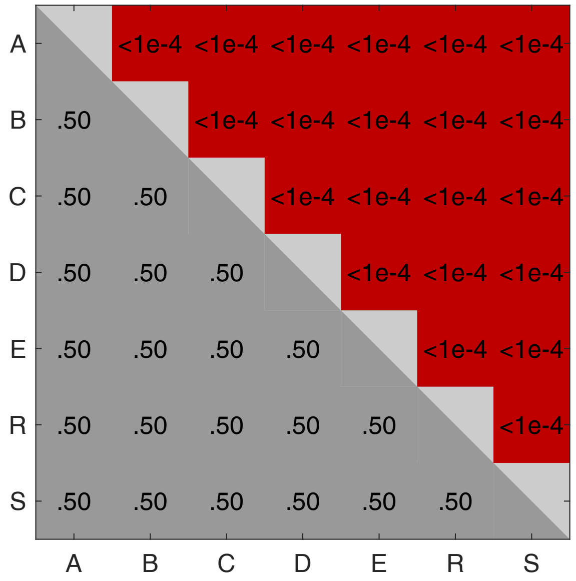

**Heatmap Analysis of Box 04**

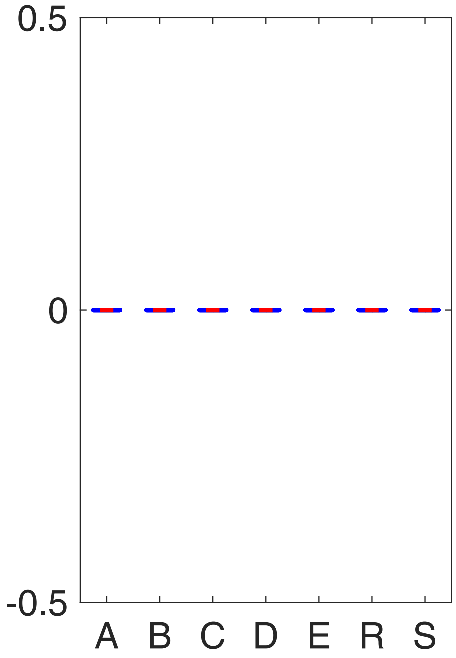


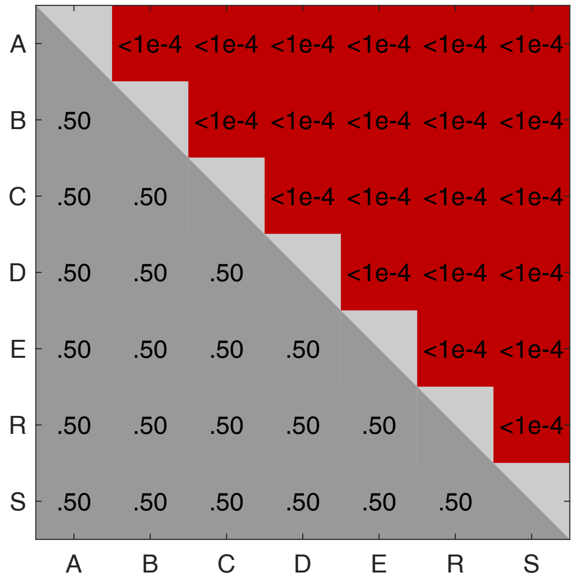


**Heatmap Analysis of Box 05**

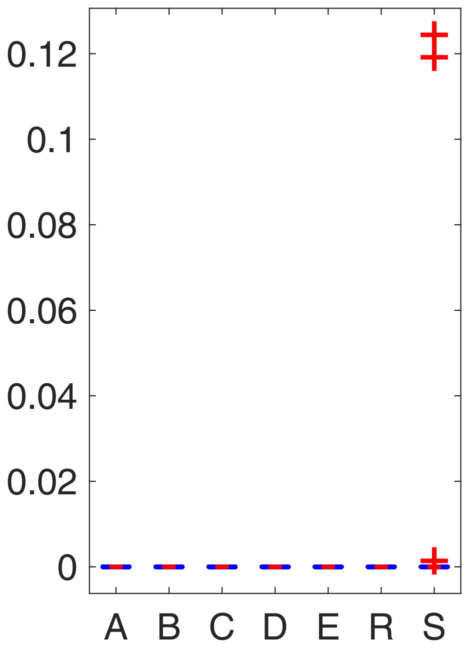


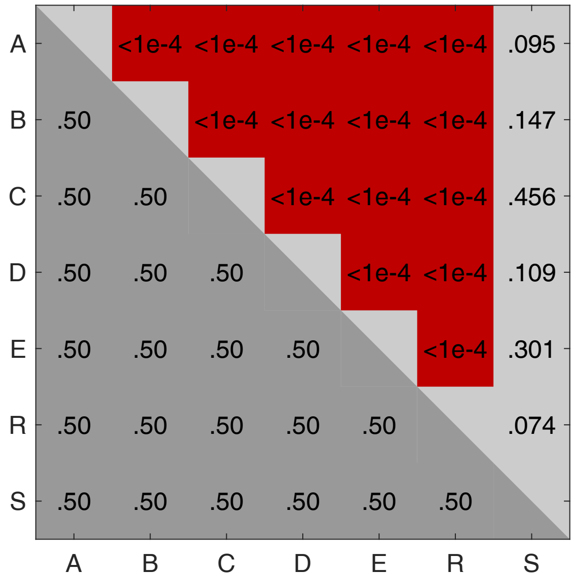

**Heatmap Analysis of Box 06**

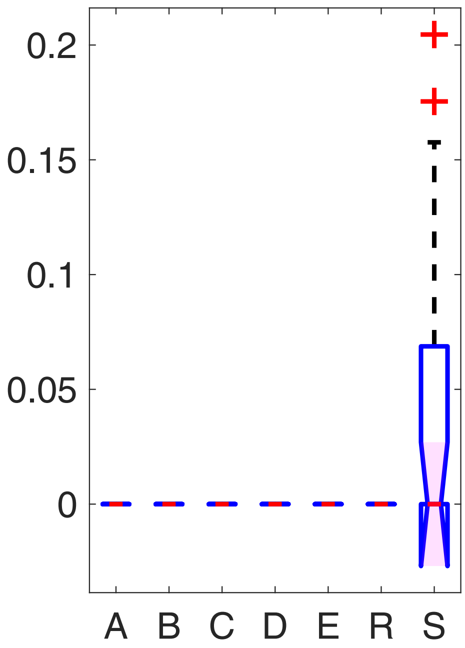


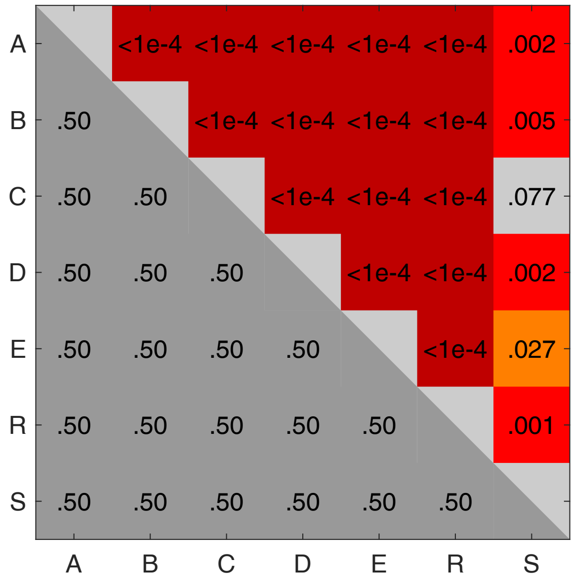


**Heatmap Analysis of Box 07**

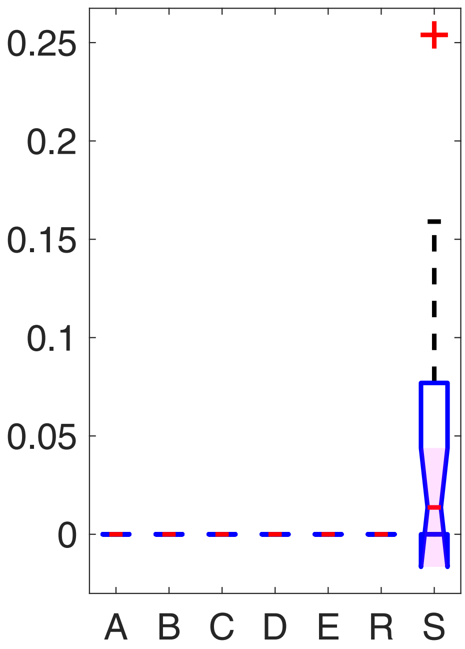


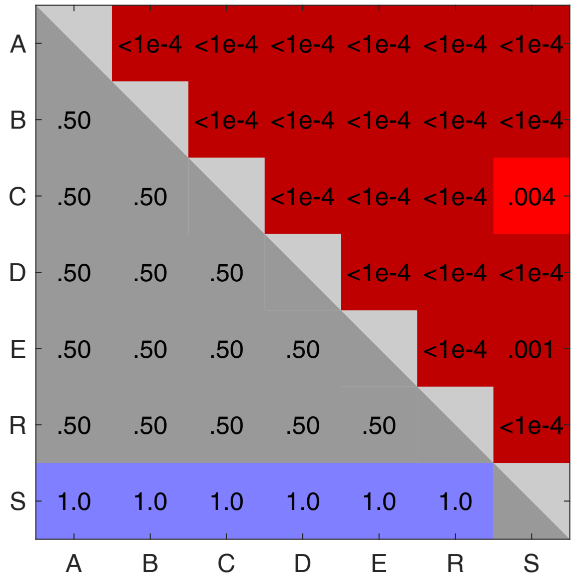

**Heatmap Analysis of Box 08**

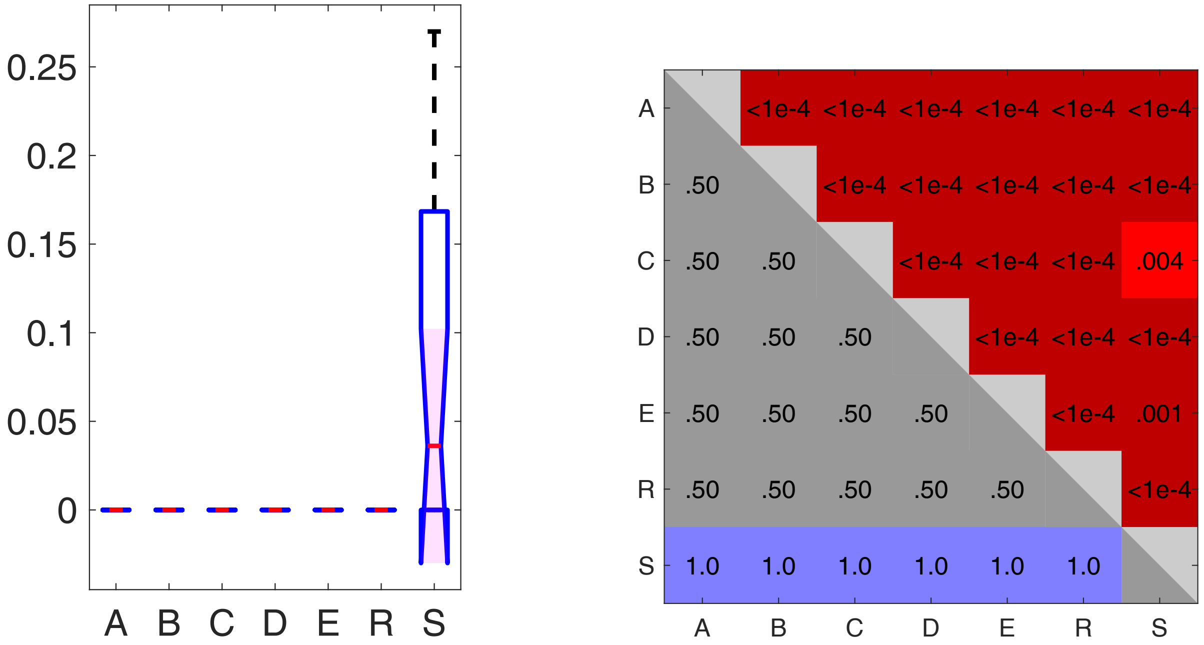


**Heatmap Analysis of Box 09**

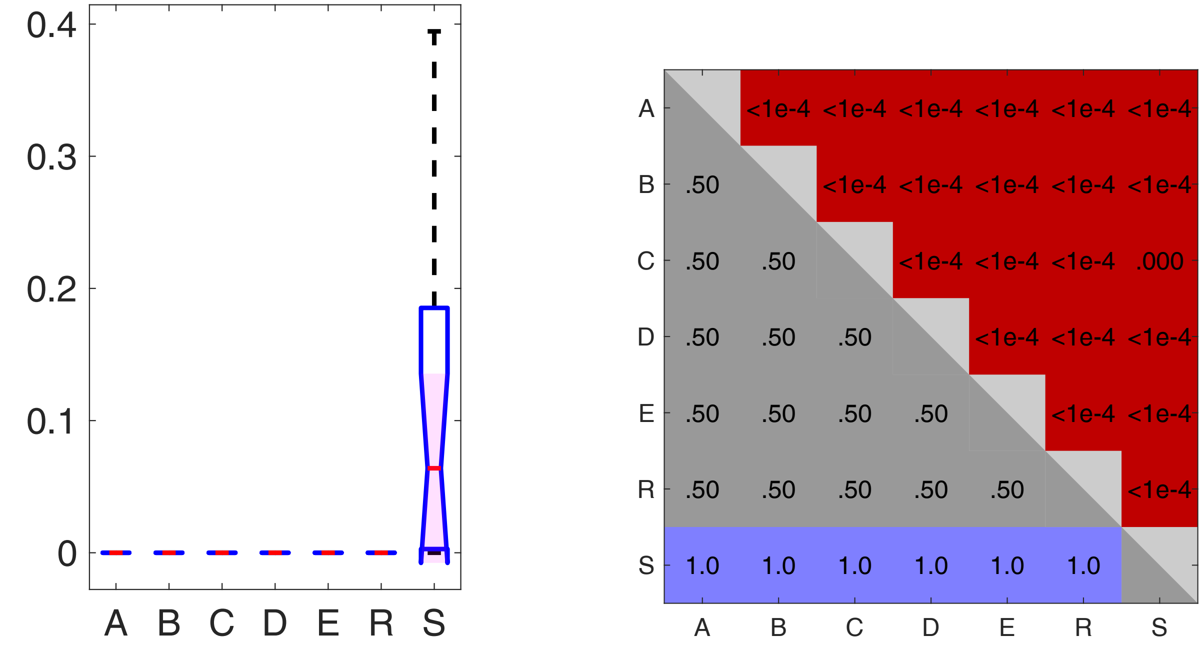

**Heatmap Analysis of Box 0A**

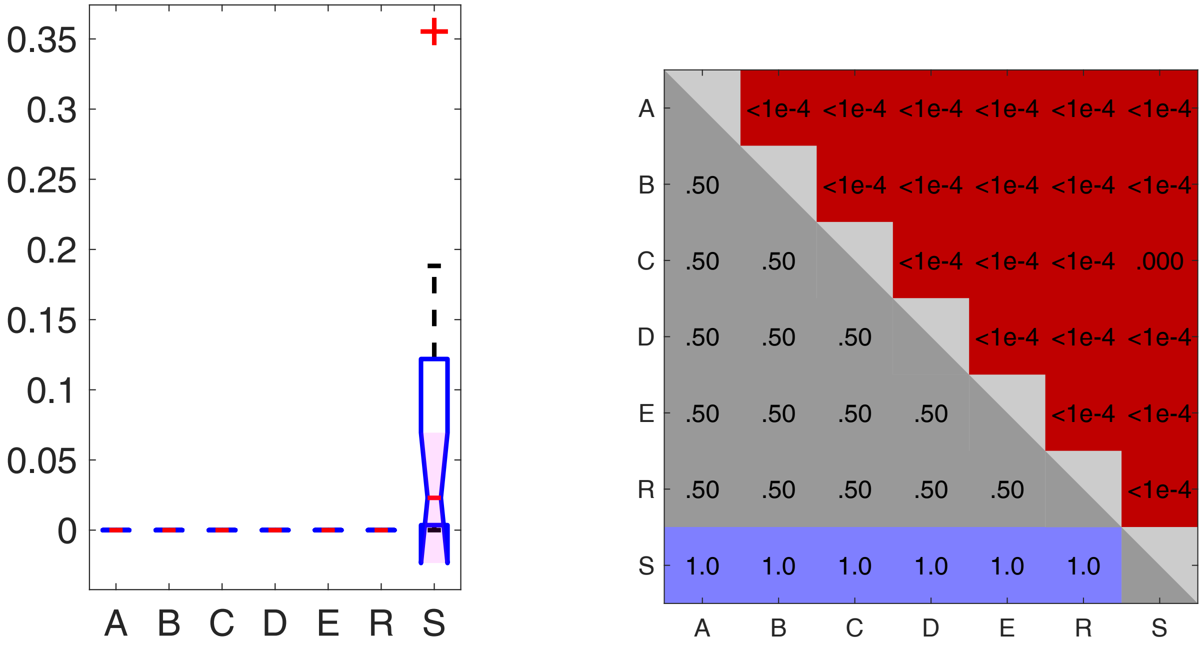


**Heatmap Analysis of Box 0B**

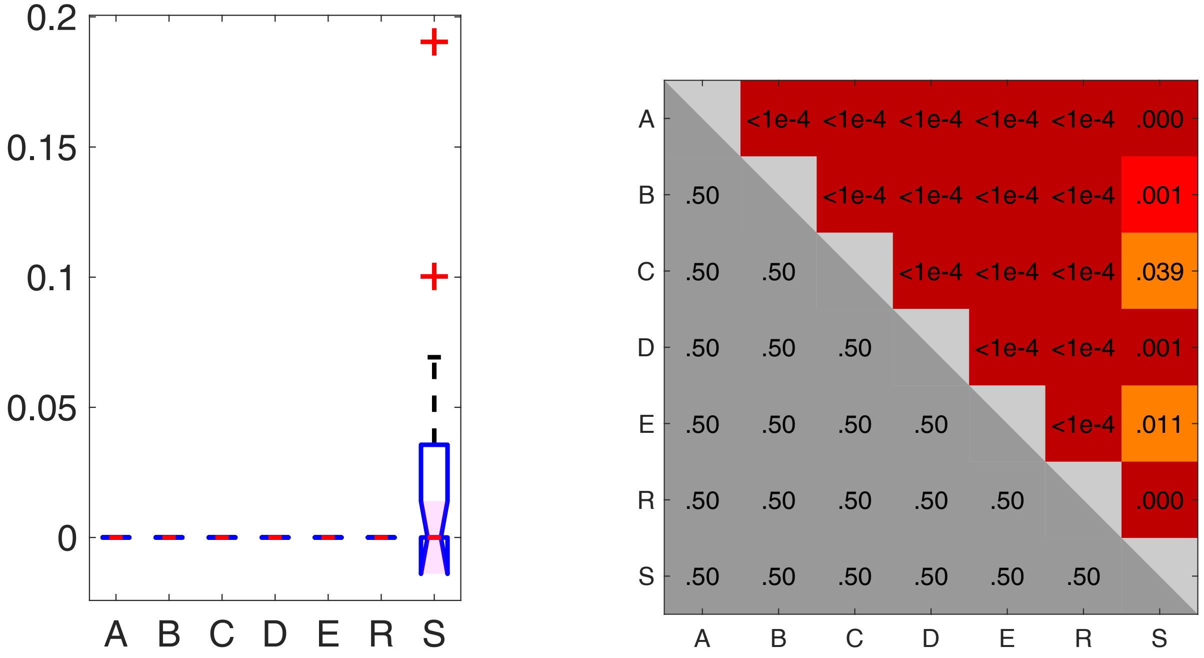

**Heatmap Analysis of Box 0C**

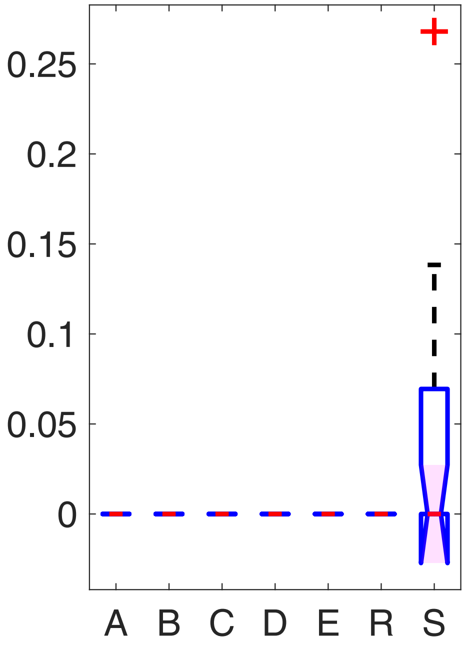


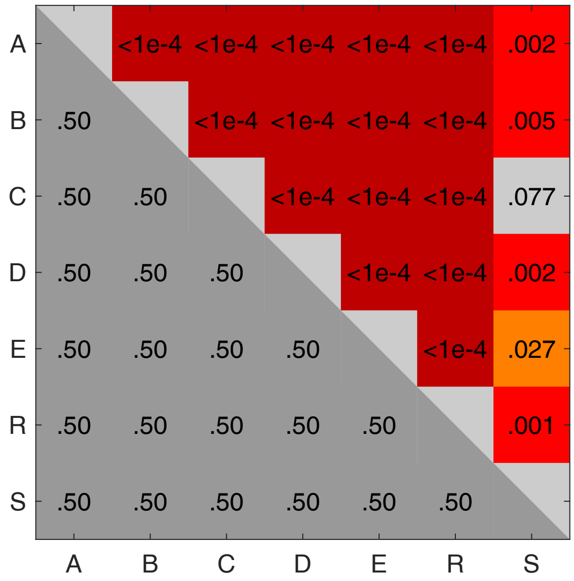


**Heatmap Analysis of Box 0D**

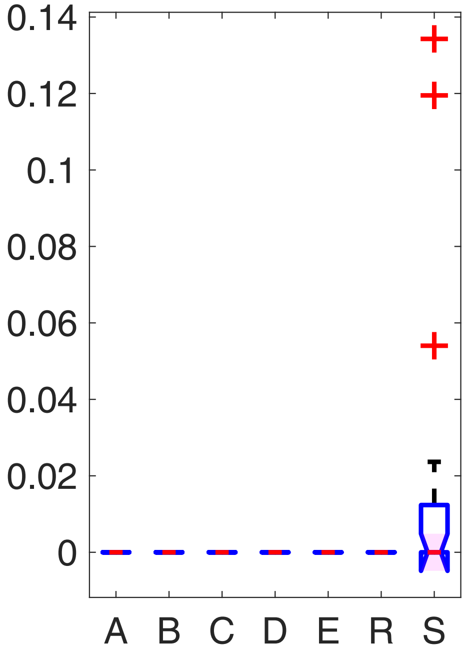


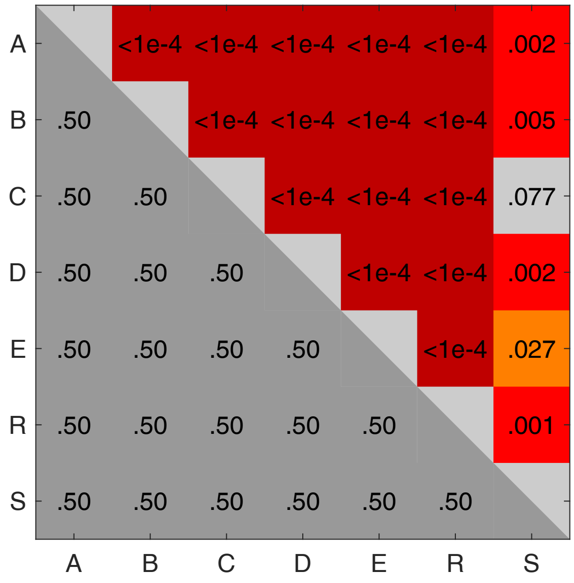

**Heatmap Analysis of Box 0E**

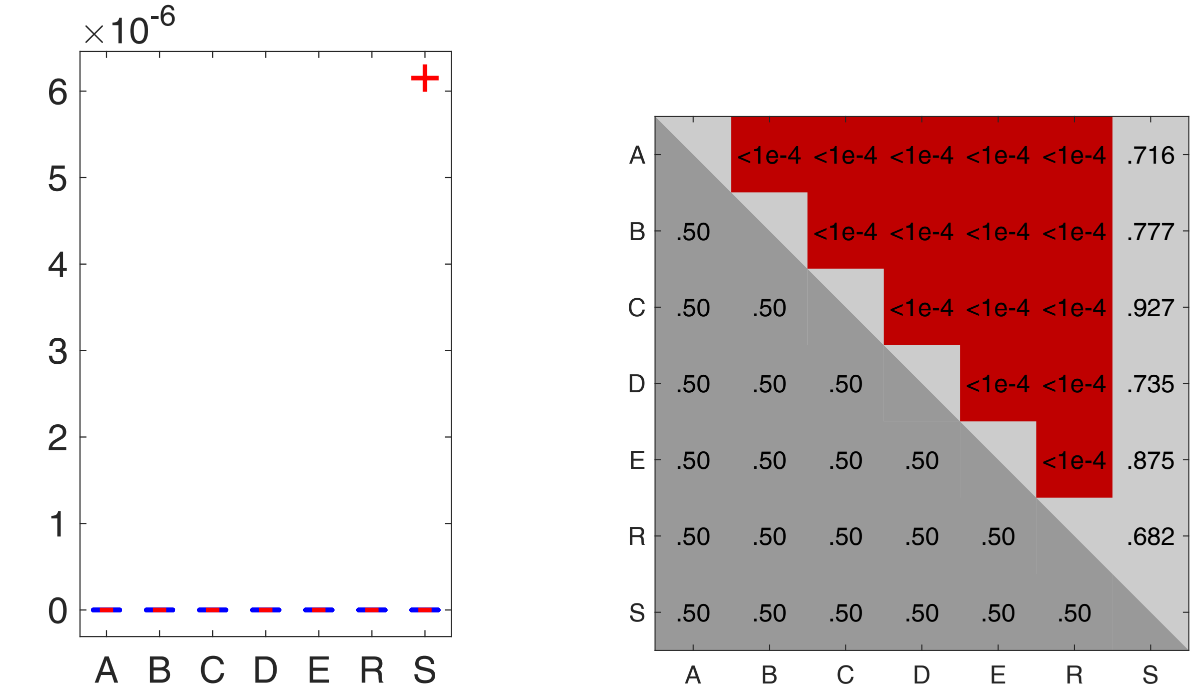


**Heatmap Analysis of Box 0F**

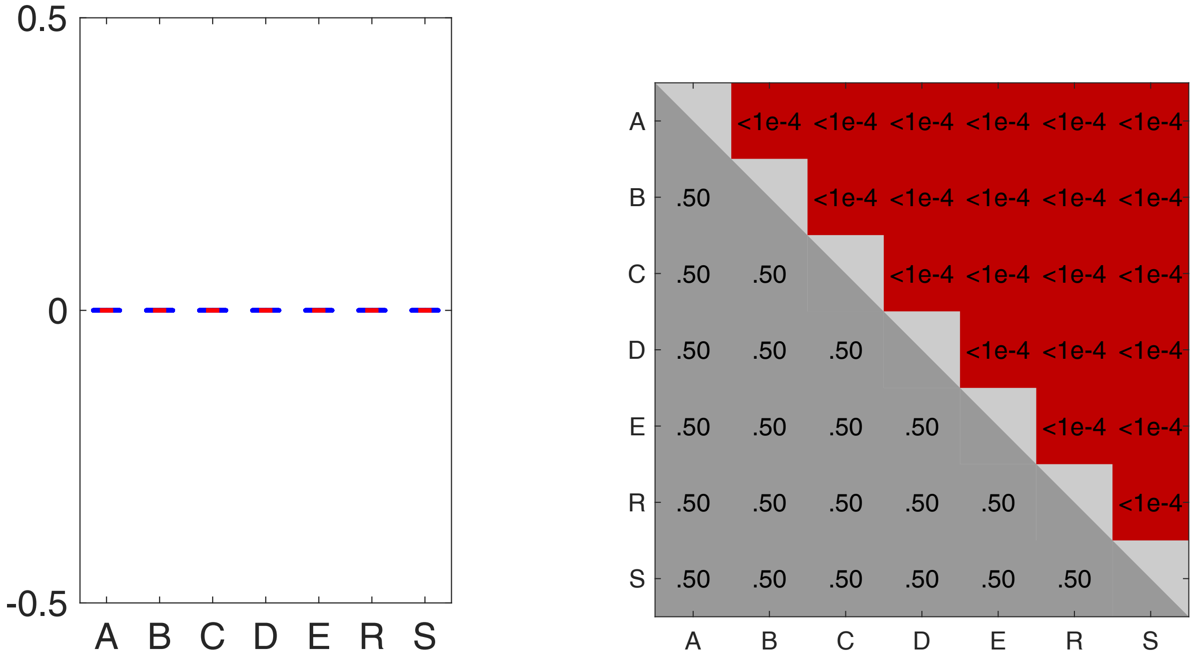

**Heatmap Analysis of Box 0G**

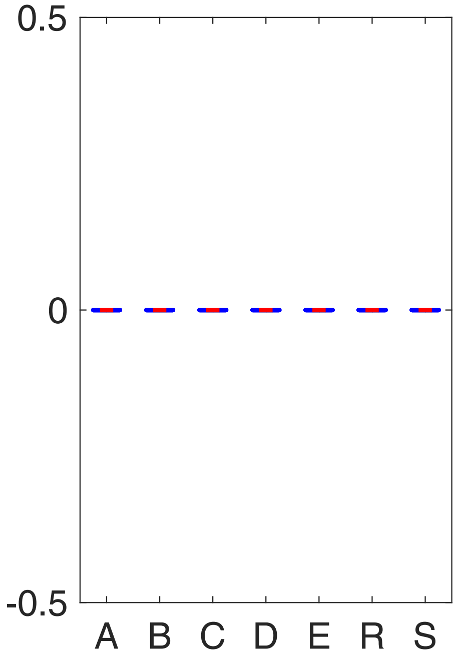


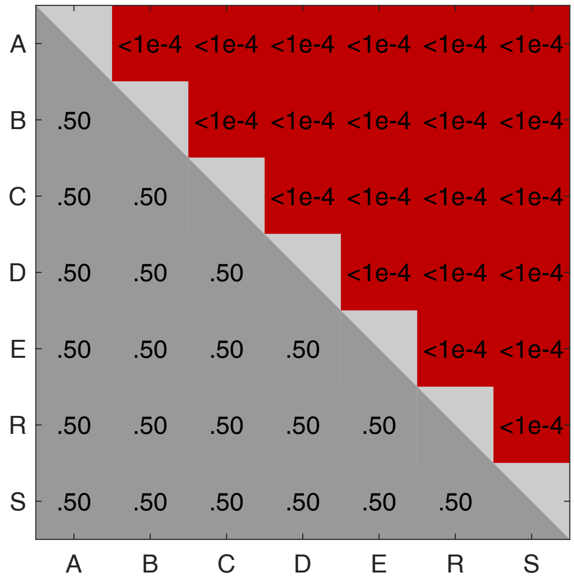


**Heatmap Analysis of Box 0H**

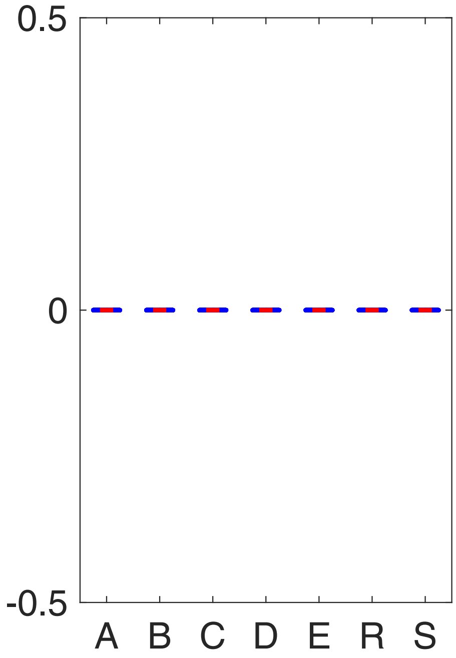


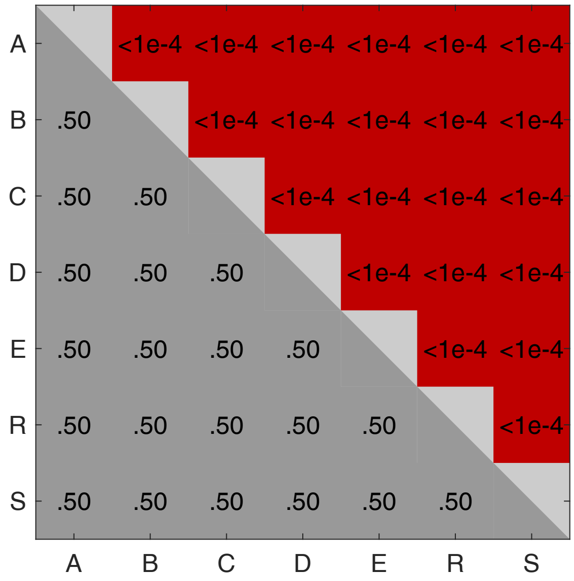

**Heatmap Analysis of Box 10**

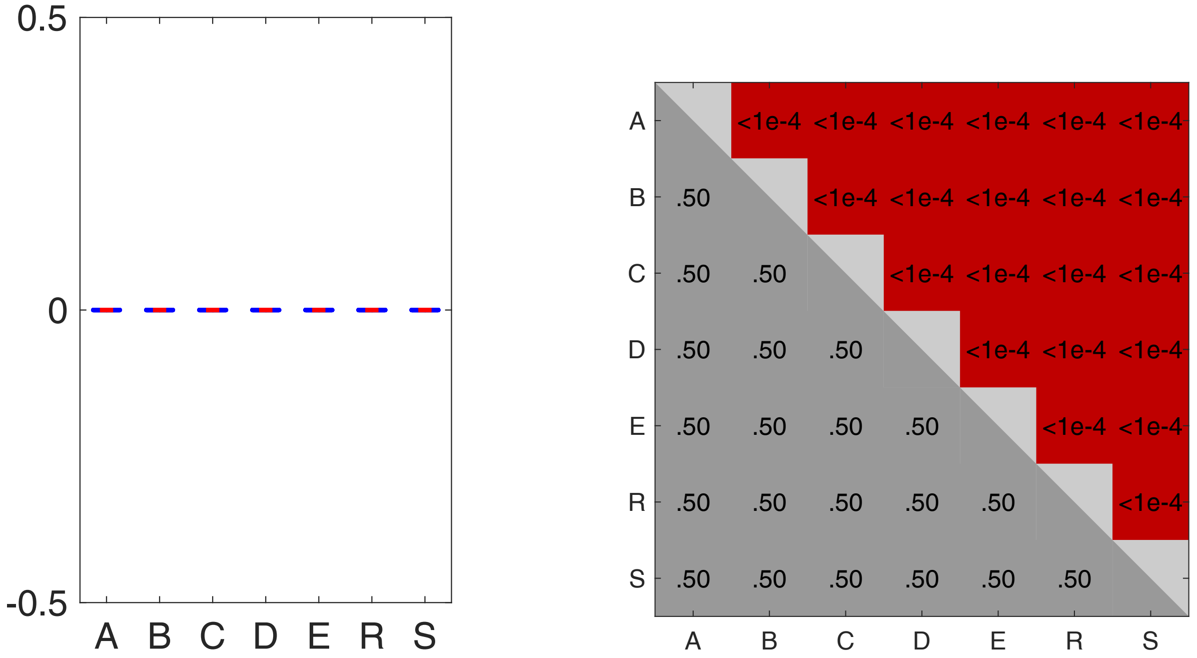


**Heatmap Analysis of Box 11**

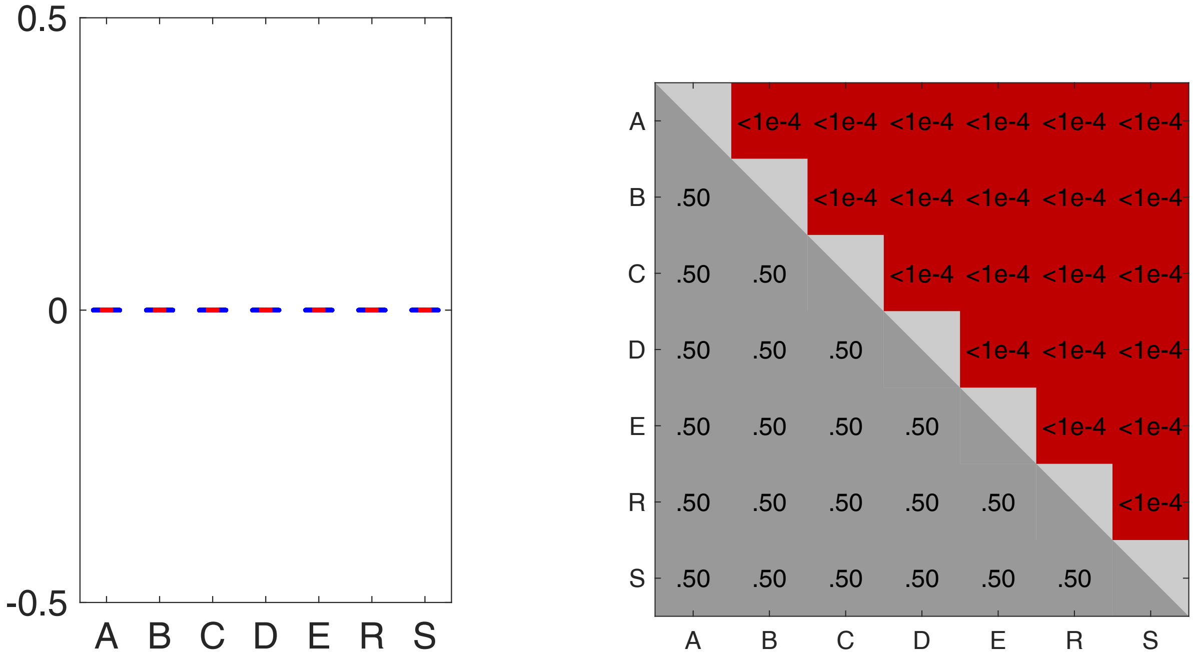

**Heatmap Analysis of Box 12**

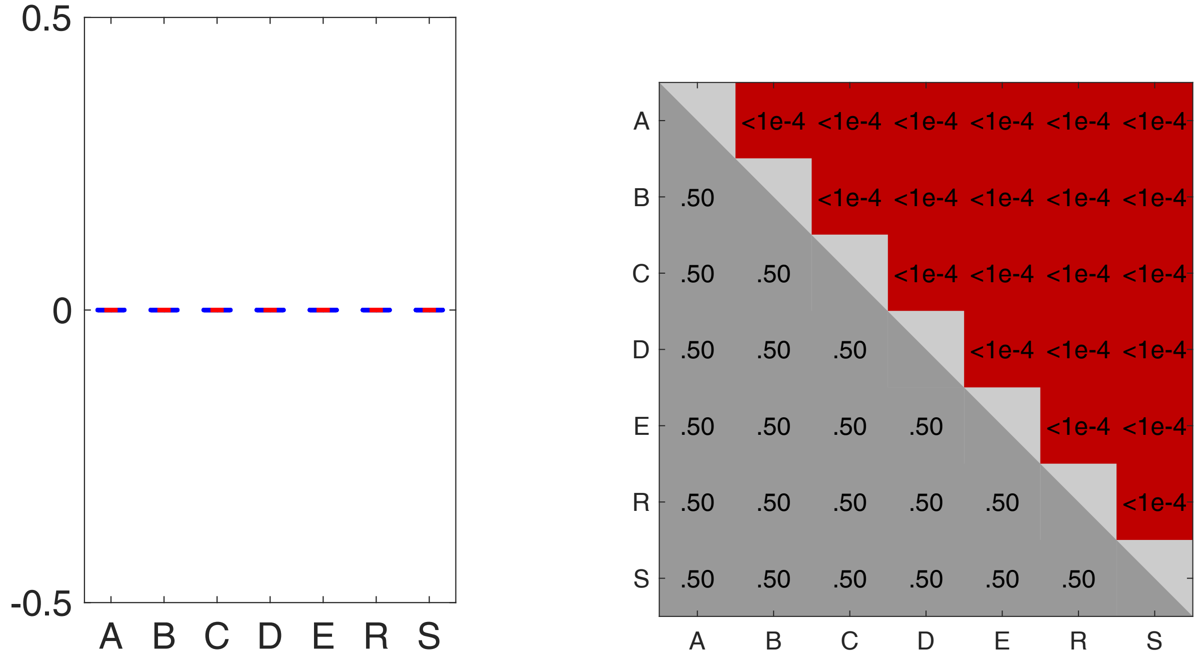


**Heatmap Analysis of Box 13**

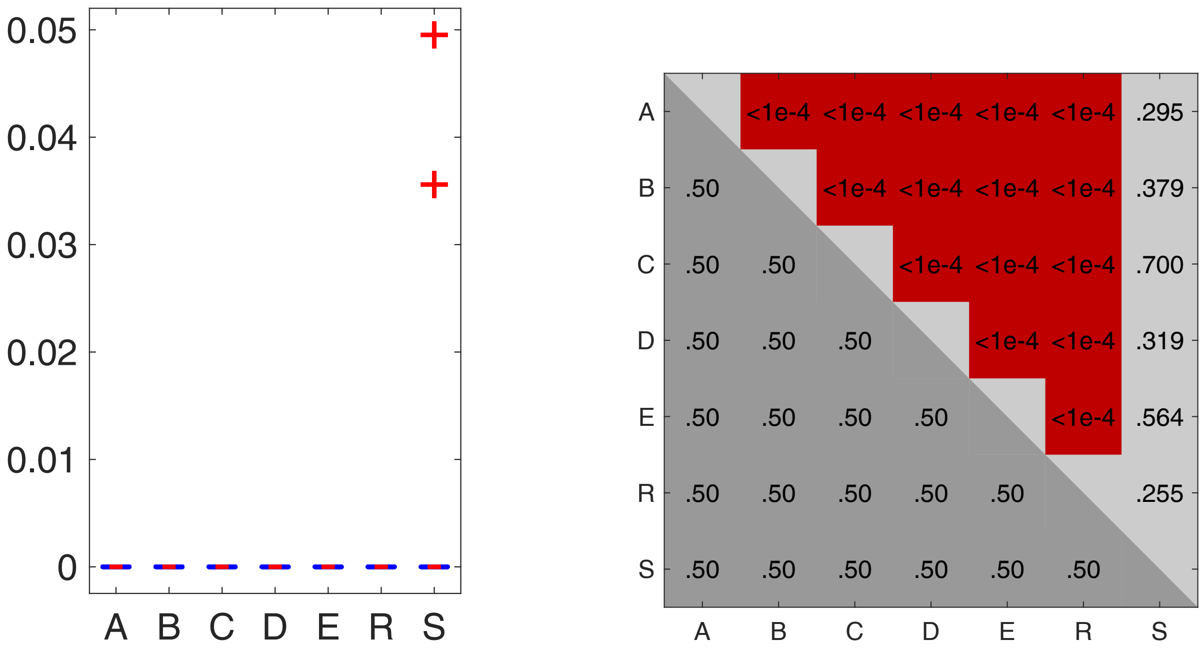

**Heatmap Analysis of Box 14**

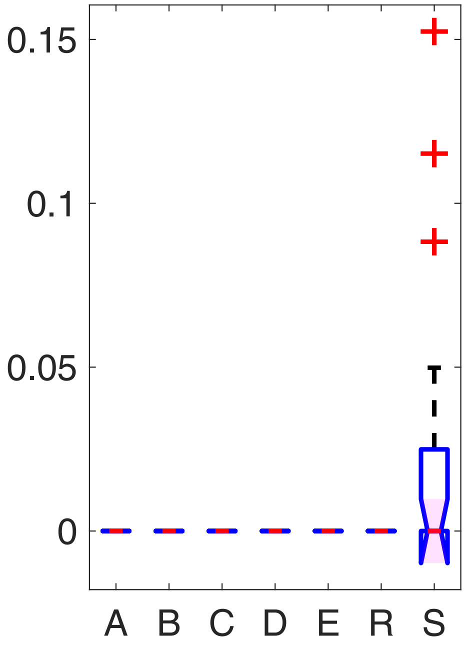


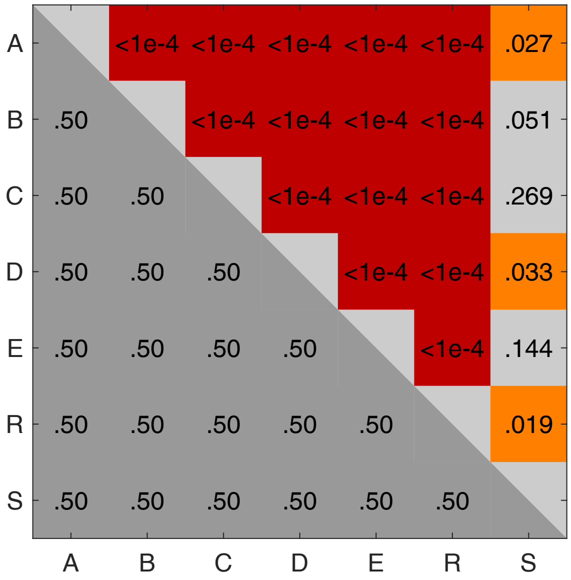


**Heatmap Analysis of Box 15**

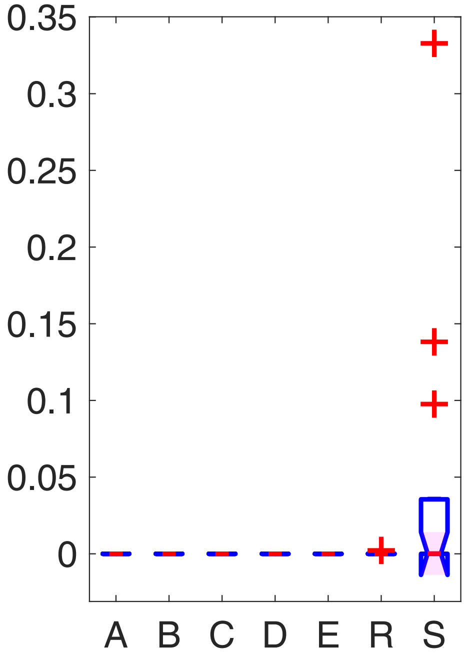


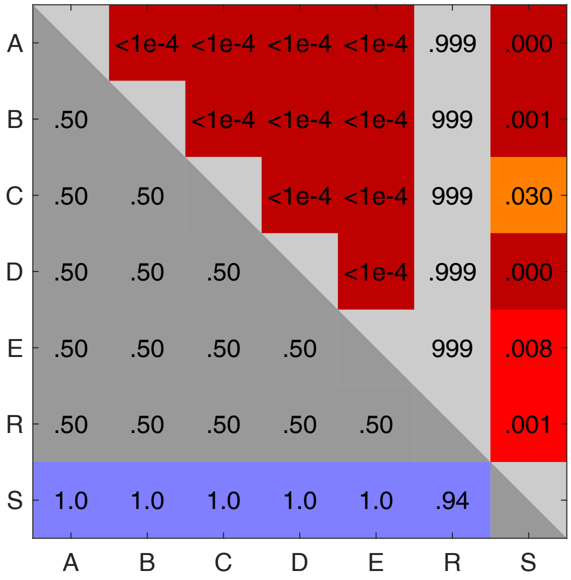

**Heatmap Analysis of Box 16**

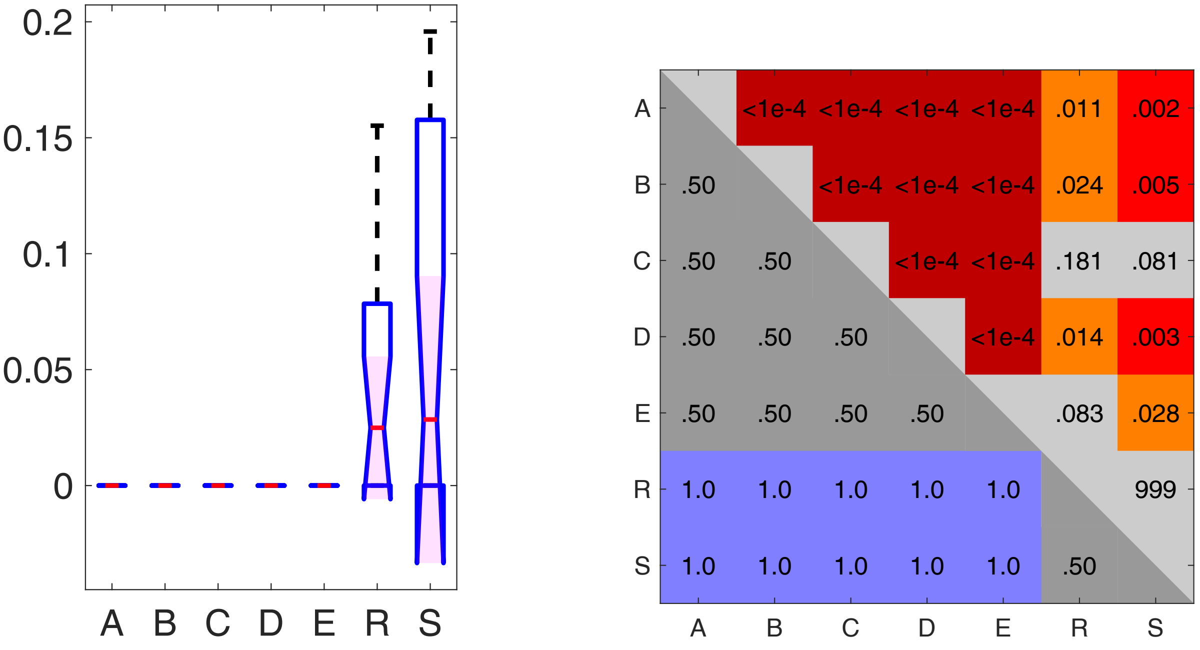


**Heatmap Analysis of Box 17**

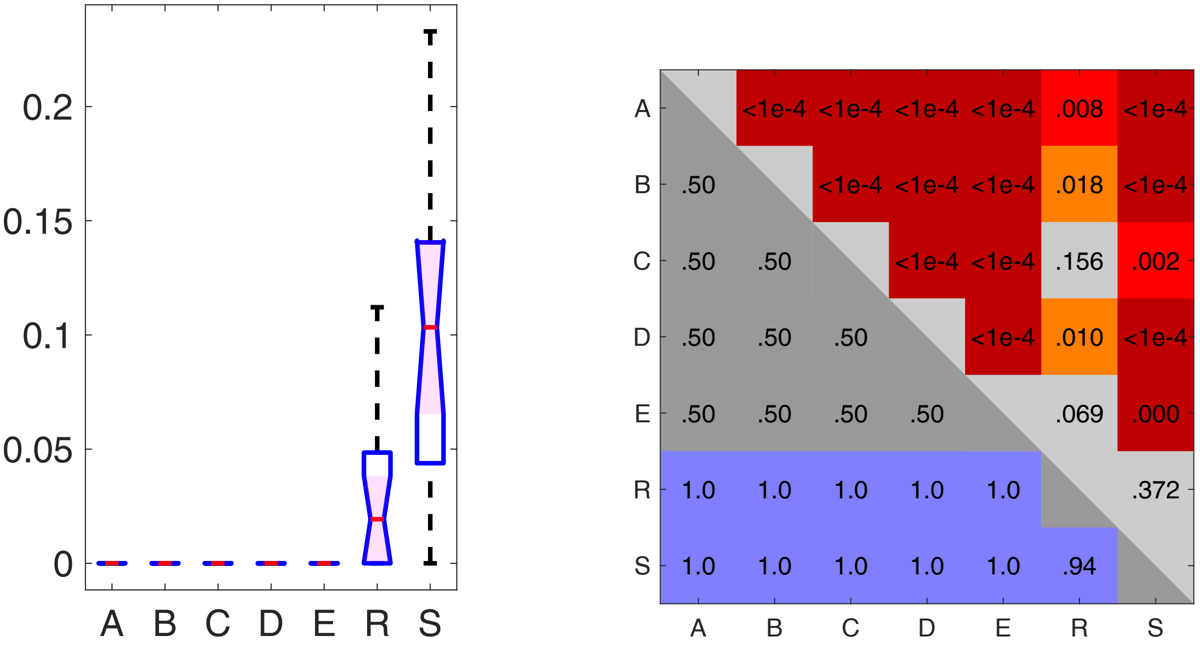

**Heatmap Analysis of Box 18**

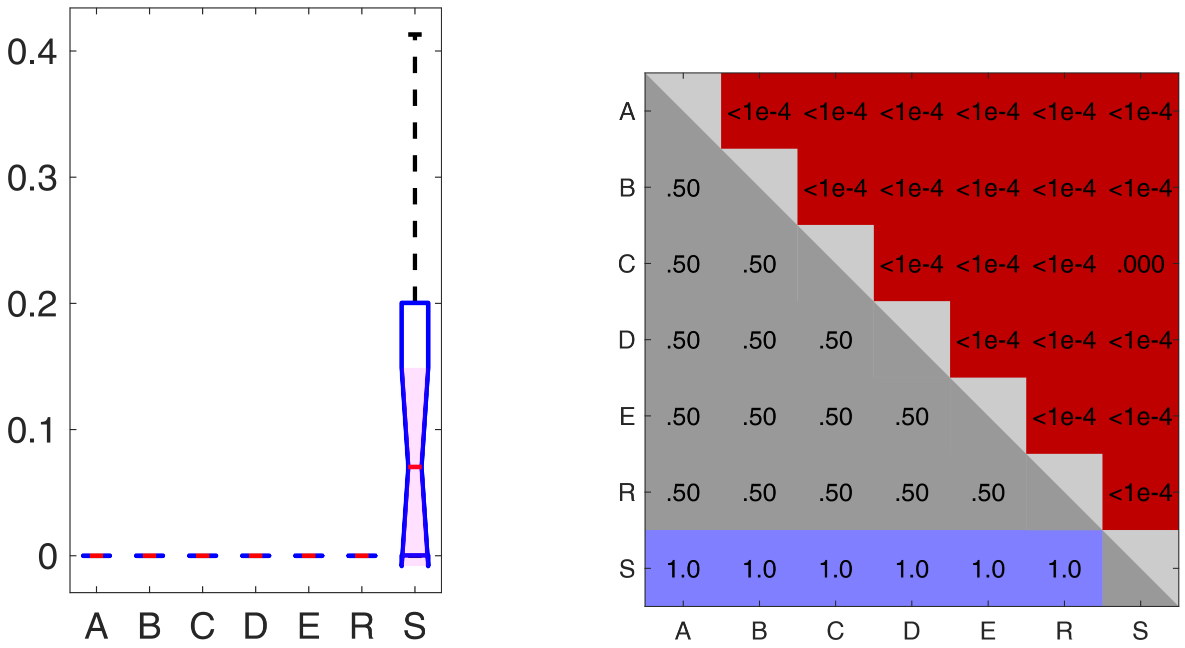


**Heatmap Analysis of Box 19**

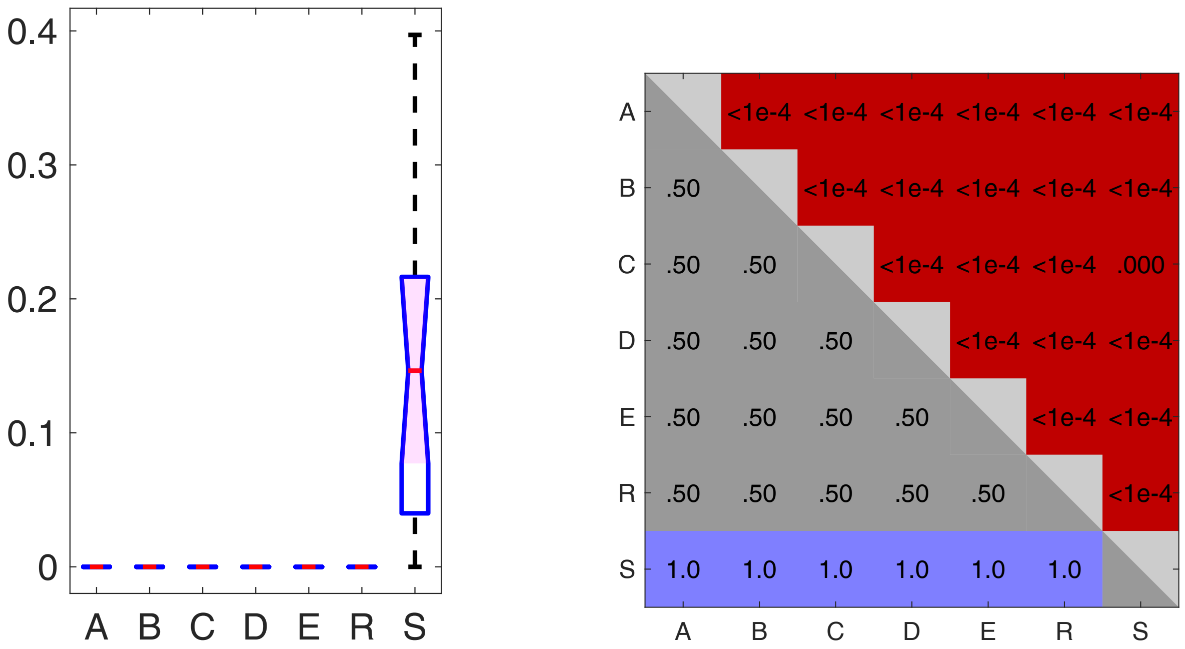

**Heatmap Analysis of Box 1A**

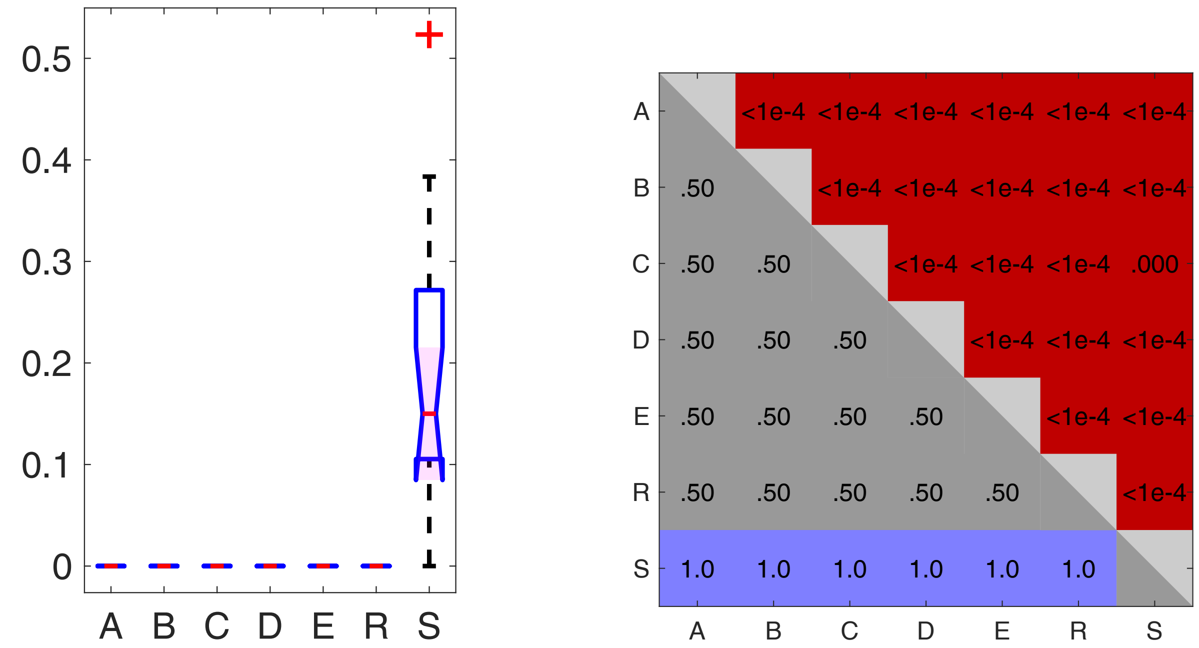


**Heatmap Analysis of Box 1B**

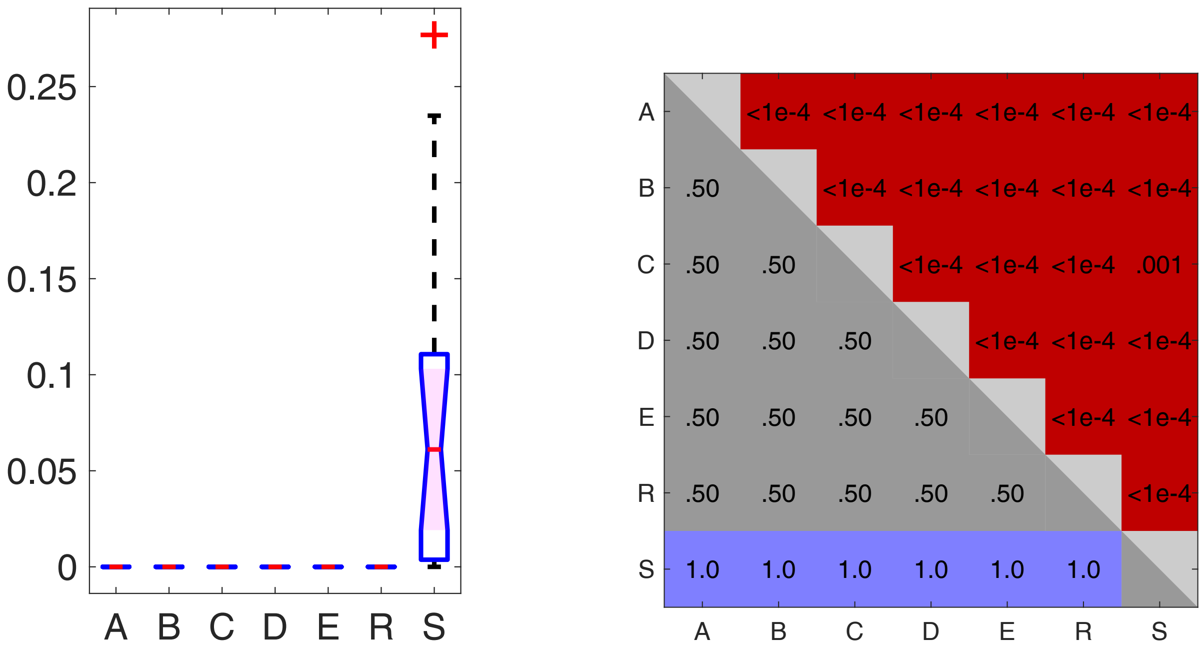

**Heatmap Analysis of Box 1C**

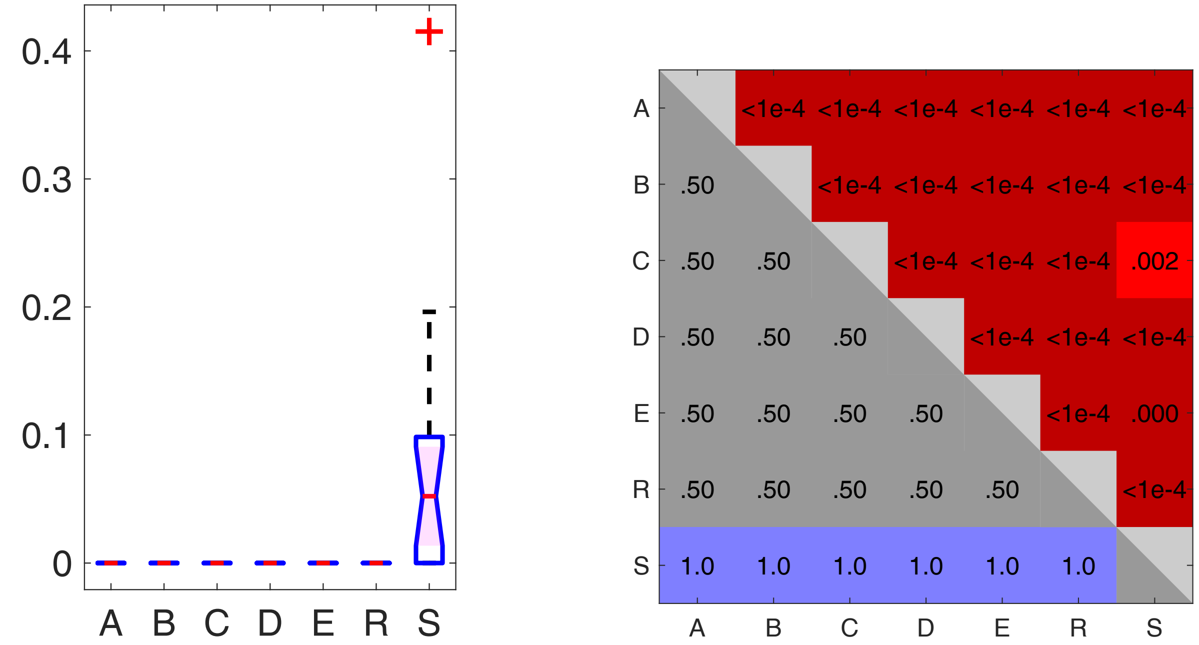


**Heatmap Analysis of Box 1D**

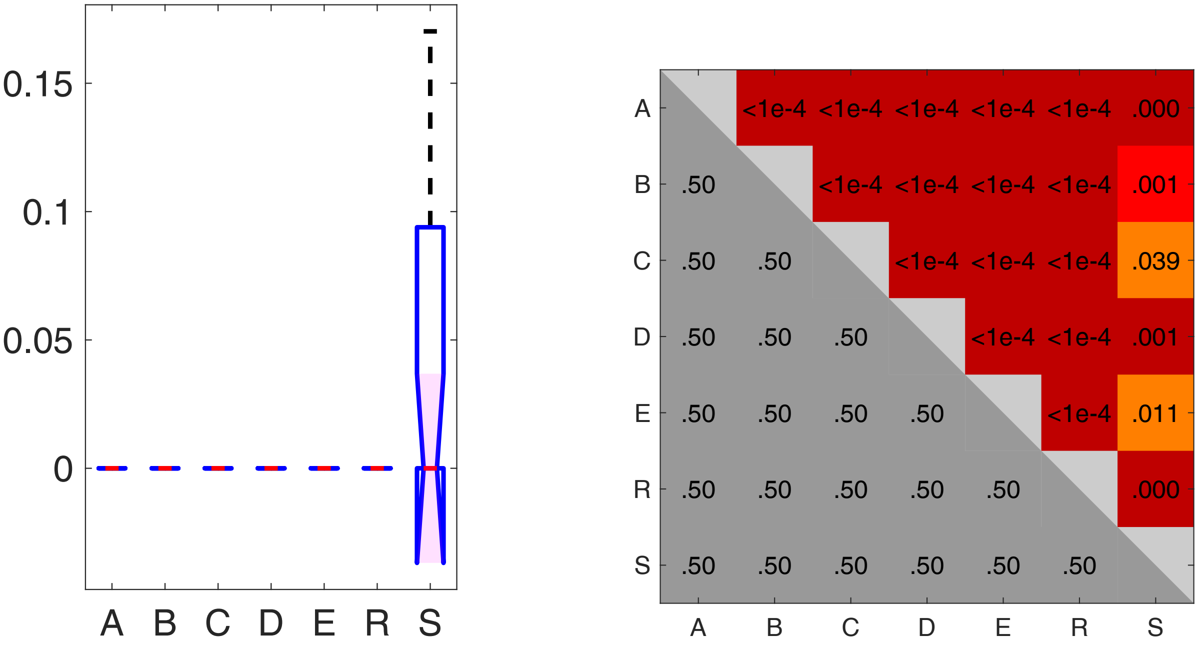

**Heatmap Analysis of Box 1E**

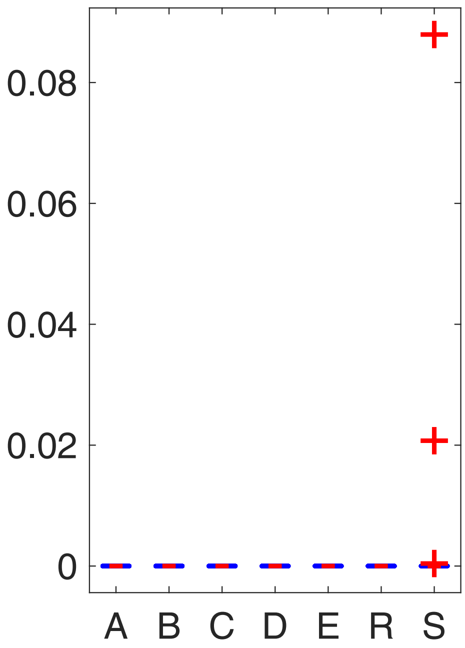


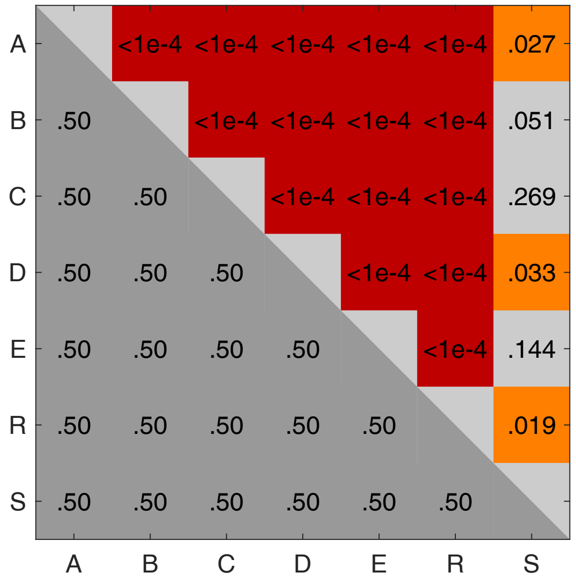


**Heatmap Analysis of Box 1F**

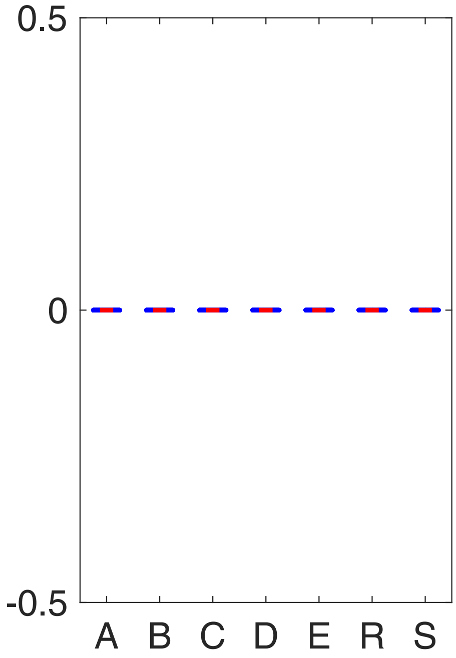


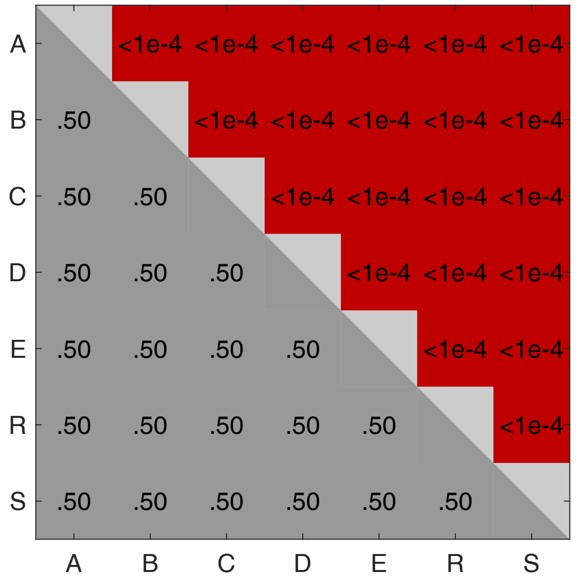

**Heatmap Analysis of Box 1G**

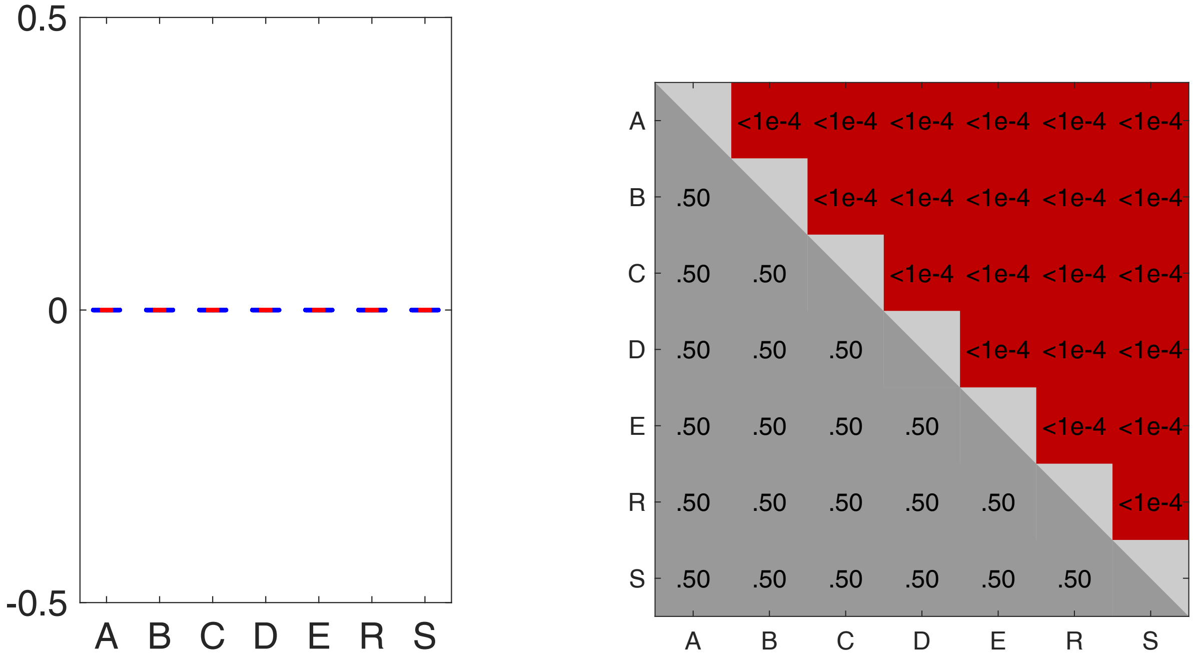


**Heatmap Analysis of Box 1H**

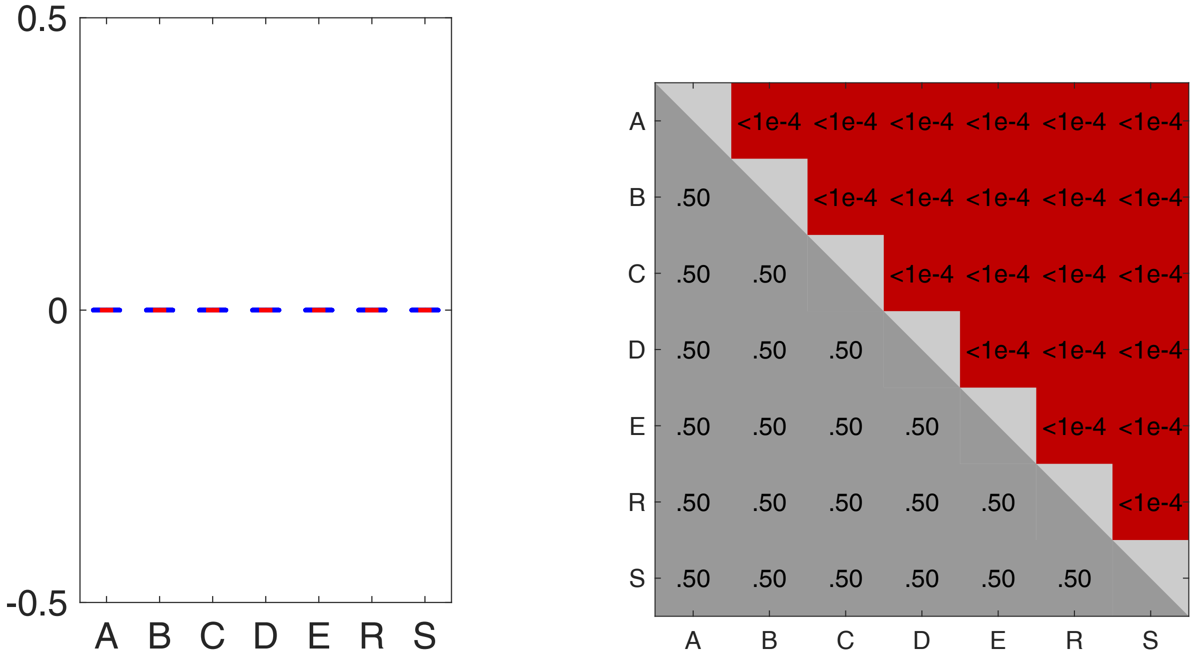

**Heatmap Analysis of Box 20**

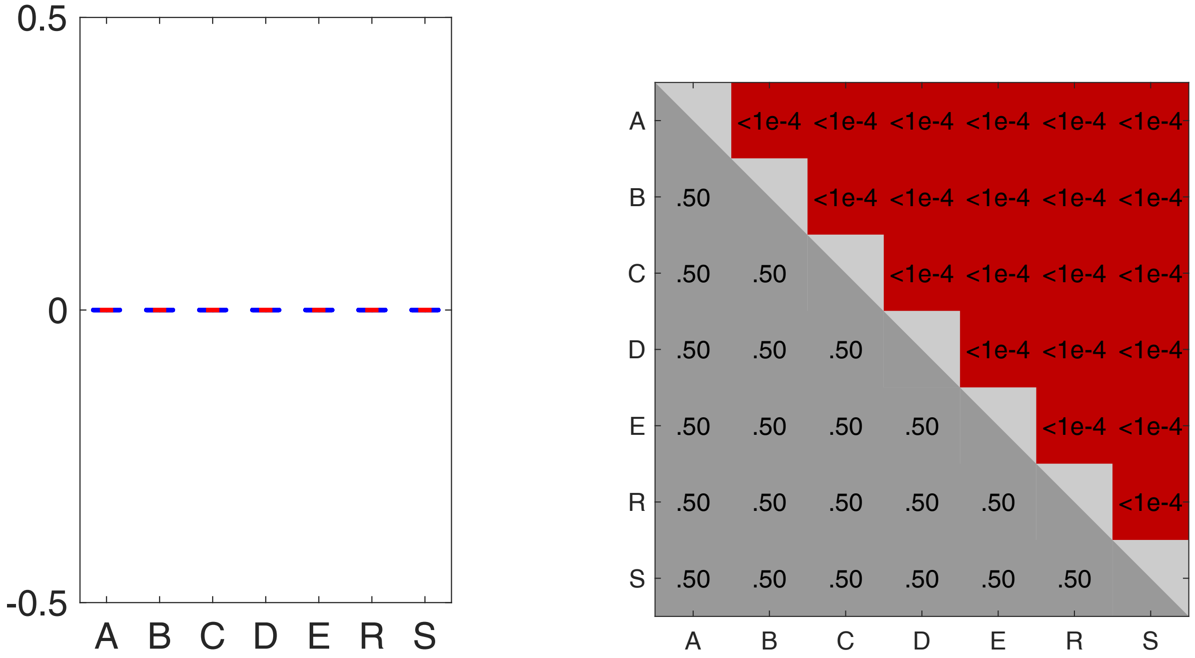


**Heatmap Analysis of Box 21**

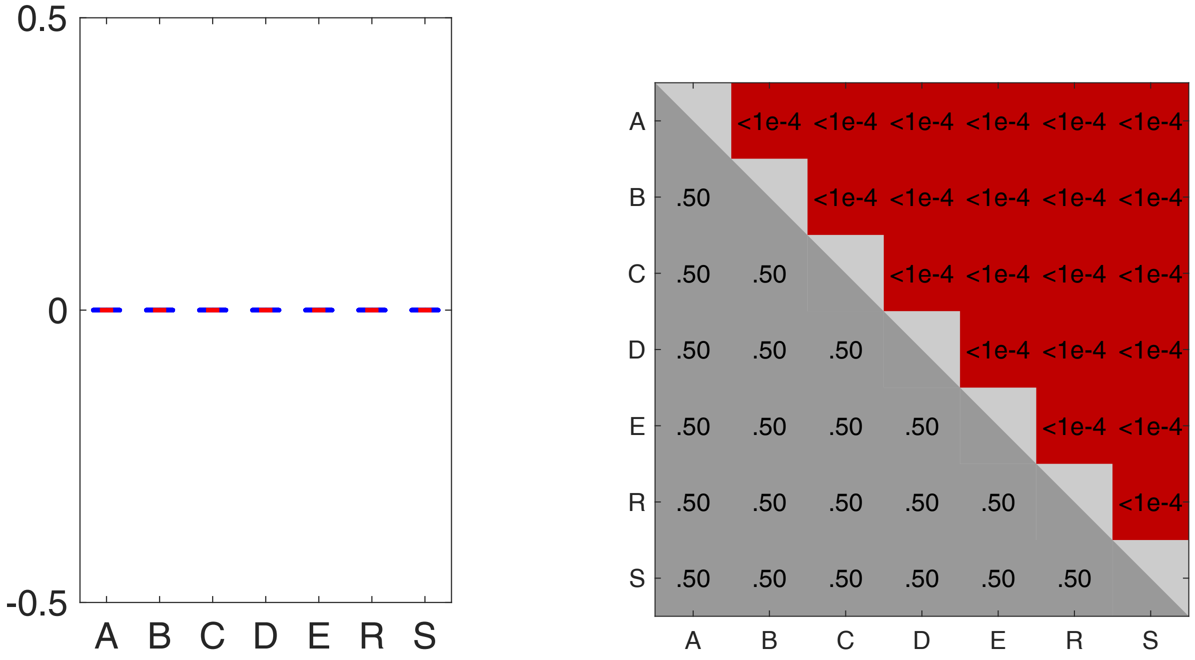

**Heatmap Analysis of Box 22**

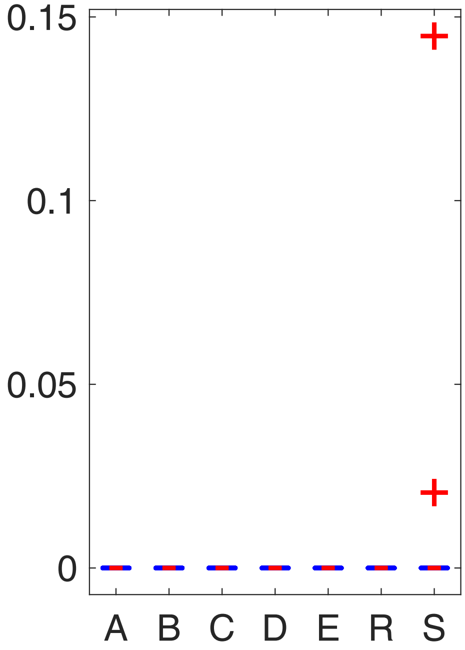


| | A | B | C | D | E | R | S |
|---|---|---|---|---|---|---|---|
| A | | <1e-4 | <1e-4 | <1e-4 | <1e-4 | <1e-4 | .295 |
| B | .50 | | <1e-4 | <1e-4 | <1e-4 | <1e-4 | .379 |
| C | .50 | .50 | | <1e-4 | <1e-4 | <1e-4 | .700 |
| D | .50 | .50 | .50 | | <1e-4 | <1e-4 | .319 |
| E | .50 | .50 | .50 | .50 | | <1e-4 | .564 |
| R | .50 | .50 | .50 | .50 | .50 | | .255 |
| S | .50 | .50 | .50 | .50 | .50 | .50 | |

**Heatmap Analysis of Box 23**

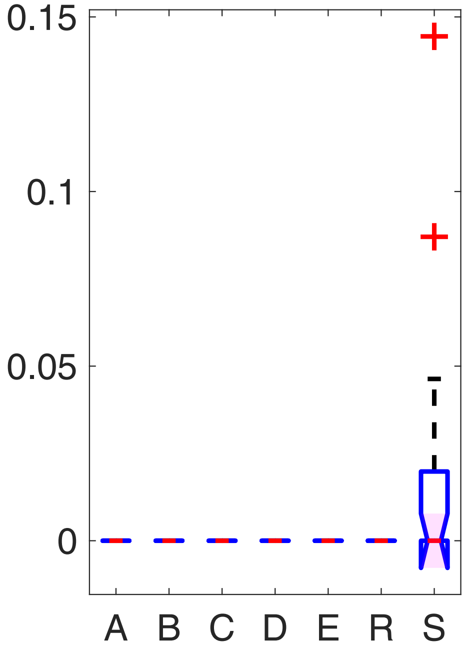


| | A | B | C | D | E | R | S |
|---|---|---|---|---|---|---|---|
| A | | <1e-4 | <1e-4 | <1e-4 | <1e-4 | <1e-4 | .002 |
| B | .50 | | <1e-4 | <1e-4 | <1e-4 | <1e-4 | .005 |
| C | .50 | .50 | | <1e-4 | <1e-4 | <1e-4 | .077 |
| D | .50 | .50 | .50 | | <1e-4 | <1e-4 | .002 |
| E | .50 | .50 | .50 | .50 | | <1e-4 | .027 |
| R | .50 | .50 | .50 | .50 | .50 | | .001 |
| S | .50 | .50 | .50 | .50 | .50 | .50 | |

**Heatmap Analysis of Box 24**

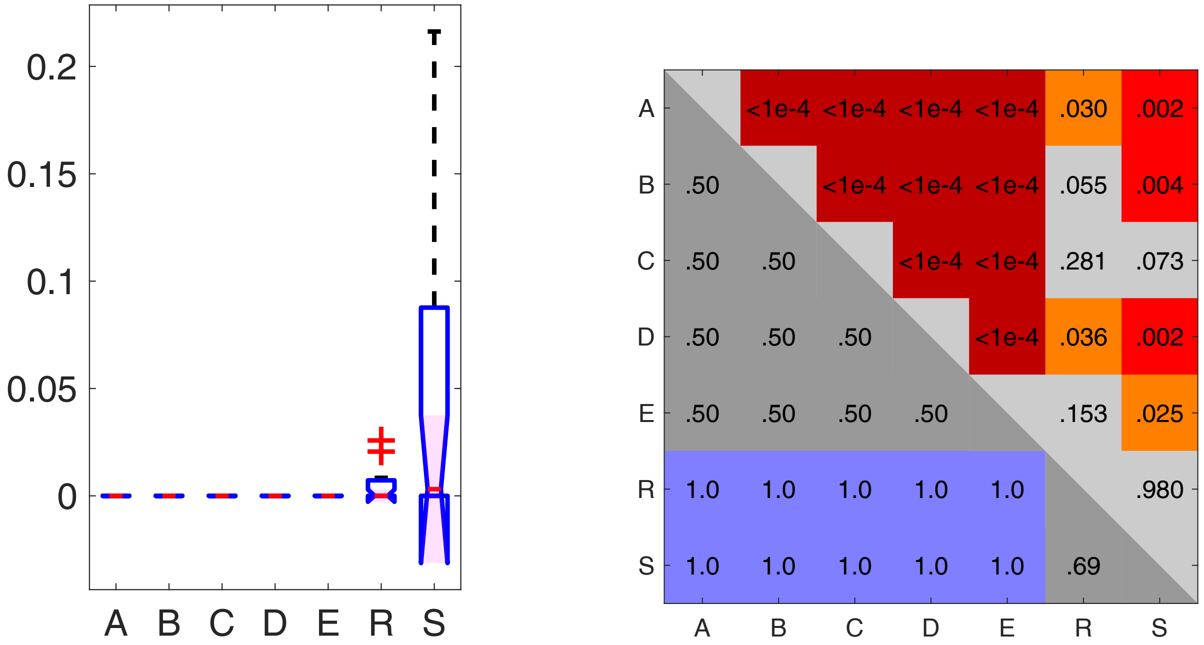


**Heatmap Analysis of Box 25**

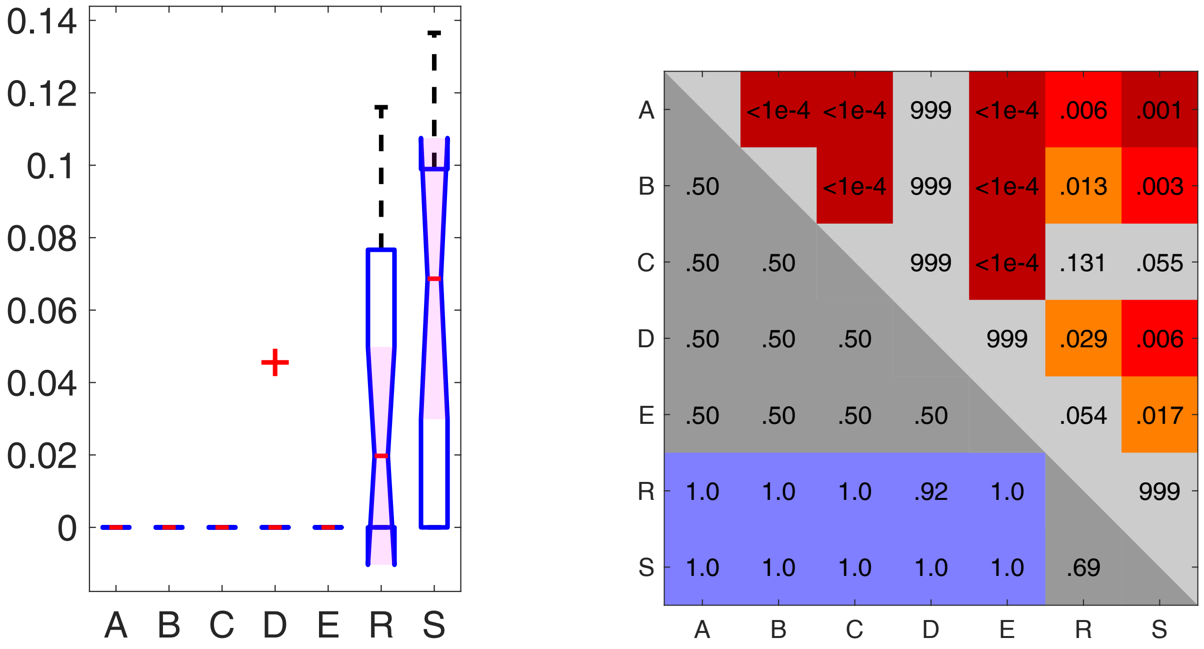

**Heatmap Analysis of Box 26**

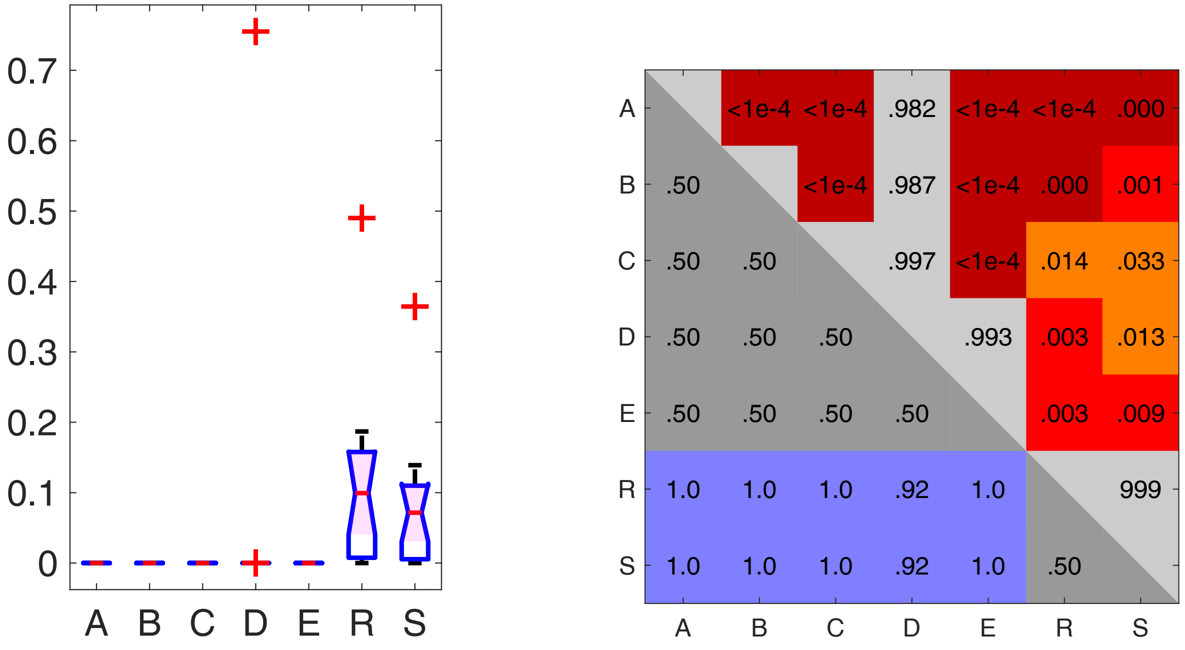


**Heatmap Analysis of Box 27**

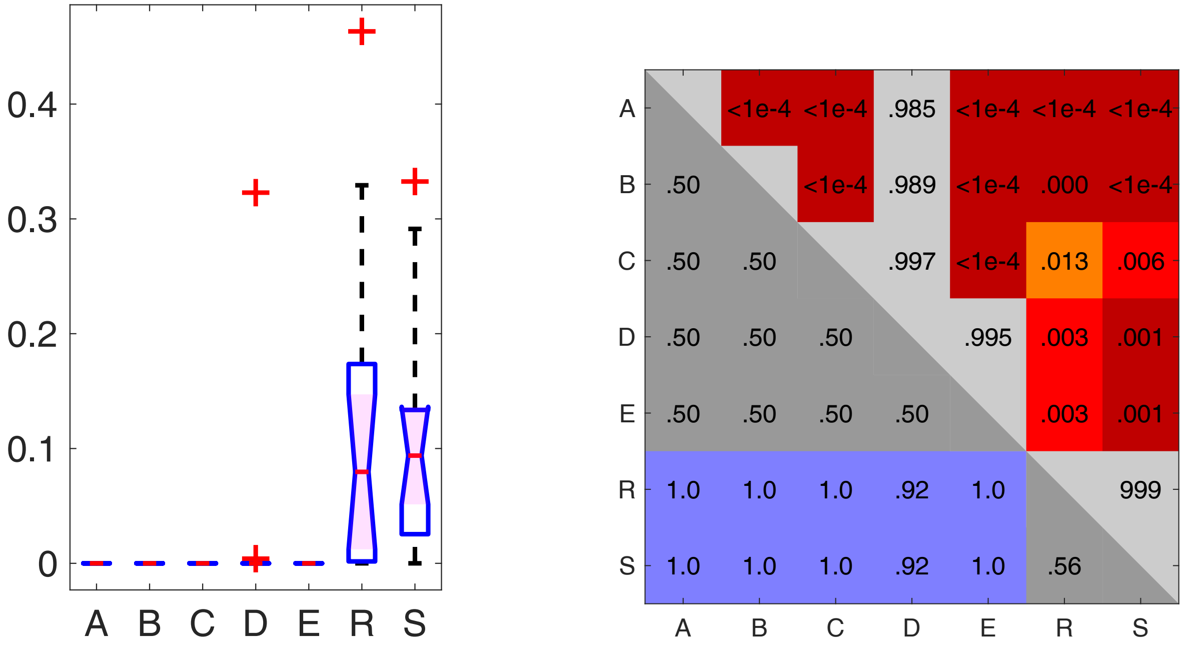

**Heatmap Analysis of Box 28**

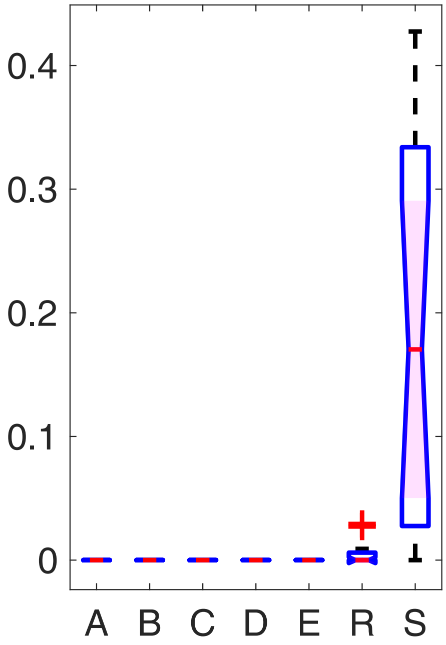


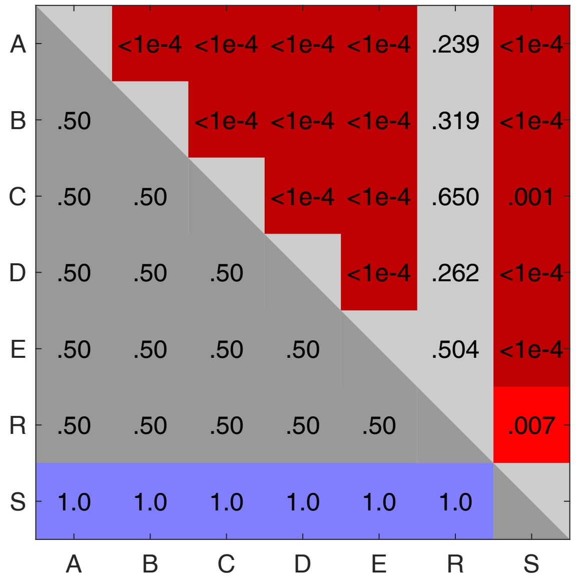


**Heatmap Analysis of Box 29**

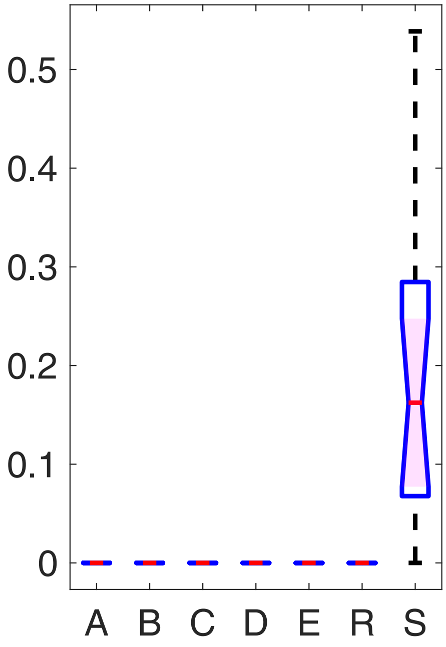


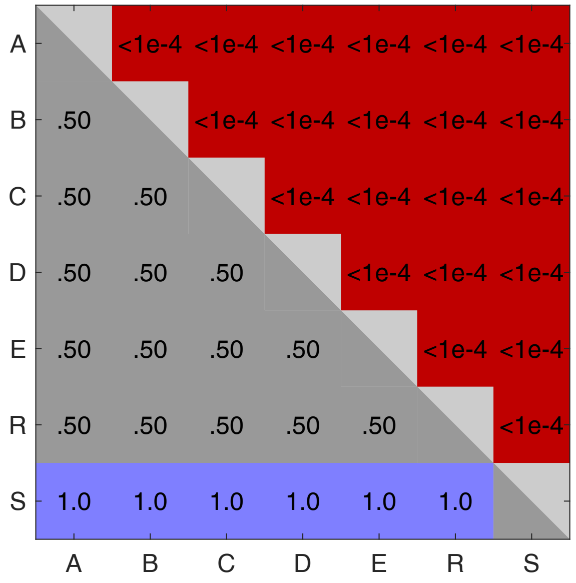

**Heatmap Analysis of Box 2A**

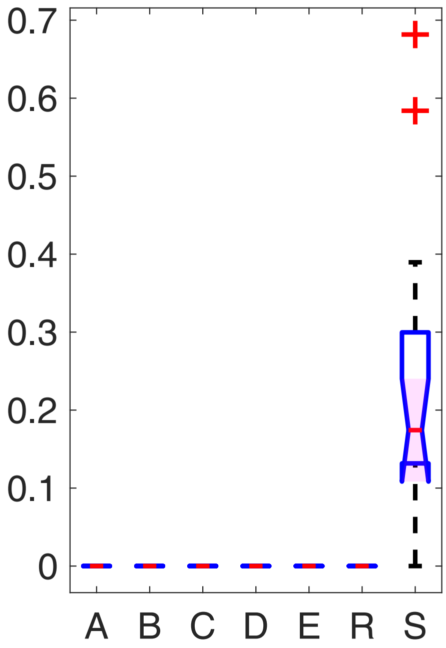


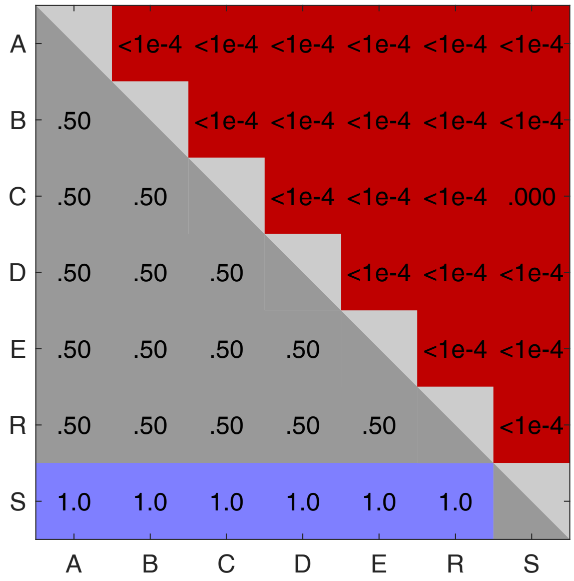


**Heatmap Analysis of Box 2B**

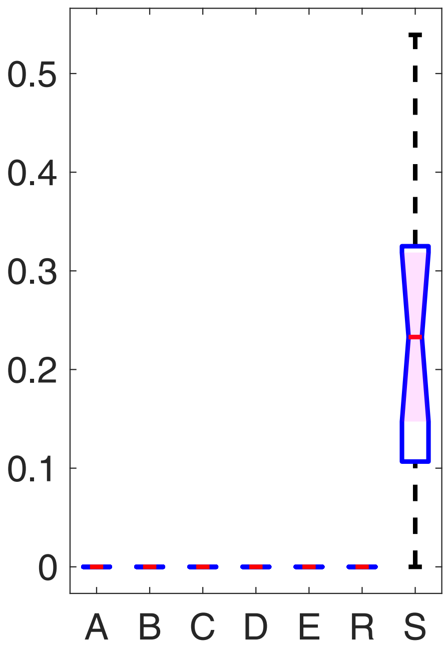


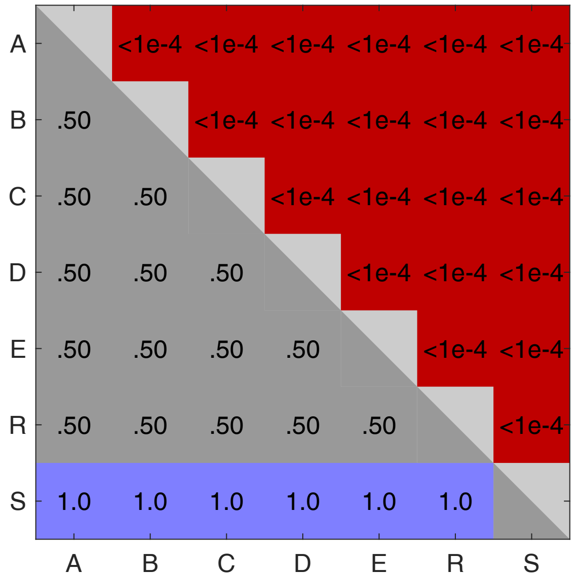

**Heatmap Analysis of Box 2C**

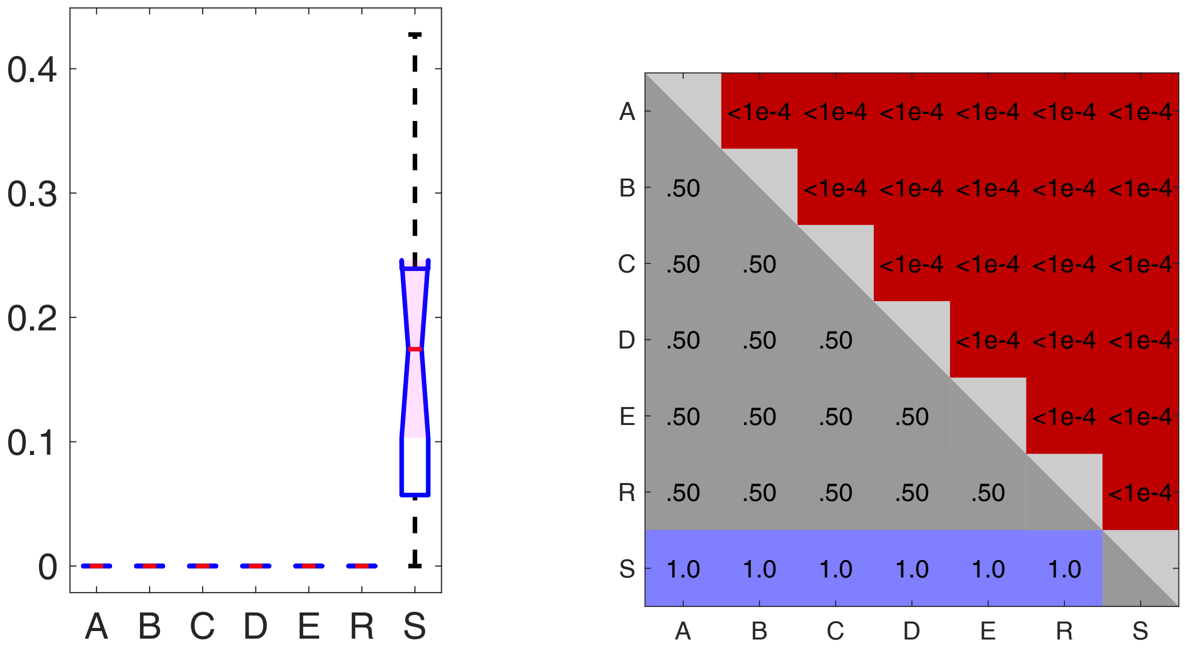


**Heatmap Analysis of Box 2D**

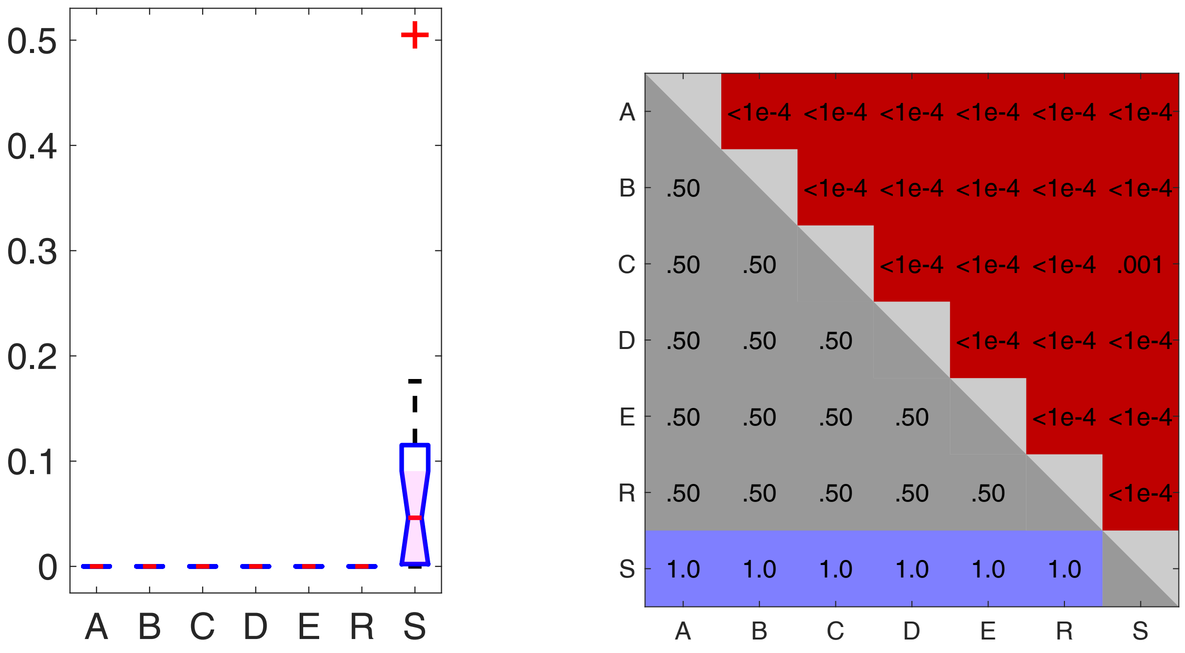

**Heatmap Analysis of Box 2E**

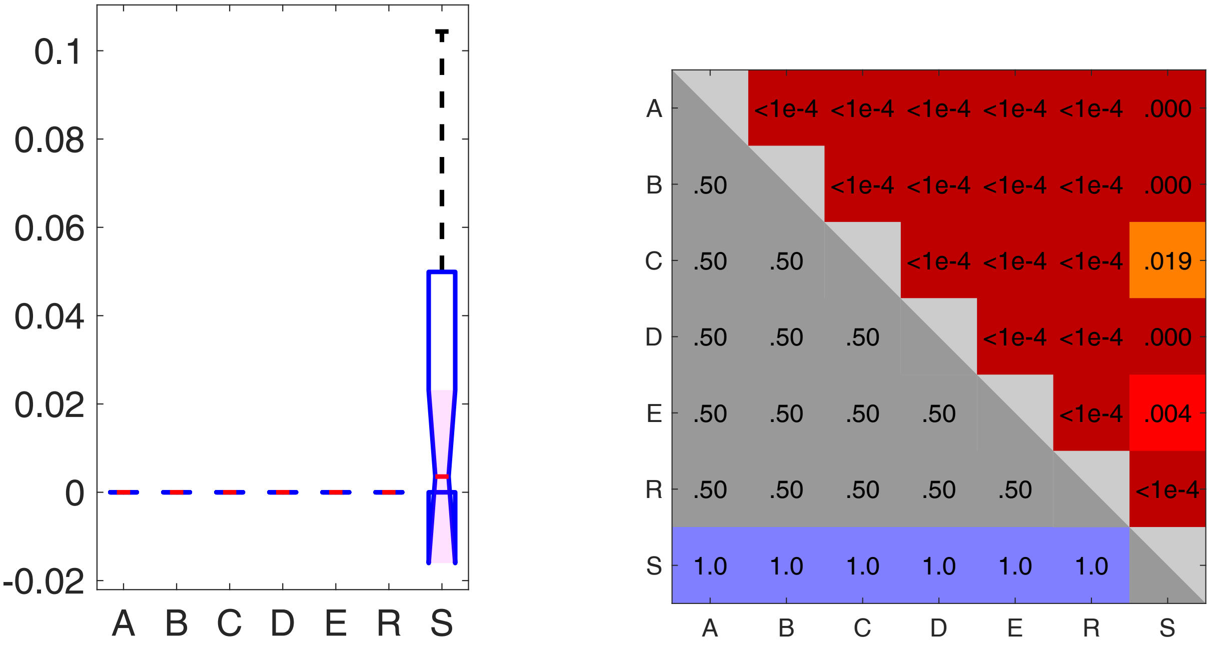


**Heatmap Analysis of Box 2F**

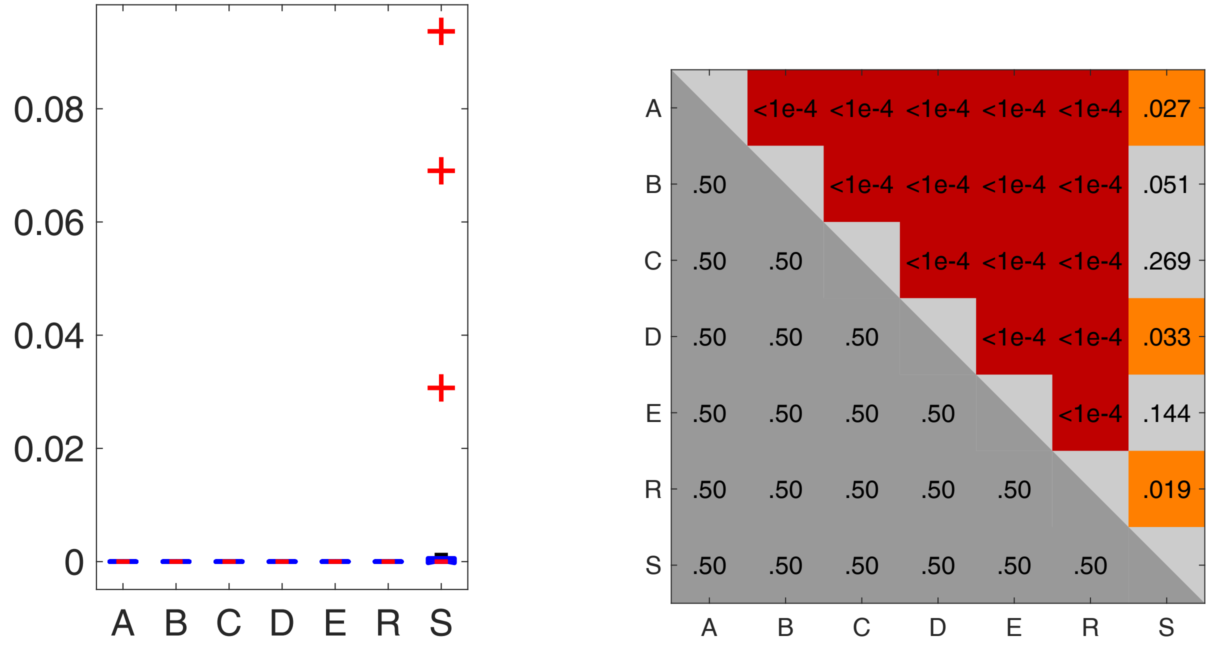

**Heatmap Analysis of Box 2G**

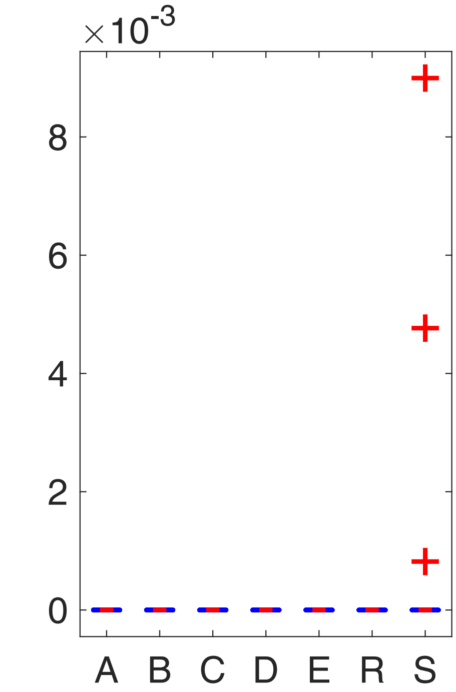


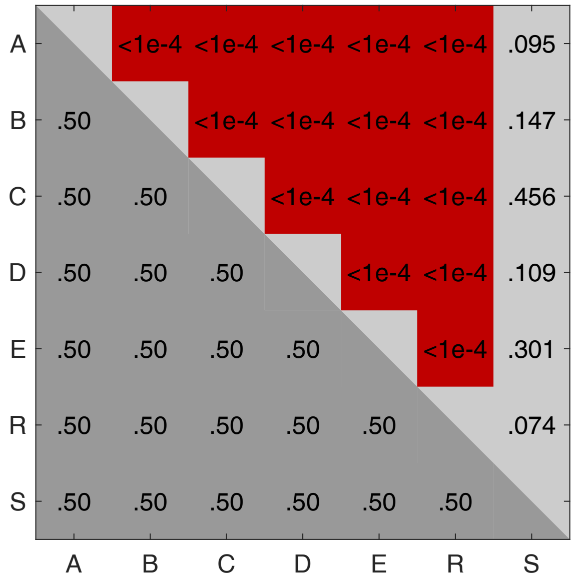


**Heatmap Analysis of Box 2H**

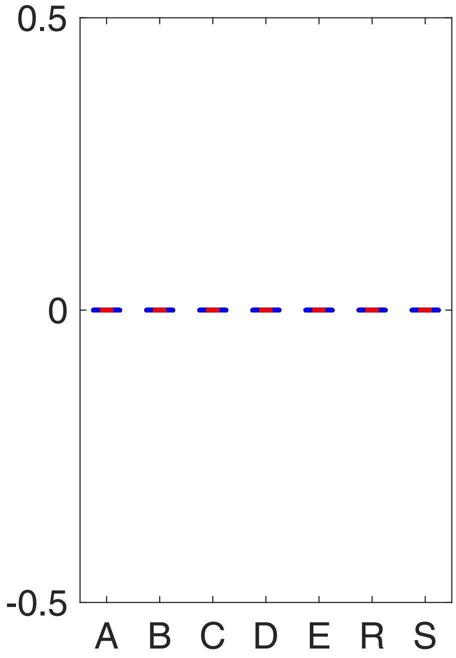


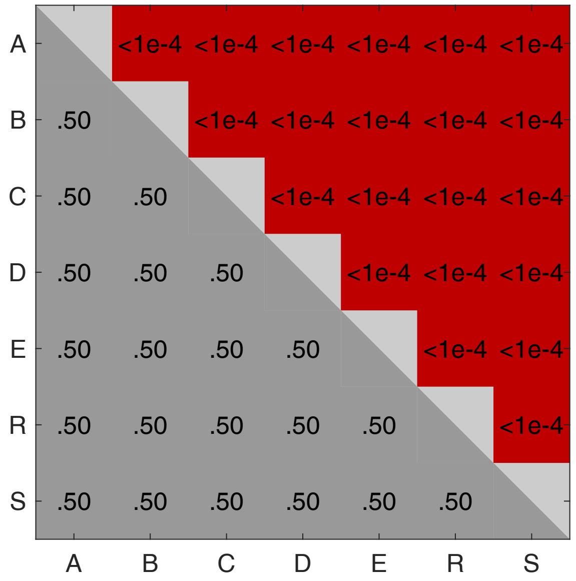

**Heatmap Analysis of Box 30**

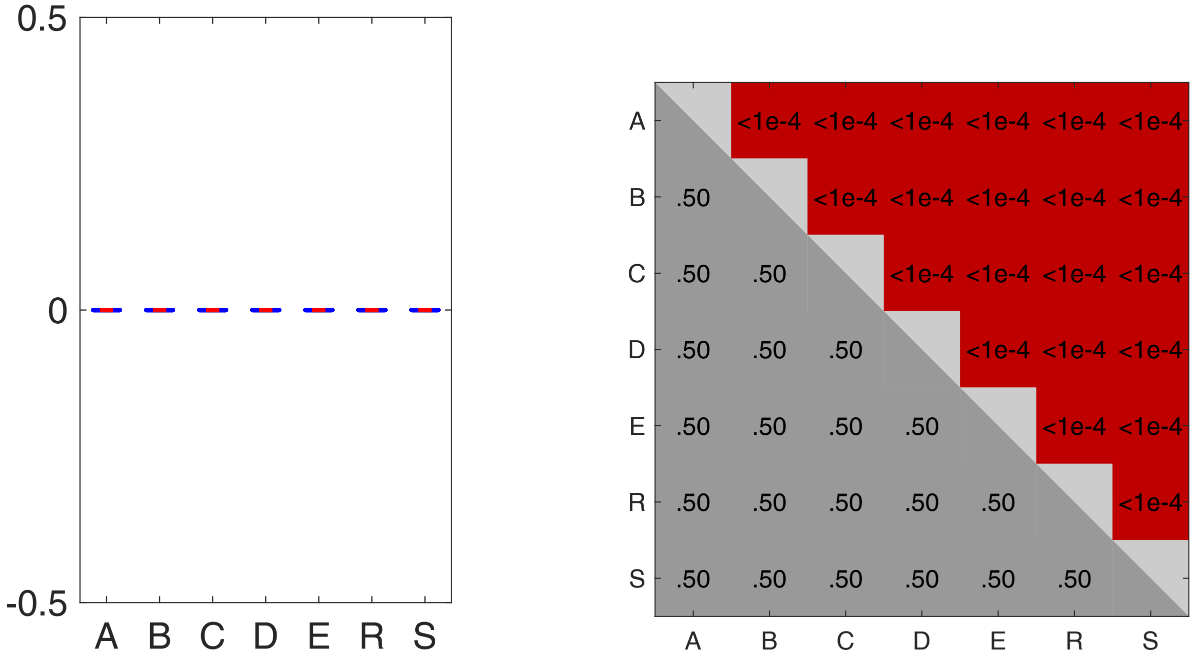


**Heatmap Analysis of Box 31**

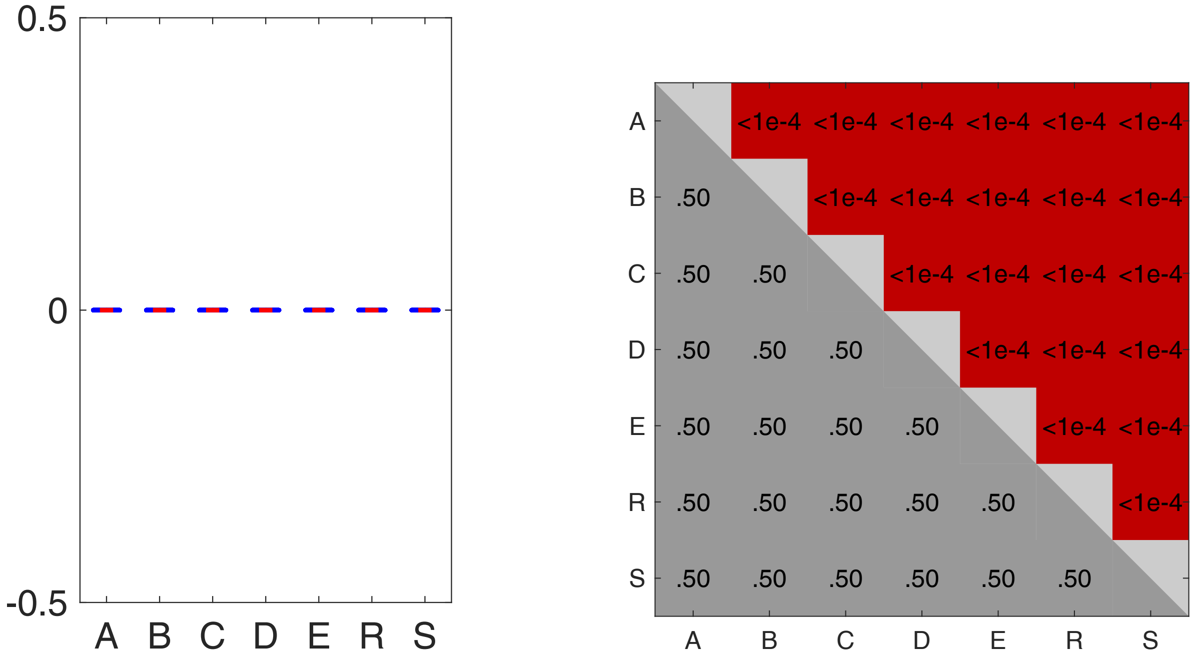

**Heatmap Analysis of Box 32**

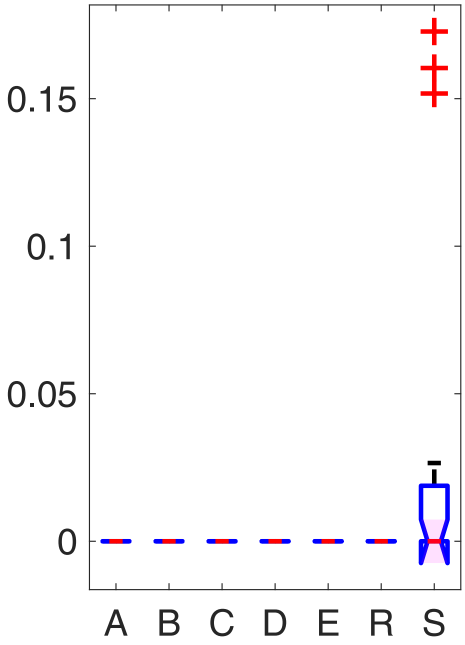


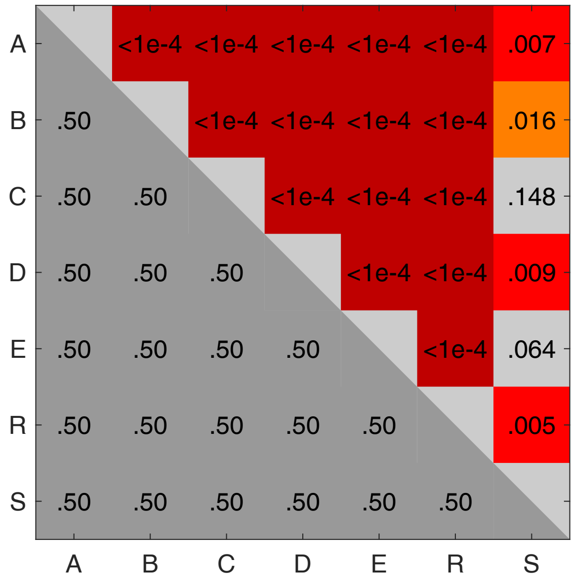


**Heatmap Analysis of Box 33**

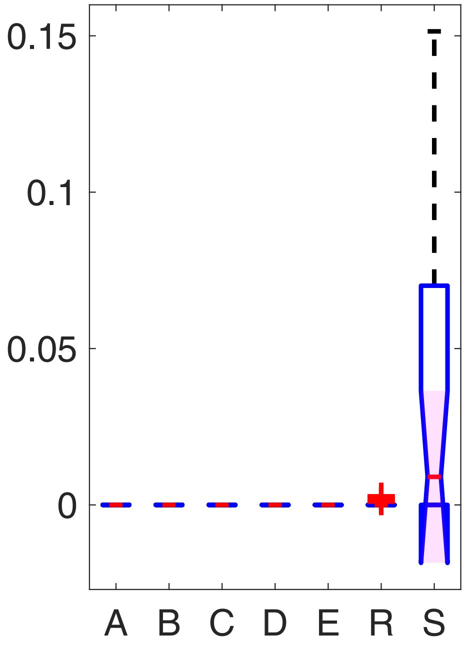


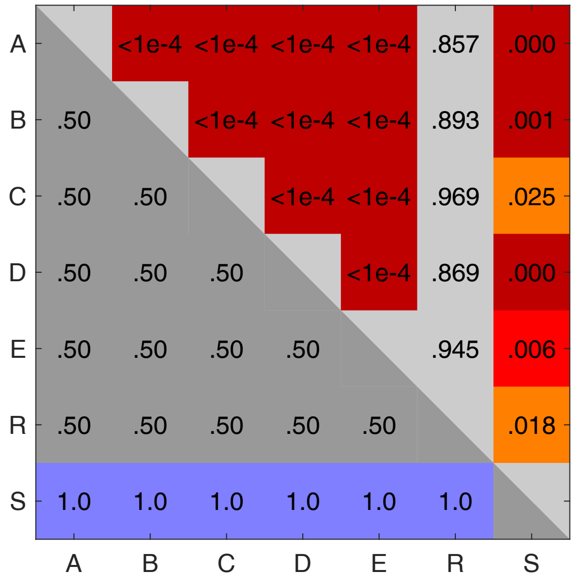

**Heatmap Analysis of Box 34**

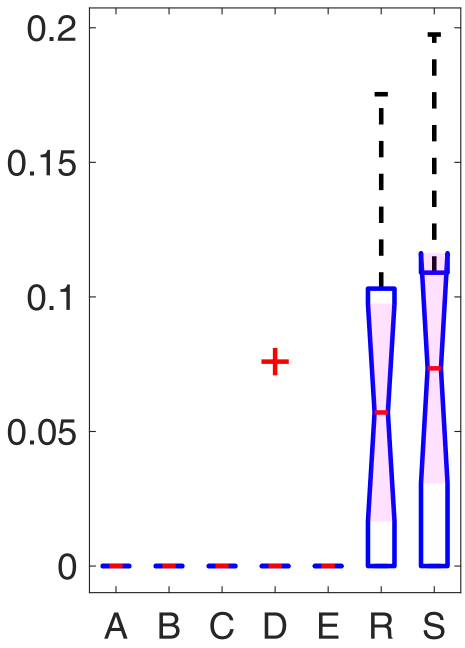


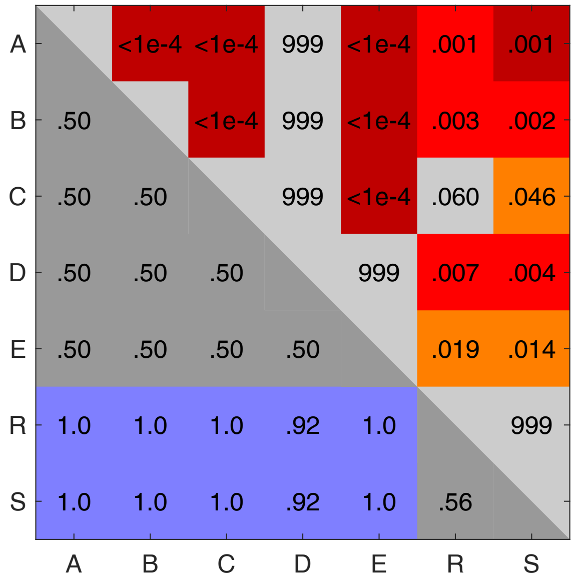


**Heatmap Analysis of Box 35**

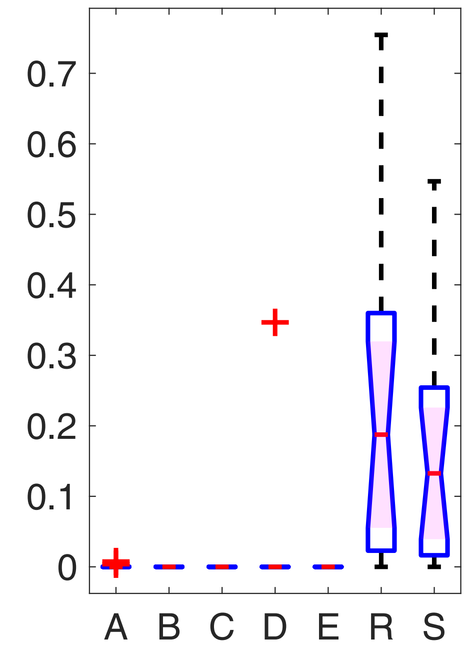


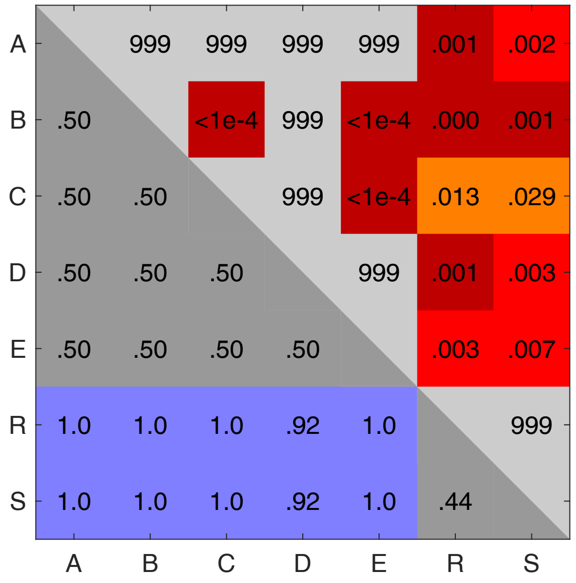

**Heatmap Analysis of Box 36**

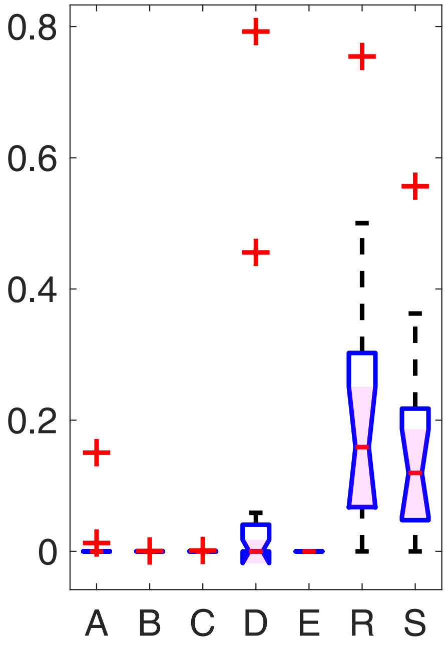


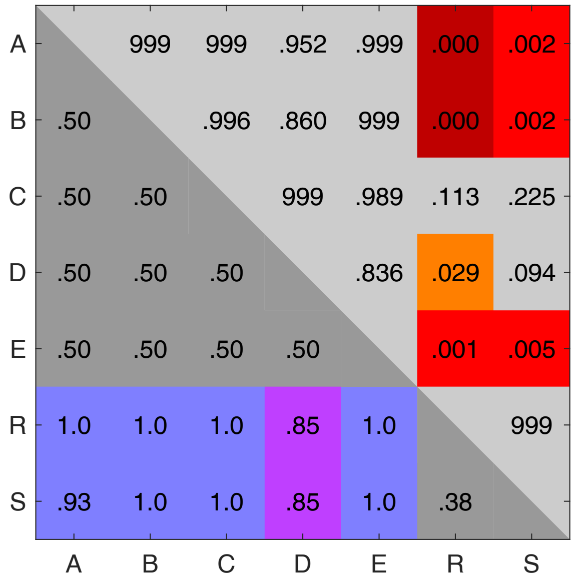


**Heatmap Analysis of Box 37**

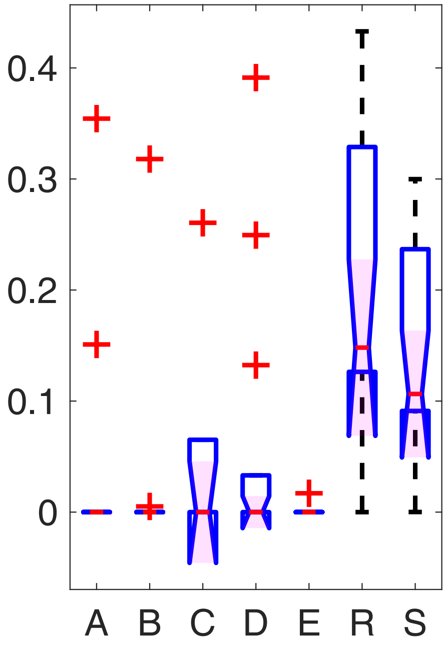


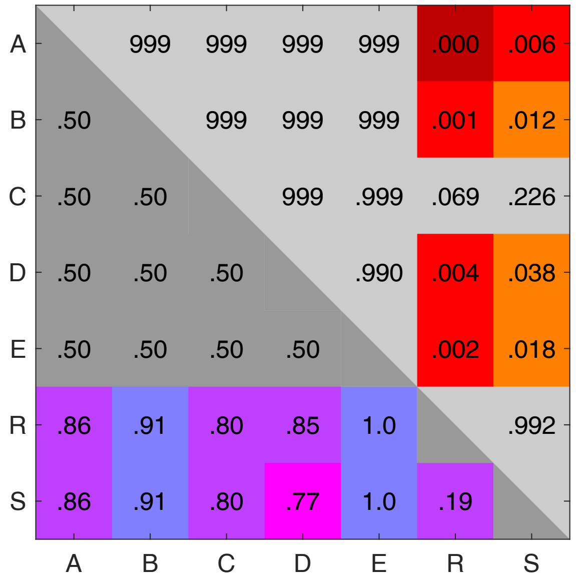

**Heatmap Analysis of Box 38**

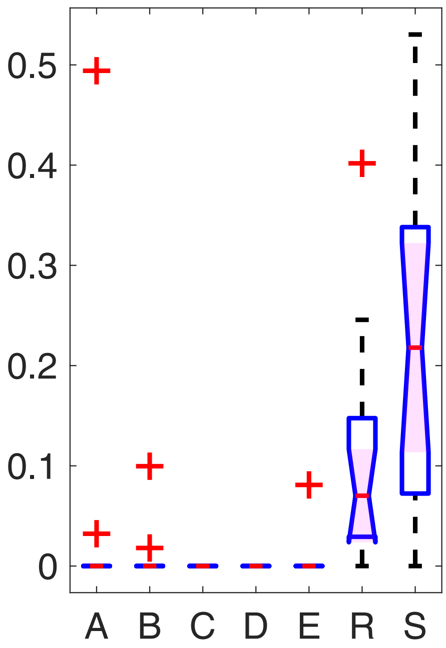


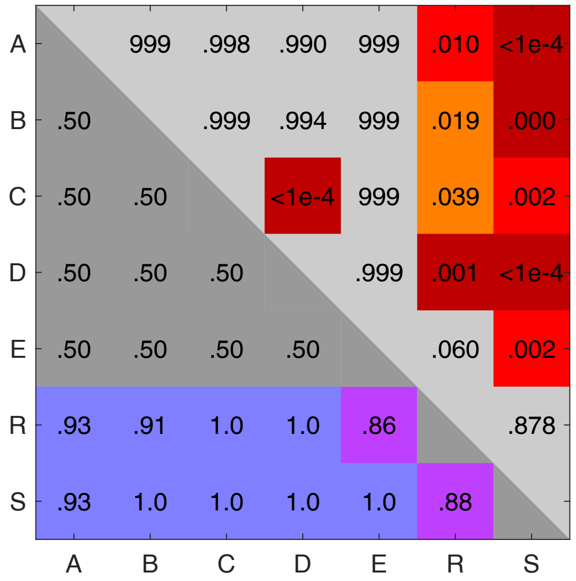


**Heatmap Analysis of Box 39**

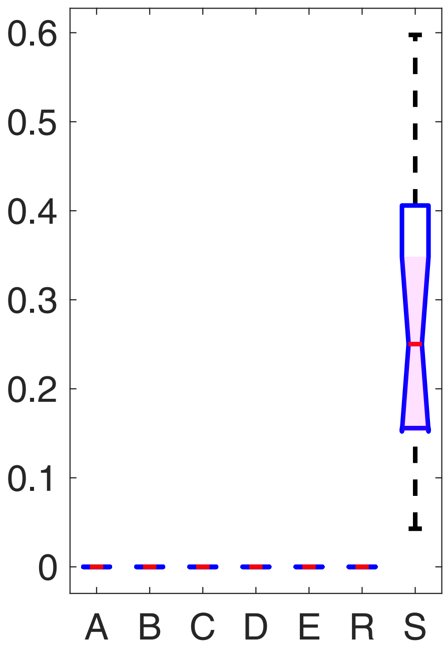


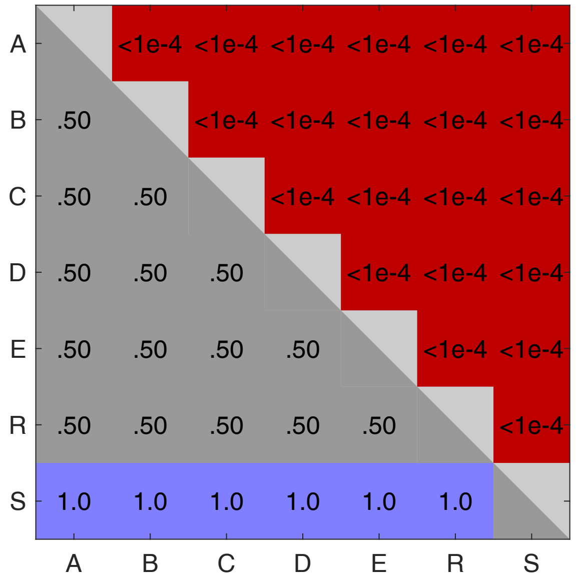

**Heatmap Analysis of Box 3A**

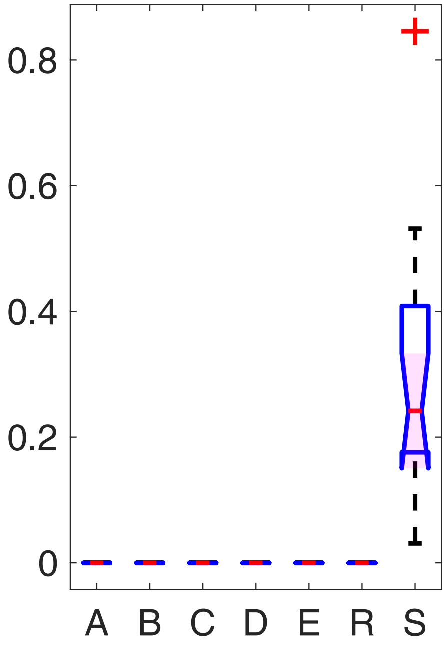


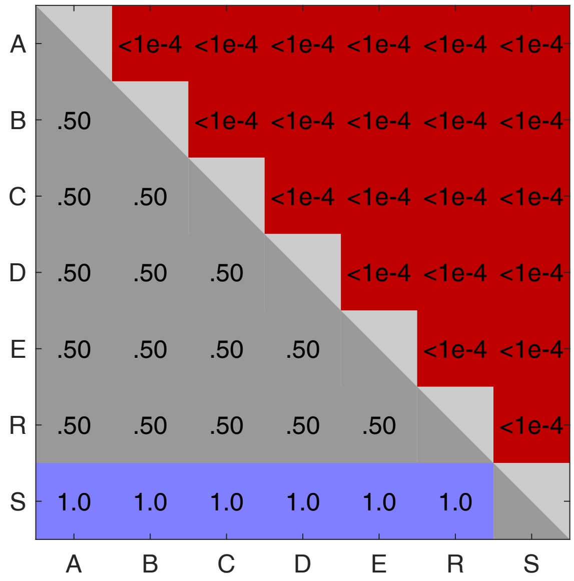


**Heatmap Analysis of Box 3B**

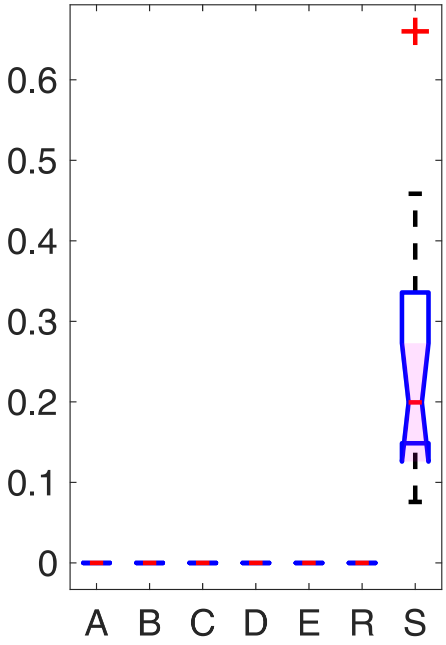


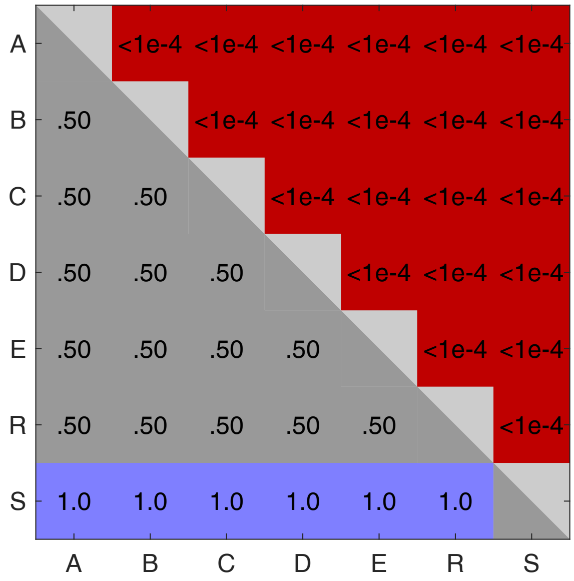

**Heatmap Analysis of Box 3C**

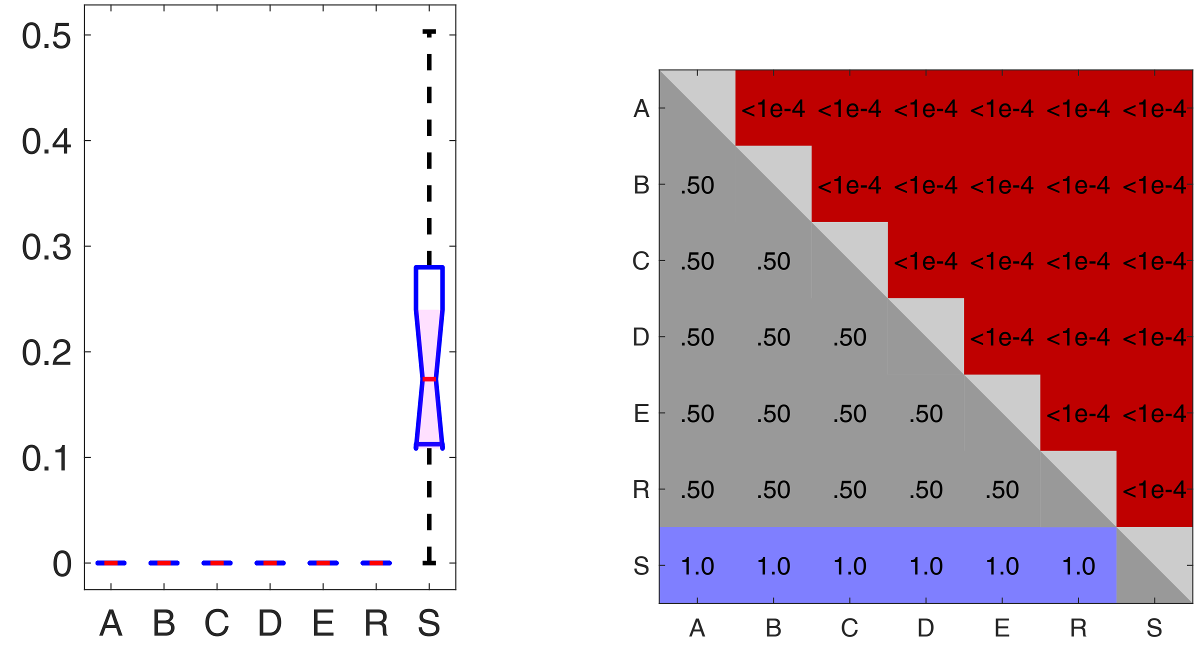


**Heatmap Analysis of Box 3D**

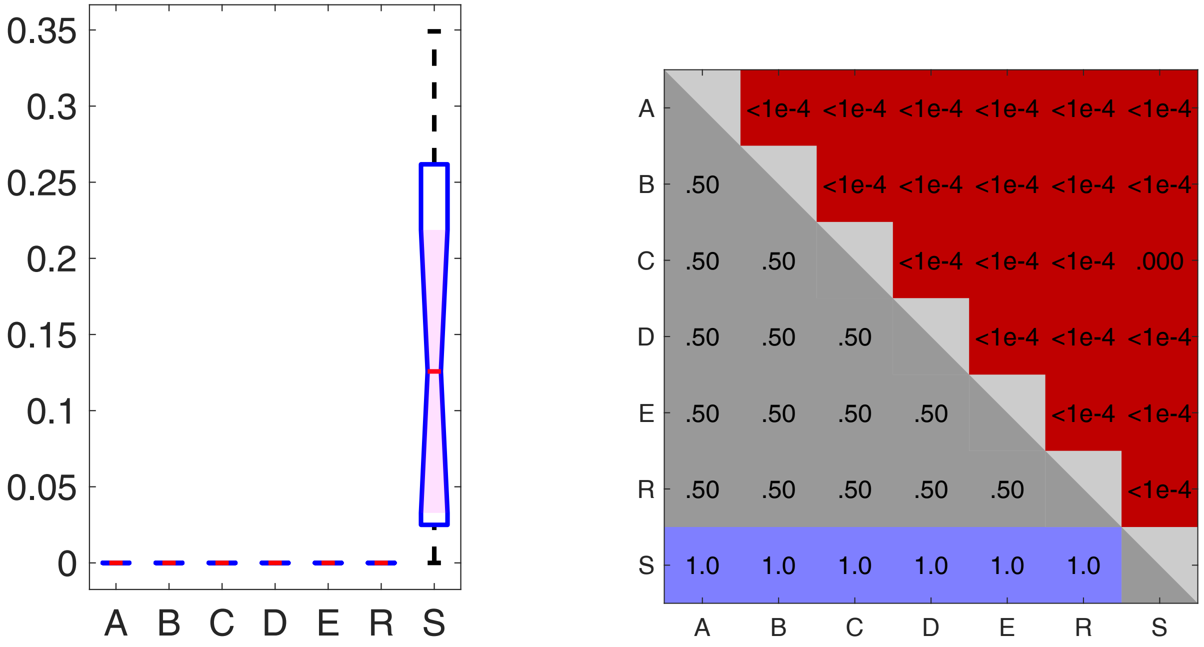

**Heatmap Analysis of Box 3E**

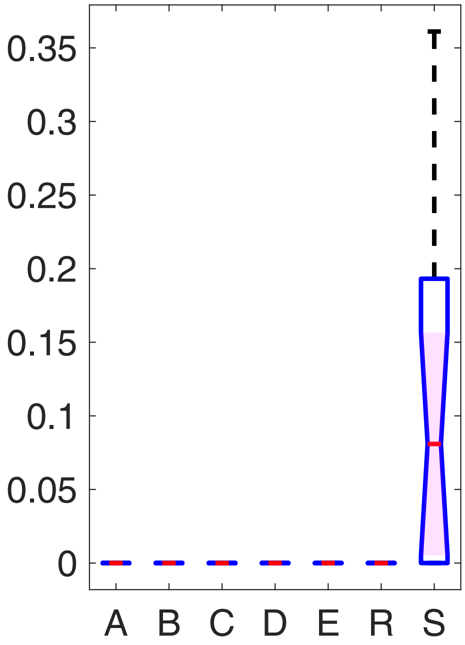


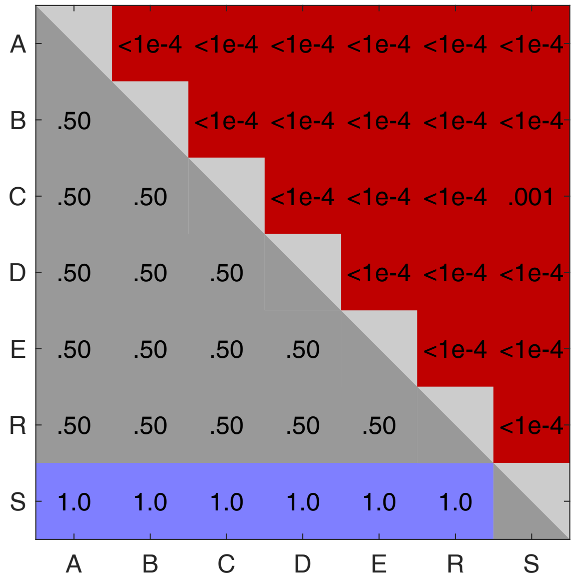


**Heatmap Analysis of Box 3F**

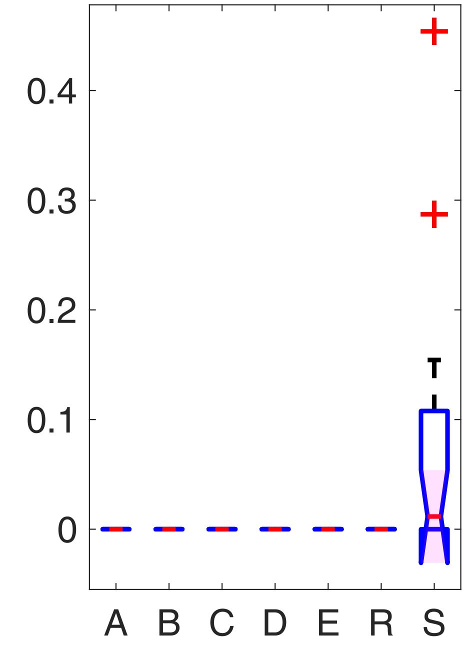


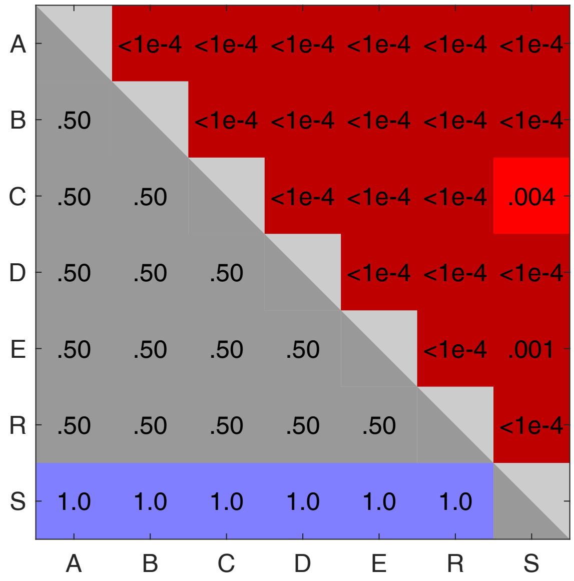

**Heatmap Analysis of Box 3G**

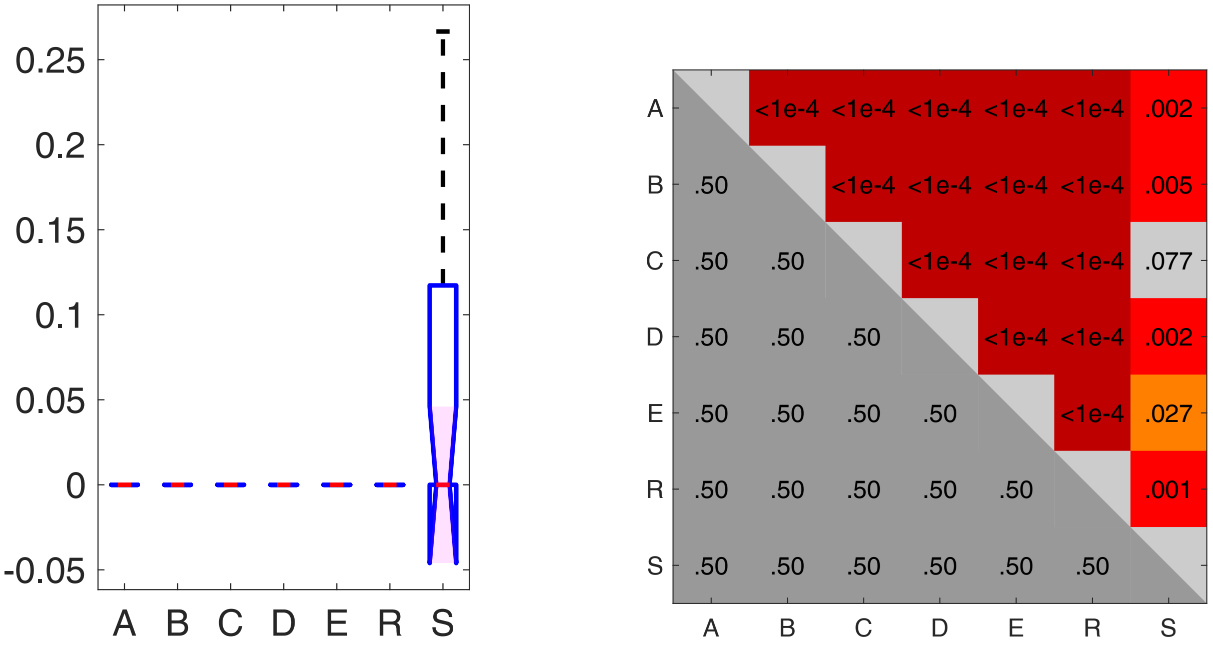


**Heatmap Analysis of Box 3H**

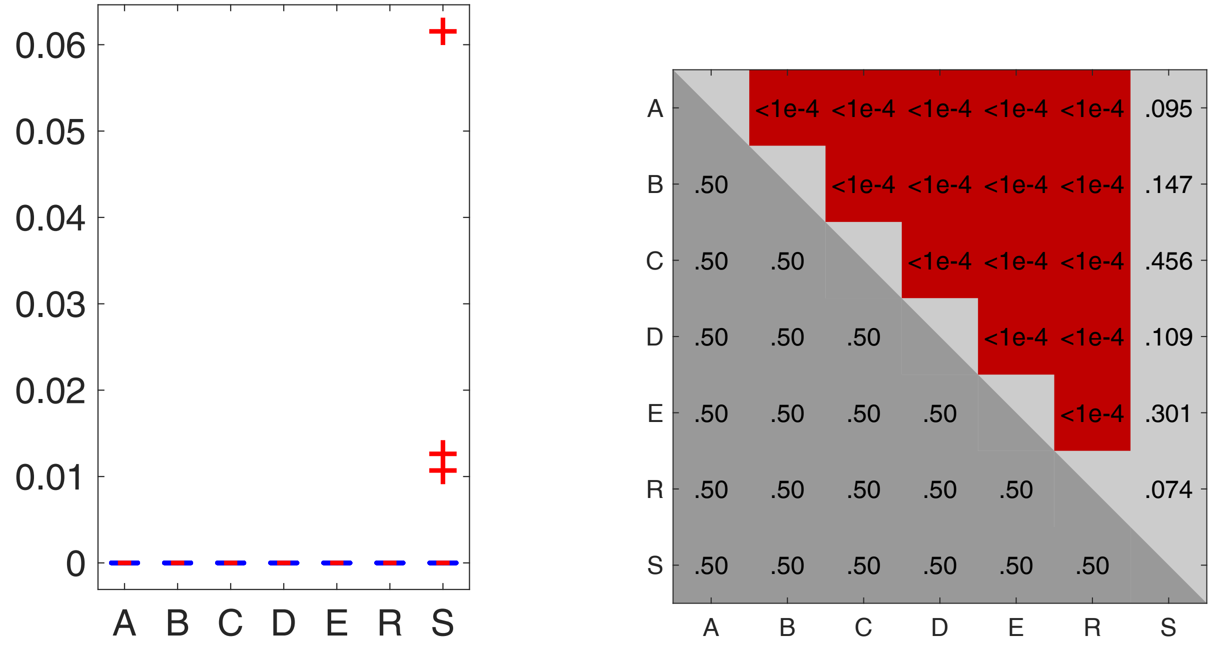

**Heatmap Analysis of Box 40**

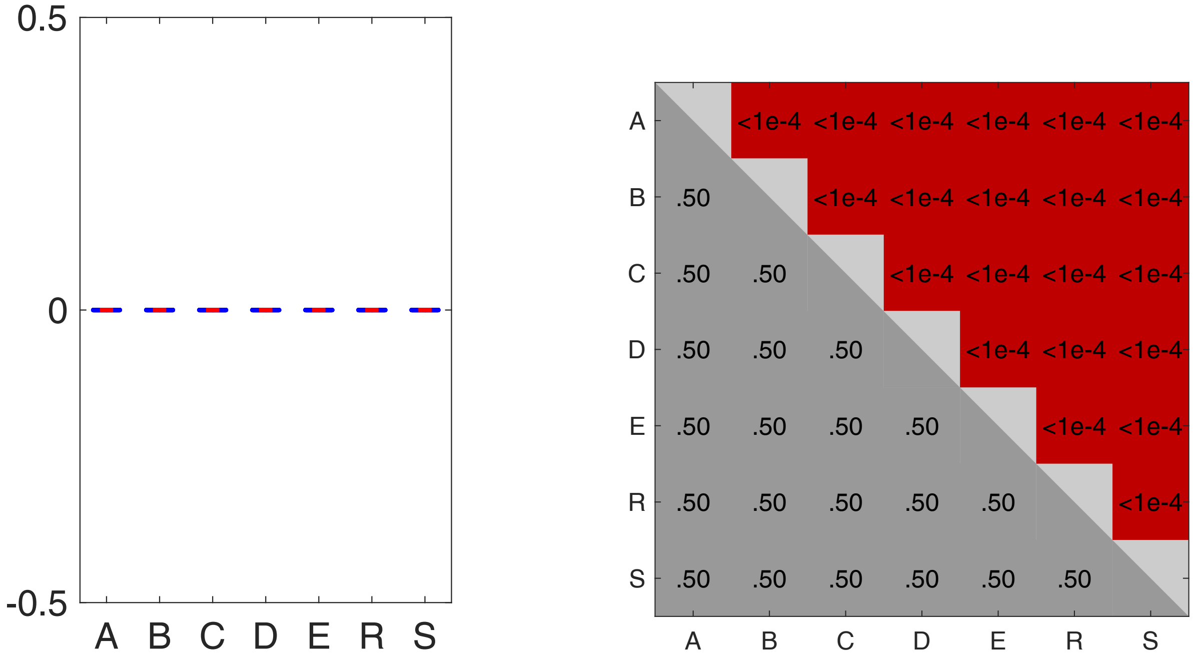


**Heatmap Analysis of Box 41**

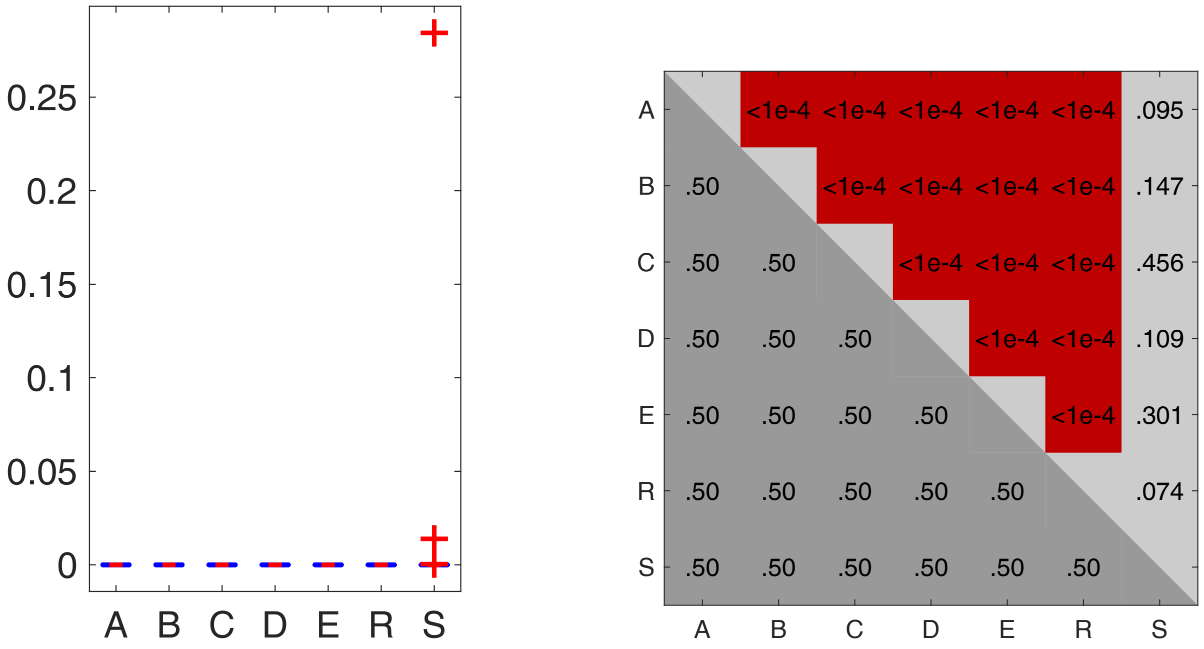

**Heatmap Analysis of Box 42**

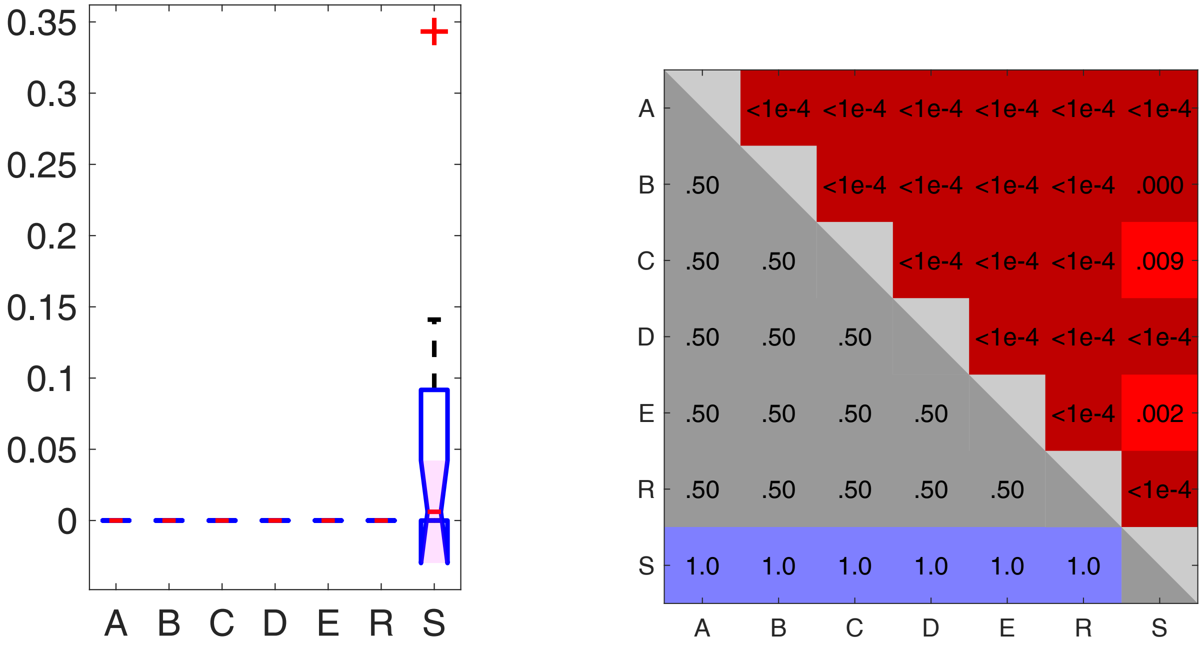


**Heatmap Analysis of Box 43**

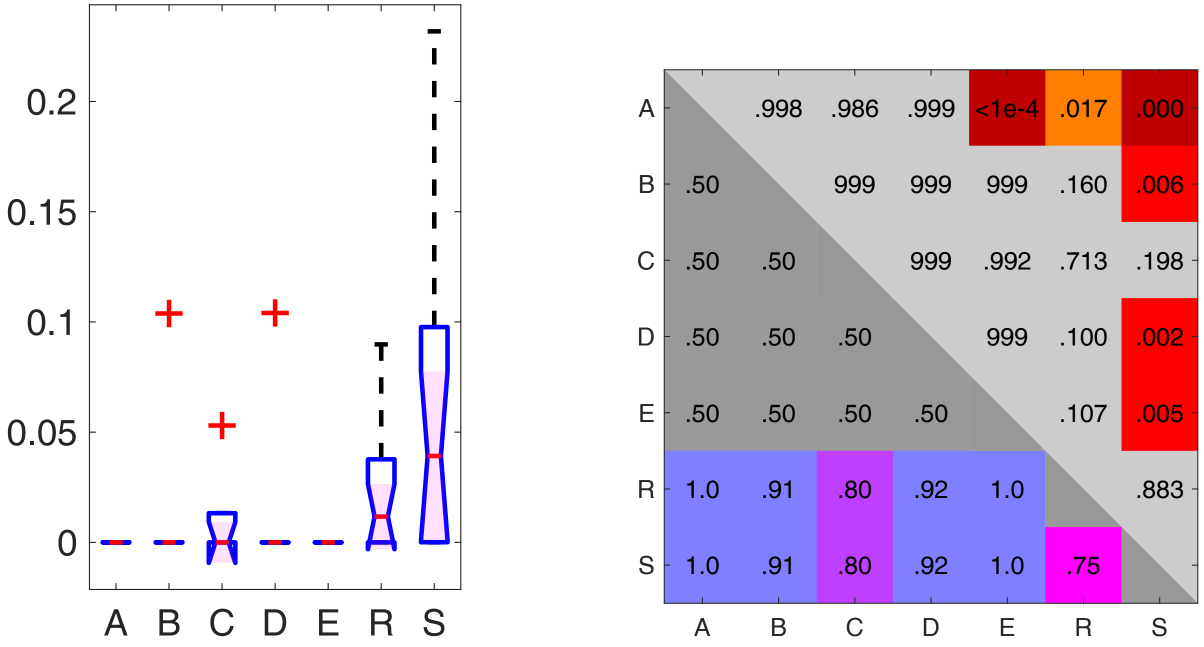

**Heatmap Analysis of Box 44**

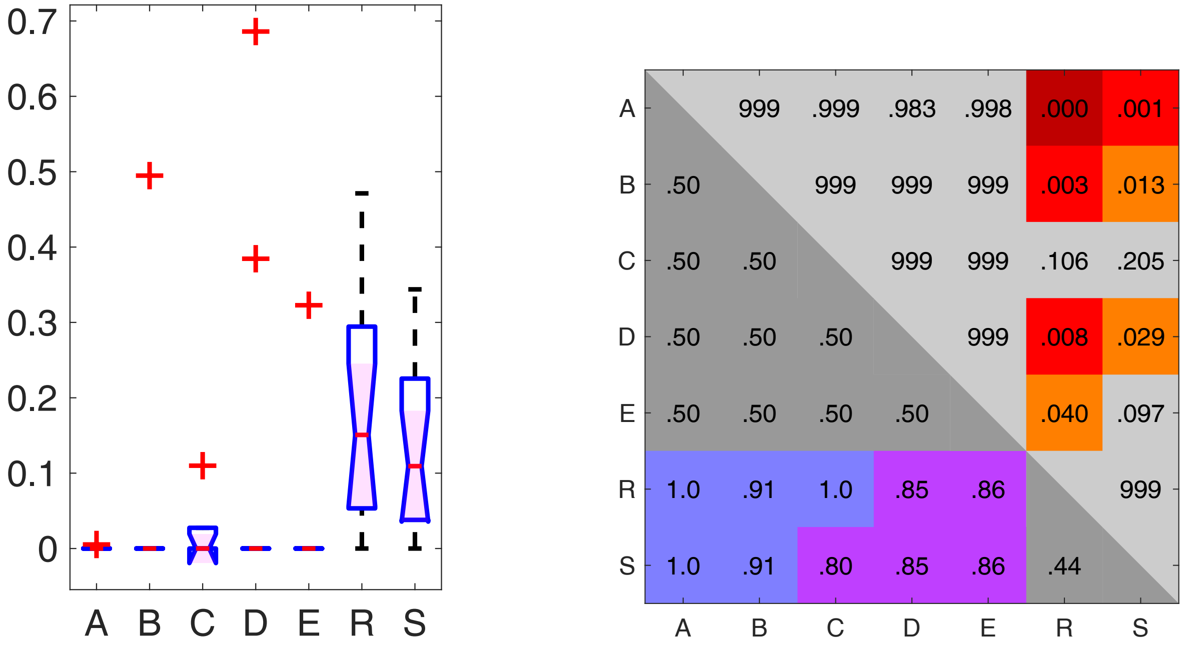


**Heatmap Analysis of Box 45**

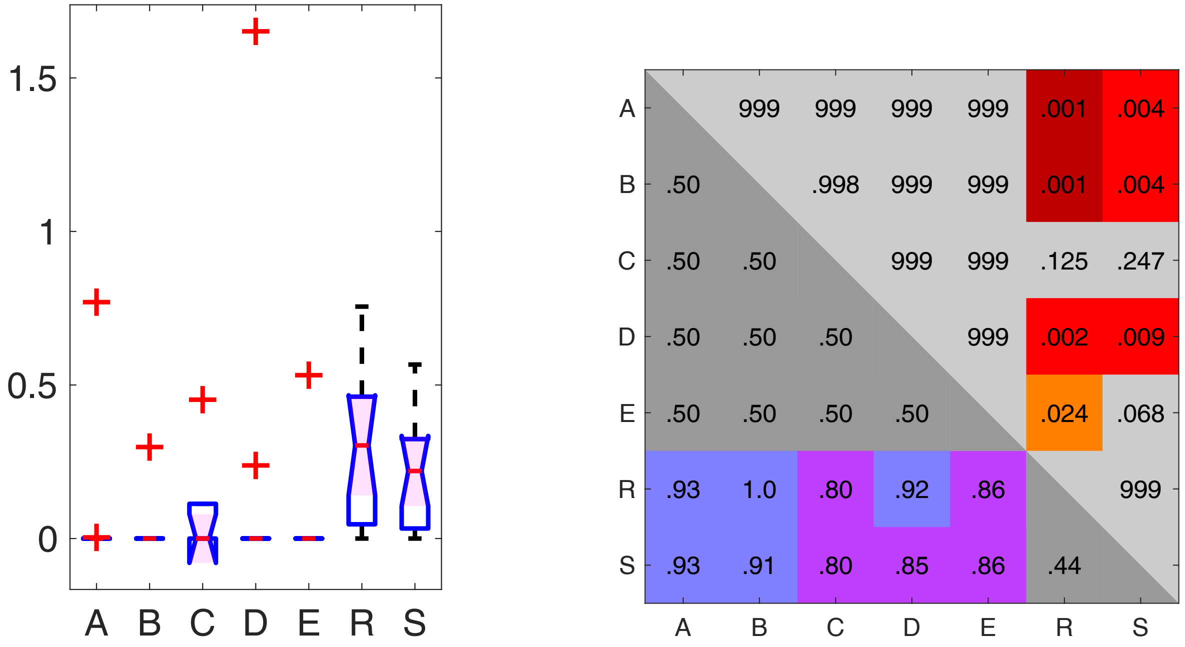

**Heatmap Analysis of Box 46**

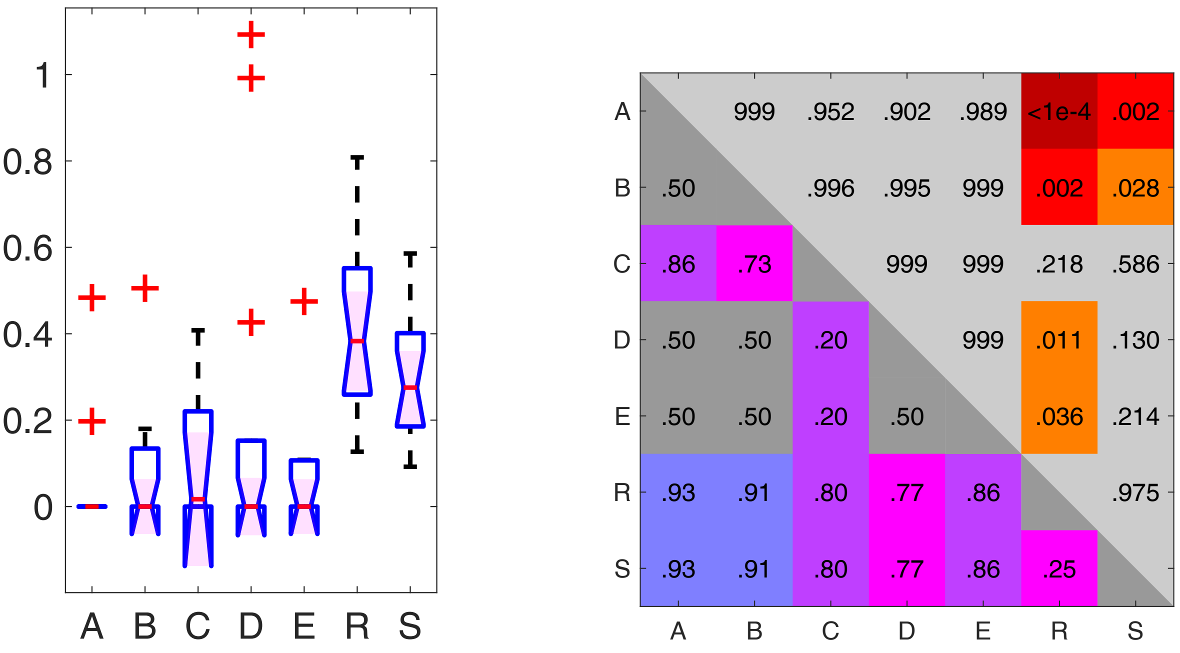


**Heatmap Analysis of Box 47**

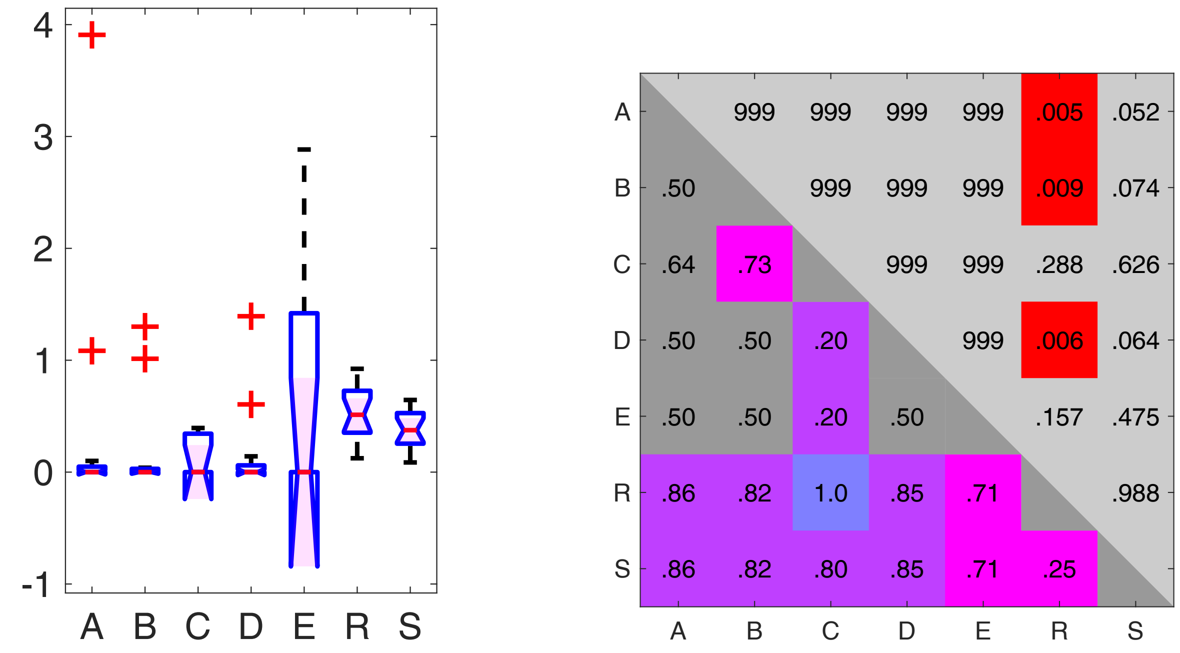

**Heatmap Analysis of Box 48**

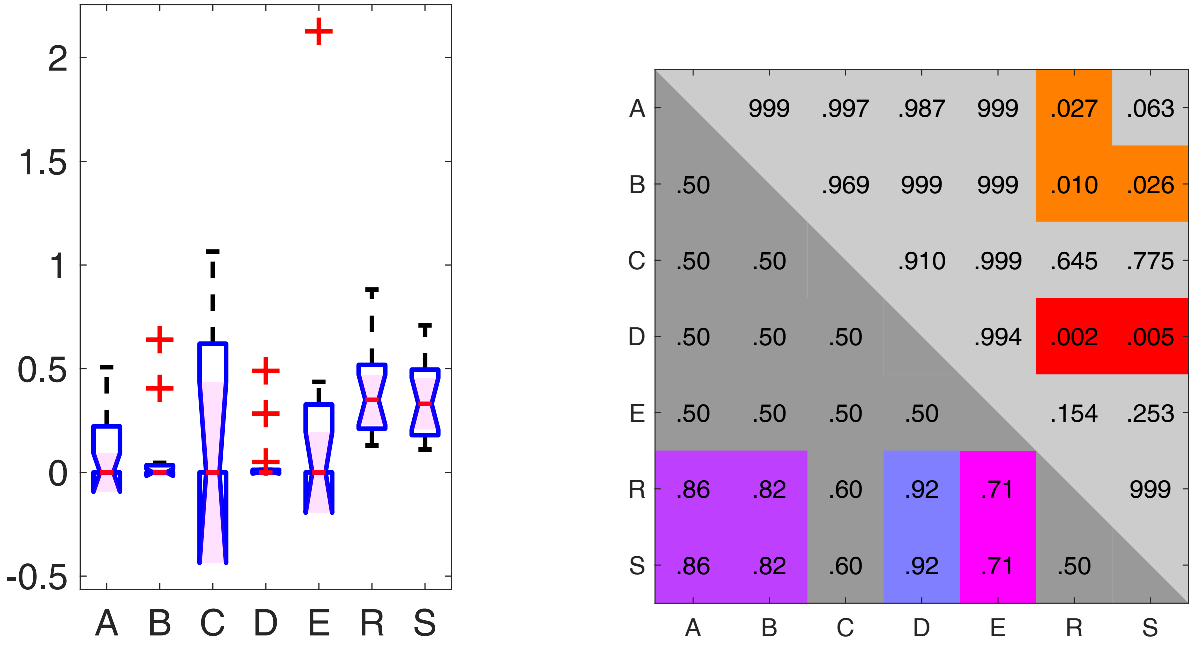


**Heatmap Analysis of Box 49**

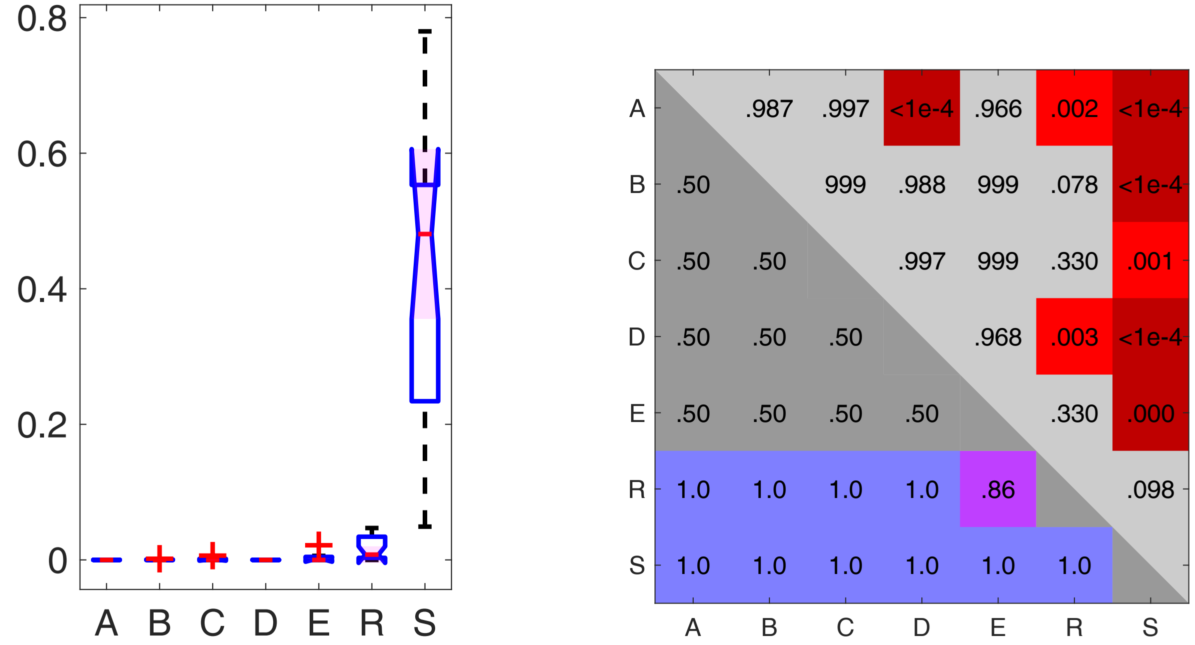

**Heatmap Analysis of Box 4A**

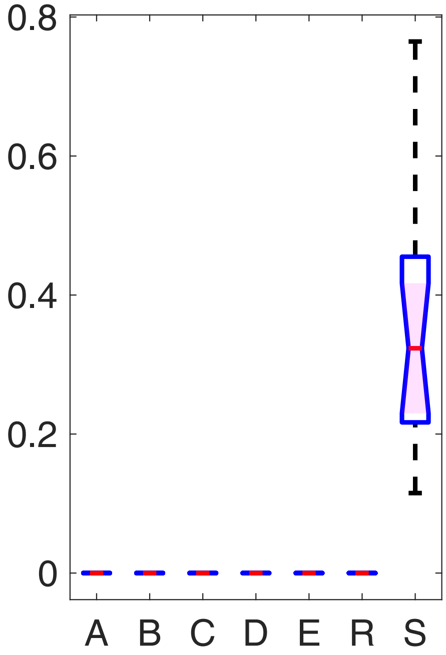


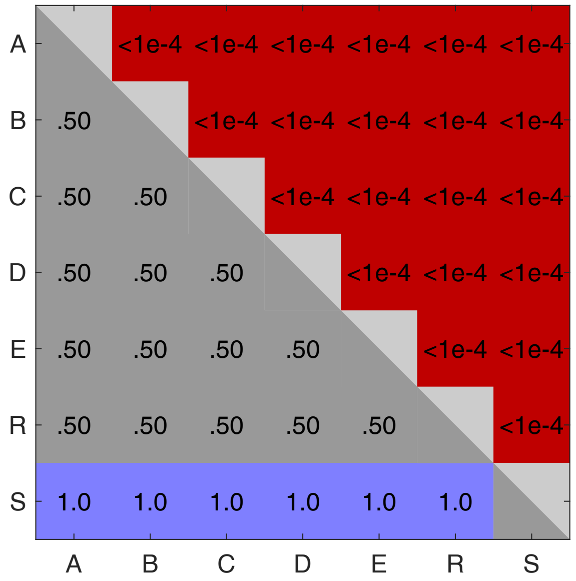


**Heatmap Analysis of Box 4B**

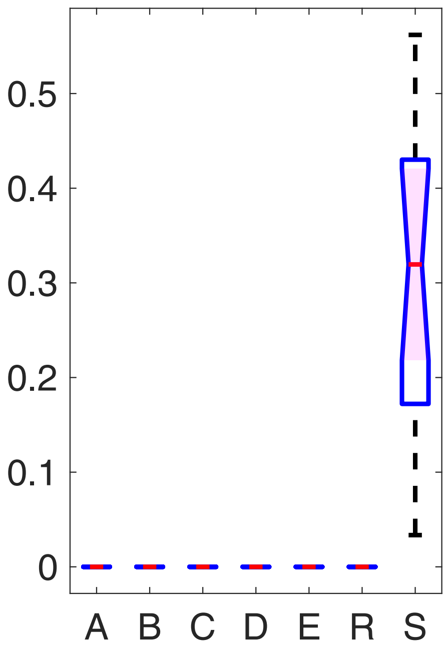


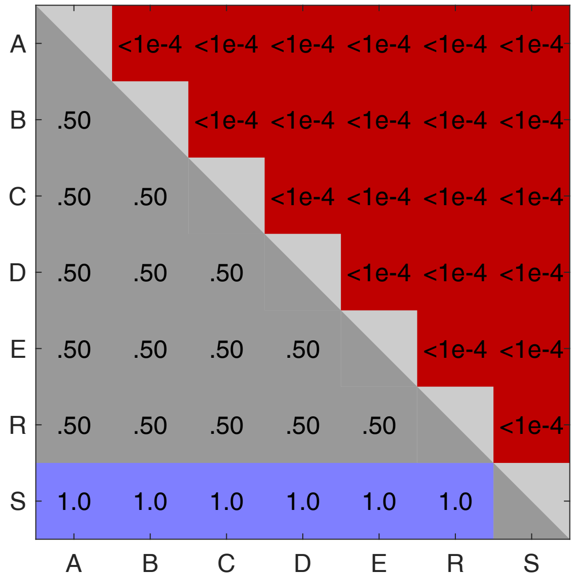

**Heatmap Analysis of Box 4C**

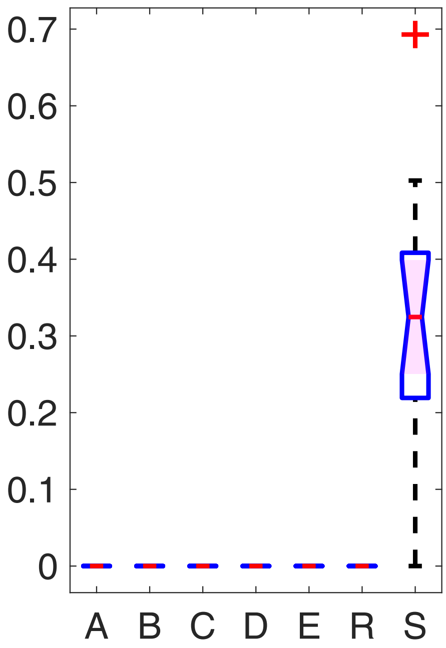


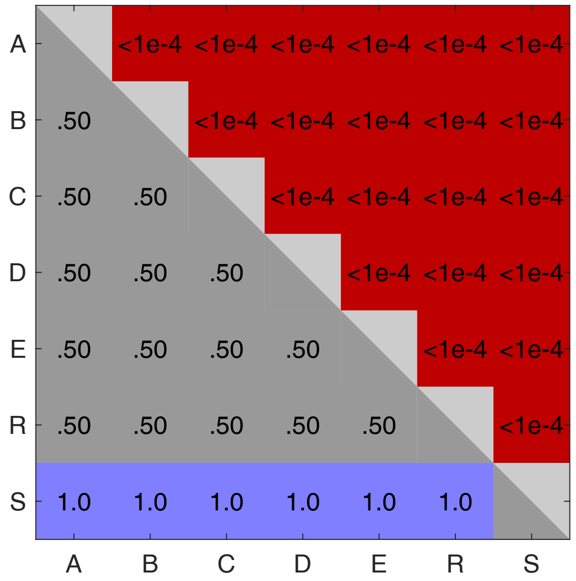


**Heatmap Analysis of Box 4D**

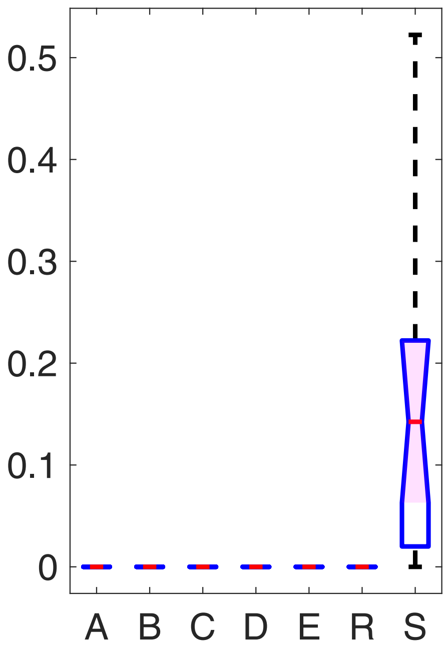


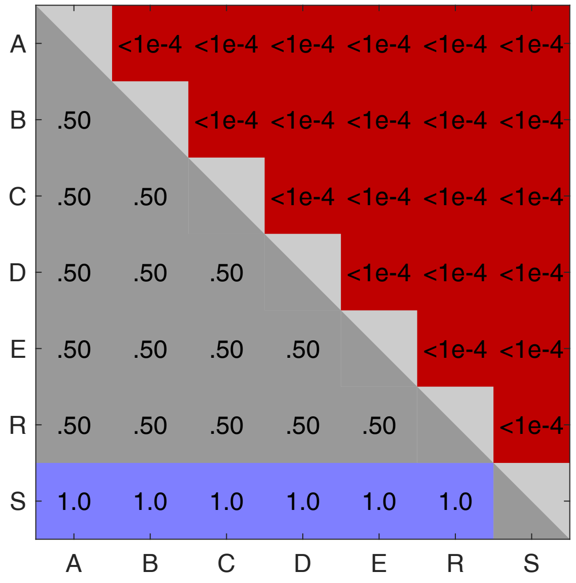

**Heatmap Analysis of Box 4E**

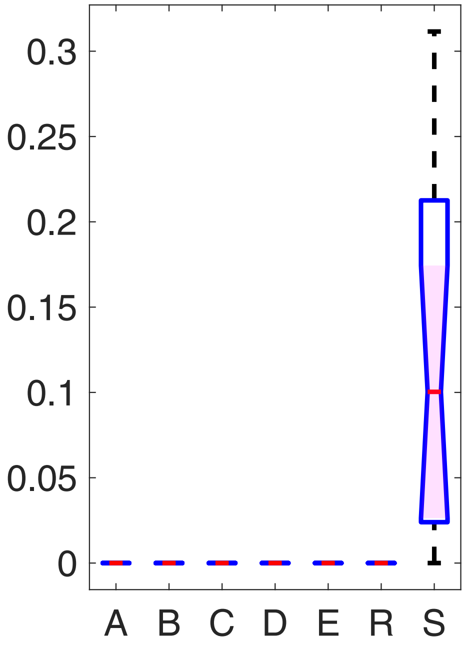


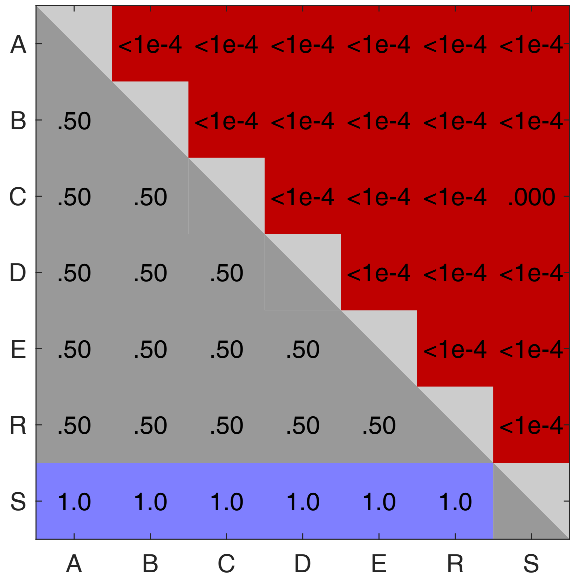


**Heatmap Analysis of Box 4F**

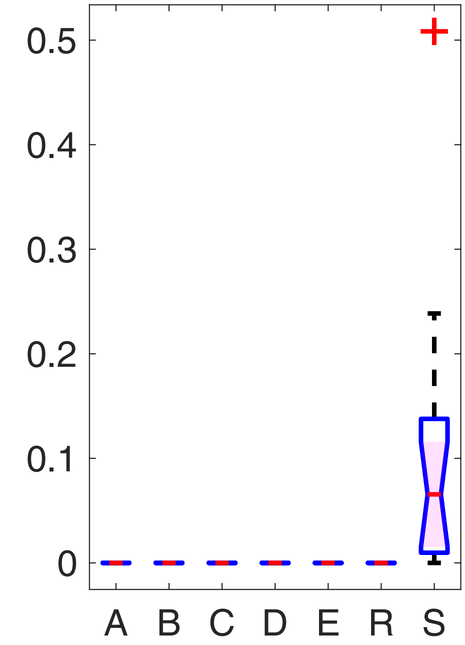


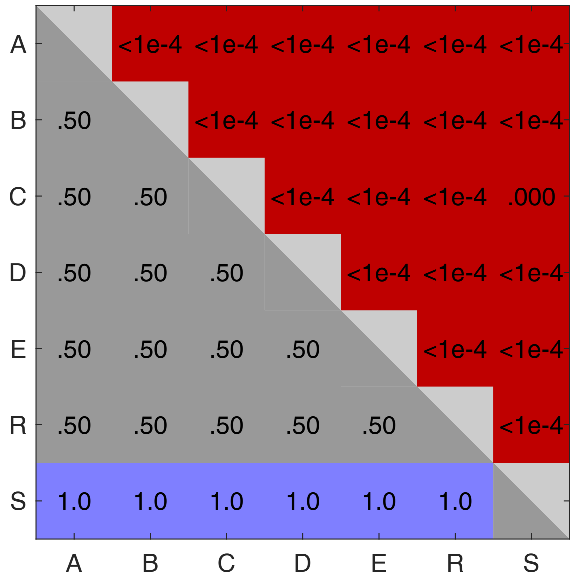

**Heatmap Analysis of Box 4G**

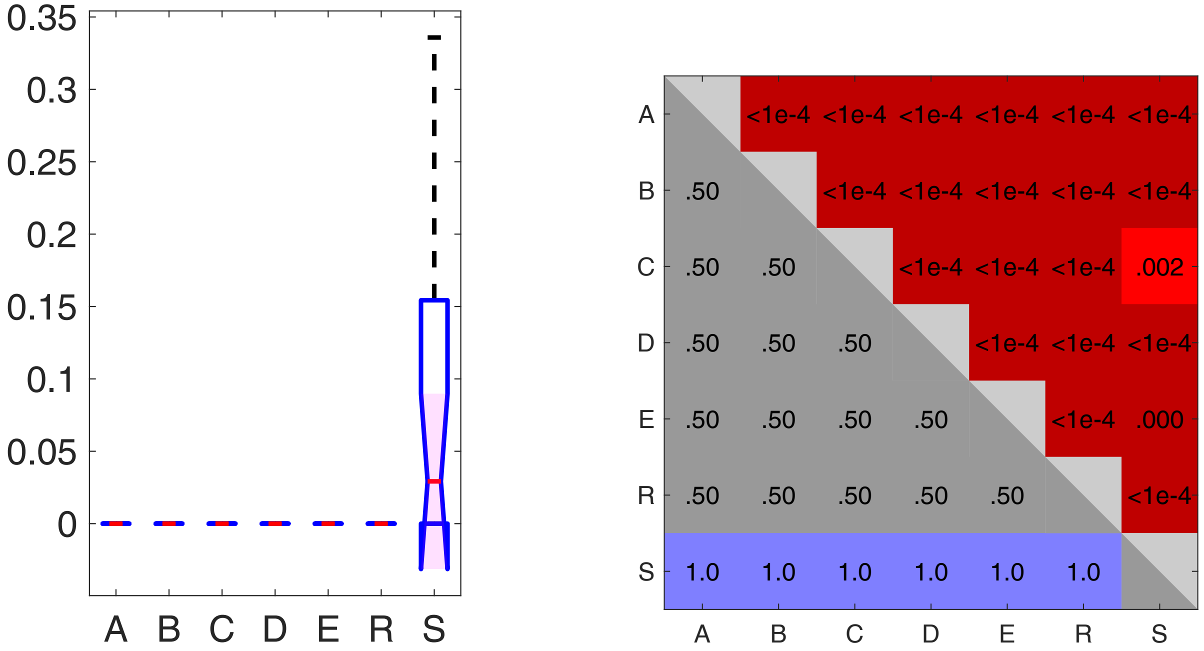


**Heatmap Analysis of Box 4H**

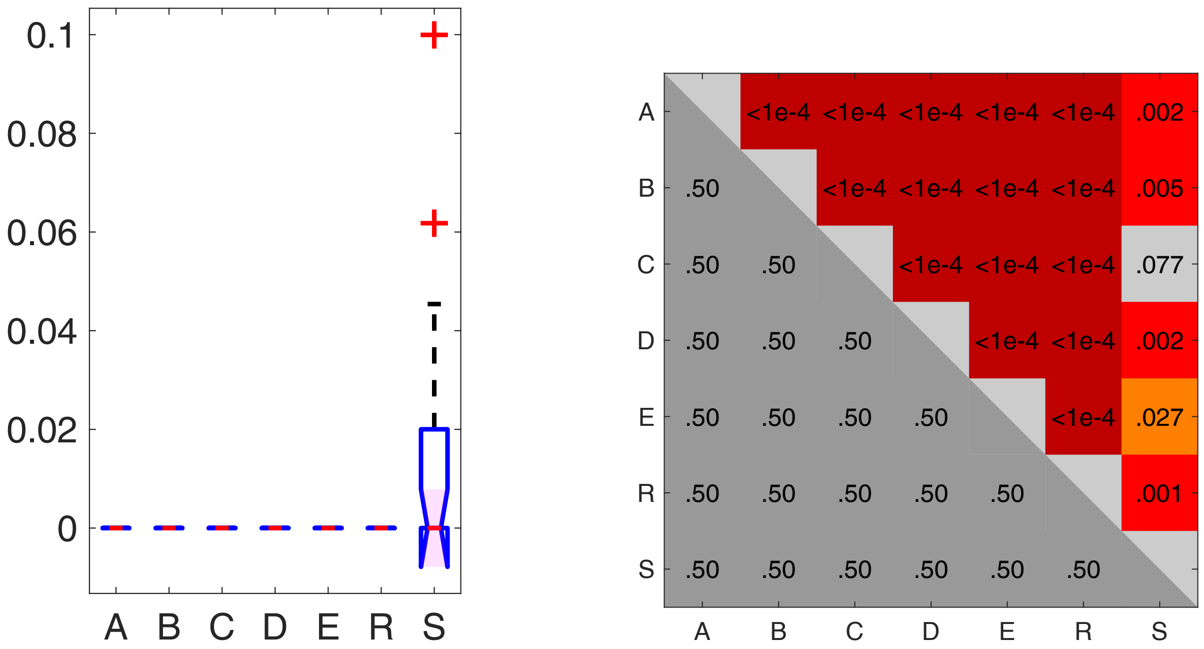

**Heatmap Analysis of Box 50**

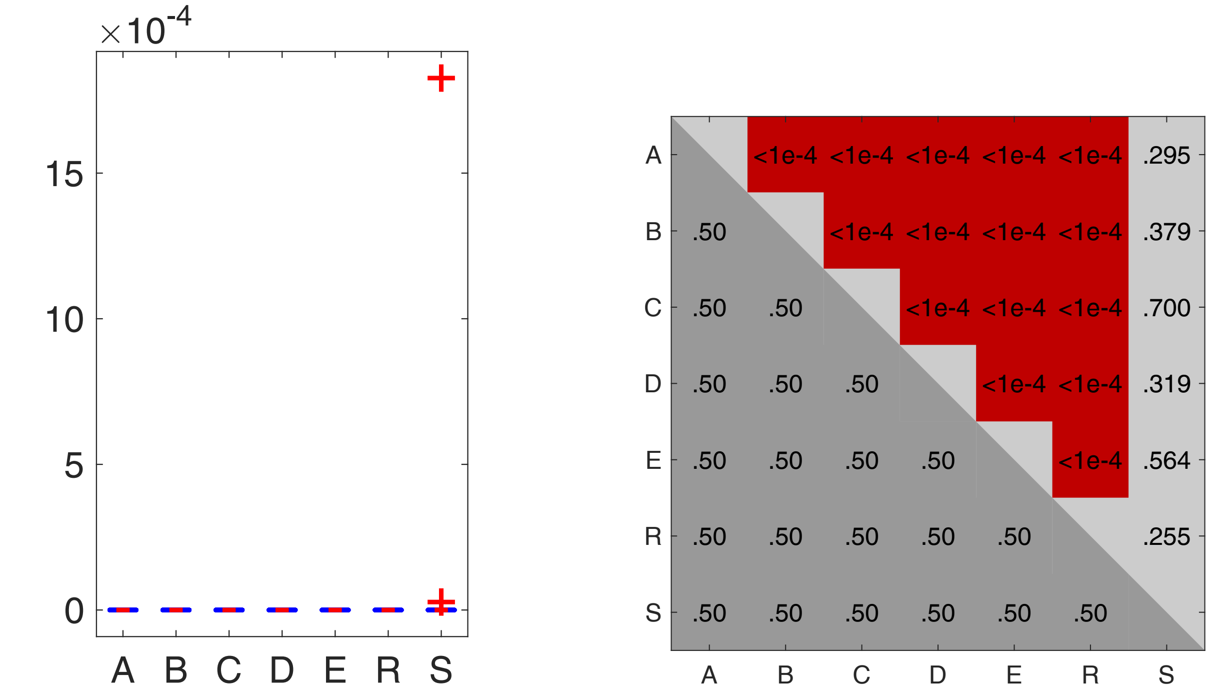


**Heatmap Analysis of Box 51**

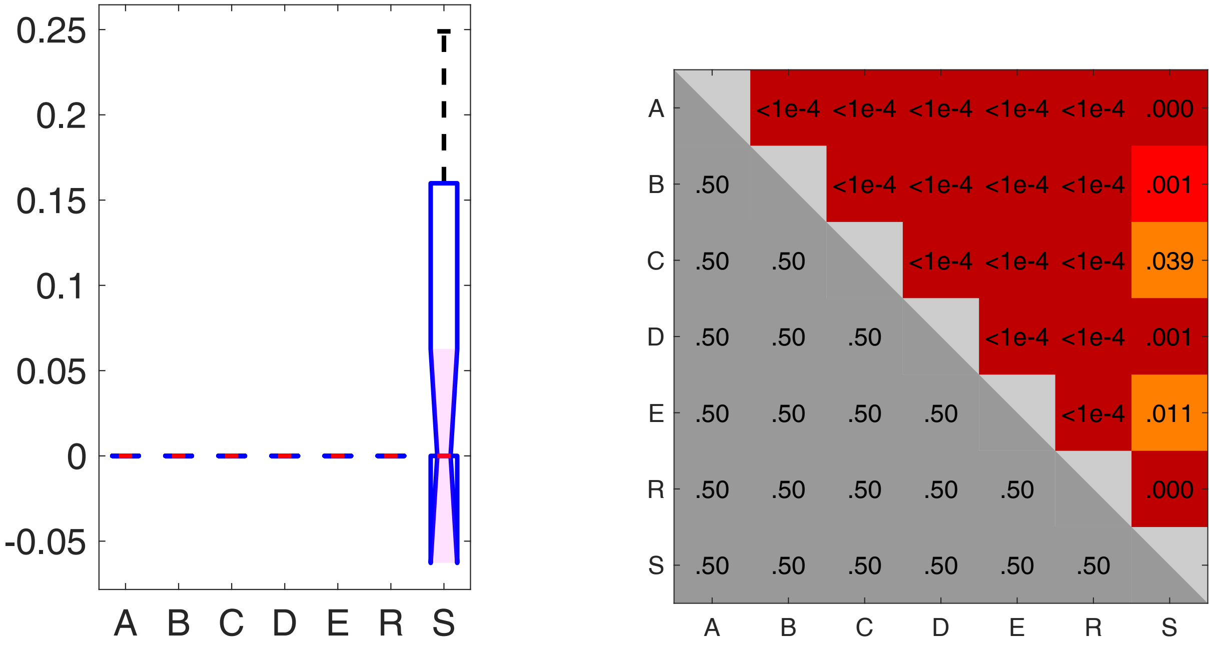

**Heatmap Analysis of Box 52**

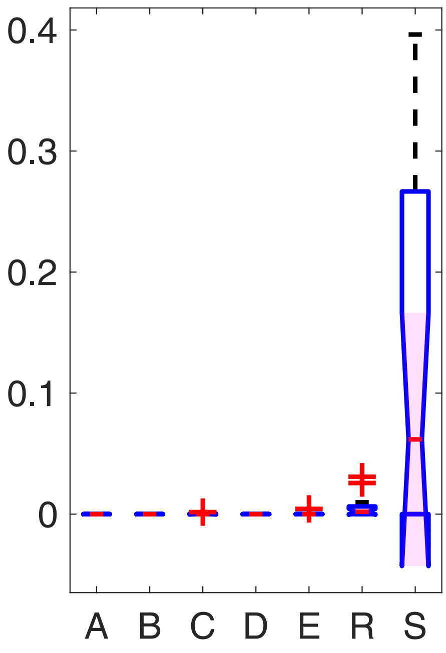


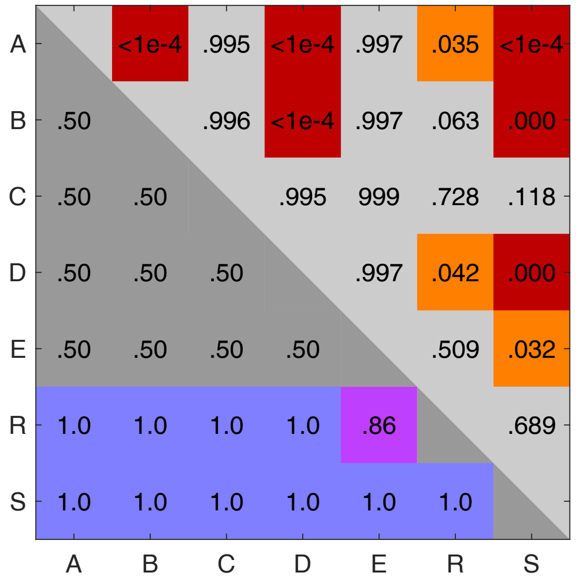


**Heatmap Analysis of Box 53**

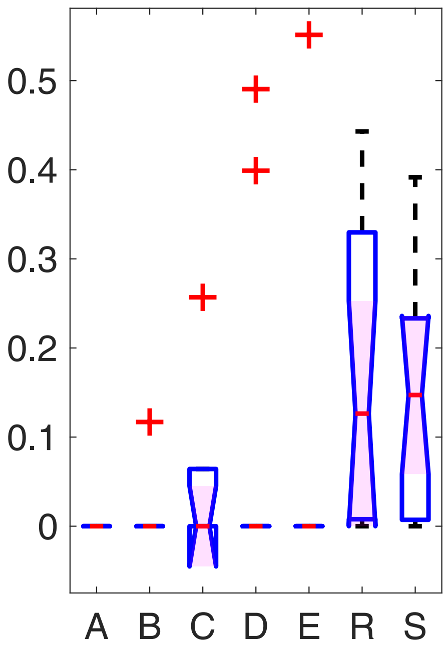


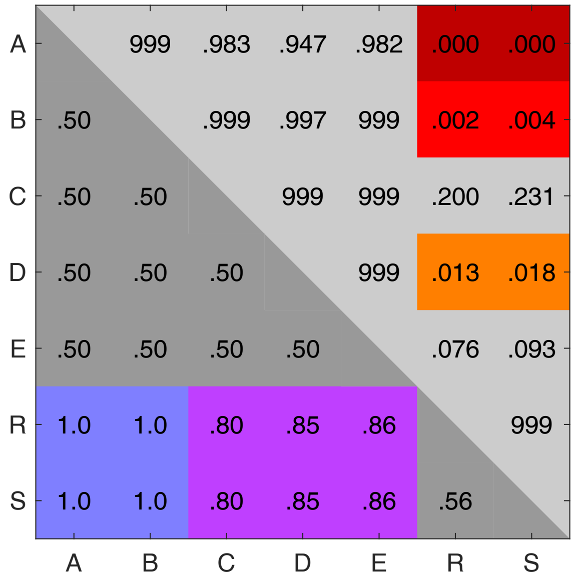

**Heatmap Analysis of Box 54**

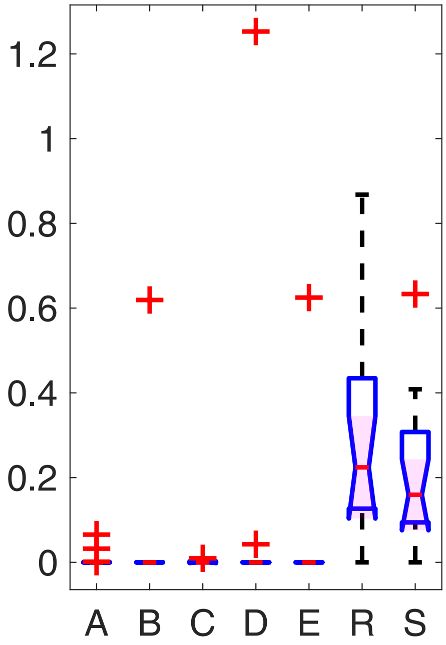


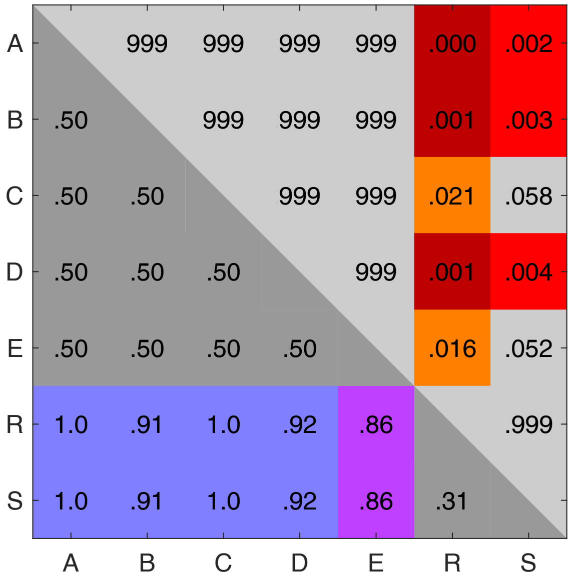


**Heatmap Analysis of Box 55**

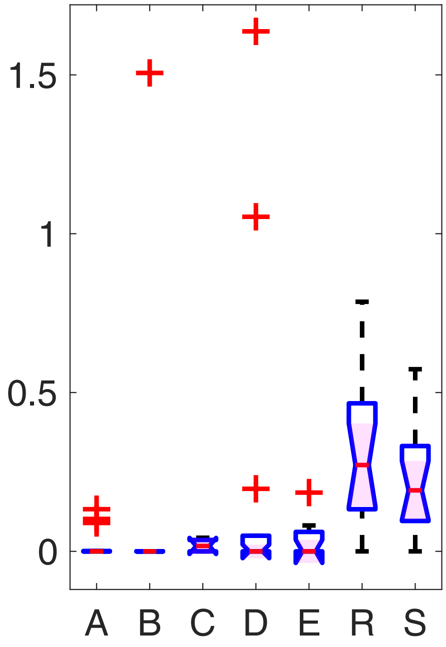


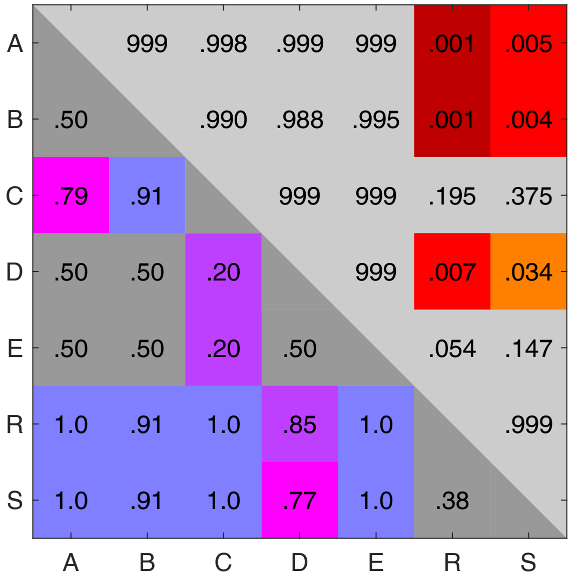

**Heatmap Analysis of Box 56**

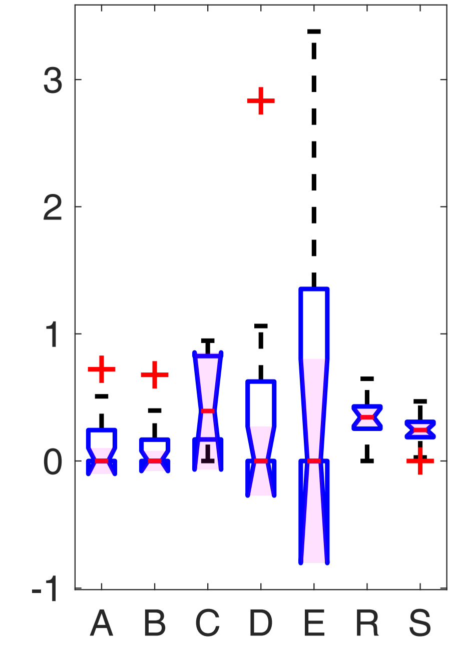


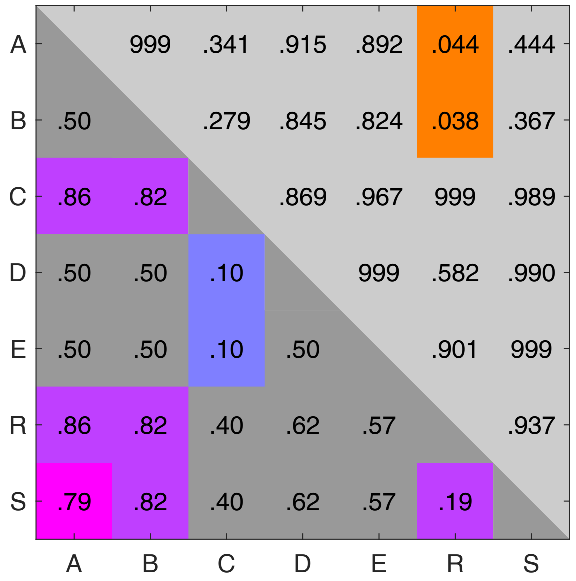


**Heatmap Analysis of Box 57**

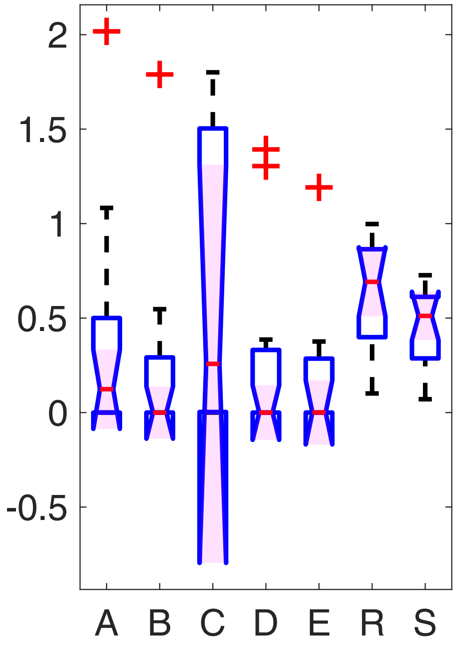


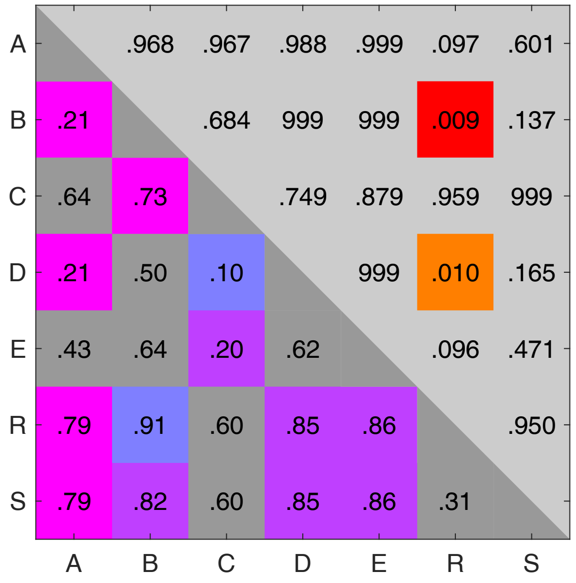

### Heatmap Analysis of Box 58

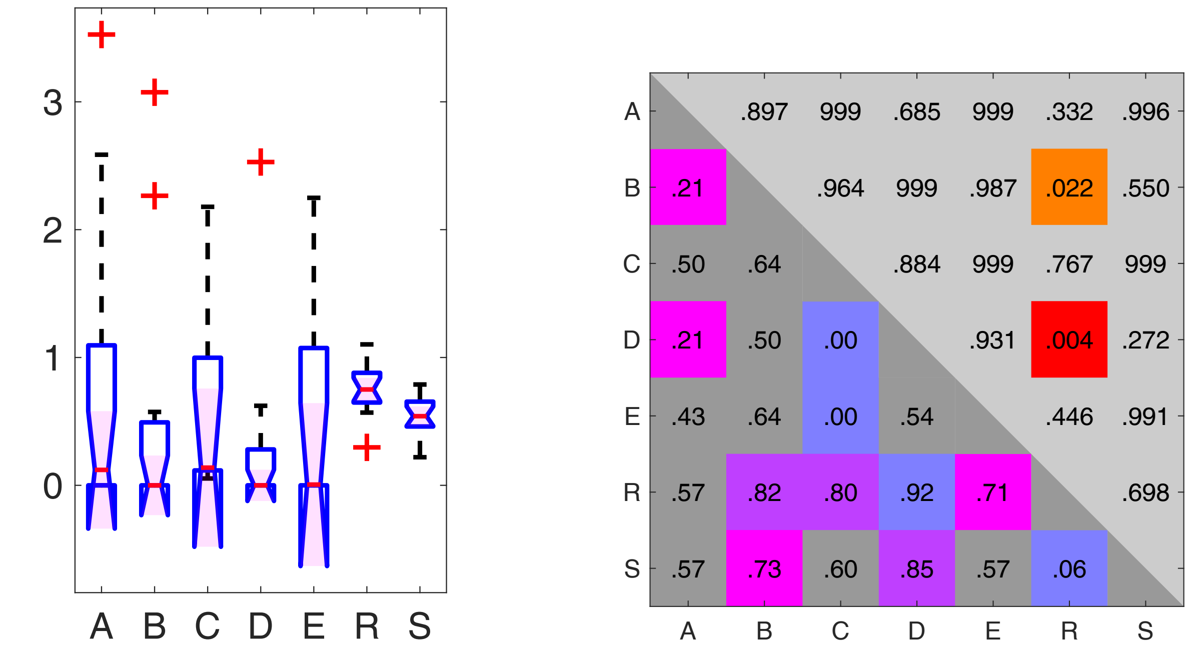


### Heatmap Analysis of Box 59

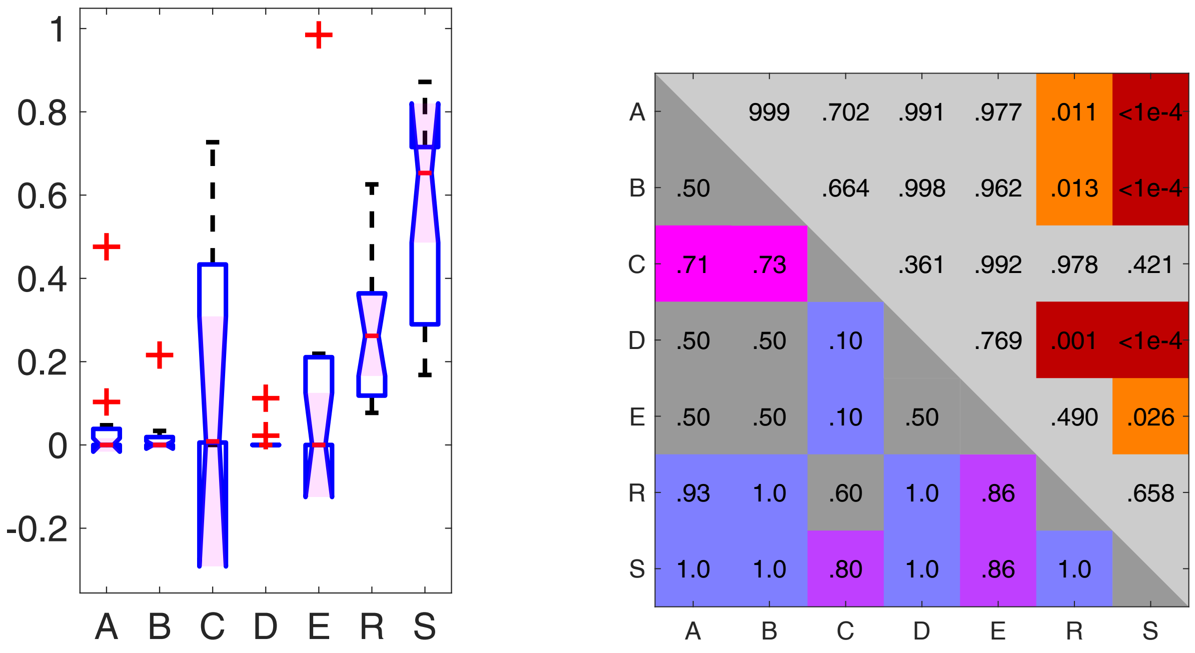

**Heatmap Analysis of Box 5A**

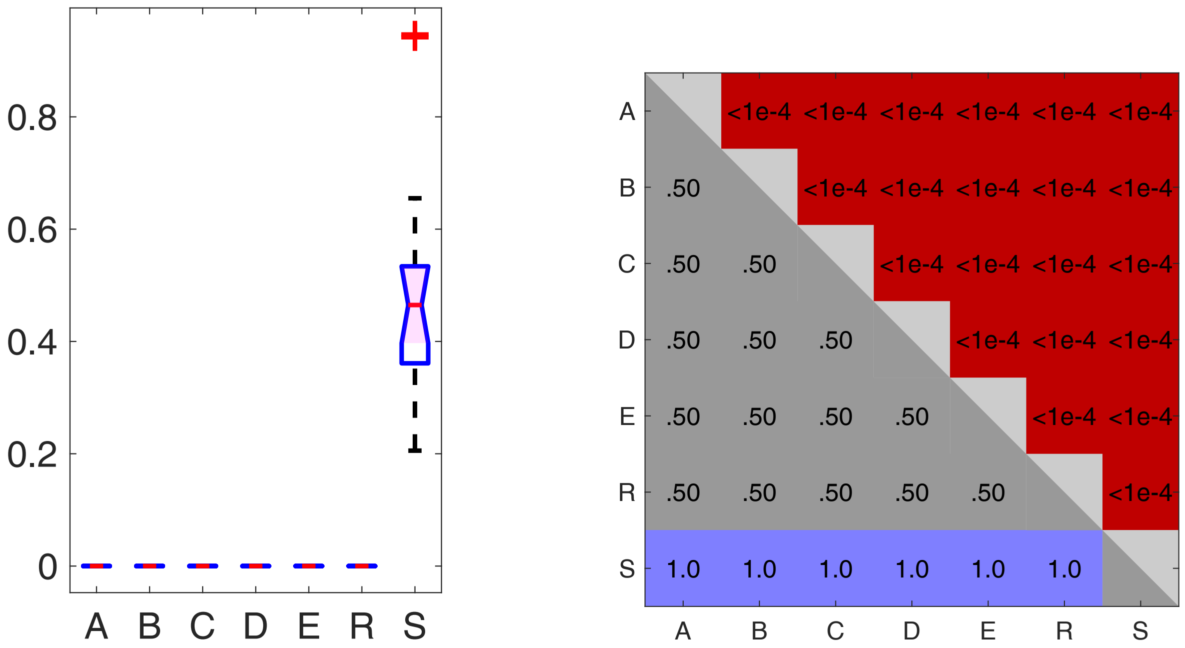


**Heatmap Analysis of Box 5B**

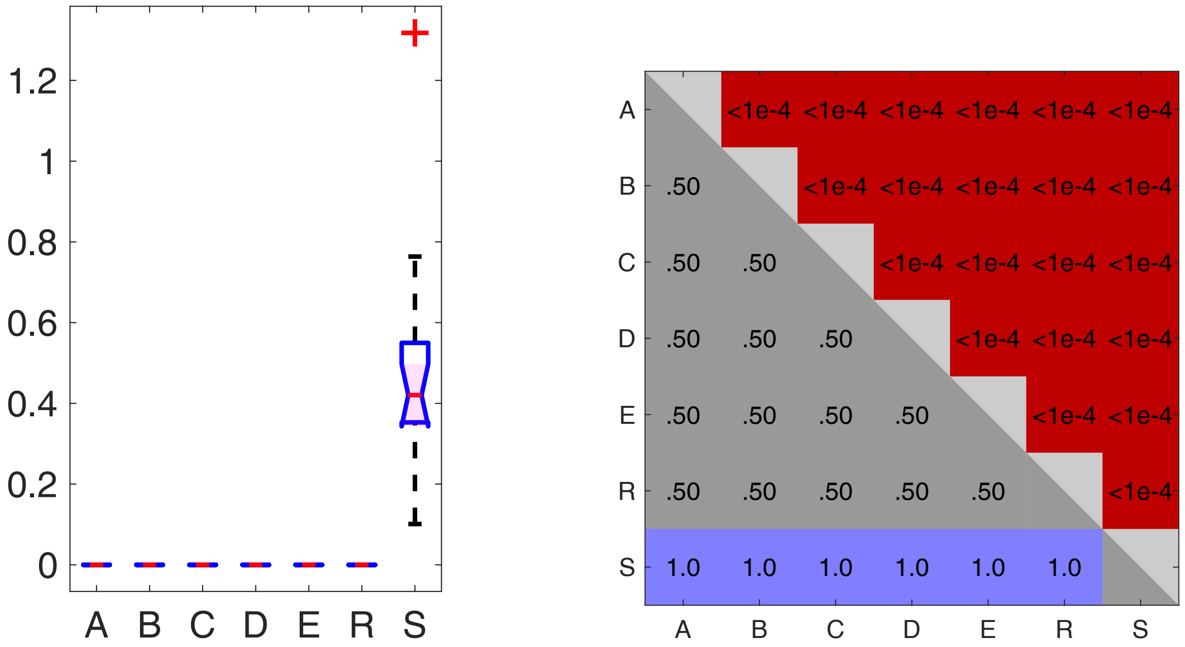

**Heatmap Analysis of Box 5C**

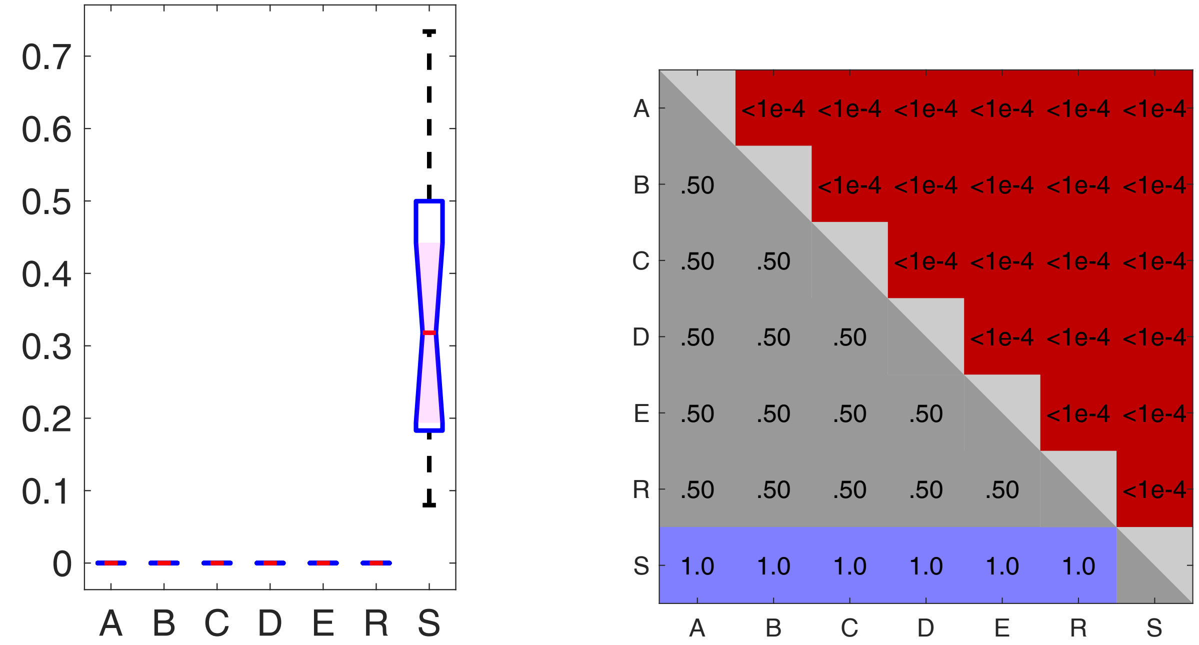


**Heatmap Analysis of Box 5D**

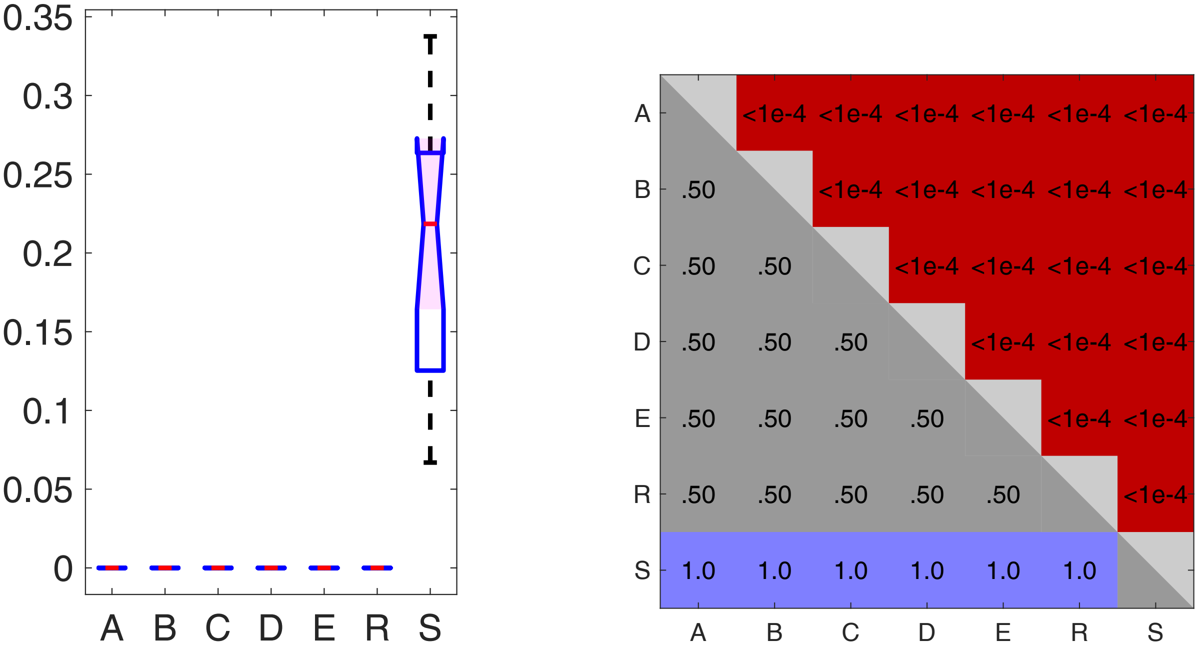

**Heatmap Analysis of Box 5E**

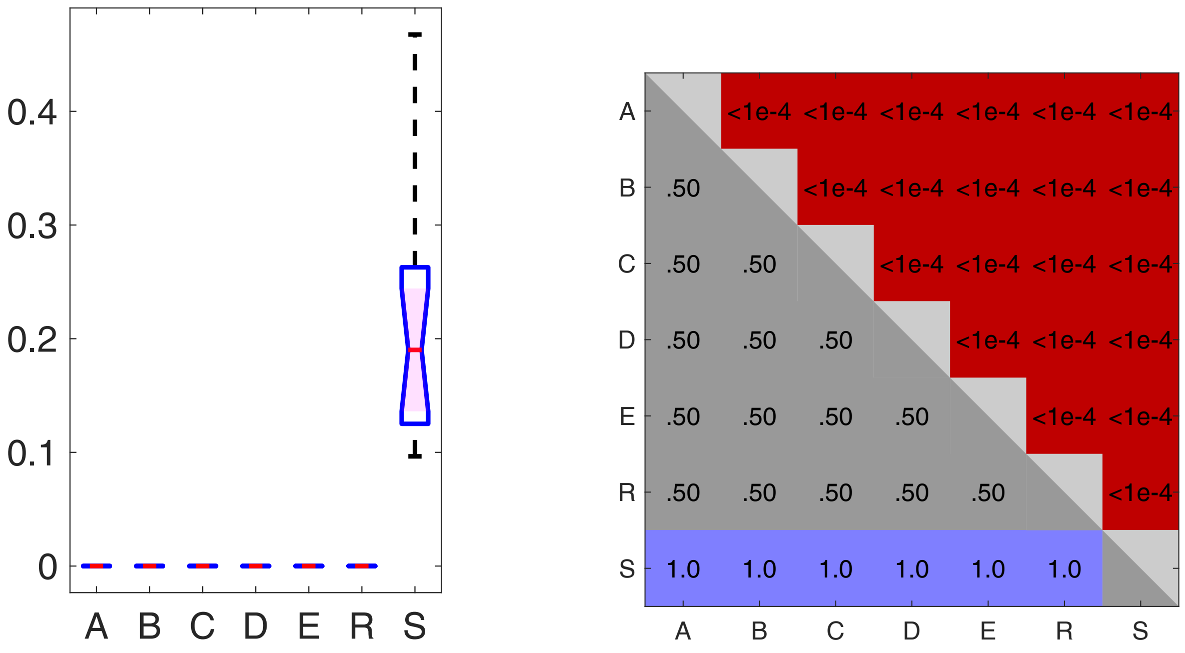


**Heatmap Analysis of Box 5F**

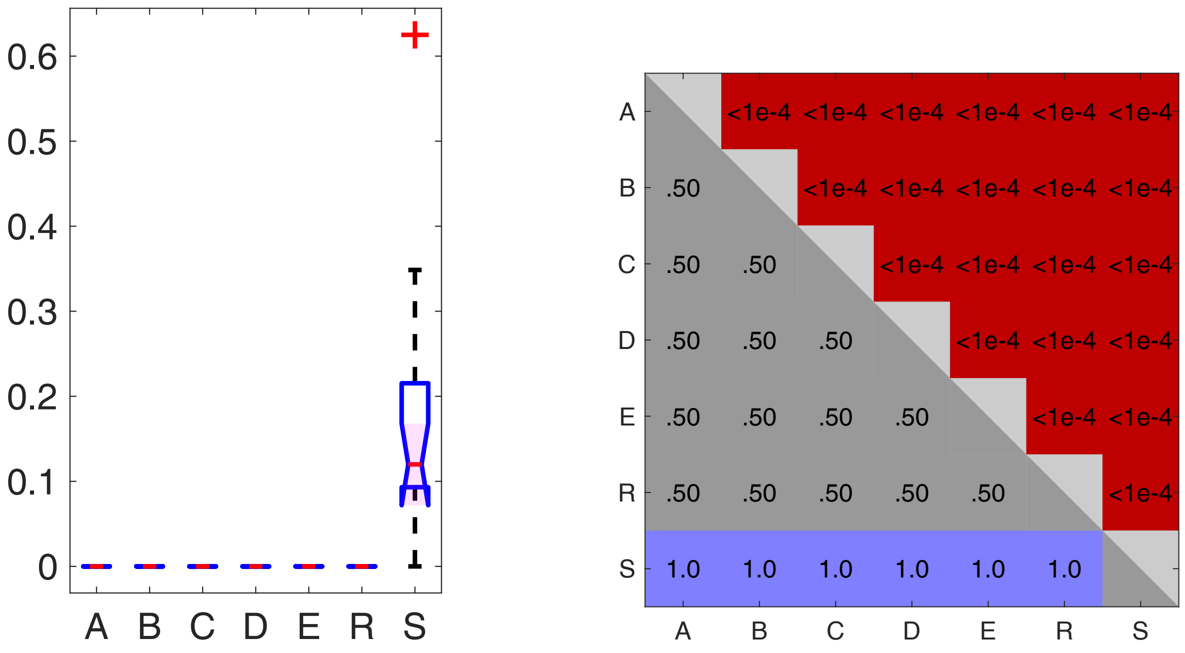

**Heatmap Analysis of Box 5G**

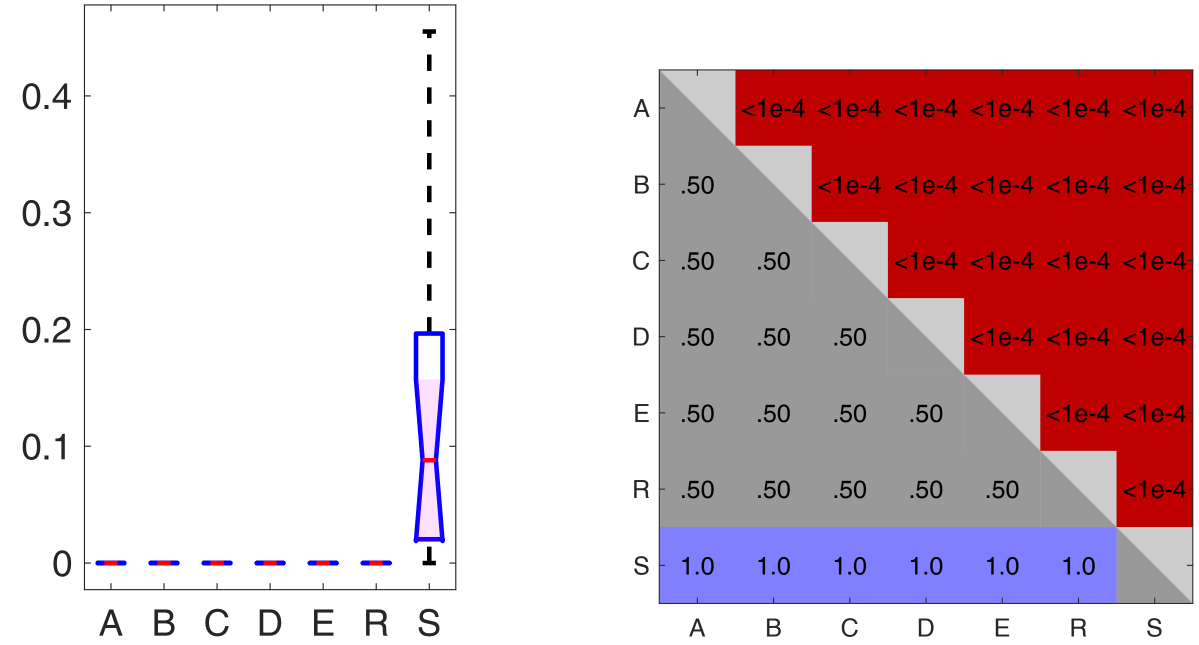


**Heatmap Analysis of Box 5H**

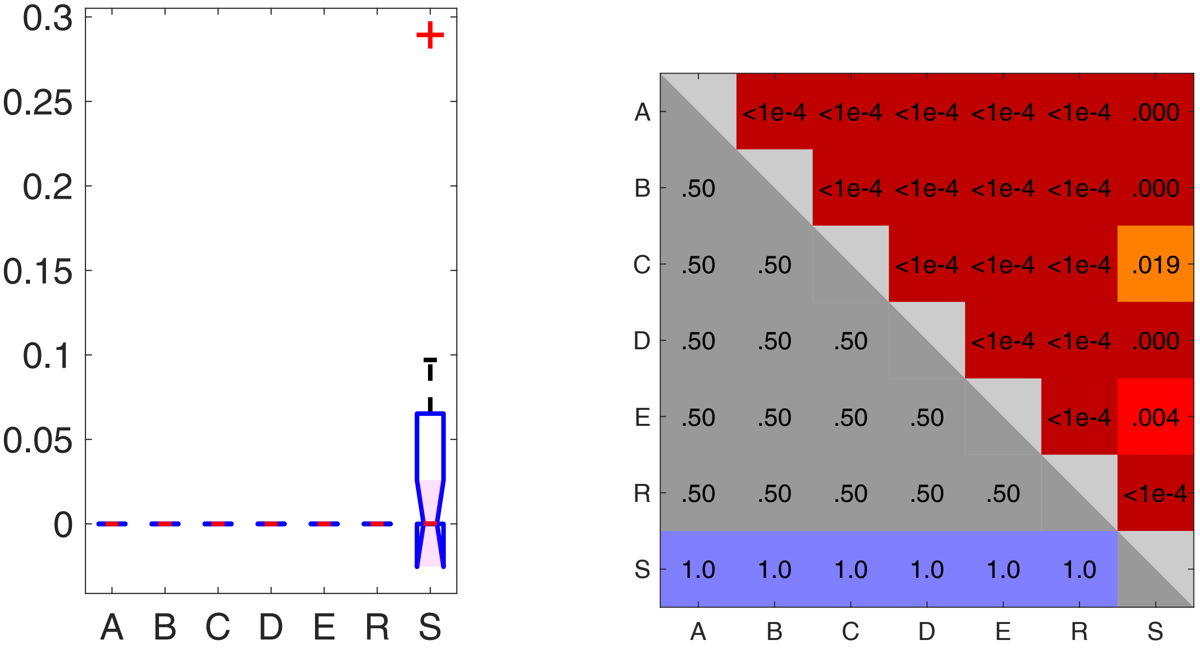

**Heatmap Analysis of Box 60**

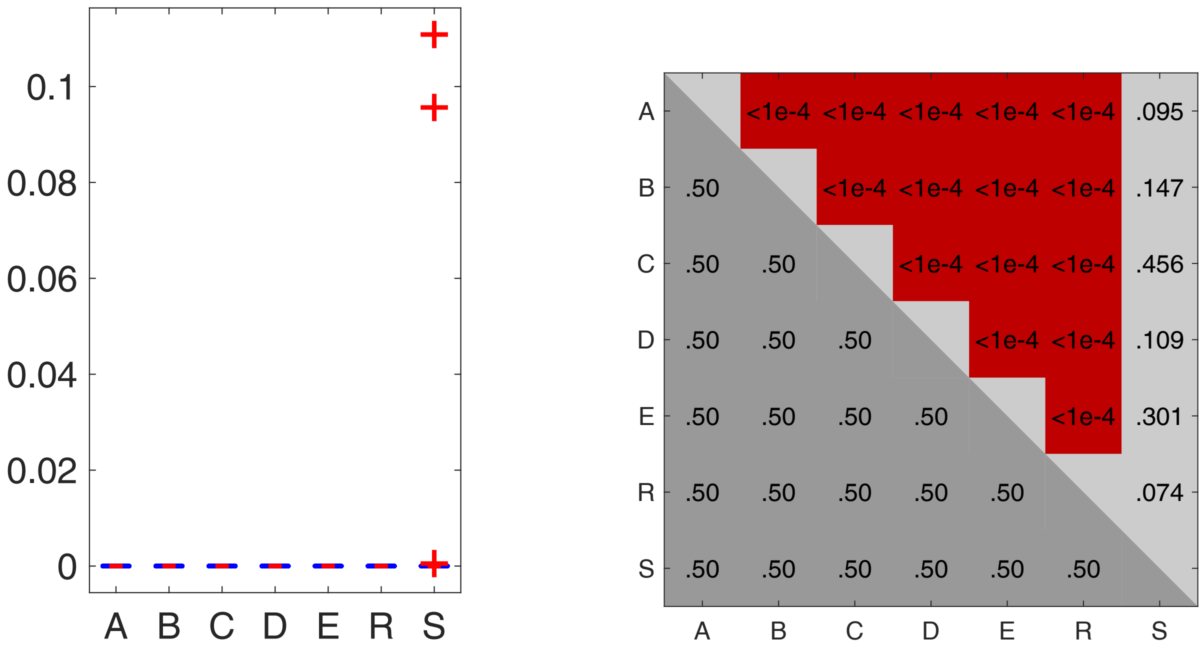


**Heatmap Analysis of Box 61**

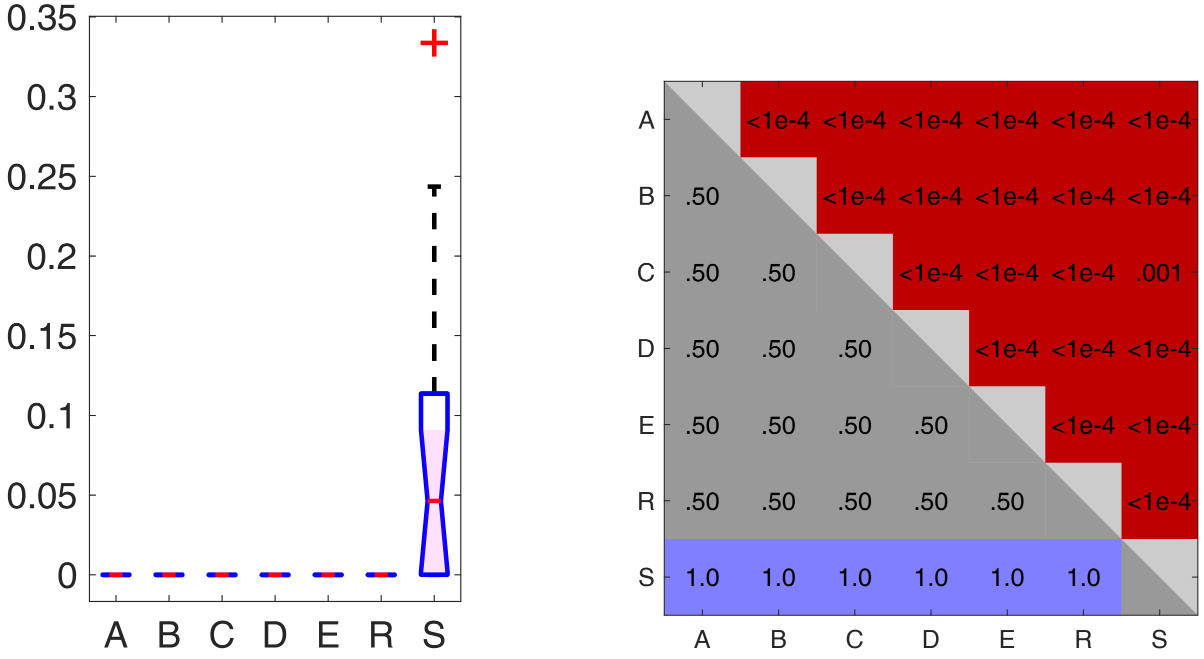

**Heatmap Analysis of Box 62**

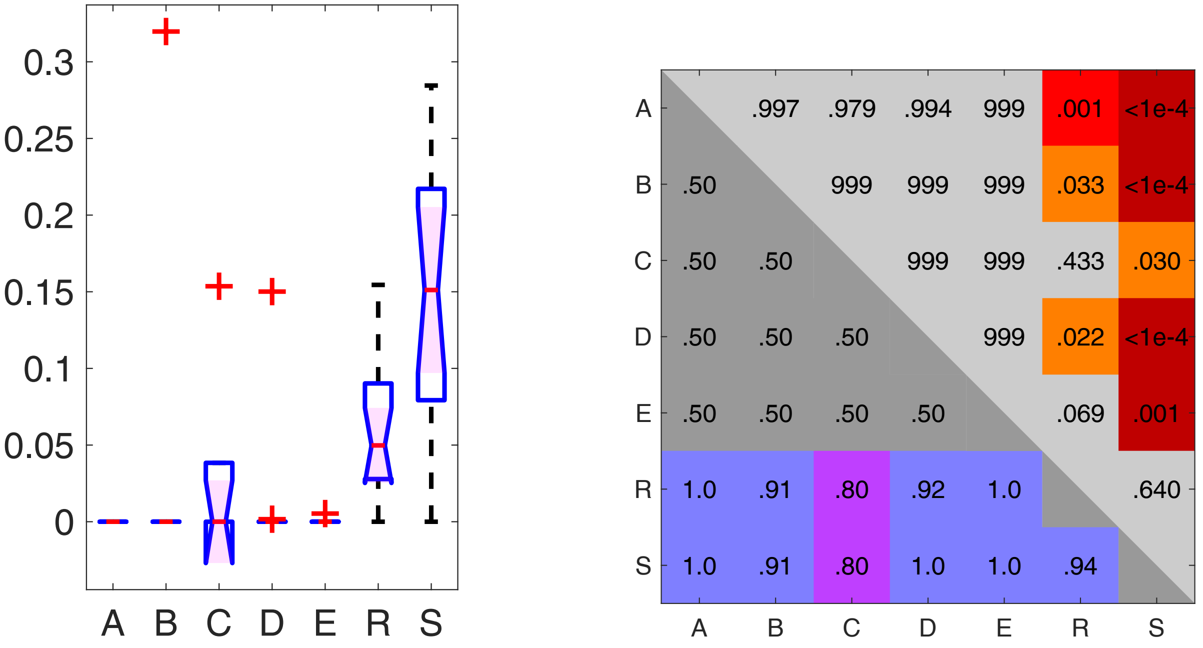


**Heatmap Analysis of Box 63**

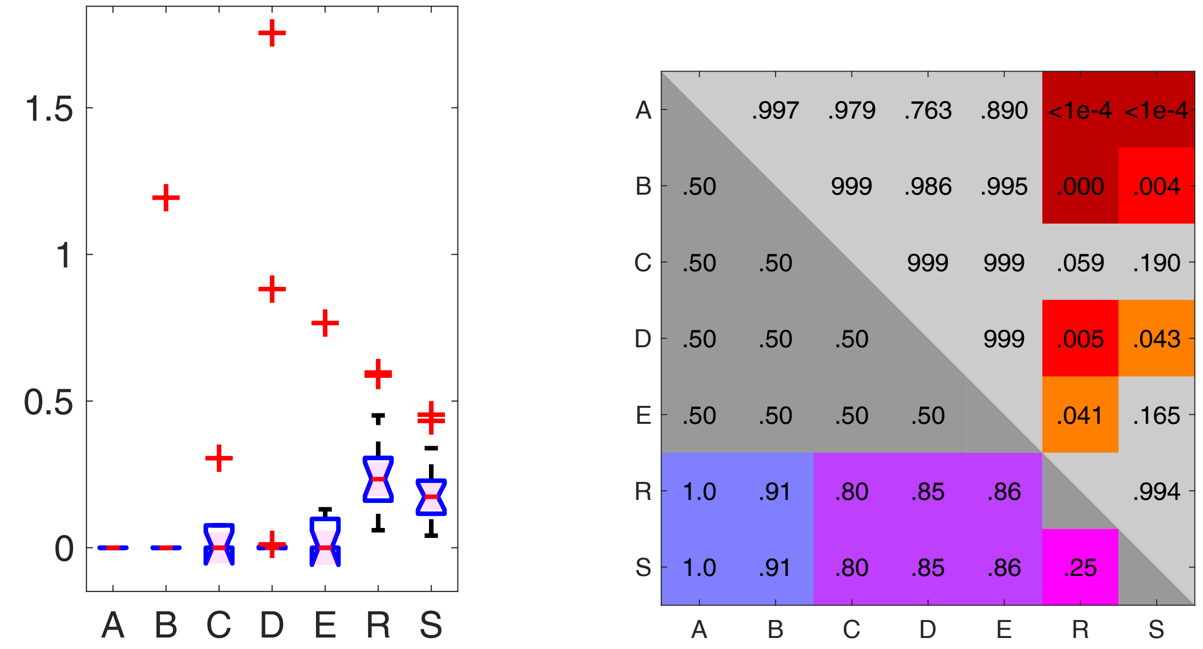

**Heatmap Analysis of Box 64**

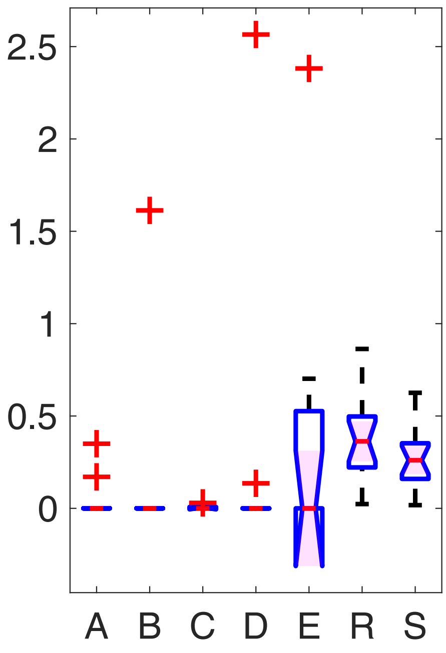


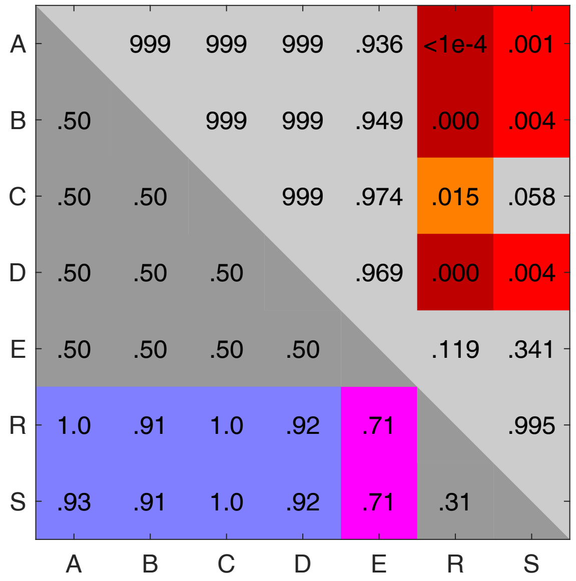


**Heatmap Analysis of Box 65**

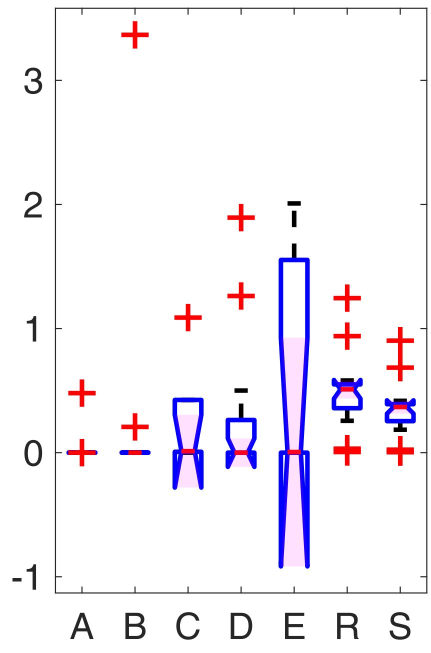


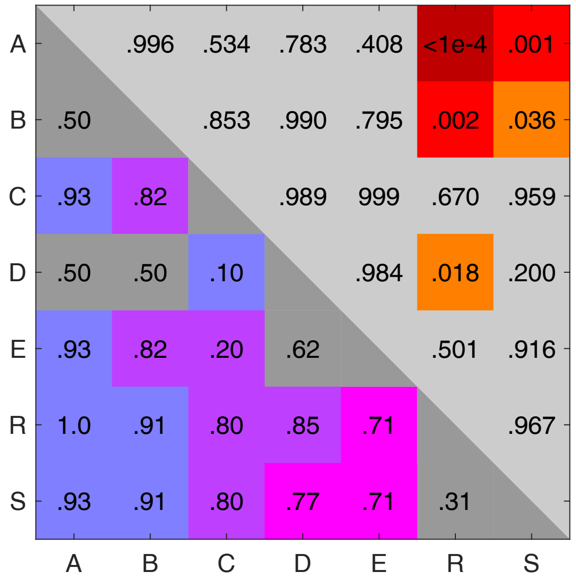

**Heatmap Analysis of Box 66**

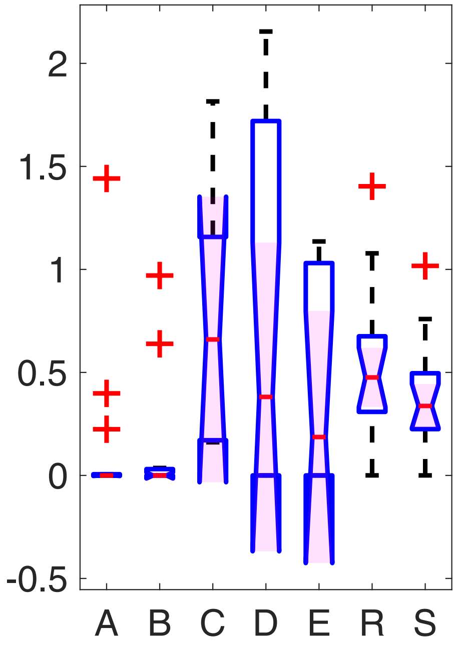


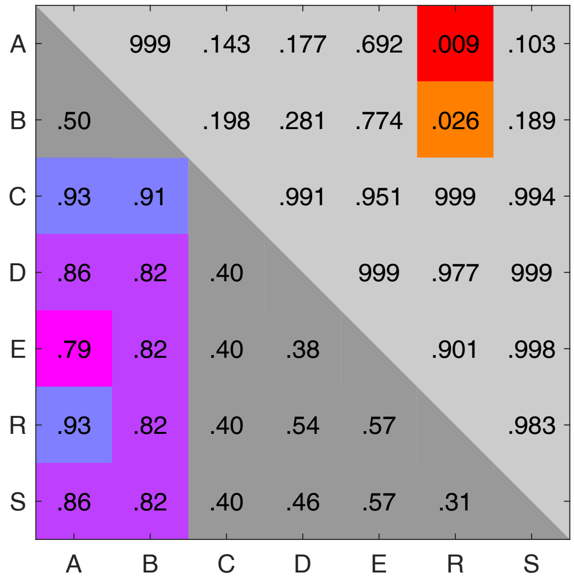


**Heatmap Analysis of Box 67**

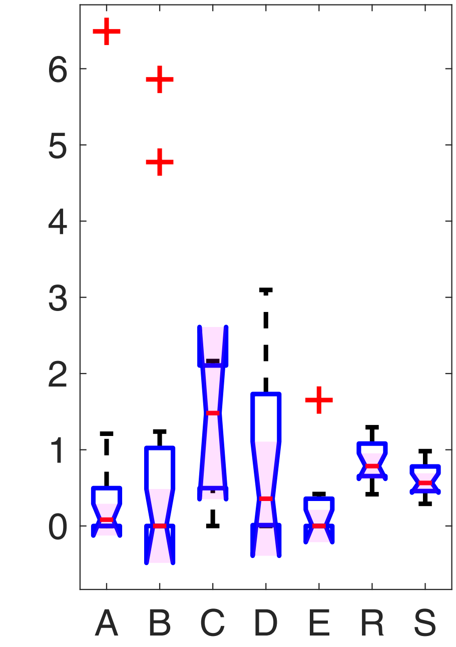


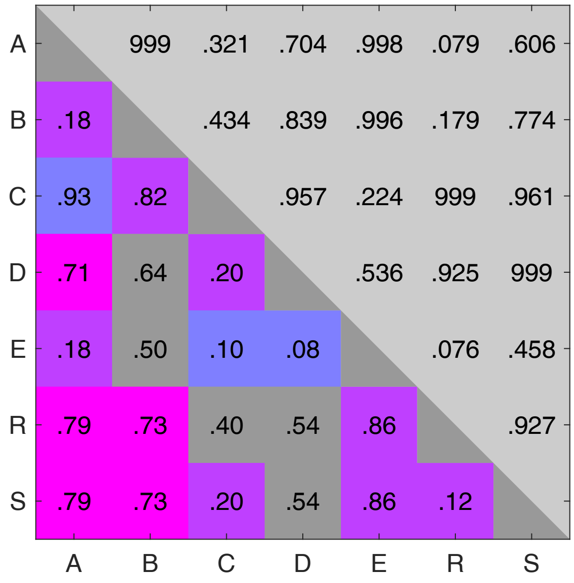

**Heatmap Analysis of Box 68**

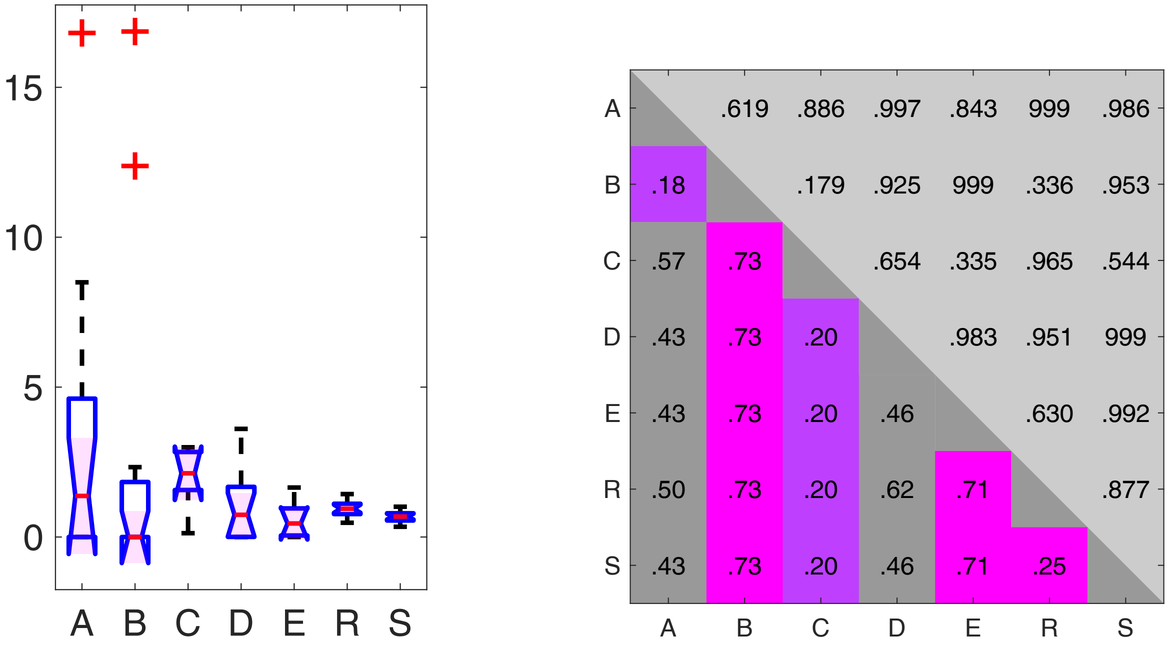


**Heatmap Analysis of Box 69**

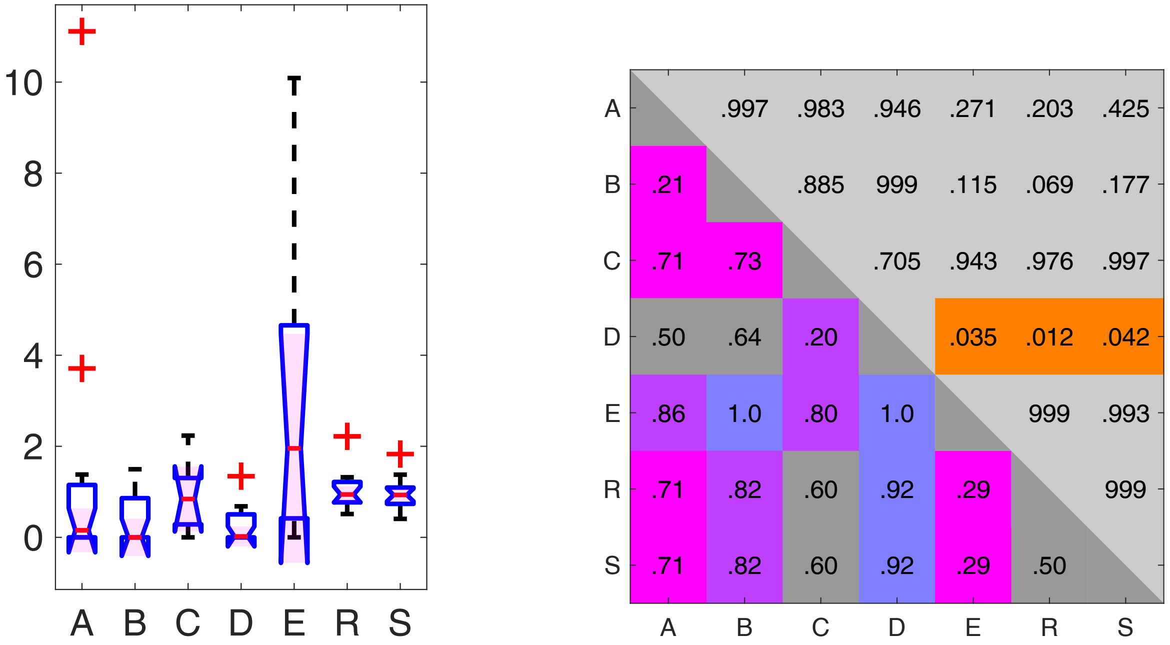

**Heatmap Analysis of Box 6A**

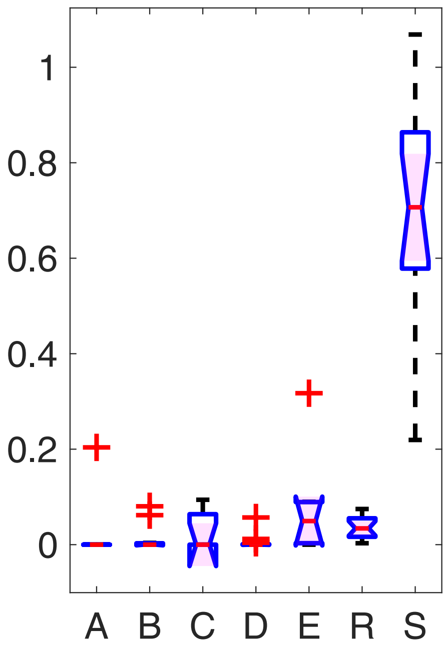


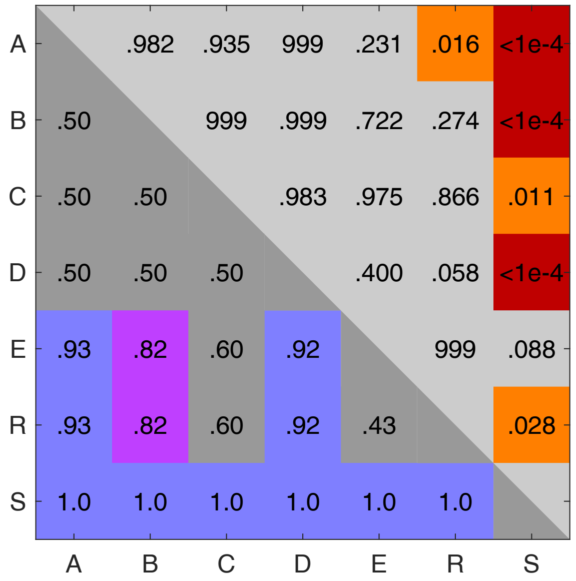


**Heatmap Analysis of Box 6B**

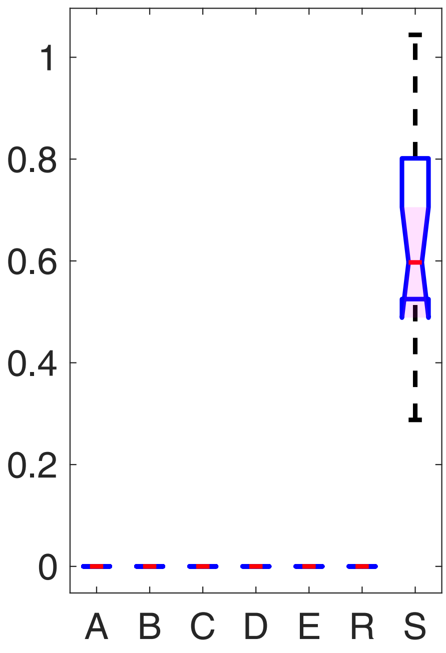


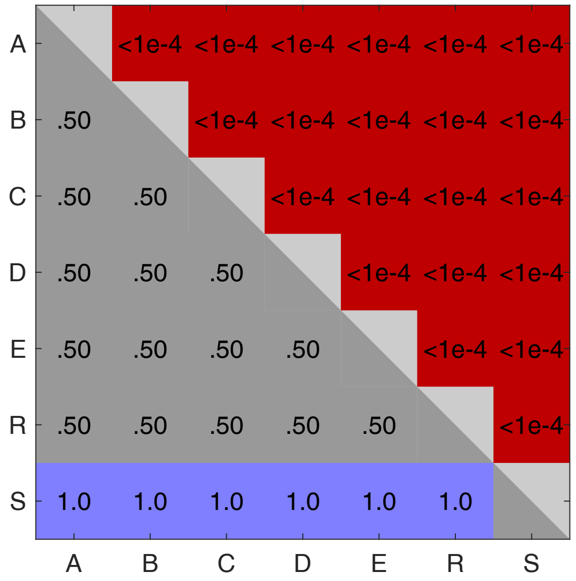

**Heatmap Analysis of Box 6C**

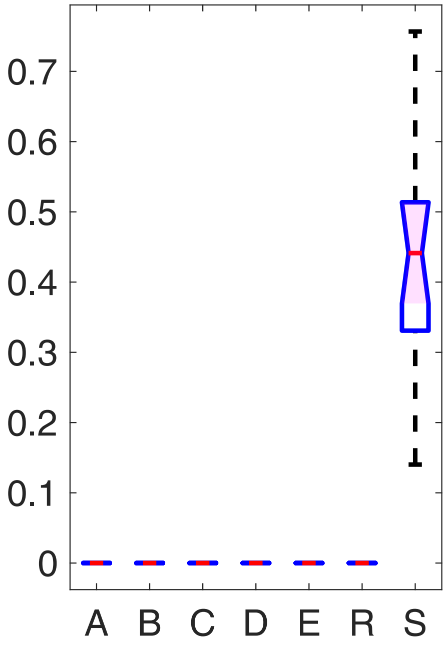


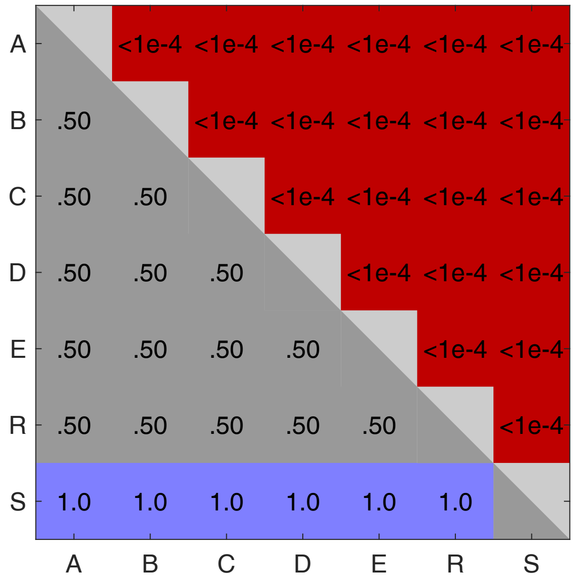


**Heatmap Analysis of Box 6D**

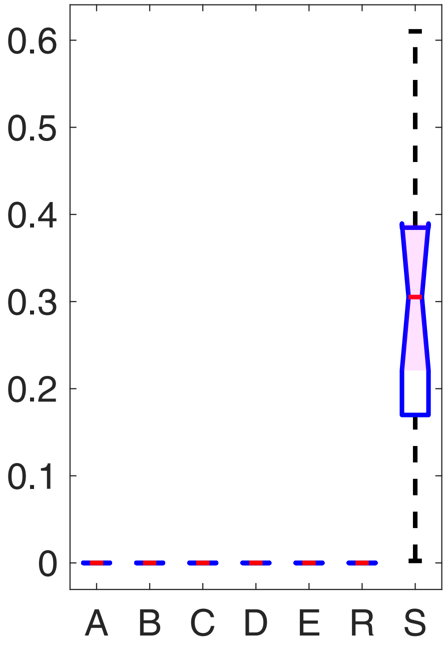


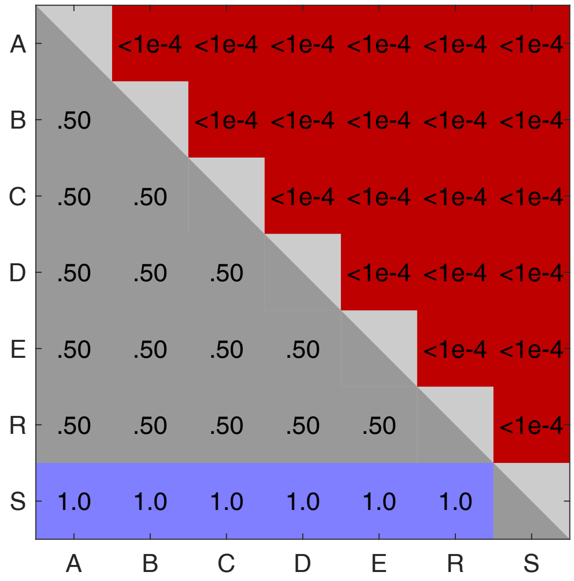

**Heatmap Analysis of Box 6E**

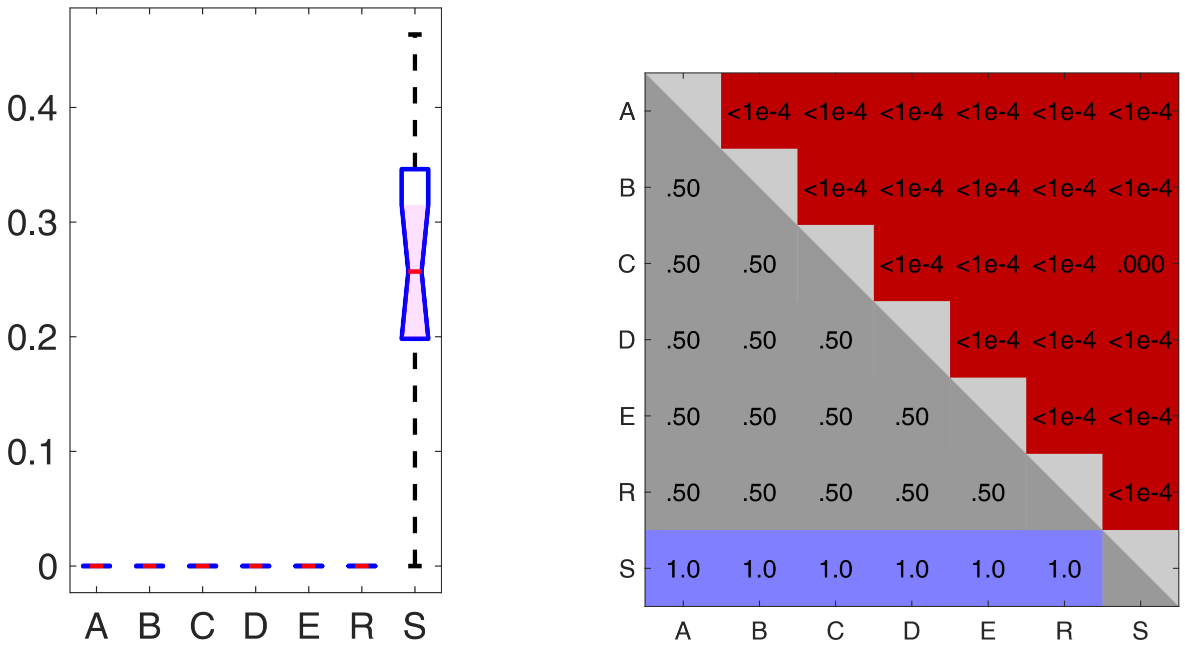


**Heatmap Analysis of Box 6F**

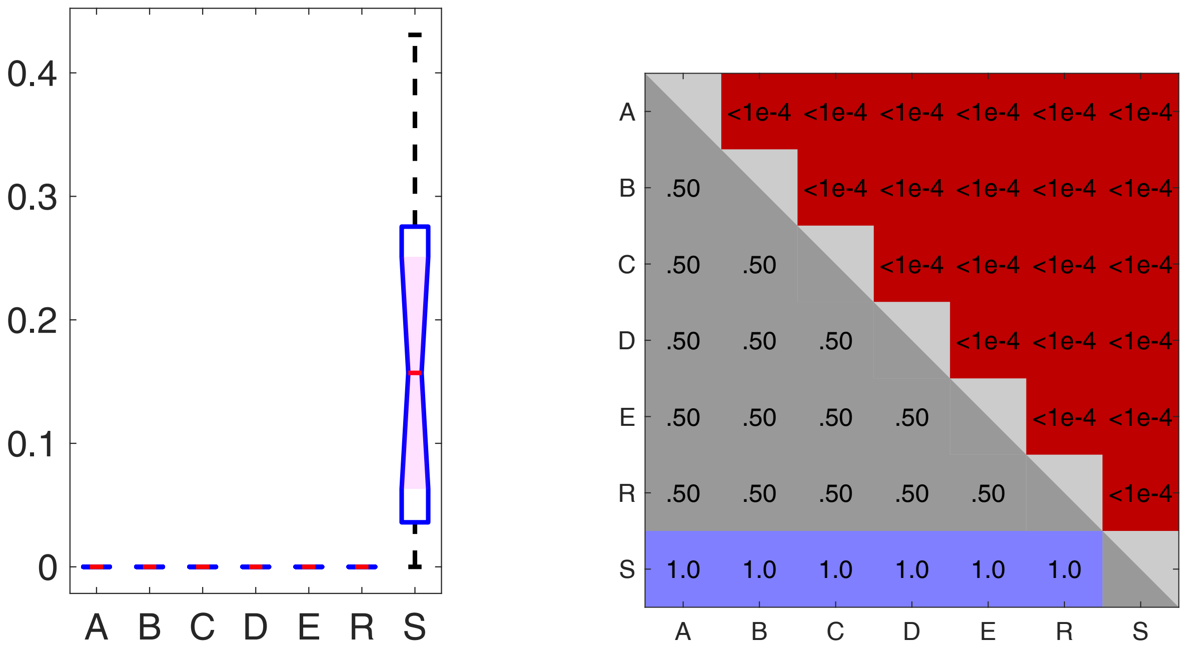

**Heatmap Analysis of Box 6G**

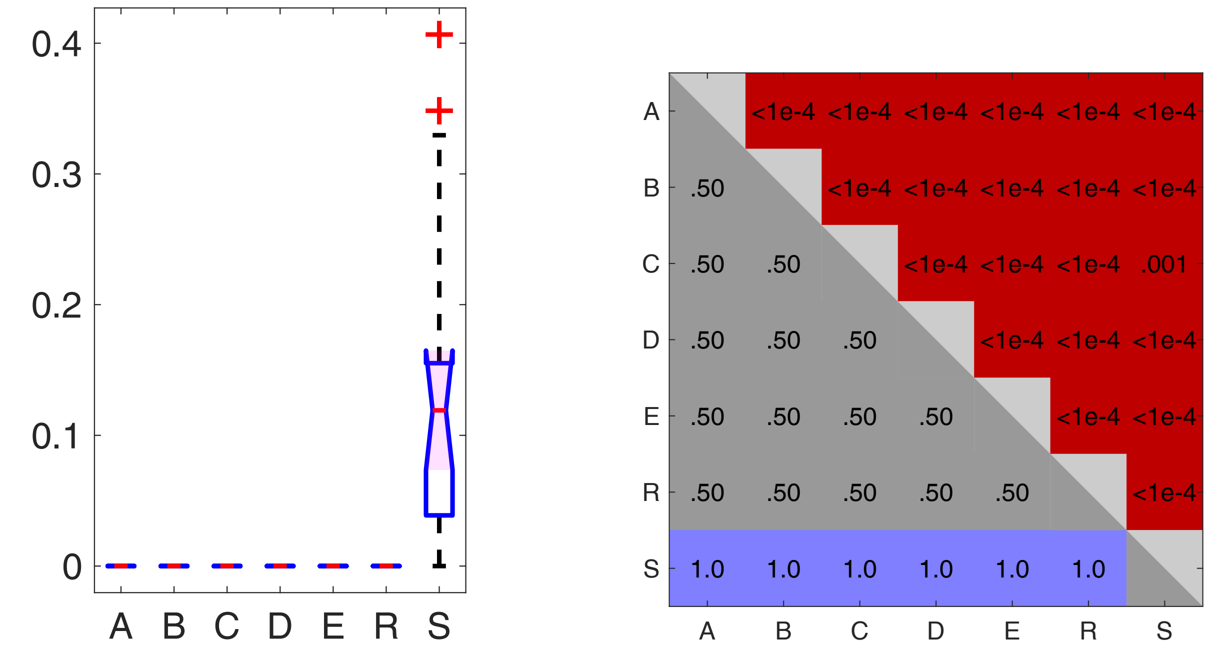


**Heatmap Analysis of Box 6H**

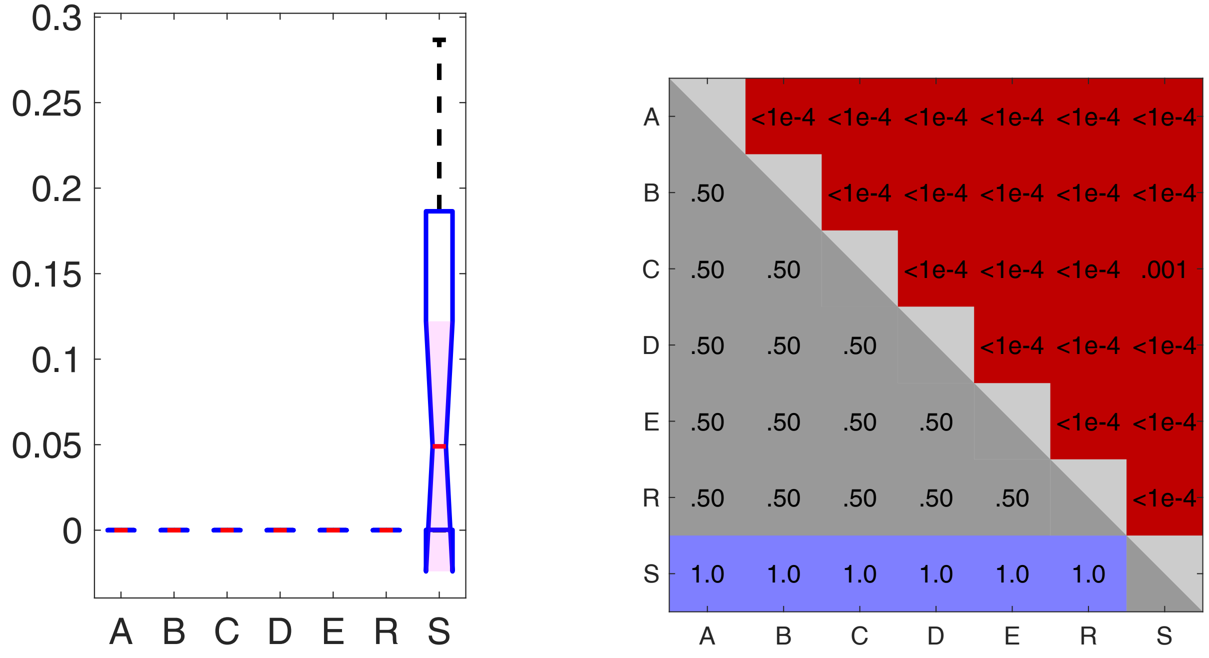

**Heatmap Analysis of Box 70**

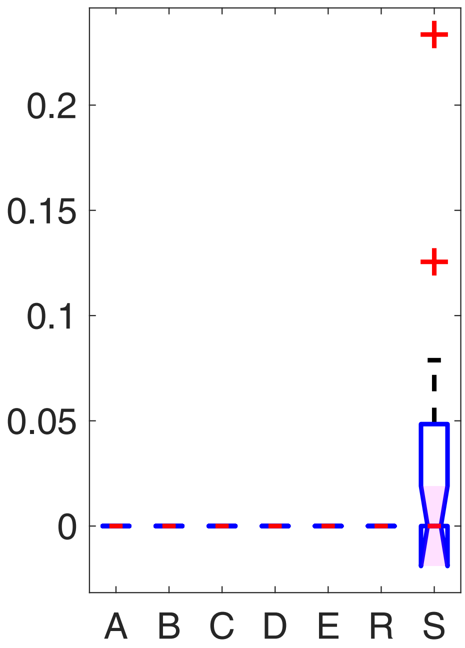


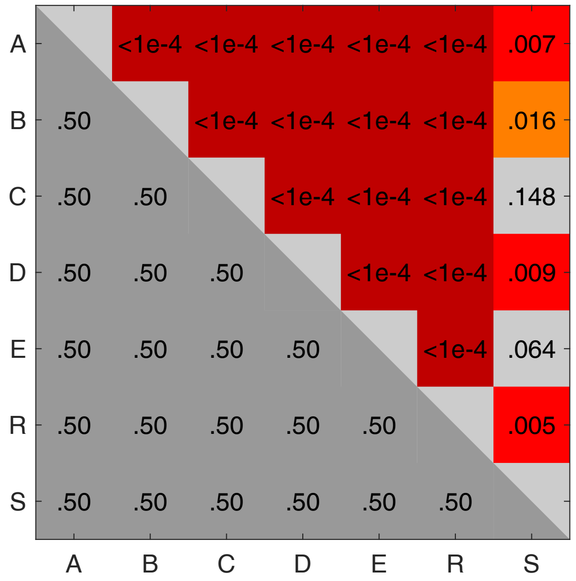


**Heatmap Analysis of Box 71**

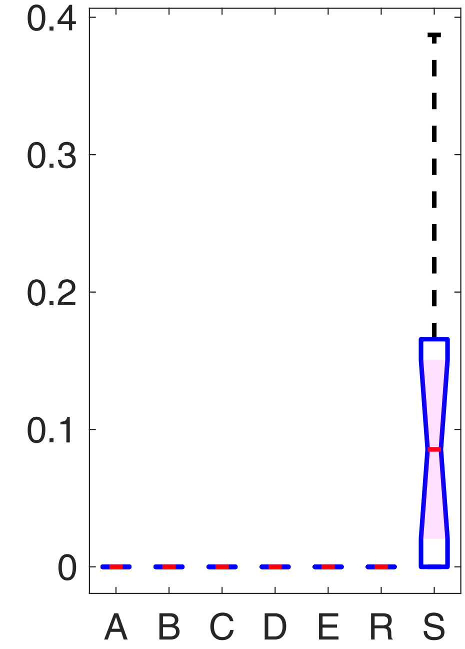


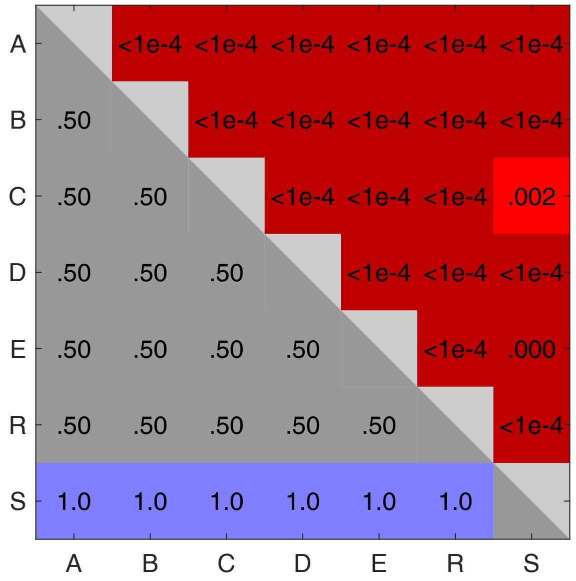

**Heatmap Analysis of Box 72**

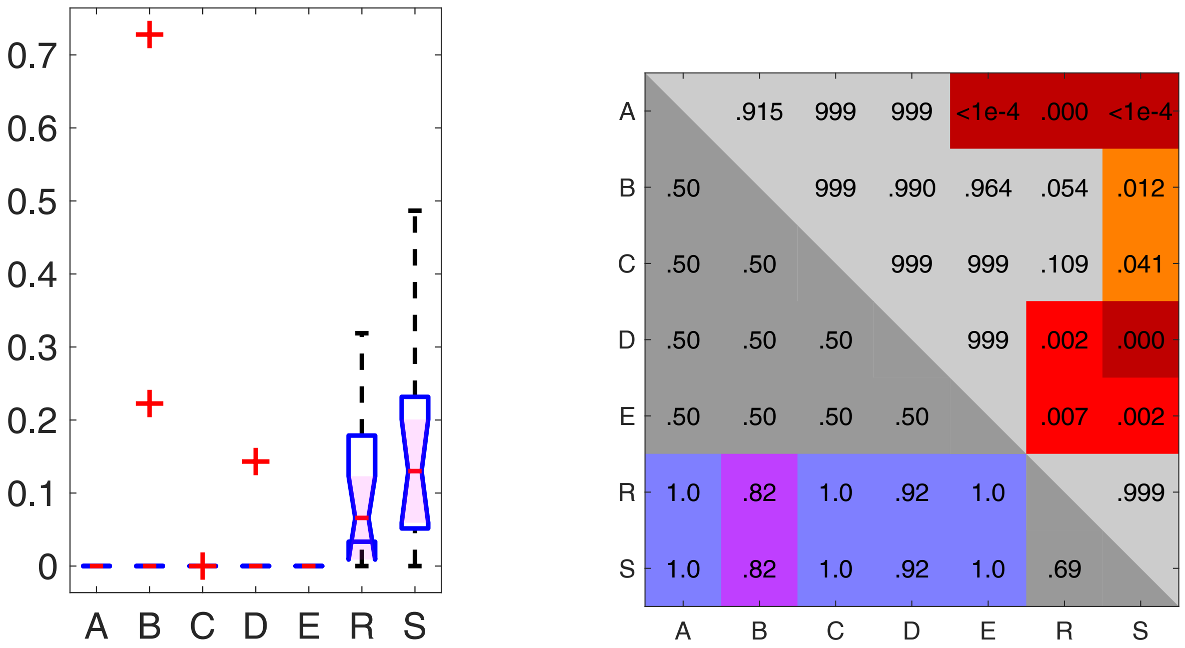


**Heatmap Analysis of Box 73**

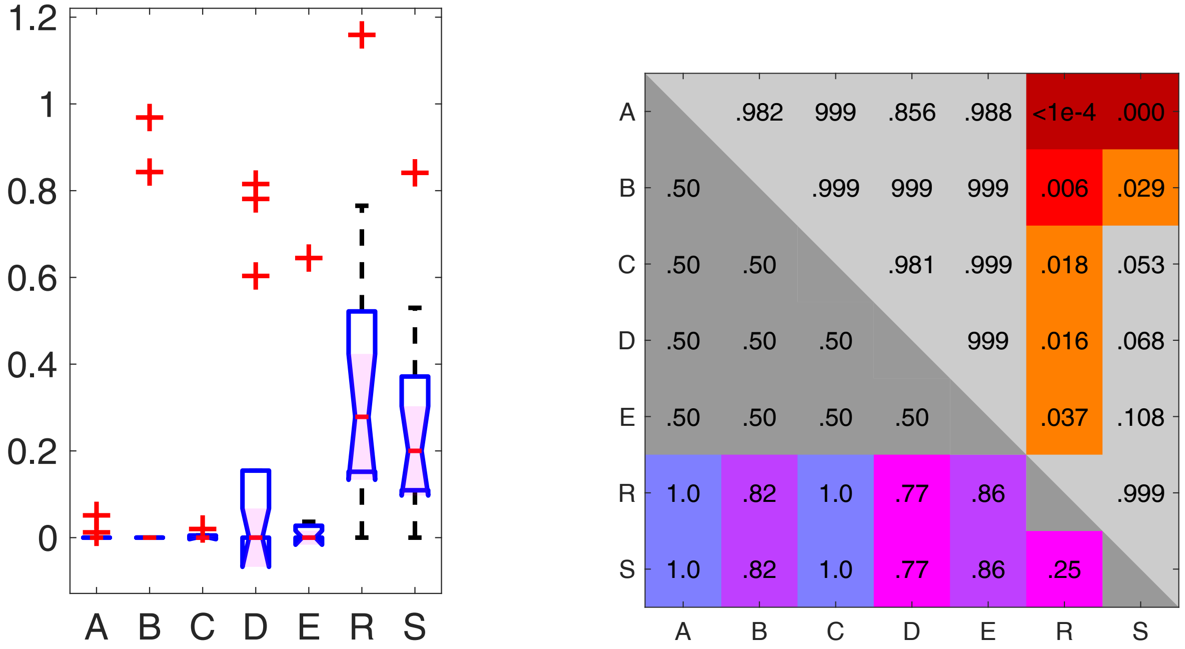

**Heatmap Analysis of Box 74**

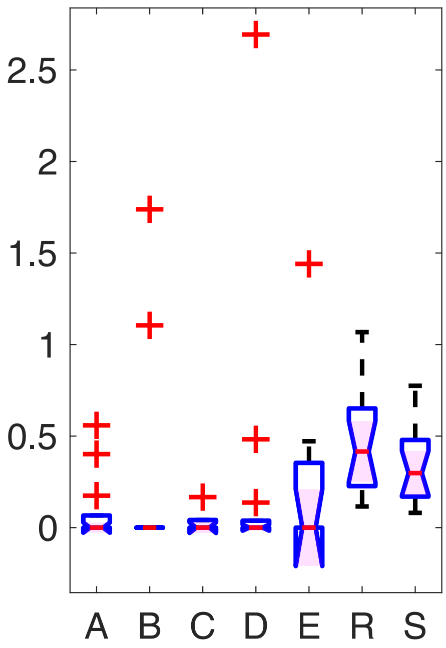


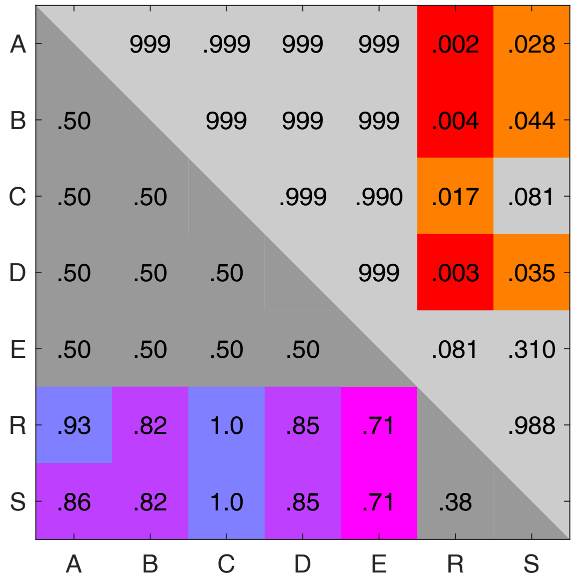


**Heatmap Analysis of Box 75**

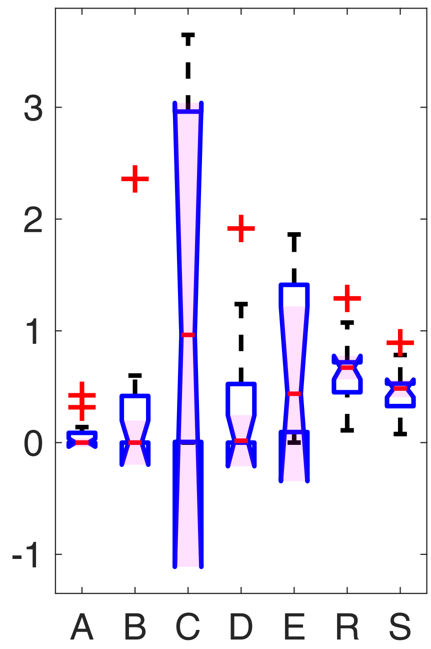


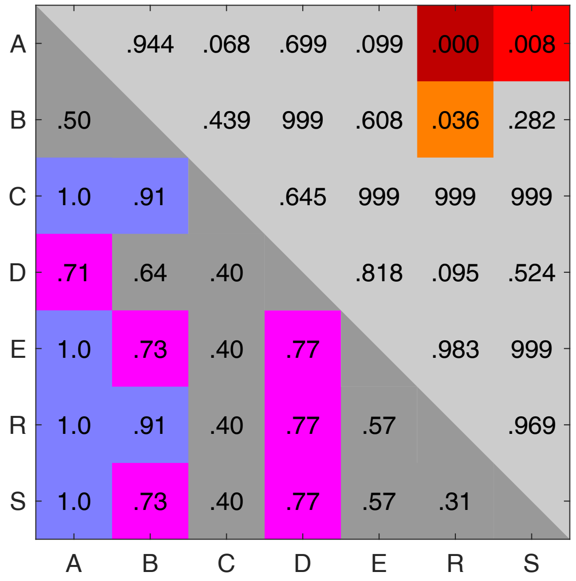

**Heatmap Analysis of Box 76**

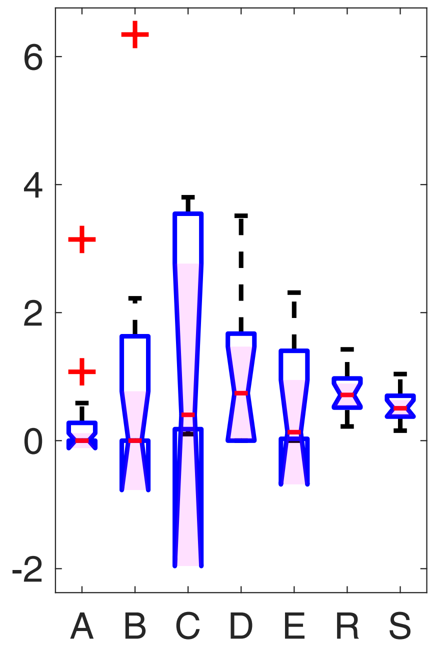


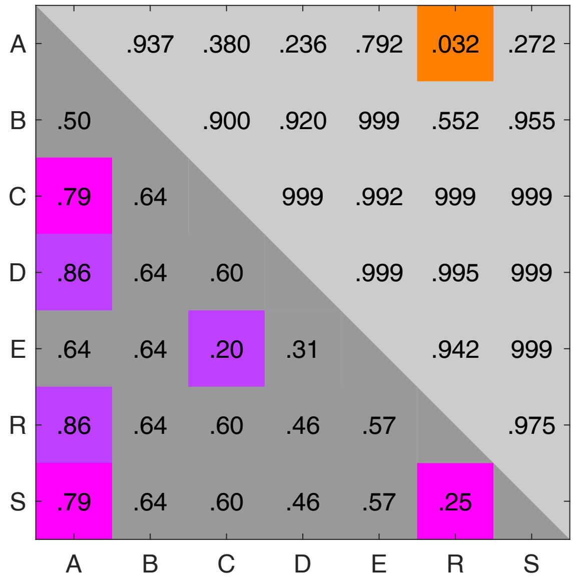


**Heatmap Analysis of Box 77**

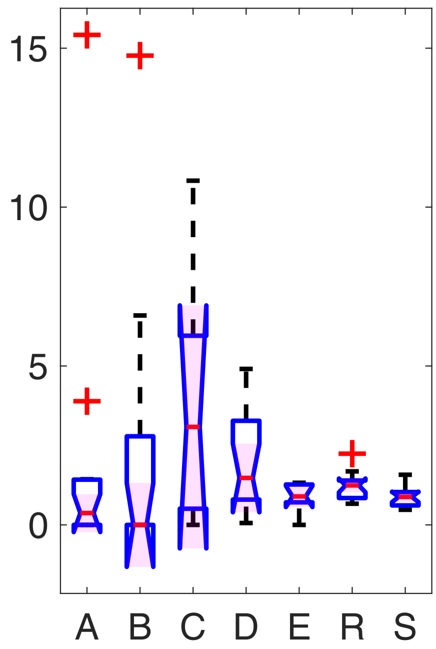


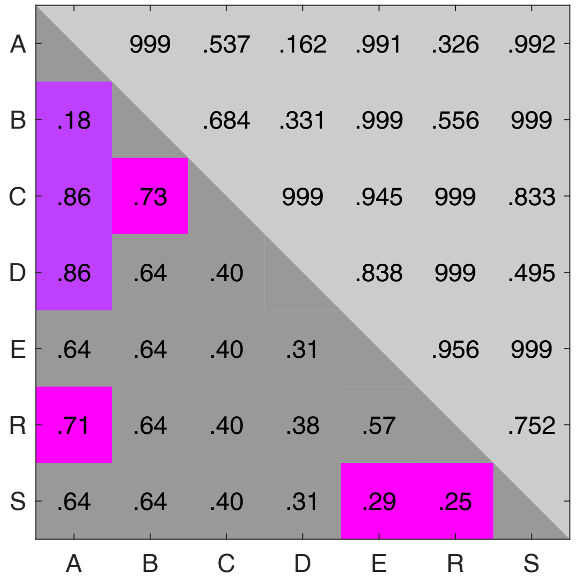

**Heatmap Analysis of Box 78**

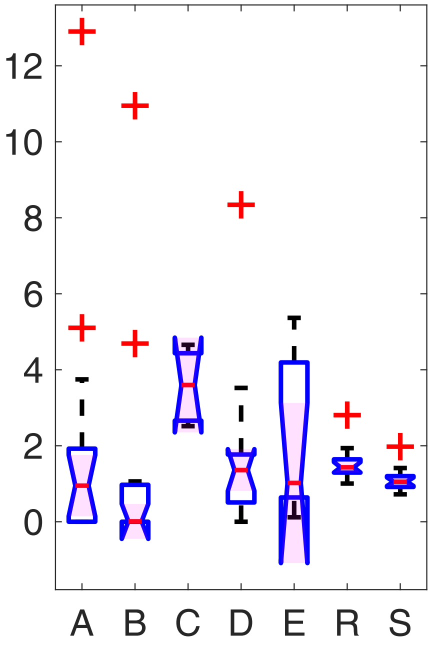


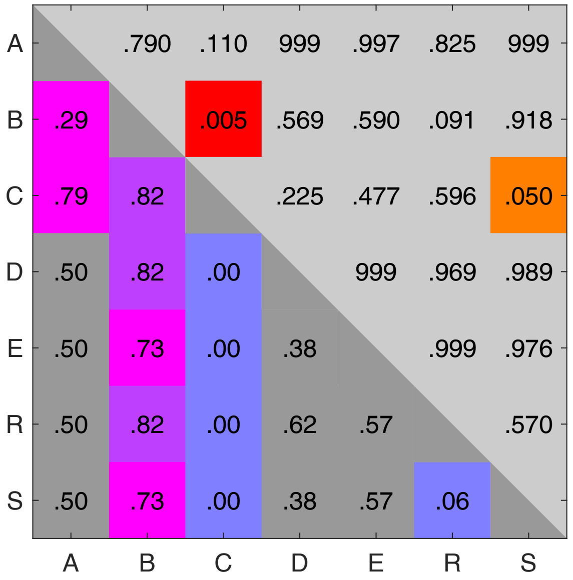


**Heatmap Analysis of Box 79**

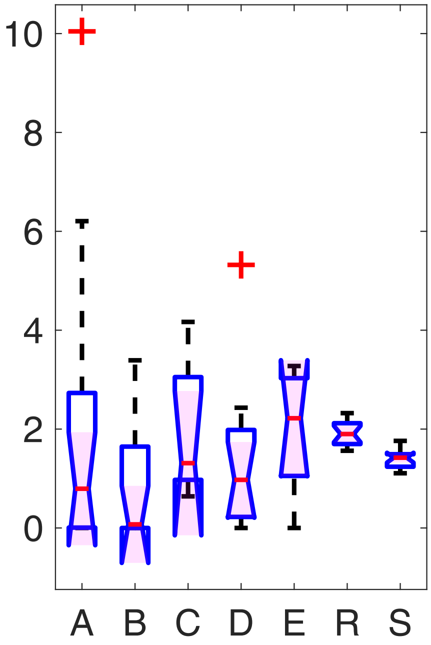


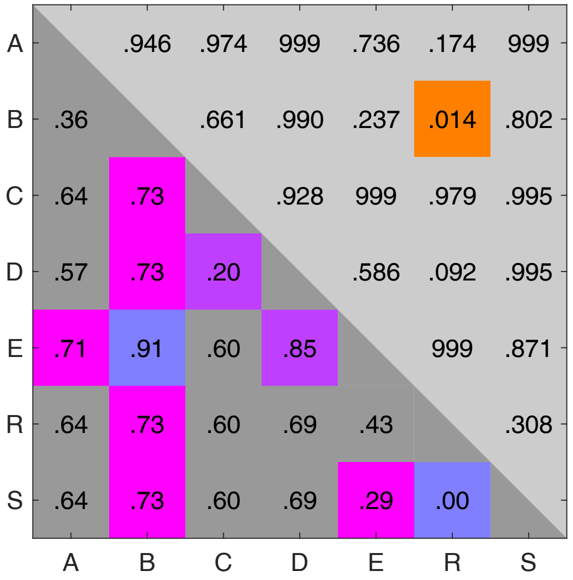

**Heatmap Analysis of Box 7A**

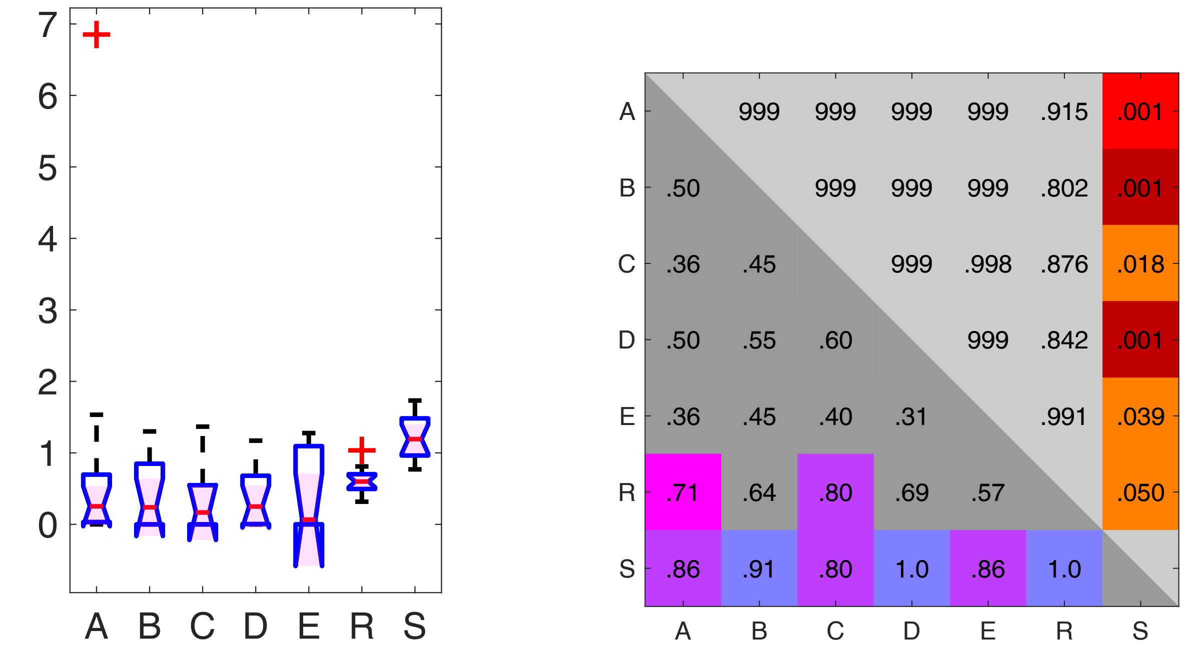


**Heatmap Analysis of Box 7B**

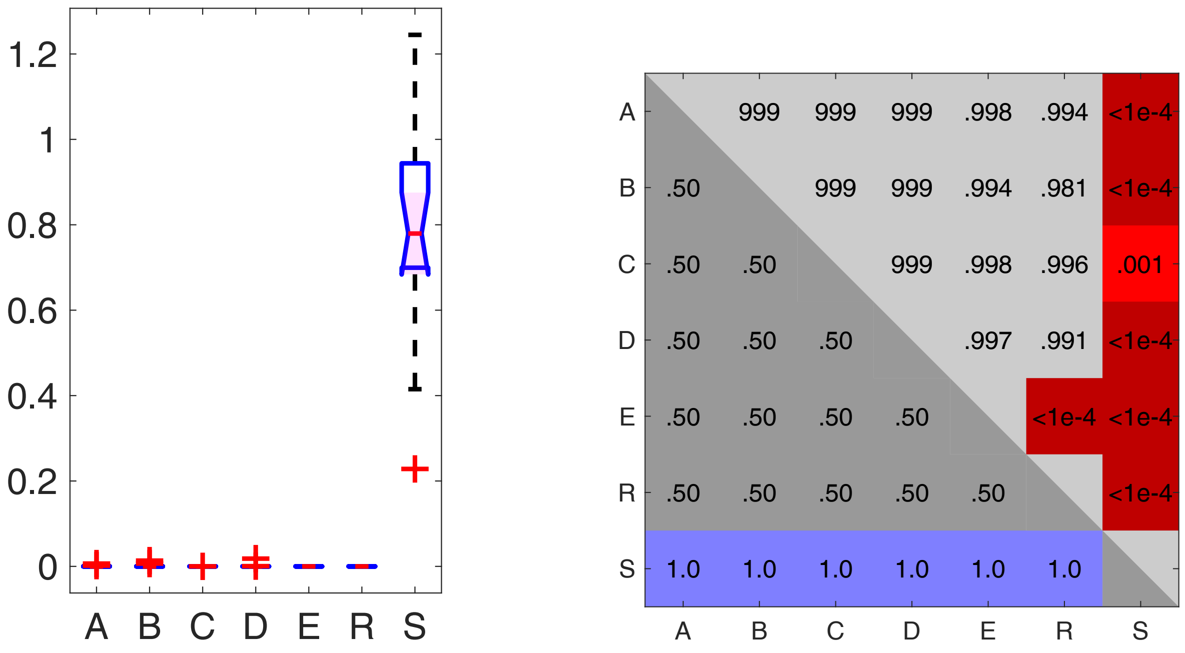

**Heatmap Analysis of Box 7C**

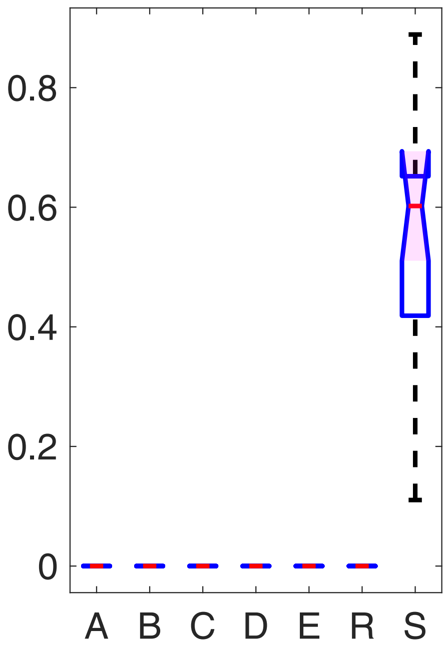


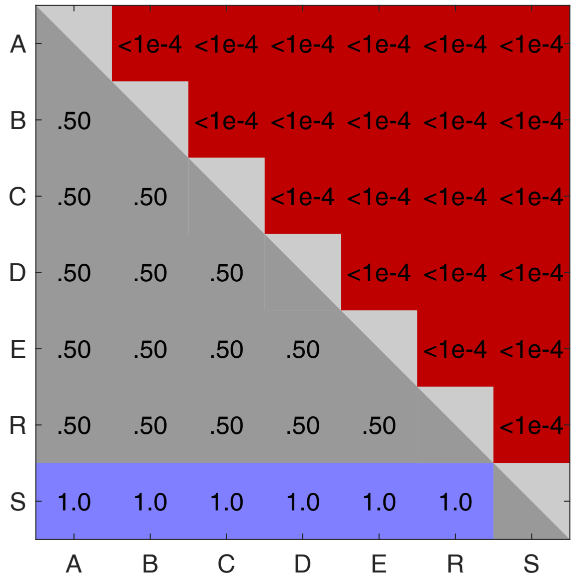


**Heatmap Analysis of Box 7D**

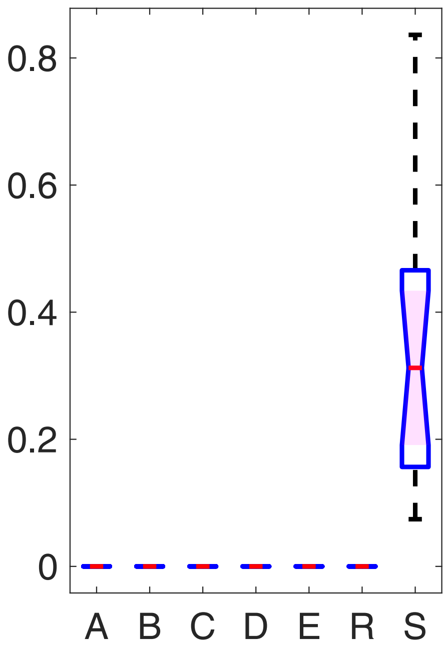


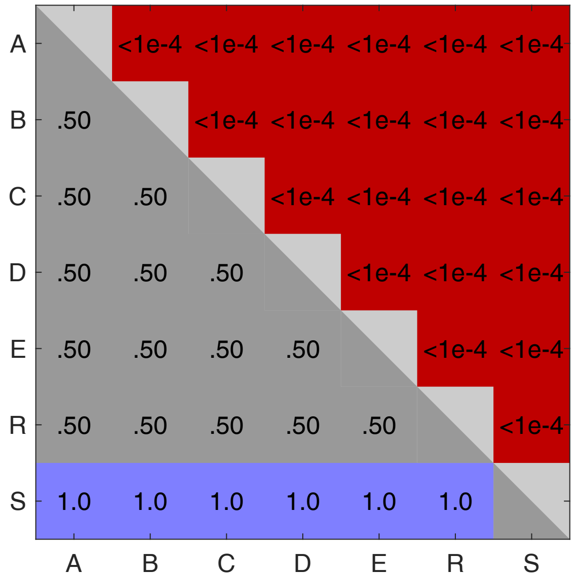

**Heatmap Analysis of Box 7E**

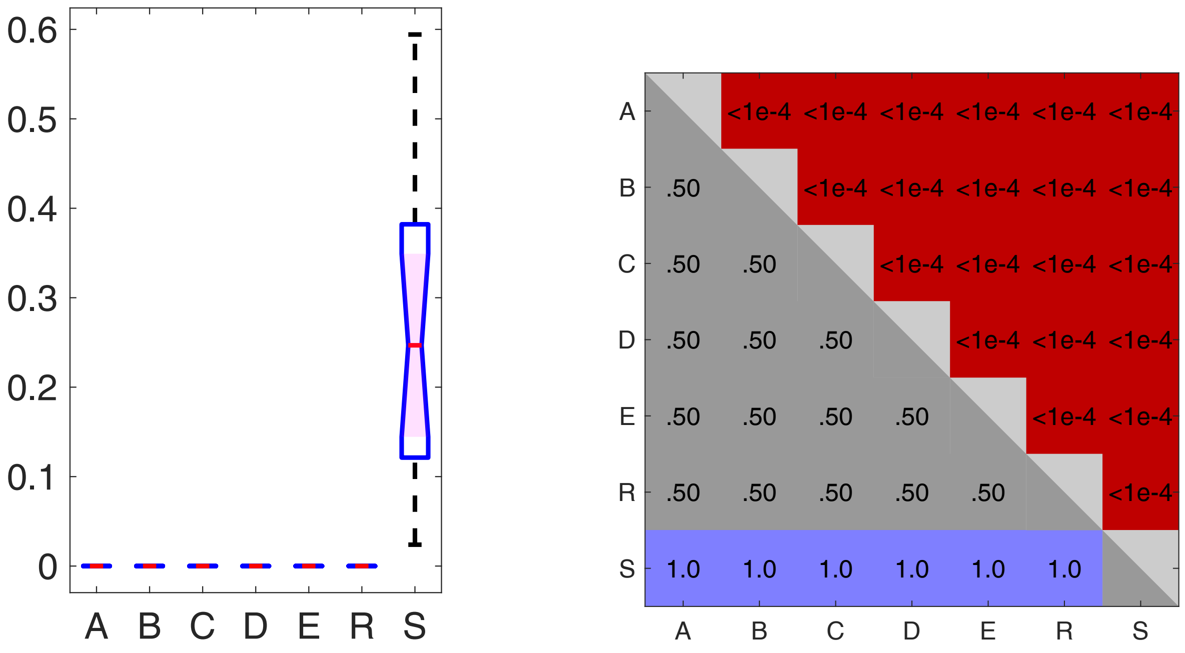


**Heatmap Analysis of Box 7F**

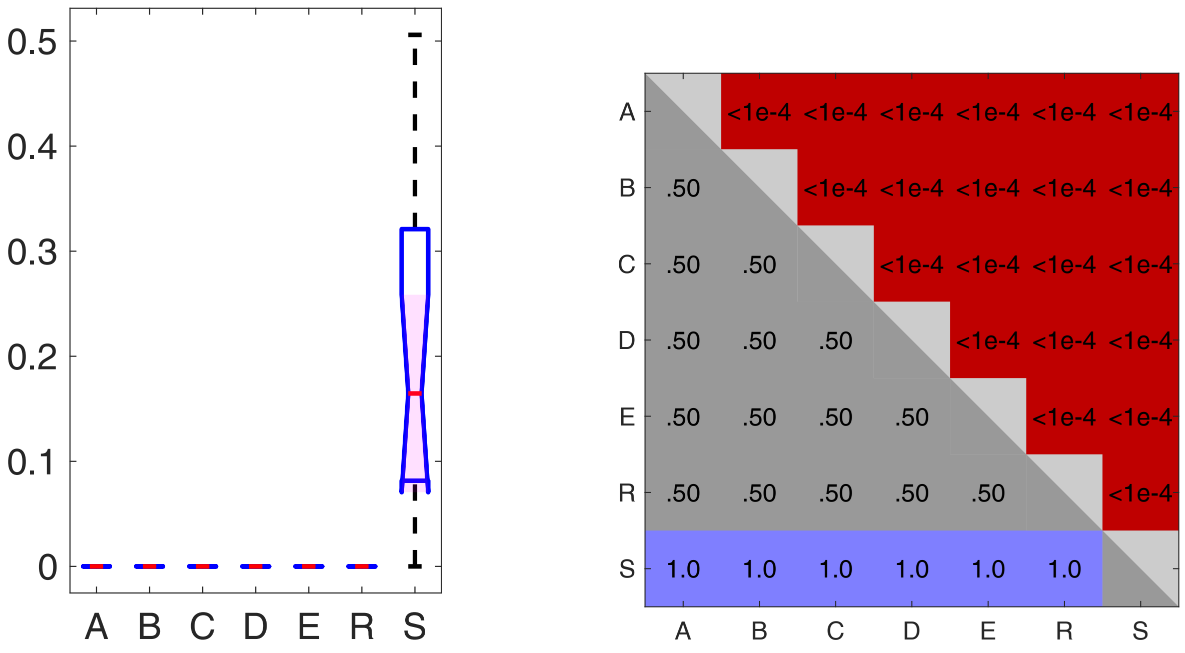

**Heatmap Analysis of Box 7G**

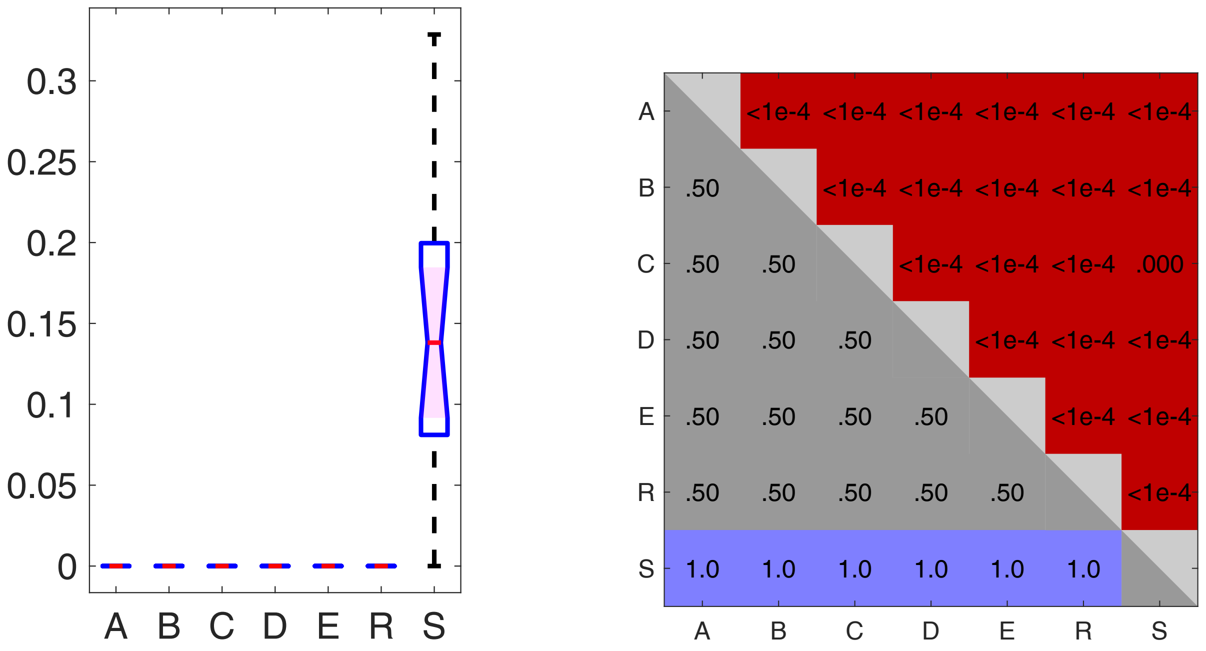


**Heatmap Analysis of Box 7H**

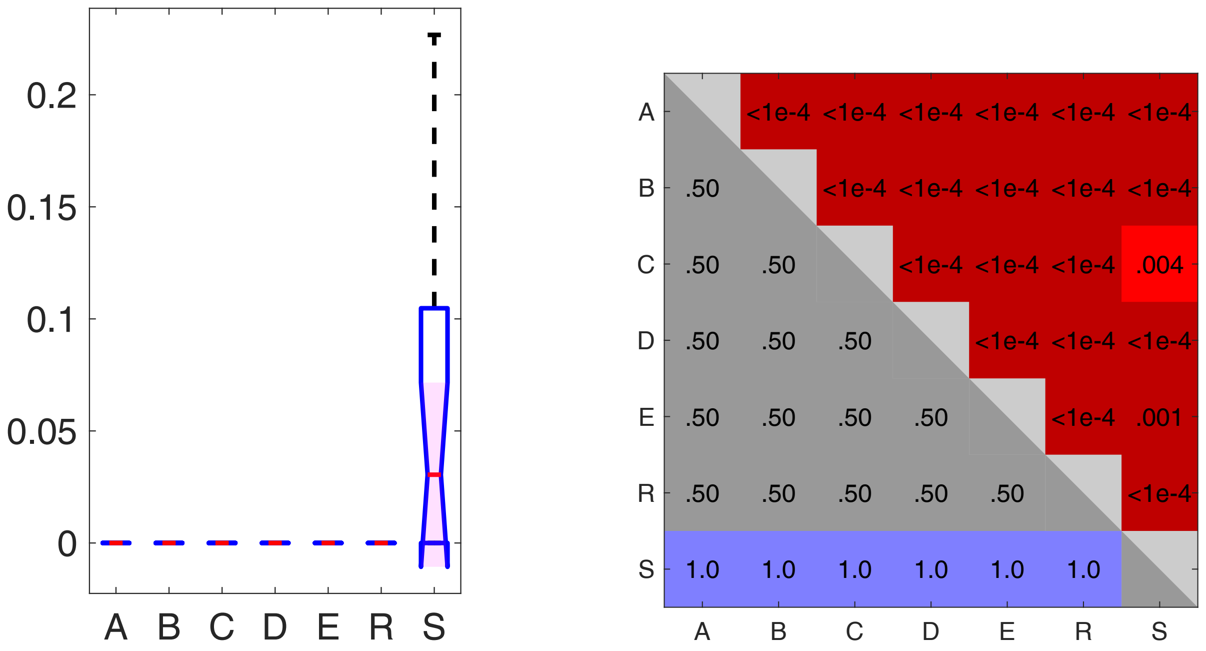

**Heatmap Analysis of Box 80**

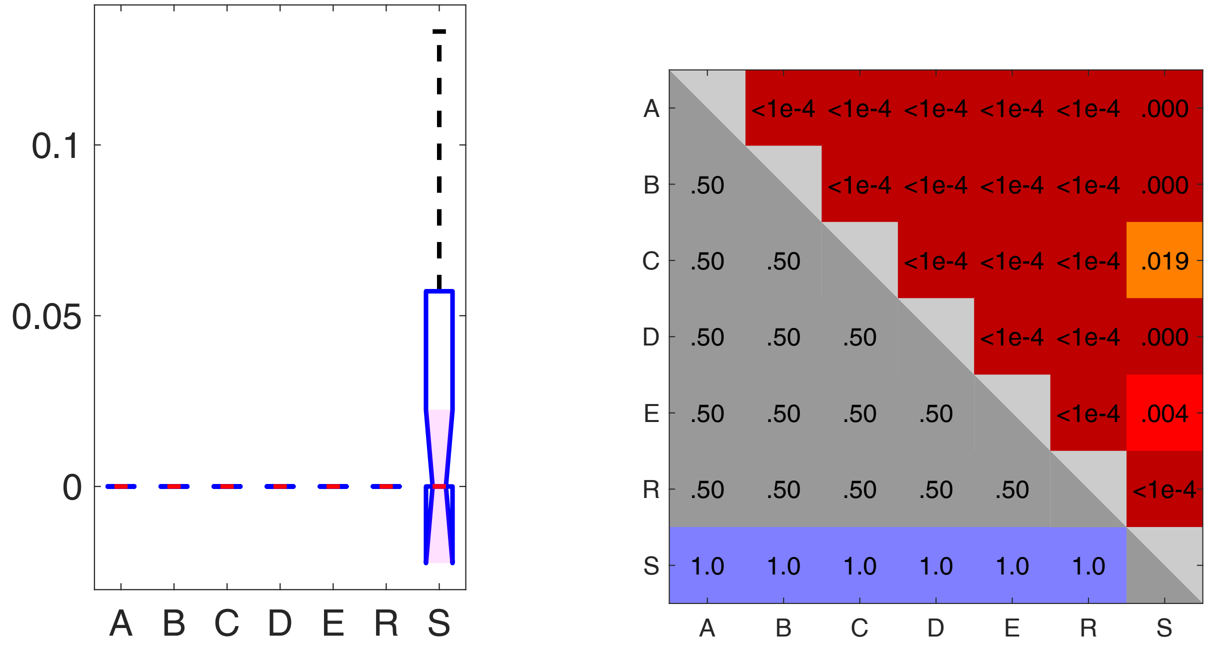


**Heatmap Analysis of Box 81**

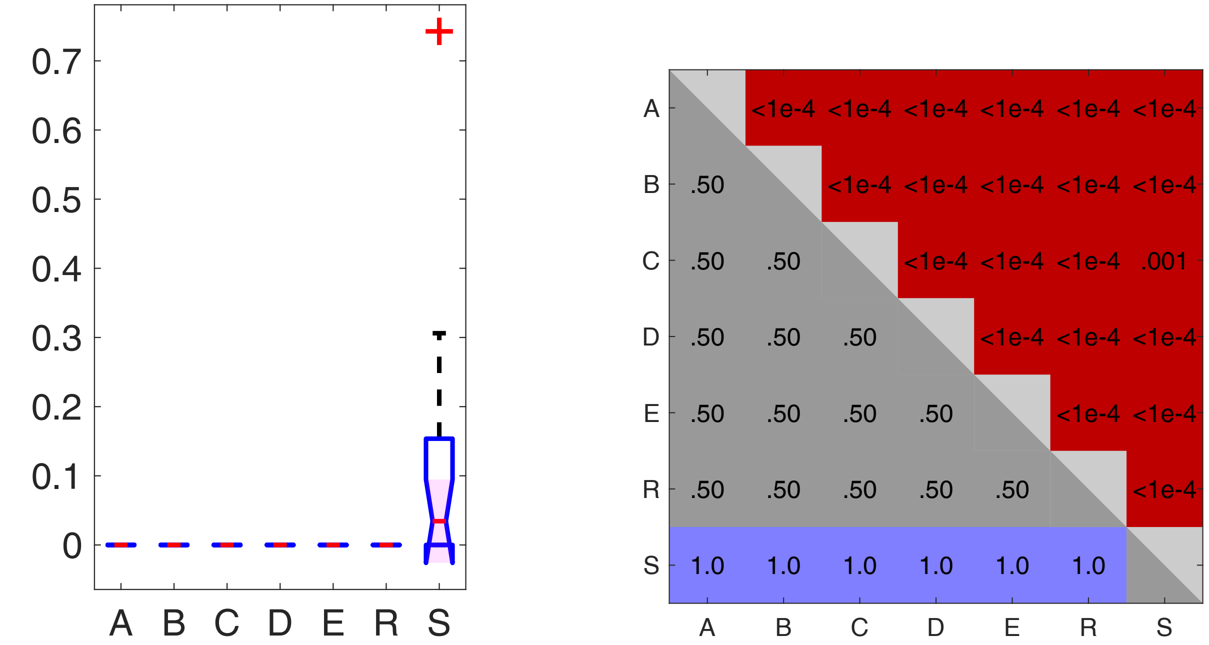

**Heatmap Analysis of Box 82**

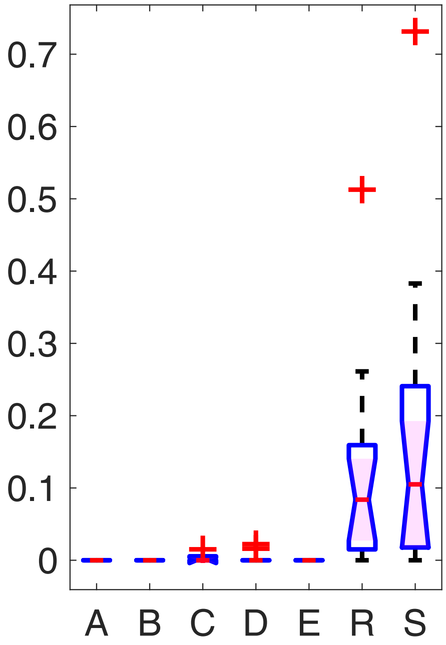


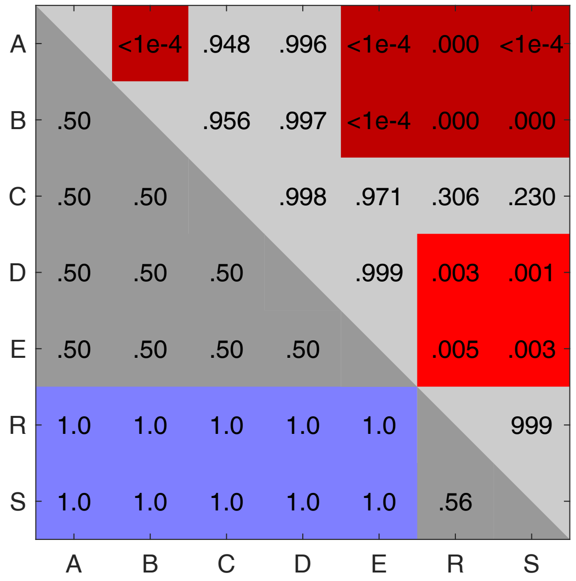


**Heatmap Analysis of Box 83**

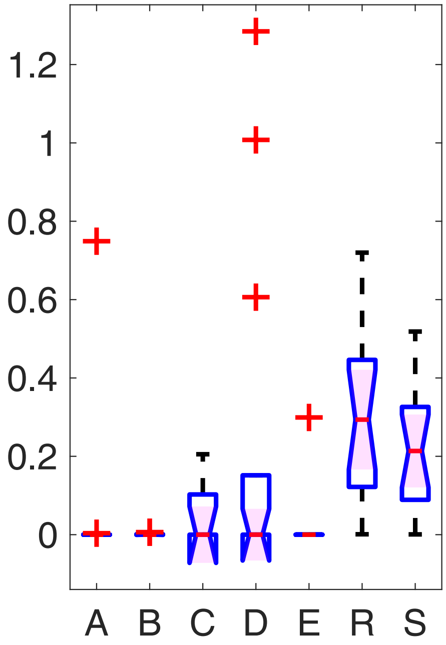


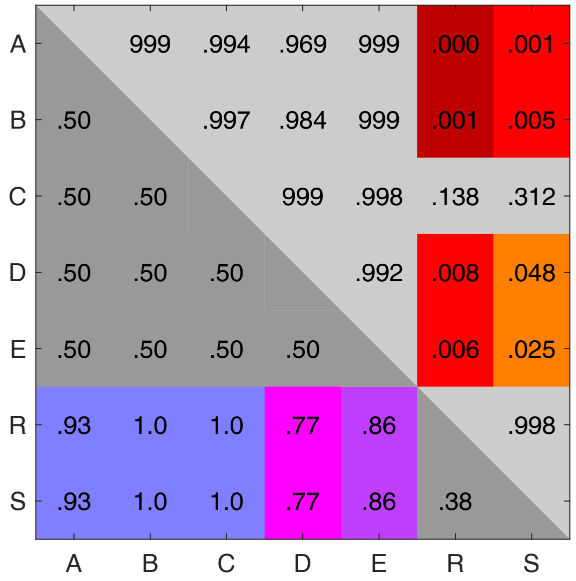

**Heatmap Analysis of Box 84**

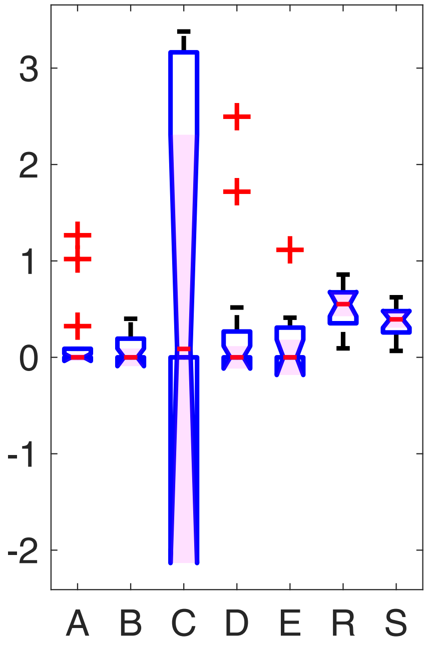


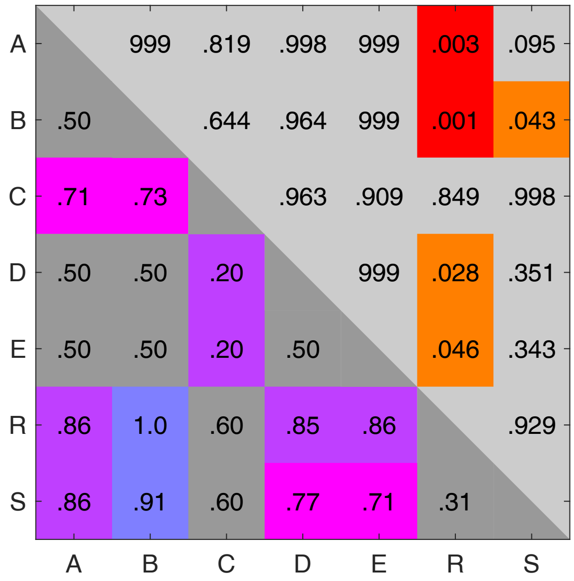


**Heatmap Analysis of Box 85**

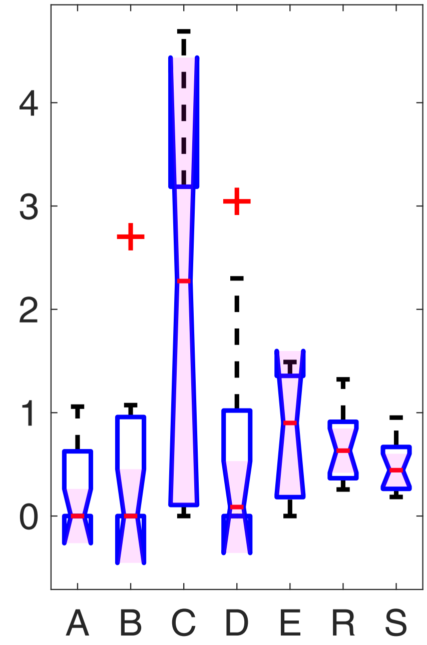


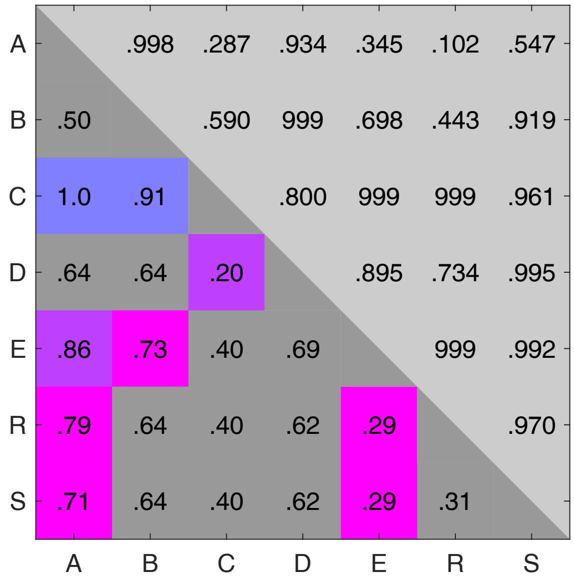

**Heatmap Analysis of Box 86**

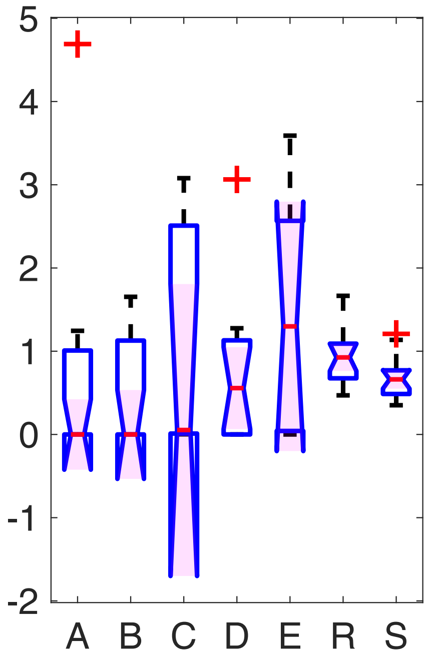


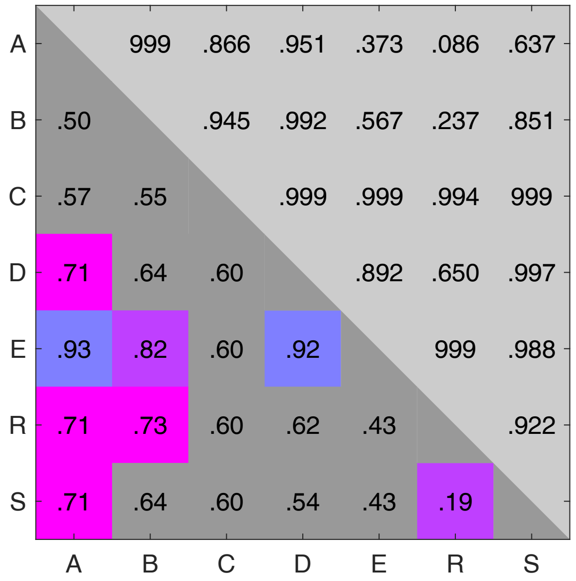


**Heatmap Analysis of Box 87**

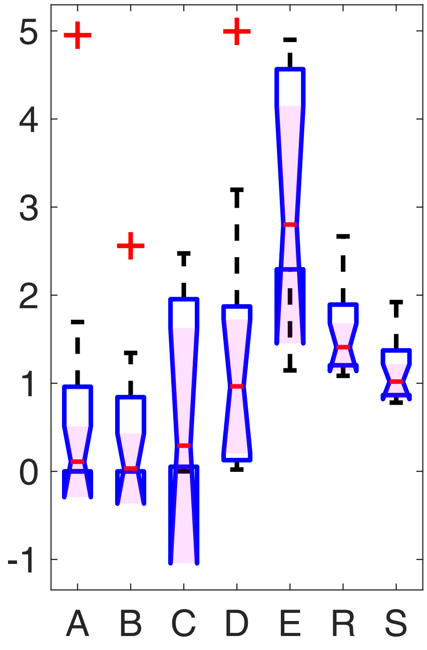


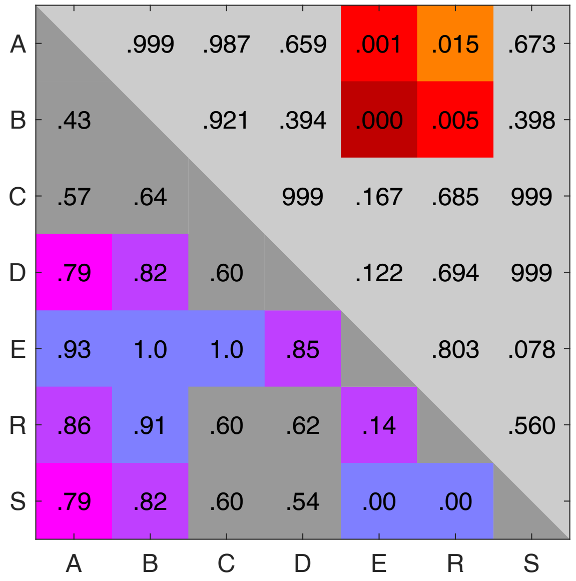

### Heatmap Analysis of Box 88

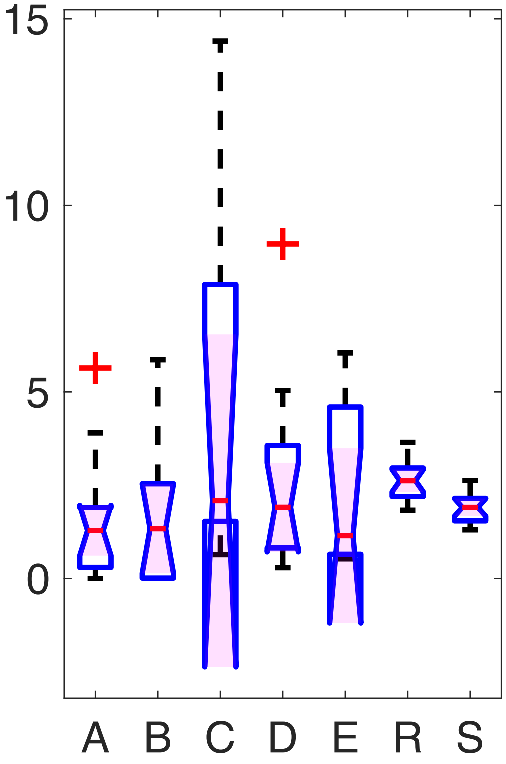


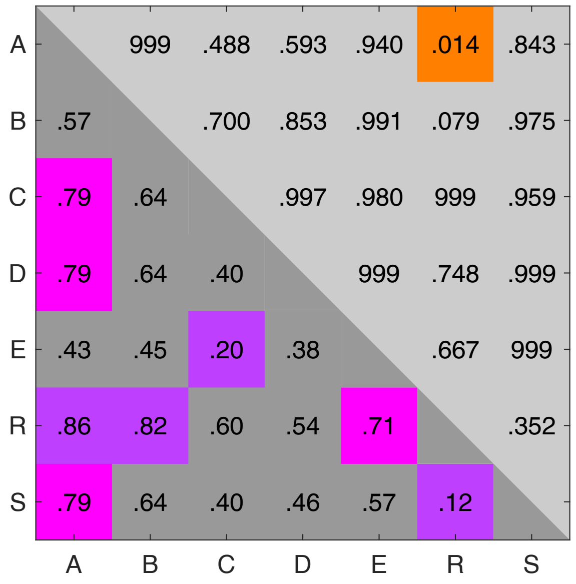


### Heatmap Analysis of Box 89

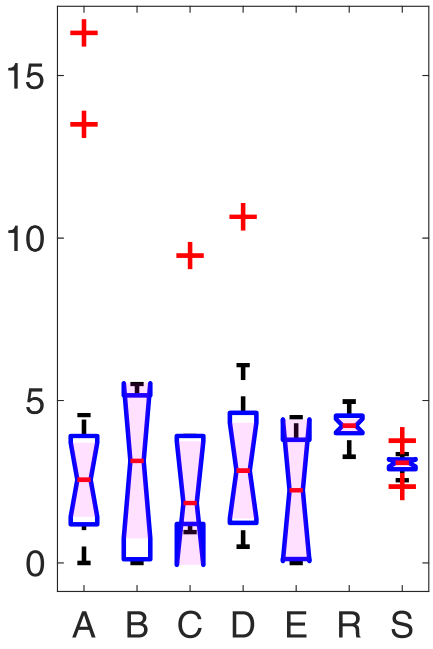


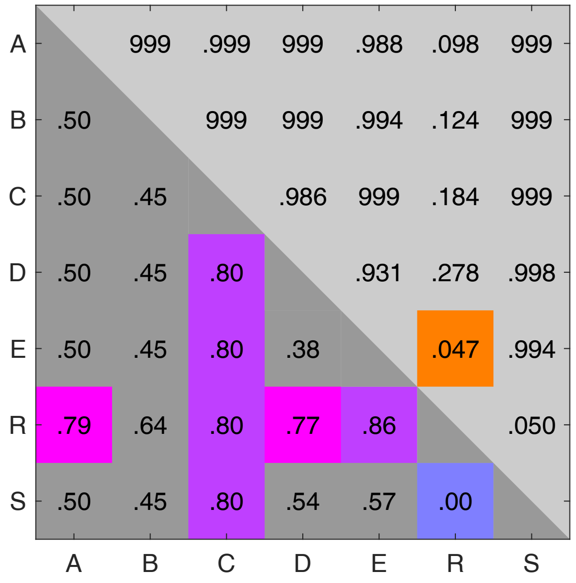

**Heatmap Analysis of Box 8A**

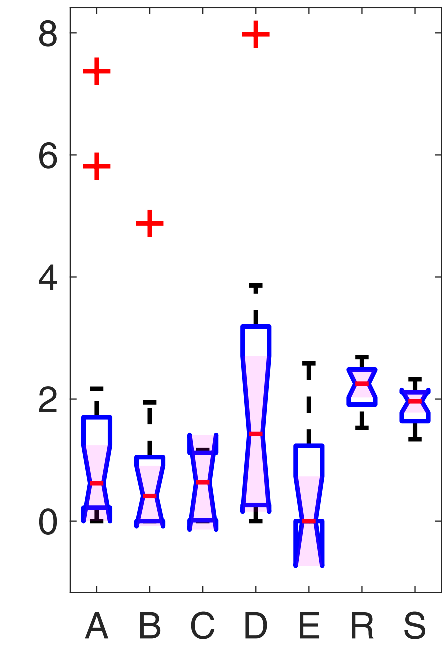


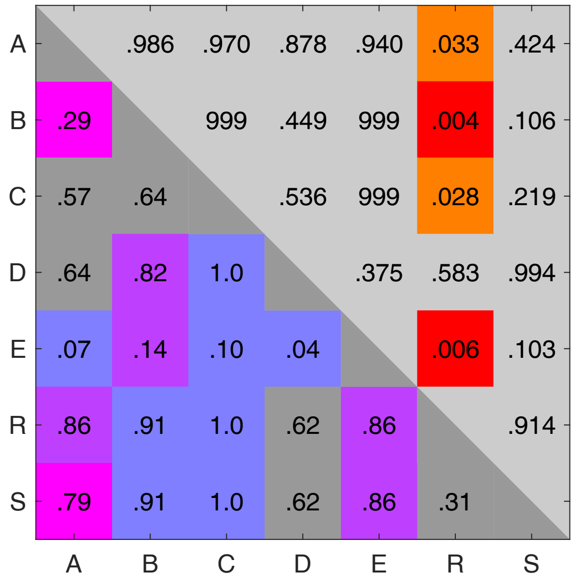


**Heatmap Analysis of Box 8B**

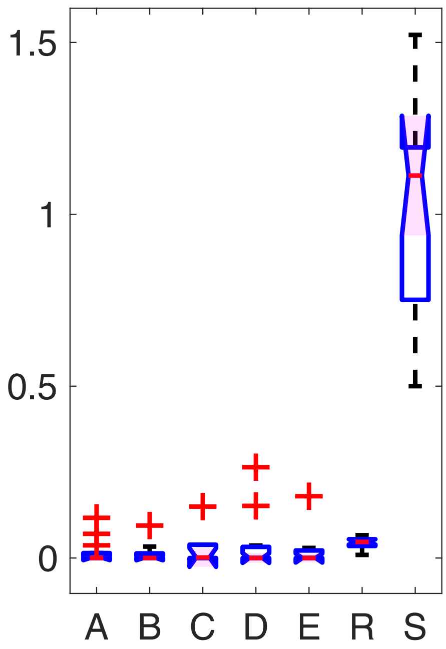


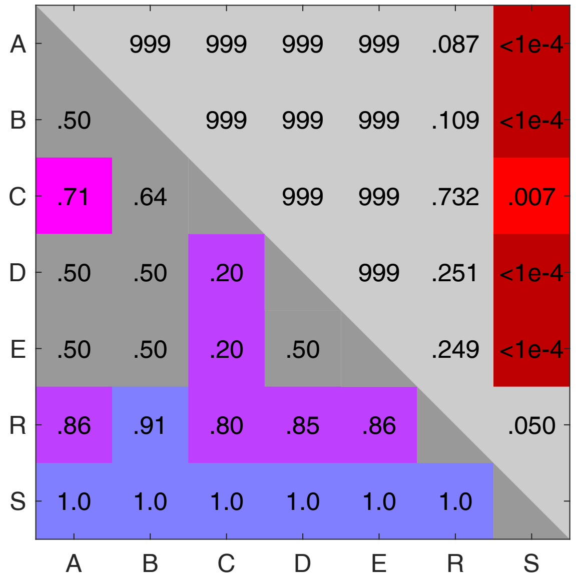

**Heatmap Analysis of Box 8C**

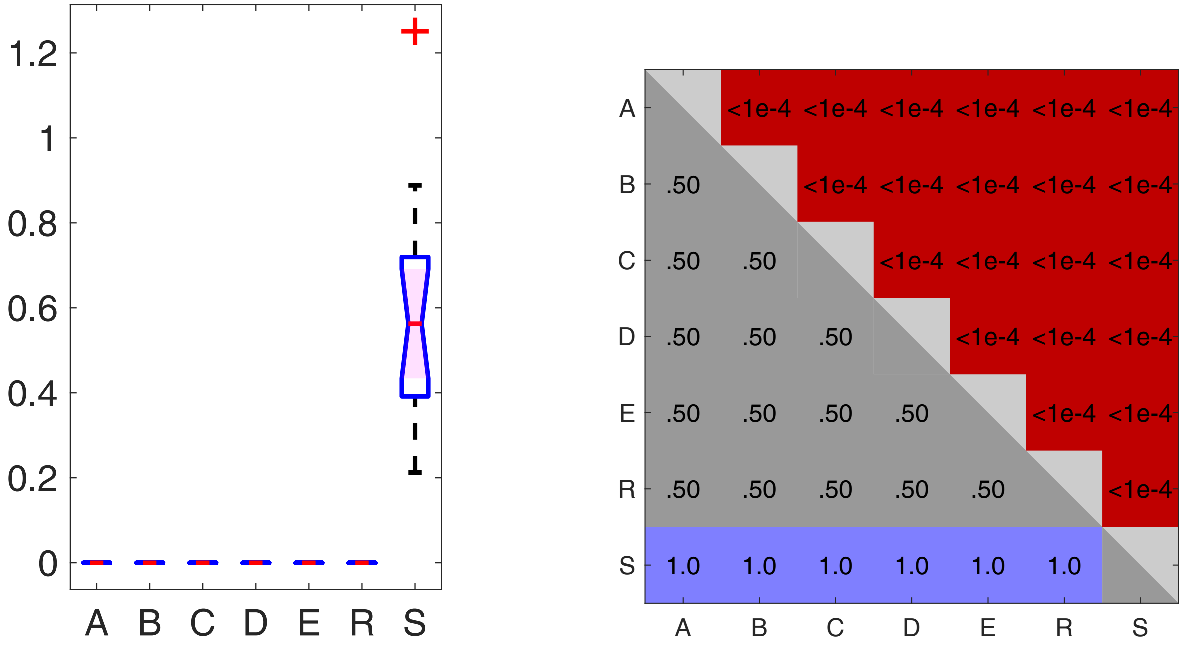


**Heatmap Analysis of Box 8D**

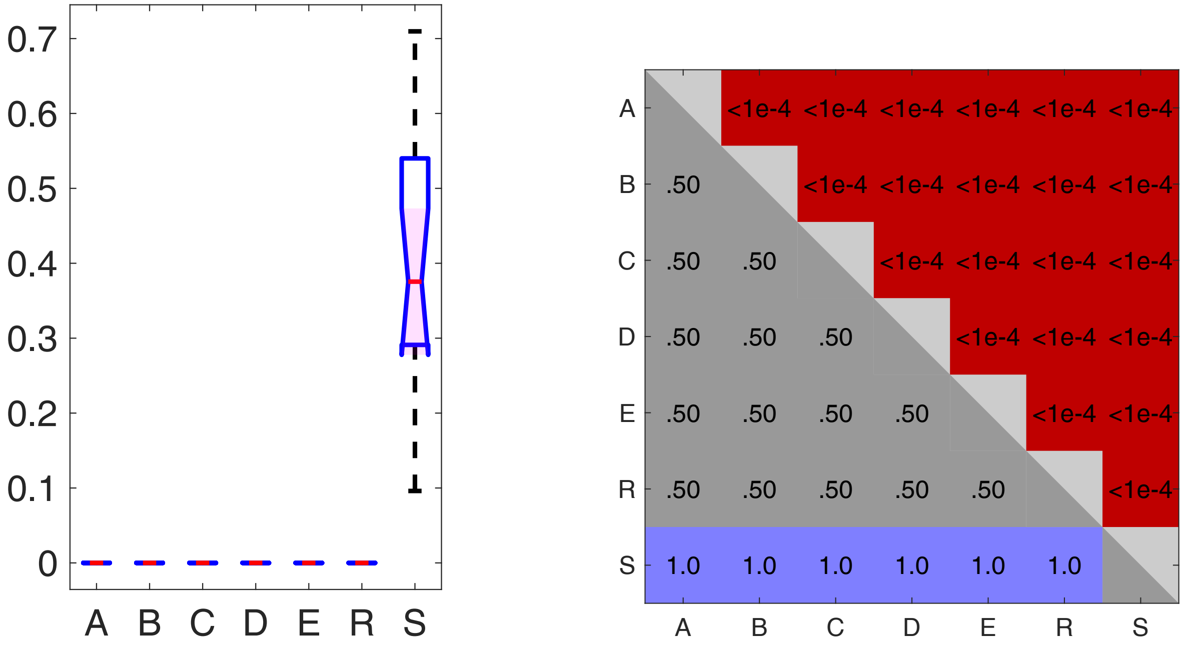

**Heatmap Analysis of Box 8E**

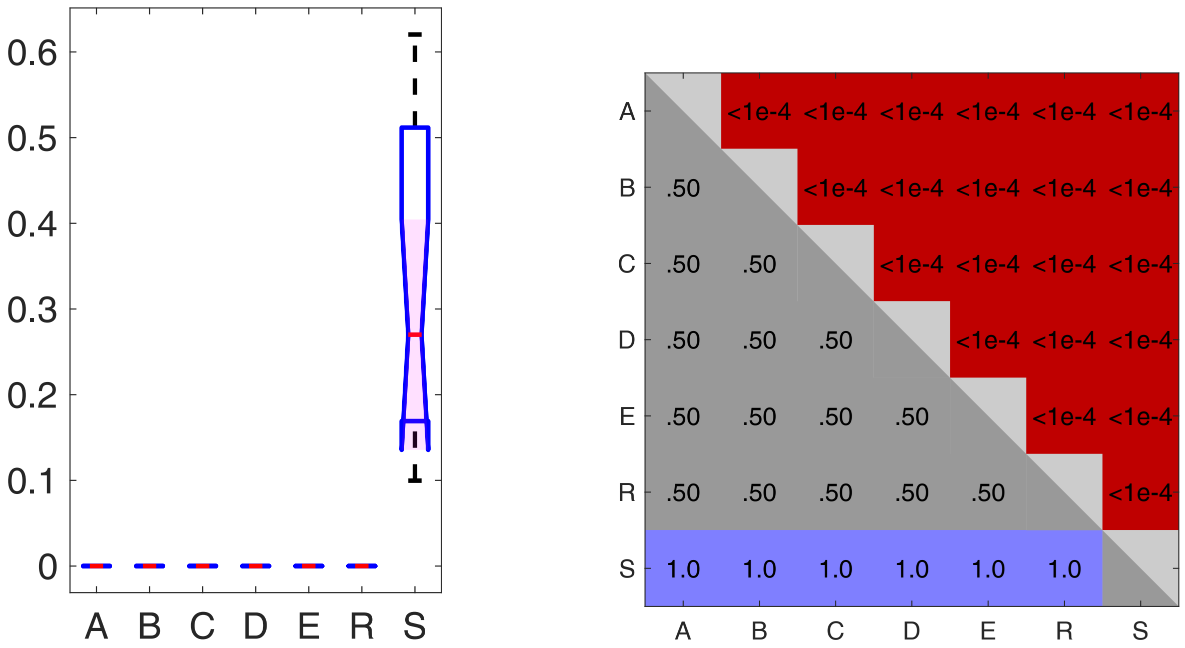


**Heatmap Analysis of Box 8F**

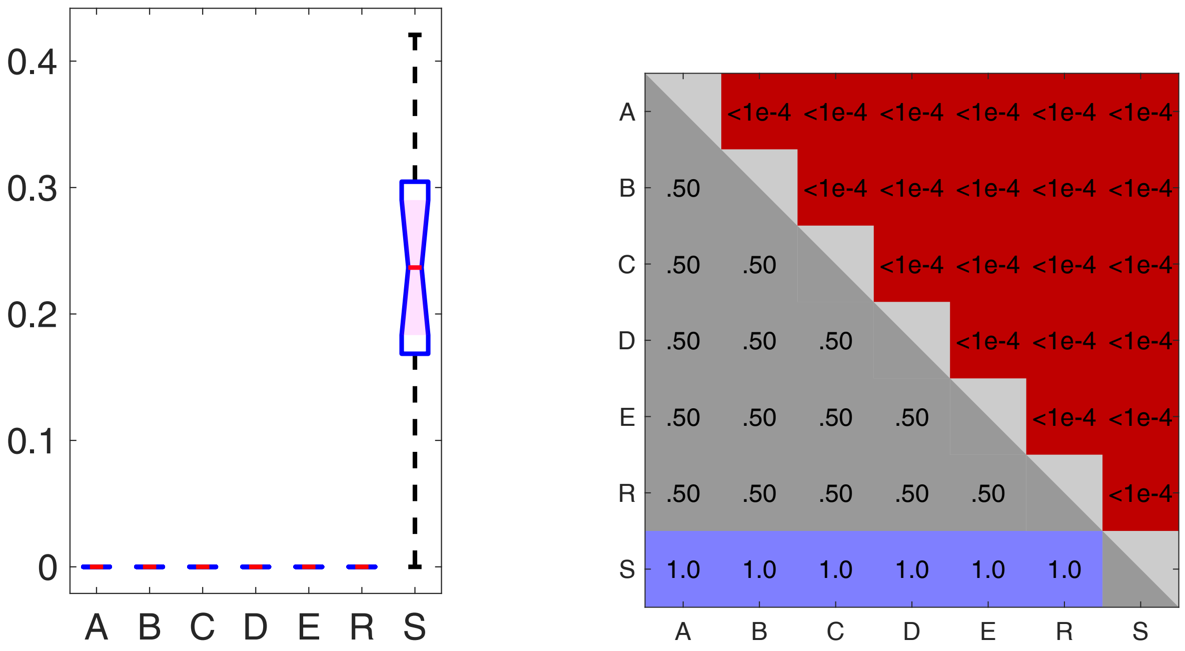

**Heatmap Analysis of Box 8G**

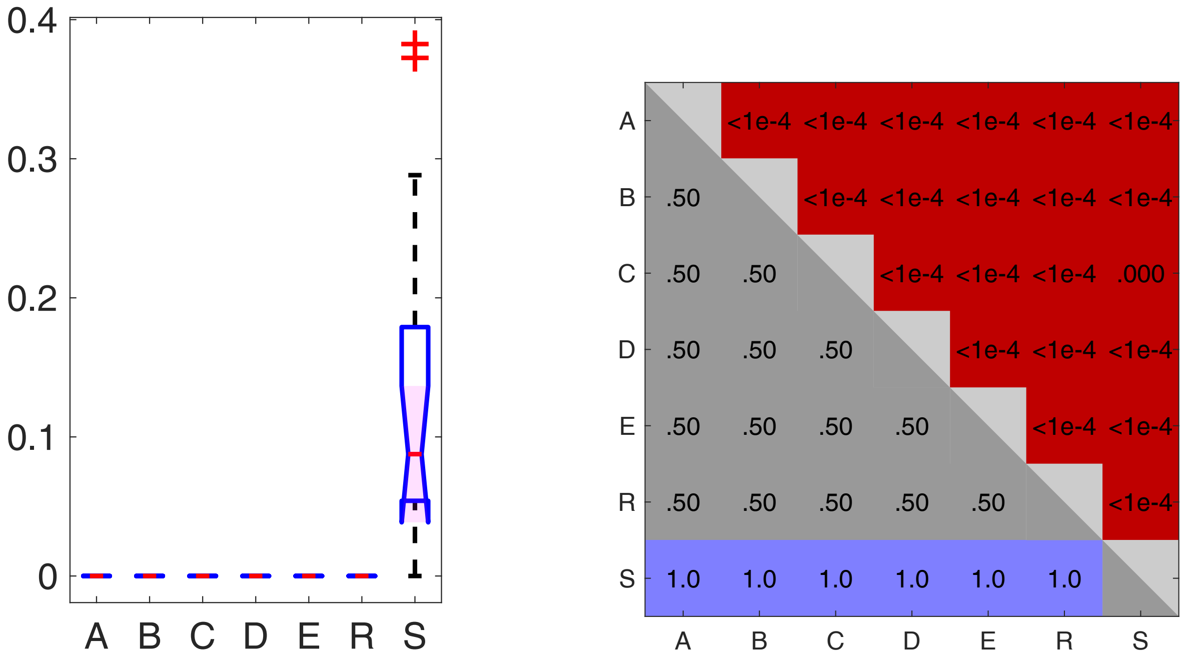


**Heatmap Analysis of Box 8H**

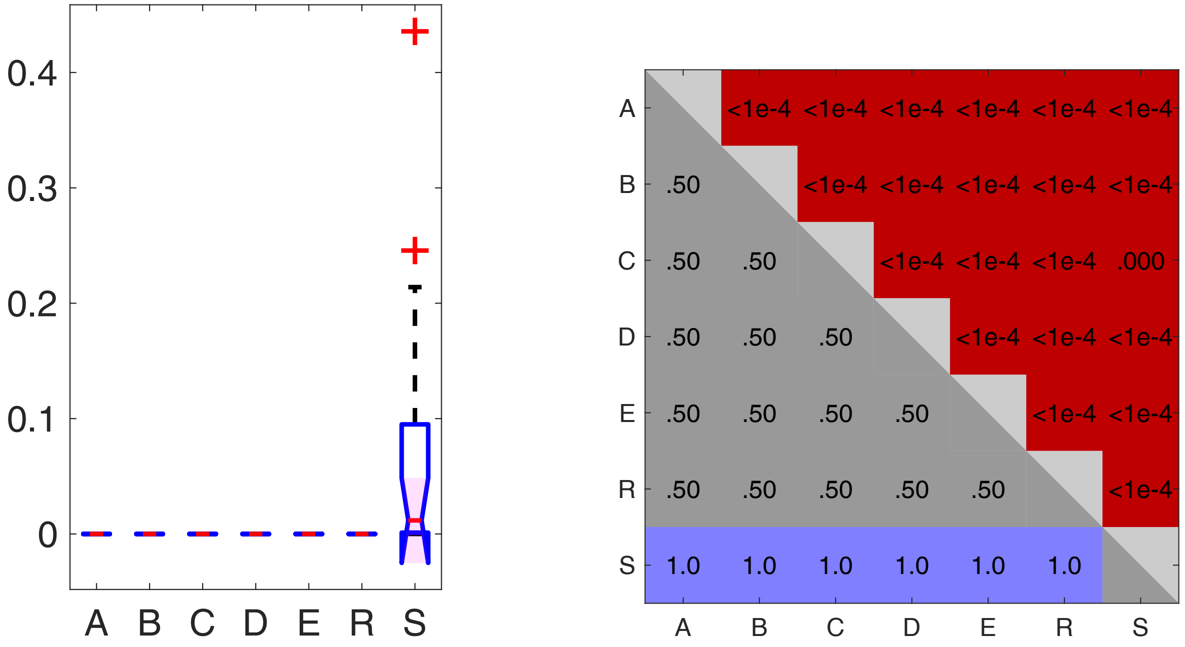

**Heatmap Analysis of Box 90**

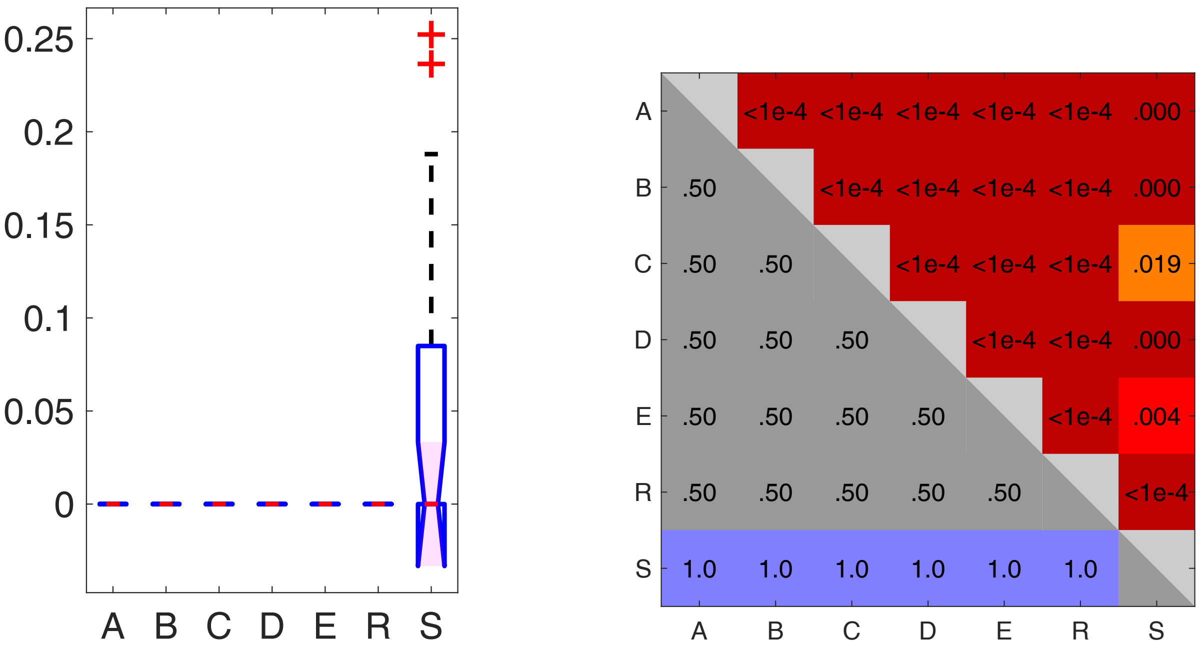


**Heatmap Analysis of Box 91**

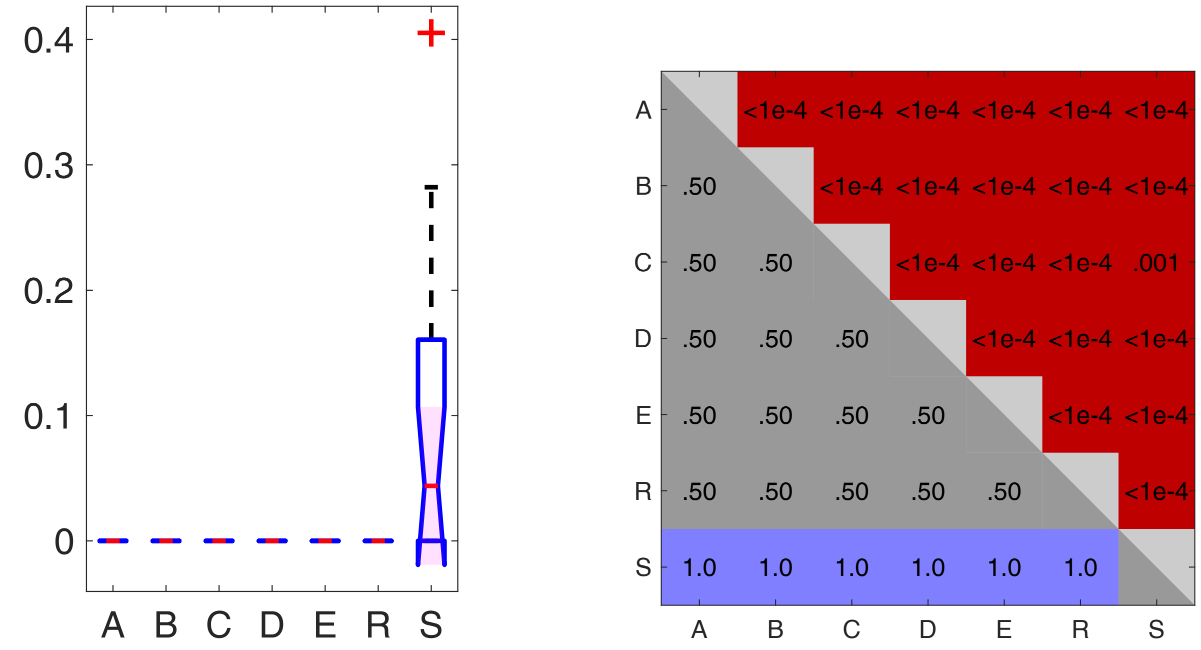

**Heatmap Analysis of Box 92**

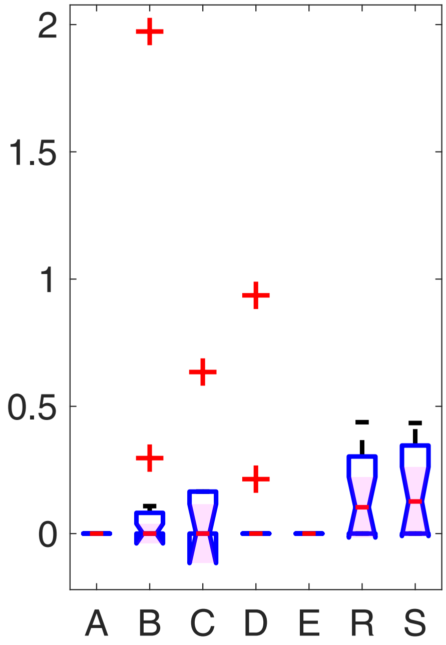


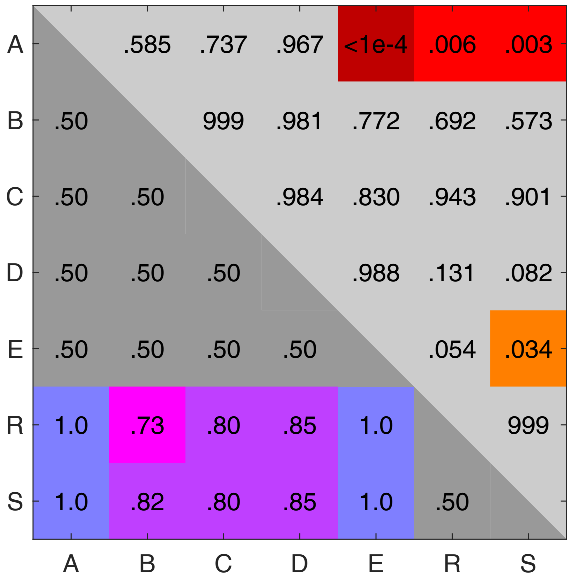


**Heatmap Analysis of Box 93**

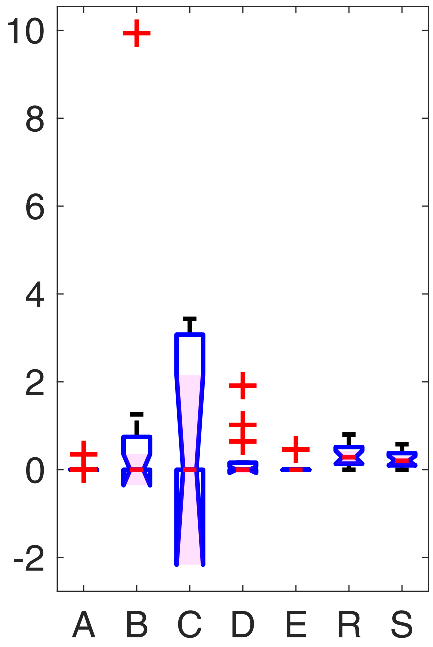


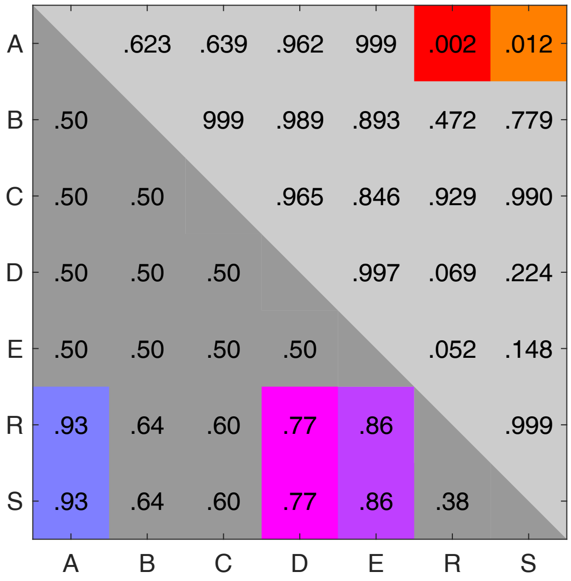

**Heatmap Analysis of Box 94**

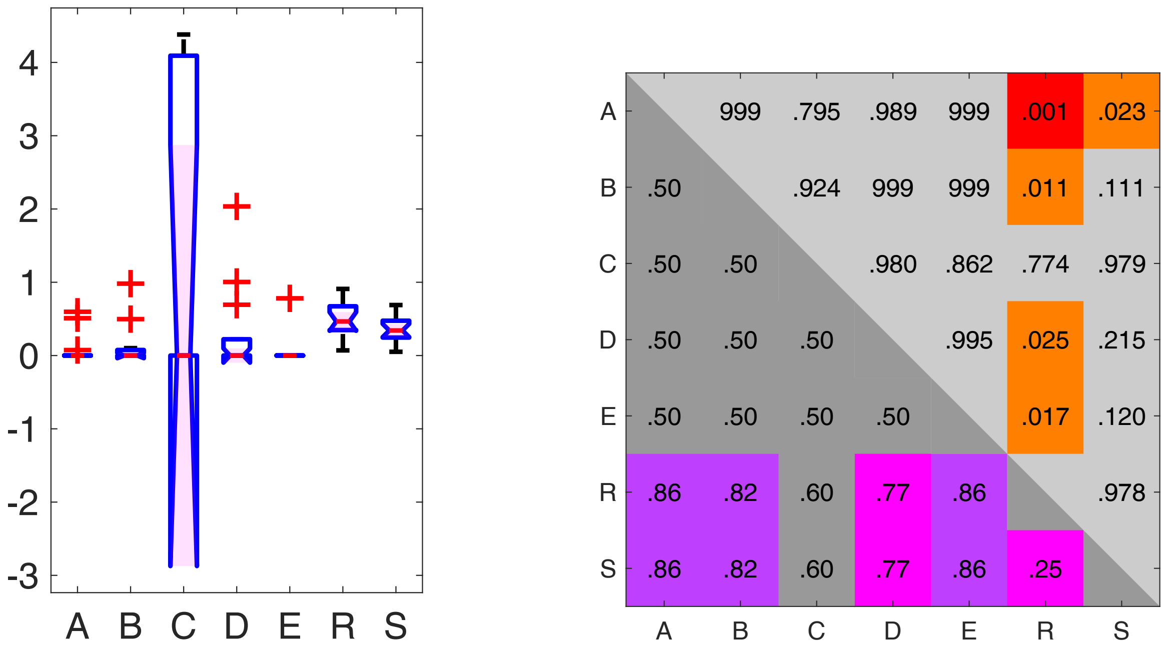


**Heatmap Analysis of Box 95**

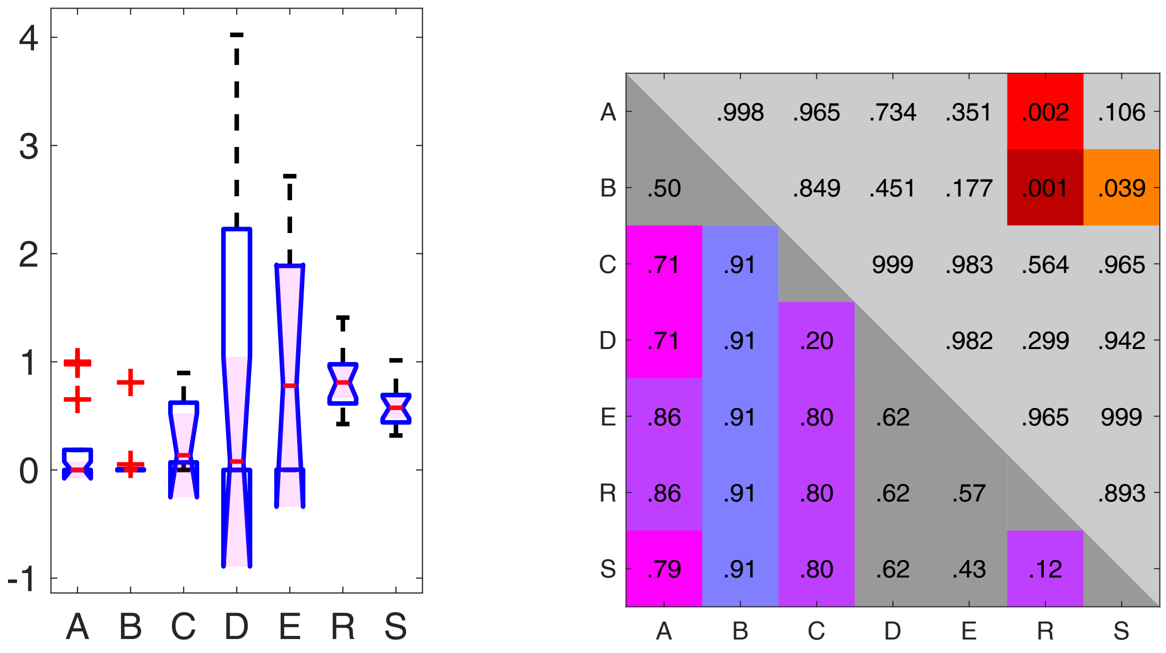

**Heatmap Analysis of Box 96**

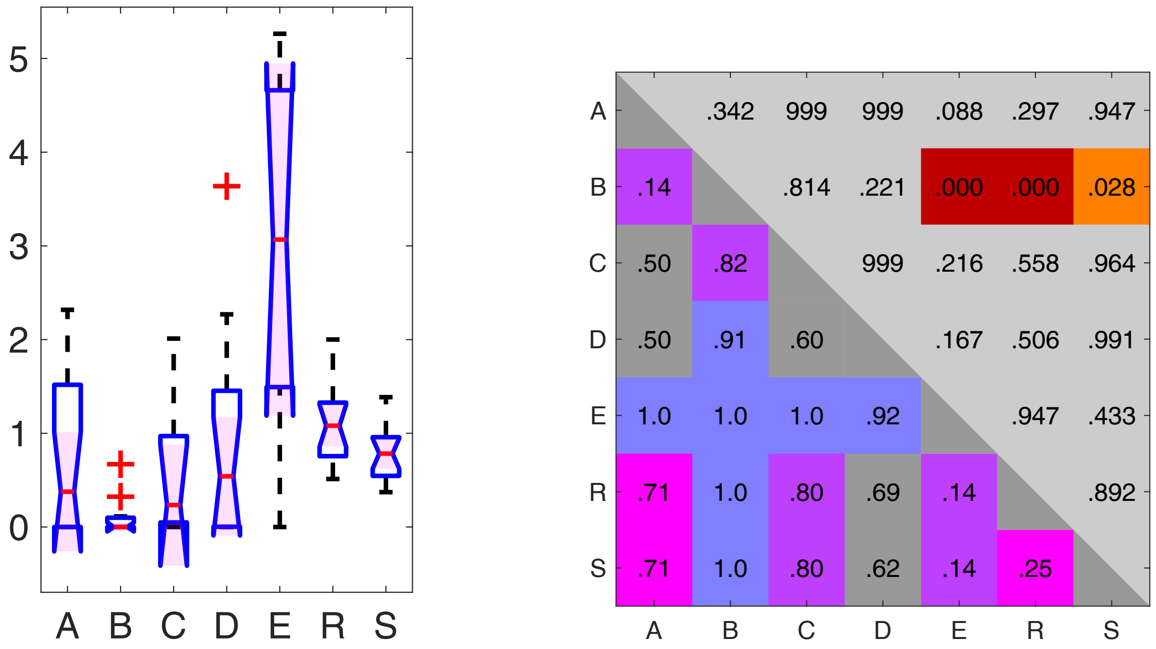


**Heatmap Analysis of Box 97**

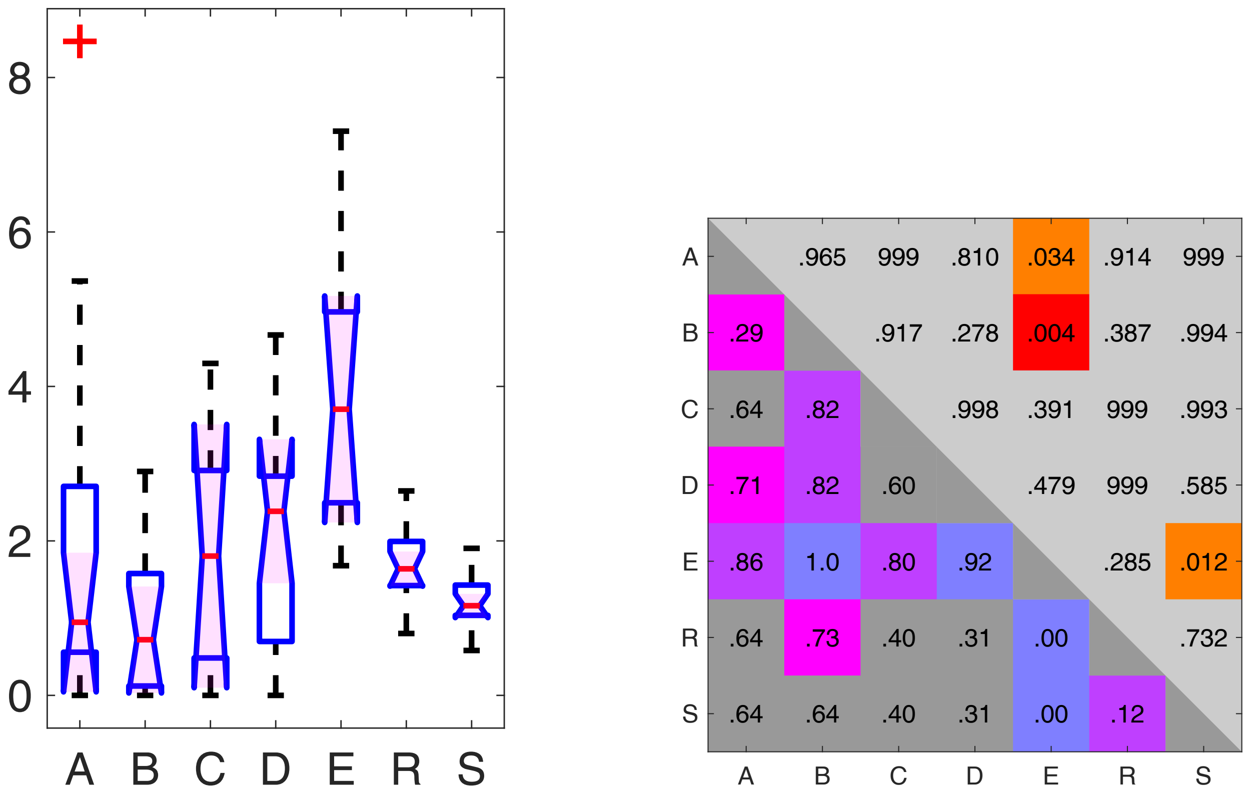

**Heatmap Analysis of Box 98**

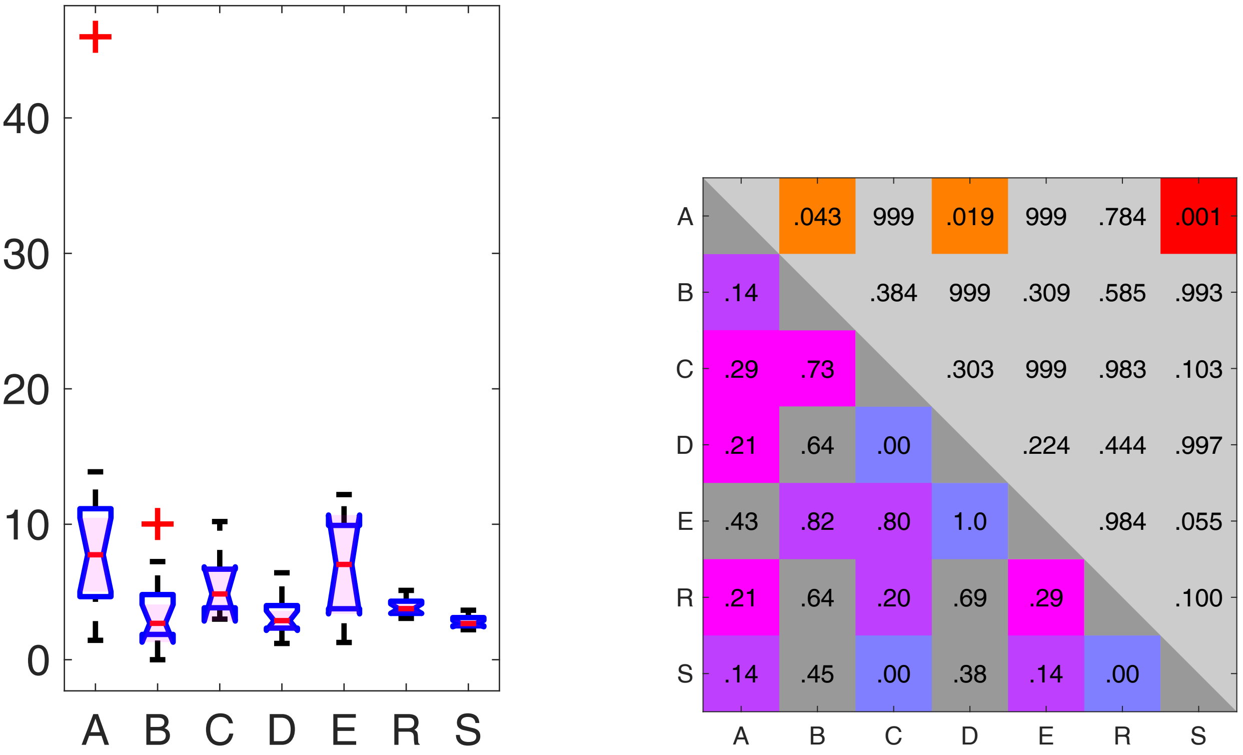


**Heatmap Analysis of Box 99**

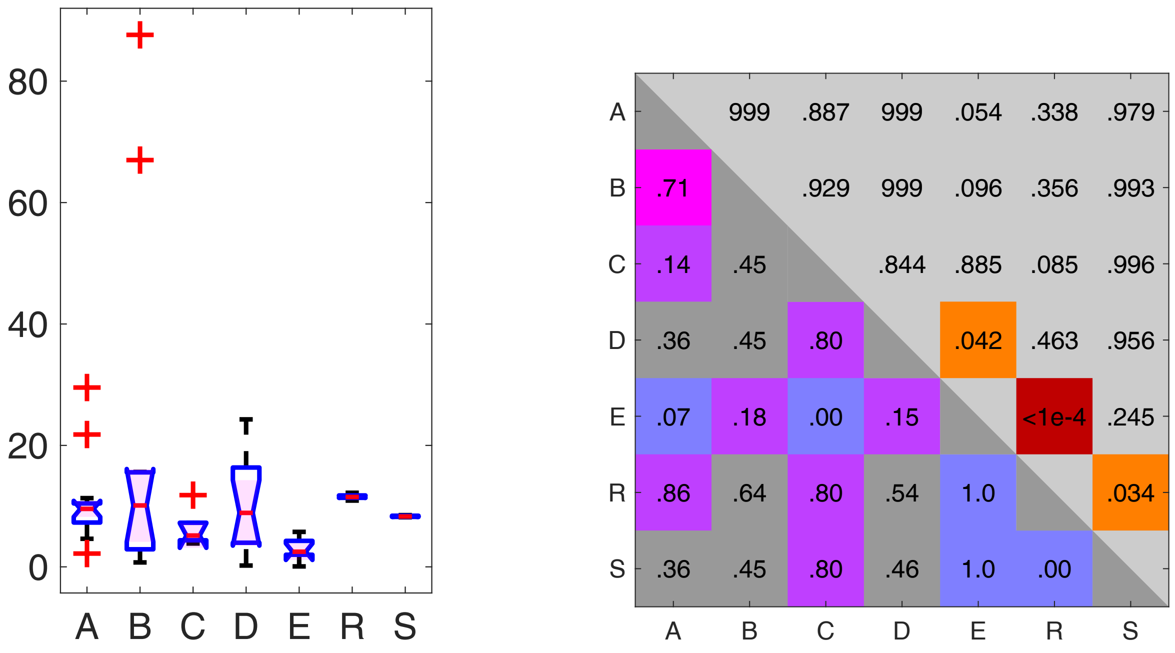

**Heatmap Analysis of Box 9A**

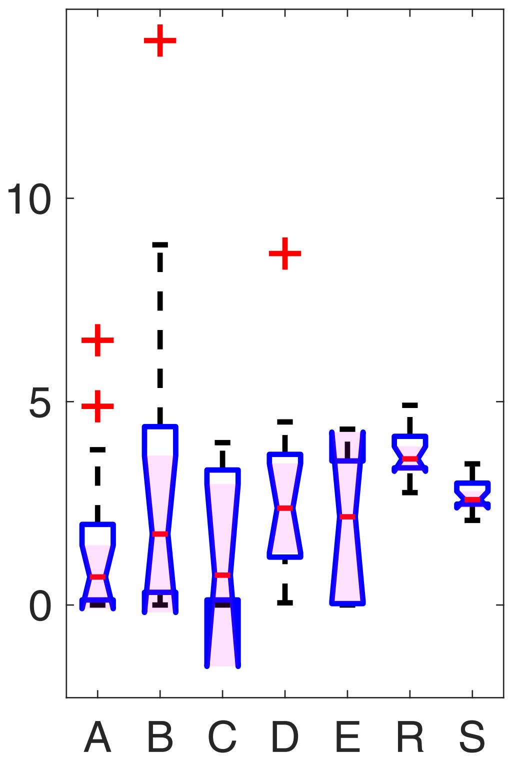


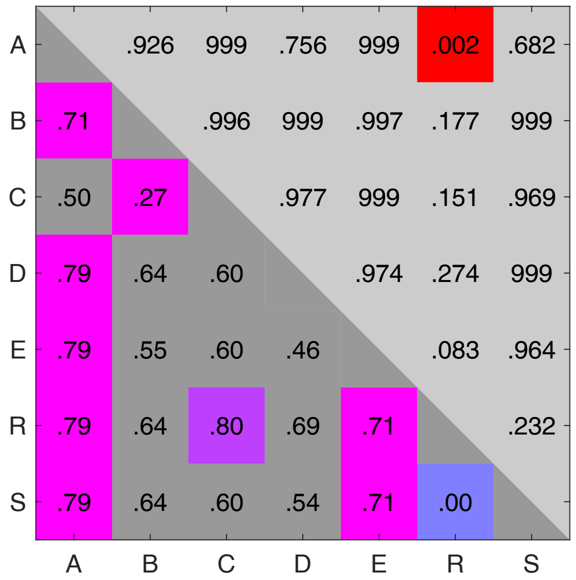


**Heatmap Analysis of Box 9B**

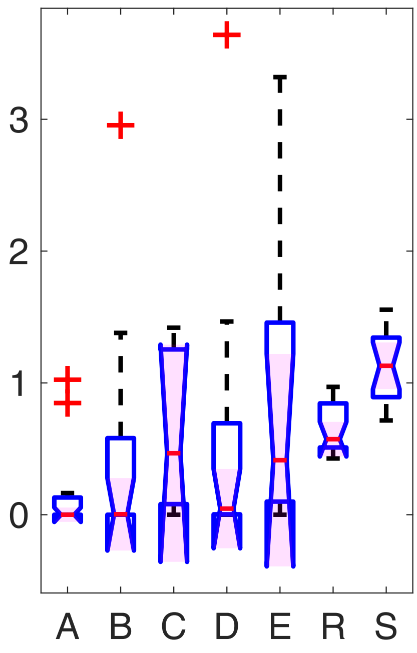


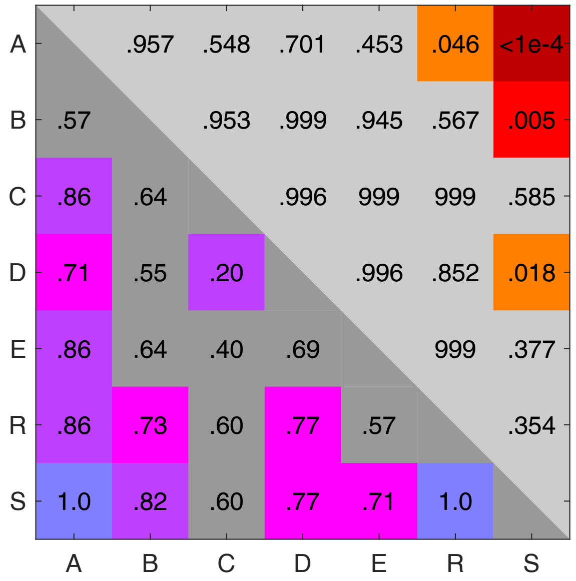

**Heatmap Analysis of Box 9C**

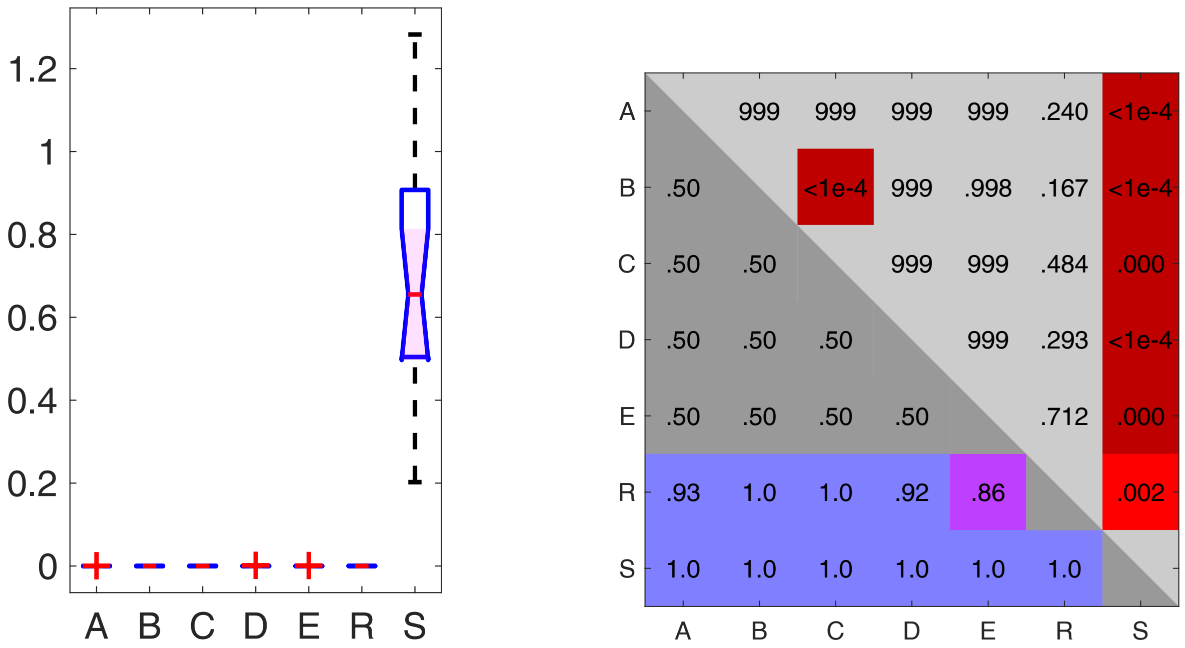


**Heatmap Analysis of Box 9D**

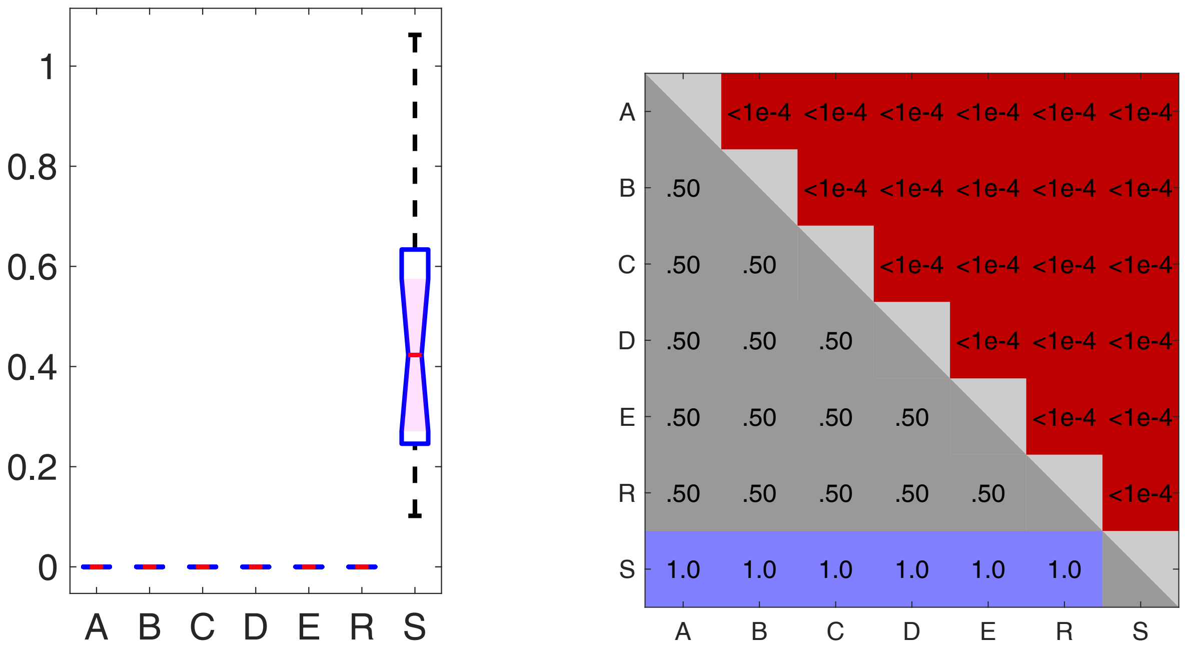

**Heatmap Analysis of Box 9E**

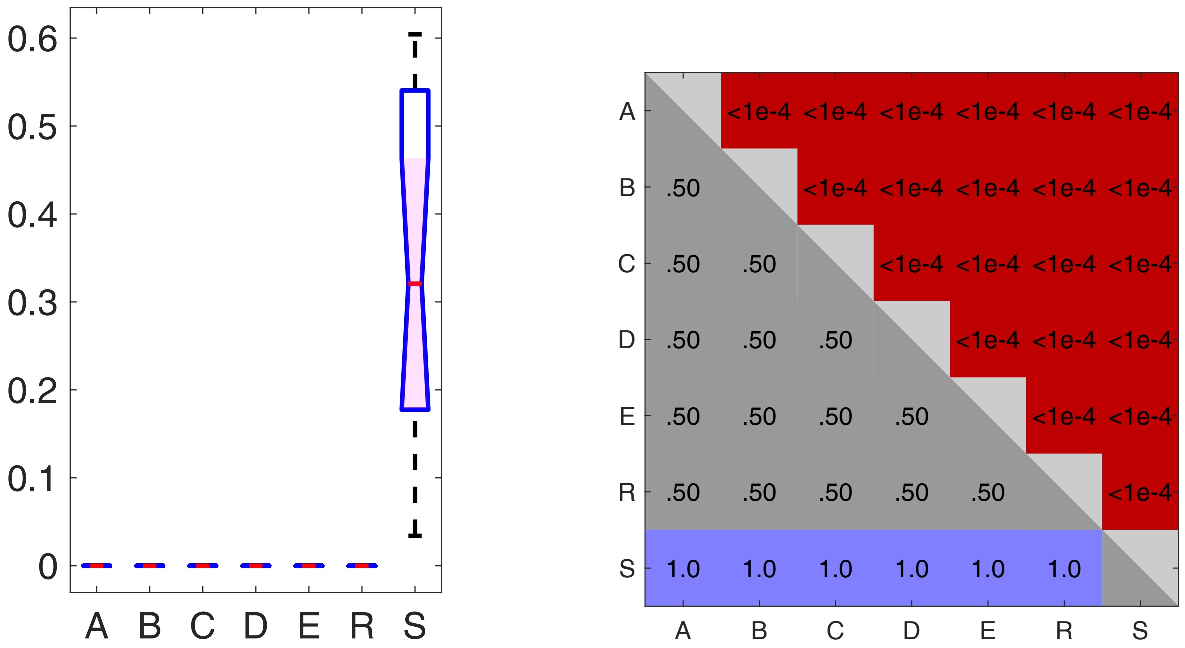


**Heatmap Analysis of Box 9F**

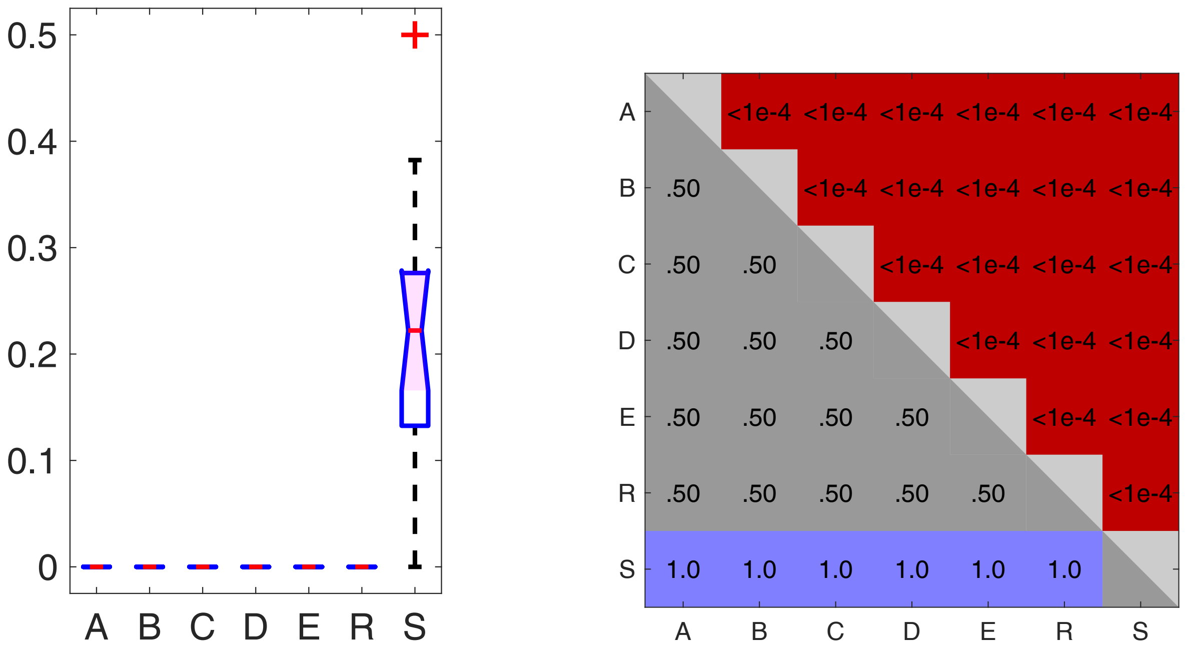

**Heatmap Analysis of Box 9G**

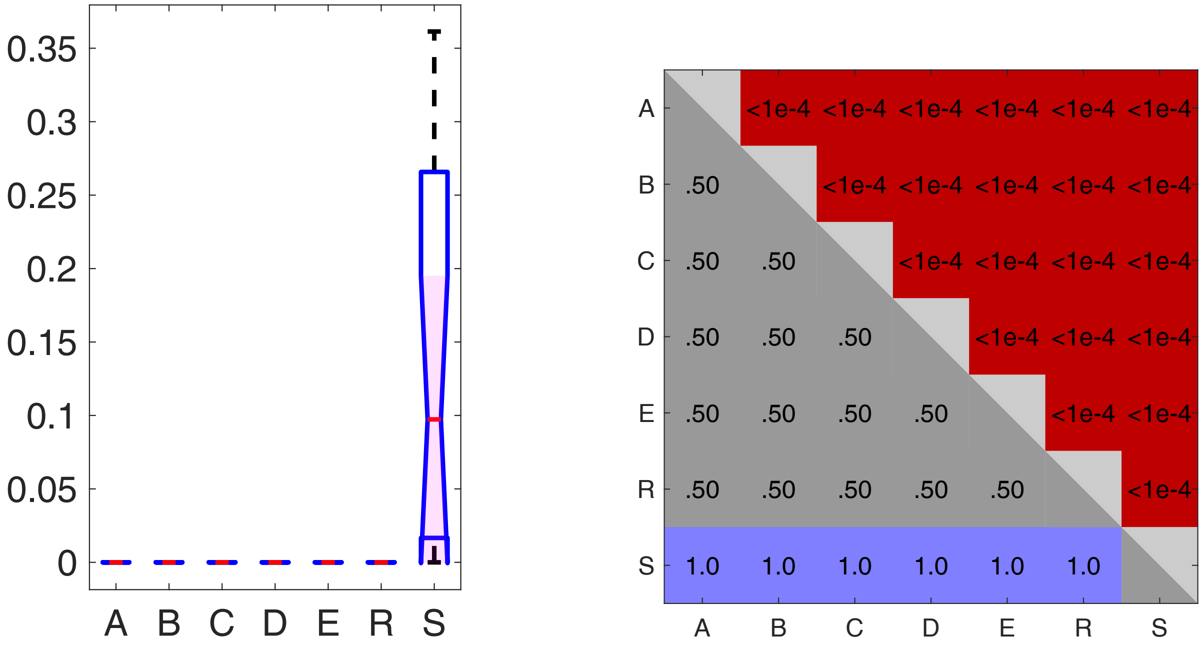


**Heatmap Analysis of Box 9H**

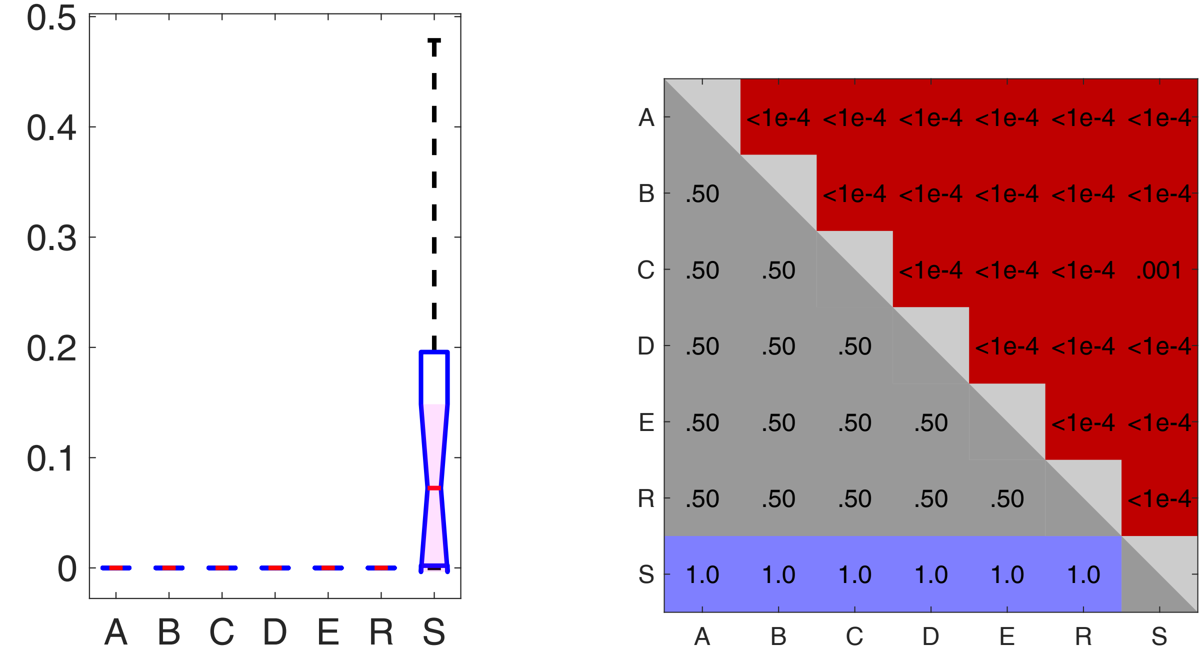

**Heatmap Analysis of Box A0**

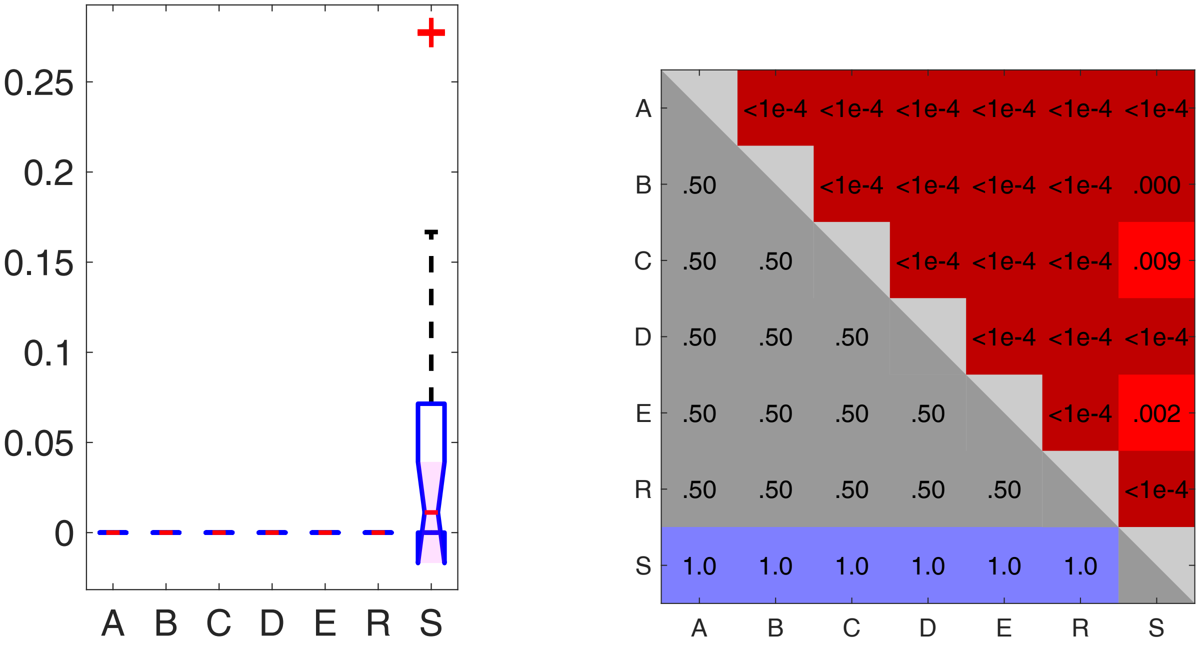


**Heatmap Analysis of Box A1**

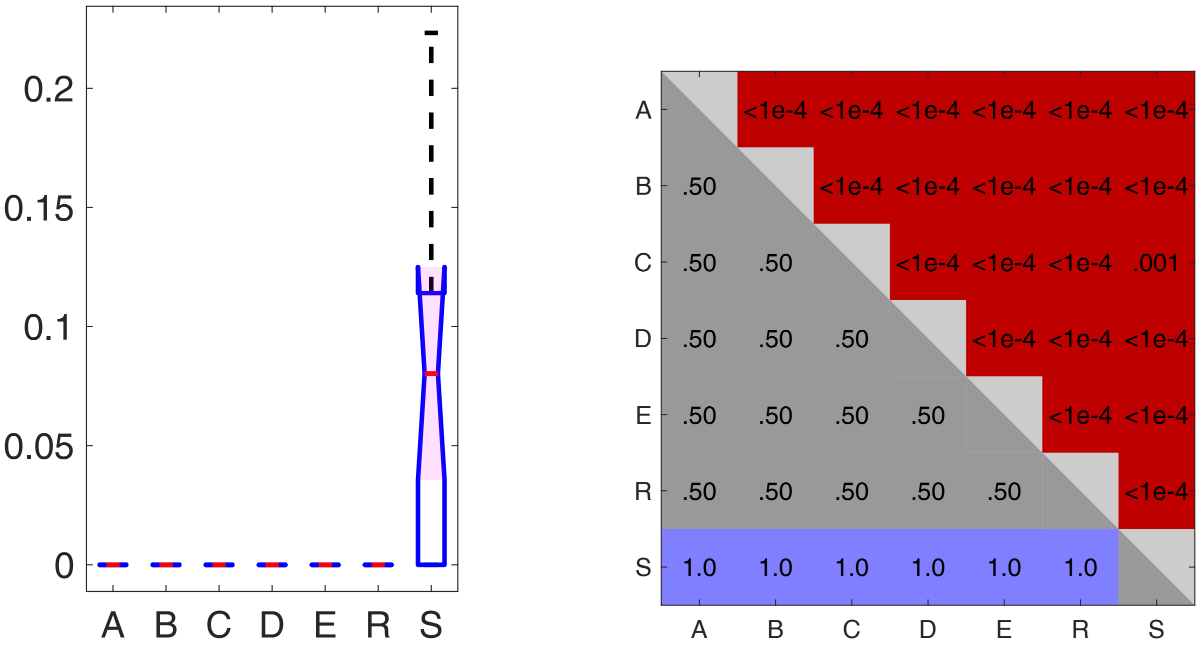

**Heatmap Analysis of Box A2**

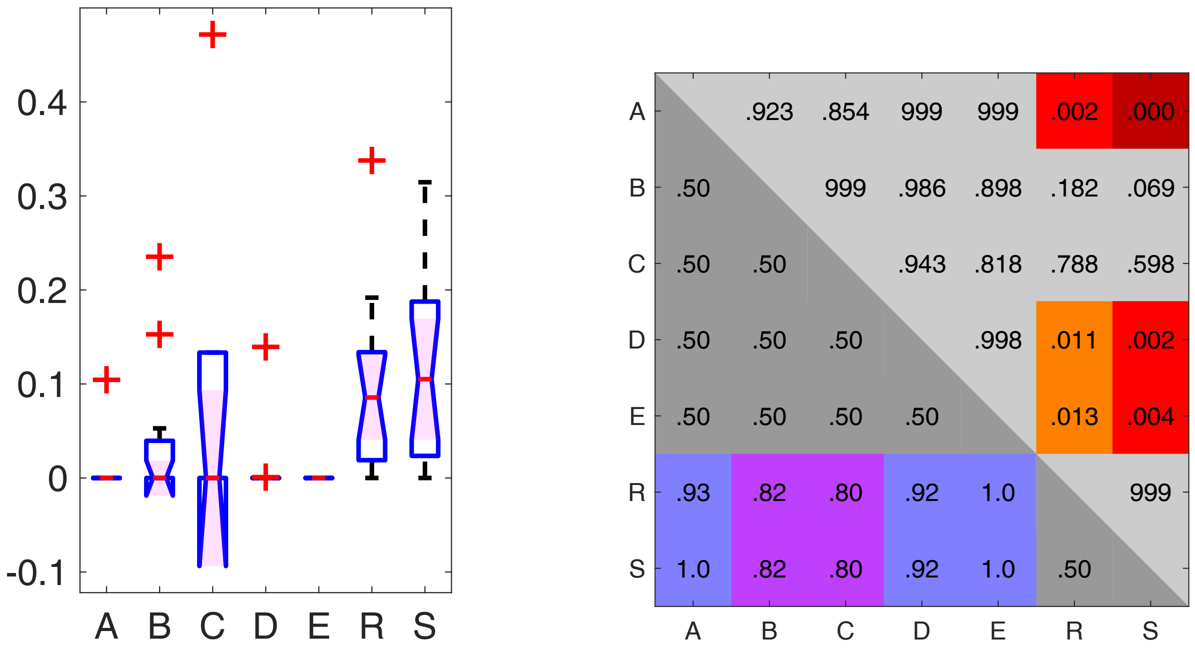


**Heatmap Analysis of Box A3**

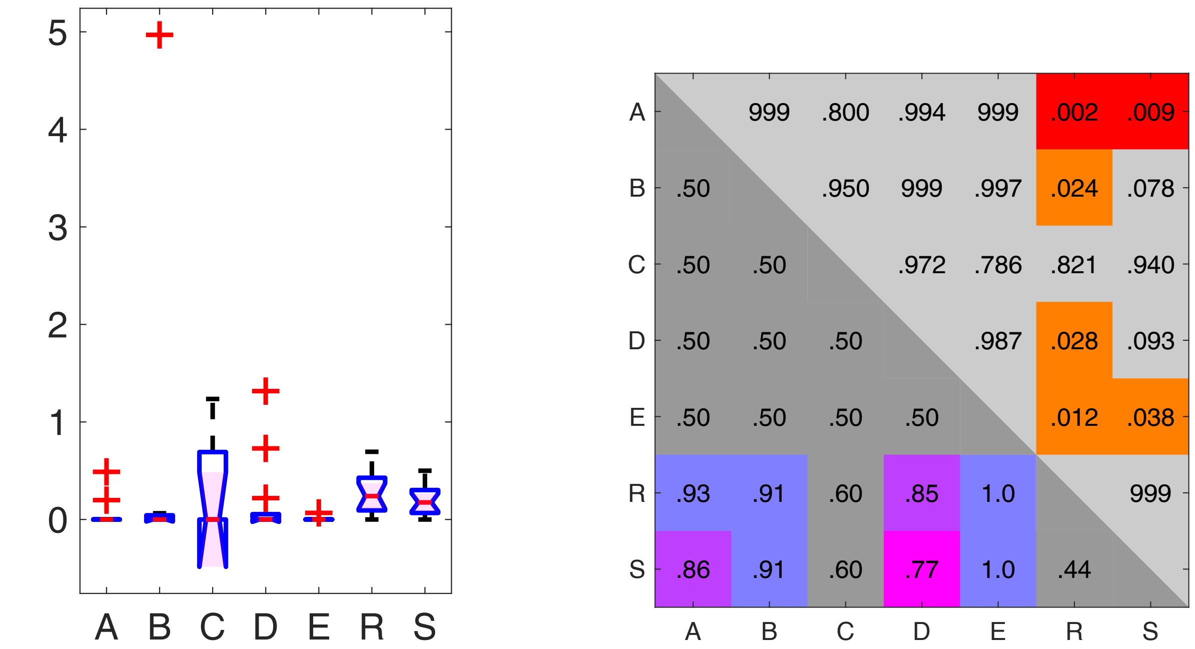

**Heatmap Analysis of Box A4**

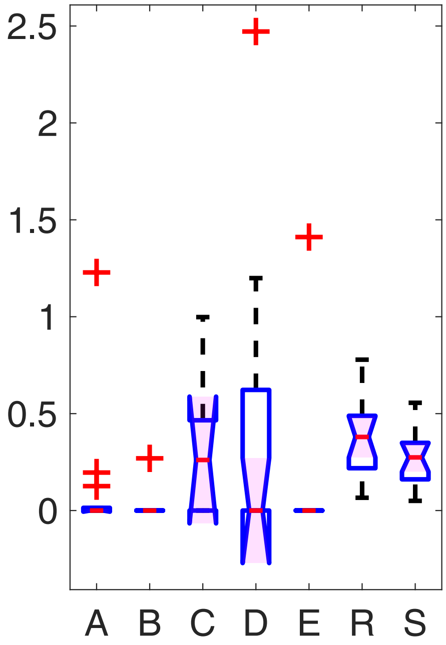


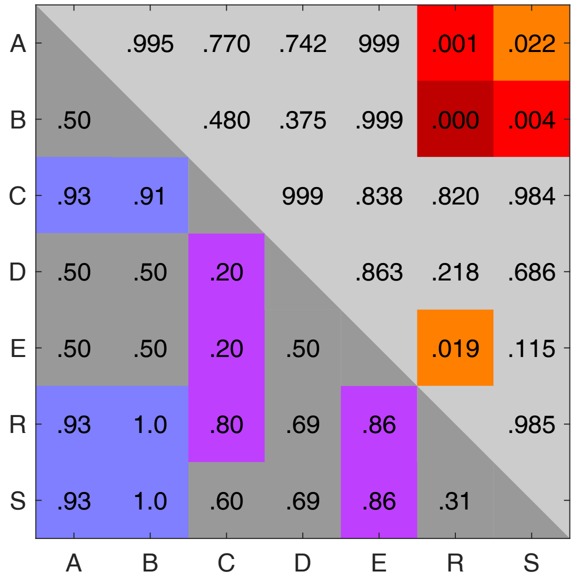


**Heatmap Analysis of Box A5**

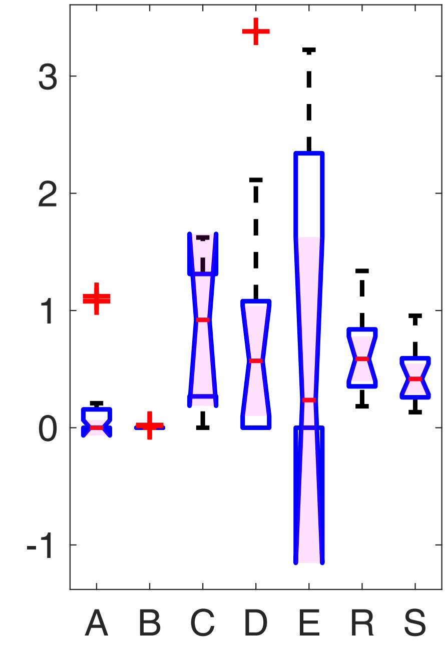


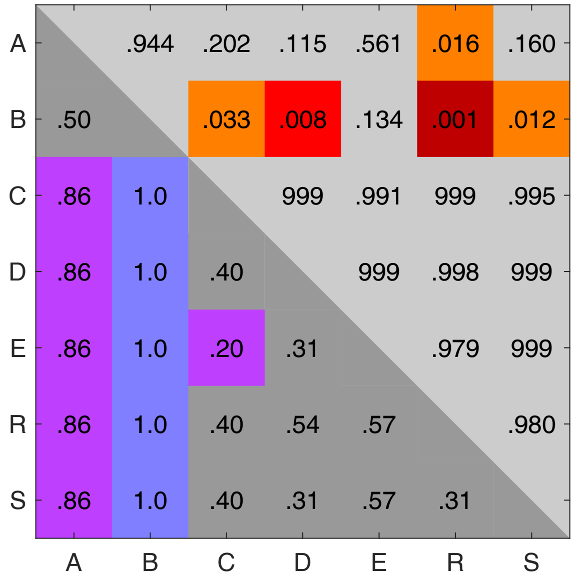

**Heatmap Analysis of Box A6**

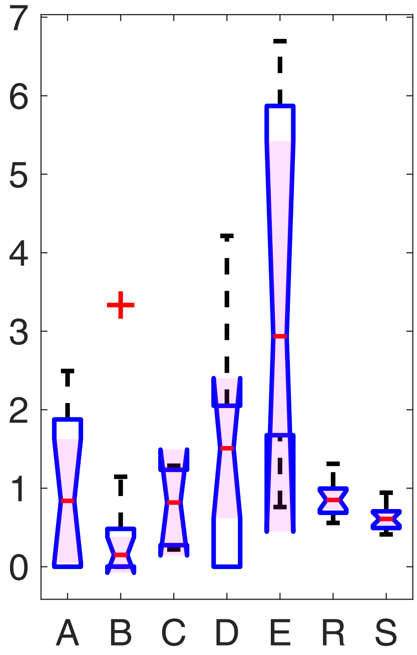


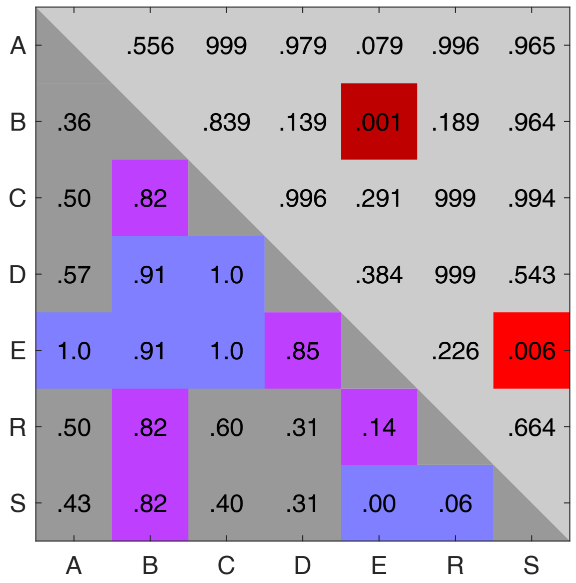


**Heatmap Analysis of Box A7**

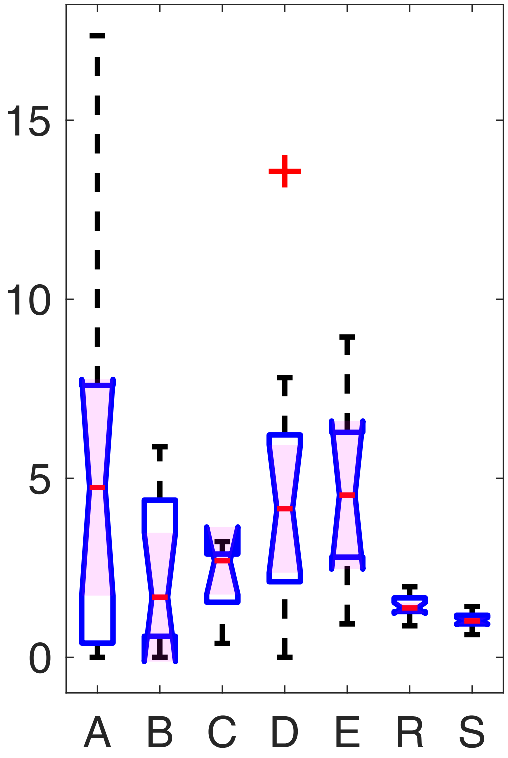


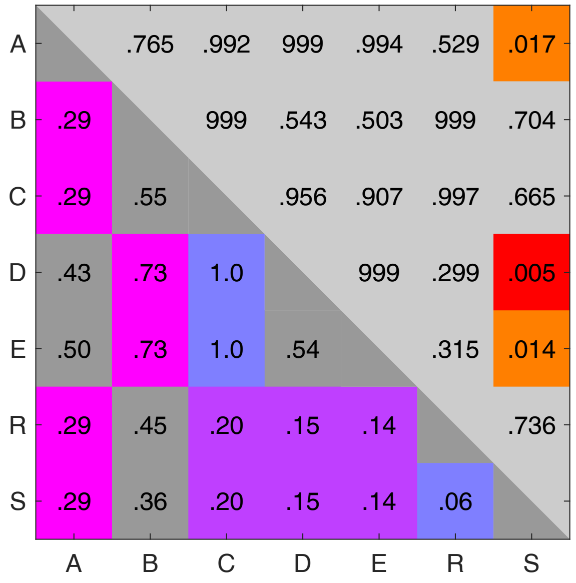

**Heatmap Analysis of Box A8**

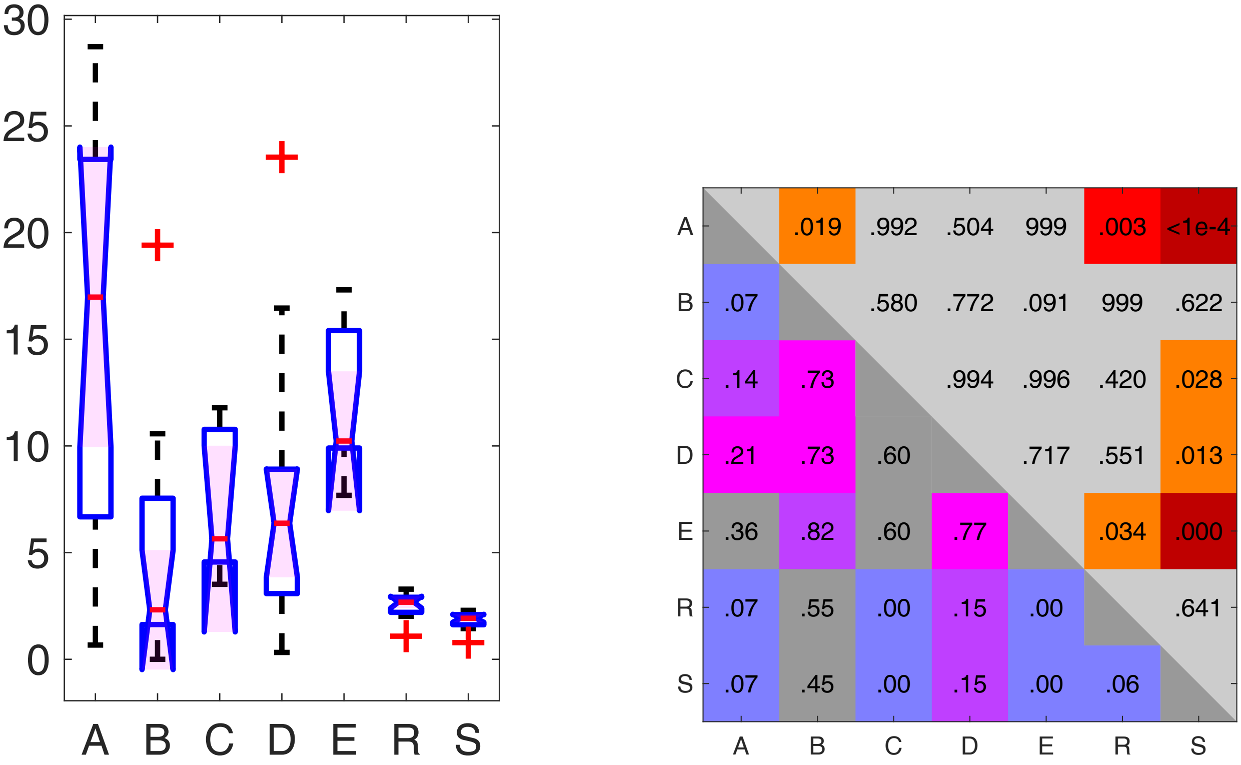


**Heatmap Analysis of Box A9**

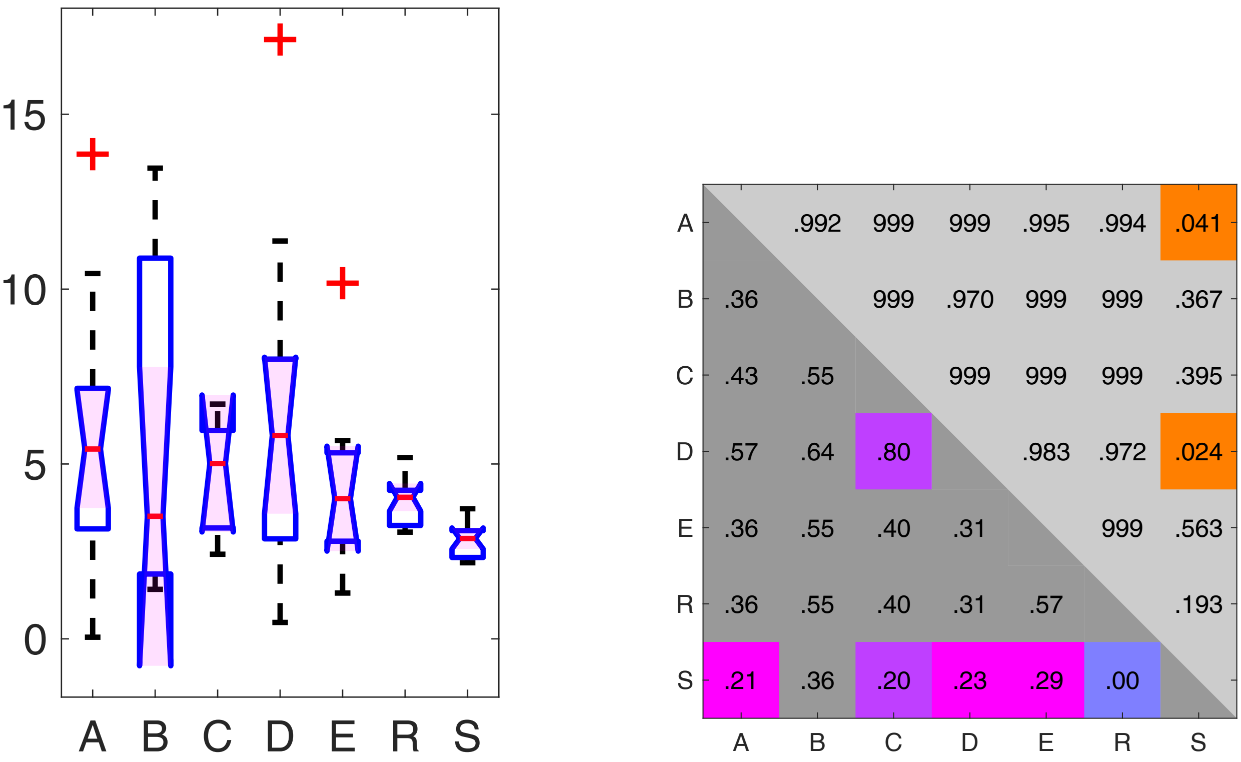

**Heatmap Analysis of Box AA**

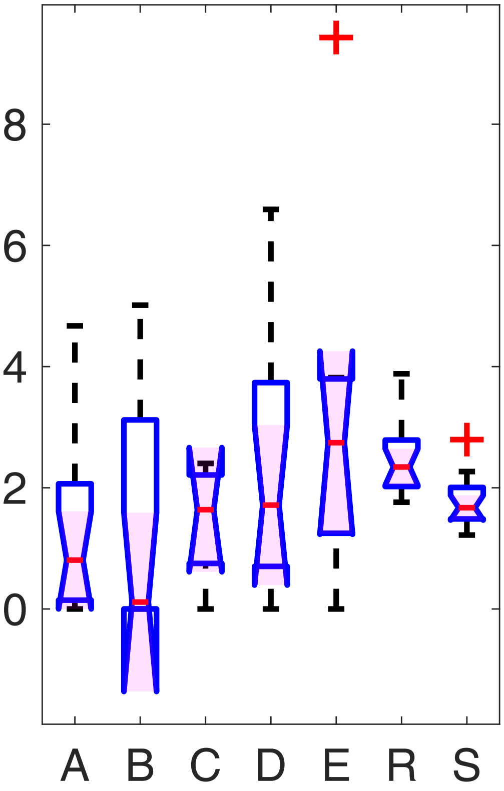


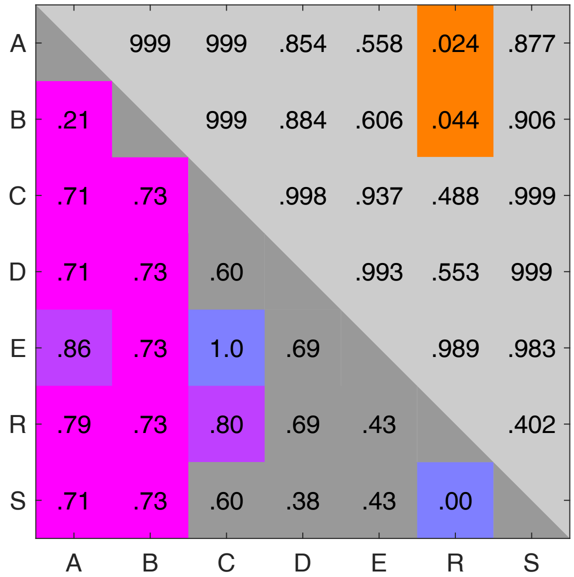


**Heatmap Analysis of Box AB**

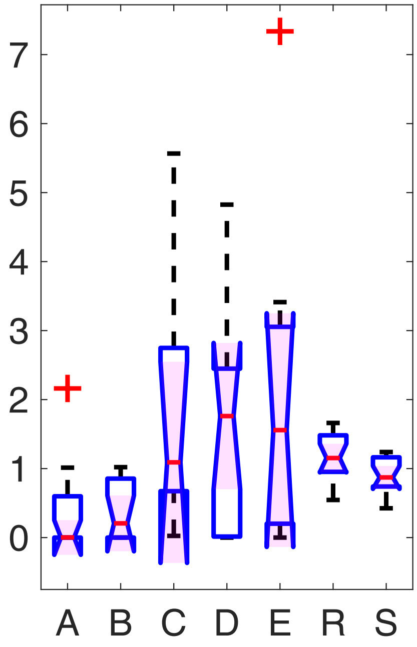


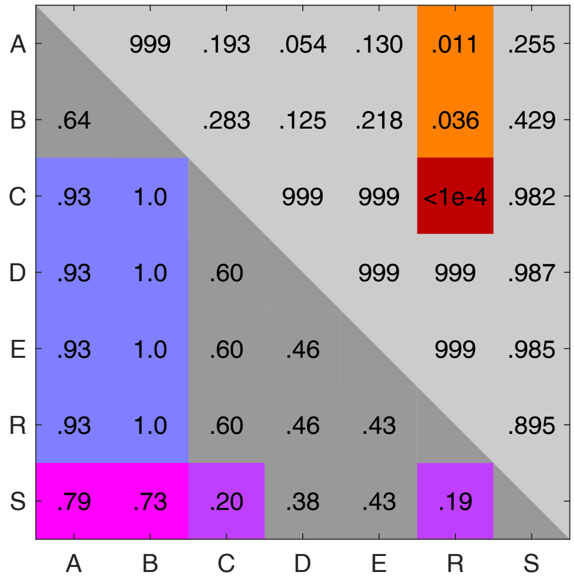

**Heatmap Analysis of Box AC**

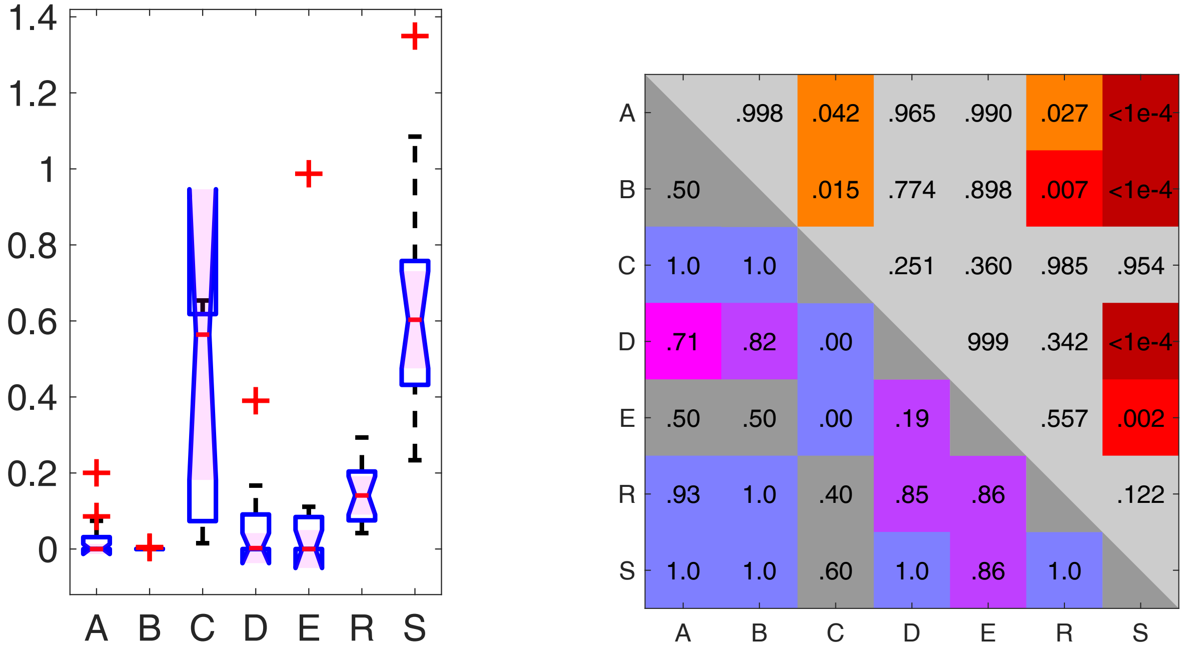


**Heatmap Analysis of Box AD**

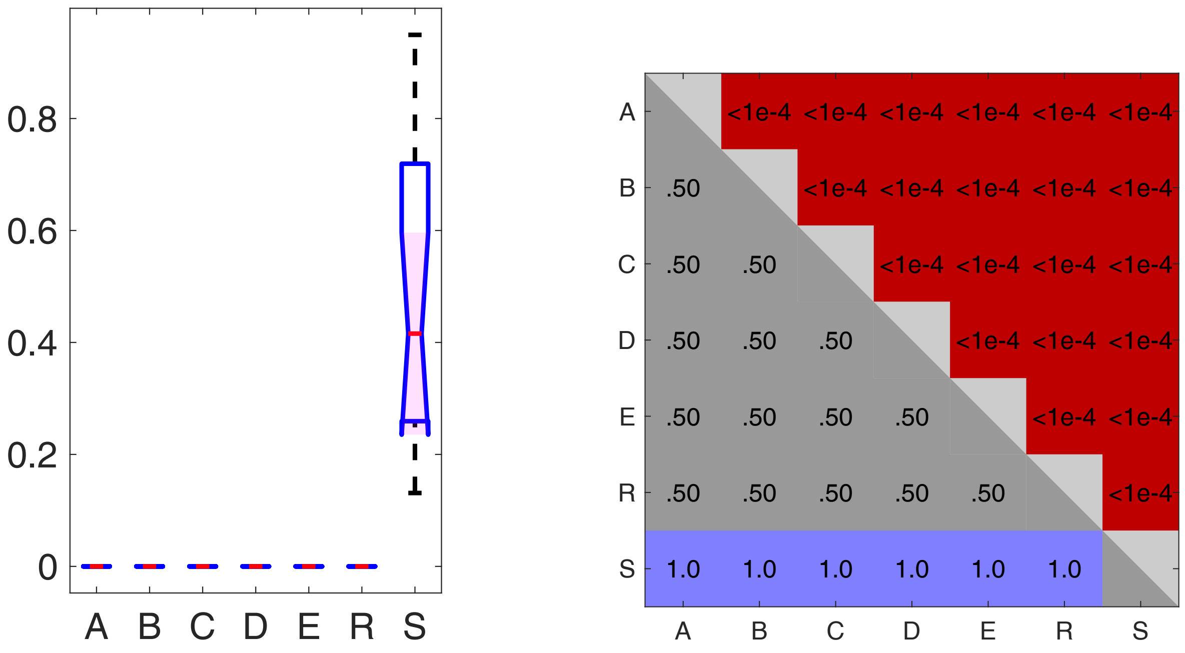

**Heatmap Analysis of Box AE**

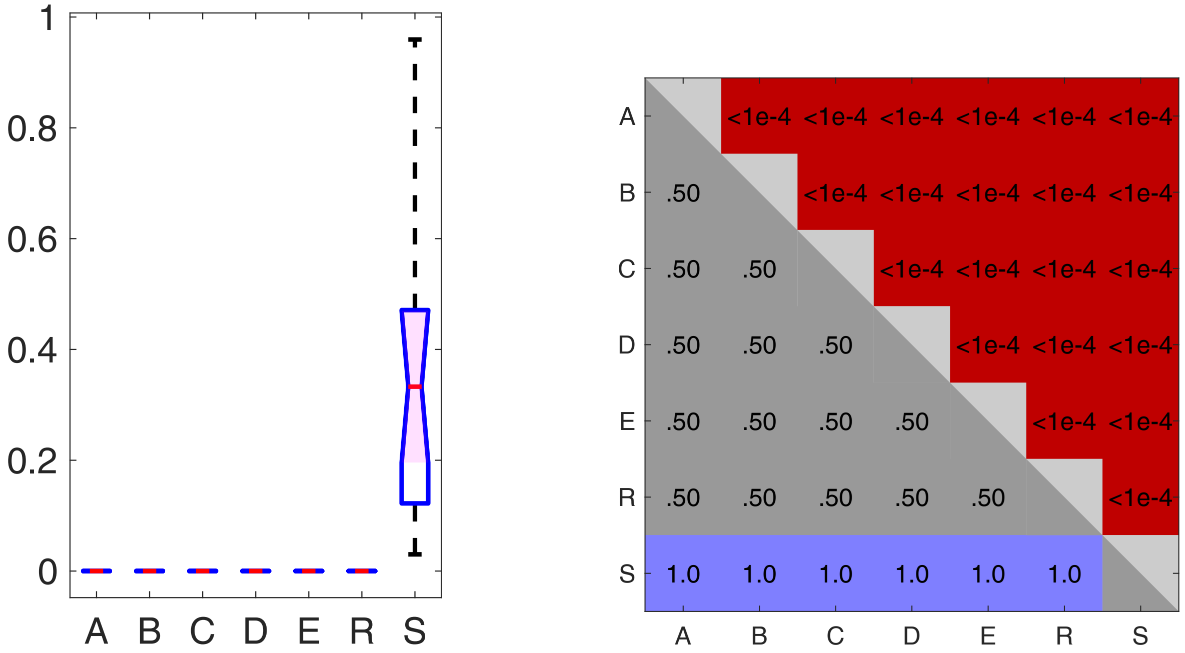


**Heatmap Analysis of Box AF**

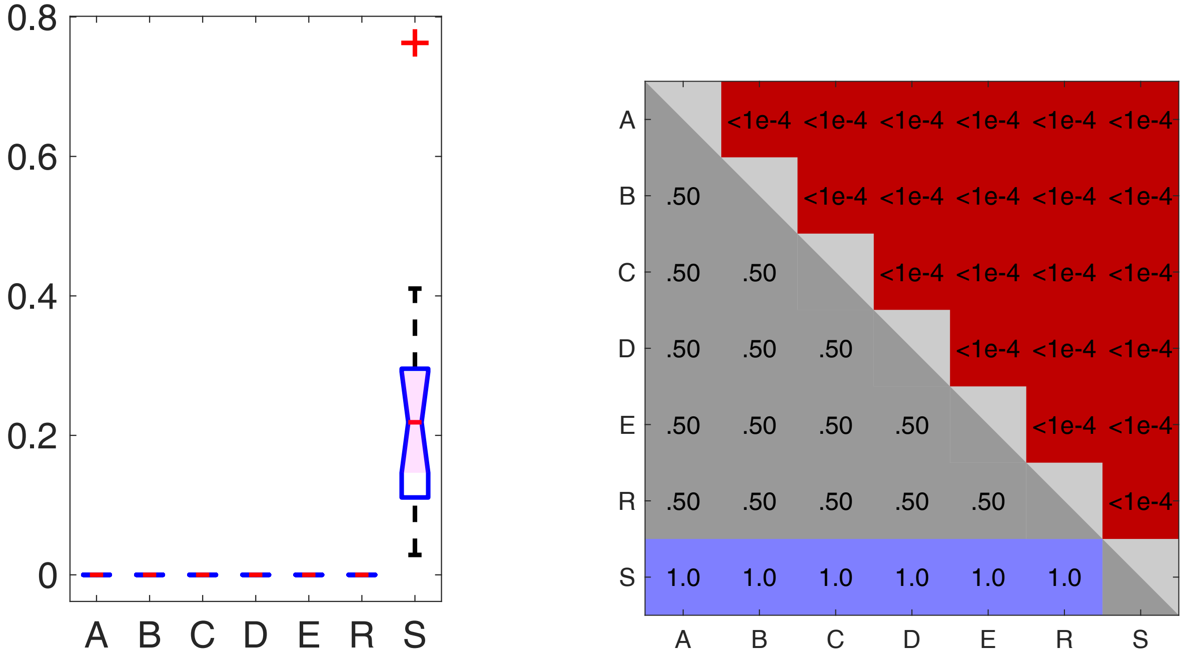

**Heatmap Analysis of Box AG**

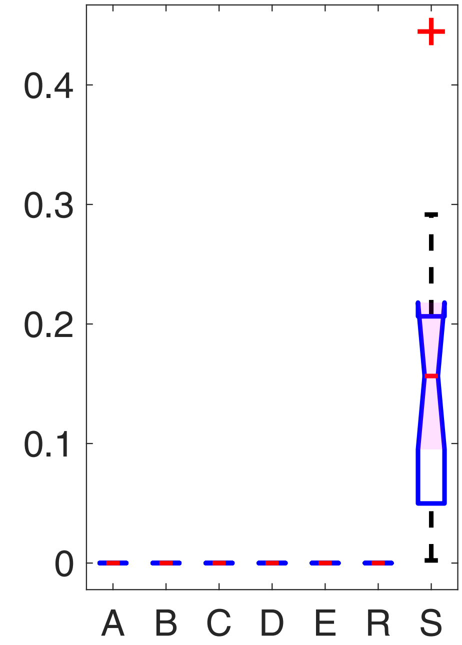


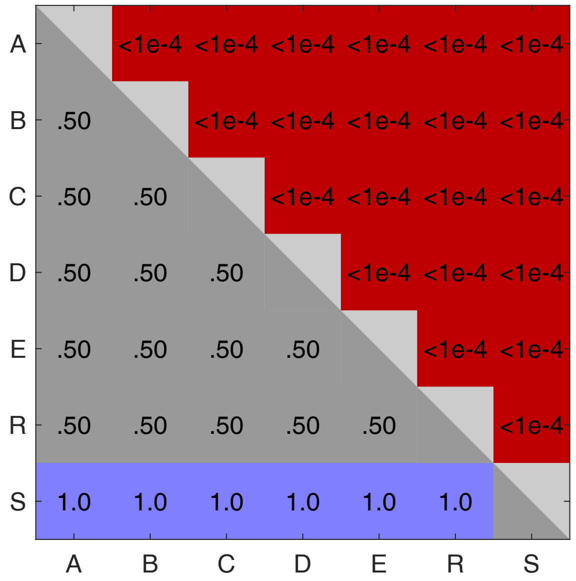


**Heatmap Analysis of Box AH**

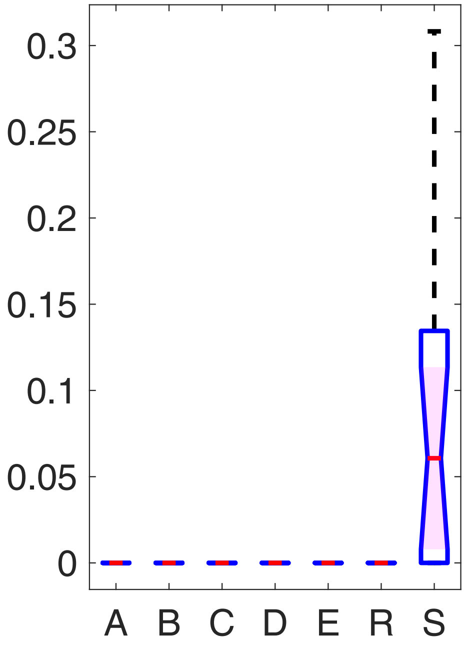


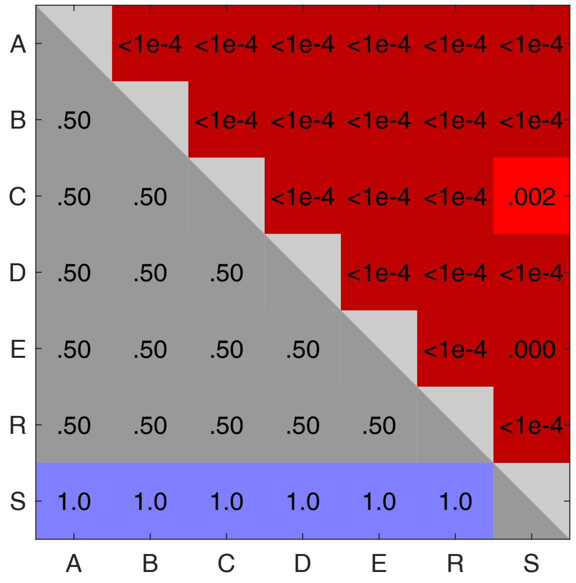

**Heatmap Analysis of Box B0**

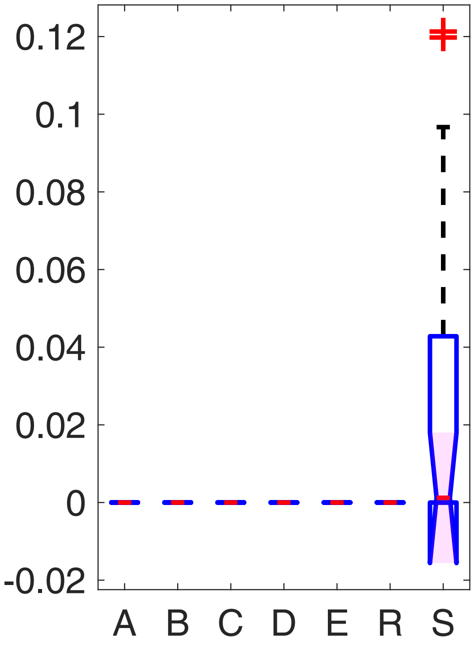


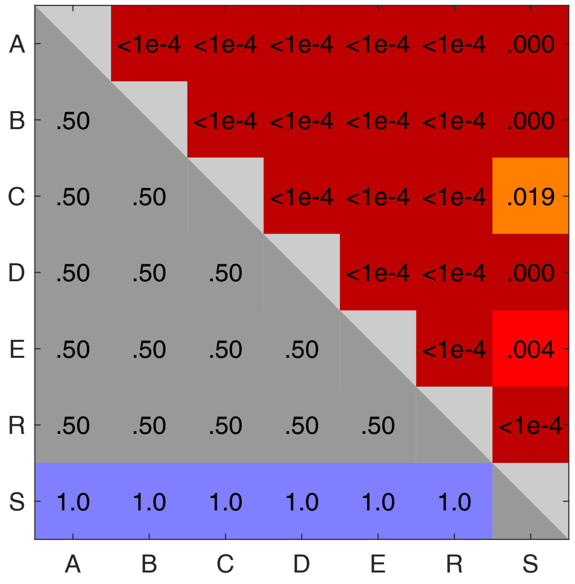


**Heatmap Analysis of Box B1**

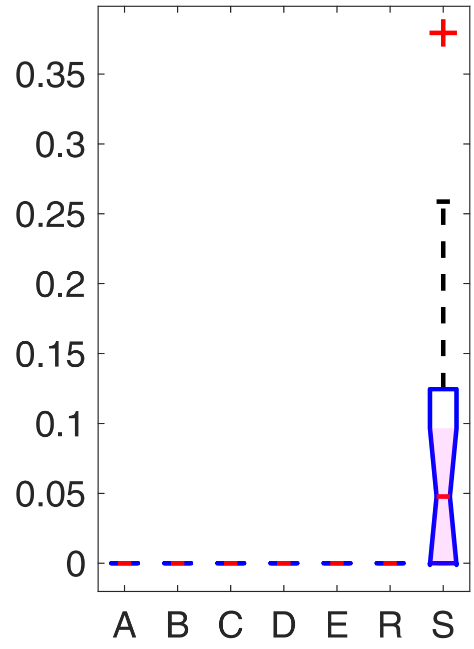


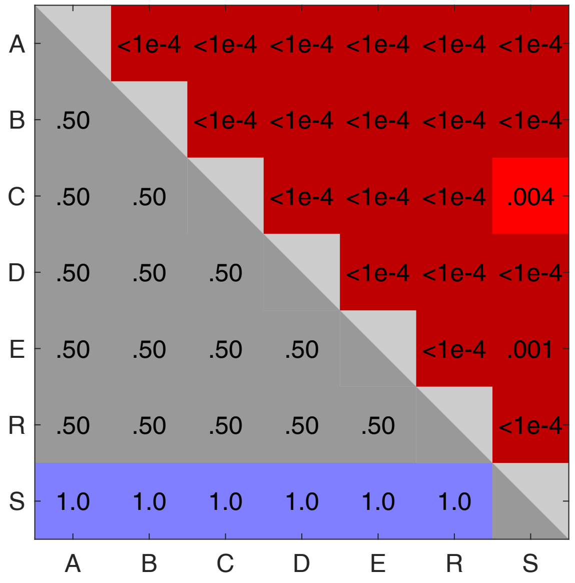

**Heatmap Analysis of Box B2**

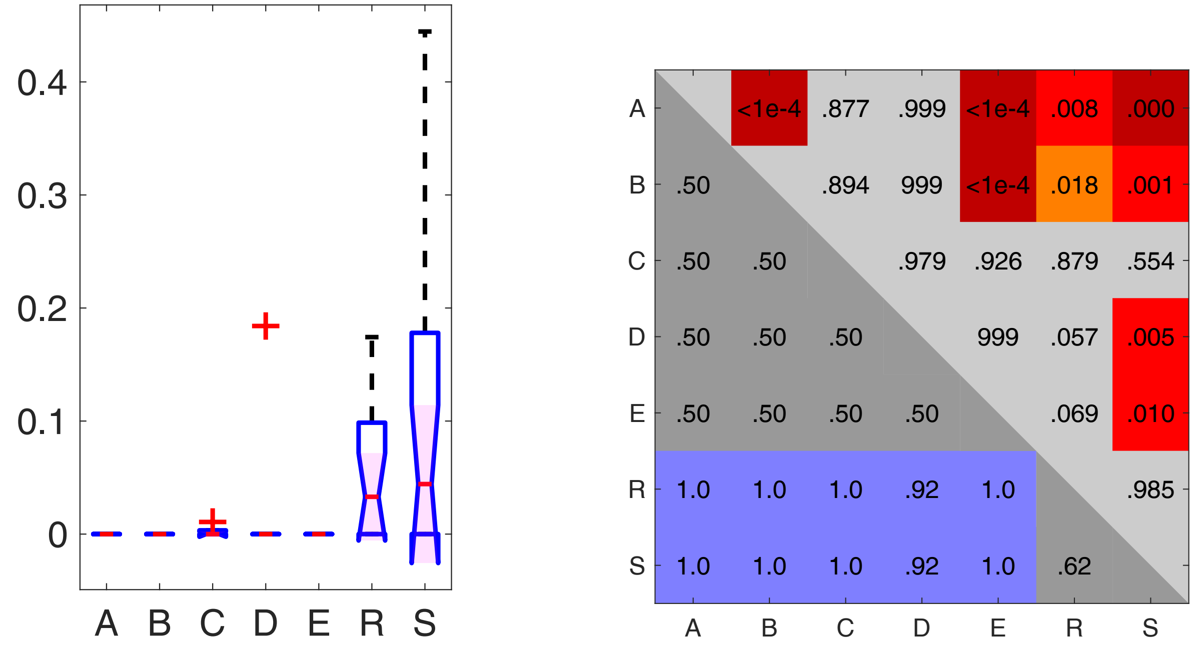


**Heatmap Analysis of Box B3**

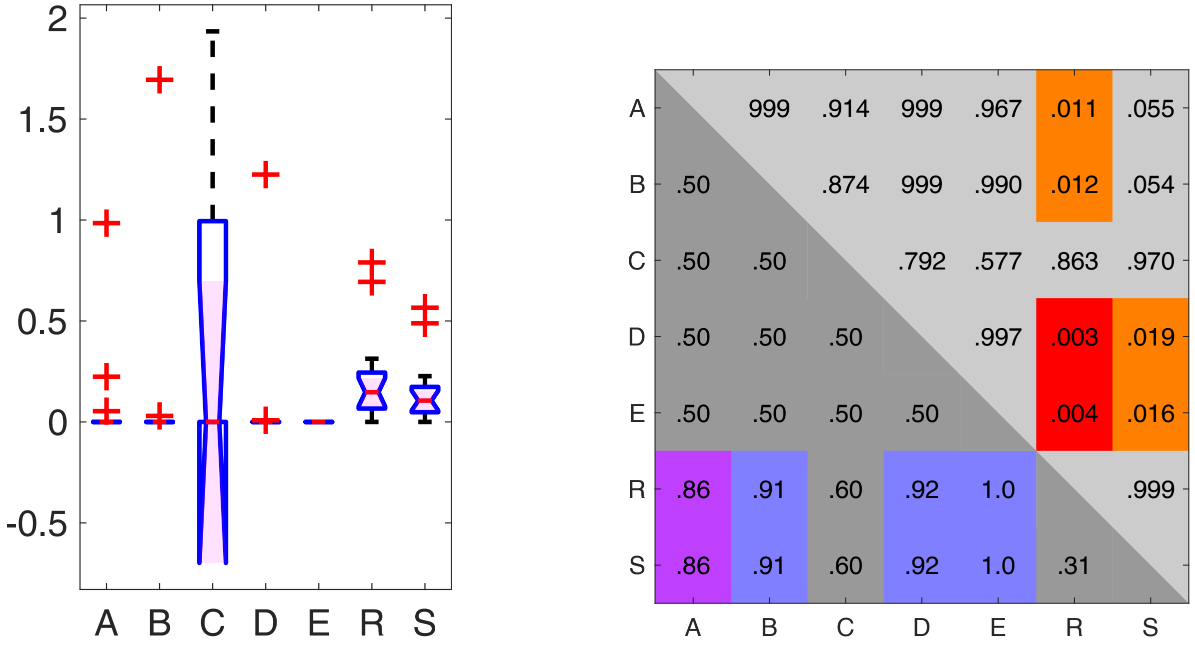

**Heatmap Analysis of Box B4**

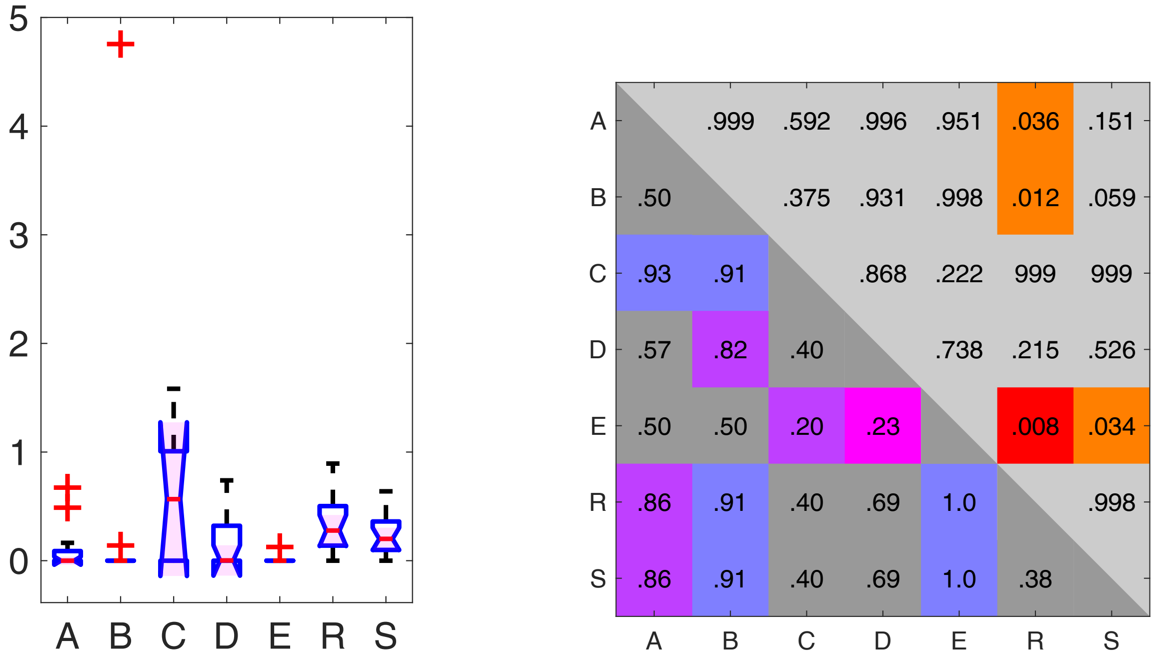


**Heatmap Analysis of Box B5**

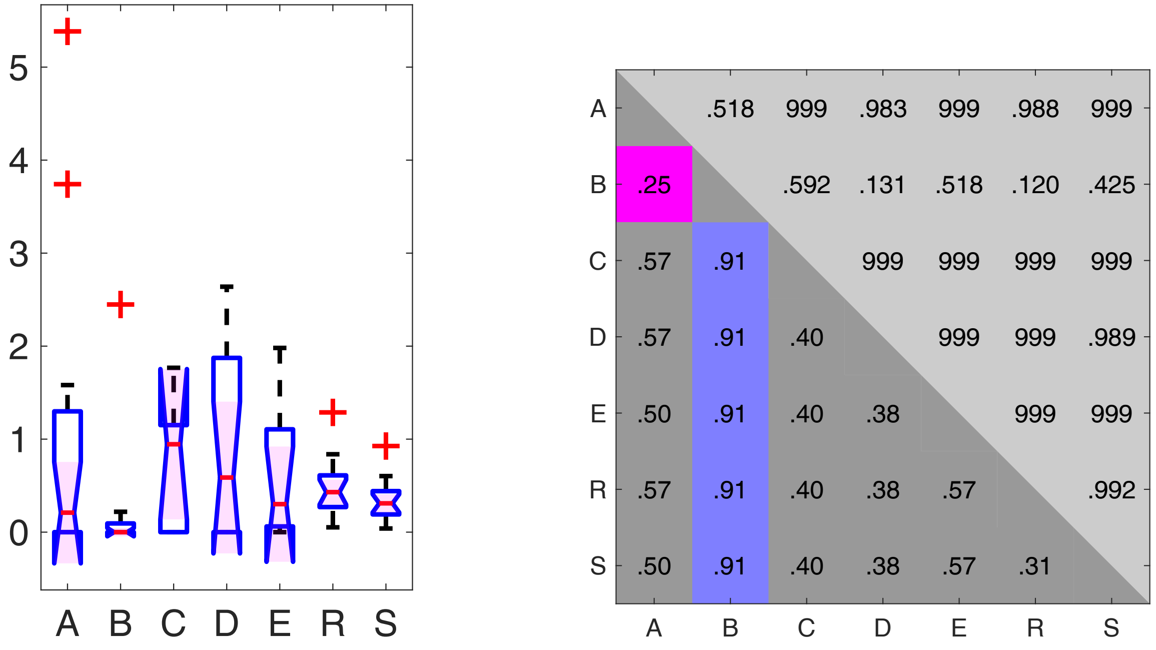

**Heatmap Analysis of Box B6**

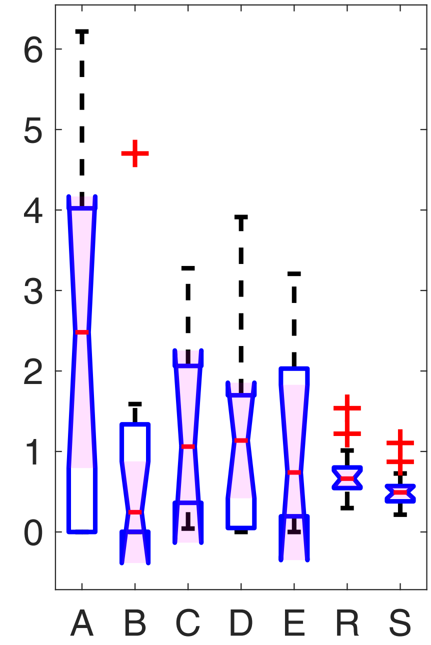


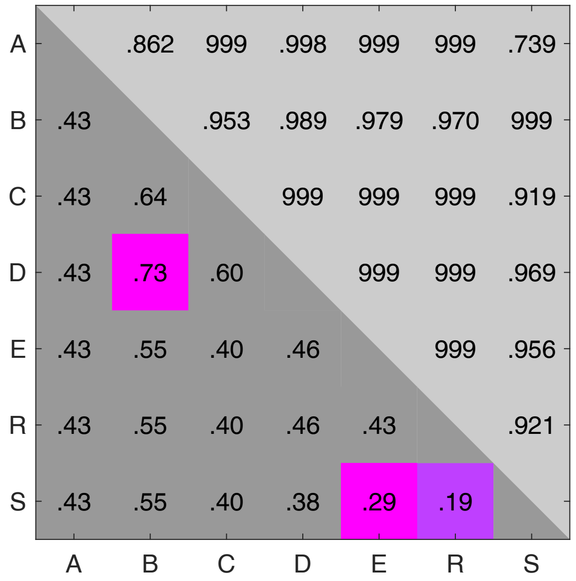


**Heatmap Analysis of Box B7**

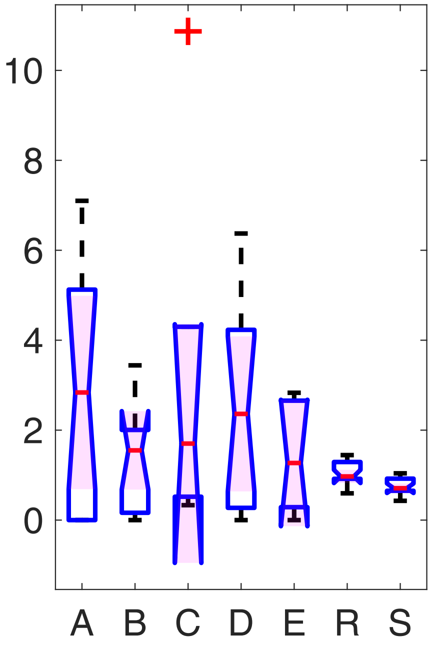


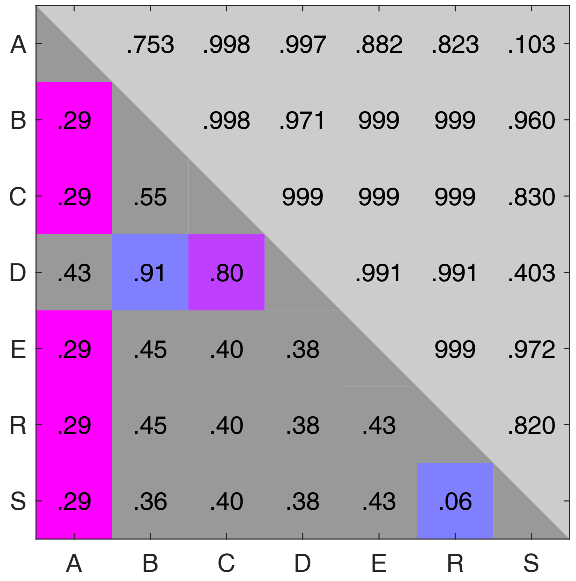

**Heatmap Analysis of Box B8**

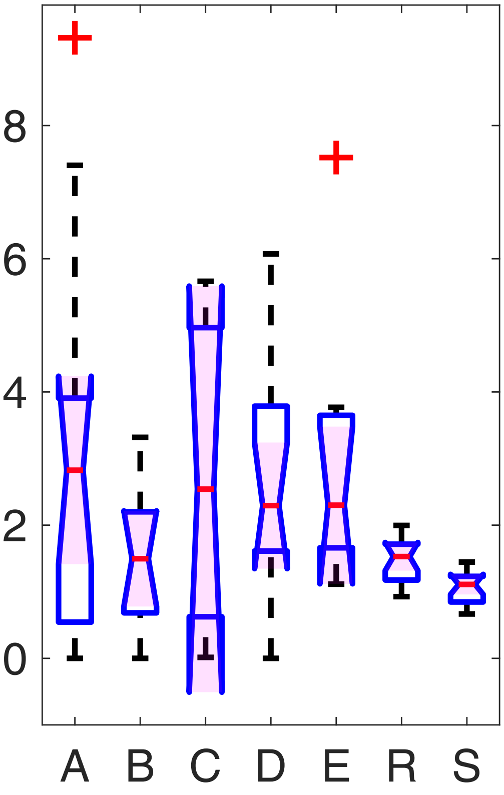


| | A | B | C | D | E | R | S |
|---|---|---|---|---|---|---|---|
| A | | .711 | 999 | 999 | .998 | .757 | .035 |
| B | .29 | | .969 | .536 | .538 | 999 | .868 |
| C | .43 | .82 | | 999 | .995 | .984 | .455 |
| D | .43 | .73 | .40 | | 999 | .571 | .016 |
| E | .43 | .73 | .40 | .54 | | .583 | .040 |
| R | .29 | .55 | .40 | .23 | .14 | | .656 |
| S | .29 | .36 | .40 | .15 | .00 | .25 | |

**Heatmap Analysis of Box B9**

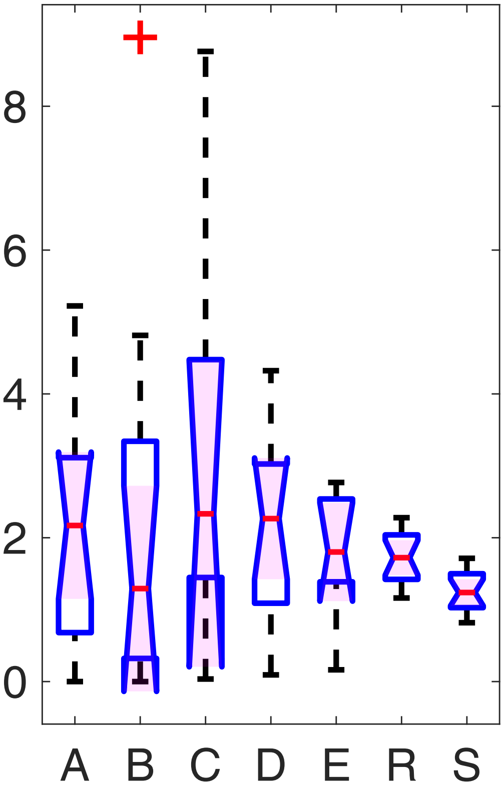


| | A | B | C | D | E | R | S |
|---|---|---|---|---|---|---|---|
| A | | .992 | .990 | 999 | 999 | 999 | .407 |
| B | .36 | | .875 | .967 | .997 | .997 | .914 |
| C | .57 | .64 | | .998 | .994 | .977 | .301 |
| D | .57 | .64 | .40 | | 999 | 999 | .282 |
| E | .43 | .55 | .20 | .38 | | 999 | .671 |
| R | .43 | .55 | .20 | .38 | .43 | | .475 |
| S | .36 | .45 | .20 | .31 | .14 | .12 | |

**Heatmap Analysis of Box BA**

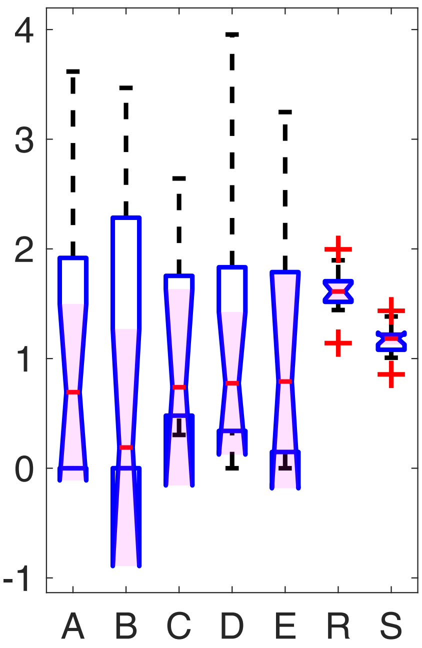


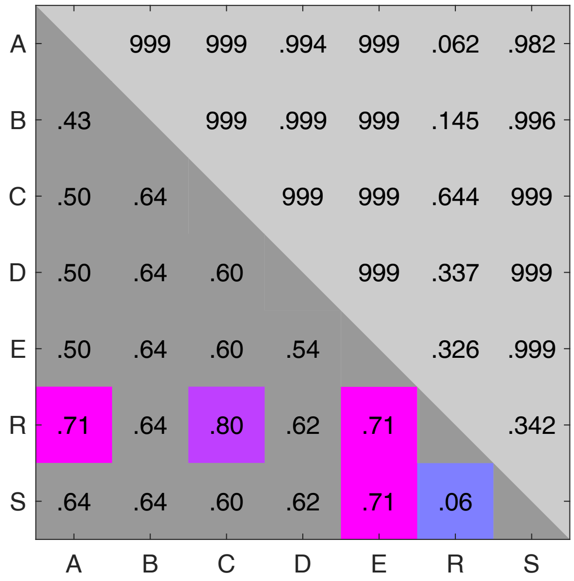


**Heatmap Analysis of Box BB**

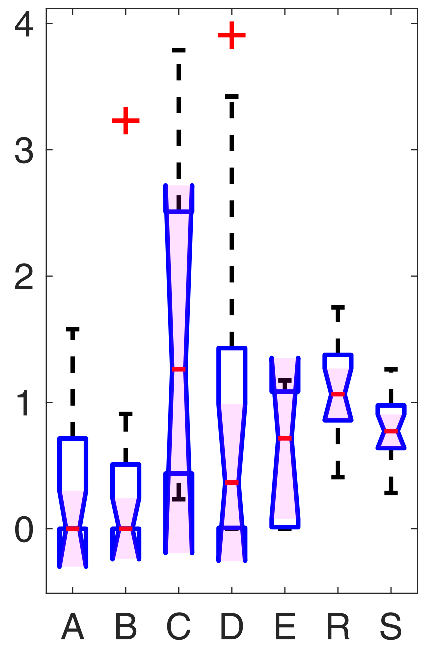


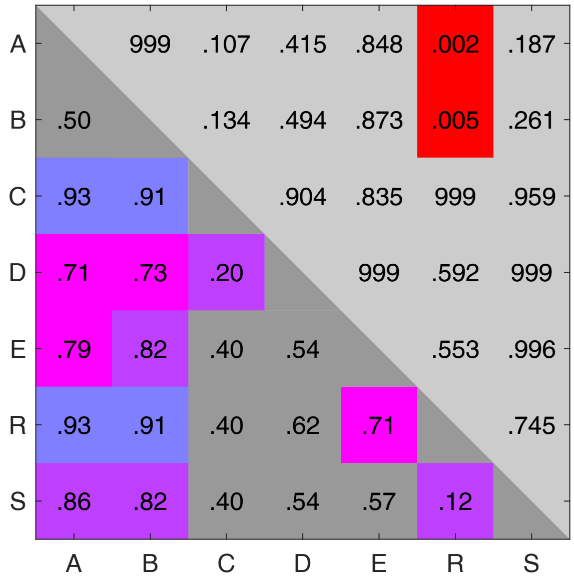

**Heatmap Analysis of Box BC**

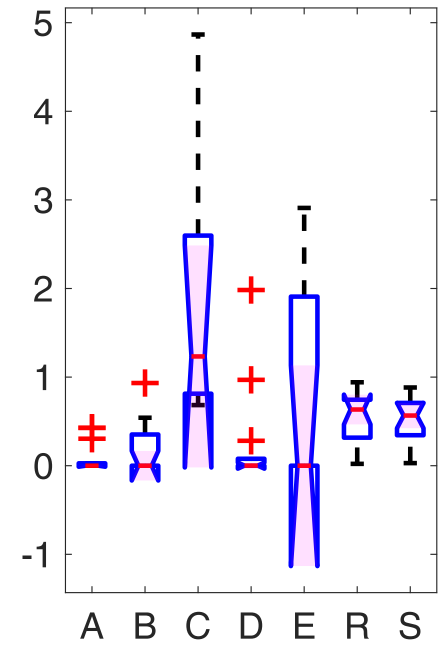


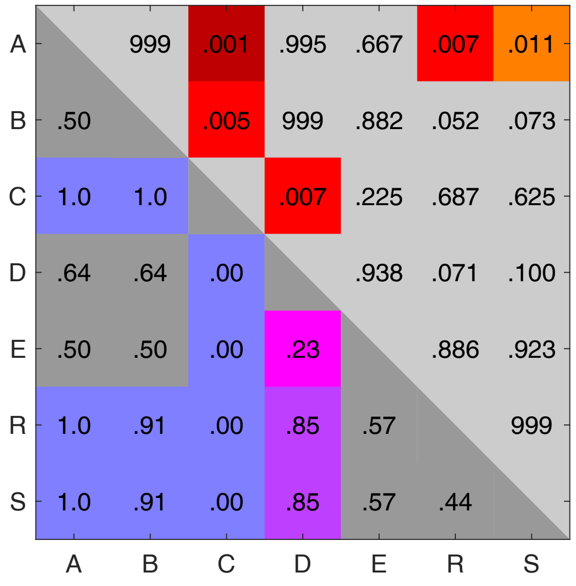


**Heatmap Analysis of Box BD**

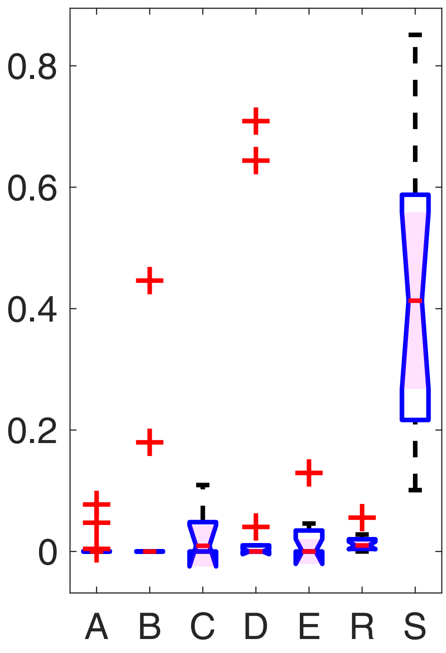


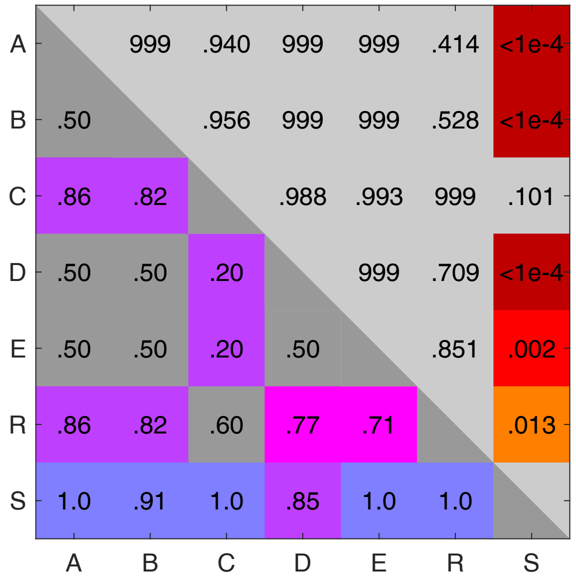

**Heatmap Analysis of Box BE**

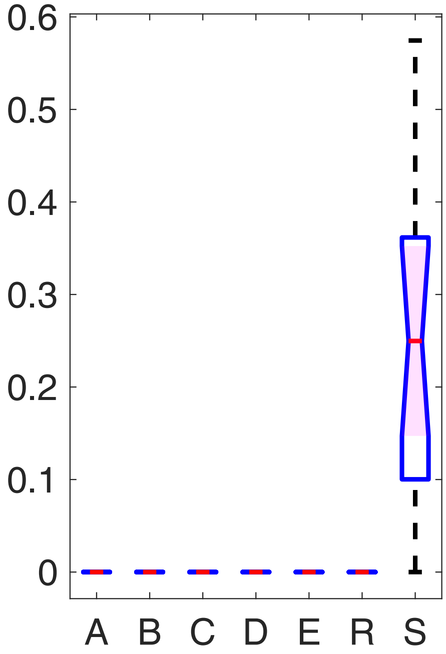


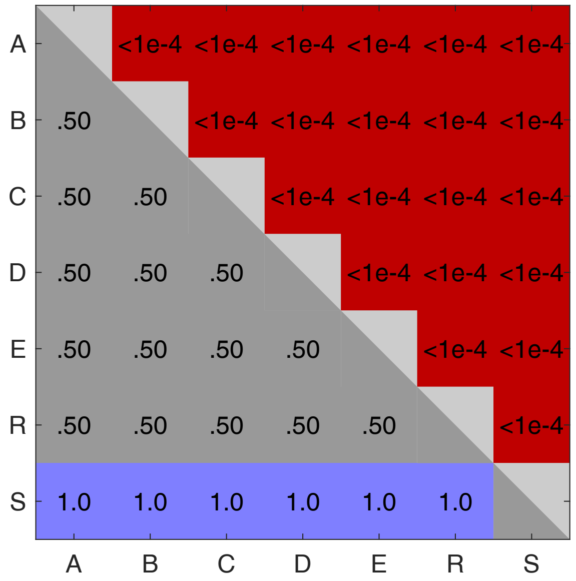


**Heatmap Analysis of Box BF**

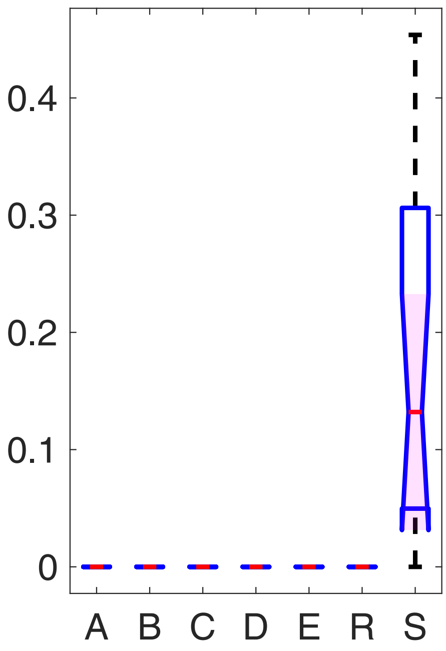


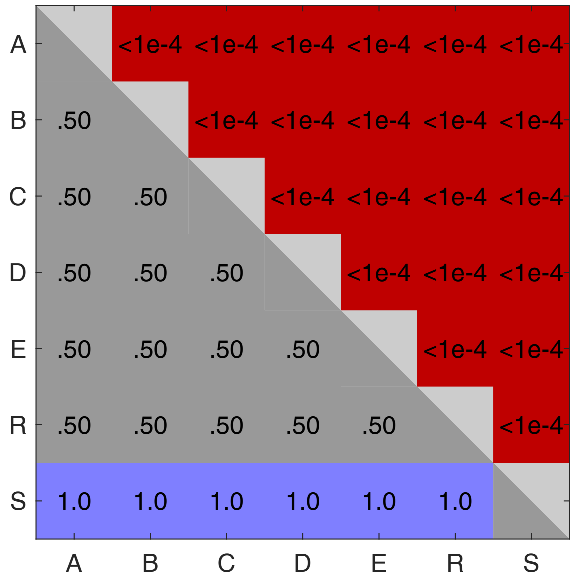

**Heatmap Analysis of Box BG**

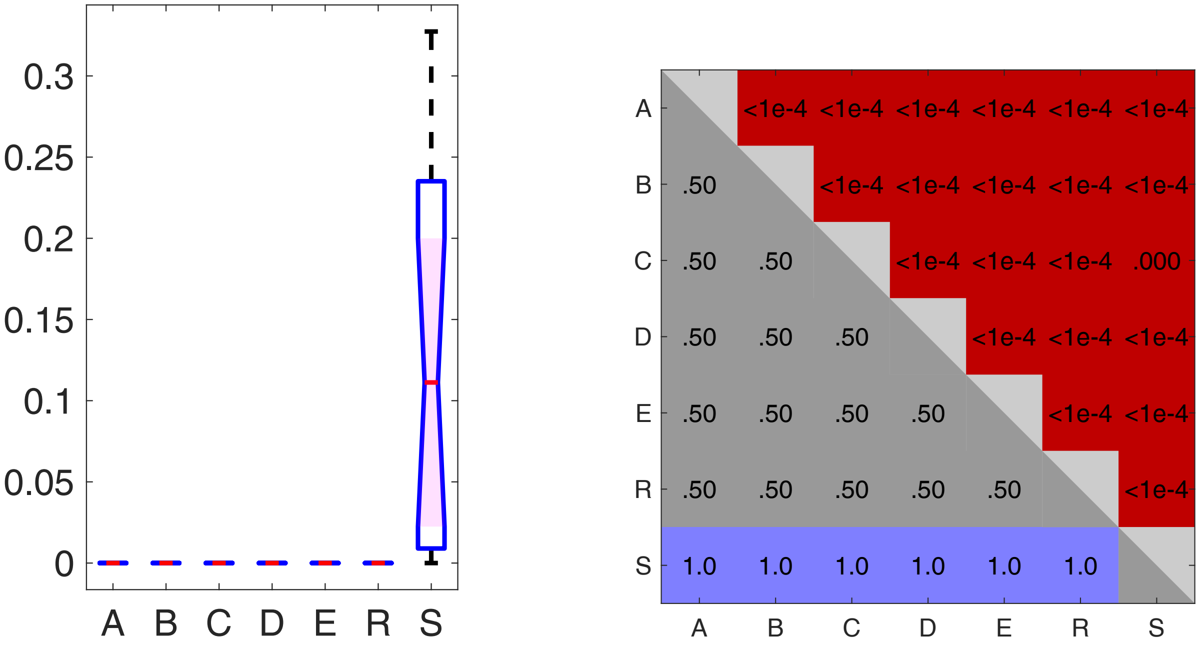


**Heatmap Analysis of Box BH**

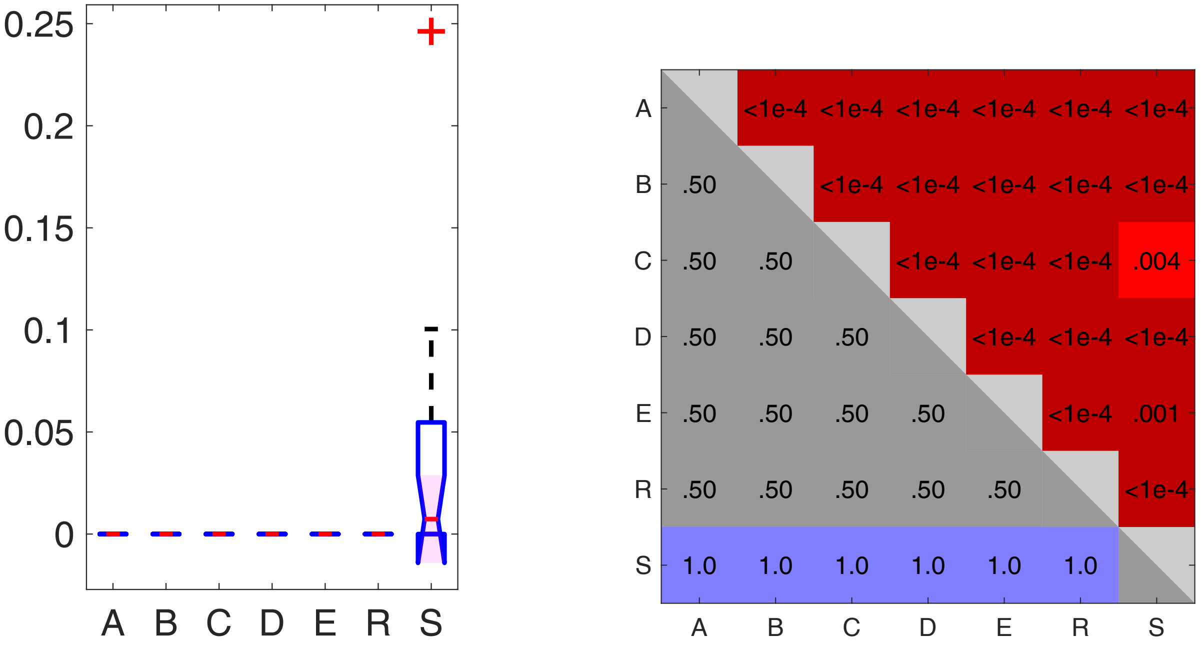

**Heatmap Analysis of Box C0**

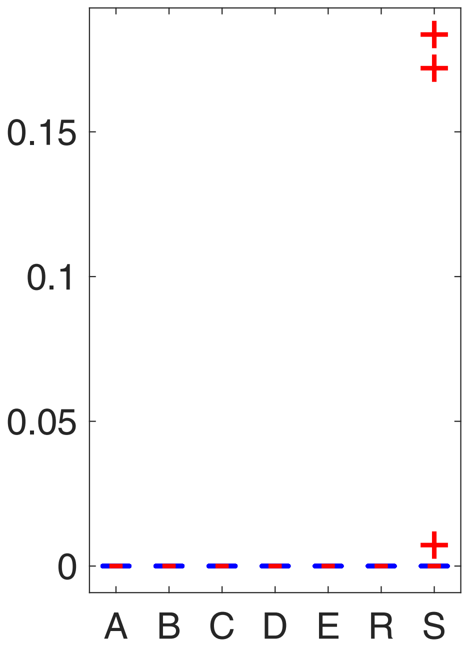


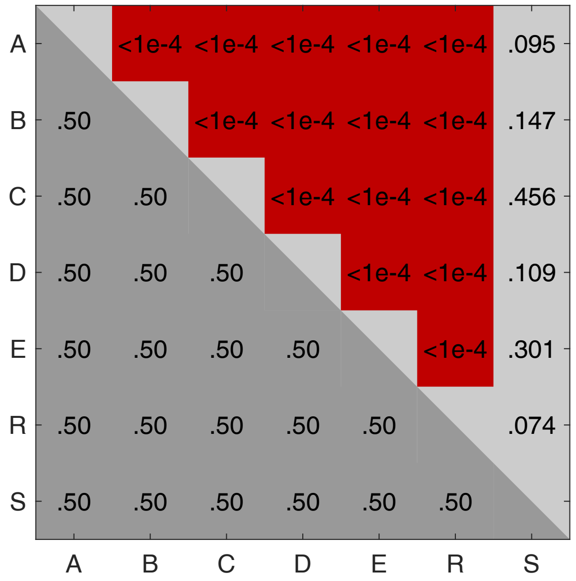


**Heatmap Analysis of Box C1**

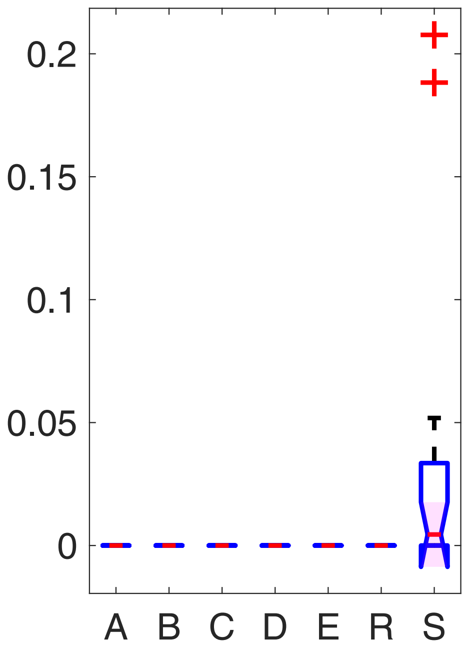


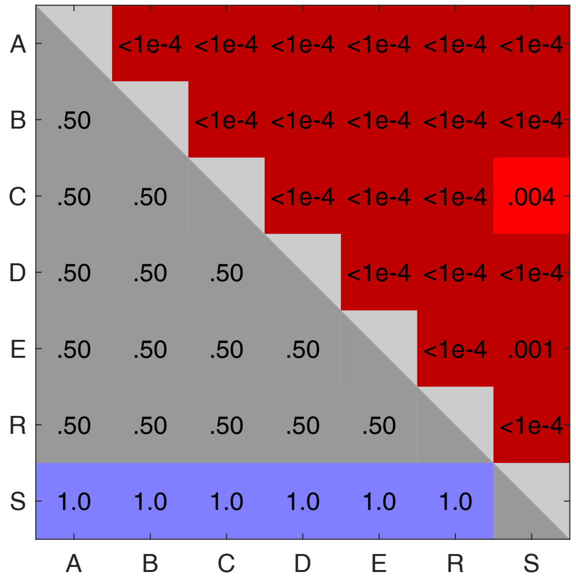

**Heatmap Analysis of Box C2**

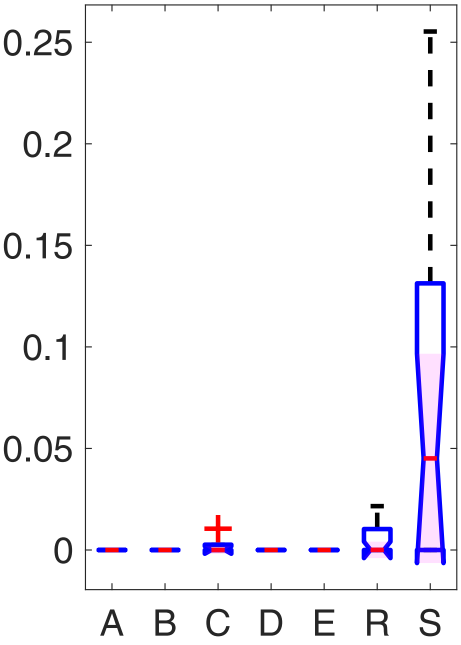


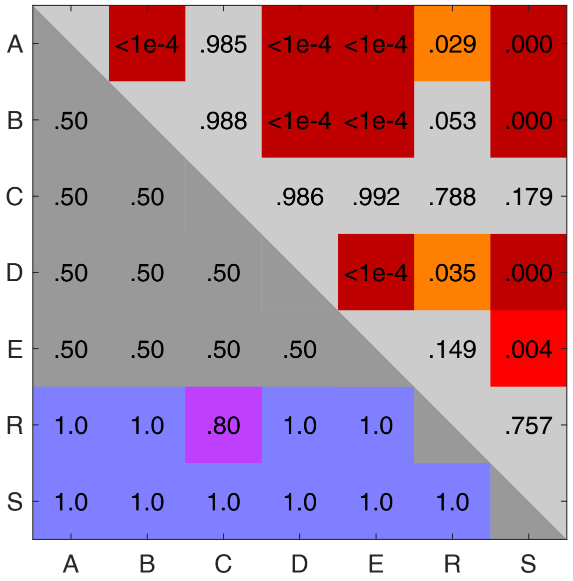


**Heatmap Analysis of Box C3**

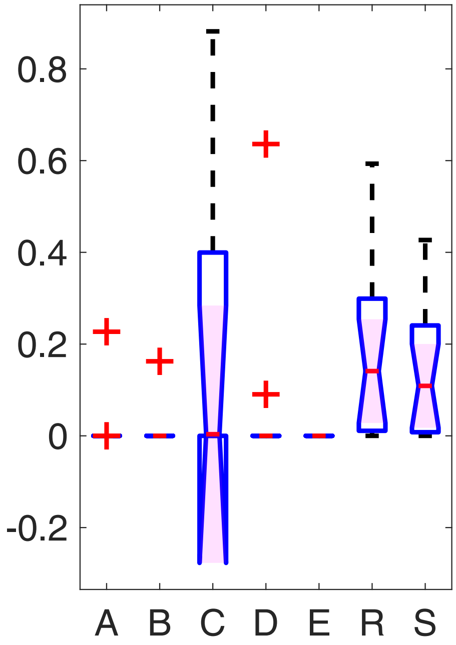


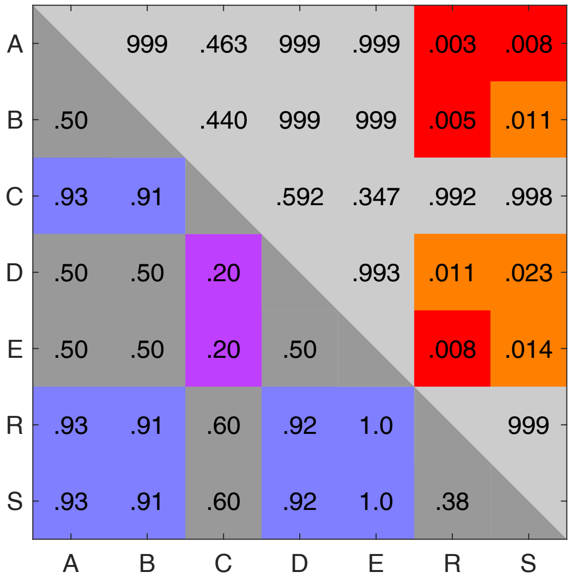

**Heatmap Analysis of Box C4**

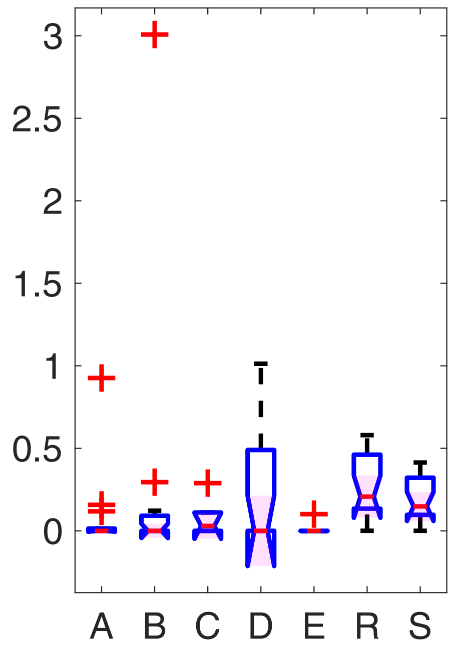


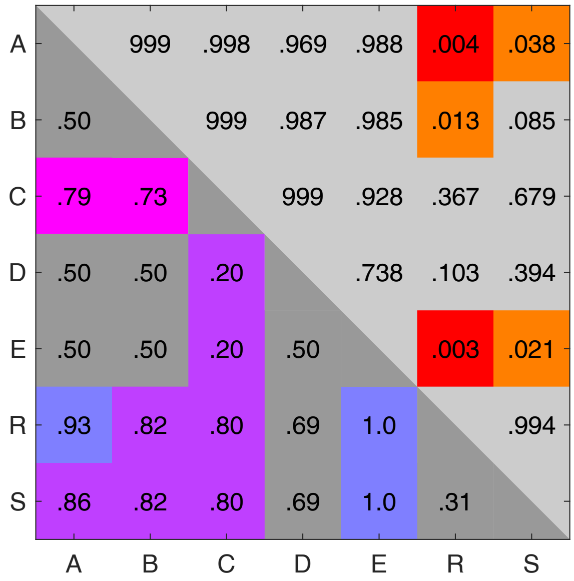


**Heatmap Analysis of Box C5**

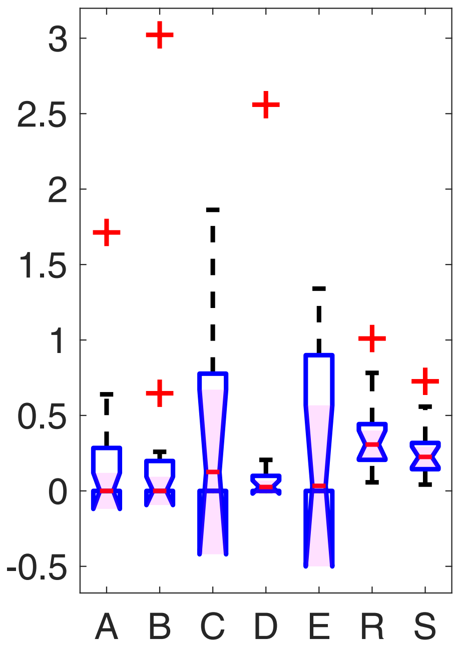


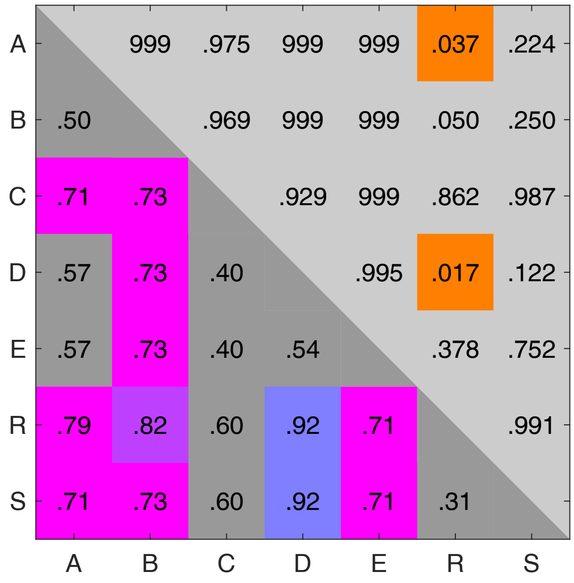

**Heatmap Analysis of Box C6**

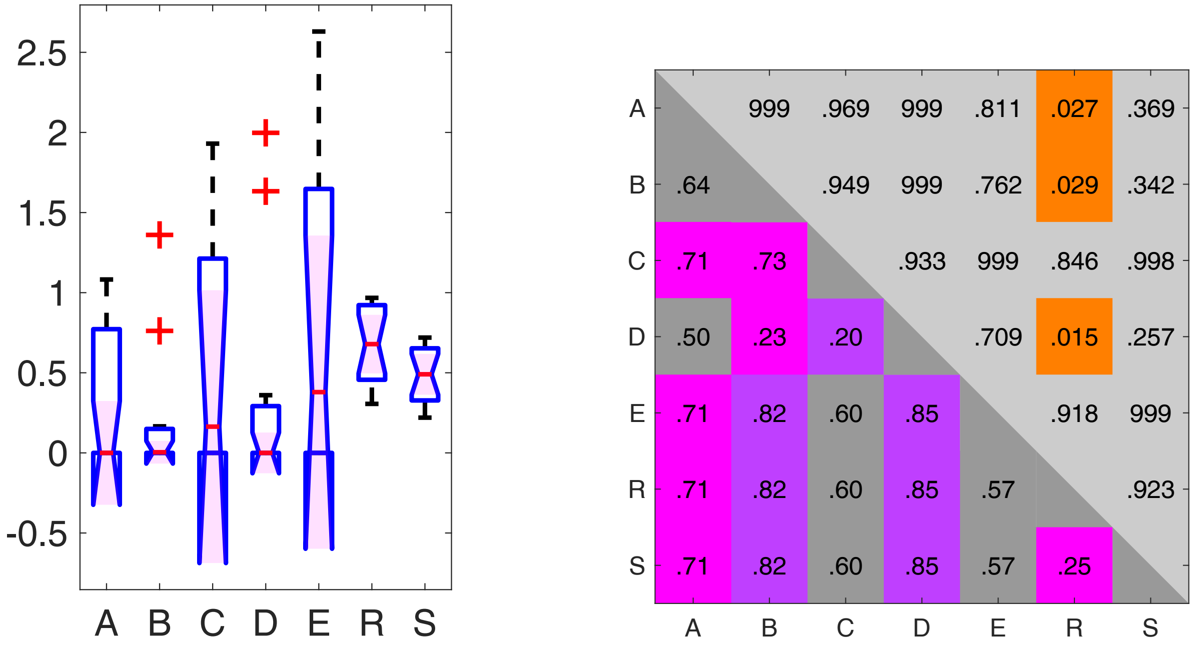


**Heatmap Analysis of Box C7**

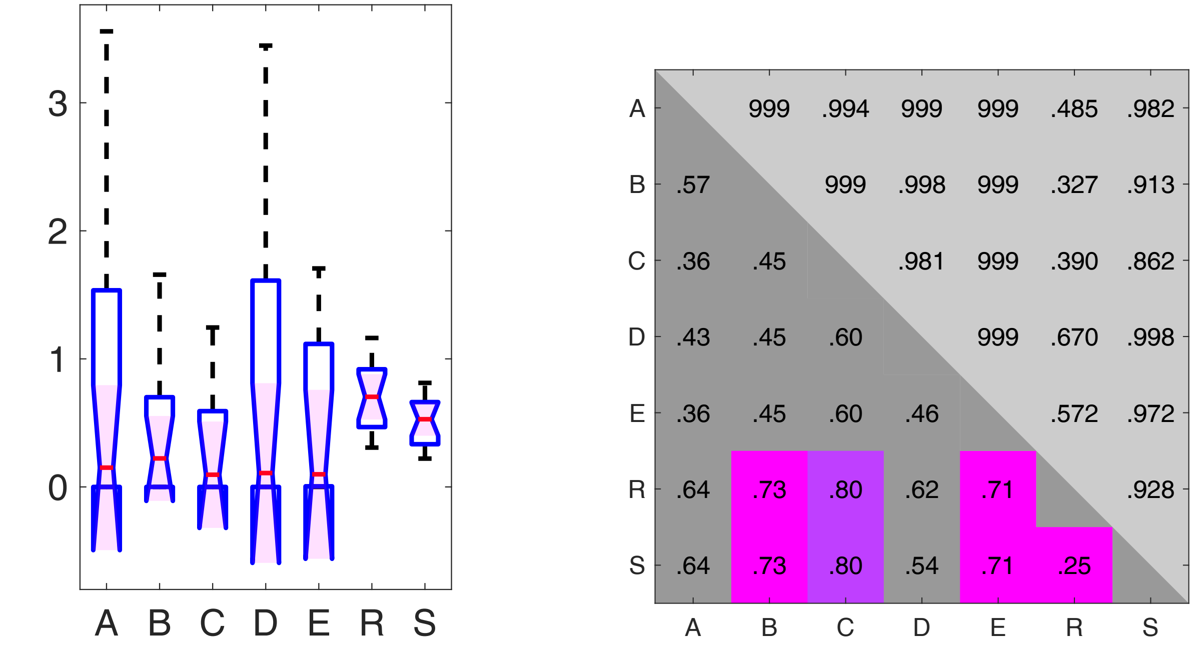

**Heatmap Analysis of Box C8**

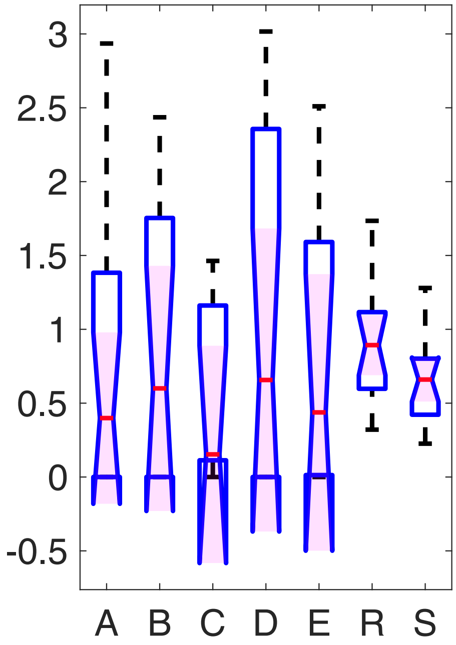


| | A | B | C | D | E | R | S |
|---|---|---|---|---|---|---|---|
| A | | 999 | 999 | .961 | 999 | .753 | 999 |
| B | .64 | | 999 | .999 | 999 | .964 | 999 |
| C | .43 | .36 | | .979 | 999 | .890 | 999 |
| D | .64 | .55 | .60 | | .997 | 999 | .998 |
| E | .50 | .45 | .60 | .38 | | .953 | 999 |
| R | .64 | .55 | .60 | .54 | .57 | | .934 |
| S | .64 | .55 | .60 | .54 | .57 | .31 | |

**Heatmap Analysis of Box C9**

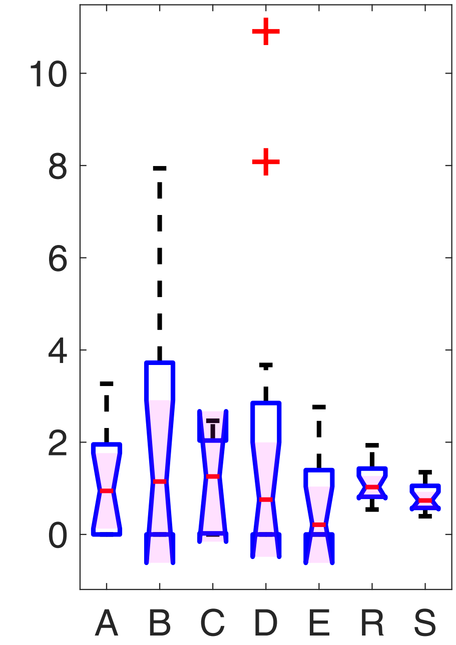


| | A | B | C | D | E | R | S |
|---|---|---|---|---|---|---|---|
| A | | 999 | 999 | 999 | .987 | .962 | 999 |
| B | .64 | | 999 | 999 | .928 | 999 | .997 |
| C | .64 | .55 | | 999 | .958 | 999 | .998 |
| D | .43 | .45 | .40 | | .943 | .997 | .999 |
| E | .43 | .45 | .40 | .38 | | .698 | .995 |
| R | .57 | .45 | .40 | .54 | .71 | | .900 |
| S | .43 | .45 | .40 | .46 | .71 | .12 | |

**Heatmap Analysis of Box CA**

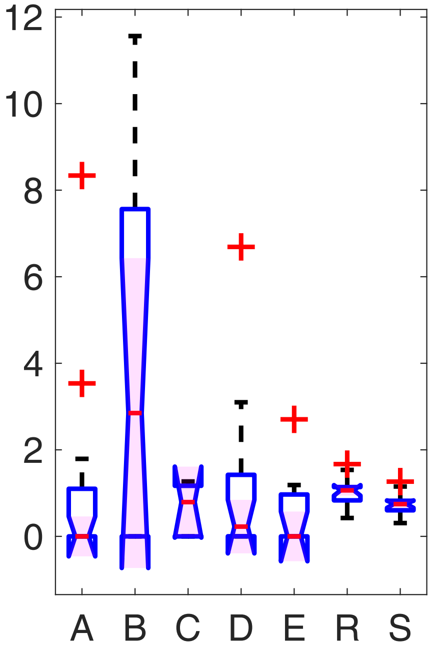


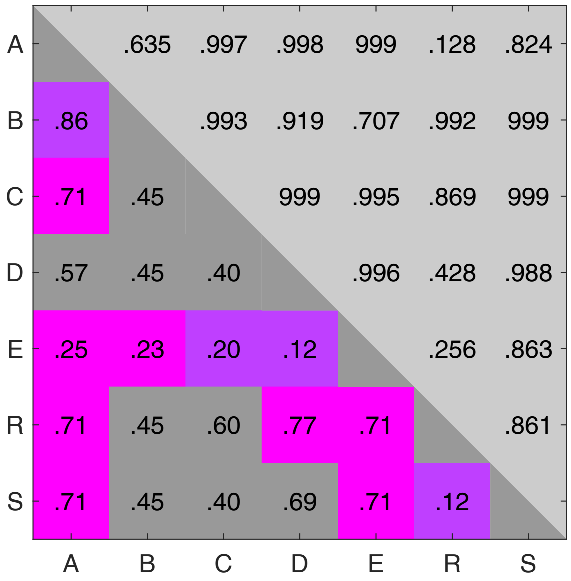


**Heatmap Analysis of Box CB**

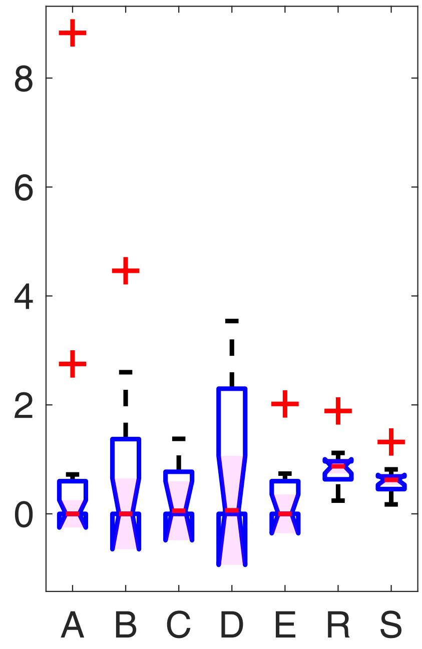


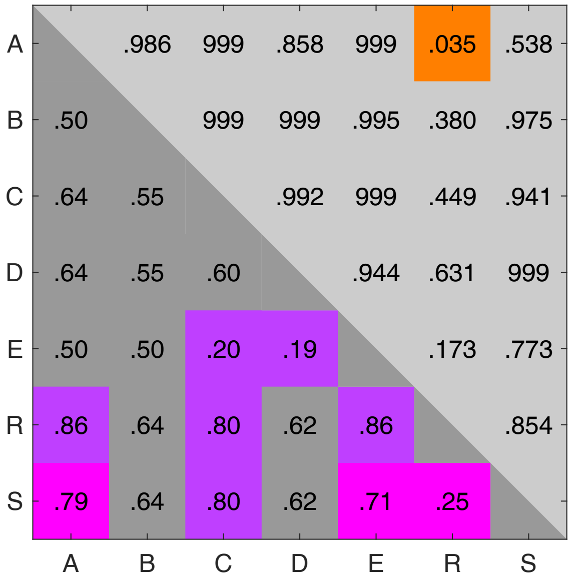

**Heatmap Analysis of Box CC**

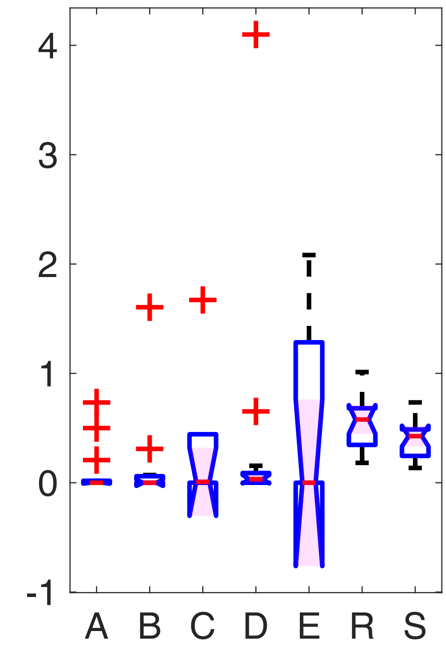


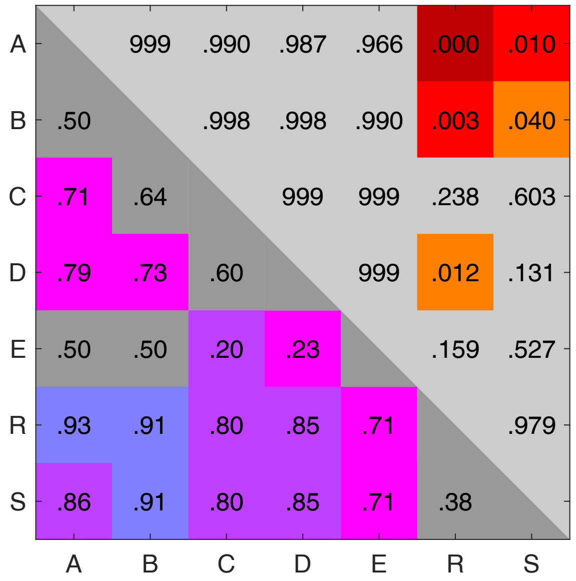


**Heatmap Analysis of Box CD**

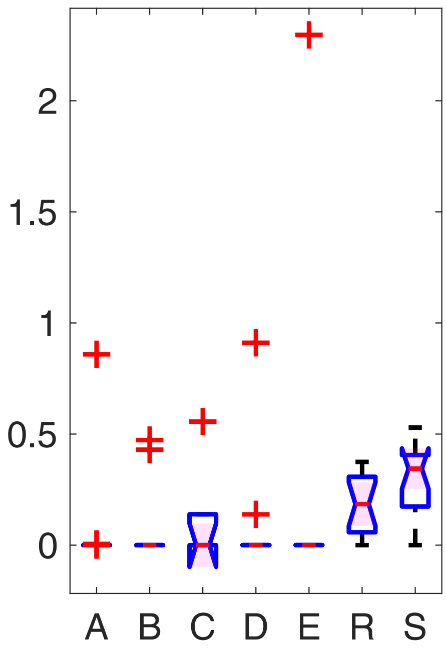


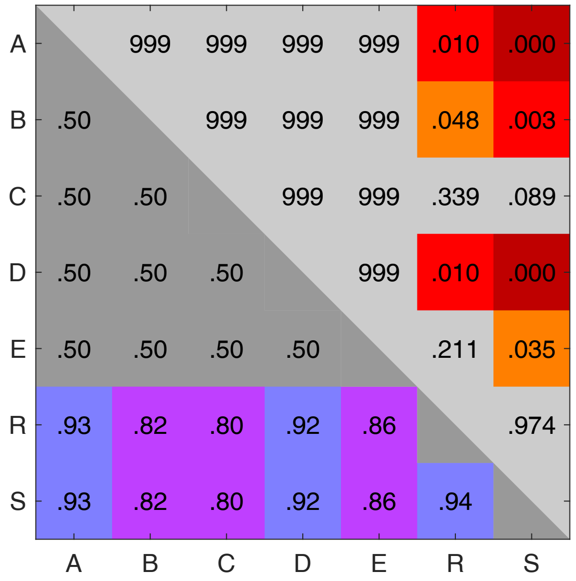

**Heatmap Analysis of Box CE**

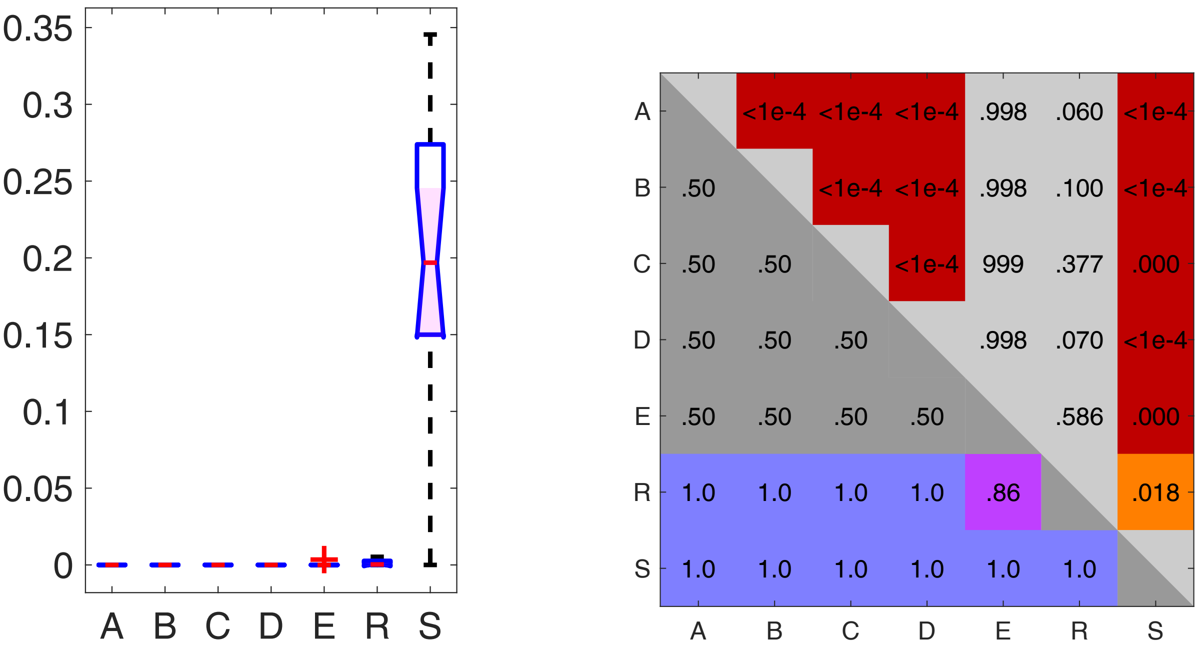


**Heatmap Analysis of Box CF**

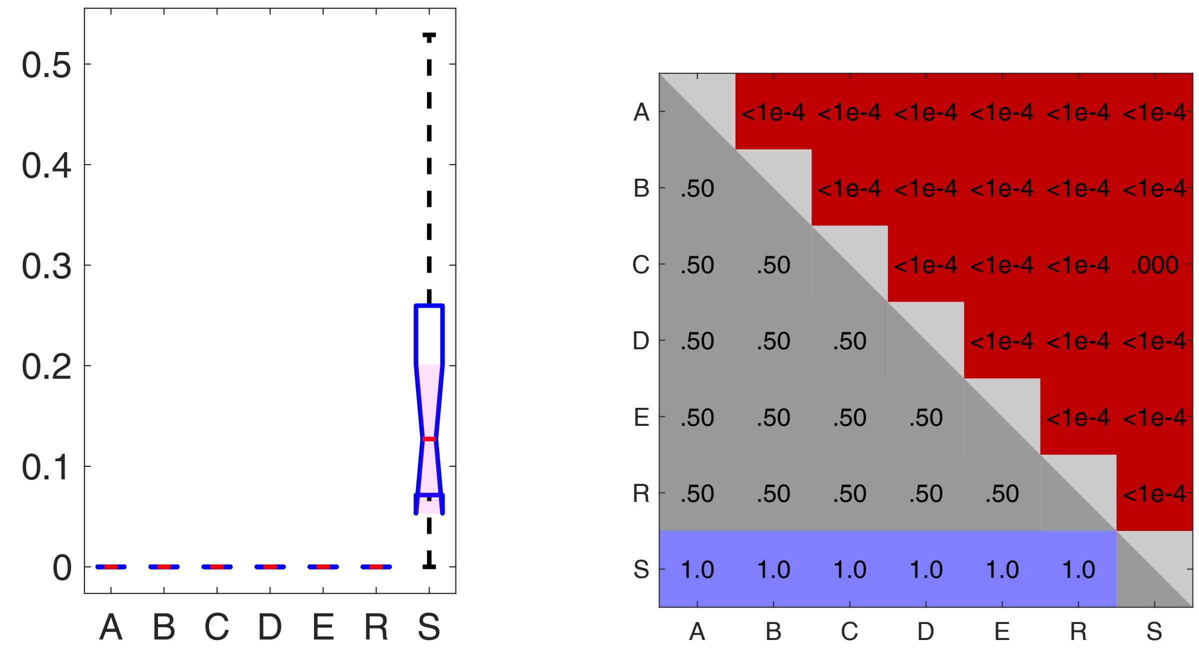

**Heatmap Analysis of Box CG**

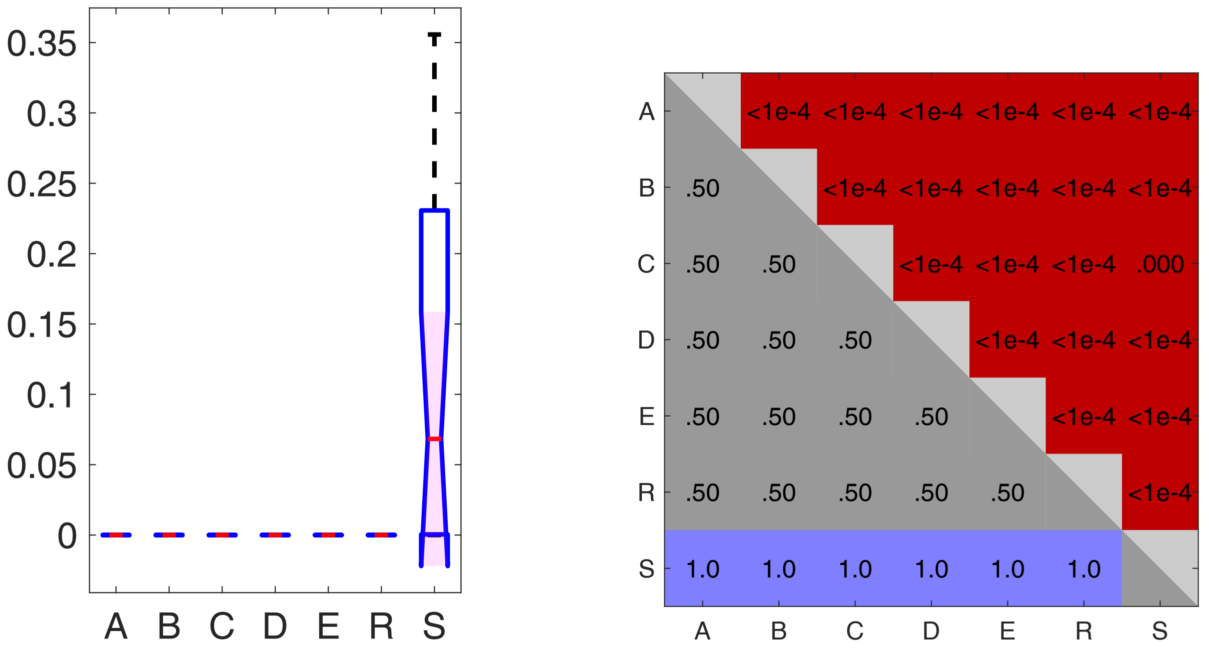


**Heatmap Analysis of Box CH**

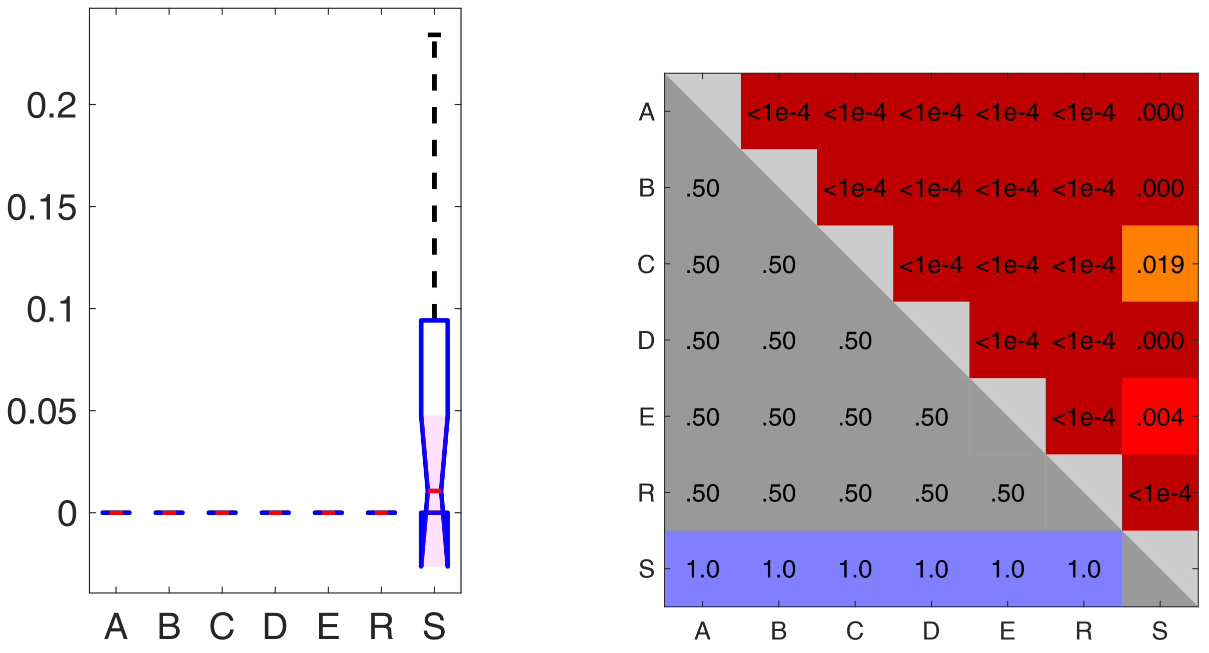

**Heatmap Analysis of Box D0**

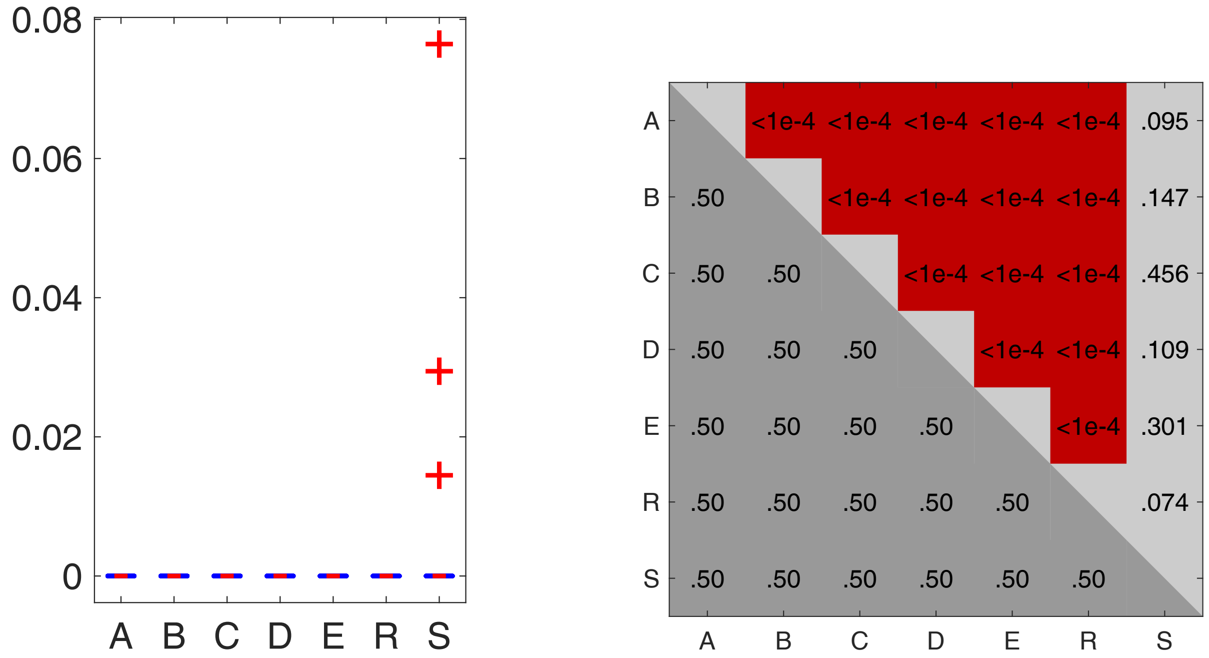


**Heatmap Analysis of Box D1**

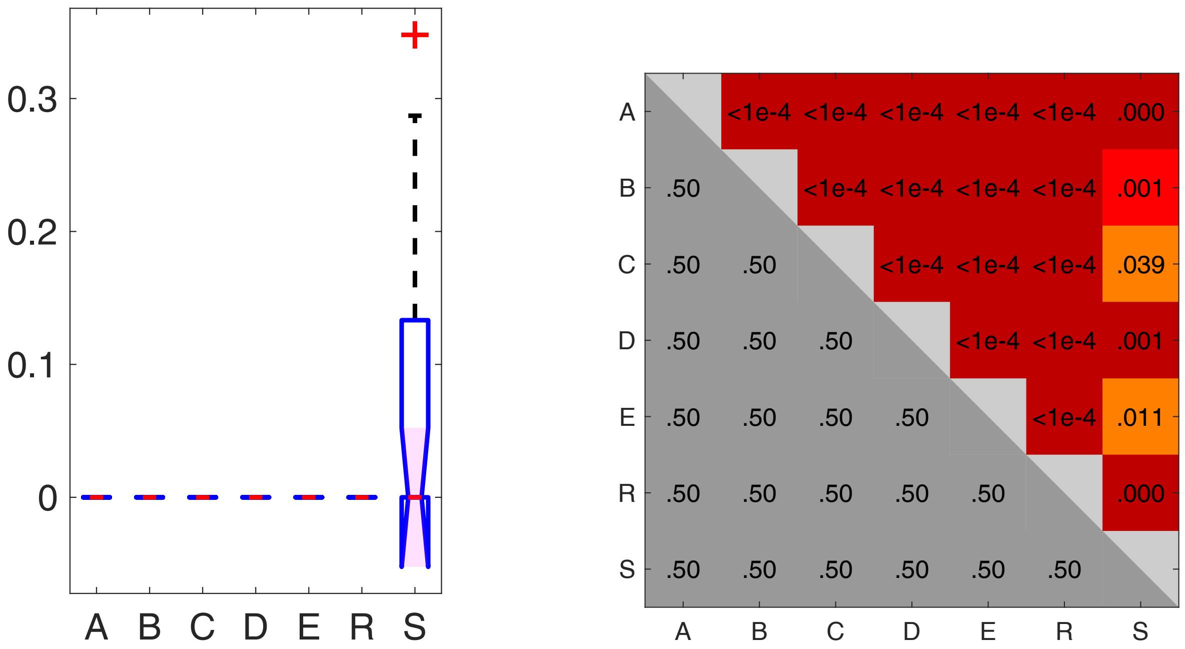

**Heatmap Analysis of Box D2**

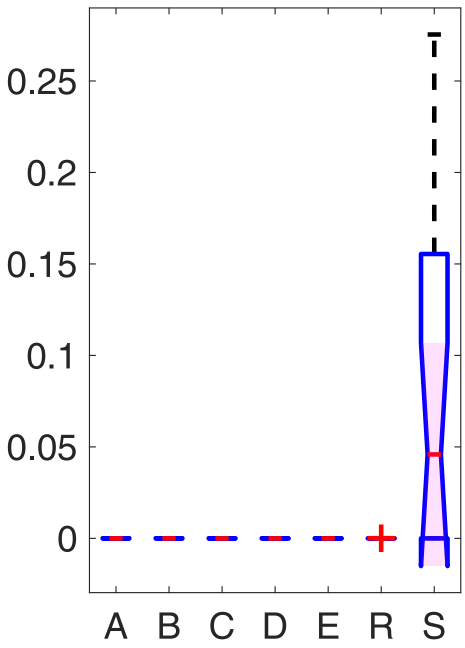


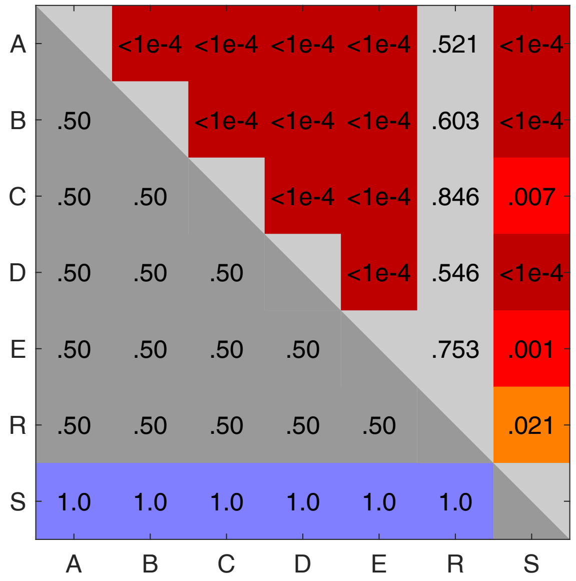


**Heatmap Analysis of Box D3**

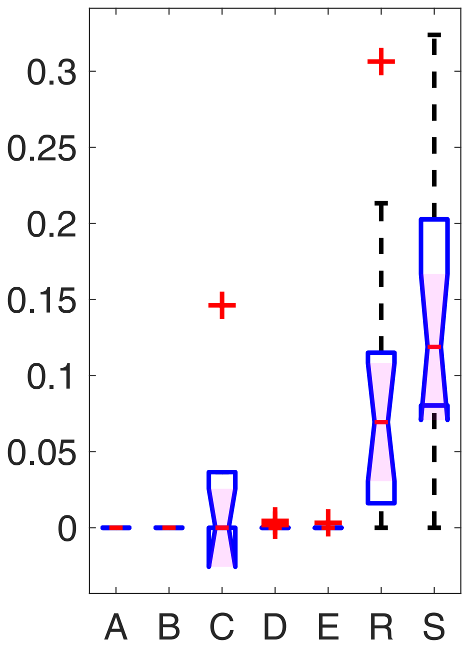


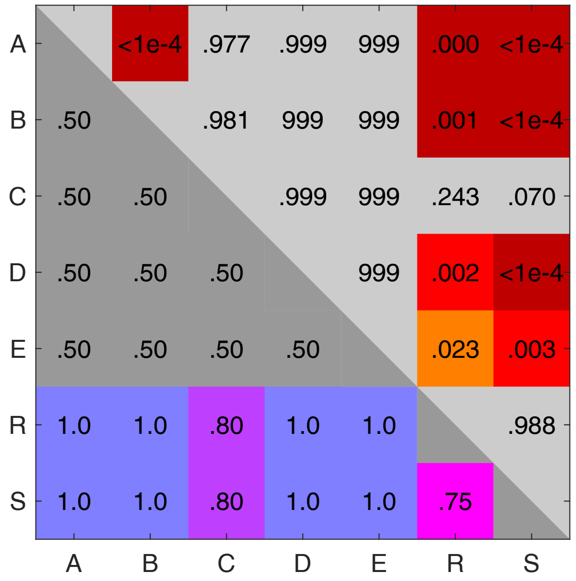

**Heatmap Analysis of Box D4**

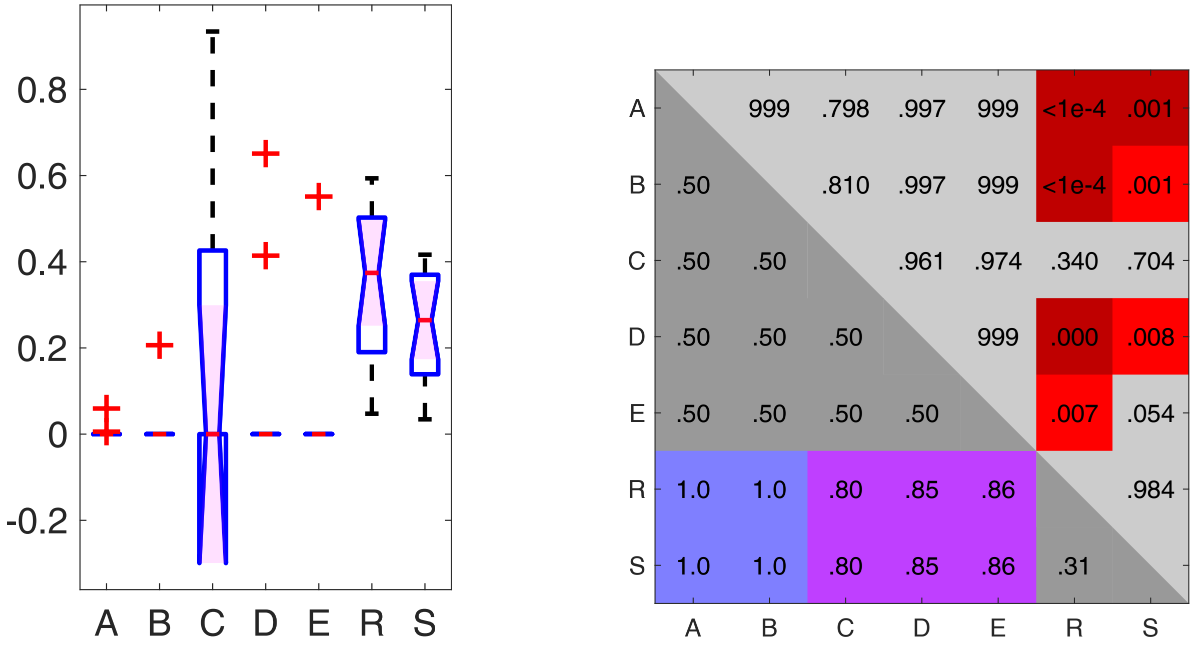


**Heatmap Analysis of Box D5**

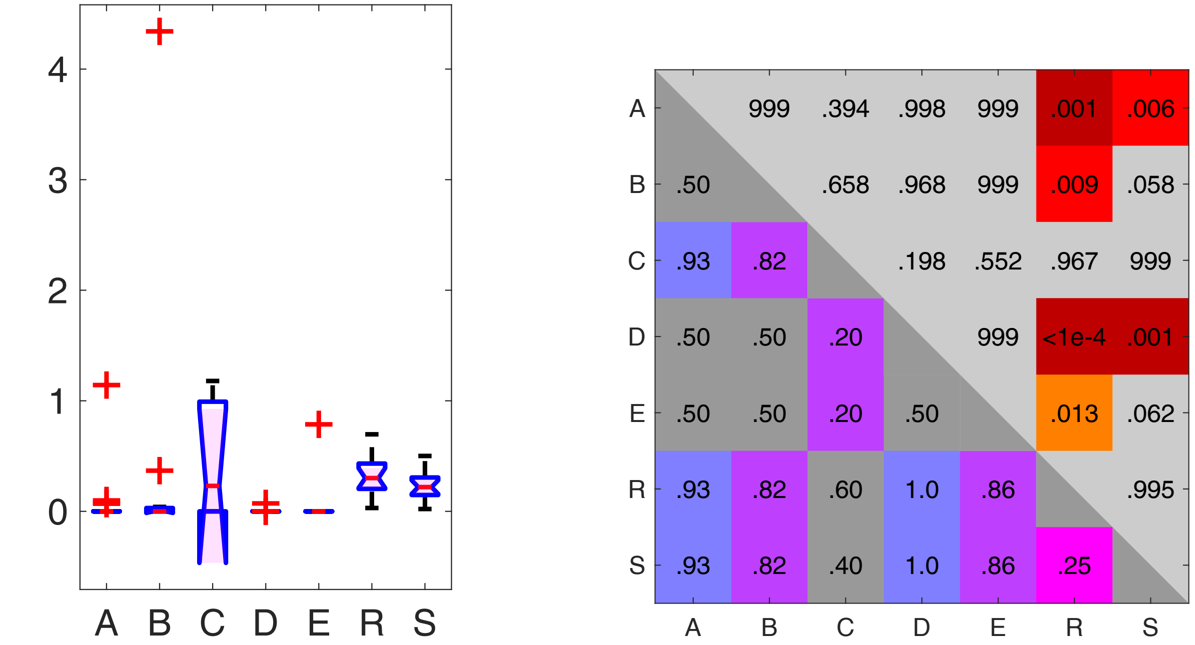

**Heatmap Analysis of Box D6**

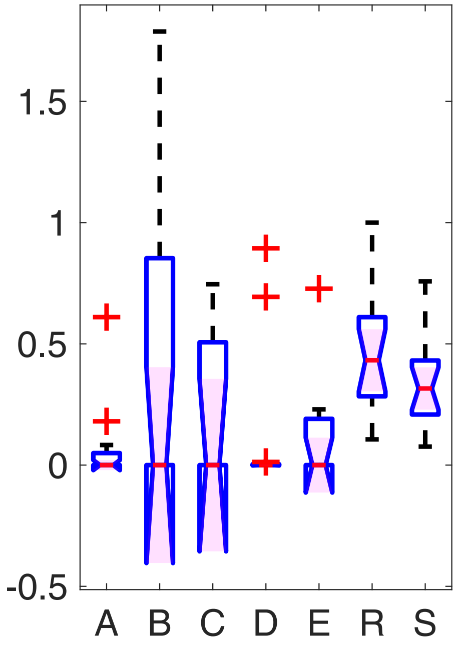


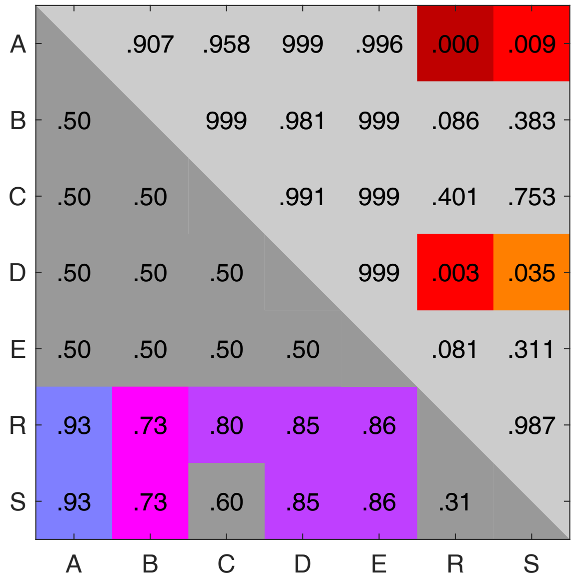


**Heatmap Analysis of Box D7**

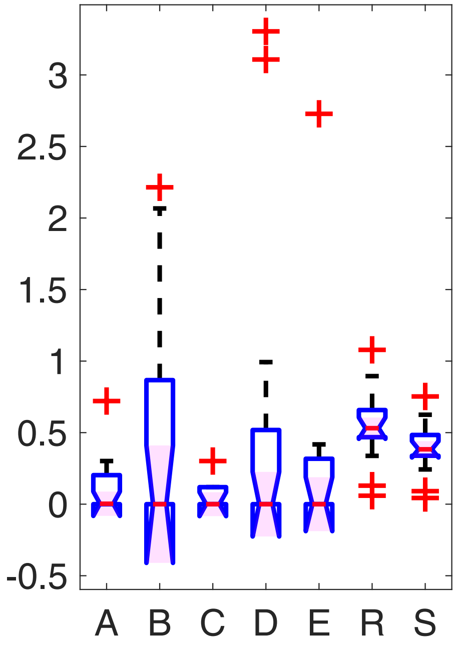


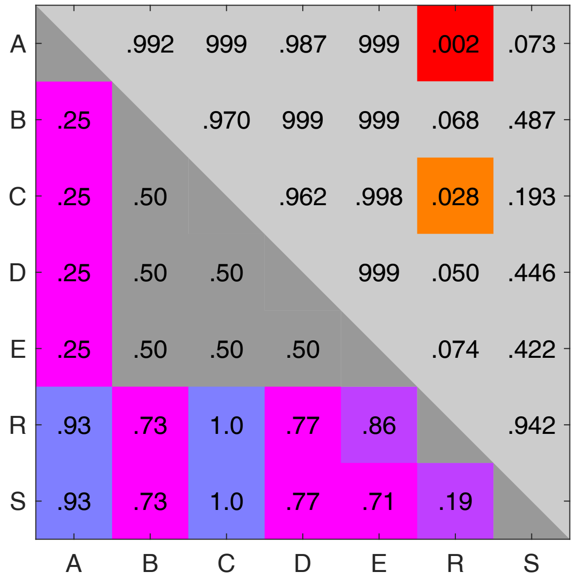

**Heatmap Analysis of Box D8**

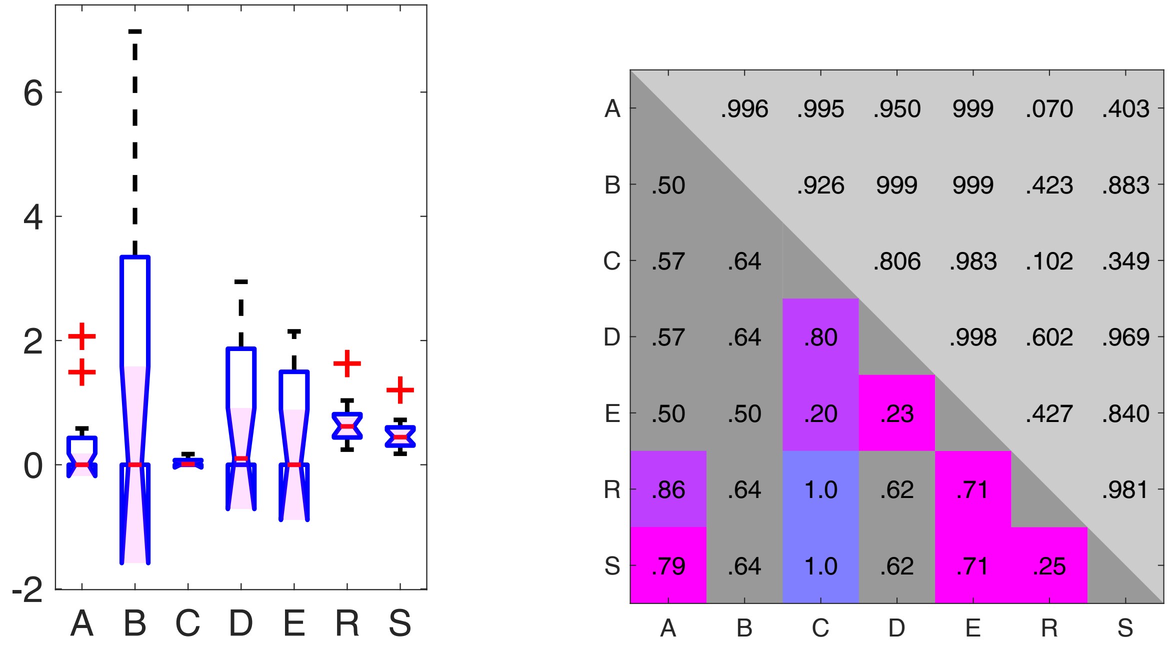


**Heatmap Analysis of Box D9**

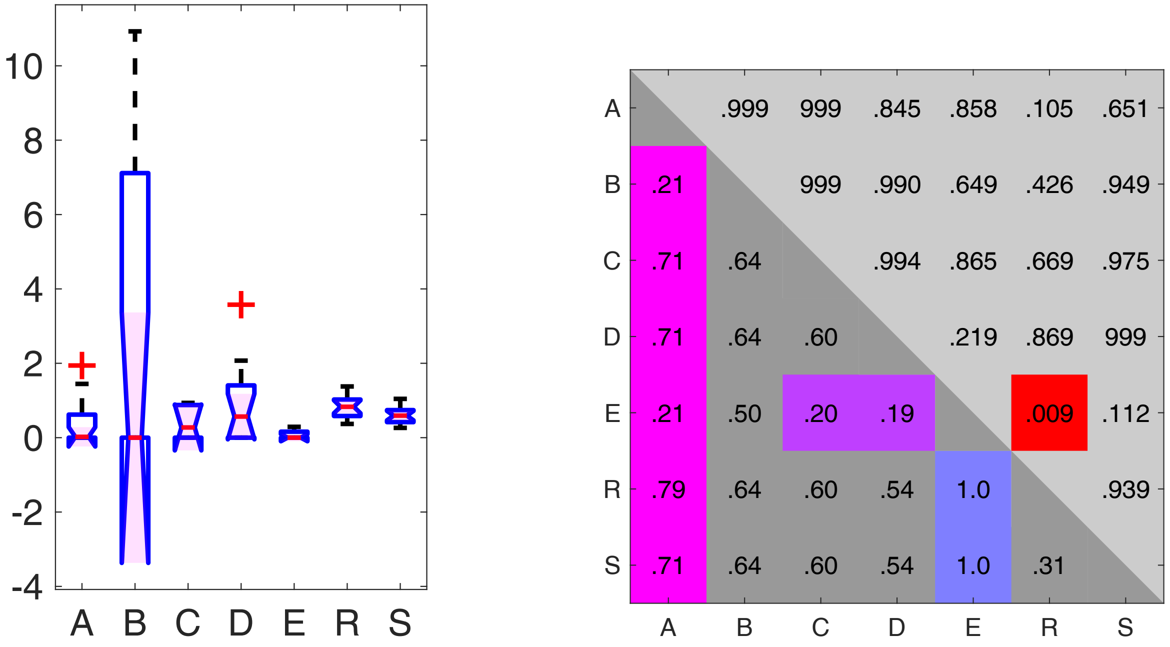

**Heatmap Analysis of Box DA**

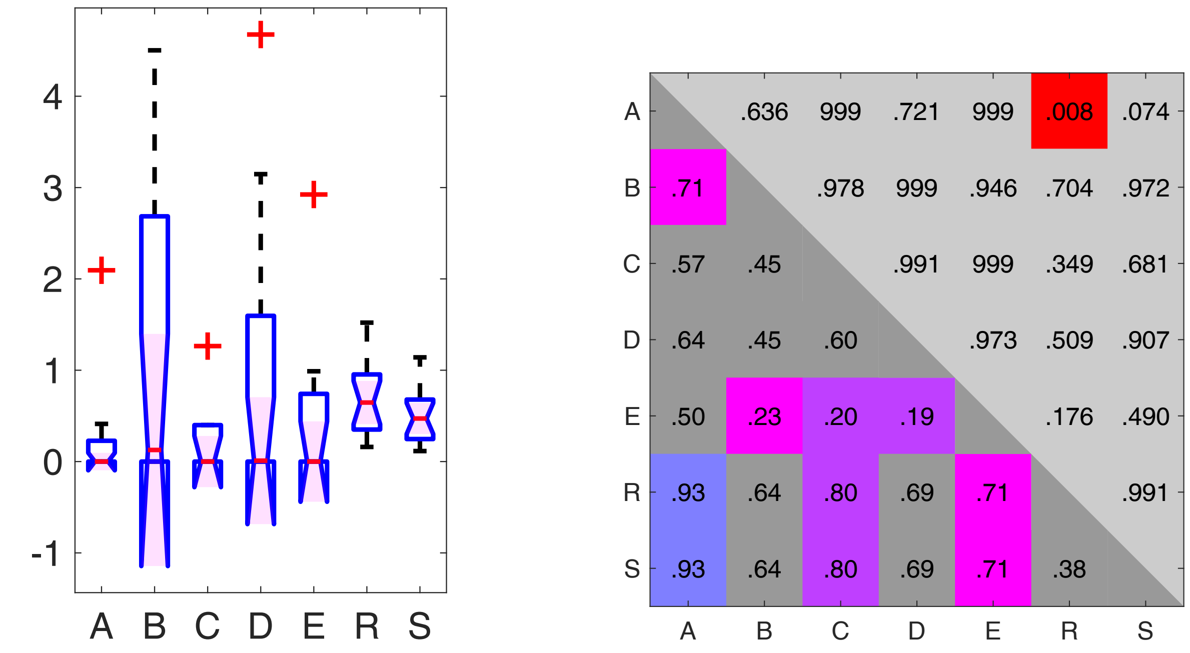


**Heatmap Analysis of Box DB**

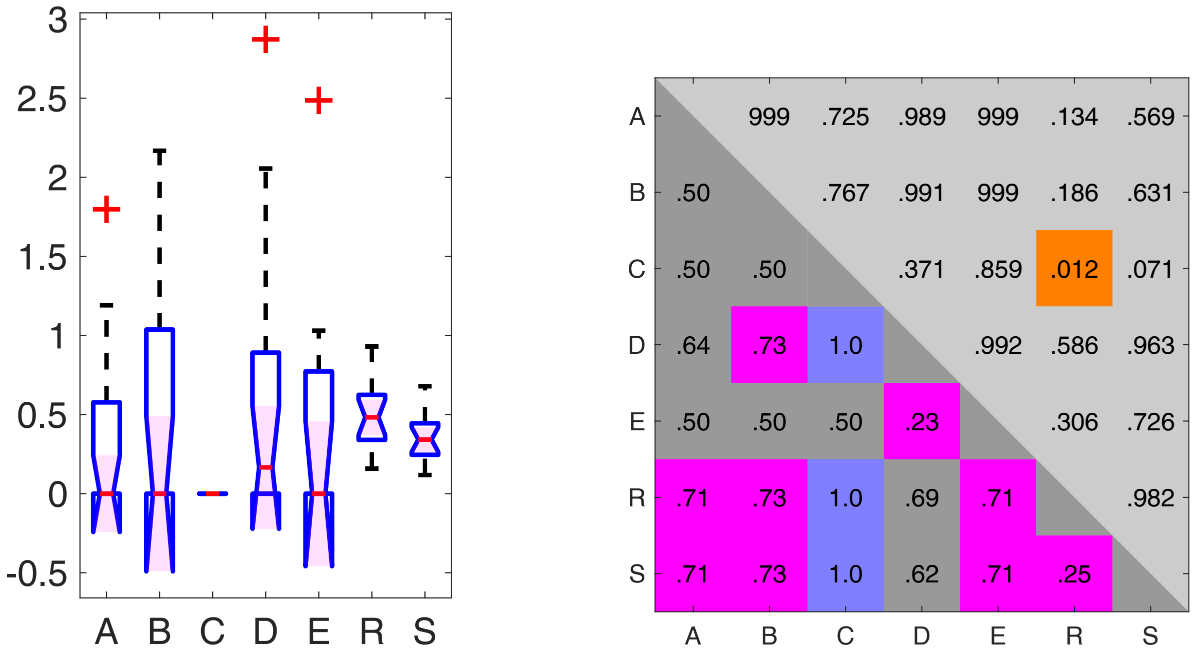

**Heatmap Analysis of Box DC**

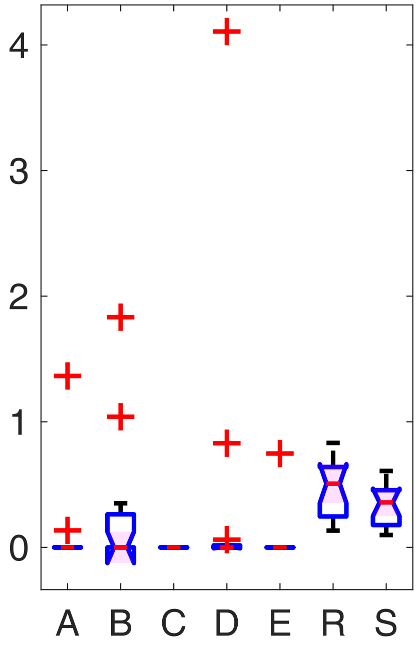


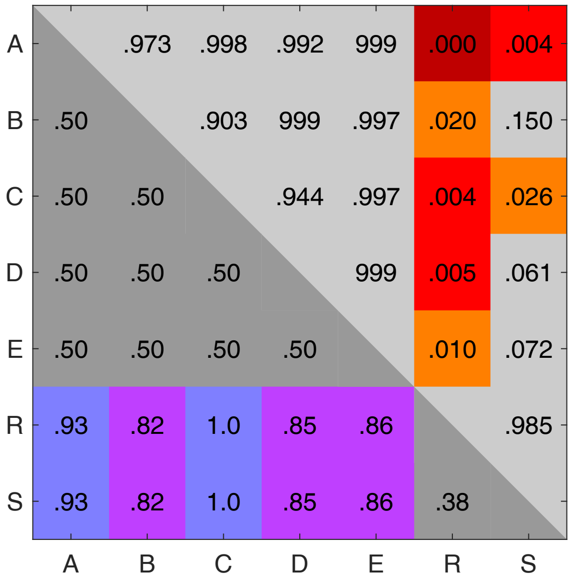


**Heatmap Analysis of Box DD**

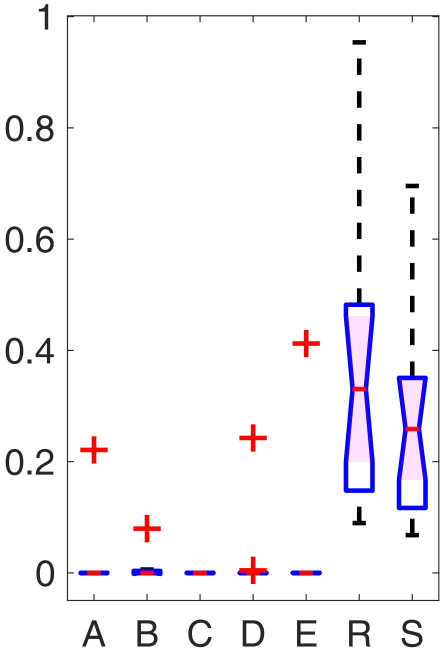


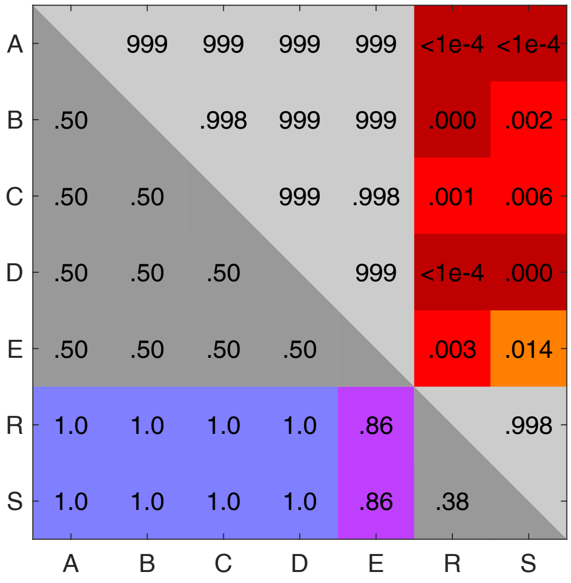

**Heatmap Analysis of Box DE**

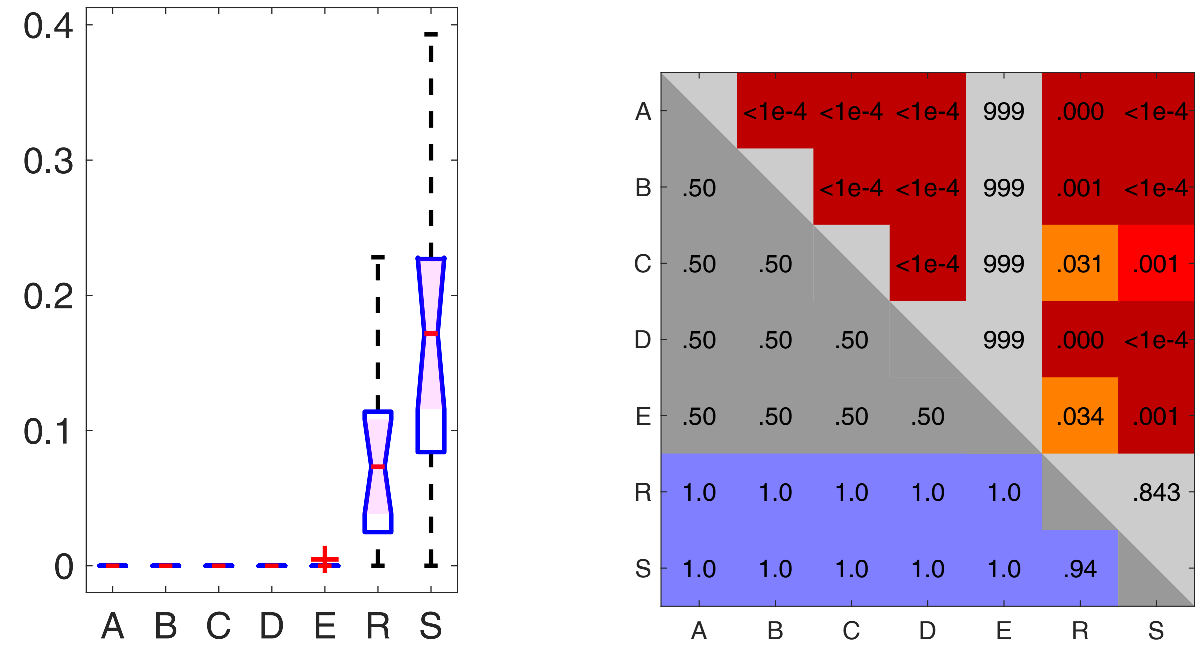


**Heatmap Analysis of Box DF**

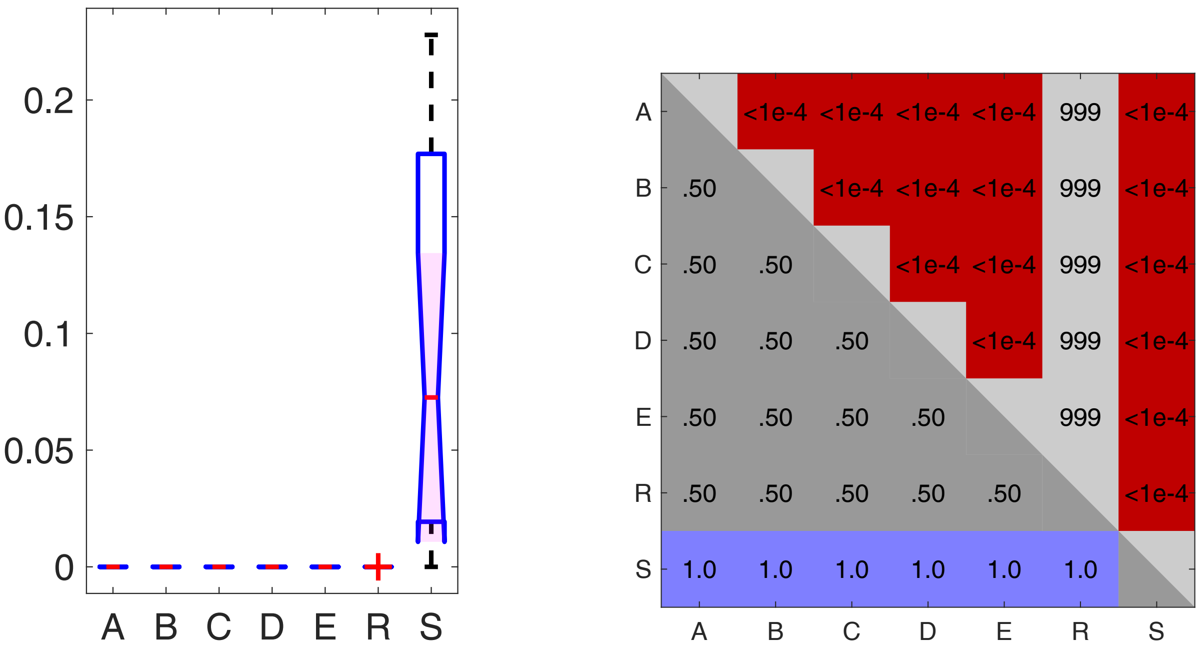

**Heatmap Analysis of Box DG**

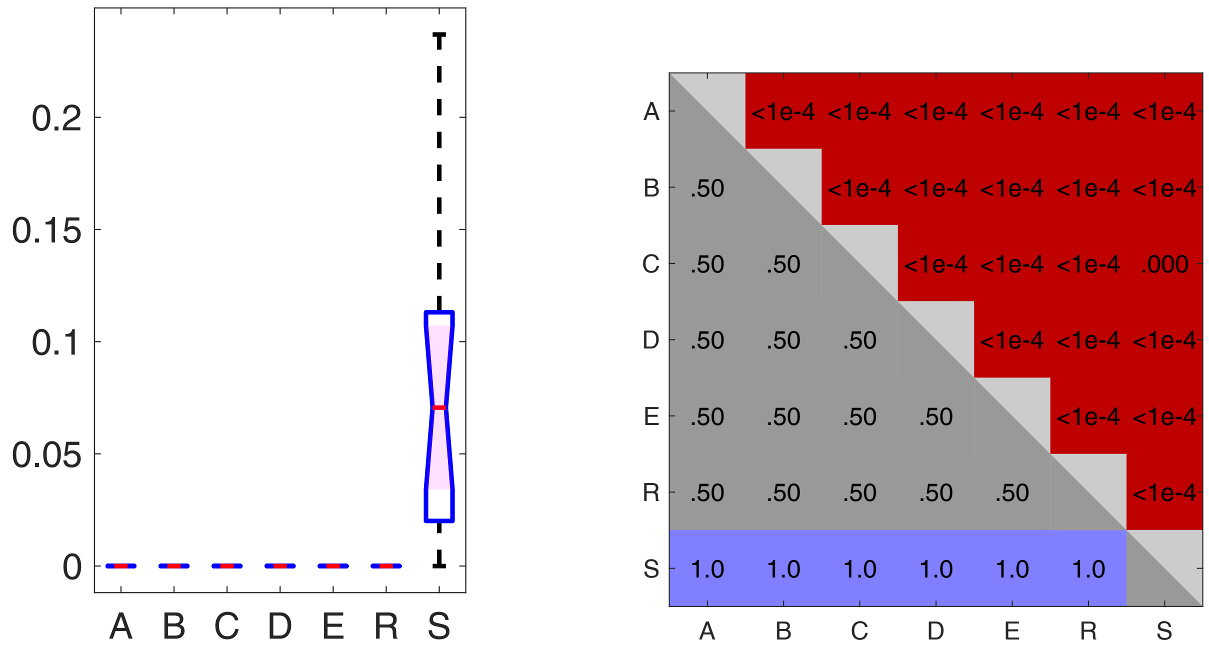


**Heatmap Analysis of Box DH**

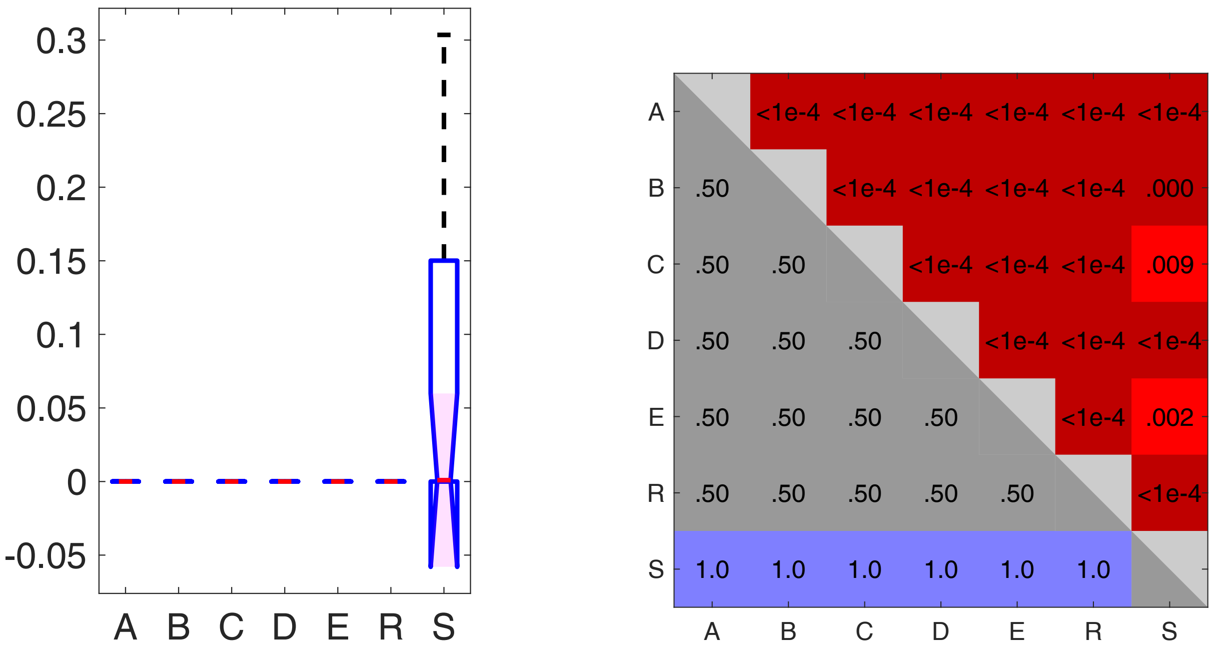

**Heatmap Analysis of Box E0**

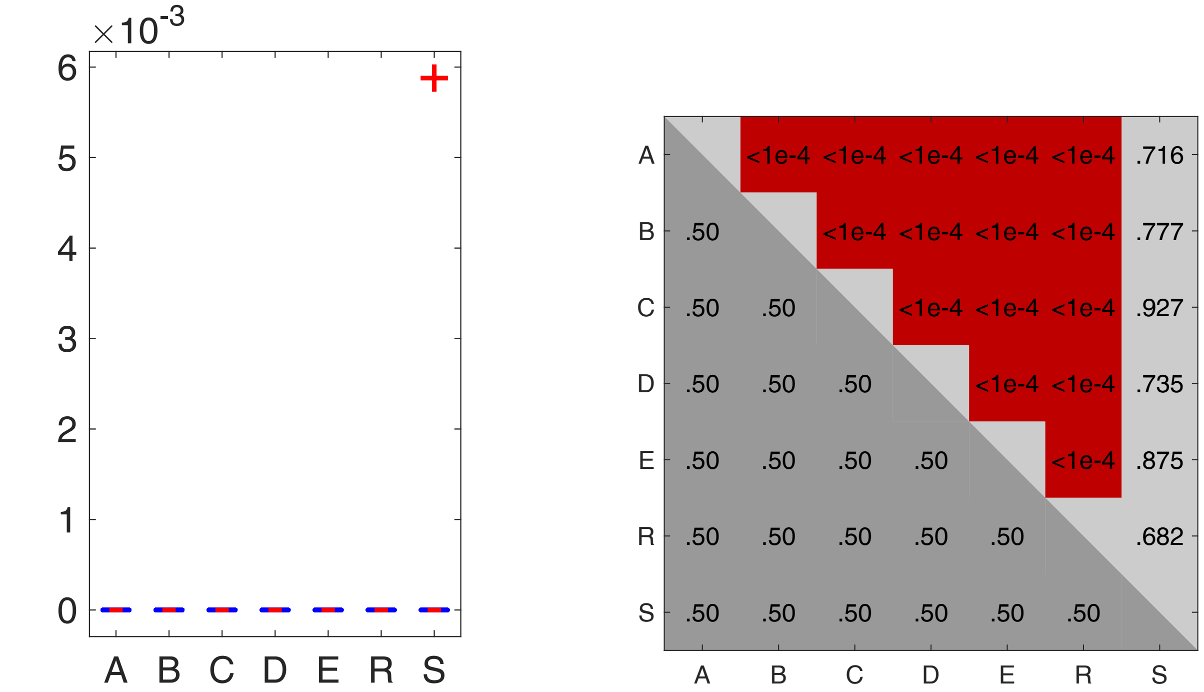


**Heatmap Analysis of Box E1**

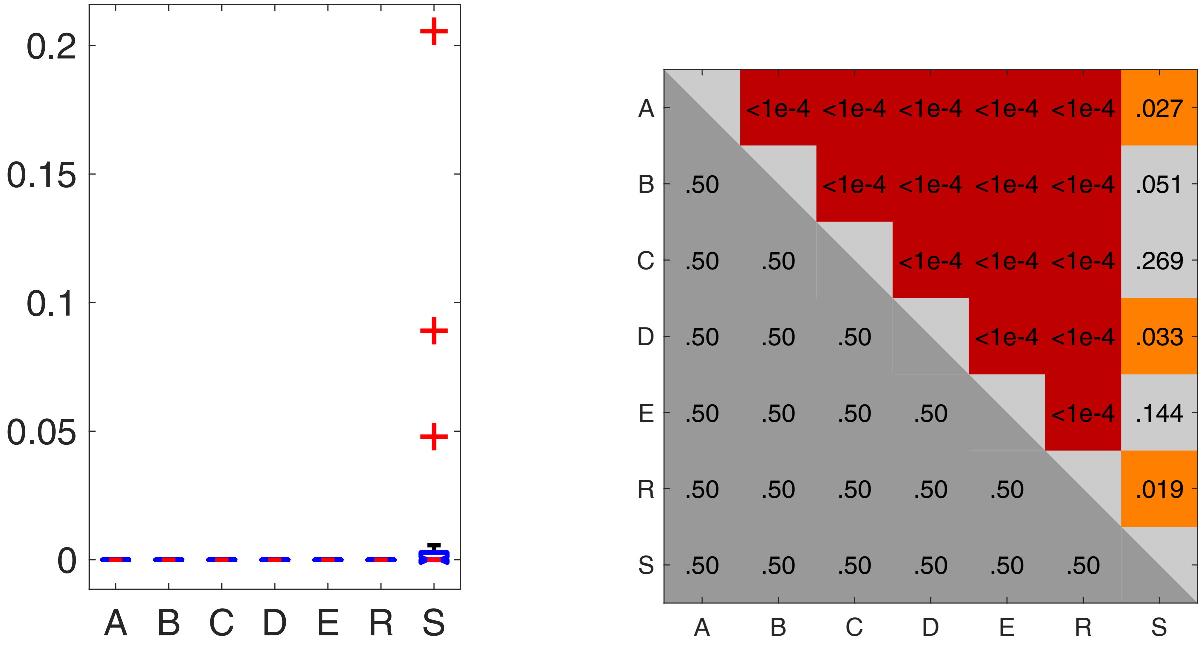

**Heatmap Analysis of Box E2**

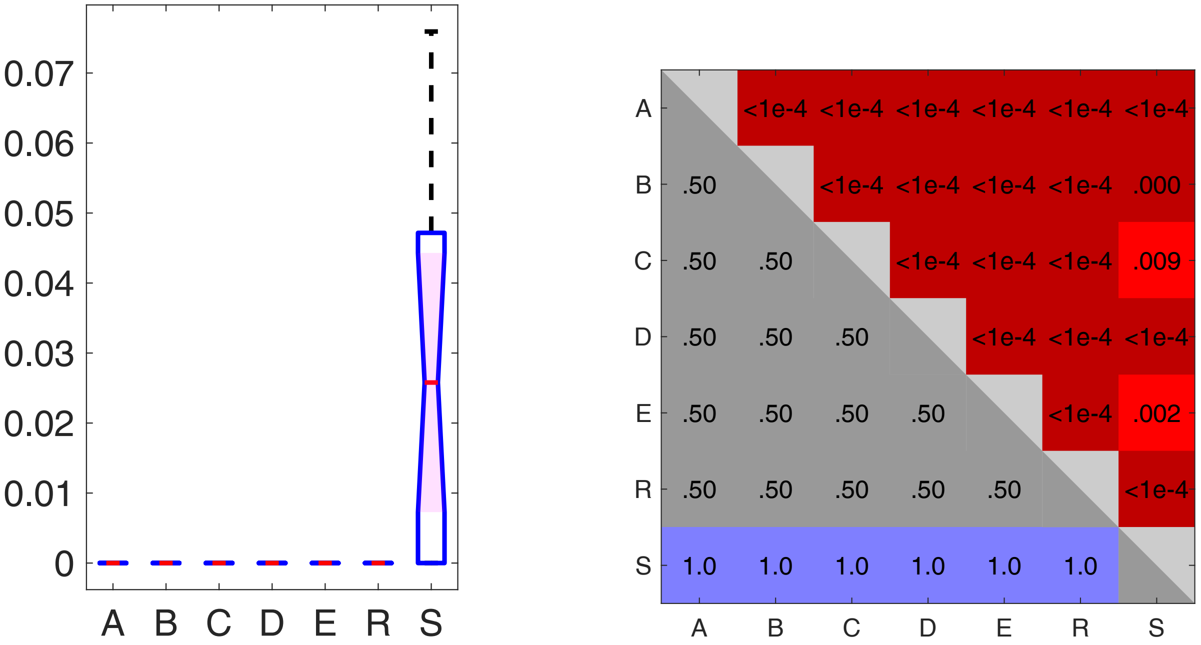


**Heatmap Analysis of Box E3**

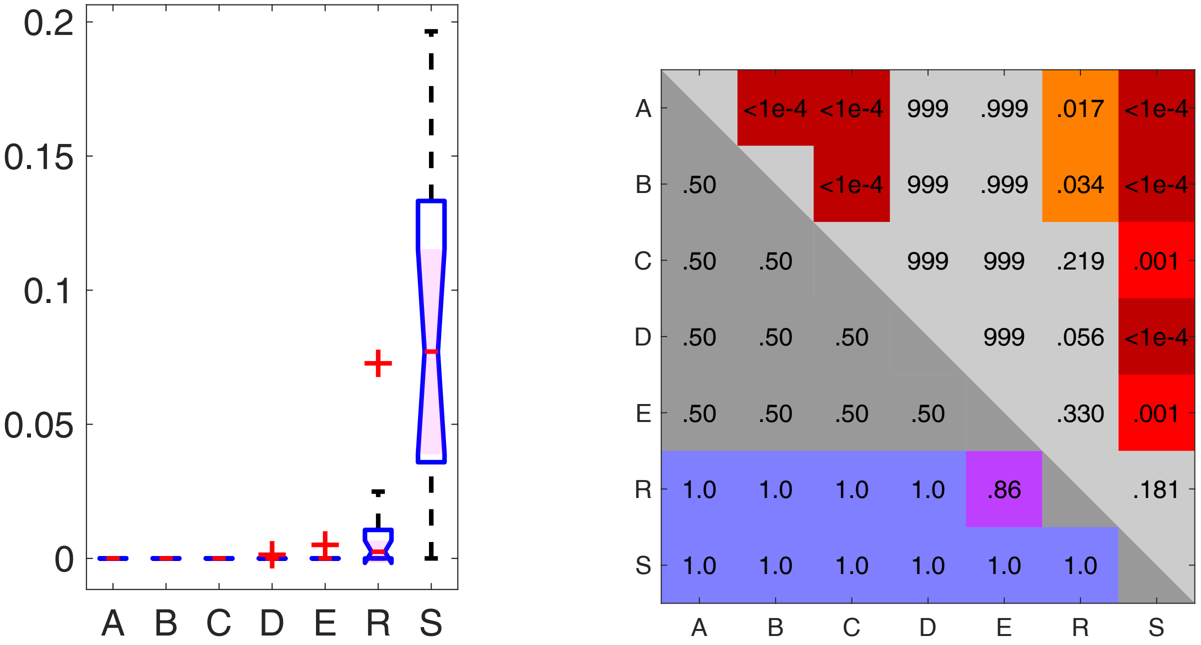

**Heatmap Analysis of Box E4**

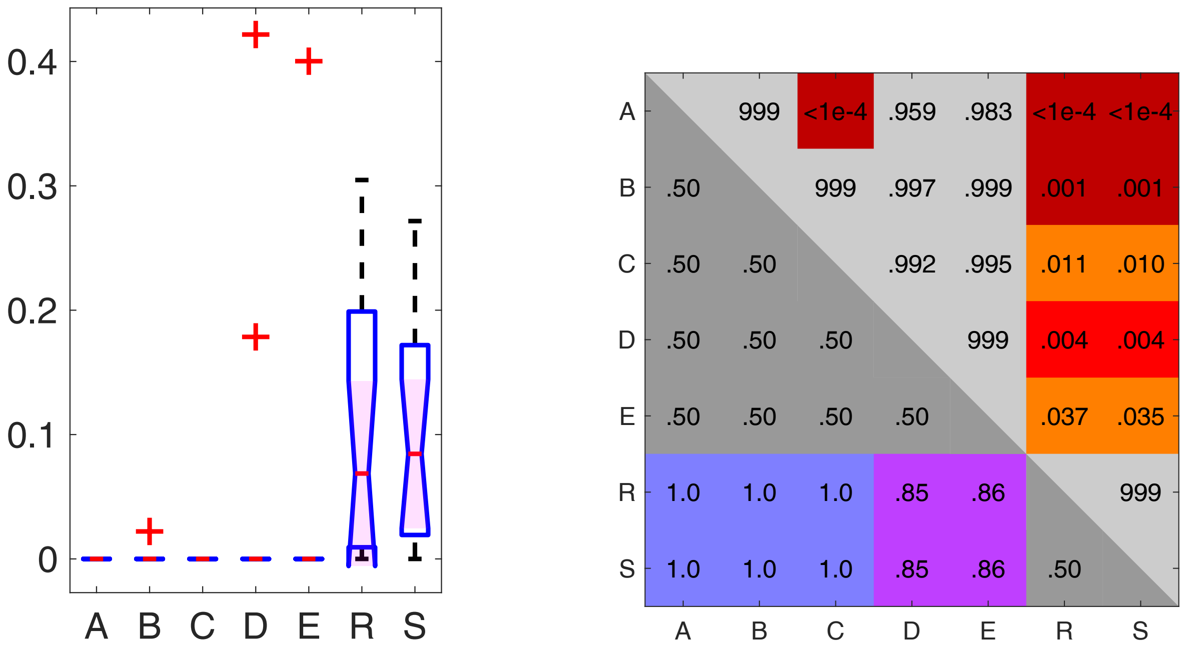


**Heatmap Analysis of Box E5**

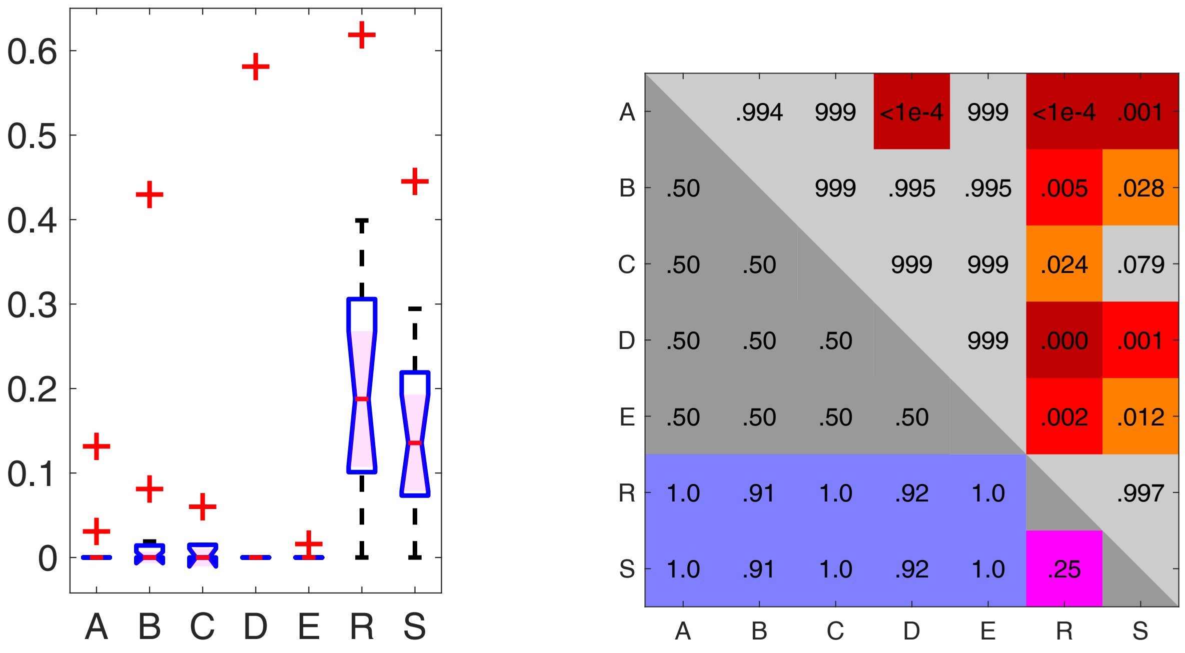

**Heatmap Analysis of Box E6**

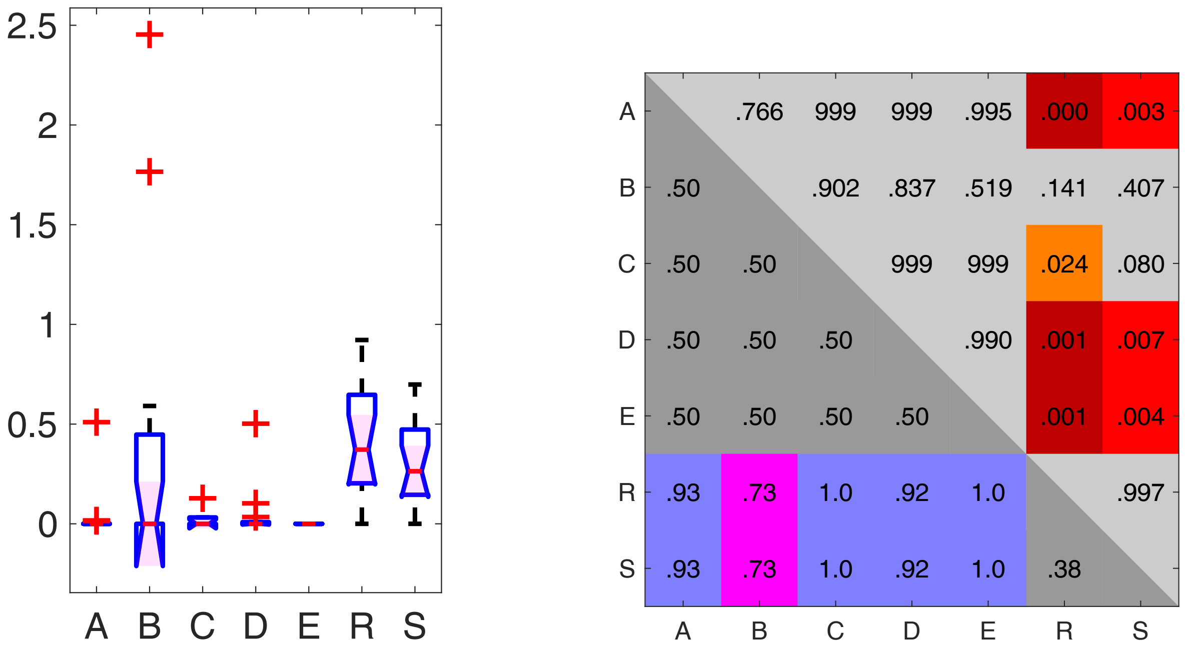


**Heatmap Analysis of Box E7**

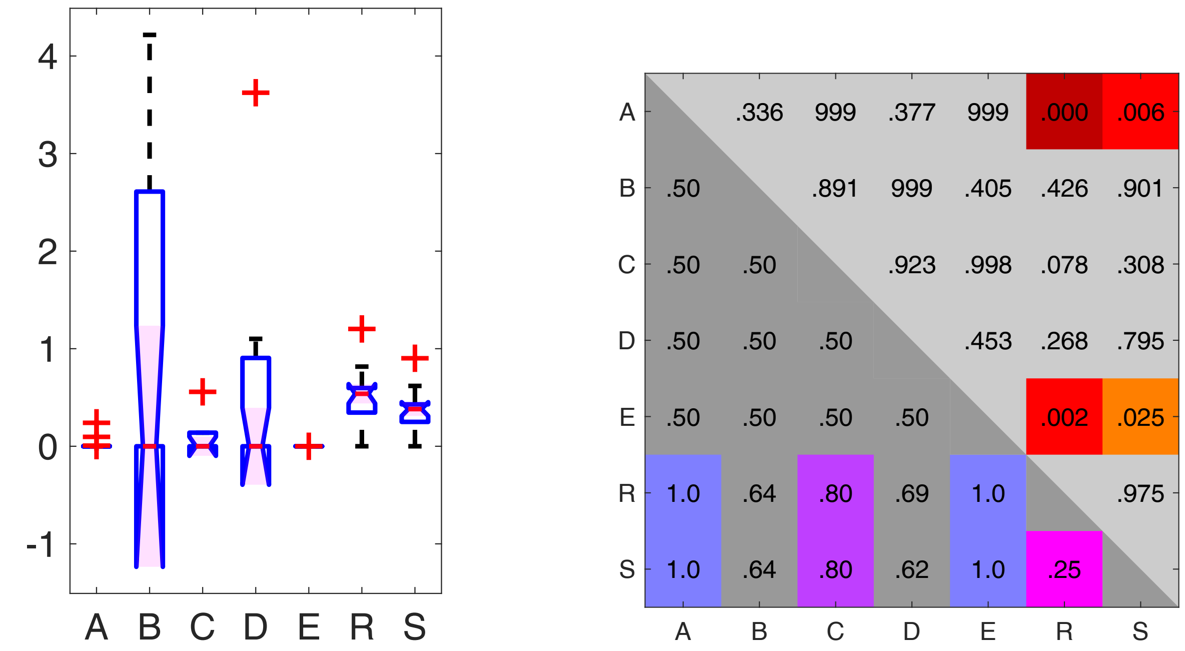

**Heatmap Analysis of Box E8**

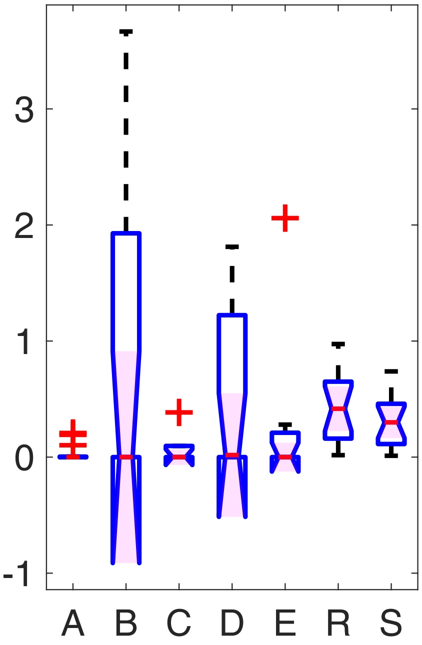


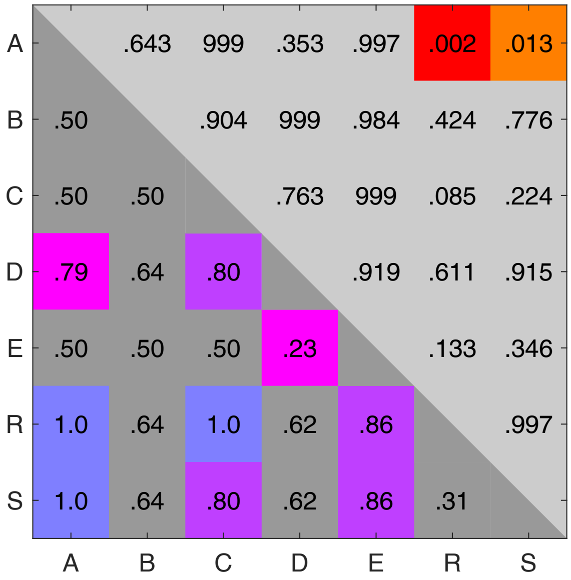


**Heatmap Analysis of Box E9**

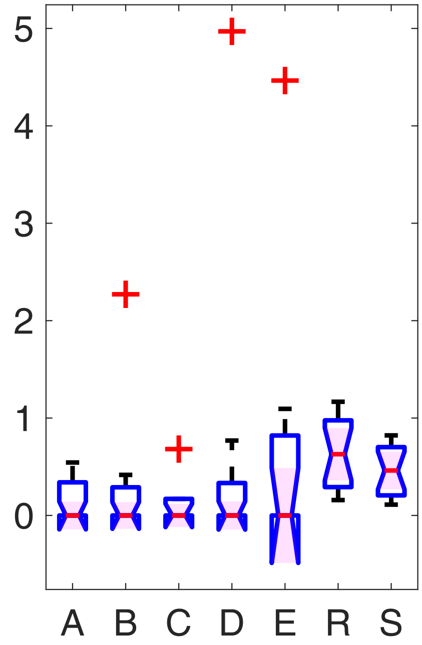


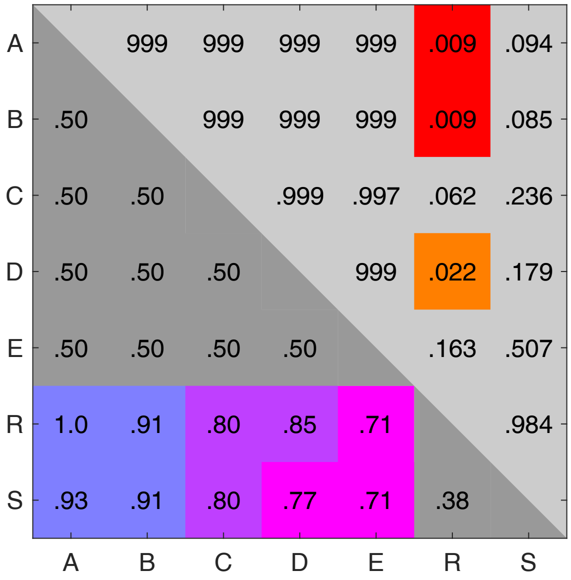

**Heatmap Analysis of Box EA**

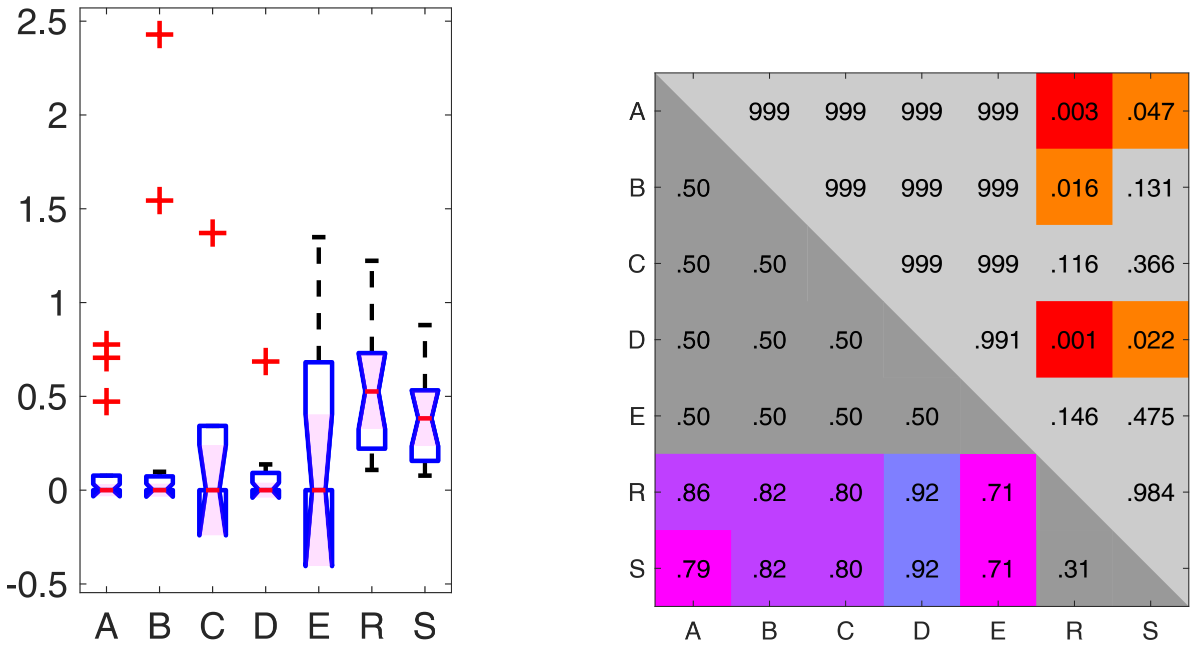


**Heatmap Analysis of Box EB**

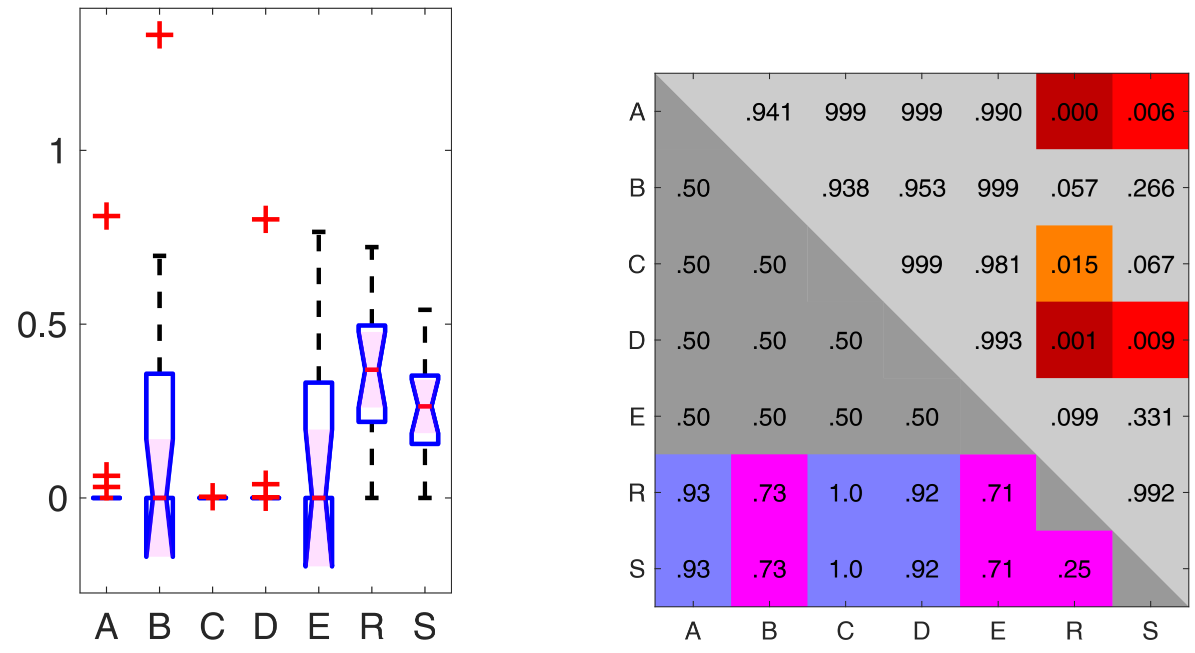

**Heatmap Analysis of Box EC**

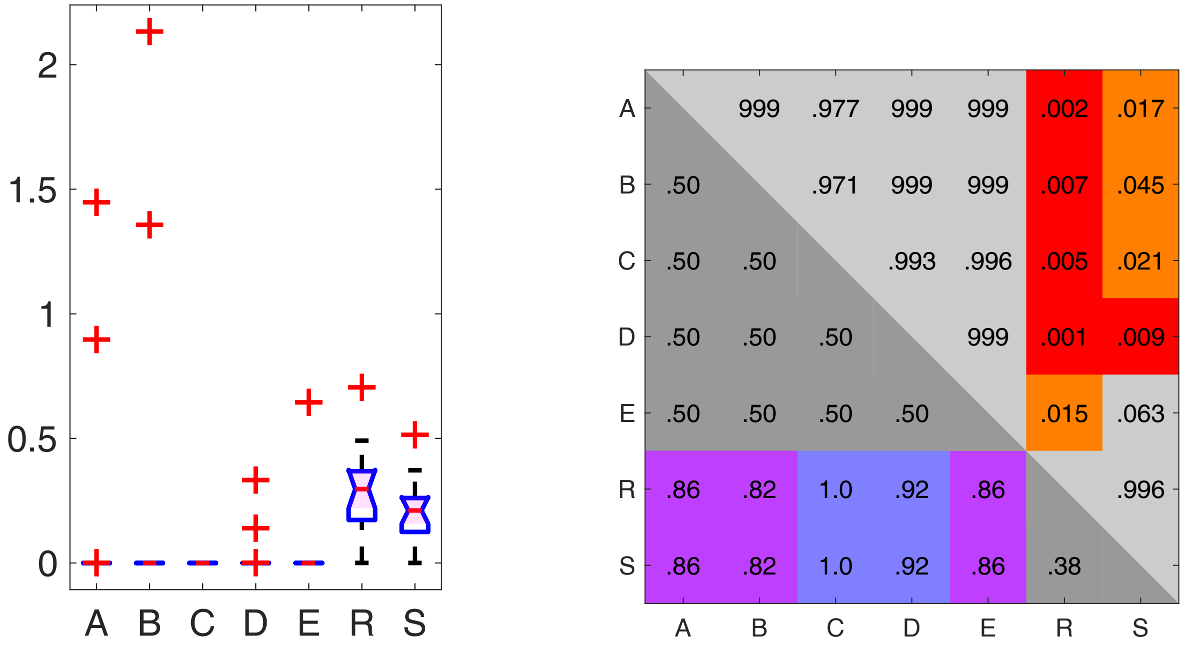


**Heatmap Analysis of Box ED**

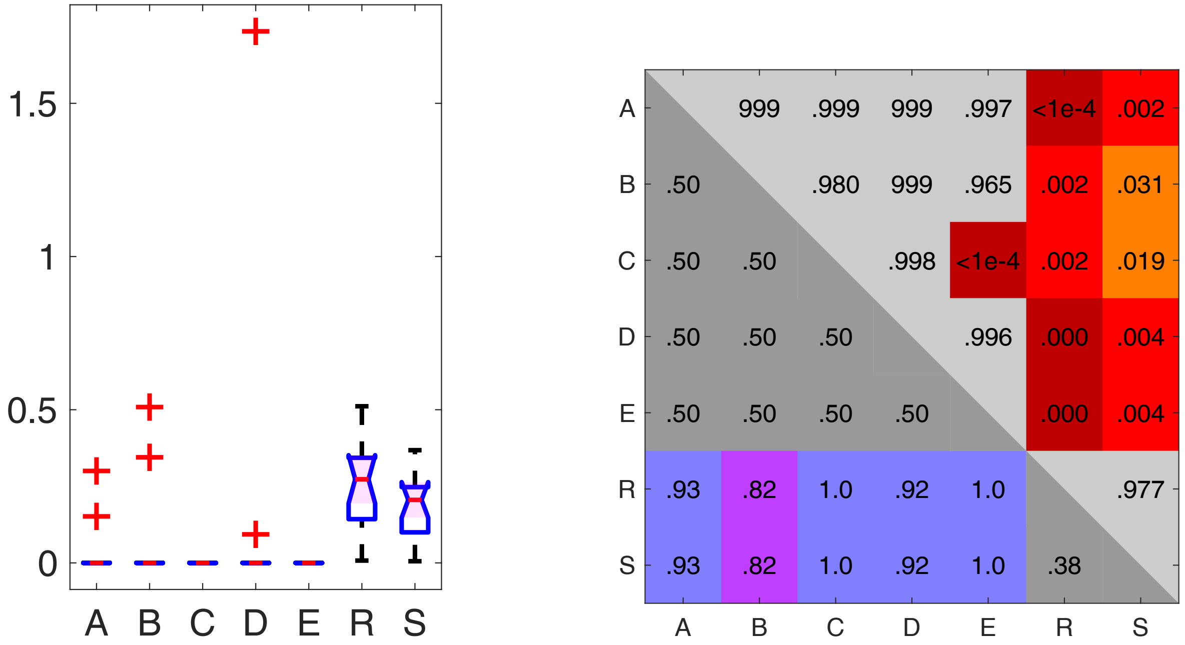

**Heatmap Analysis of Box EE**

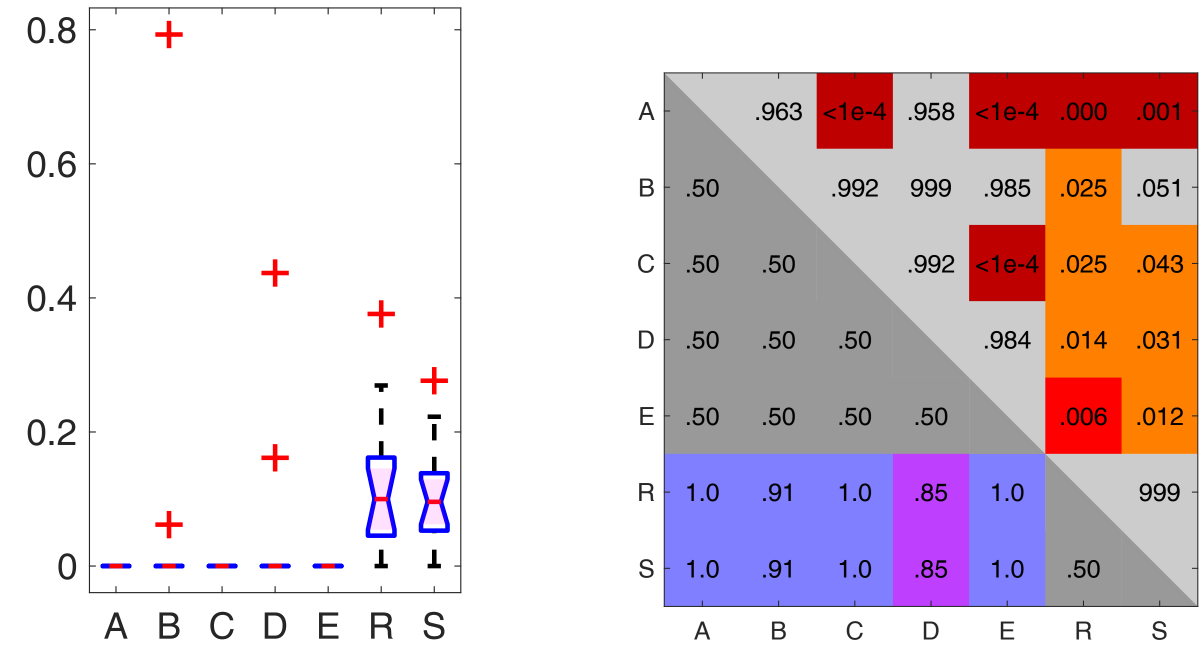


**Heatmap Analysis of Box EF**

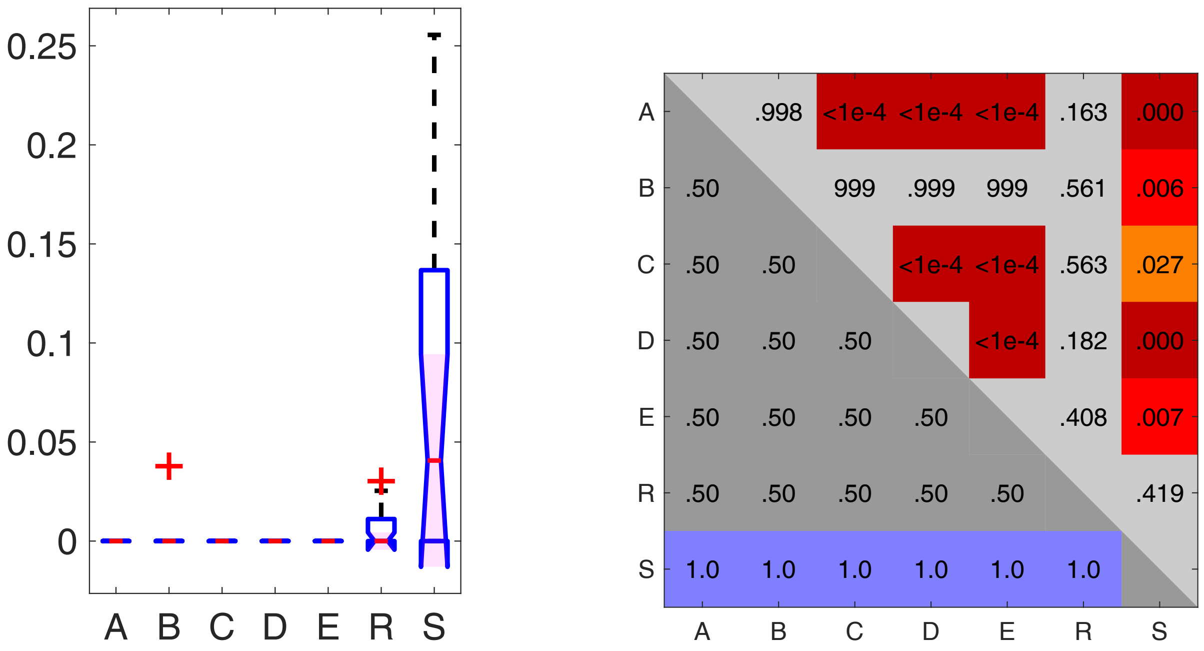

**Heatmap Analysis of Box EG**

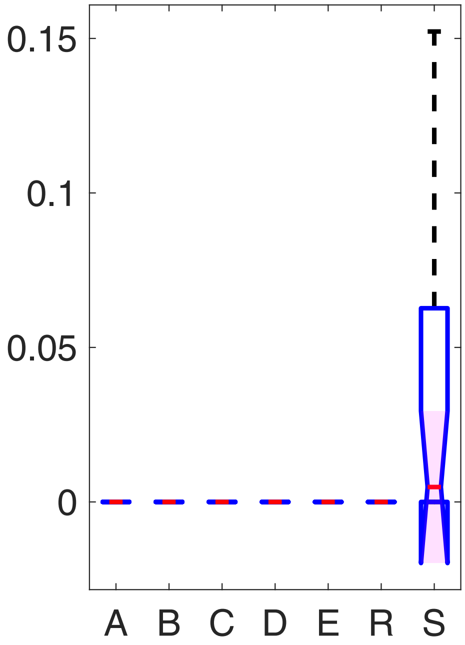


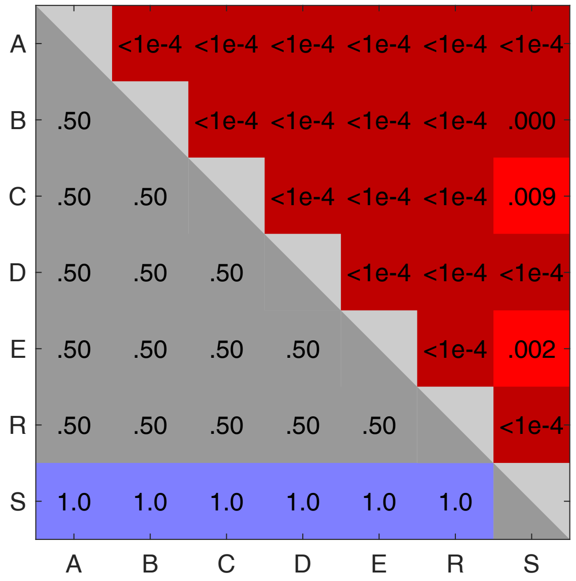


**Heatmap Analysis of Box EH**

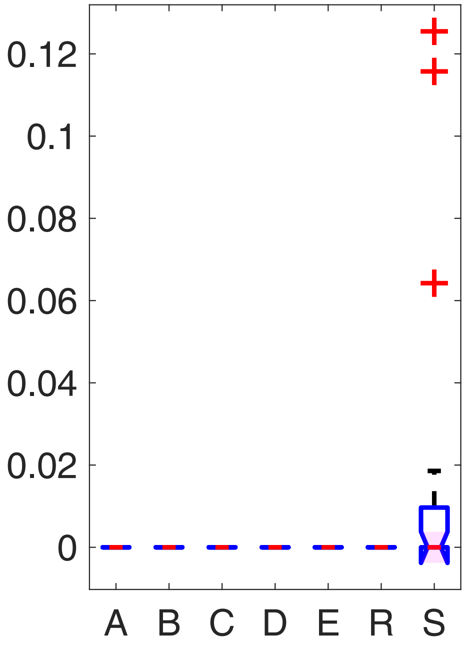


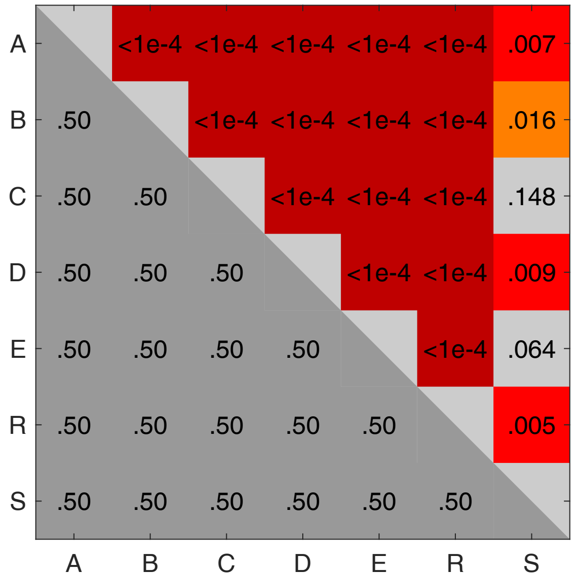

**Heatmap Analysis of Box F0**

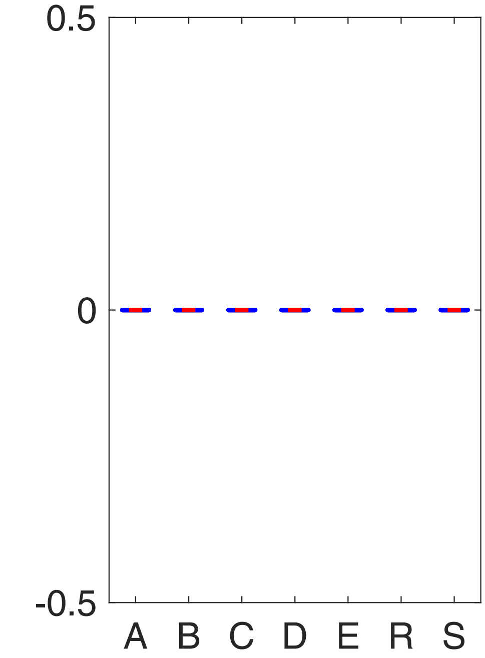


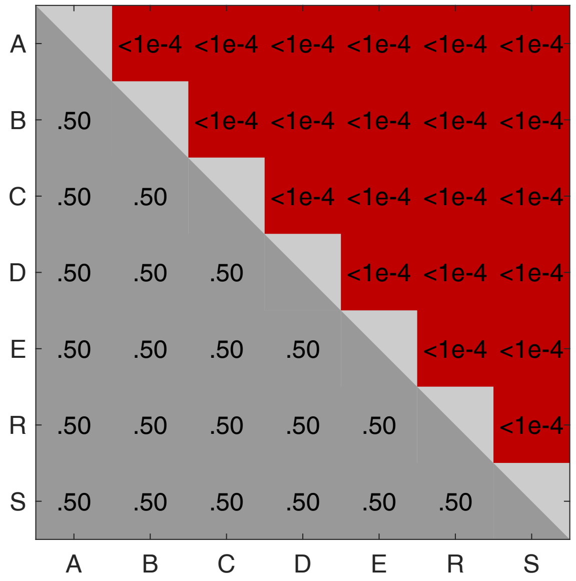


**Heatmap Analysis of Box F1**

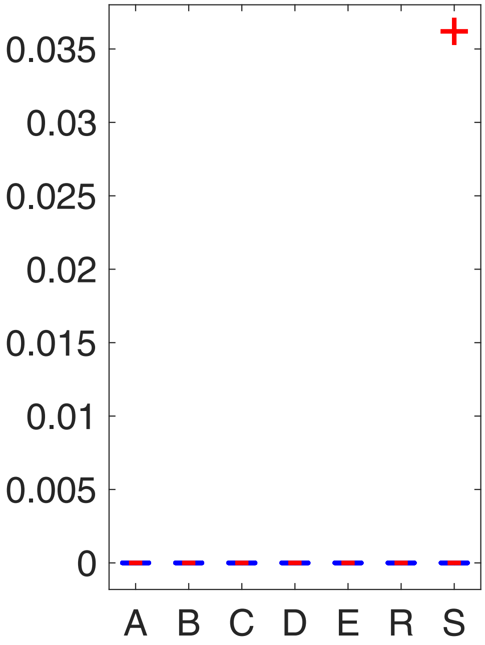


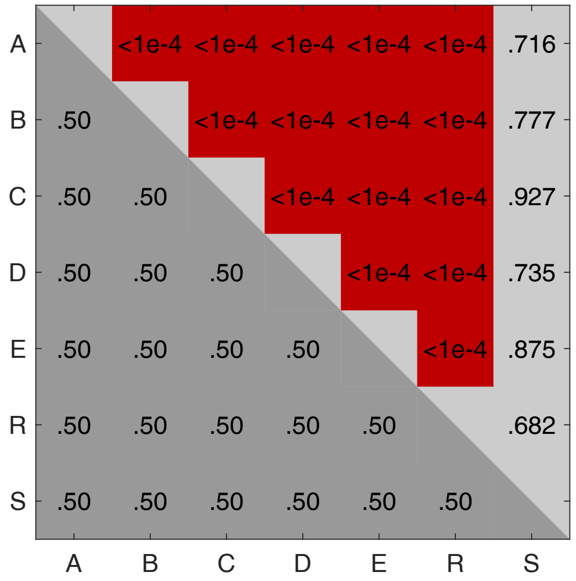

**Heatmap Analysis of Box F2**

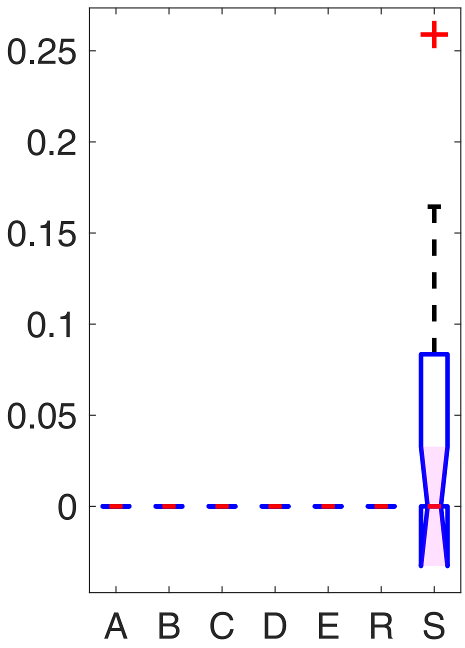


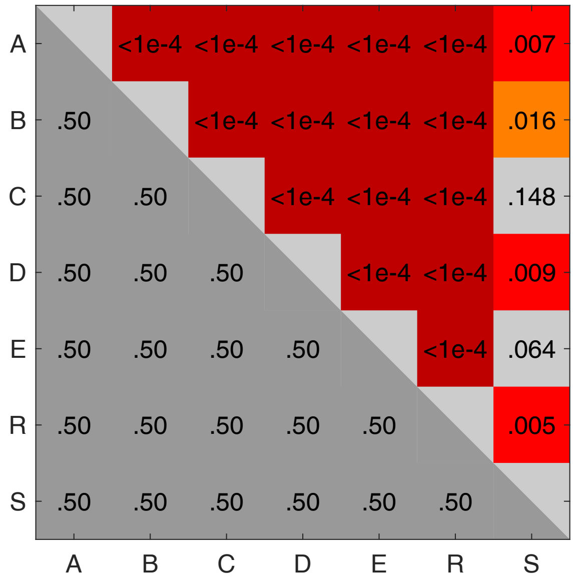


**Heatmap Analysis of Box F3**

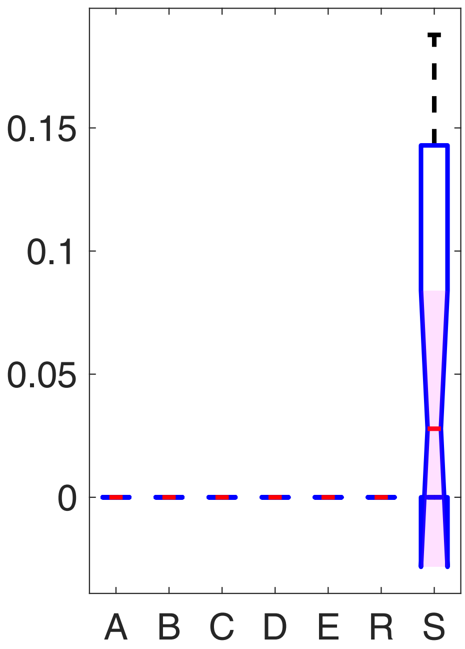


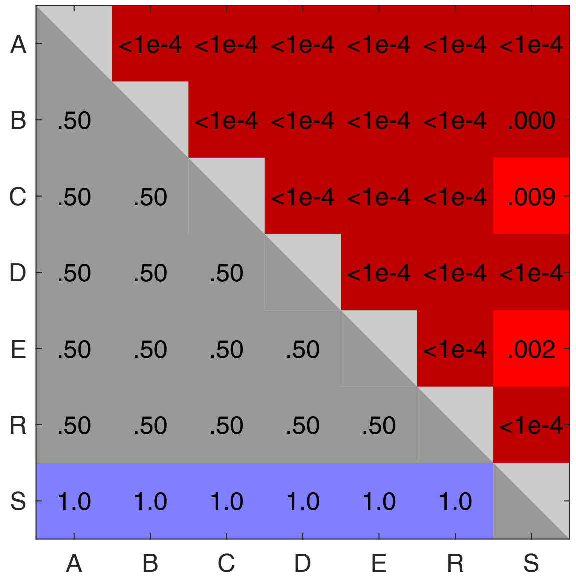

**Heatmap Analysis of Box F4**

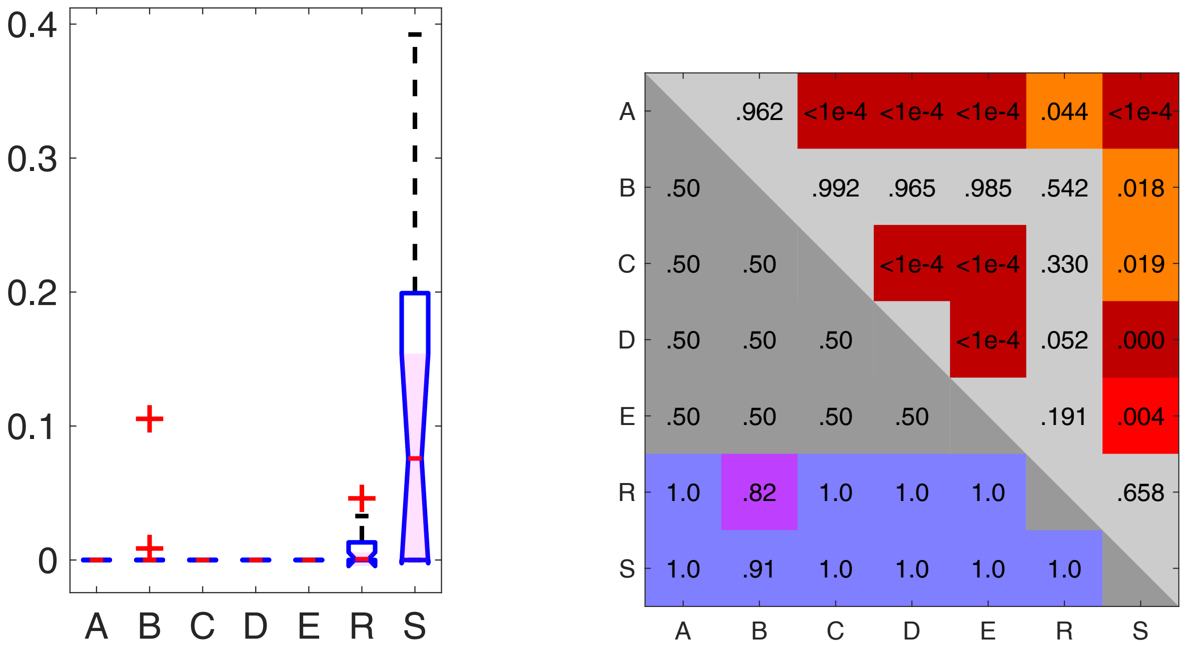


**Heatmap Analysis of Box F5**

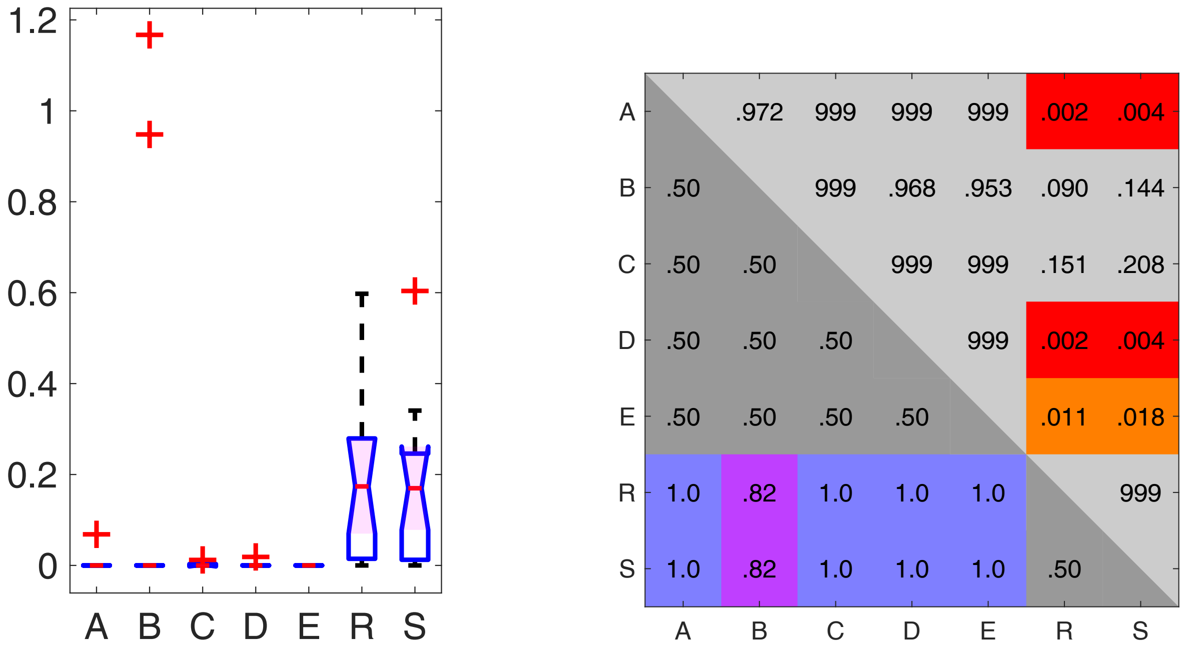

**Heatmap Analysis of Box F6**

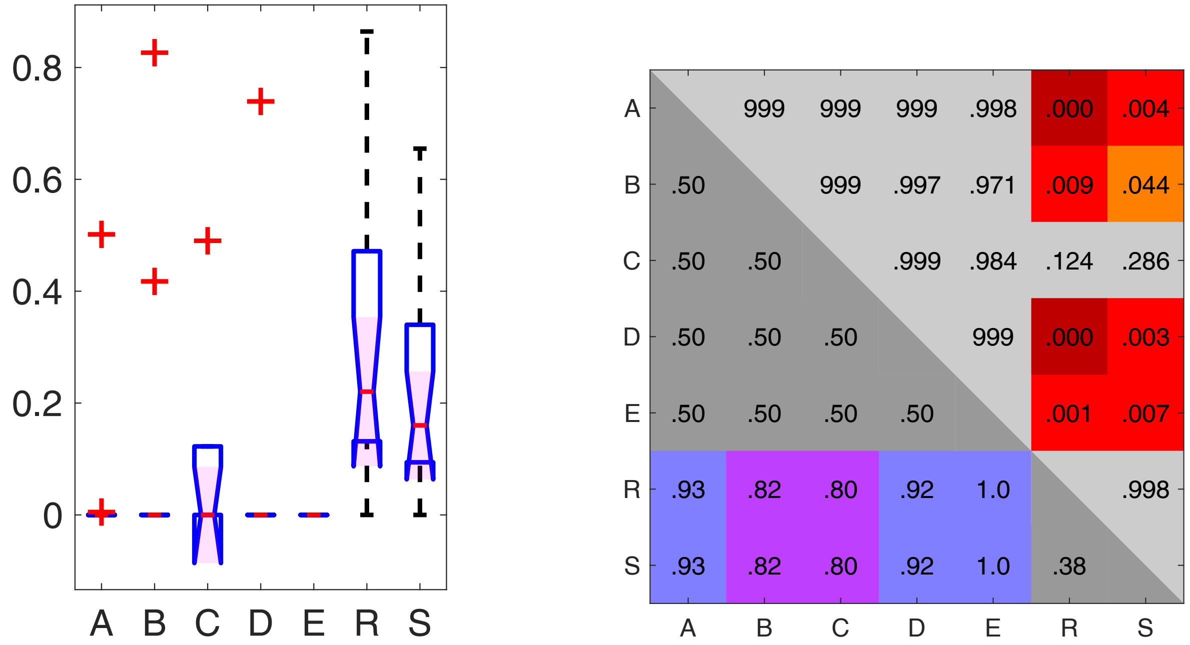


**Heatmap Analysis of Box F7**

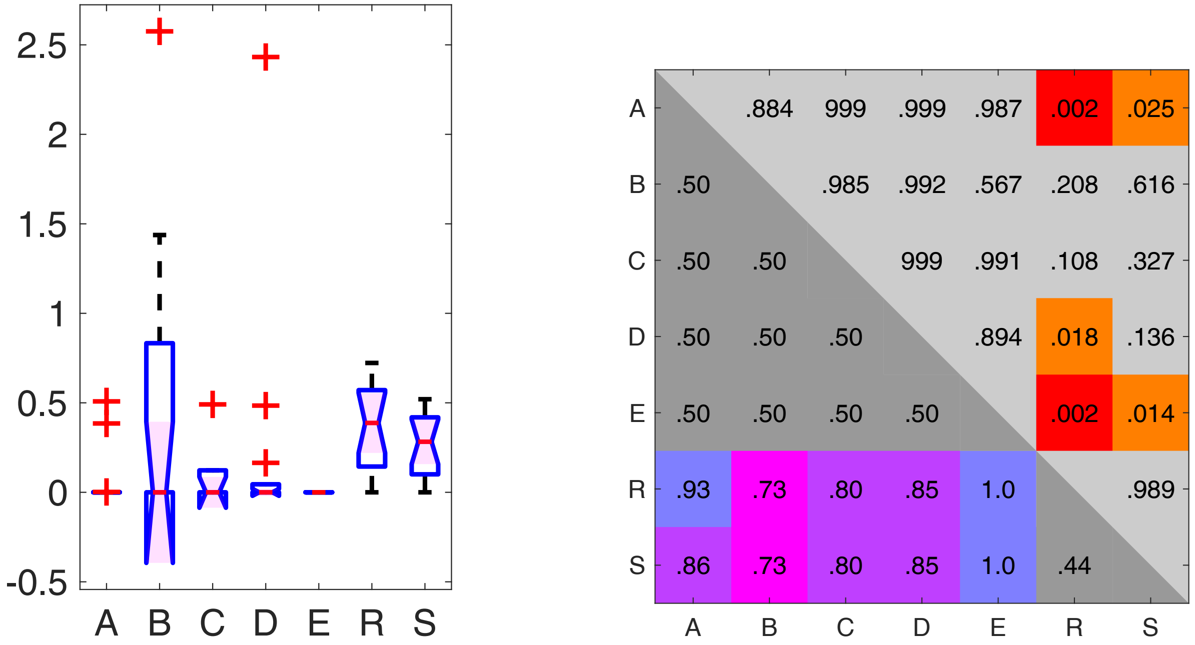

**Heatmap Analysis of Box F8**

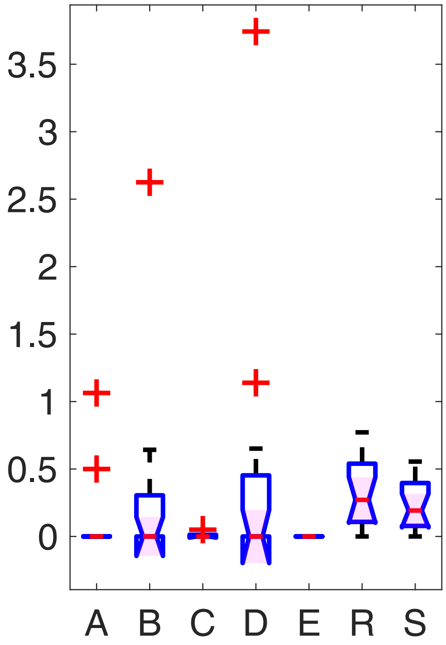


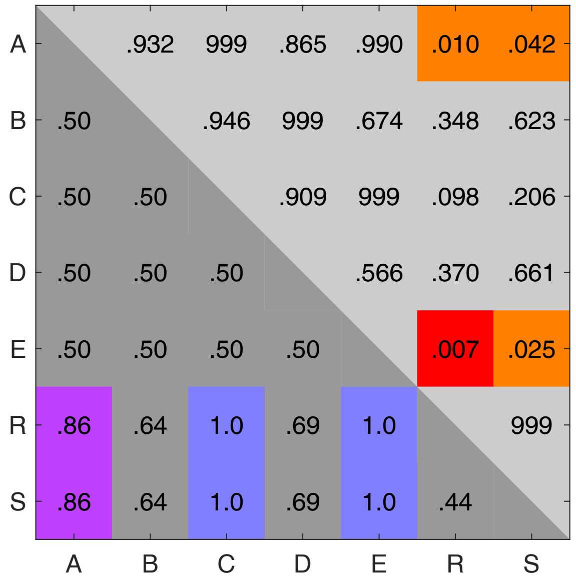


**Heatmap Analysis of Box F9**

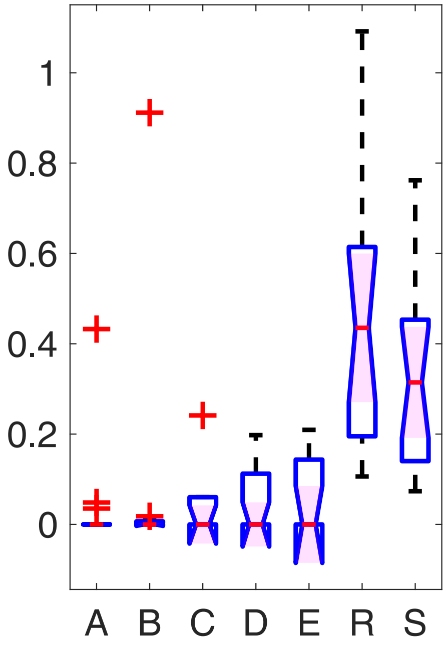


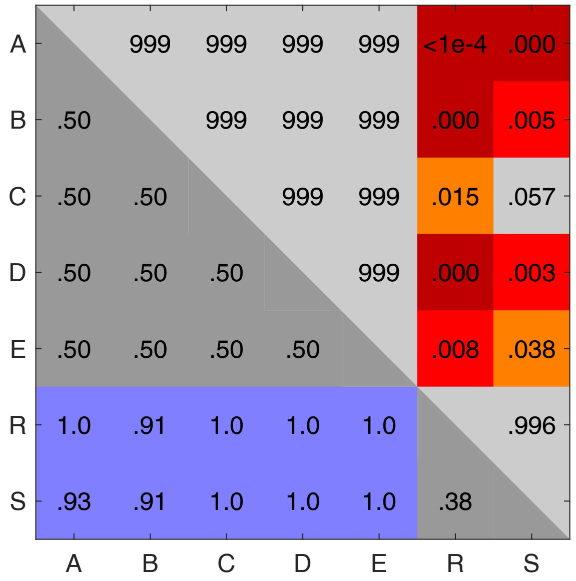

**Heatmap Analysis of Box FA**

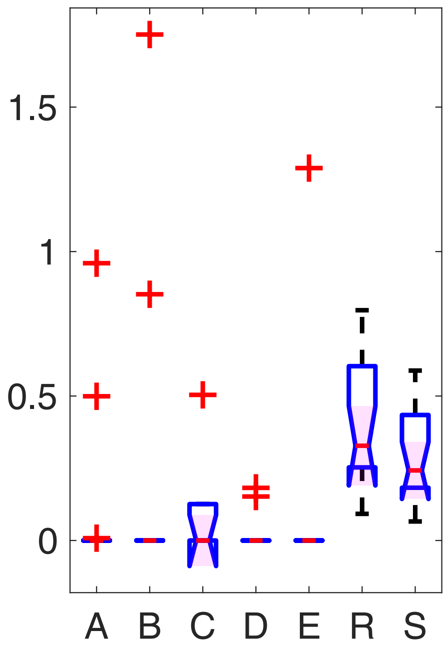


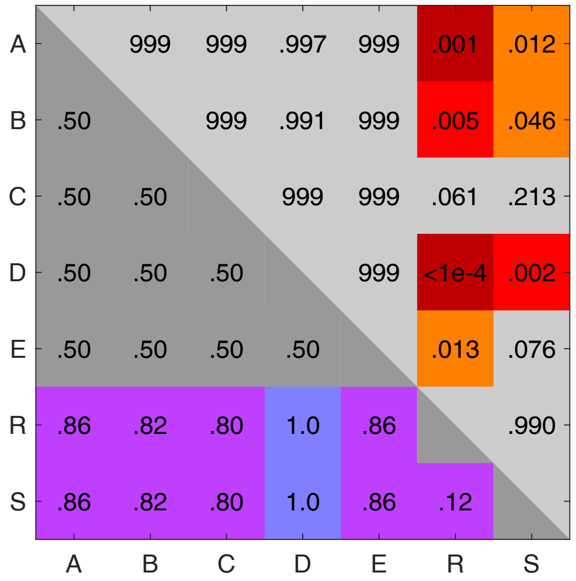


**Heatmap Analysis of Box FB**

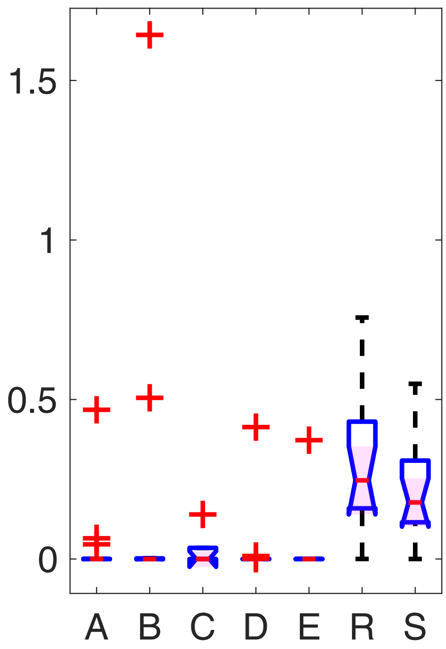


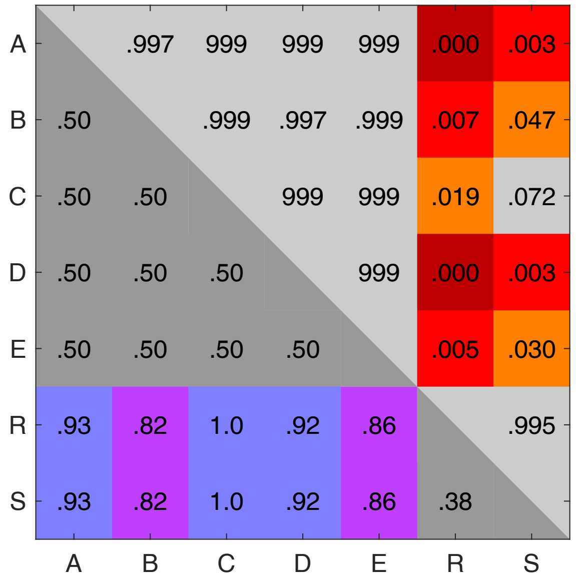

**Heatmap Analysis of Box FC**

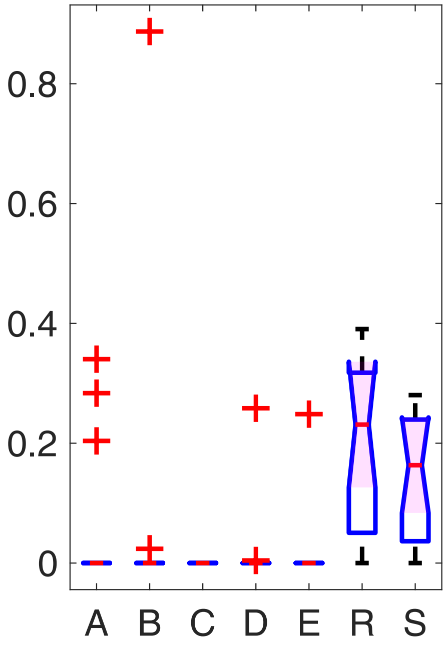


| | A | B | C | D | E | R | S |
|---|---|---|---|---|---|---|---|
| A | | 999 | .973 | .997 | 999 | .006 | .066 |
| B | .50 | | .995 | 999 | 999 | .005 | .049 |
| C | .50 | .50 | | 999 | .999 | .009 | .050 |
| D | .50 | .50 | .50 | | 999 | .001 | .013 |
| E | .50 | .50 | .50 | .50 | | .018 | .103 |
| R | .86 | .91 | 1.0 | .92 | .86 | | .989 |
| S | .79 | .91 | 1.0 | .92 | .86 | .44 | |

**Heatmap Analysis of Box FD**

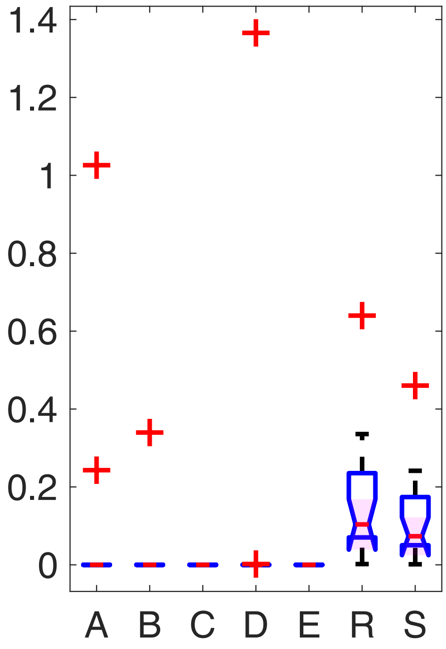


| | A | B | C | D | E | R | S |
|---|---|---|---|---|---|---|---|
| A | | 999 | .993 | 999 | .988 | .000 | .004 |
| B | .50 | | 999 | 999 | 999 | .000 | .003 |
| C | .50 | .50 | | .998 | <1e-4 | .003 | .014 |
| D | .50 | .50 | .50 | | .996 | .000 | .003 |
| E | .50 | .50 | .50 | .50 | | .000 | .003 |
| R | .86 | .91 | 1.0 | .92 | 1.0 | | .997 |
| S | .86 | .91 | 1.0 | .92 | 1.0 | .31 | |

**Heatmap Analysis of Box FE**

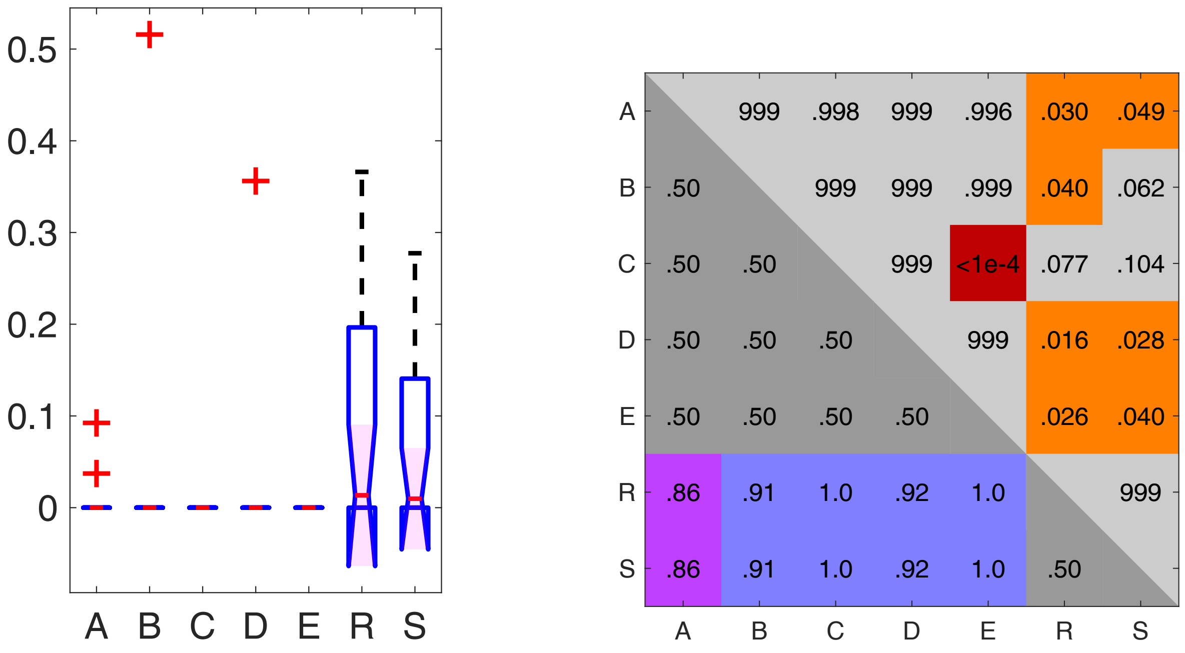


**Heatmap Analysis of Box FF**

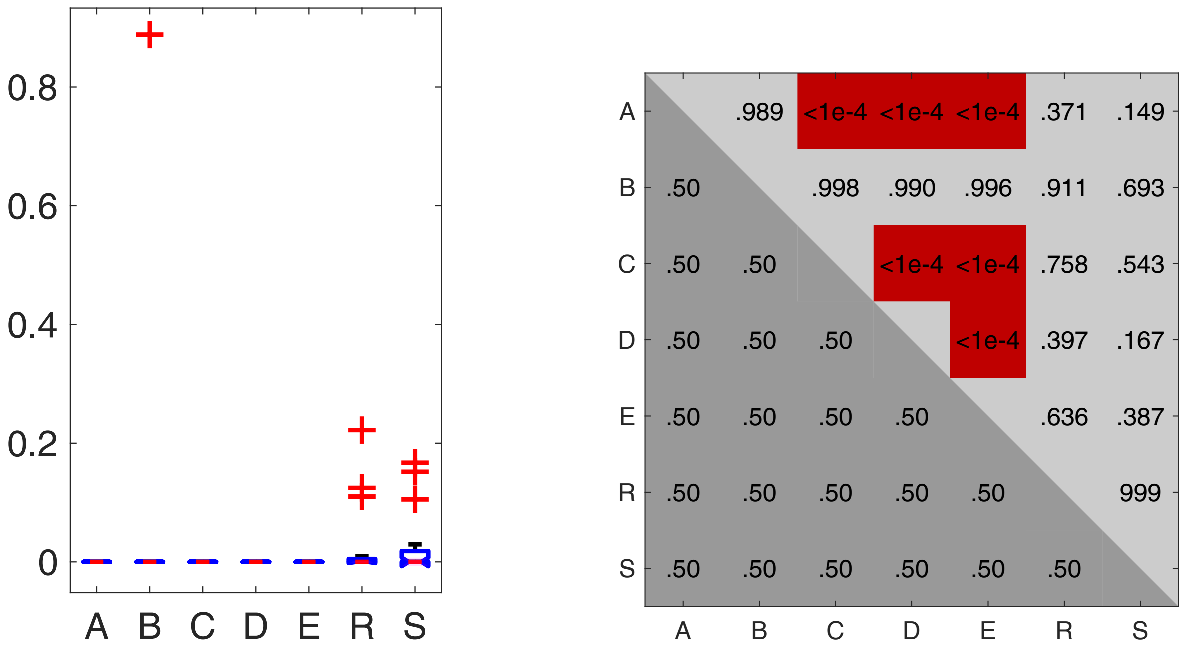

**Heatmap Analysis of Box FG**

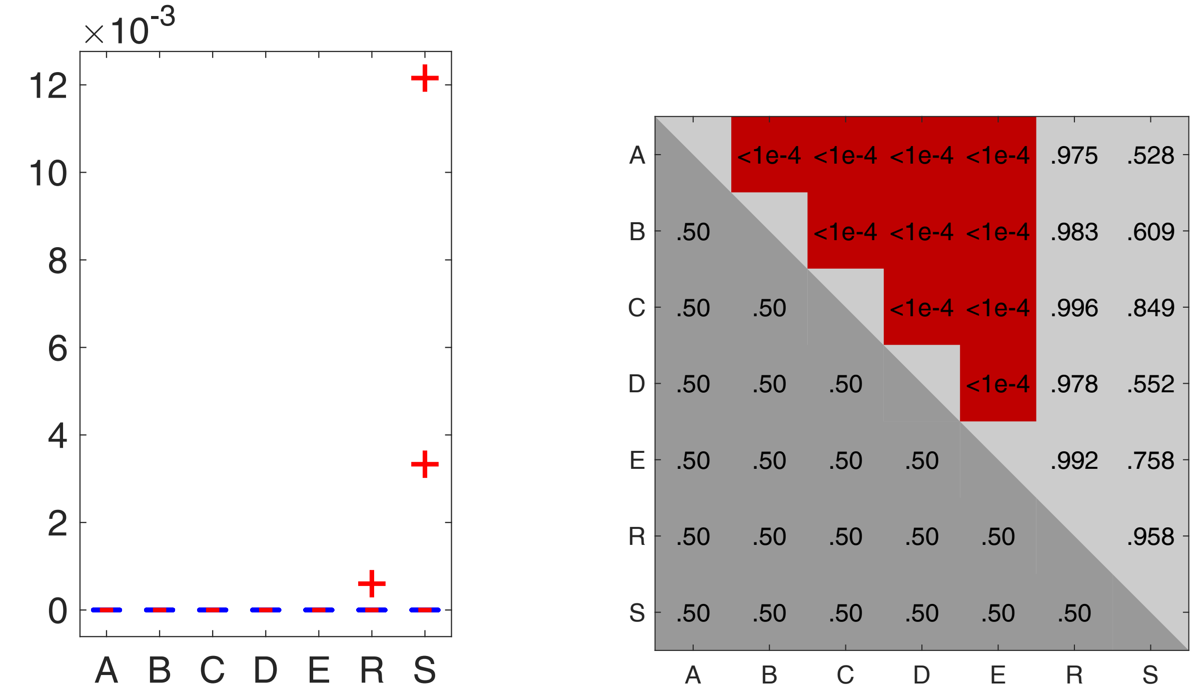


**Heatmap Analysis of Box FH**

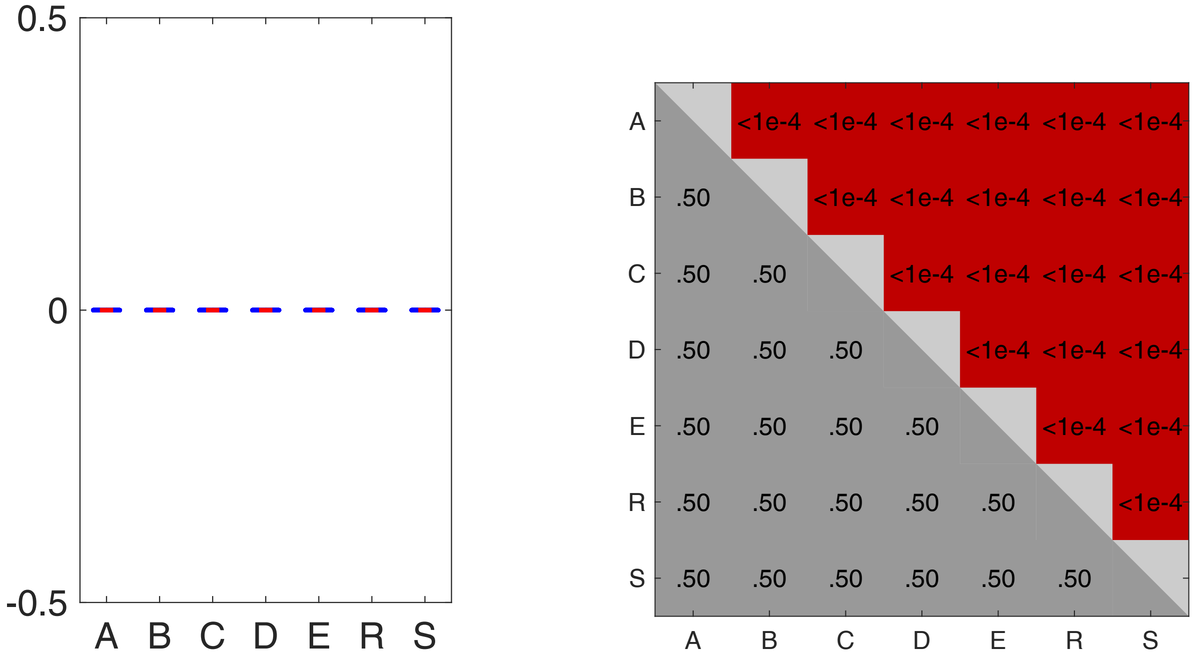

**Heatmap Analysis of Box G0**

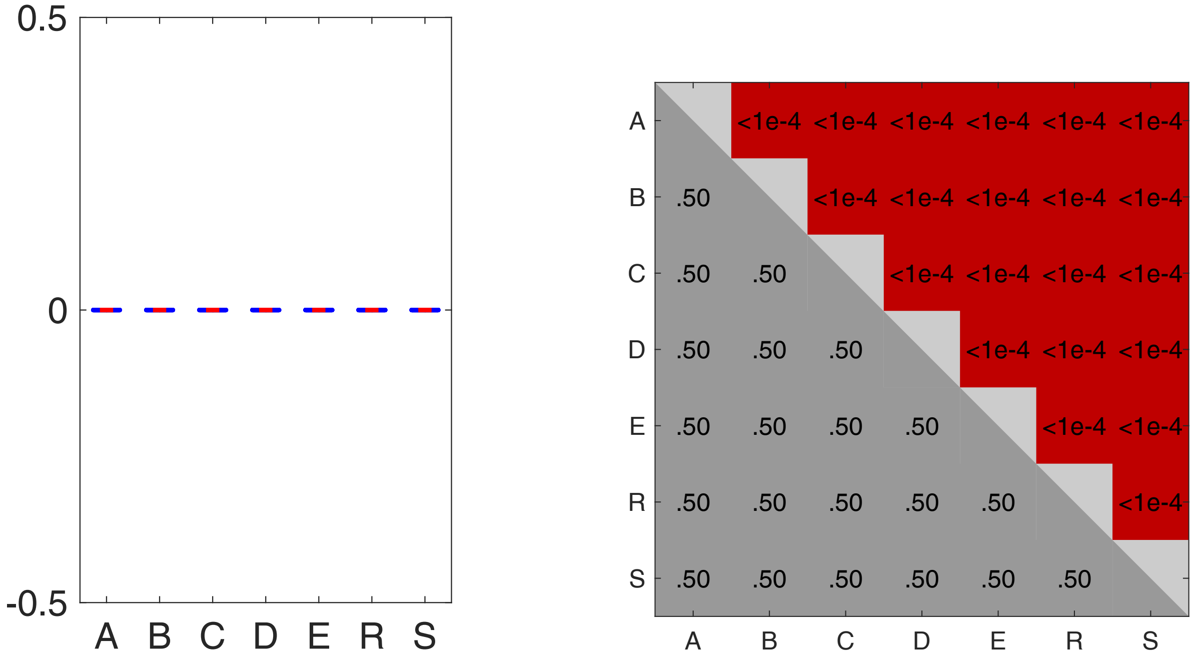


**Heatmap Analysis of Box G1**

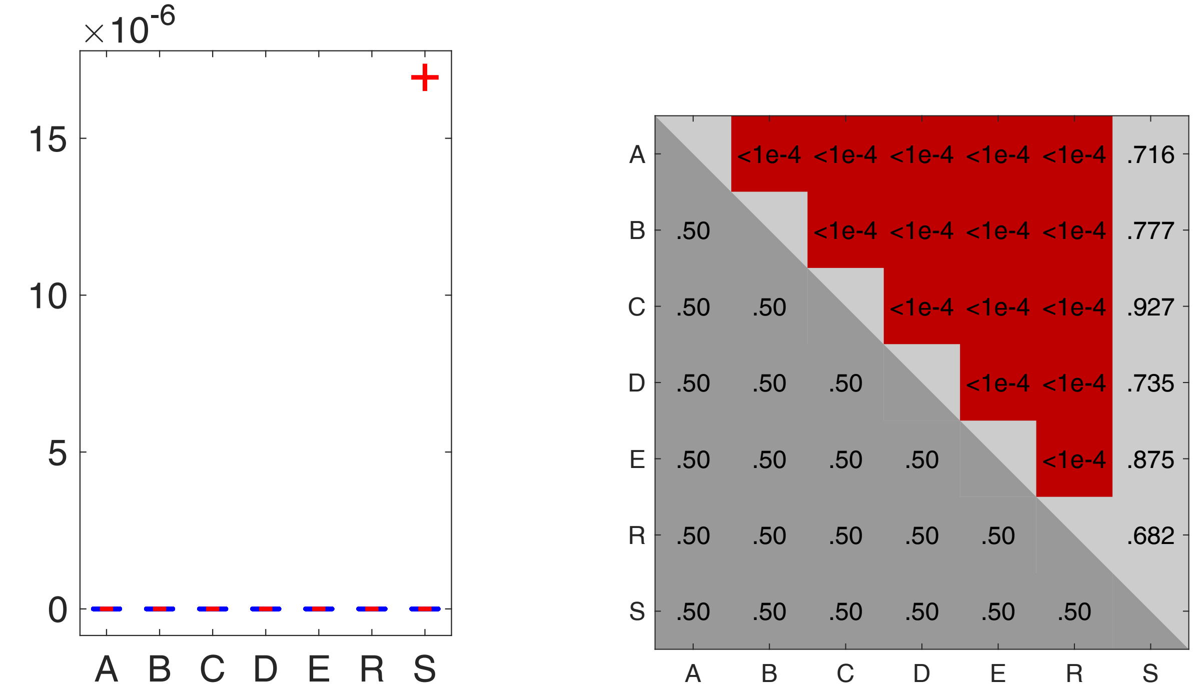

**Heatmap Analysis of Box G2**

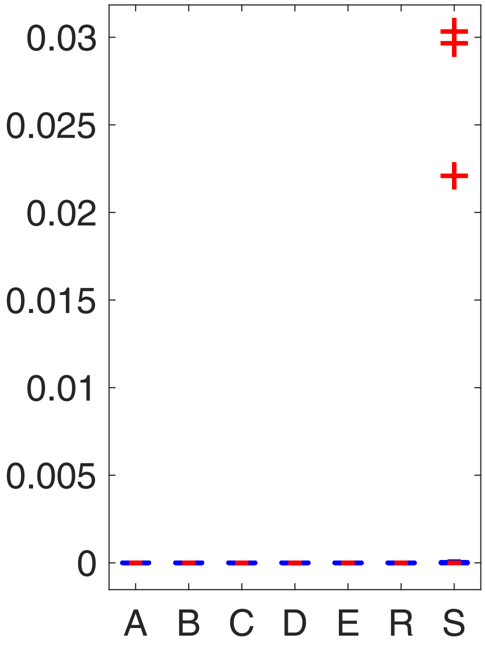


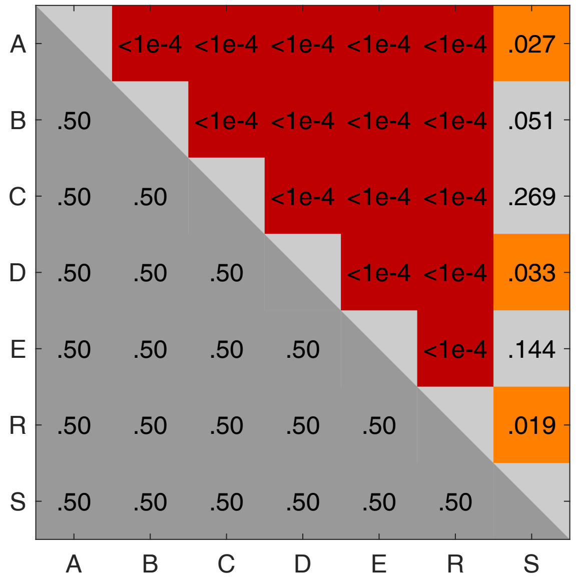


**Heatmap Analysis of Box G3**

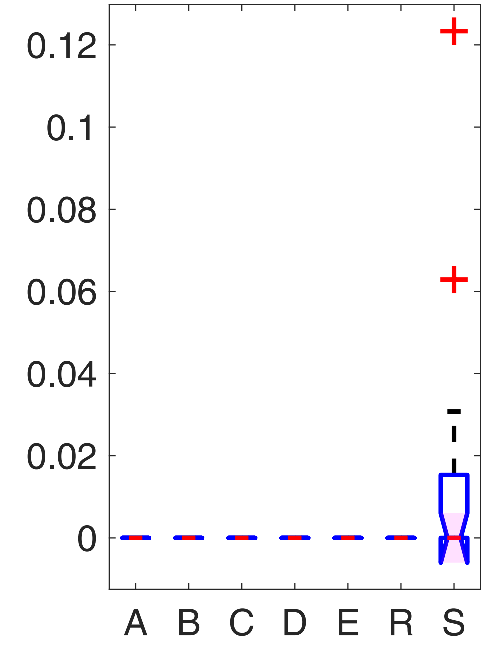


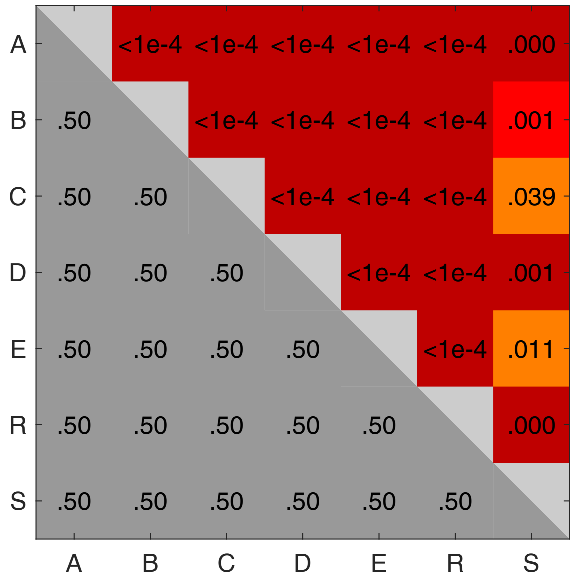

**Heatmap Analysis of Box G4**

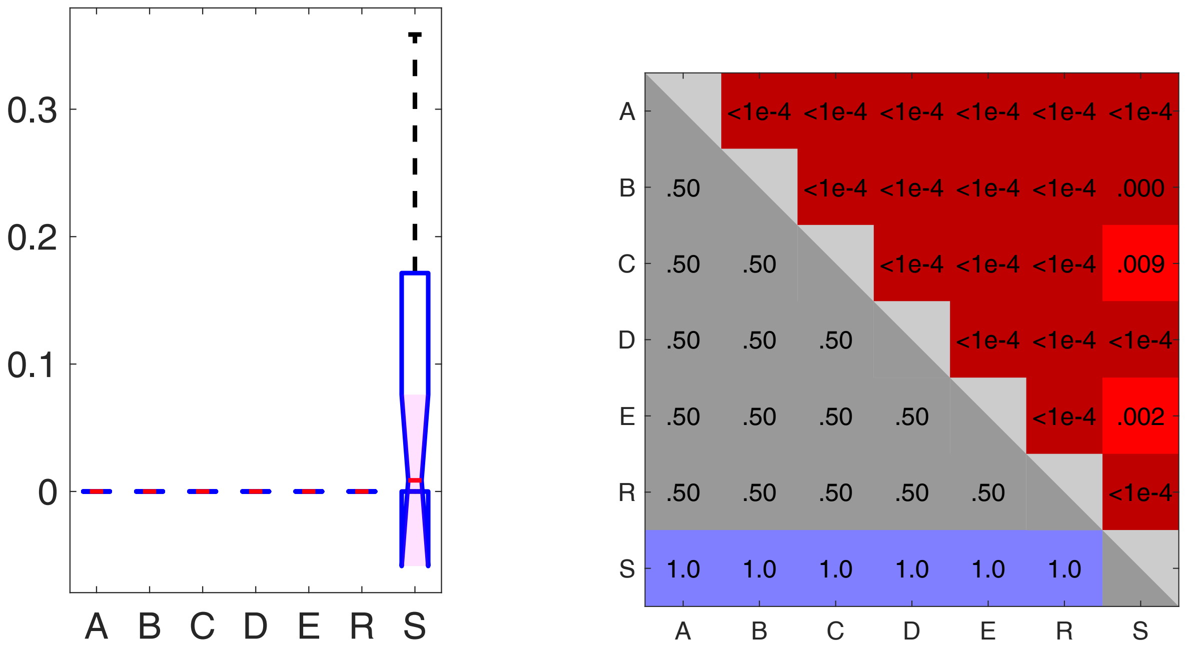


**Heatmap Analysis of Box G5**

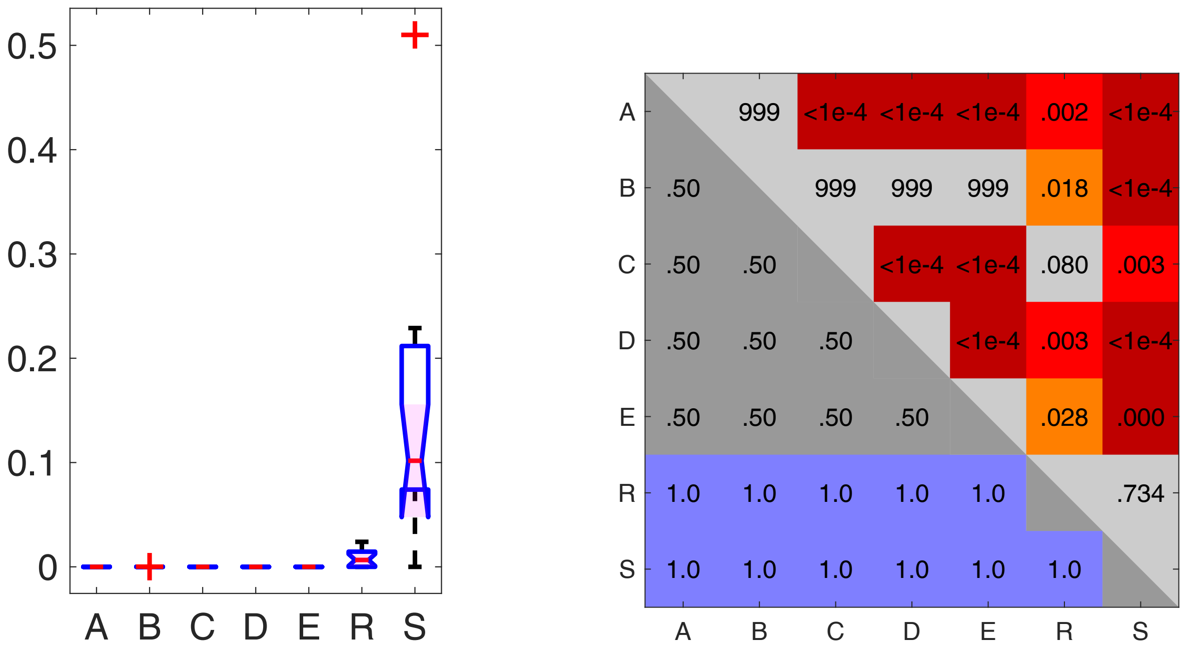

**Heatmap Analysis of Box G6**

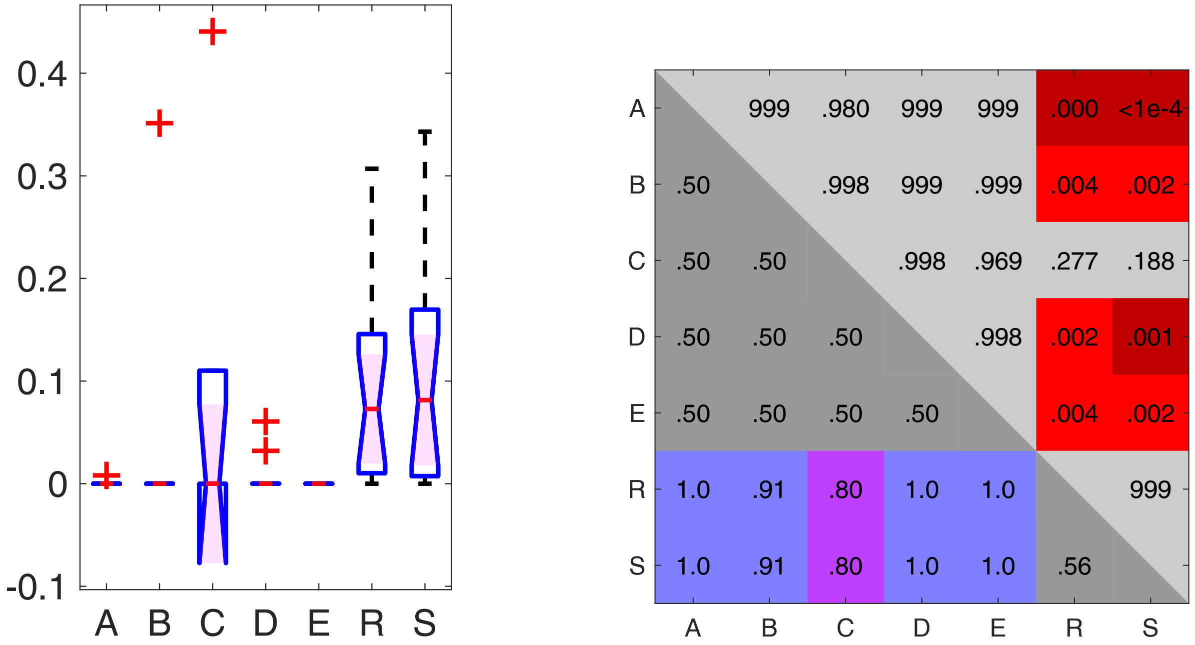


**Heatmap Analysis of Box G7**

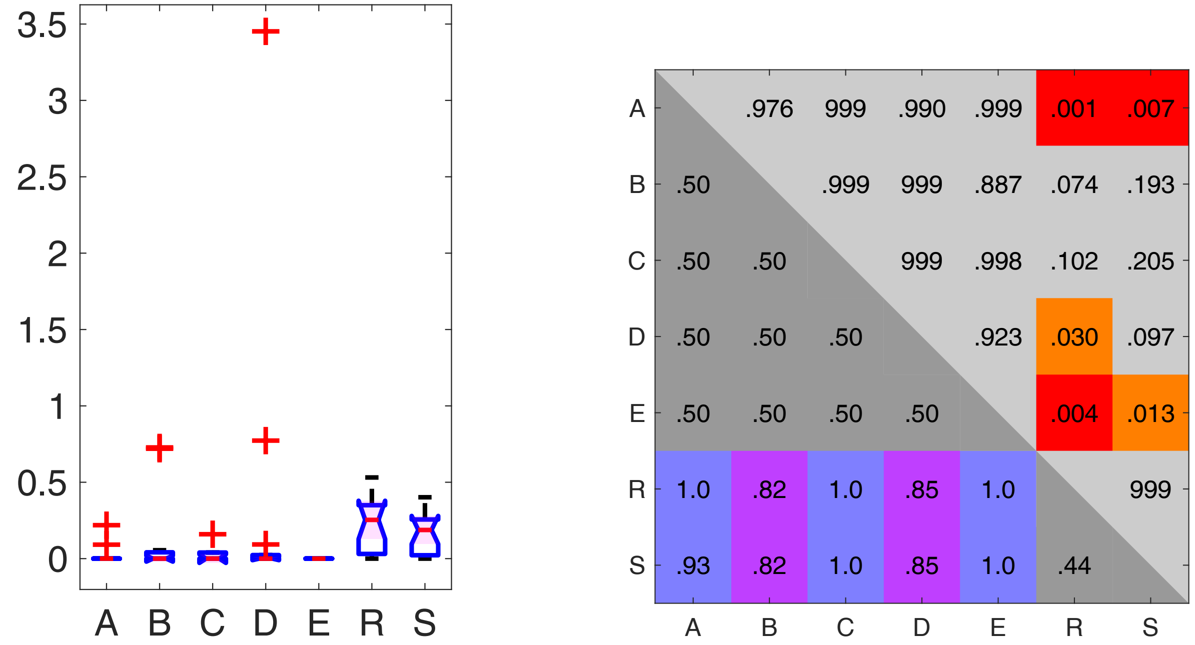

**Heatmap Analysis of Box G8**

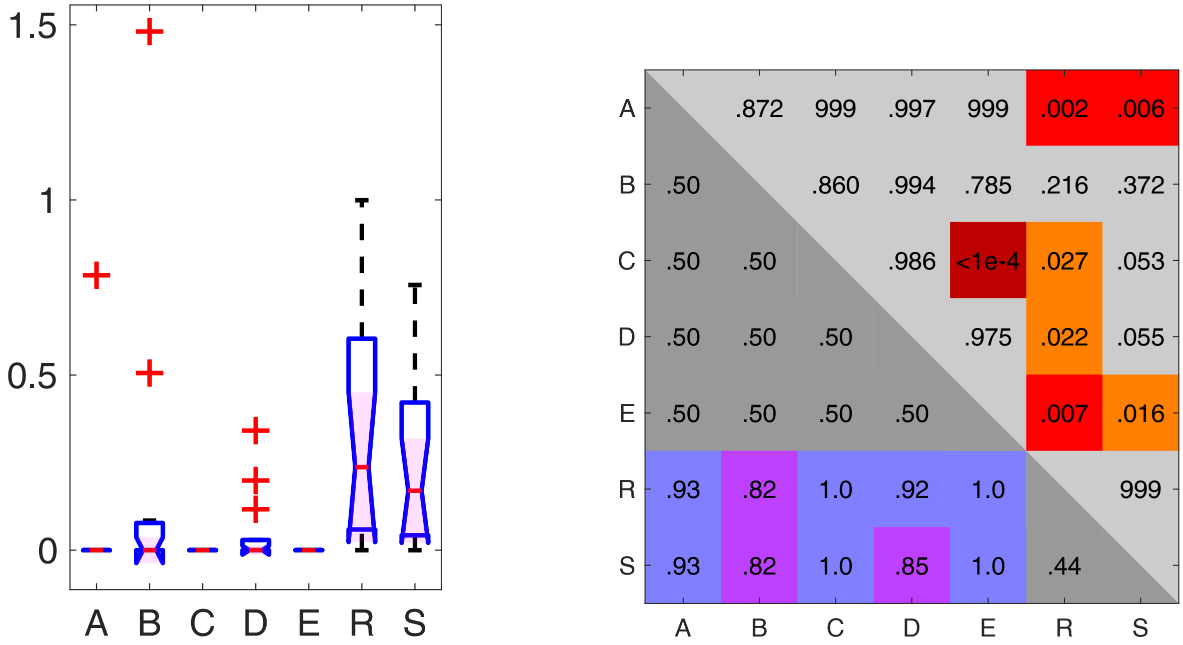


**Heatmap Analysis of Box G9**

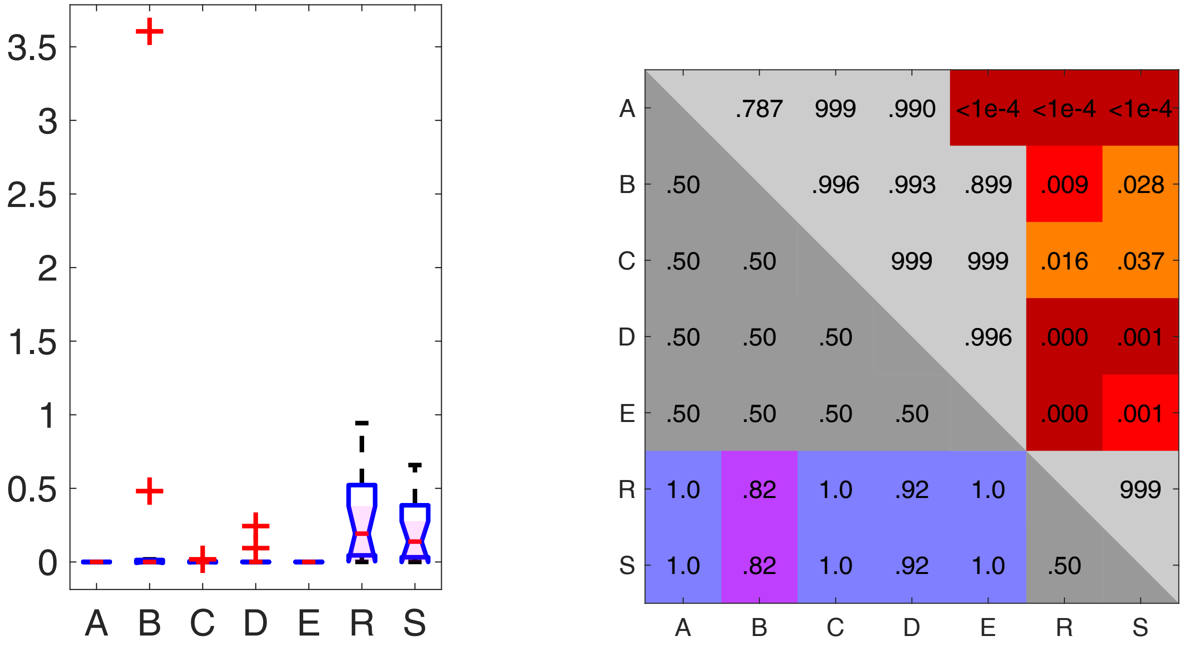

**Heatmap Analysis of Box GA**

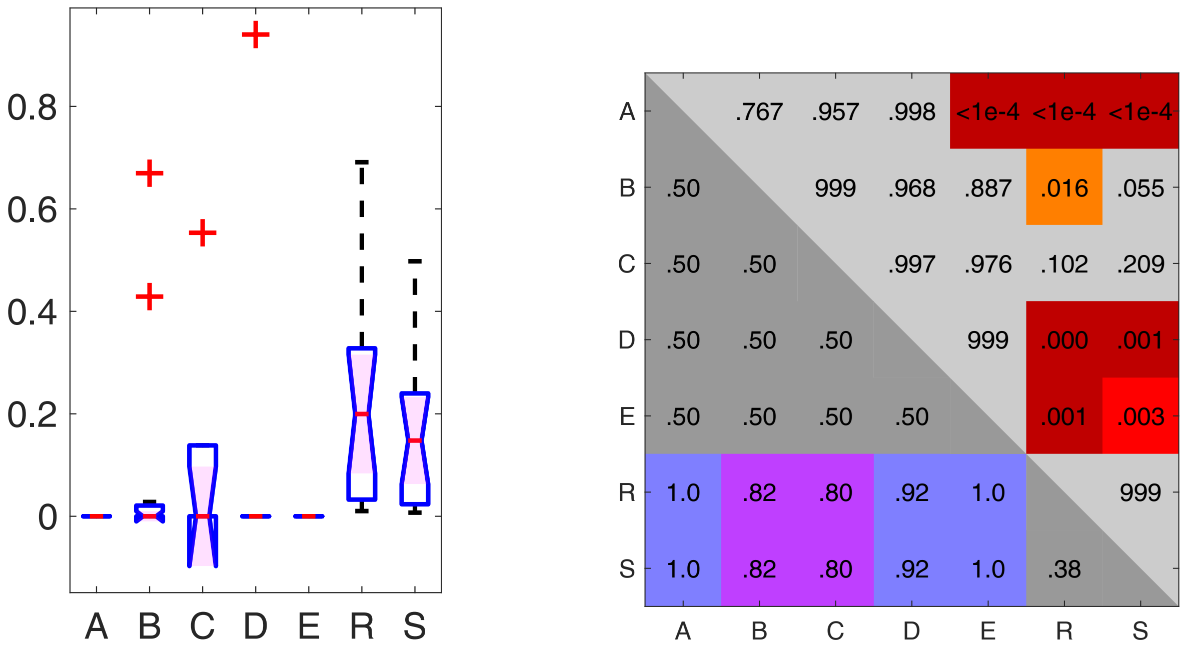


**Heatmap Analysis of Box GB**

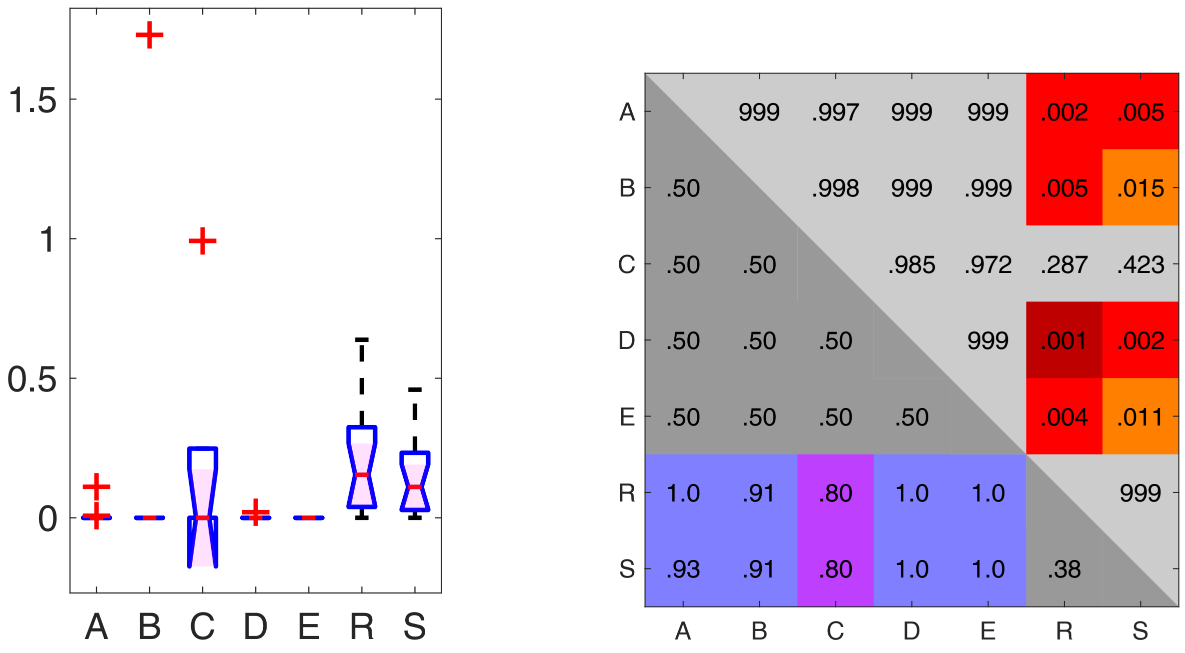

**Heatmap Analysis of Box GC**

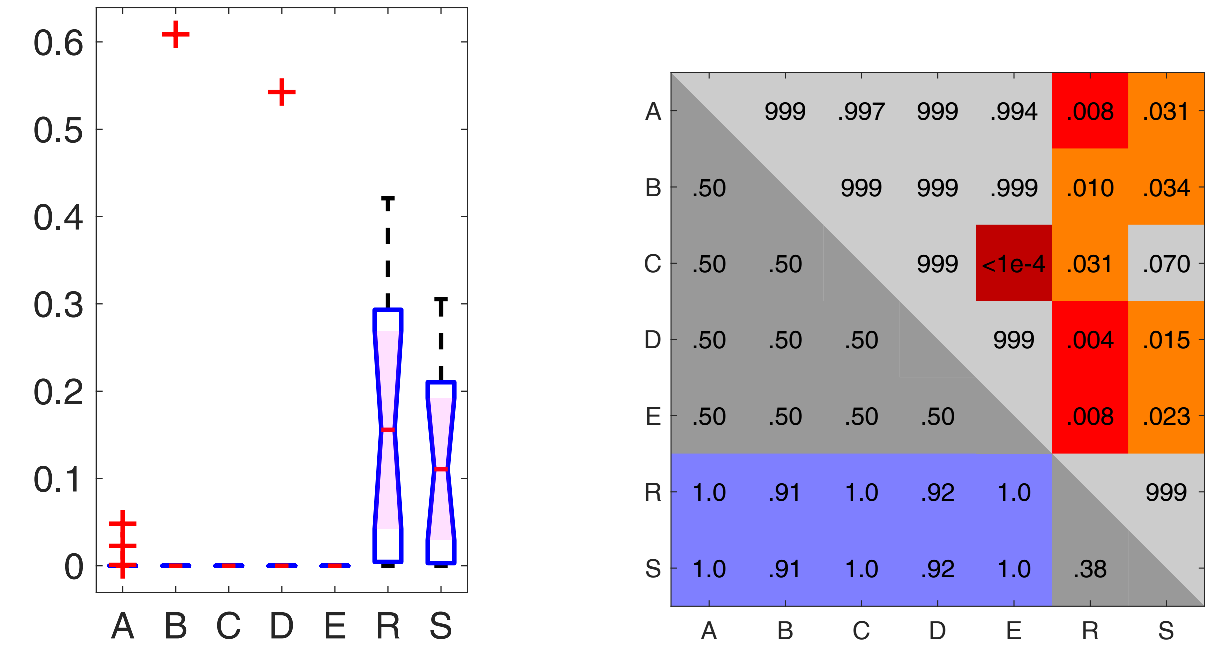


**Heatmap Analysis of Box GD**

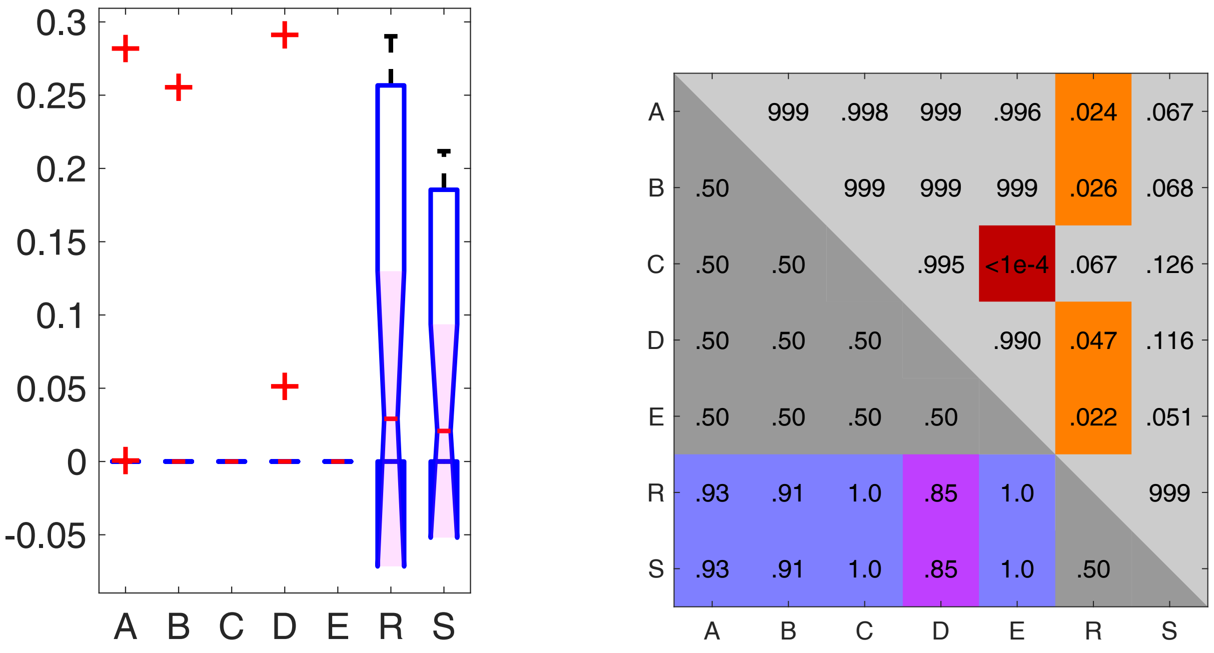

**Heatmap Analysis of Box GE**

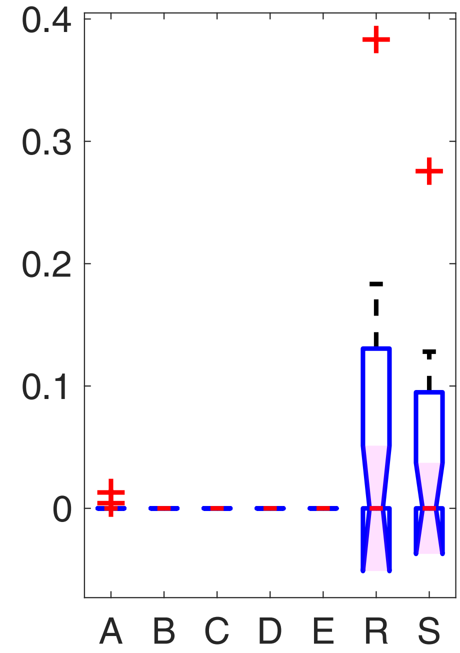


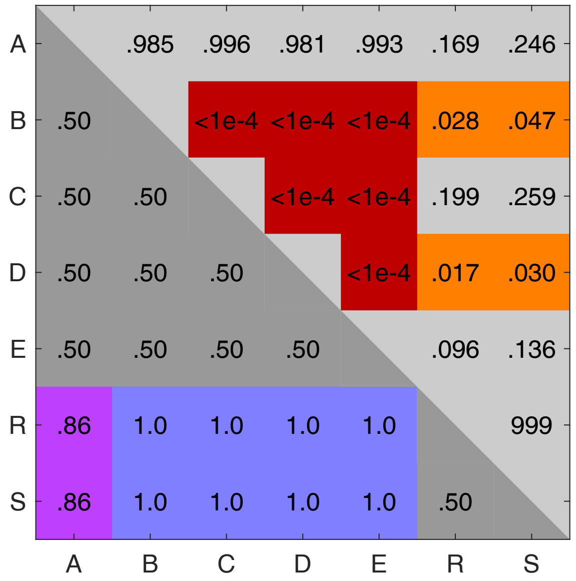


**Heatmap Analysis of Box GF**

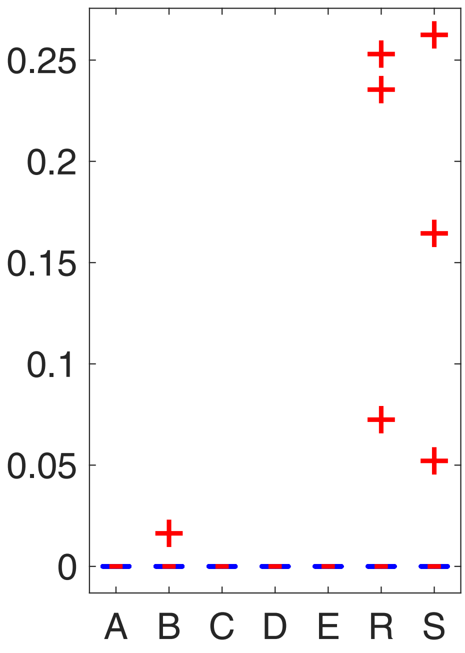


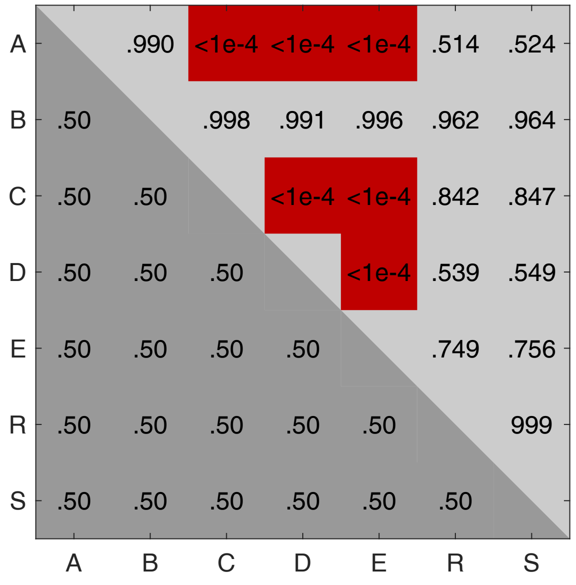

**Heatmap Analysis of Box GG**

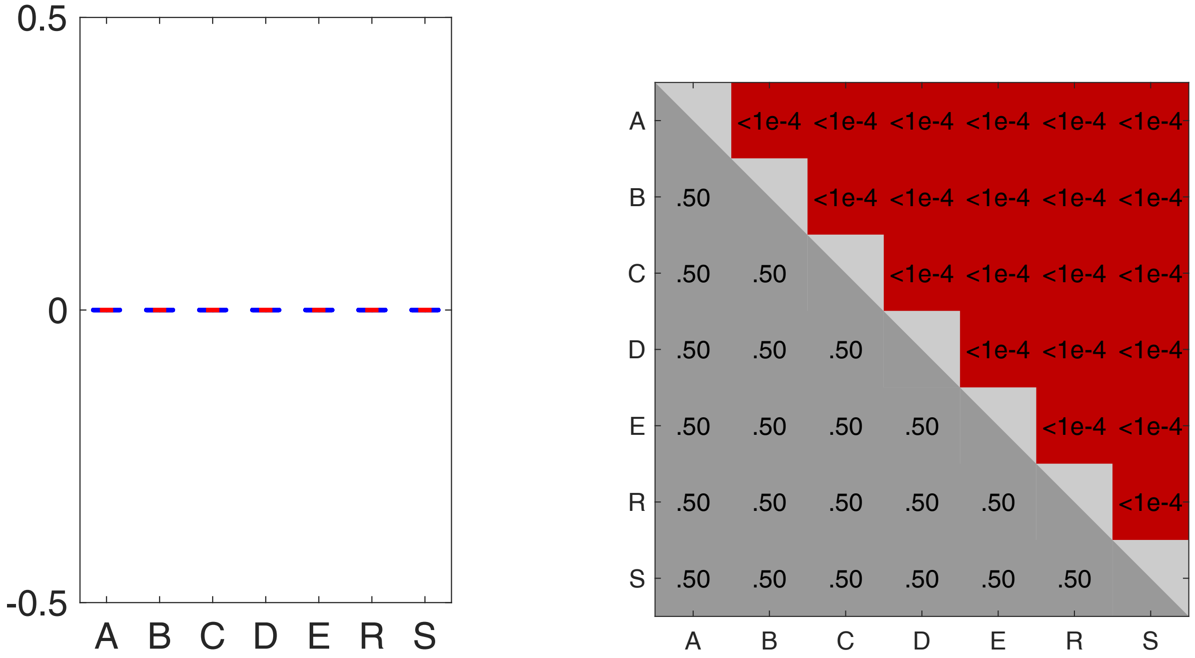


**Heatmap Analysis of Box GH**

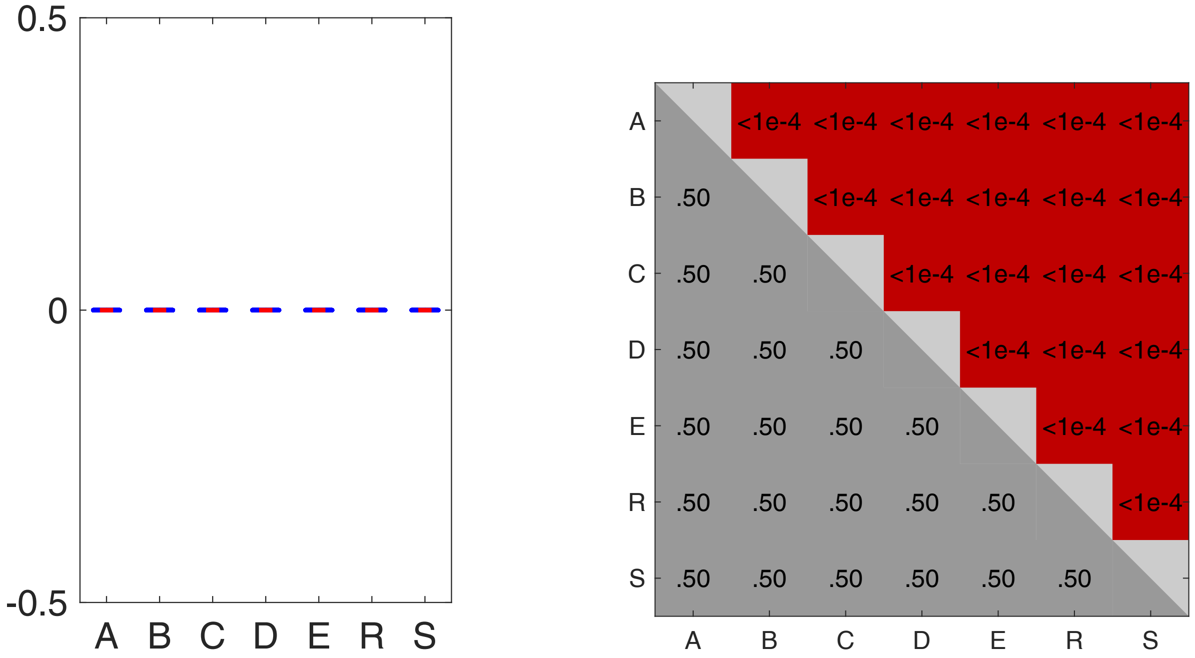

**Heatmap Analysis of Box H0**

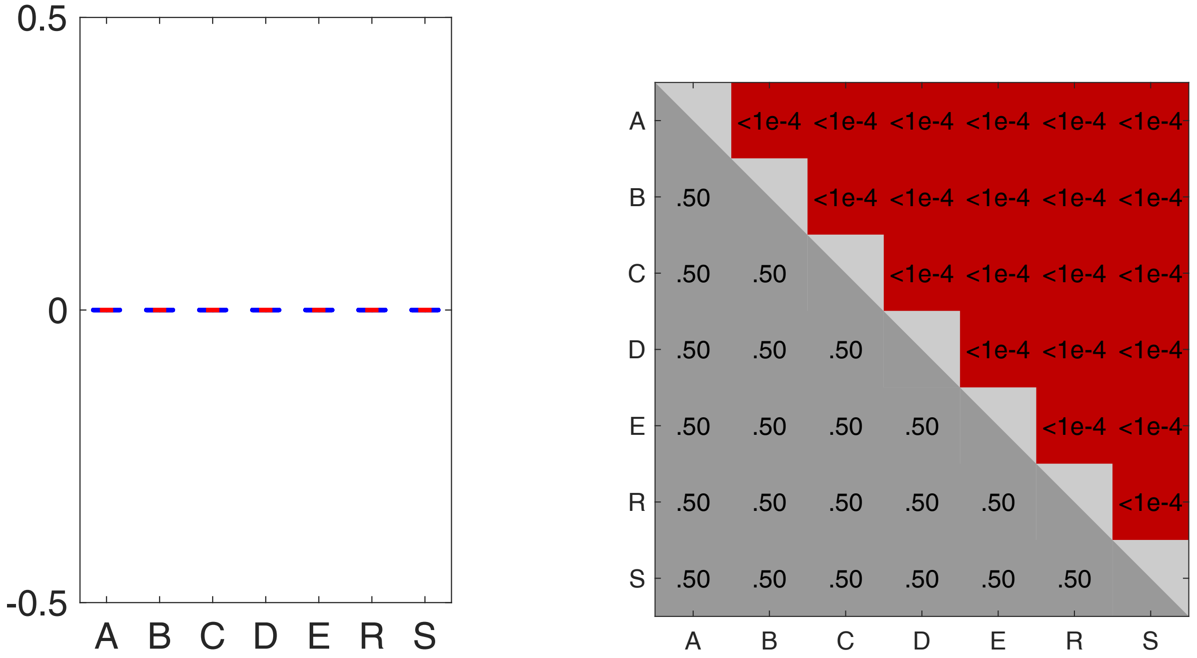


**Heatmap Analysis of Box H1**

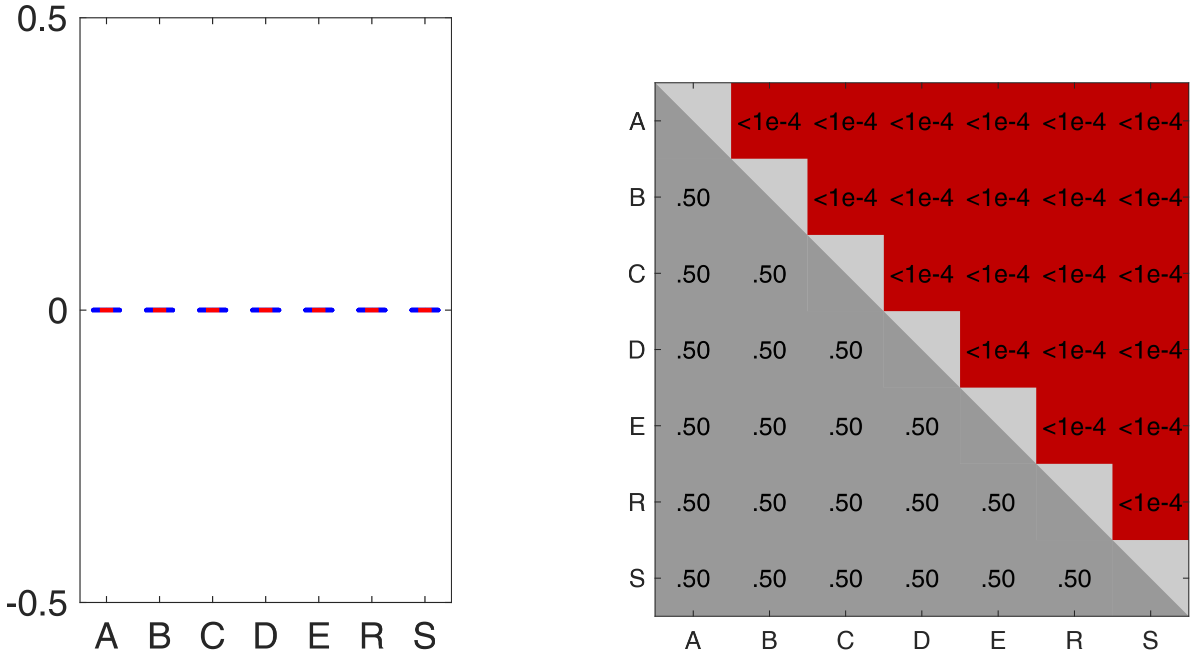

**Heatmap Analysis of Box H2**

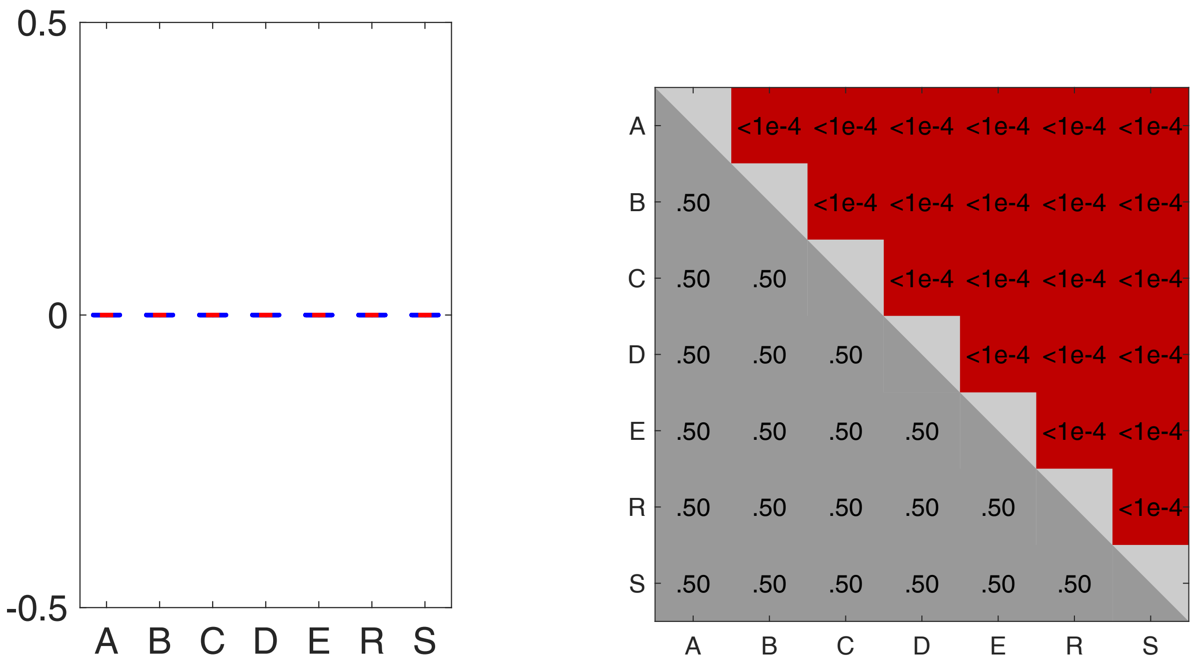


**Heatmap Analysis of Box H3**

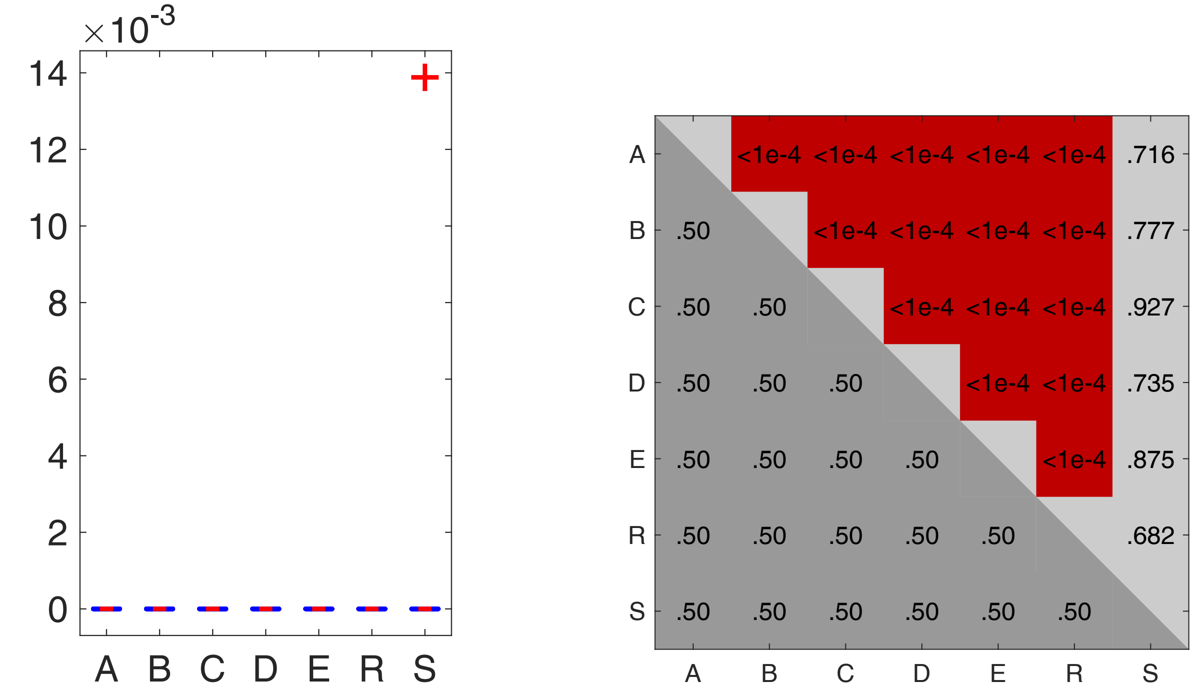

**Heatmap Analysis of Box H4**

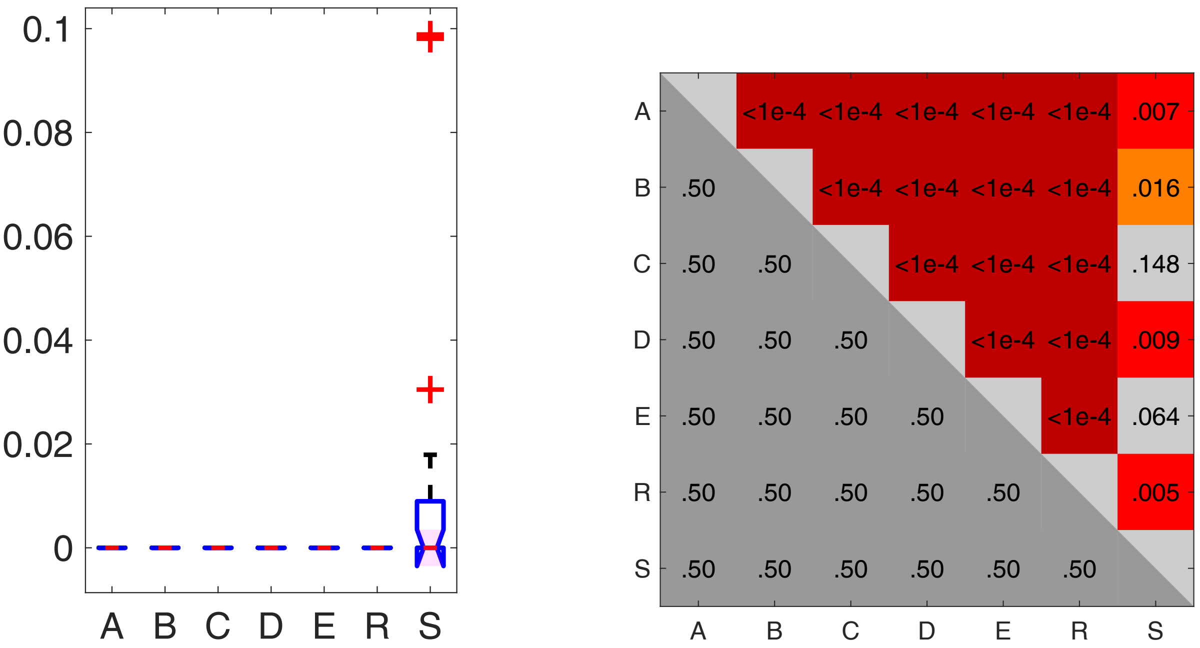


**Heatmap Analysis of Box H5**

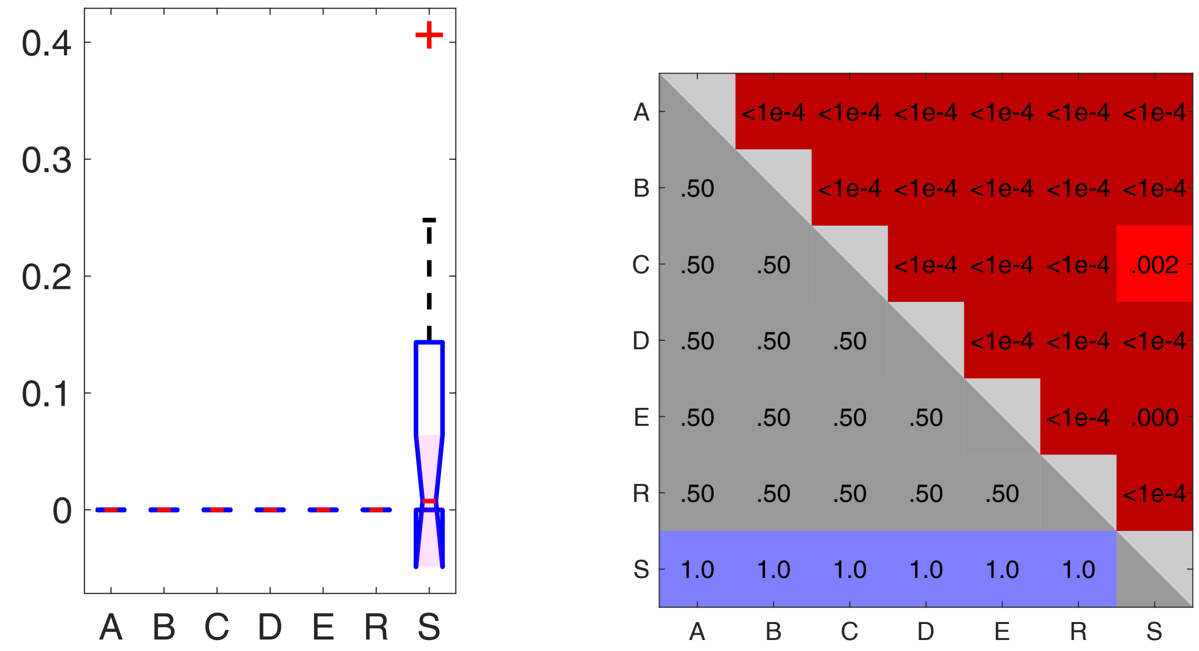

**Heatmap Analysis of Box H6**

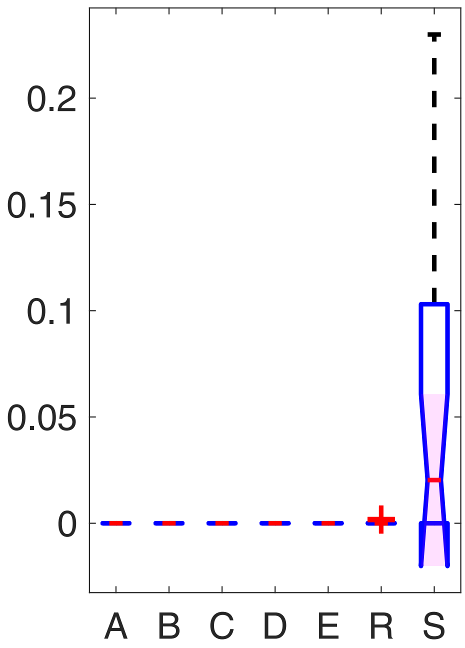


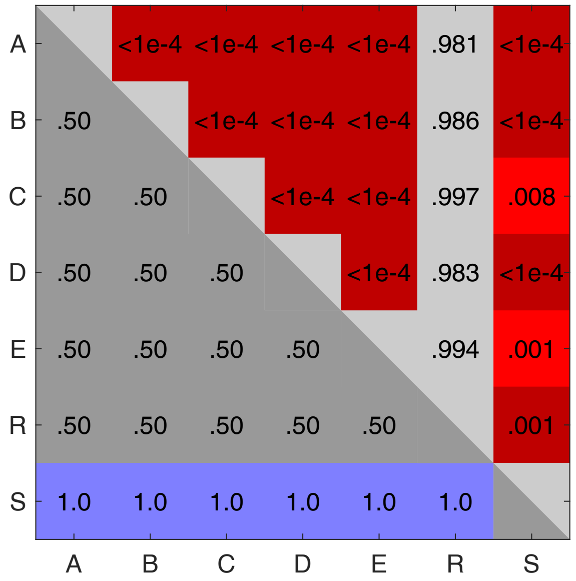


**Heatmap Analysis of Box H7**

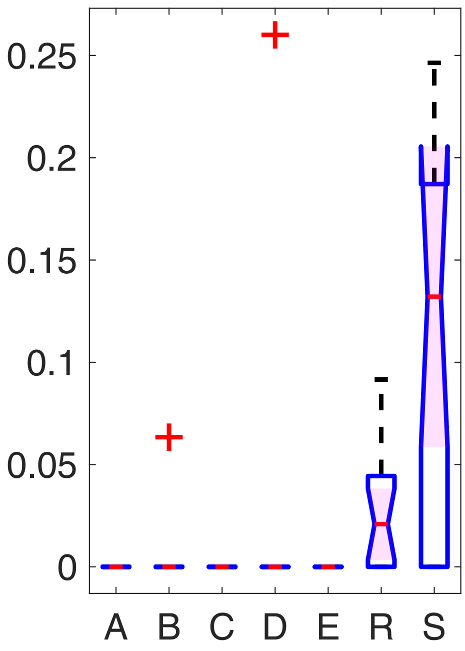


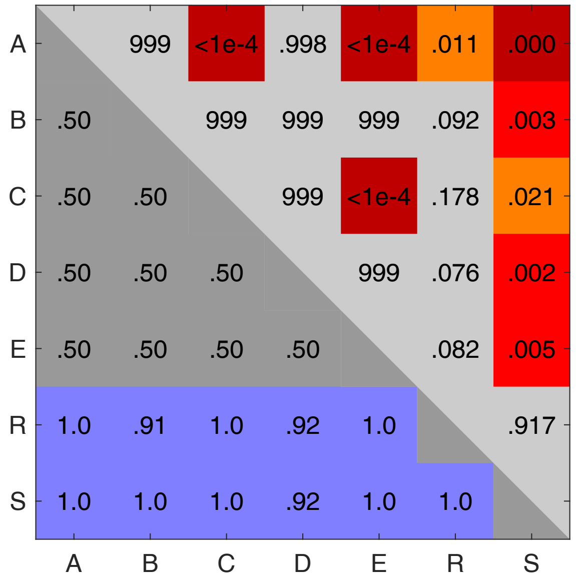

**Heatmap Analysis of Box H8**

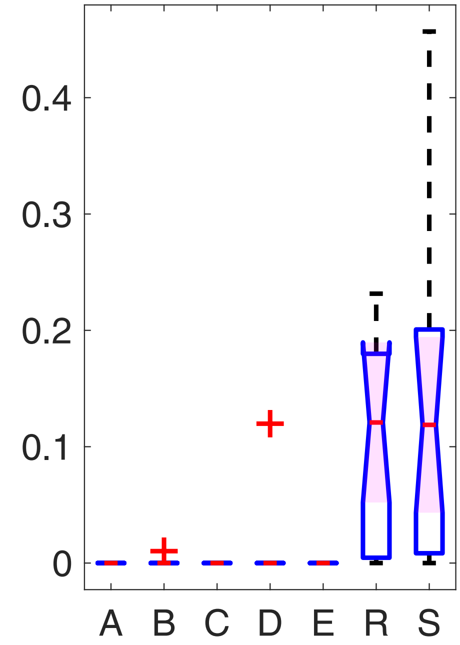


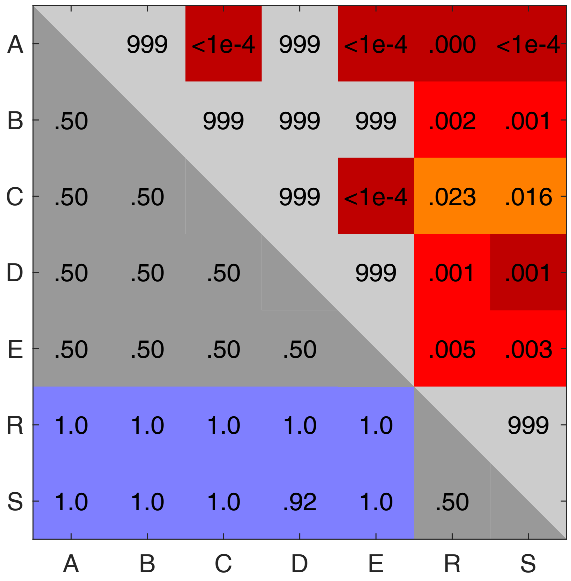


**Heatmap Analysis of Box H9**

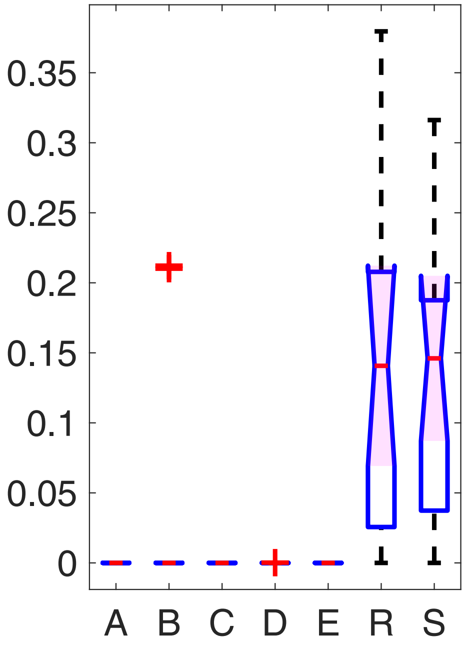


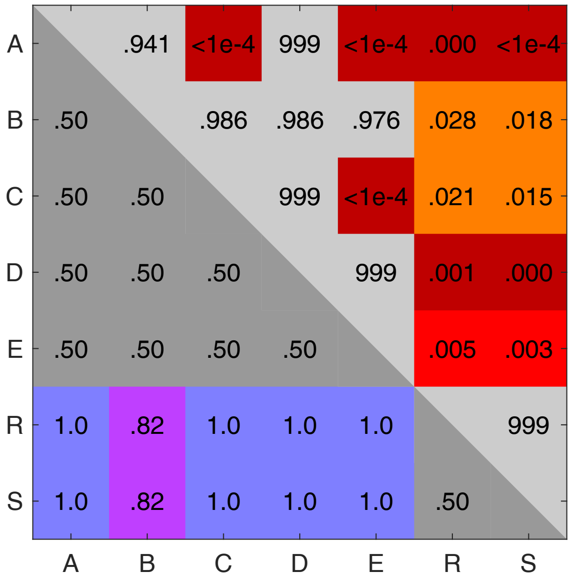

**Heatmap Analysis of Box HA**

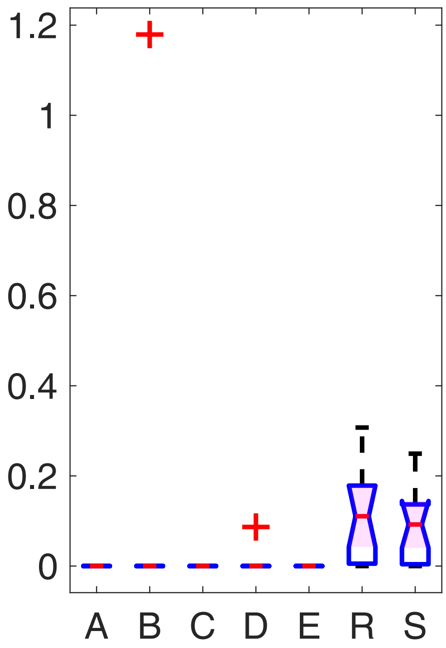


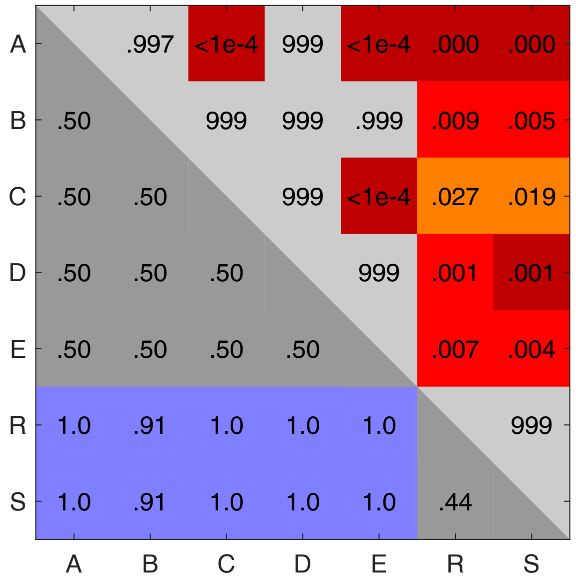


**Heatmap Analysis of Box HB**

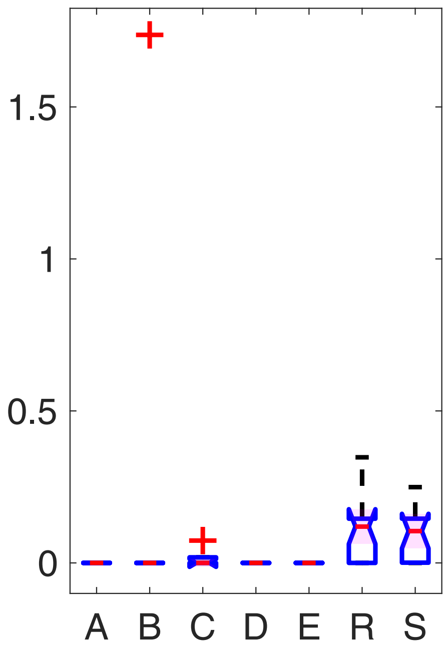


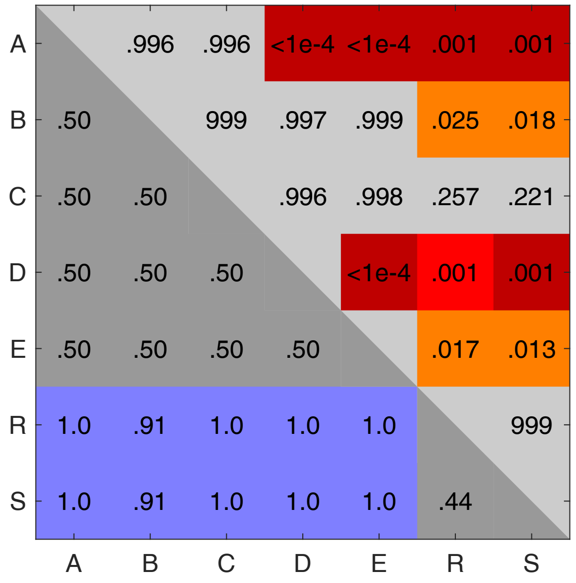

**Heatmap Analysis of Box HC**

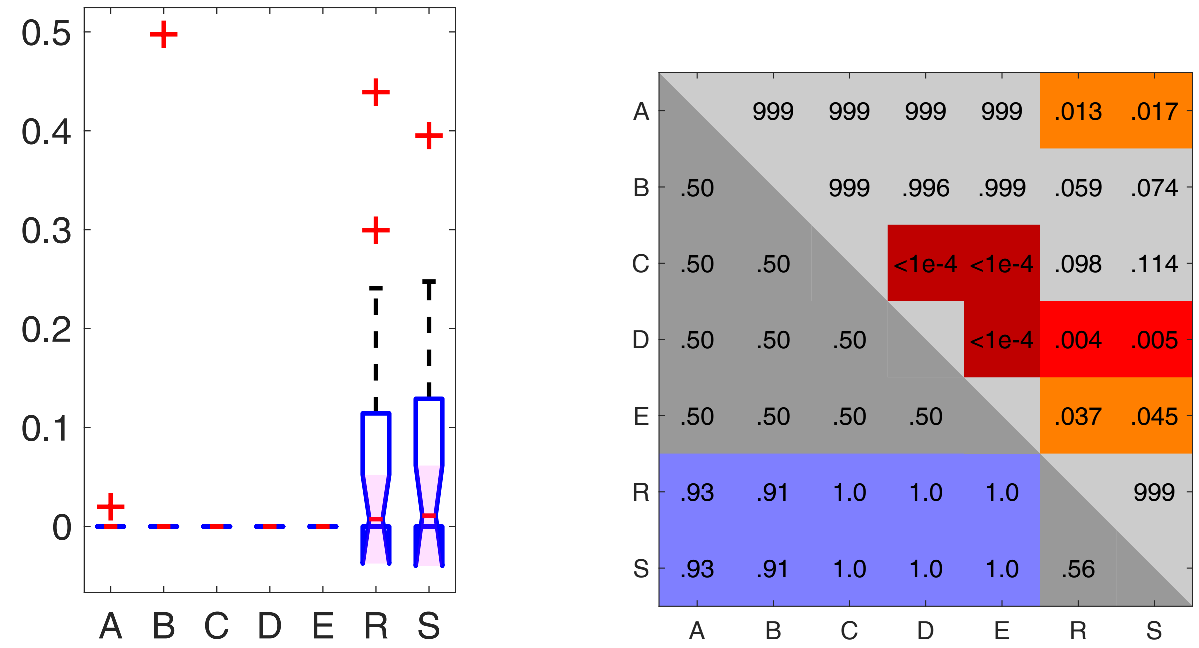


**Heatmap Analysis of Box HD**

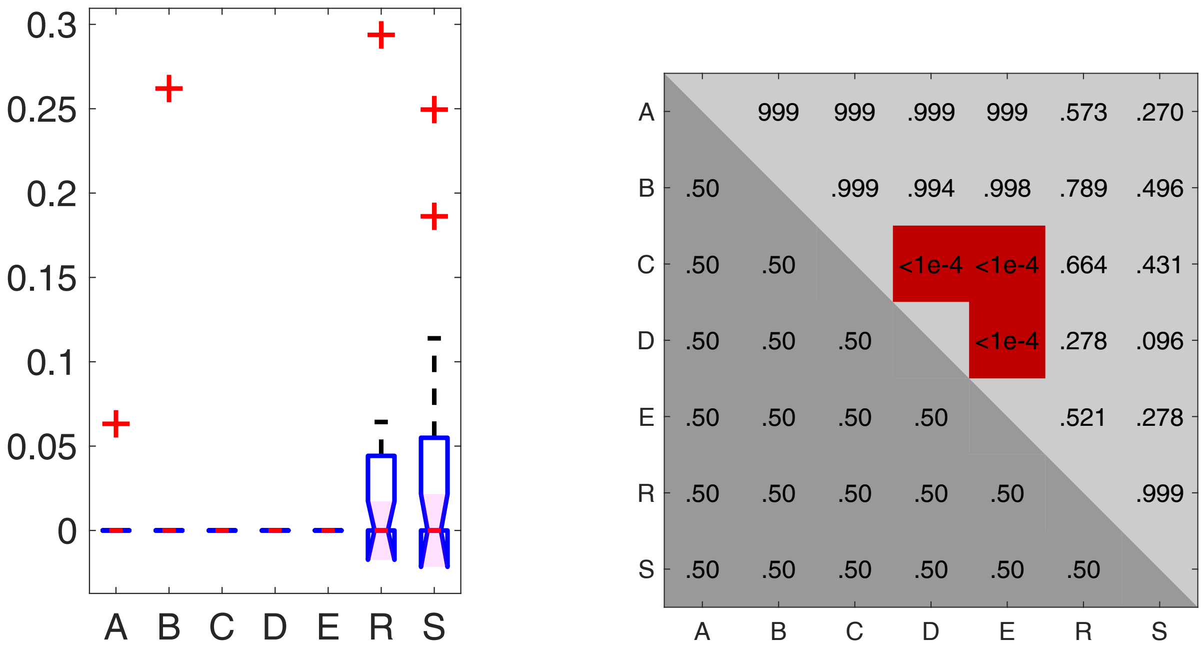

### Heatmap Analysis of Box HE

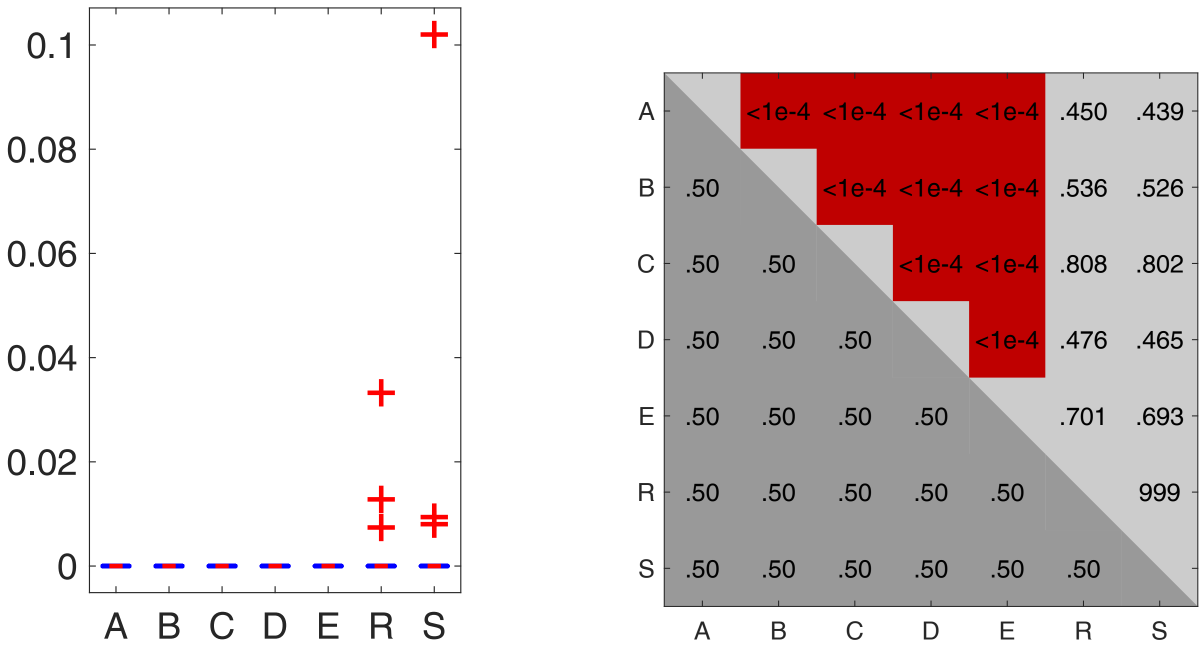


### Heatmap Analysis of Box HF

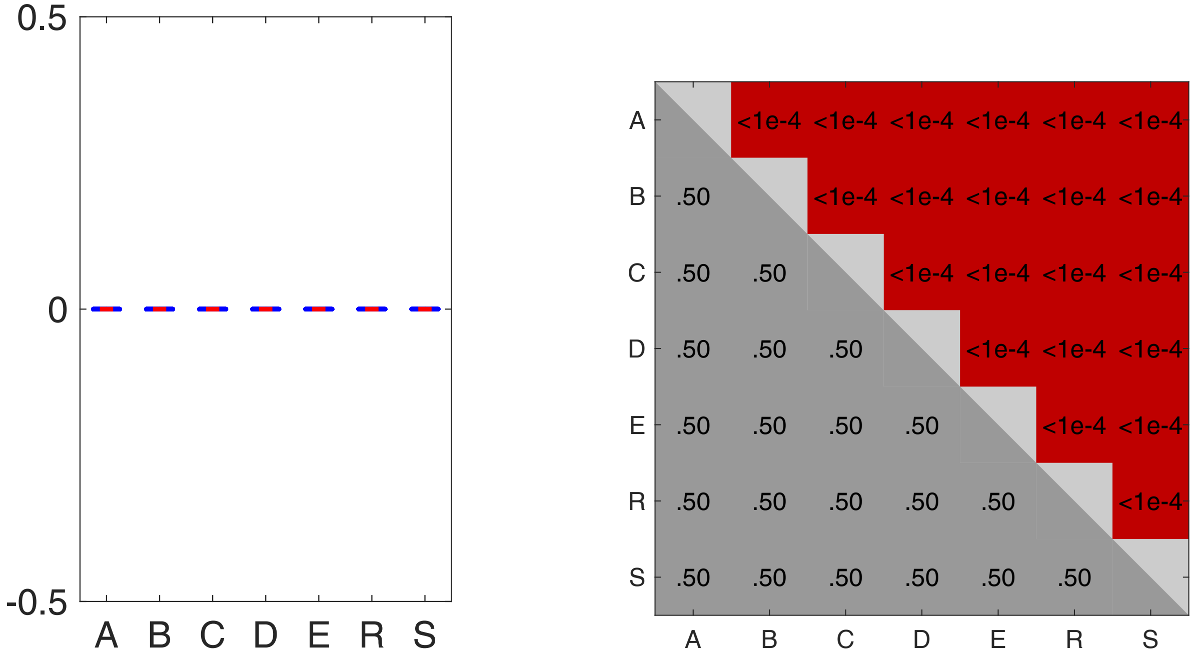

**Heatmap Analysis of Box HG**

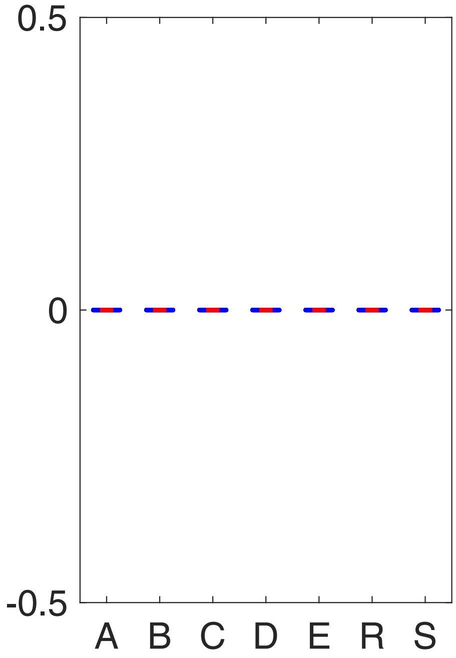


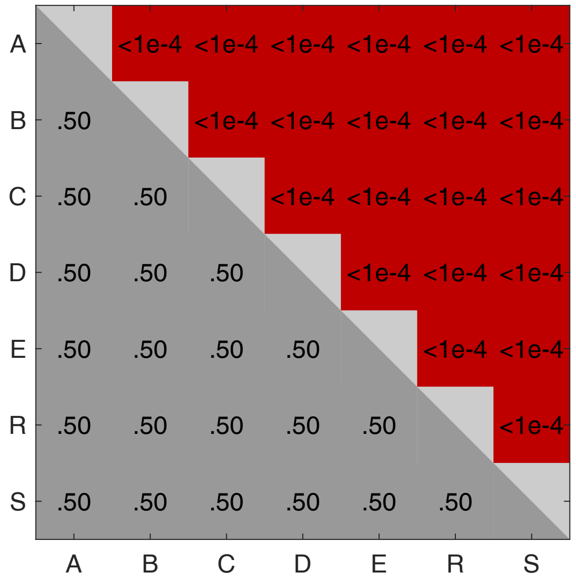


**Heatmap Analysis of Box HH**

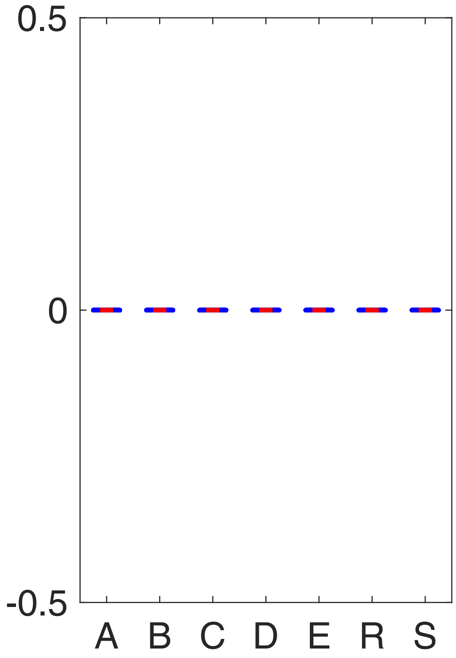


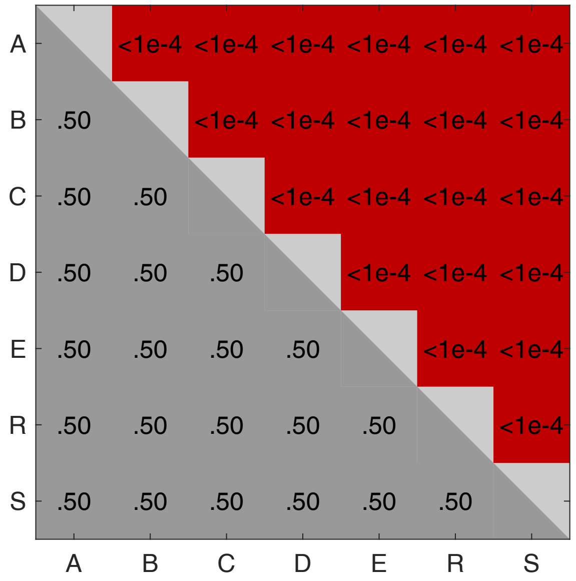

**Heatmap Analysis of Box I0**

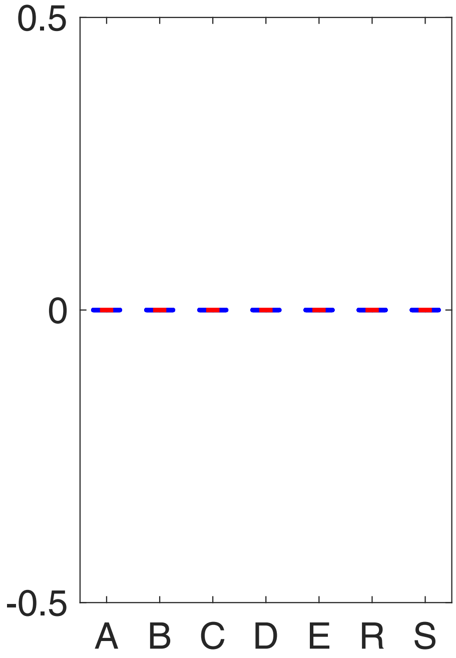


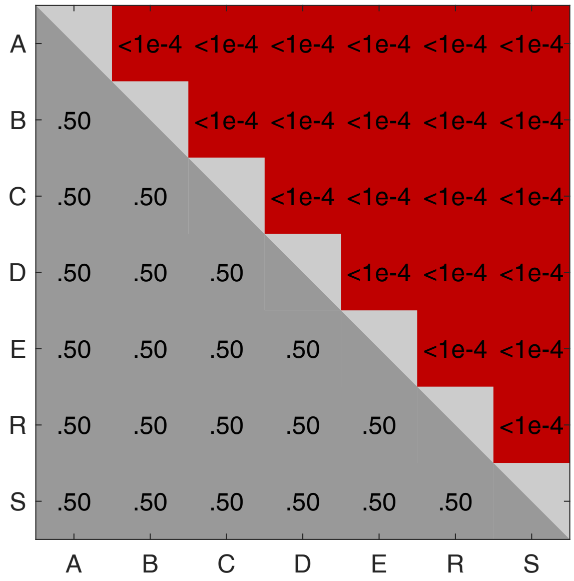


**Heatmap Analysis of Box I1**

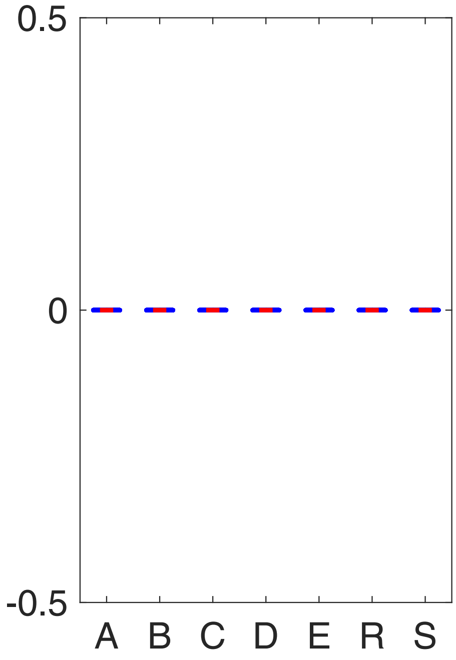


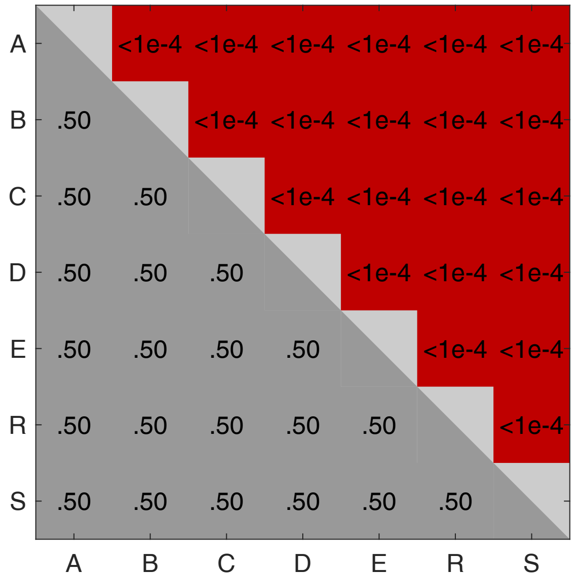

**Heatmap Analysis of Box I2**

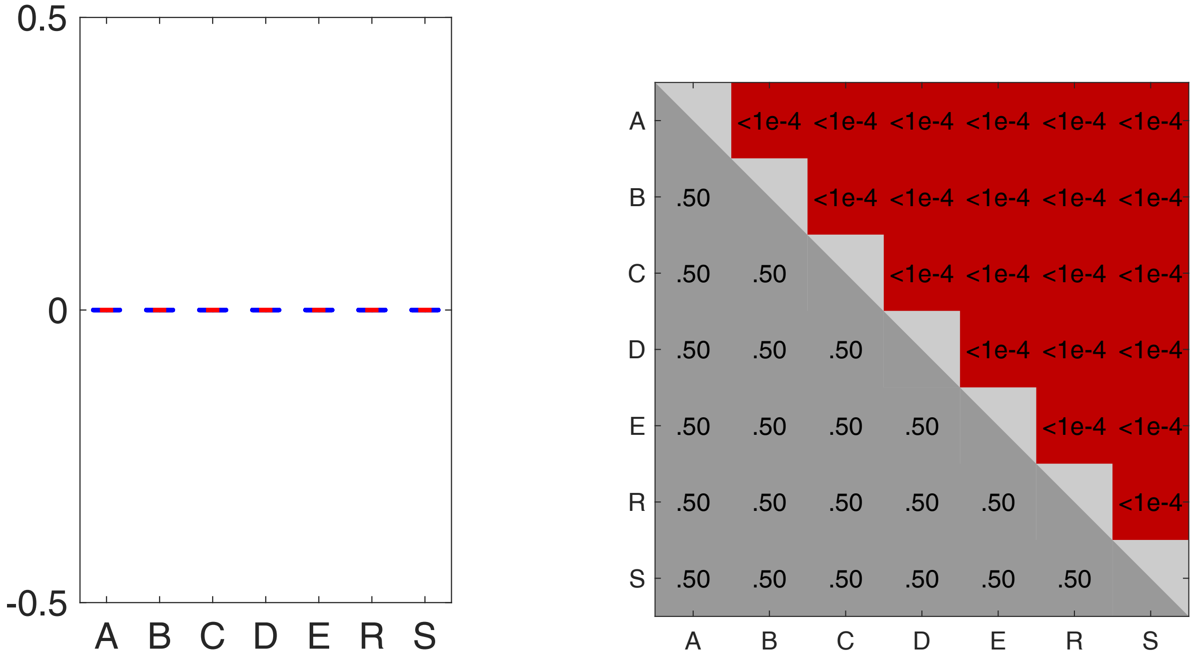


**Heatmap Analysis of Box I3**

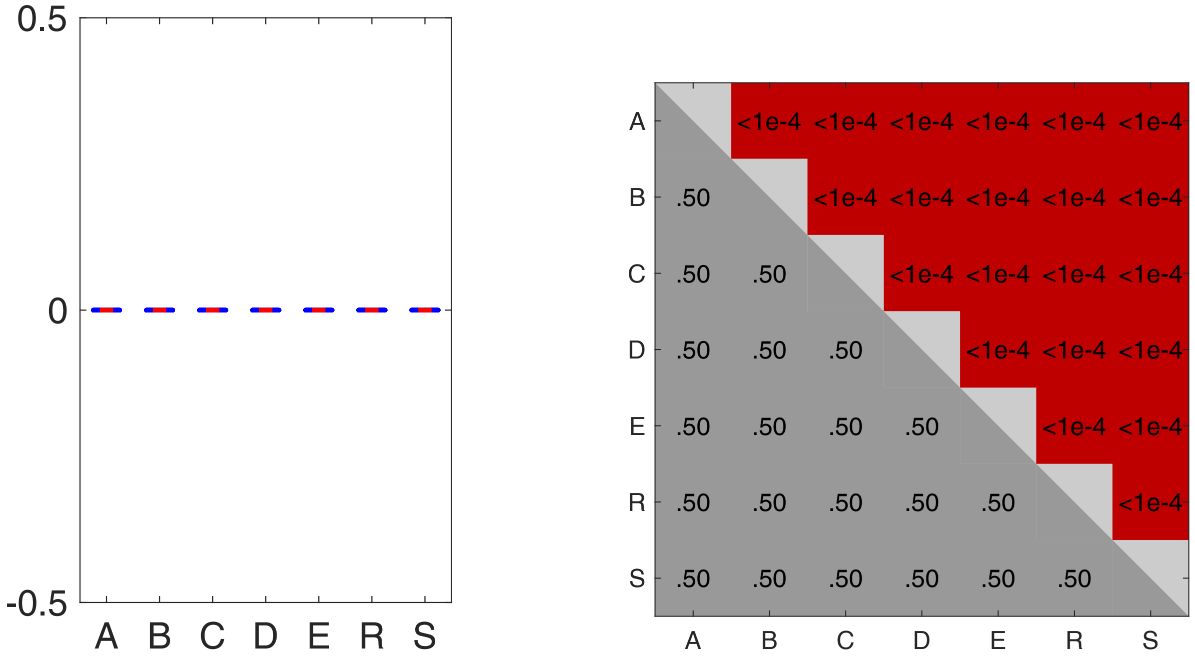

**Heatmap Analysis of Box I4**

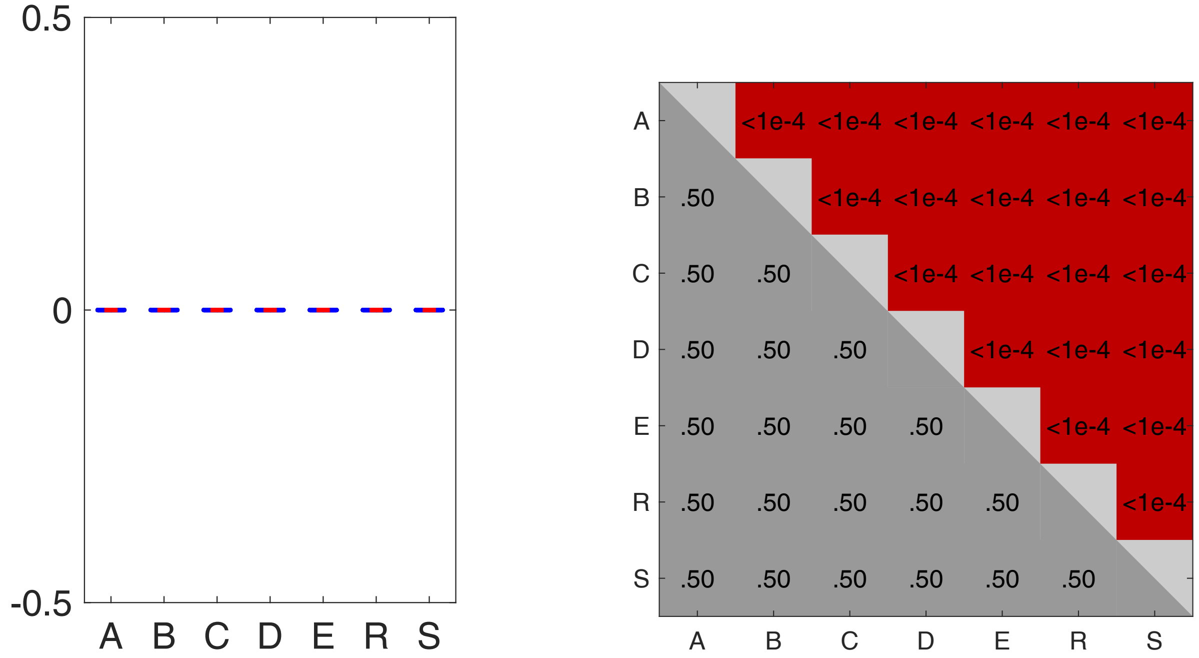


**Heatmap Analysis of Box I5**

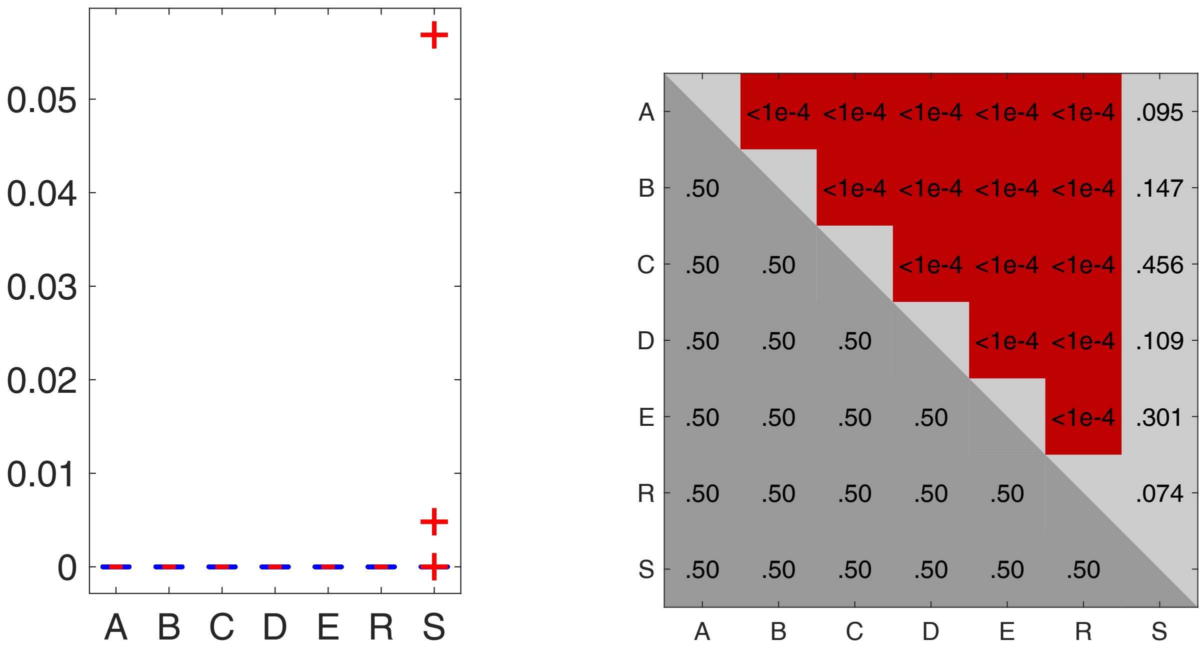

**Heatmap Analysis of Box I6**

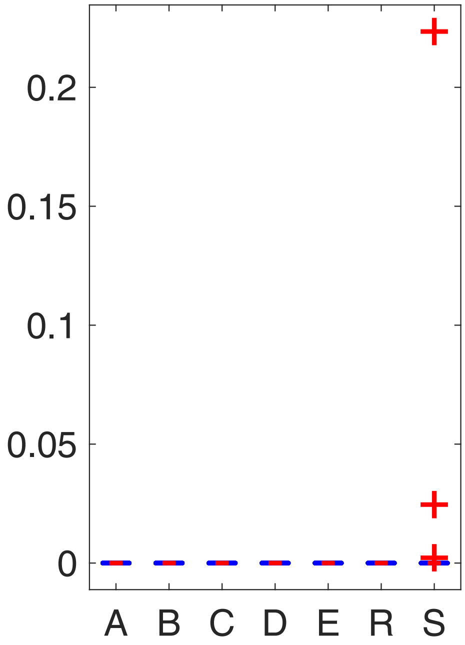


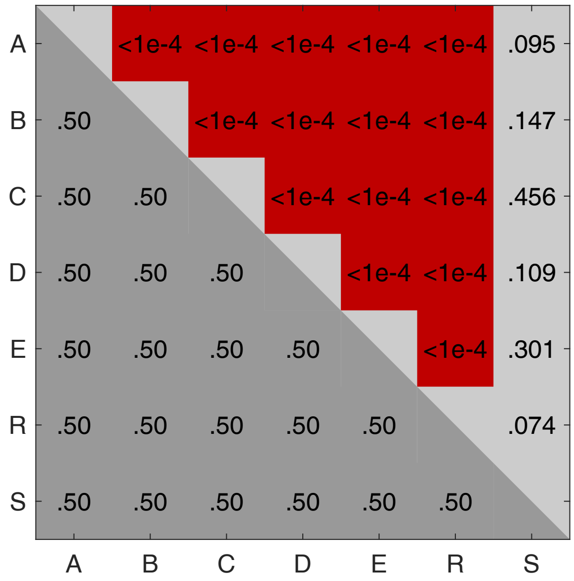


**Heatmap Analysis of Box I7**

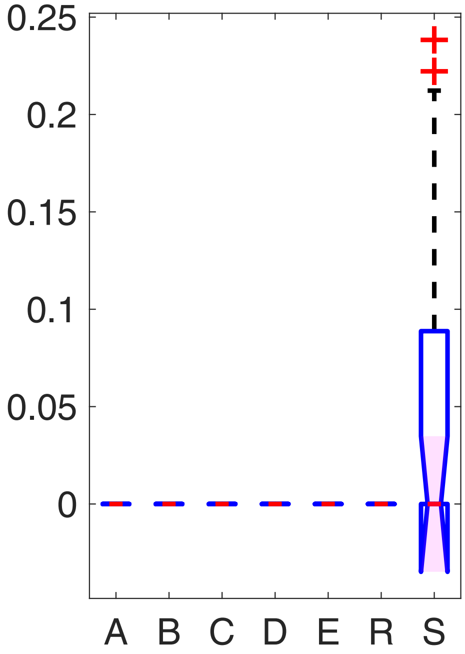


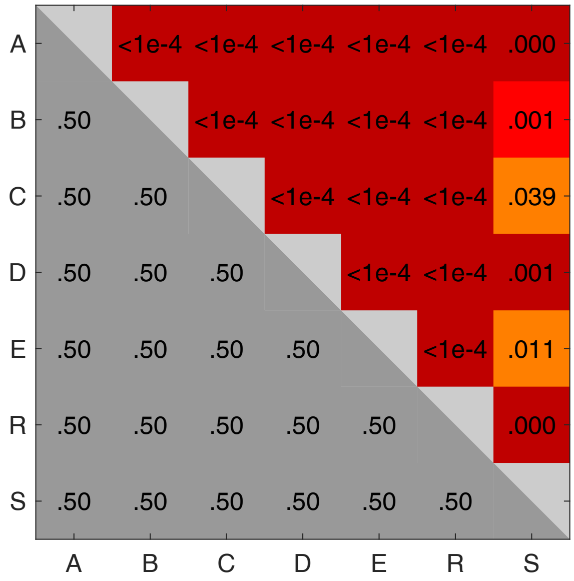

**Heatmap Analysis of Box I8**

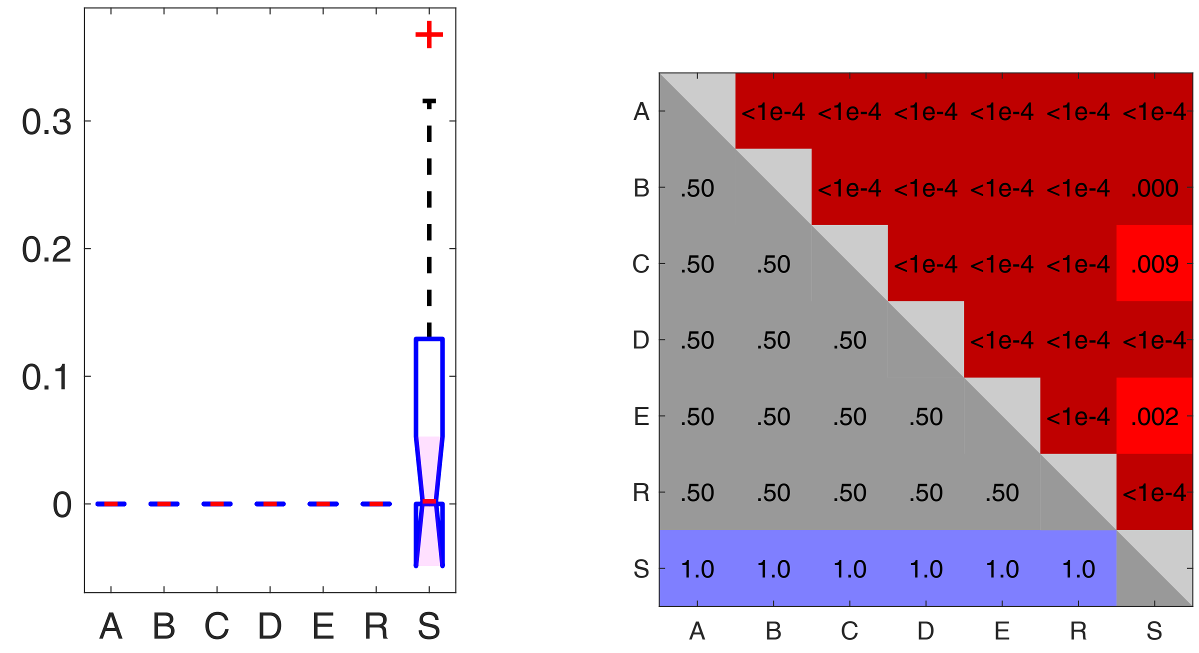


**Heatmap Analysis of Box I9**

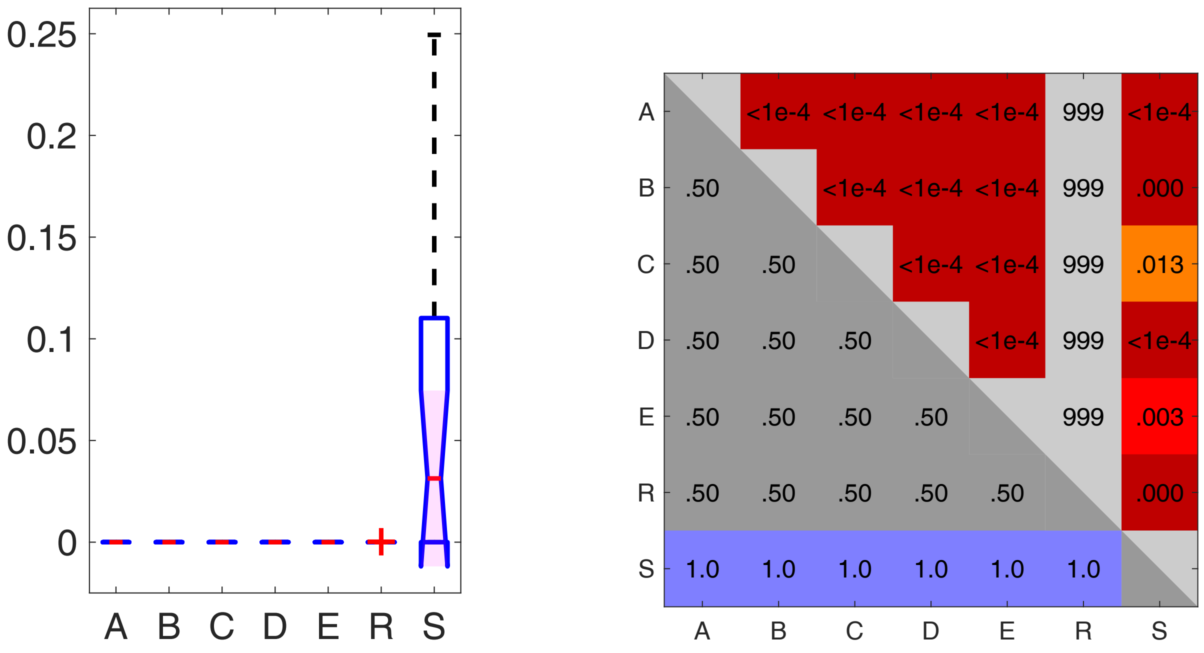

**Heatmap Analysis of Box IA**

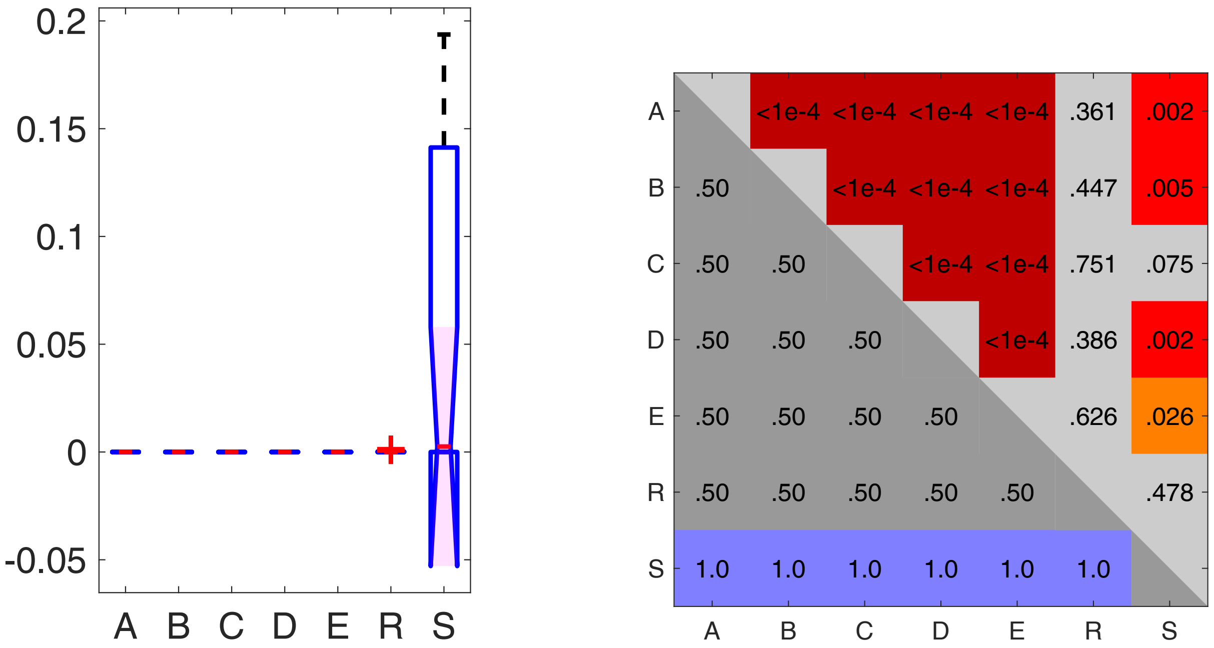


**Heatmap Analysis of Box IB**

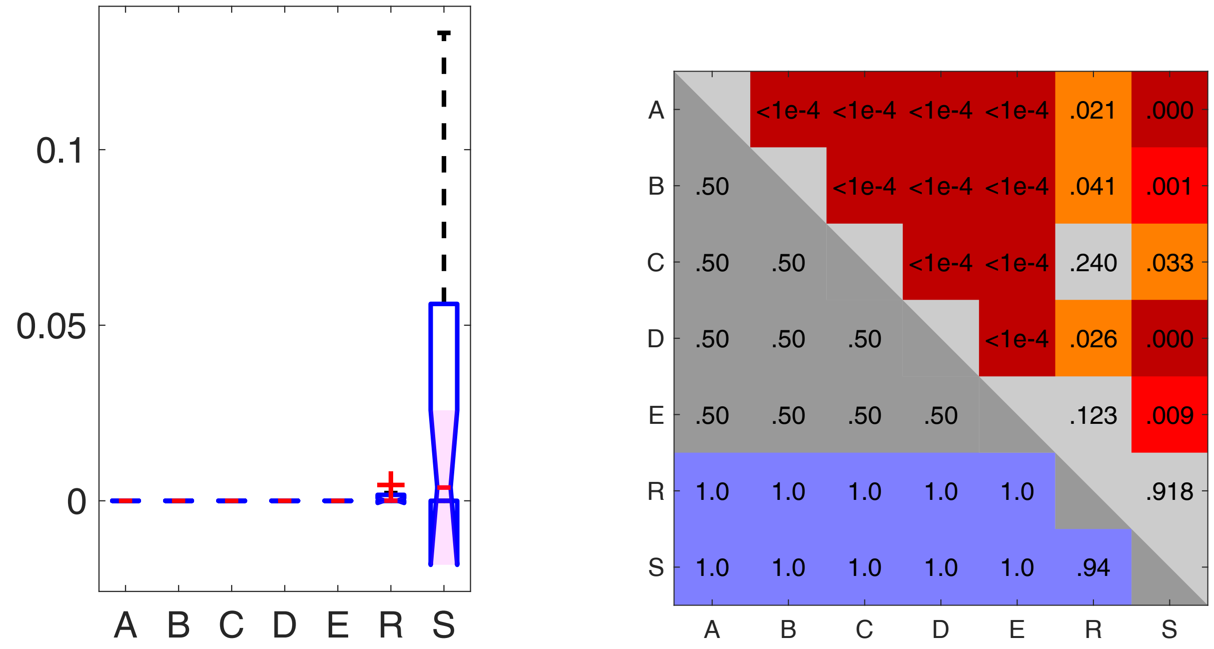

**Heatmap Analysis of Box IC**

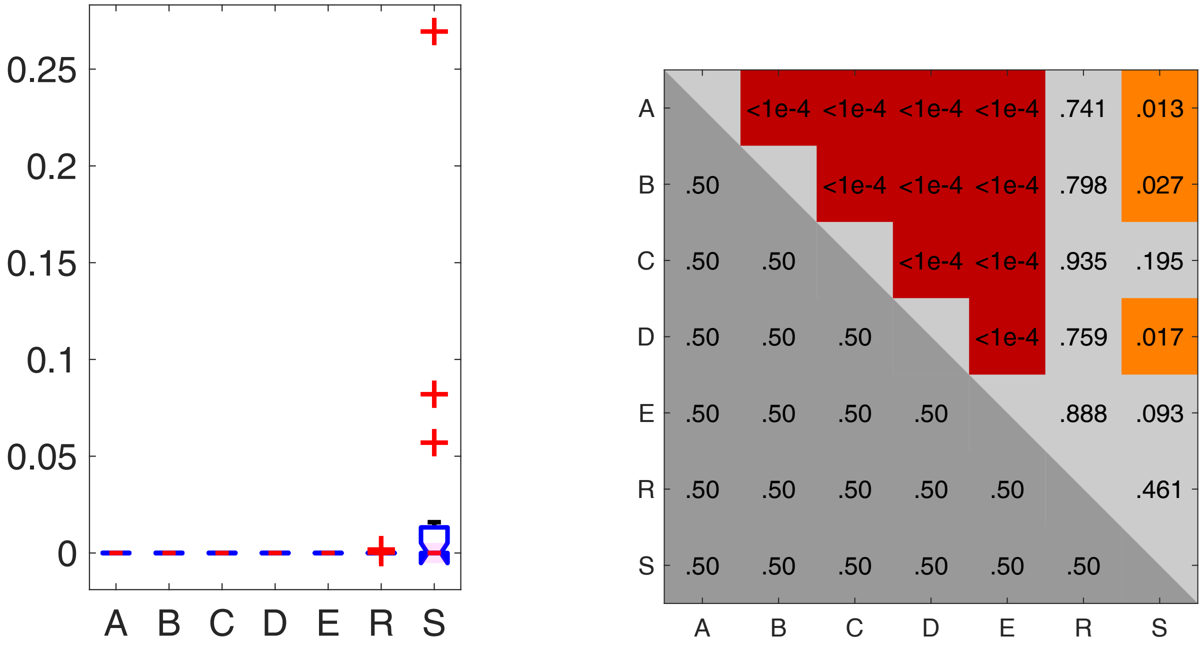


**Heatmap Analysis of Box ID**

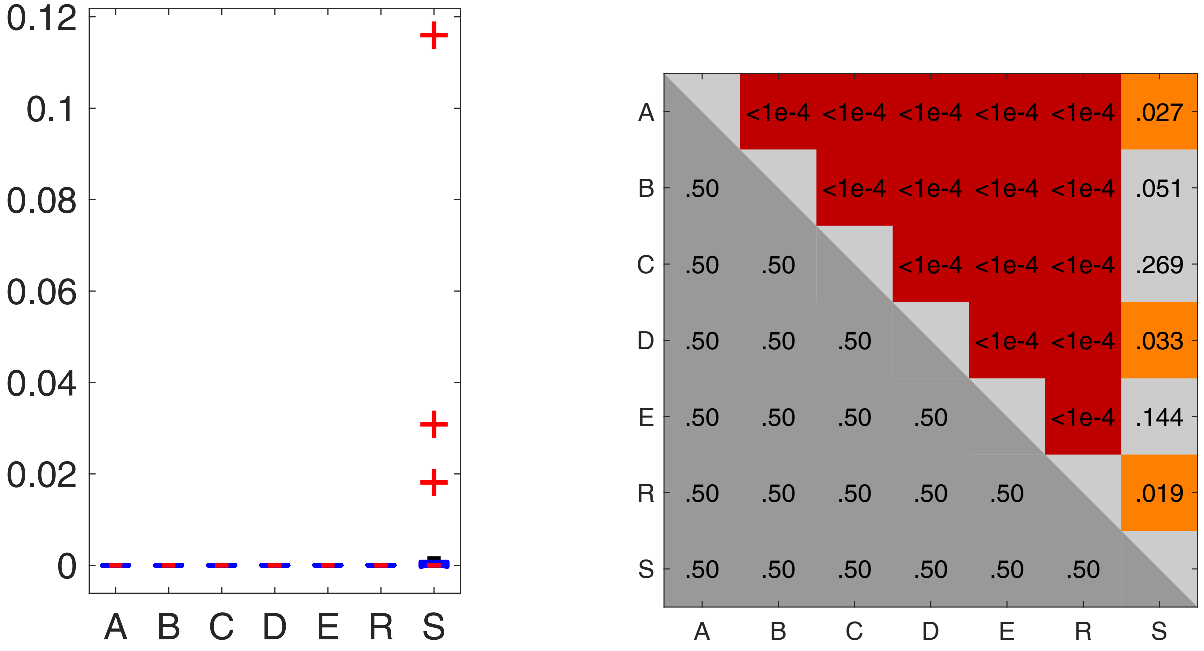

**Heatmap Analysis of Box IE**

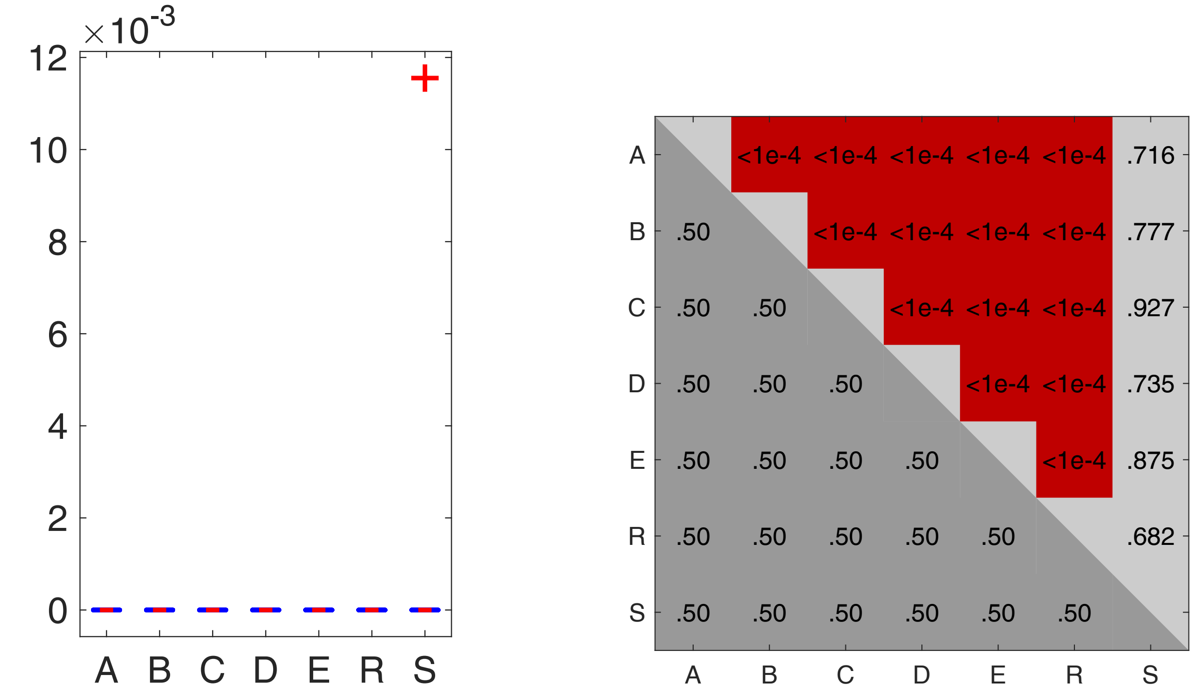


**Heatmap Analysis of Box IF**

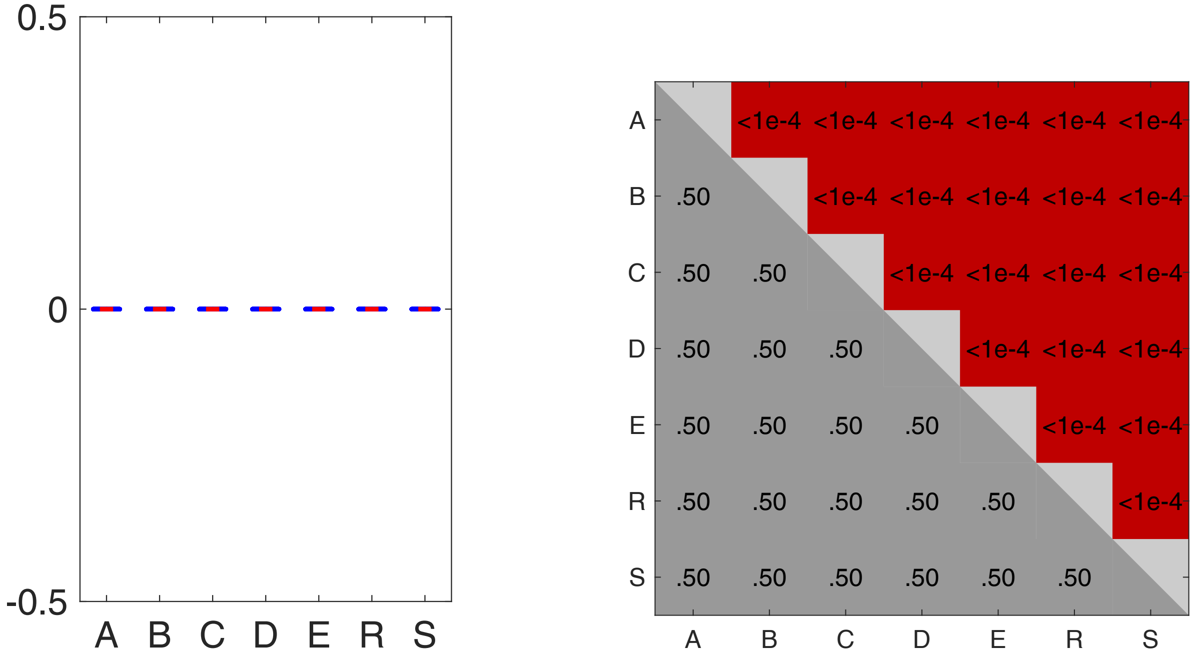

**Heatmap Analysis of Box IG**

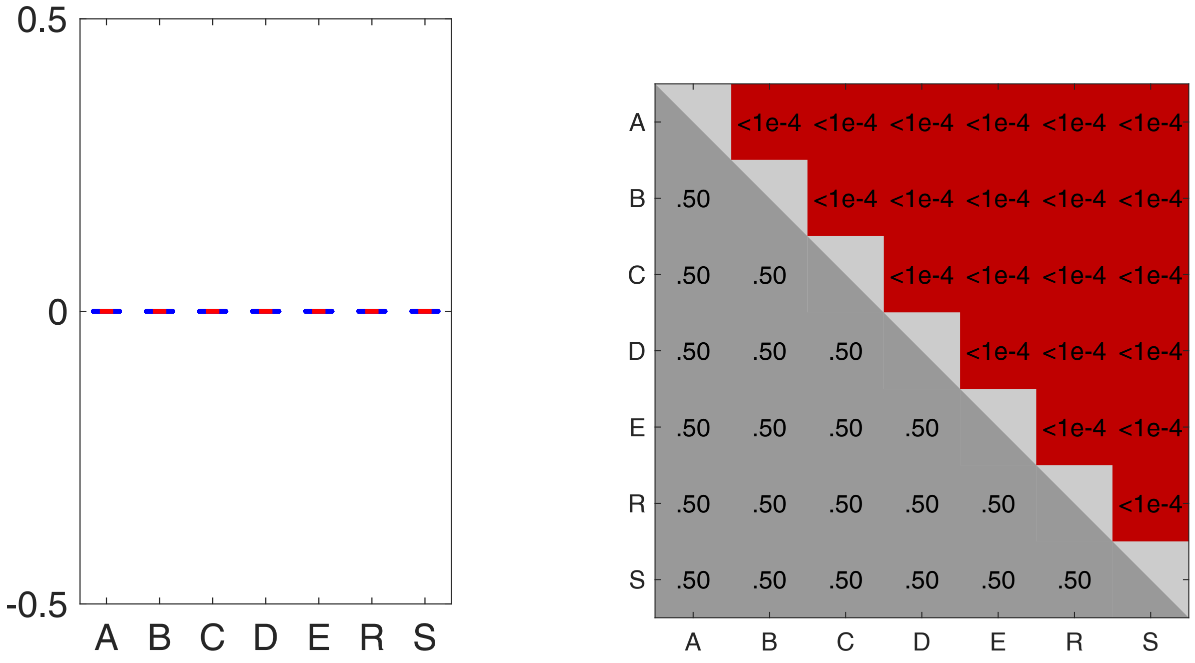


**Heatmap Analysis of Box IH**

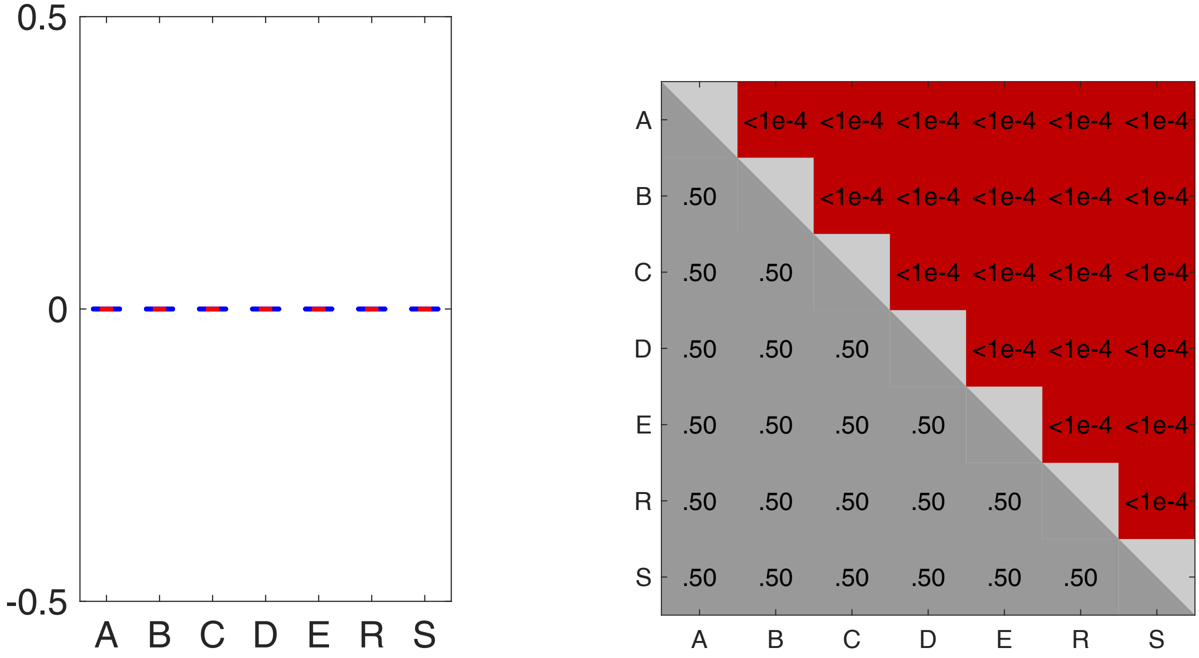

Supplement: Supplementary Data Sheet S5 — Significance analyses of heat map tiles. [file Data_Sheet_5.pdf]
