# Supplementary material for: Generalization of navigation memory in honeybees
Source: Front Behav Neurosci. 2023 Mar 6;17:1070957. doi: 10.3389/fnbeh.2023.1070957 (PMC10025308; doi:10.3389/fnbeh.2023.1070957)

GENERALIZATION OF NAVIGATION MEMORY IN HONEYBEES

SUPPLEMENT DATA SHEET 06: PAIRWISE COMPARISON OF BEE GROUPS WRT. HEAT MAPS

Eric Bullinger*
Otto-von-Guericke-Universität Magdeburg
Institut für Automatisierungstechnik
Universitätsplatz 2, 39106 Magdeburg, Germany
eric.bullinger@ovgu.de

Uwe Greggers & Randolf Menzel*
Freie Universität Berlin
Neurobiologie
Königin Luisestr. 1 -3, 14195 Berlin, Germany
menzel@neurobiologie.fu-berlin.de

14 February 2023

Contents

1 Group A vs. Other Groups **2**
1.1 Group A vs. Group B 2
1.2 Group A vs. Group C 2
1.3 Group A vs. Group D 3
1.4 Group A vs. Group E 3
1.5 Group A vs. Group R 4
1.6 Group A vs. Group S 4

2 Group B vs. Other Groups **5**
2.1 Group B vs. Group C 5
2.2 Group B vs. Group D 5
2.3 Group B vs. Group E 6
2.4 Group B vs. Group R 6
2.5 Group B vs. Group S 7

3 Group C vs. Other Groups **8**
3.1 Group C vs. Group D 8
3.2 Group C vs. Group E 8
3.3 Group C vs. Group R 9
3.4 Group C vs. Group S 9

4 Group D vs. Other Groups **10**
4.1 Group D vs. Group E 10
4.2 Group D vs. Group R 10
4.3 Group D vs. Group S 11

5 Group E vs. Other Groups **12**
5.1 Group E vs. Group R 12
5.2 Group E vs. Group S 12

6 Group R vs. Other Groups **13**
6.1 Group R vs. Group S 13

*corresponding author

1 Group A vs. Other Groups

1.1 Group A vs. Group B

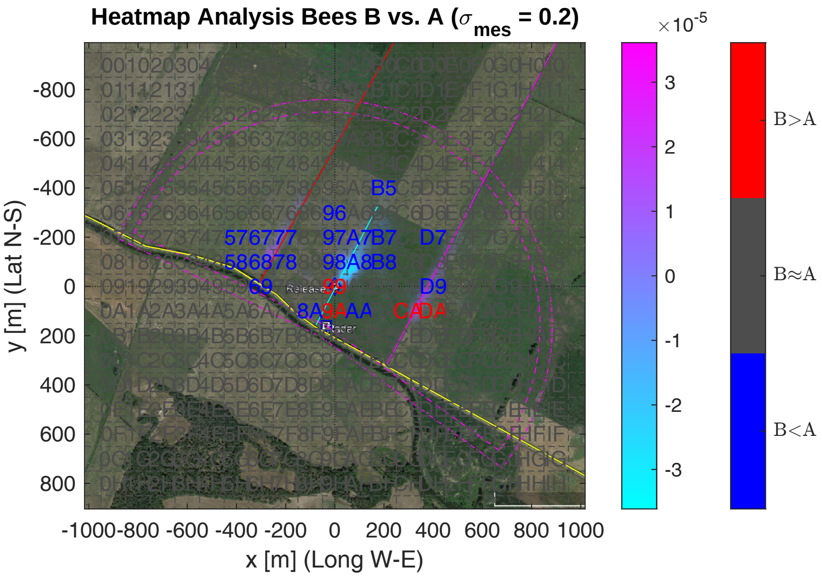

1.2 Group A vs. Group C

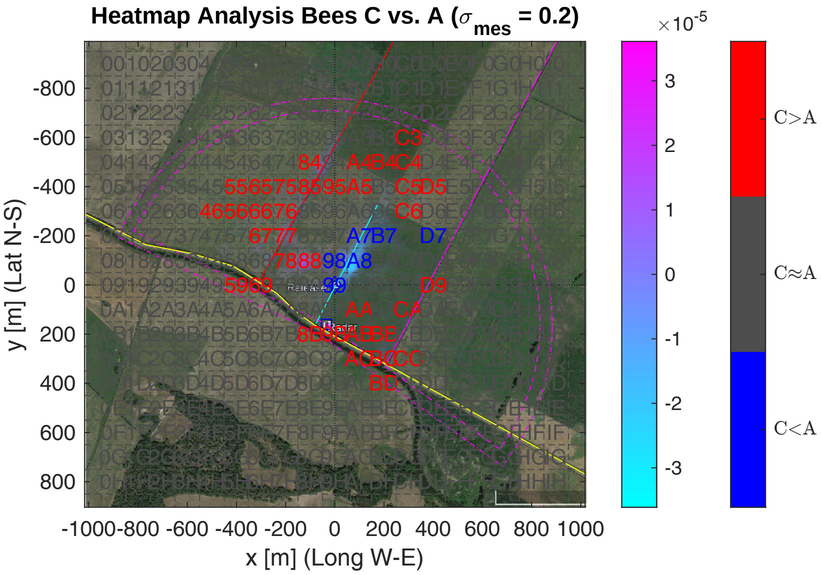

1.3 Group A vs. Group D

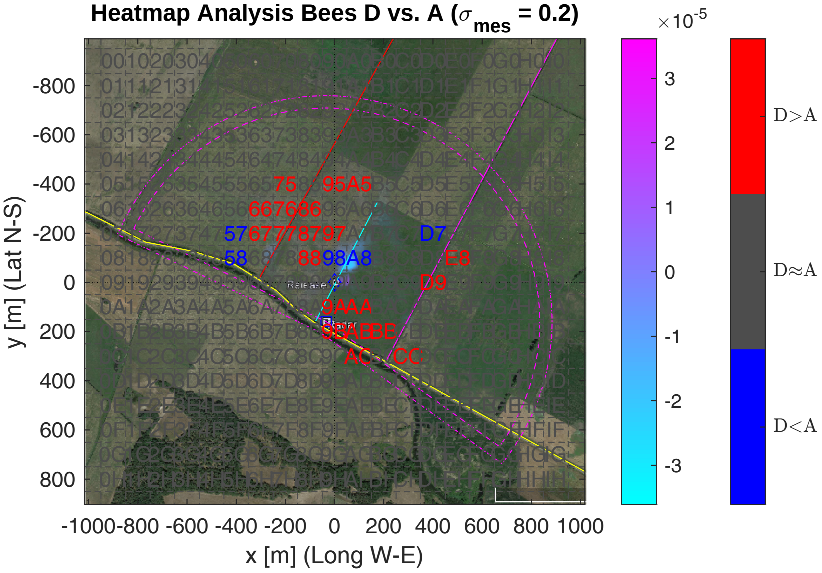

1.4 Group A vs. Group E

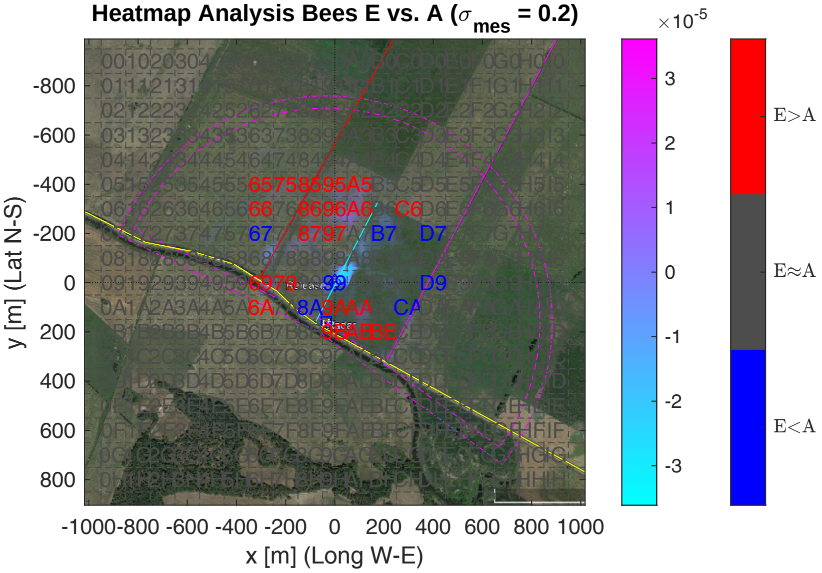

1.5 Group A vs. Group R

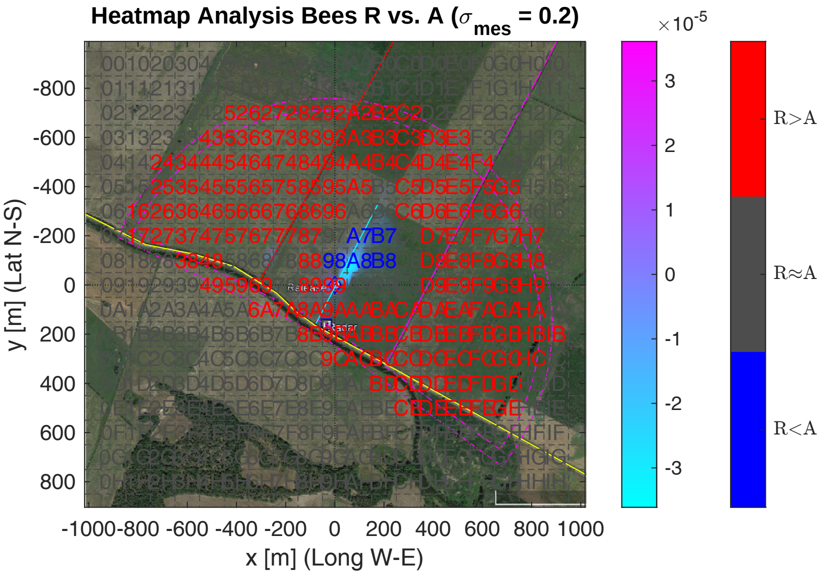

1.6 Group A vs. Group S

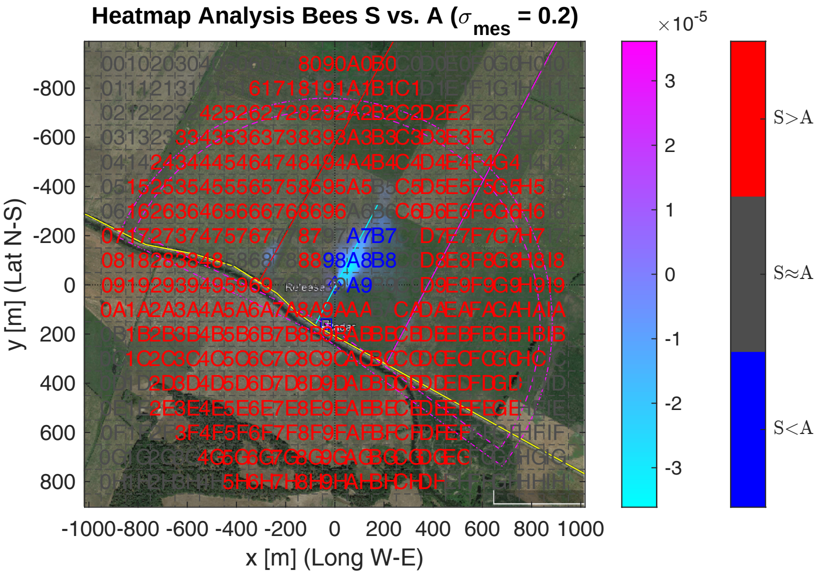

2 Group B vs. Other Groups

2.1 Group B vs. Group C

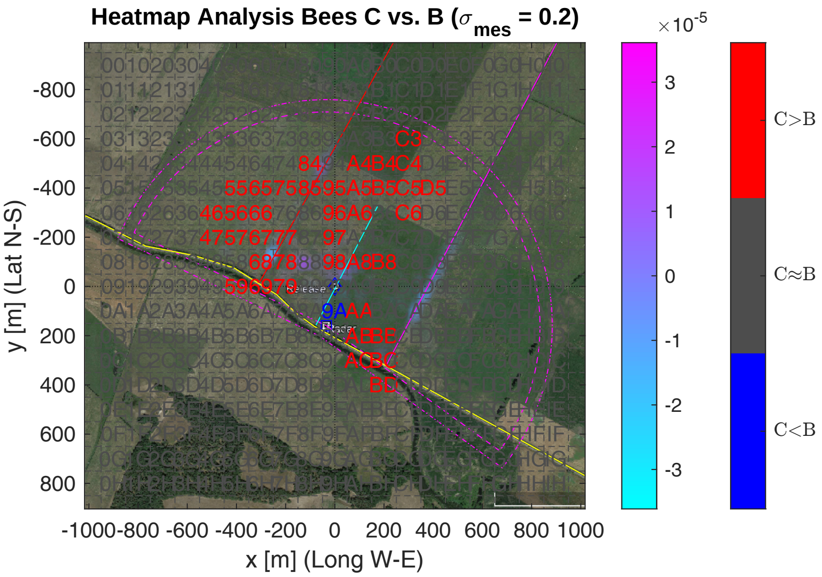

2.2 Group B vs. Group D

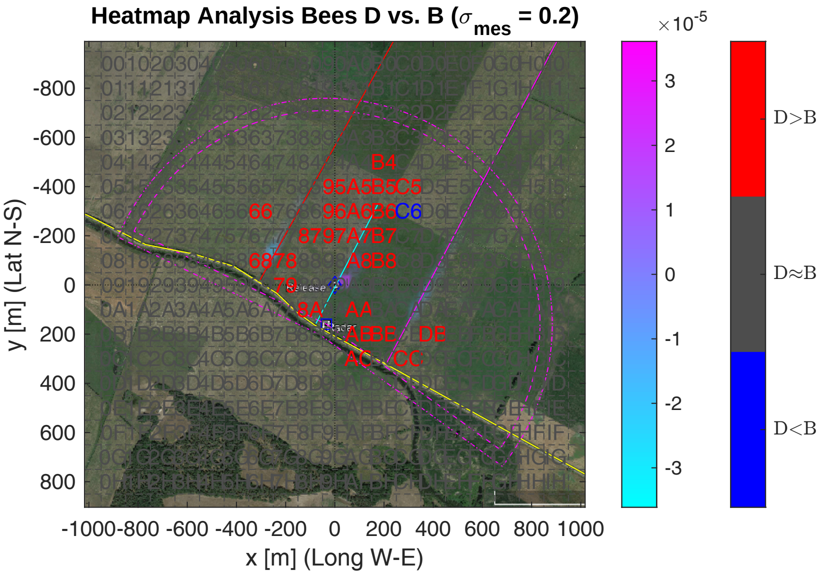

2.3 Group B vs. Group E

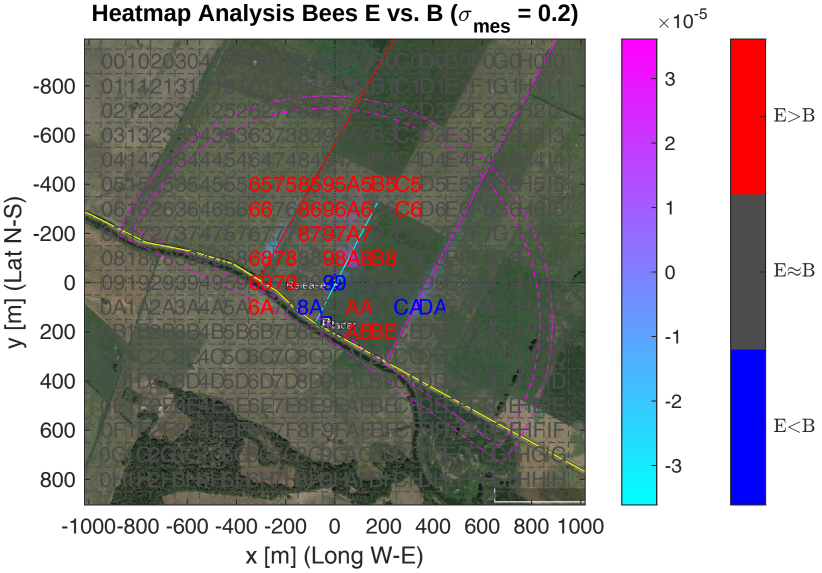

2.4 Group B vs. Group R

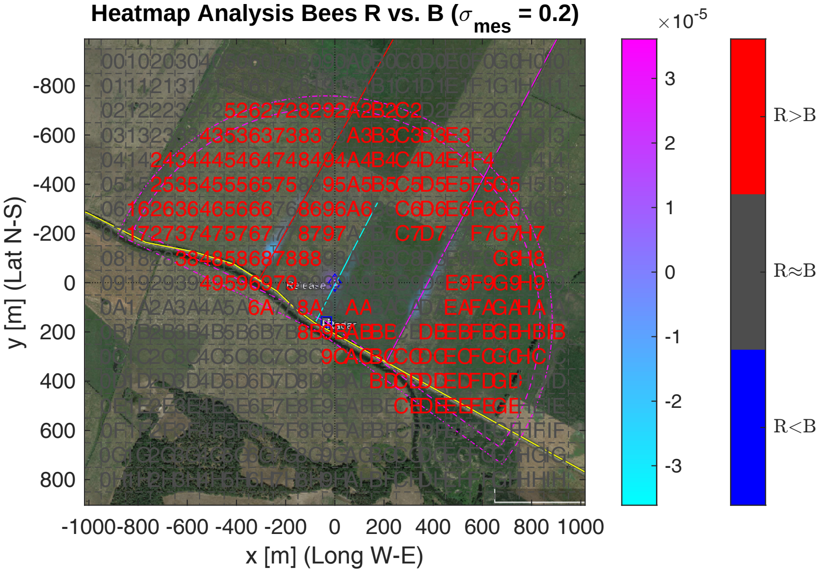

2.5 Group B vs. Group S

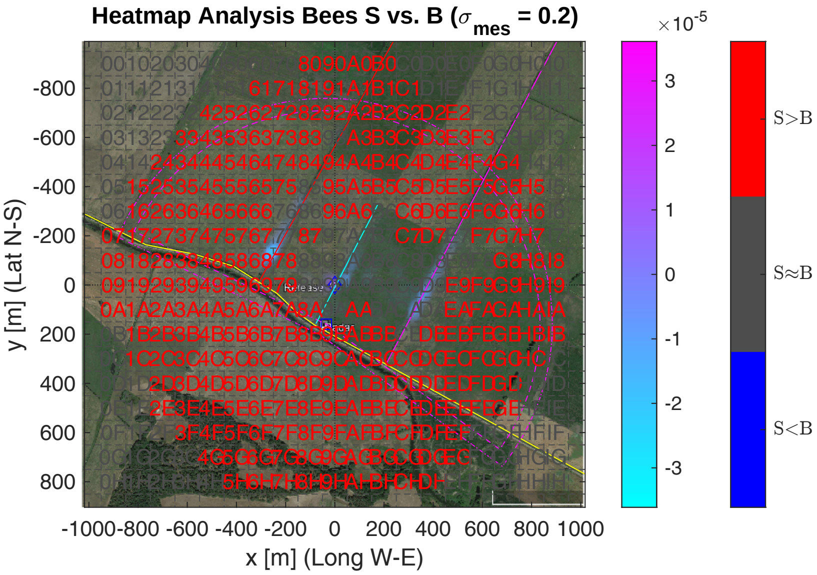

3 Group C vs. Other Groups

3.1 Group C vs. Group D

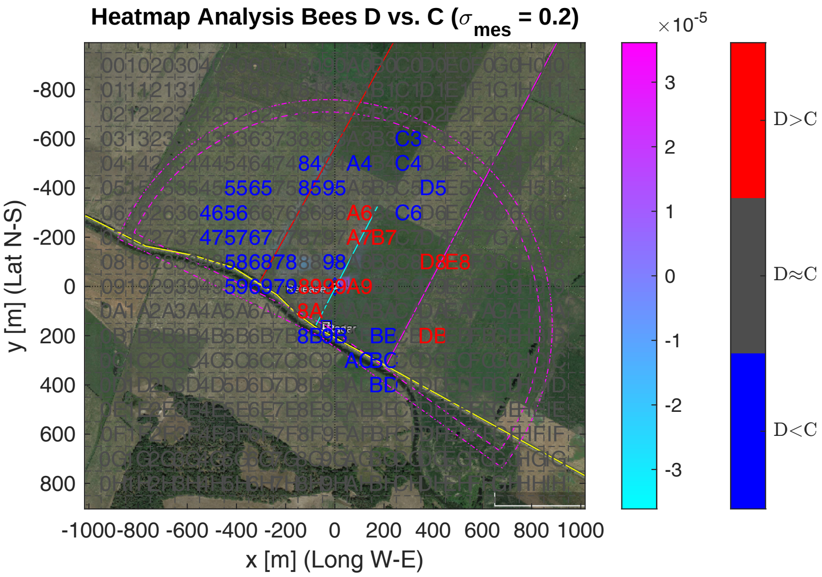

3.2 Group C vs. Group E

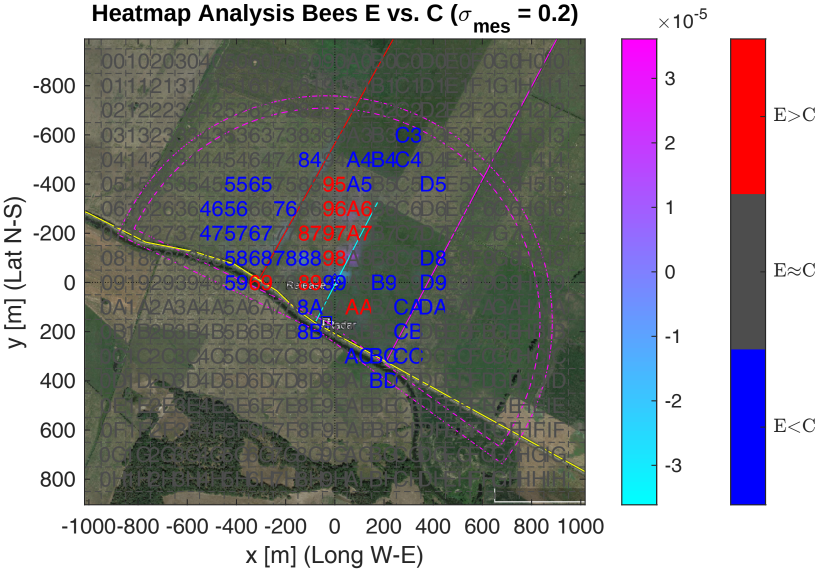

3.3 Group C vs. Group R

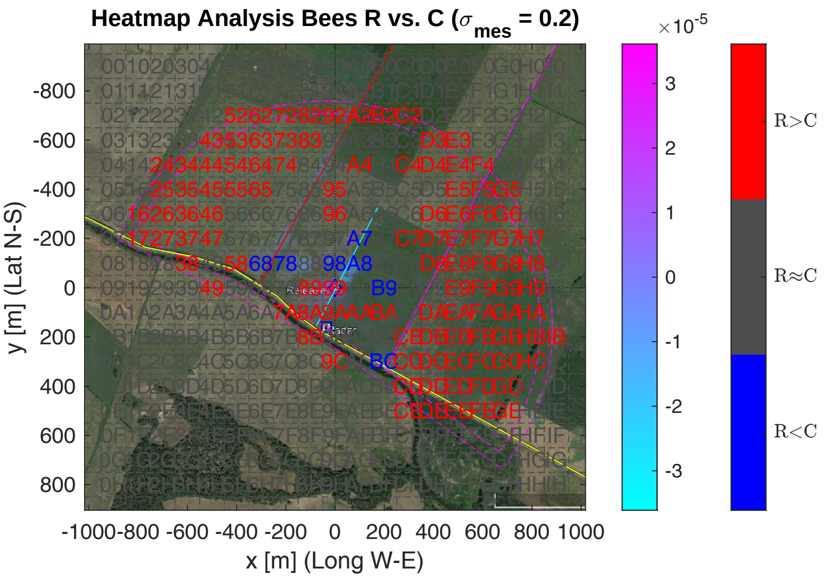

3.4 Group C vs. Group S

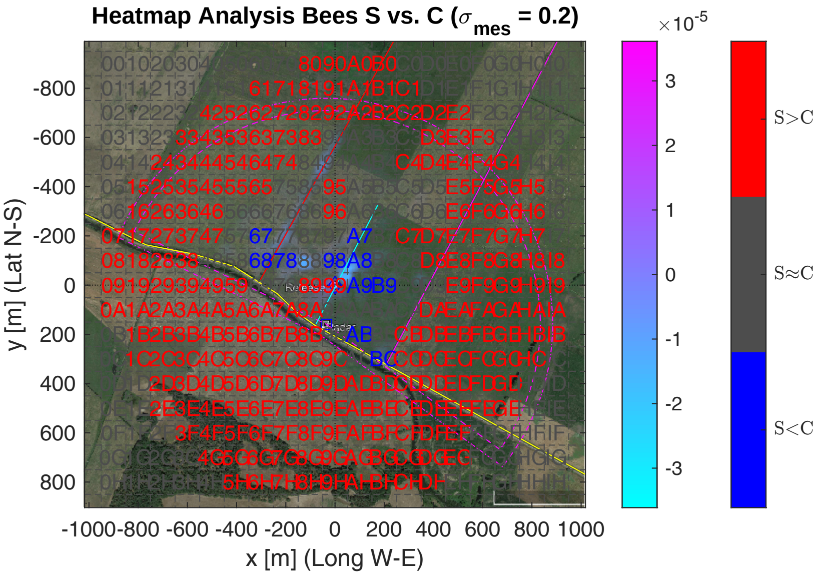

4 Group D vs. Other Groups

4.1 Group D vs. Group E

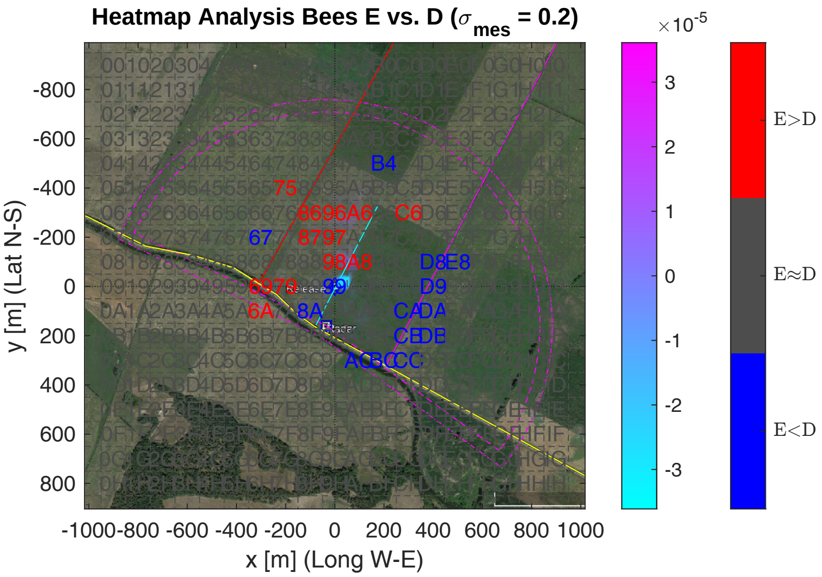

4.2 Group D vs. Group R

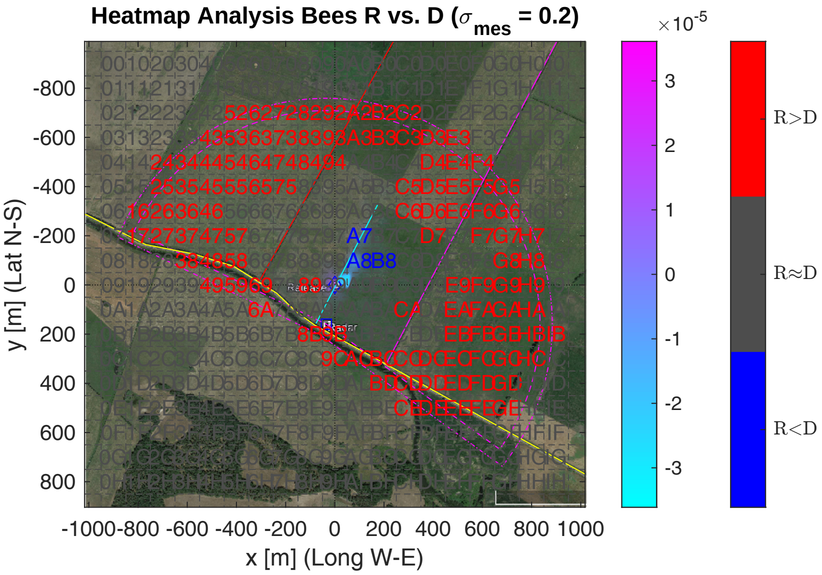

4.3 Group D vs. Group S

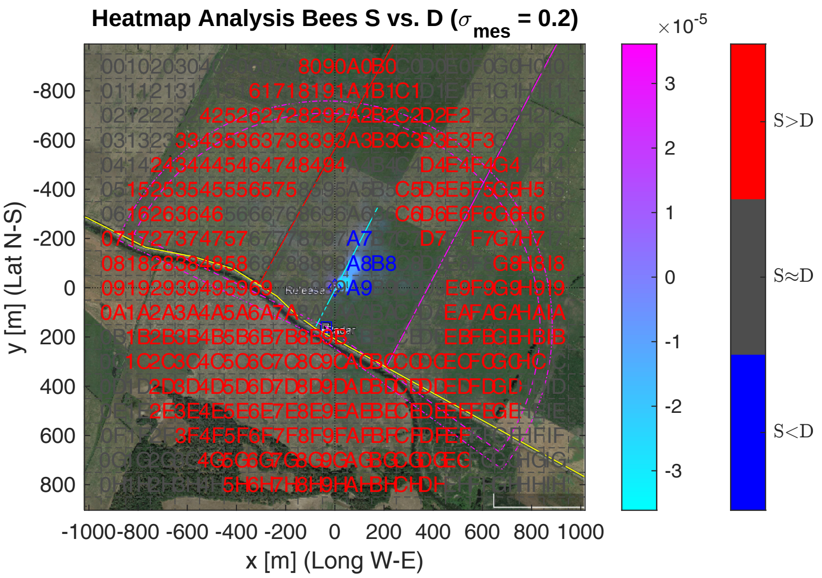

5 Group E vs. Other Groups

5.1 Group E vs. Group R

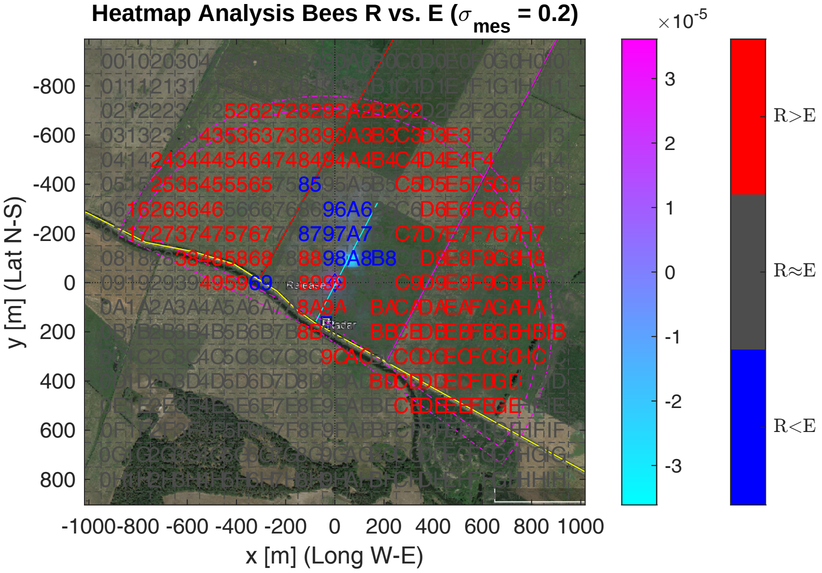

5.2 Group E vs. Group S

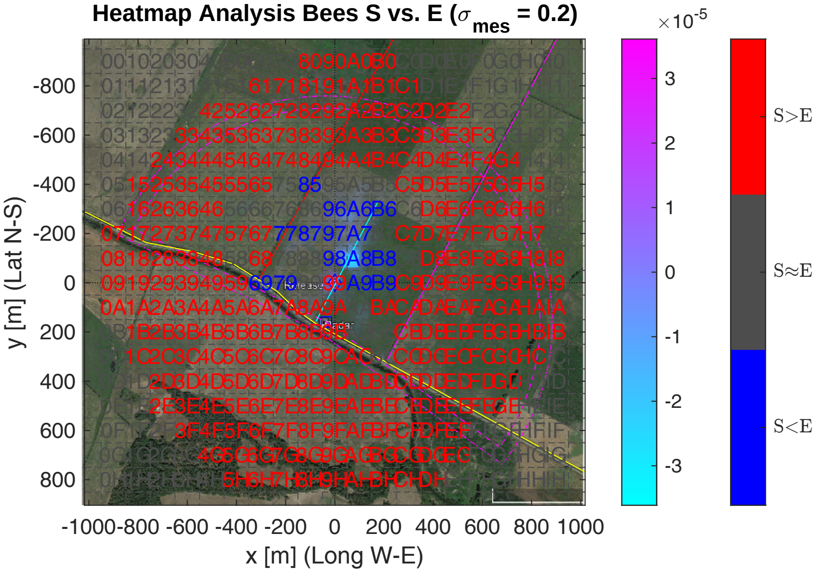

6 Group R vs. Other Groups

6.1 Group R vs. Group S

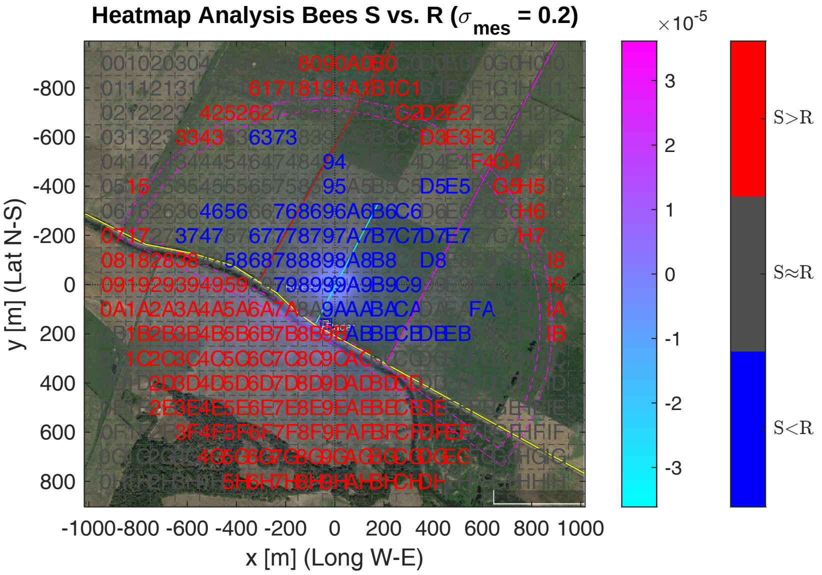

Supplement: Supplementary Data Sheet S6 — Pairwise comparison of bee groups wrt. heat maps. [file Data_Sheet_6.pdf]
