# Supplementary material for: Generalization of navigation memory in honeybees
Source: Front Behav Neurosci. 2023 Mar 6;17:1070957. doi: 10.3389/fnbeh.2023.1070957 (PMC10025308; doi:10.3389/fnbeh.2023.1070957)

Generalization of Navigation Memory in Honeybees

Supplement Data Sheet 07: Significant Tiles for Comparing One Bee Group to All Others

Eric Bullinger*
Otto-von-Guericke-Universität Magdeburg
Institut für Automatisierungstechnik
Universitätsplatz 2, 39106 Magdeburg, Germany
eric.bullinger@ovgu.de

Uwe Greggers & Randolf Menzel*
Freie Universität Berlin
Neurobiologie
Königin Luisestr. 1 -3, 14195 Berlin, Germany
menzel@neurobiologie.fu-berlin.de

14 February 2023

Contents

1 Comparison of one Group vs. all other Groups . . . 2
1.1 Group A 2
1.2 Group B 3
1.3 Group C 4
1.4 Group D 5
1.5 Group E 6
1.6 Group R 7

*corresponding author

1 Comparison of one Group vs. all other Groups

1.1 Group A

With Group S

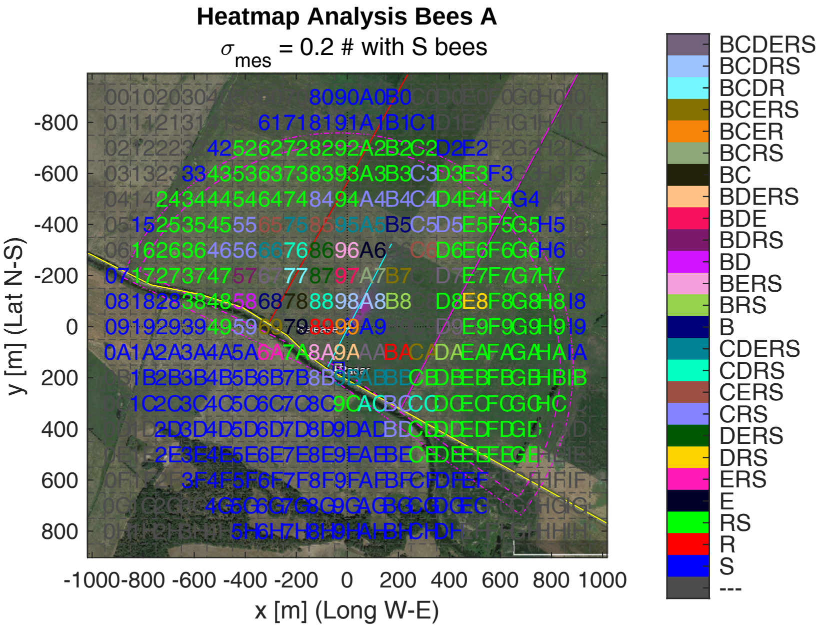

Without Group S

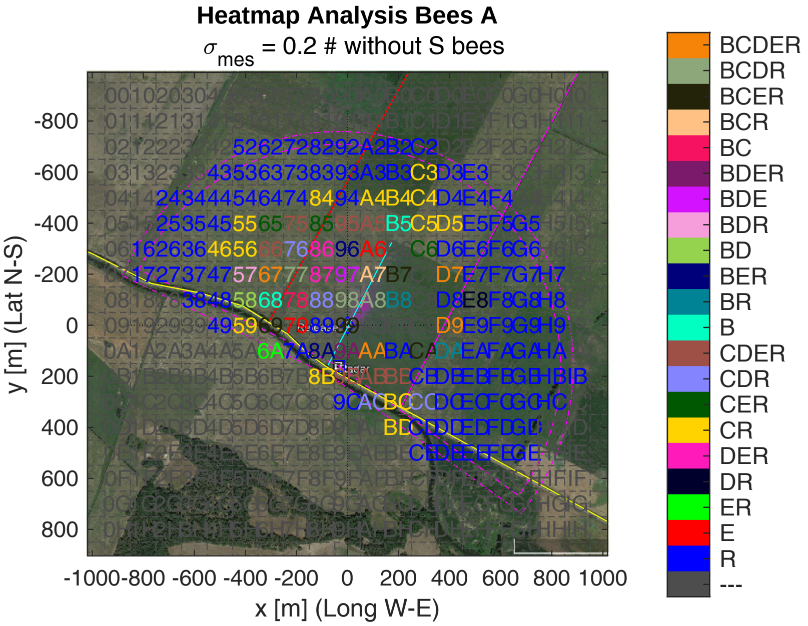

1.2 Group B

With Group S

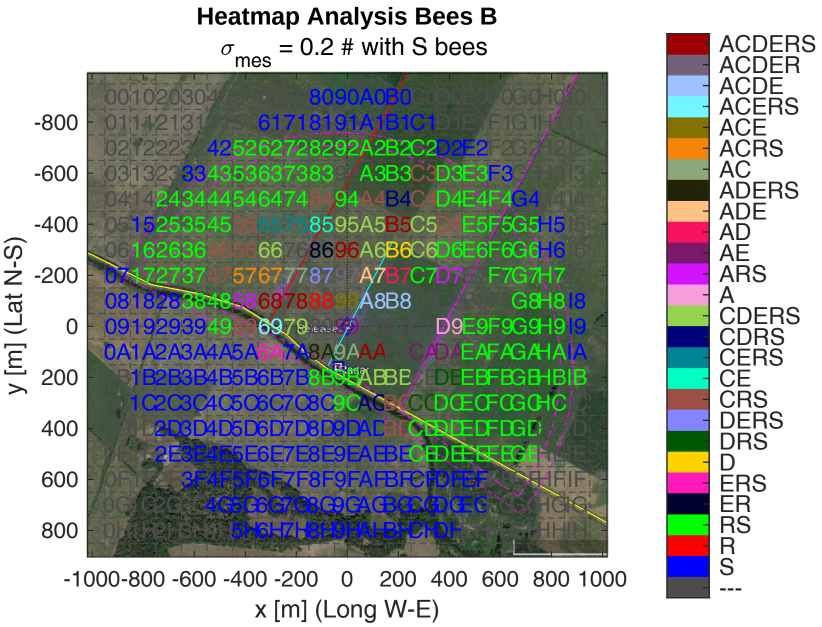

Without Group S

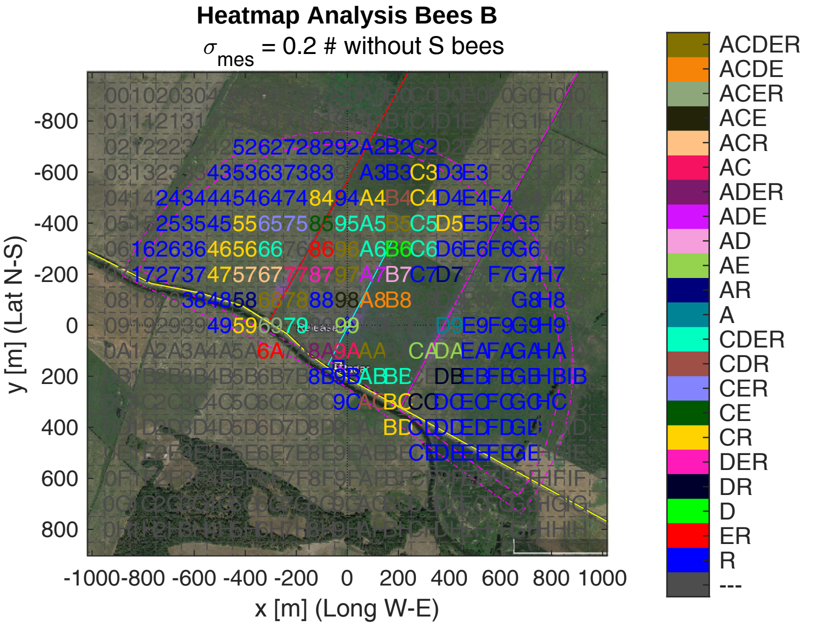

1.3 Group C

With Group S

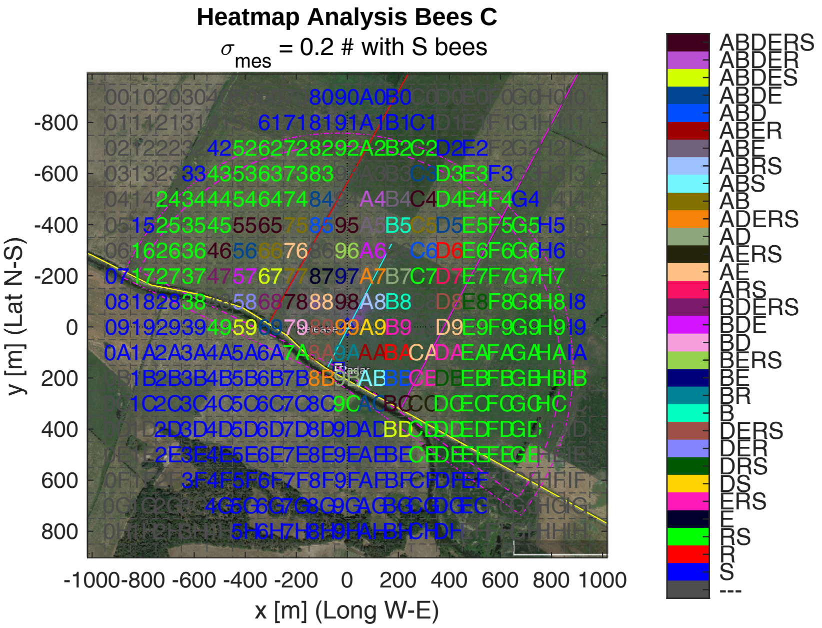

Without Group S

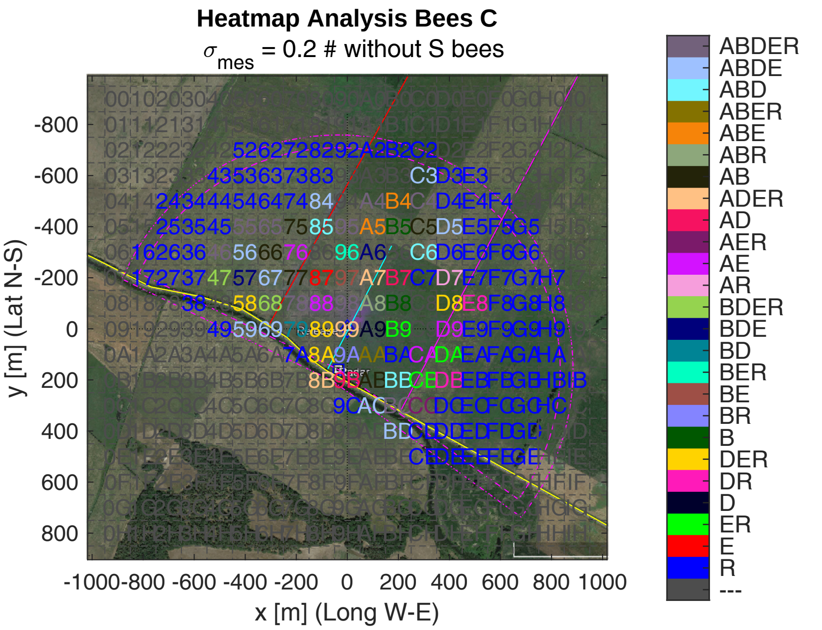

1.4 Group D

With Group S

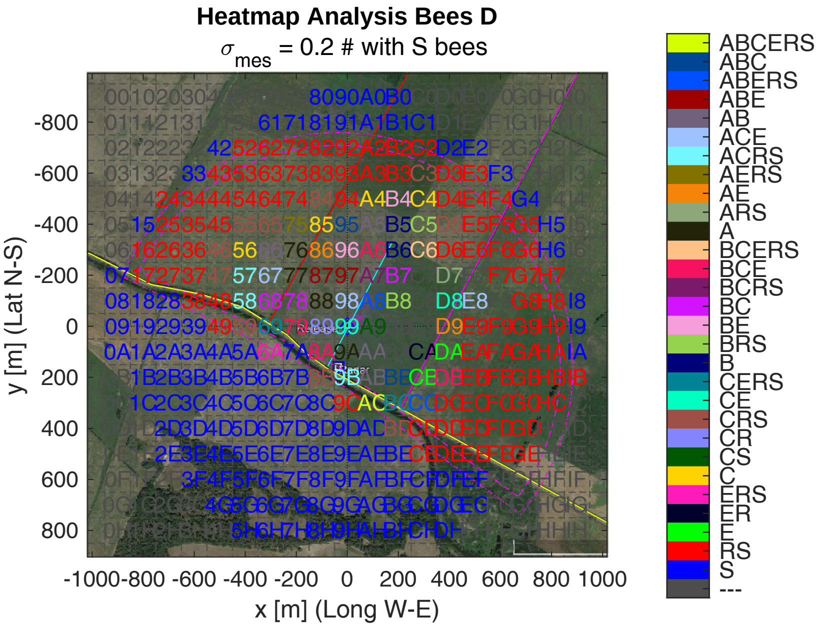

Without Group S

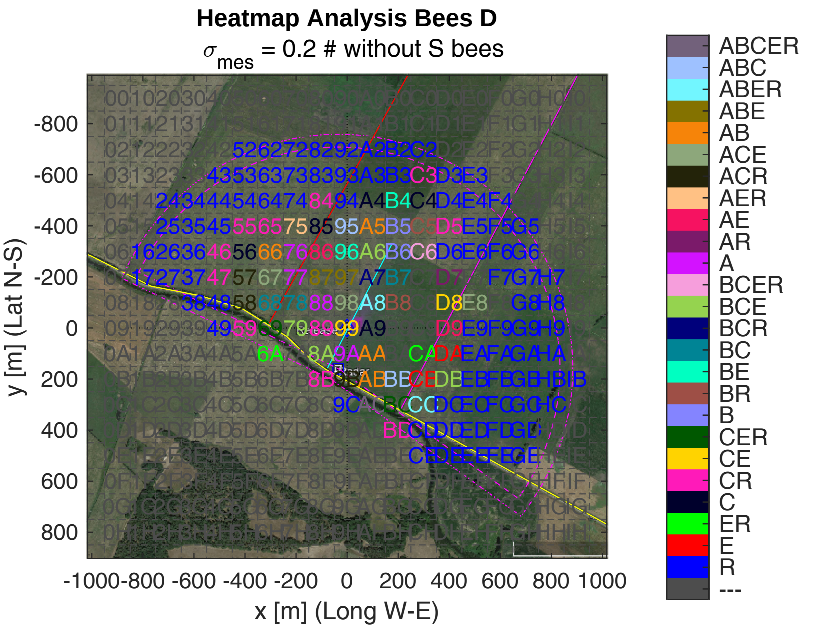

1.5 Group E

With Group S

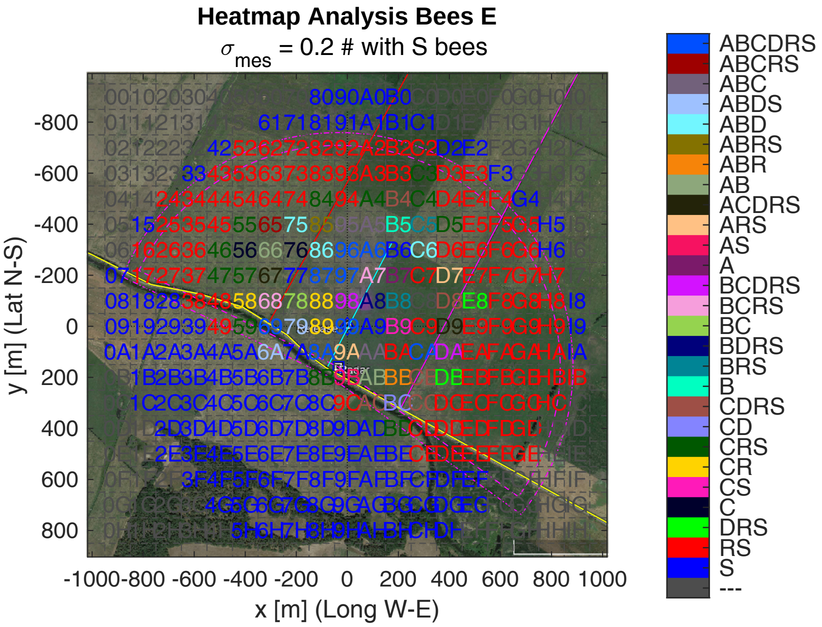

Without Group S

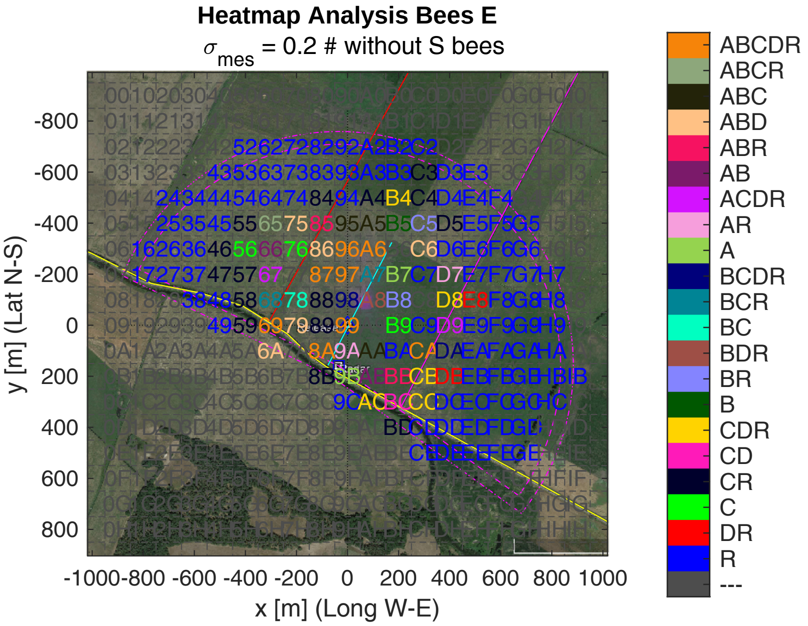

1.6 Group R

With Group S

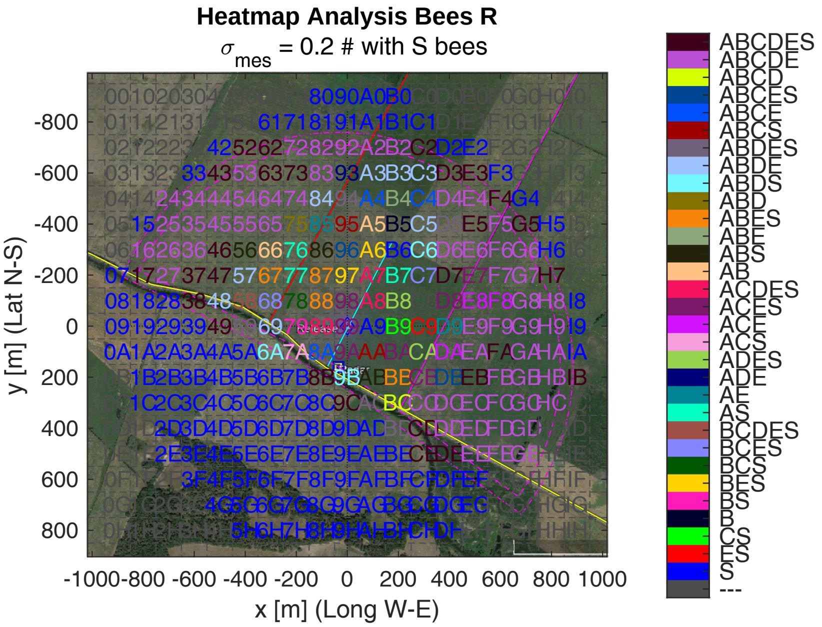

Without Group S

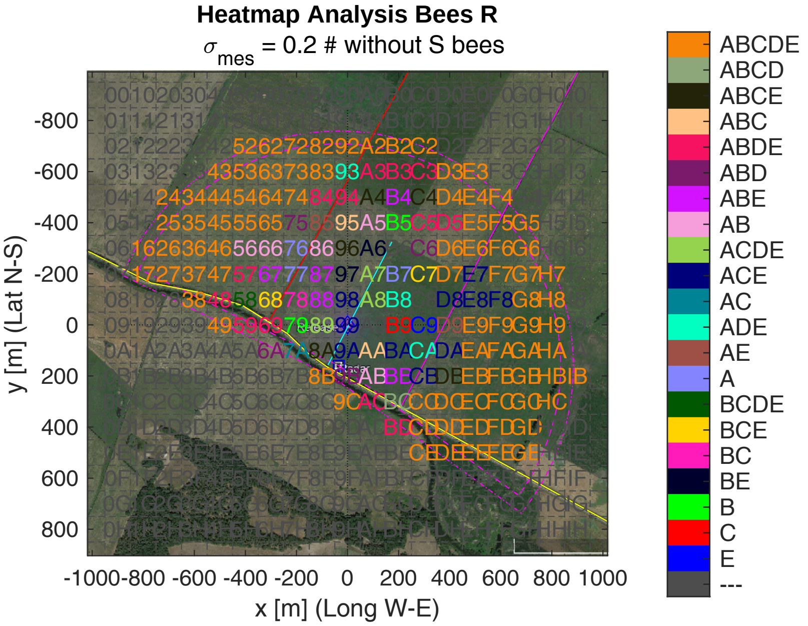

Supplement: Supplementary Data Sheet S7 — Significant tiles for comparing one bee group to all others. [file Data_Sheet_7.pdf]
