# Supplementary material for: Generalization of navigation memory in honeybees
Source: Front Behav Neurosci. 2023 Mar 6;17:1070957. doi: 10.3389/fnbeh.2023.1070957 (PMC10025308; doi:10.3389/fnbeh.2023.1070957)

Generalization of Navigation Memory in Honeybees

Supplement Data Sheet 09: PLS Results as Heat Maps

Eric Bullinger*
Otto-von-Guericke-Universität Magdeburg
Institut für Automatisierungstechnik
Universitätsplatz 2, 39106 Magdeburg, Germany
eric.bullinger@ovgu.de

Uwe Greggers & Randolf Menzel*
Freie Universität Berlin
Neurobiologie
Königin Luisestr. 1 -3, 14195 Berlin, Germany
menzel@neurobiologie.fu-berlin.de

14 February 2023

Contents

1 PLS **2**
1.1 Case with S 2
1.2 Case without S 3
1.3 Case without RS 4

*corresponding author

1 PLS

1.1 Case with S

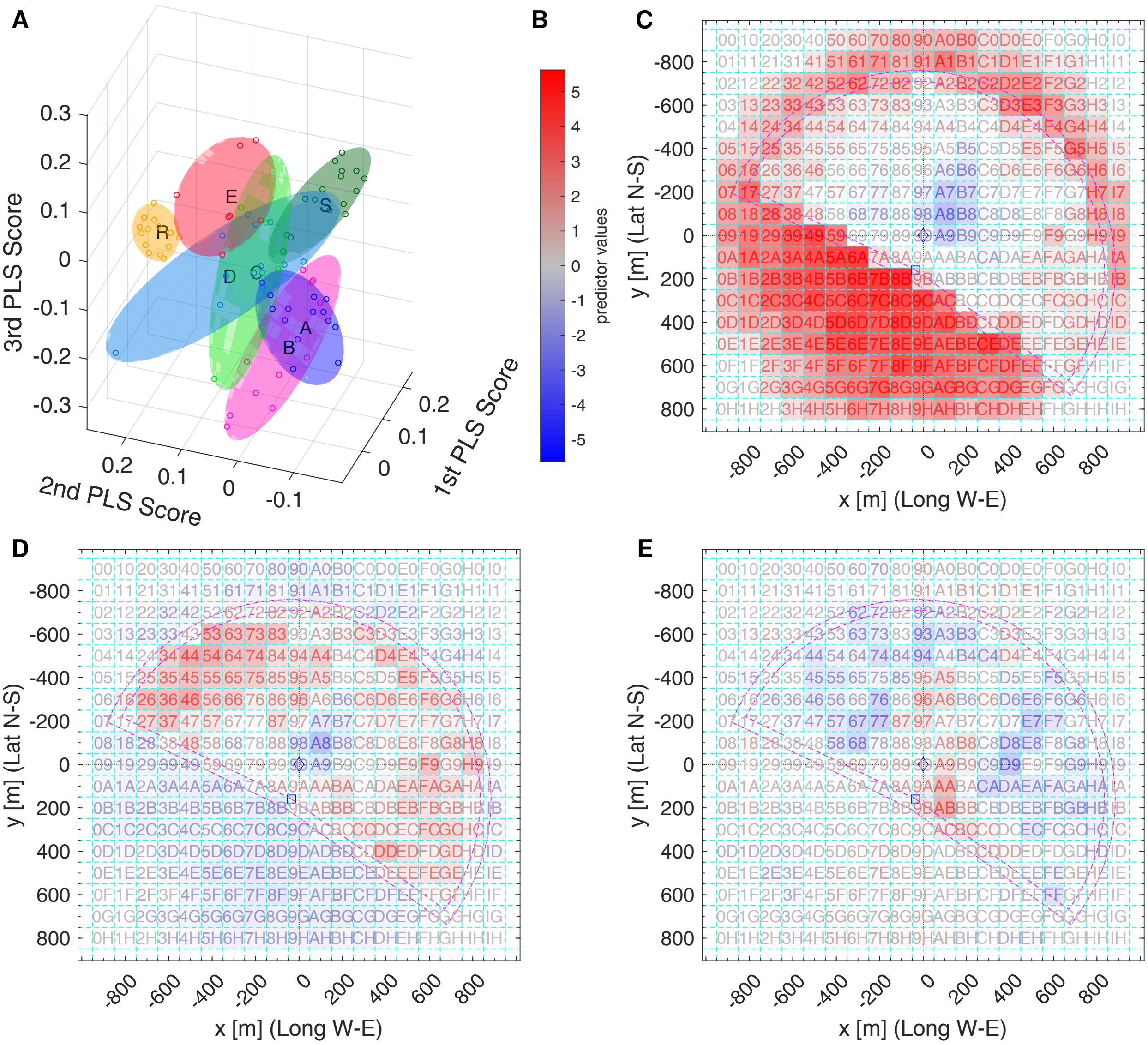

1.2 Case without S

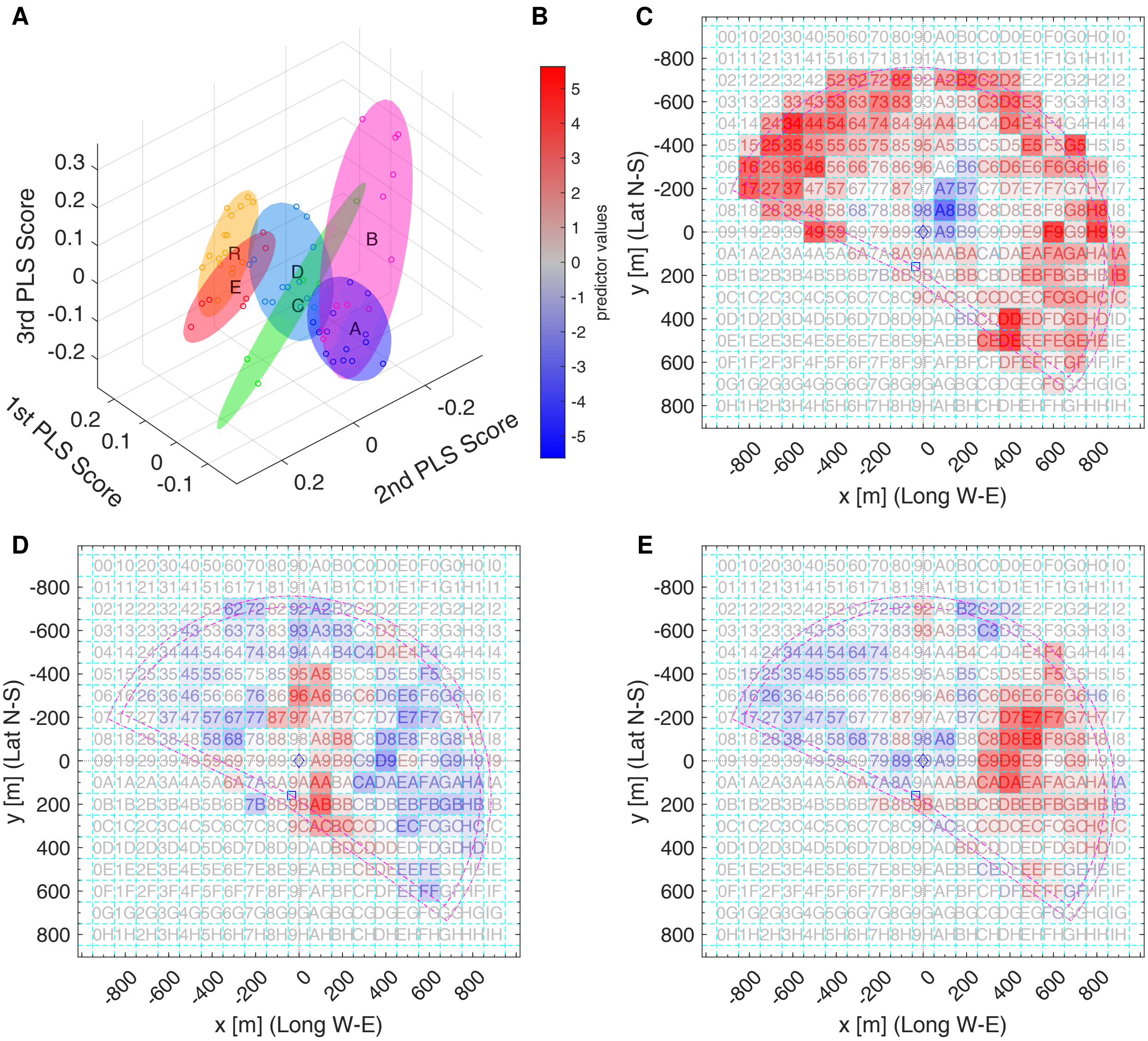

1.3 Case without RS

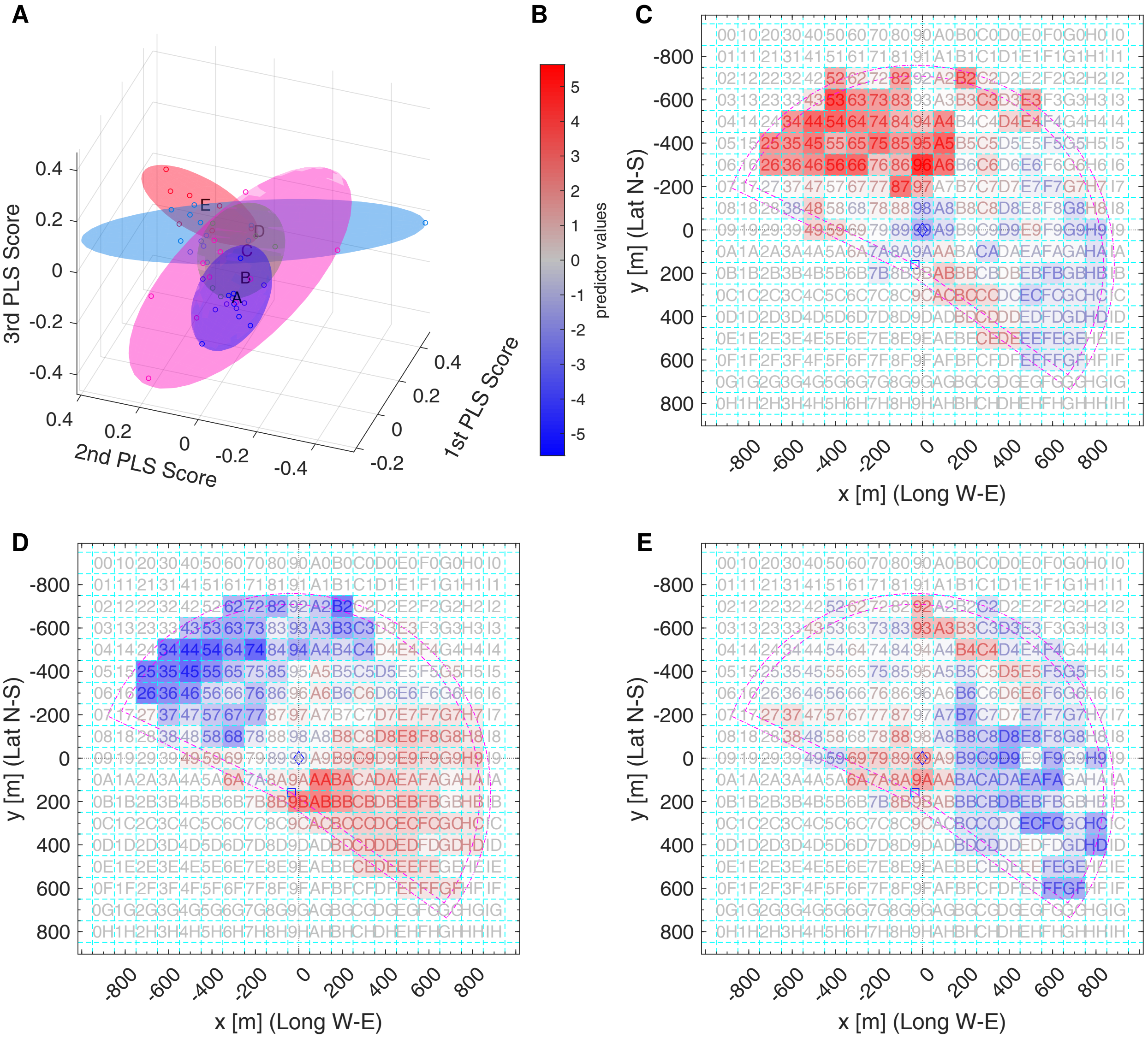

Supplement: Supplementary Data Sheet S9 — PLS results as heat maps. [file Data_Sheet_9.pdf]
