# Supplementary material for: Generalization of navigation memory in honeybees
Source: Front Behav Neurosci. 2023 Mar 6;17:1070957. doi: 10.3389/fnbeh.2023.1070957 (PMC10025308; doi:10.3389/fnbeh.2023.1070957)

GENERALIZATION OF NAVIGATION MEMORY IN HONEYBEES

SUPPLEMENT DATA SHEET10: PLS SEPARATRICES AS HEAT MAPS

Eric Bullinger*
Otto-von-Guericke-Universität Magdeburg
Institut für Automatisierungstechnik
Universitätsplatz 2, 39106 Magdeburg, Germany
eric.bullinger@ovgu.de

Uwe Greggers & Randolf Menzel*
Freie Universität Berlin
Neurobiologie
Königin Luisestr. 1 -3, 14195 Berlin, Germany
menzel@neurobiologie.fu-berlin.de

14 February 2023

Contents

1 PLS Separatrices with S **2**

1.1 Case 1: Separating not S from S . . . 2

1.2 Case 2: Separating not R and S from R and S . . . 3

1.3 Case 3: Separating R from S . . . 4

2 PLS Separatrices without S **5**

2.1 Case 1: Separating not R from R . . . 5

2.2 Case 2: Separating not E from E . . . 6

2.3 Case 3: Separating A and B from not A and B . . . 7

2.4 Case 4: Separating not E and R from E and R . . . 8

3 PLS Separatrices without R and S **9**

3.1 Case 1: Separating A and B from C and D . . . 9

3.2 Case 2: Separating C and D from E . . . 10

*corresponding author

1 PLS Separatrices with S

1.1 Case 1: Separating not S from S

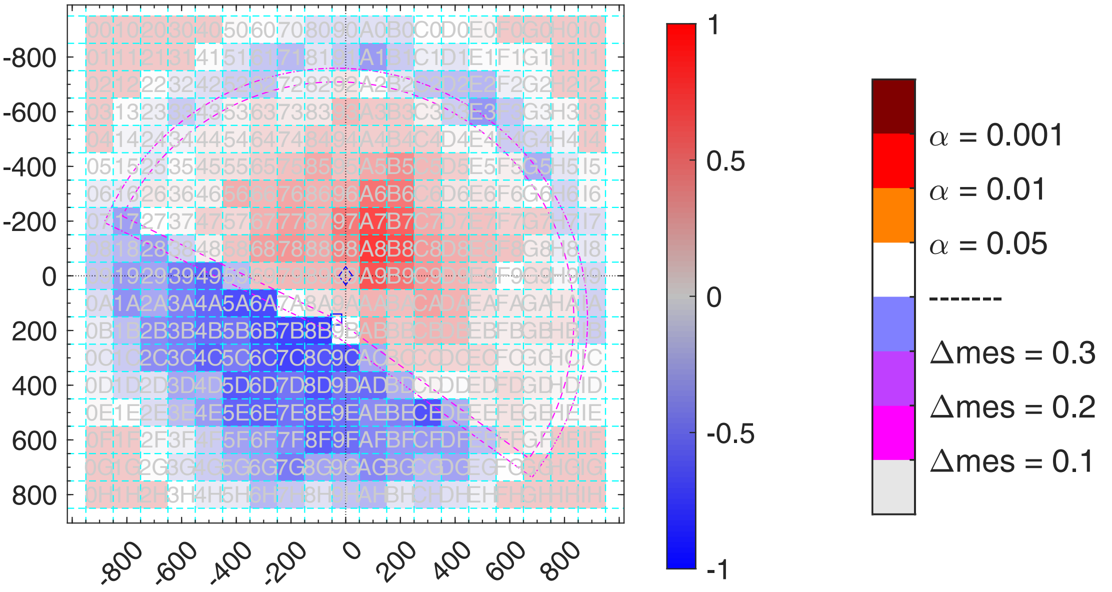

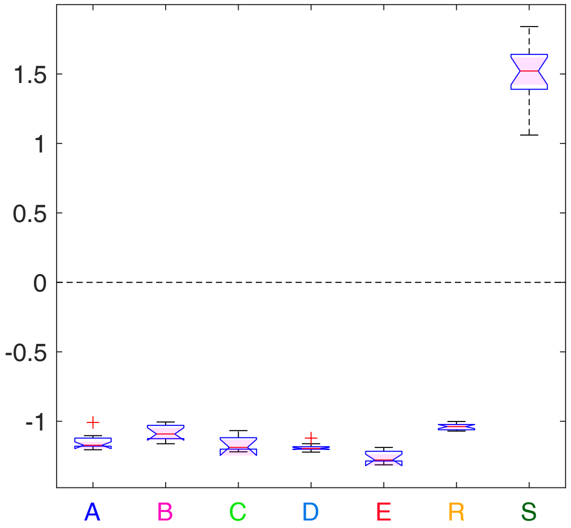

	A	B	C	D	E	R	S
A		7.31e-3		8.78e-3	6.00e-4	<1e-4	<1e-4
B	0.00		4.74e-2	1.17e-4	4.89e-4	2.99e-2	<1e-4
C		0.80			1.85e-2	1.28e-3	9.57e-4
D	0.85	1.00	0.69		6.26e-3	<1e-4	<1e-4
E	1.00	1.00	1.00	0.86		1.83e-4	1.83e-4
R	0.00	0.00	0.00	0.00	0.00		<1e-4
S	0.00	0.00	0.00	0.00	0.00	0.00	

1.2 Case 2: Separating not R and S from R and S

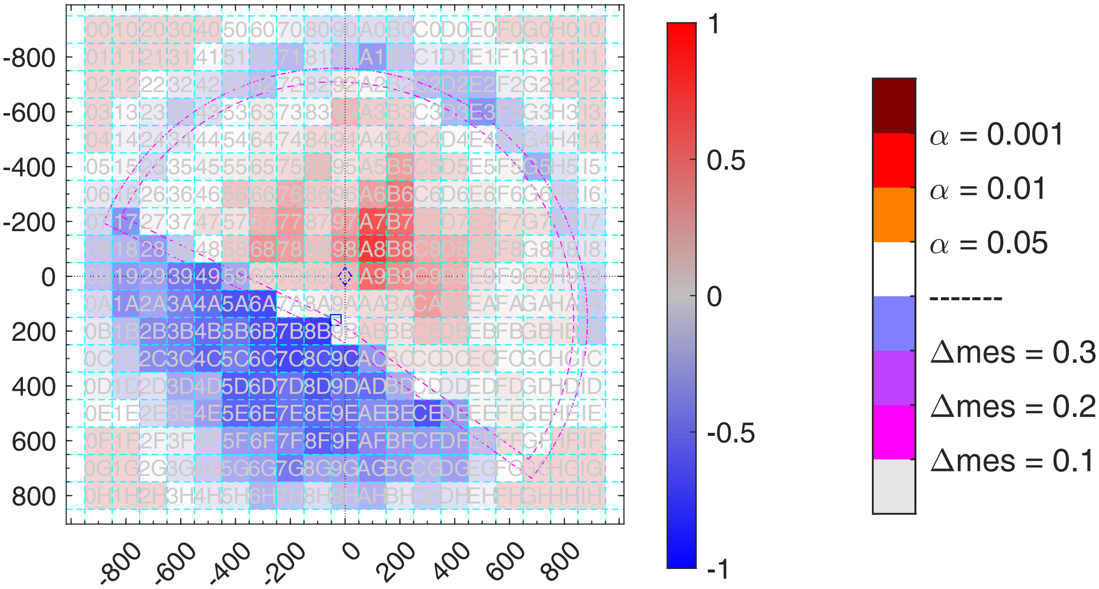

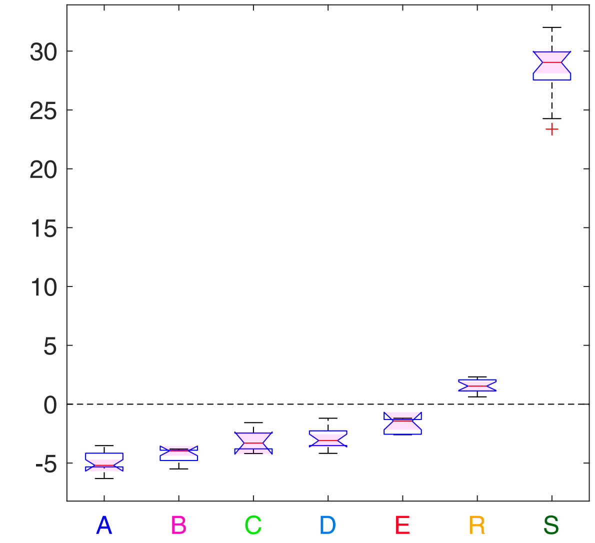

	A	B	C	D	E	R	S
A			9.53e-3	<1e-4	2.57e-4	<1e-4	<1e-4
B	0.18		1.49e-2	1.86e-4	4.89e-4	<1e-4	<1e-4
C	0.00	0.20			1.85e-2	9.57e-4	9.57e-4
D	0.00	0.08	0.38		1.57e-2	<1e-4	<1e-4
E	0.00	0.00	0.00	0.00		1.83e-4	1.83e-4
R	0.00	0.00	0.00	0.00	0.00		<1e-4
S	0.00	0.00	0.00	0.00	0.00	0.00	

1.3 Case 3: Separating R from S

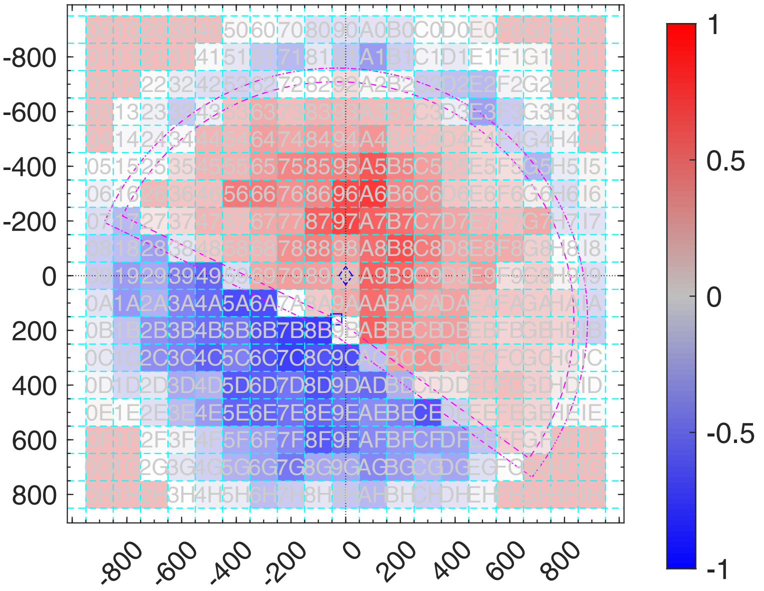

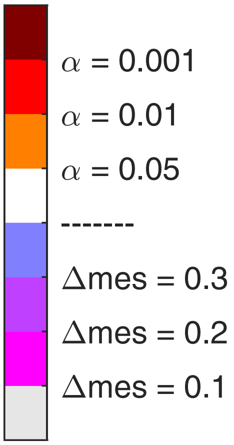

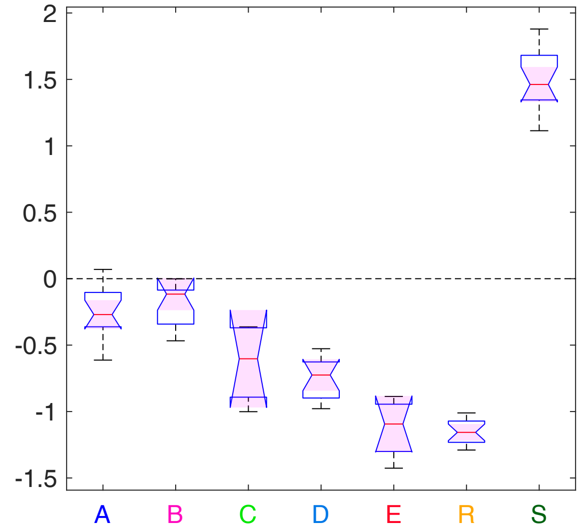

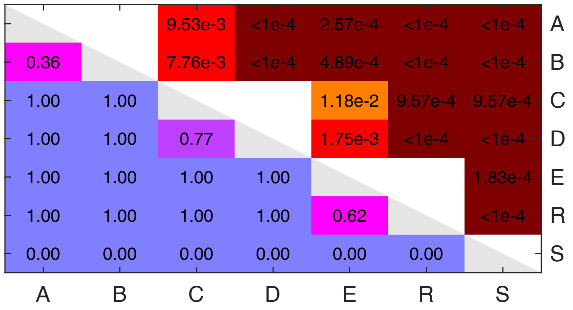

2 PLS Separatrices without S

2.1 Case 1: Separating not R from R

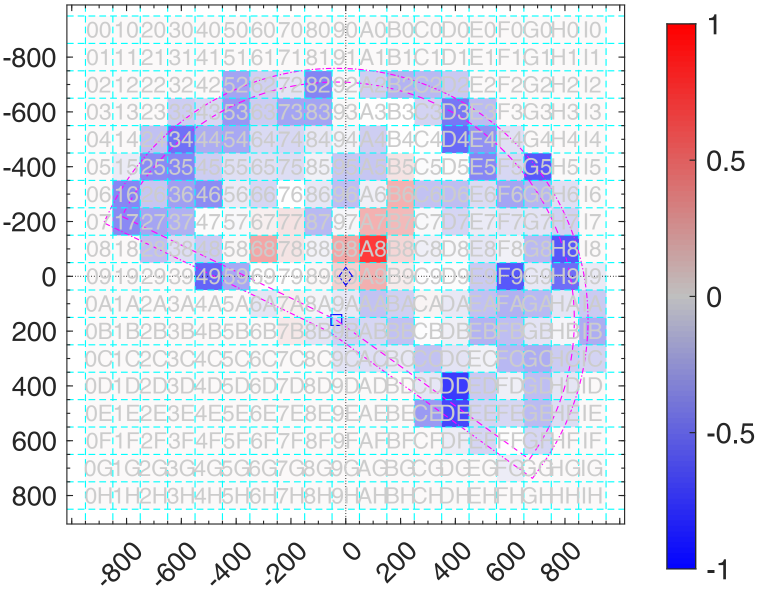

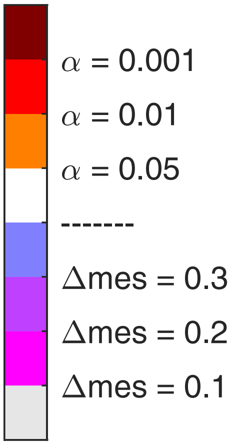

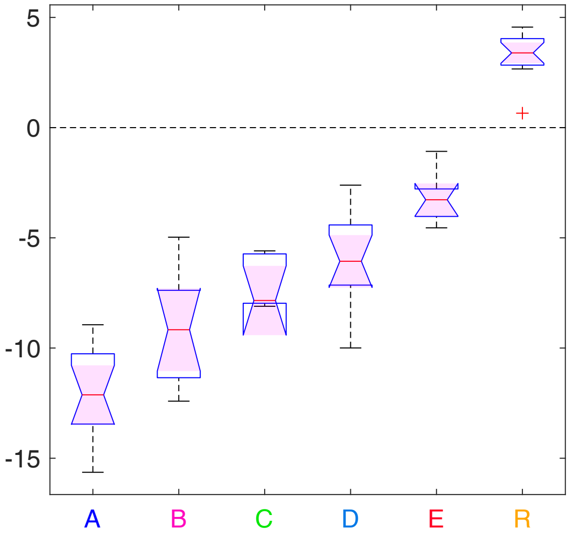

	A	B	C	D	E	R
A		1.18e-2	1.19e-3	<1e-4	2.57e-4	<1e-4
B	0.09			3.44e-3	4.89e-4	<1e-4
C	0.00	0.00			4.48e-3	9.57e-4
D	0.00	0.08	0.15		6.26e-3	<1e-4
E	0.00	0.00	0.00	0.00		1.83e-4
R	0.00	0.00	0.00	0.00	0.00	

2.2 Case 2: Separating not E from E

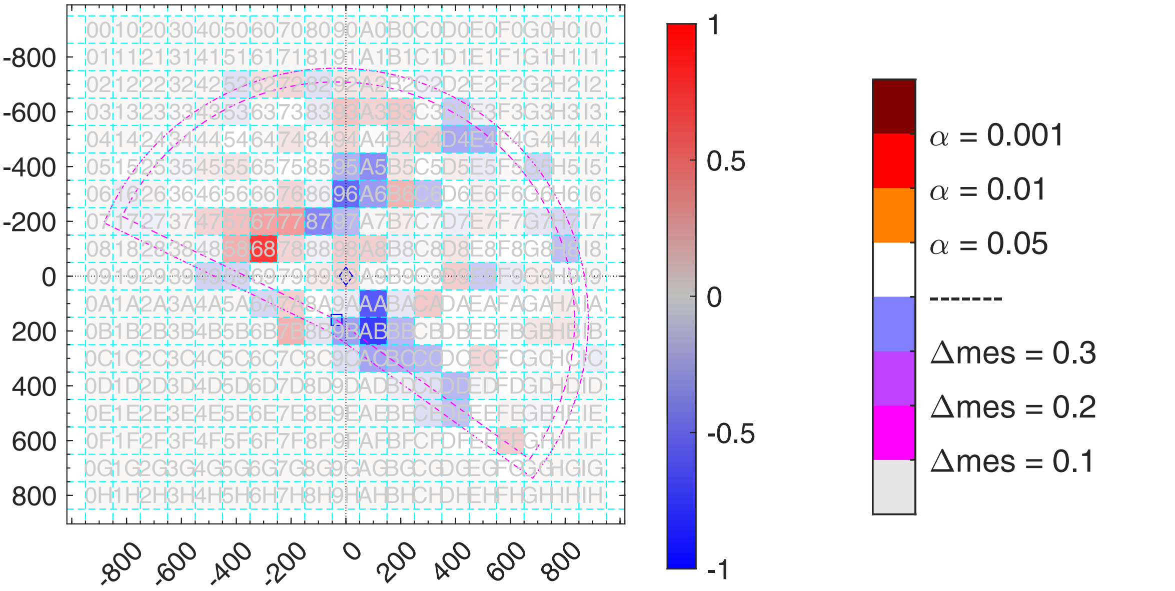

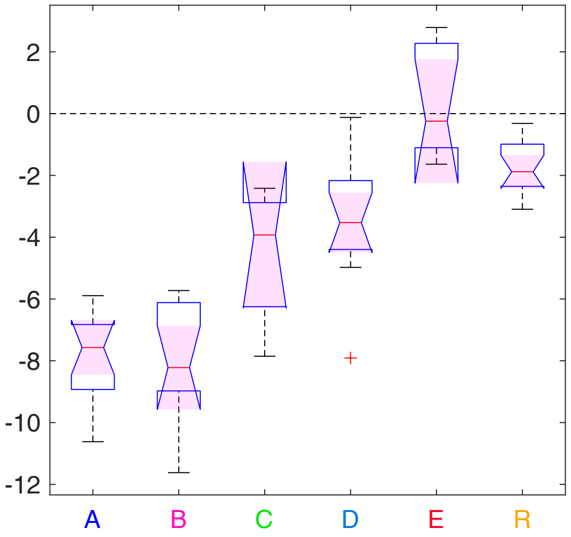

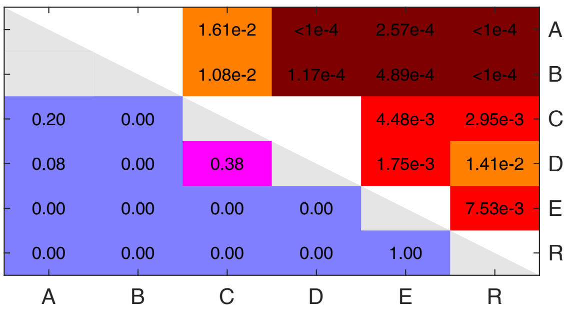

2.3 Case 3: Separating A and B from not A and B

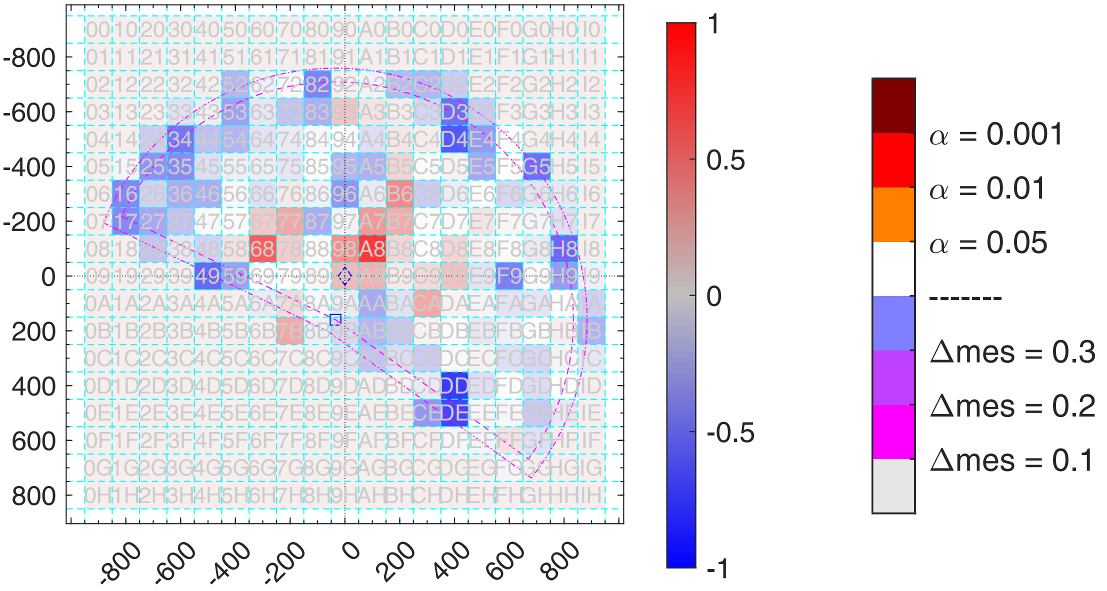

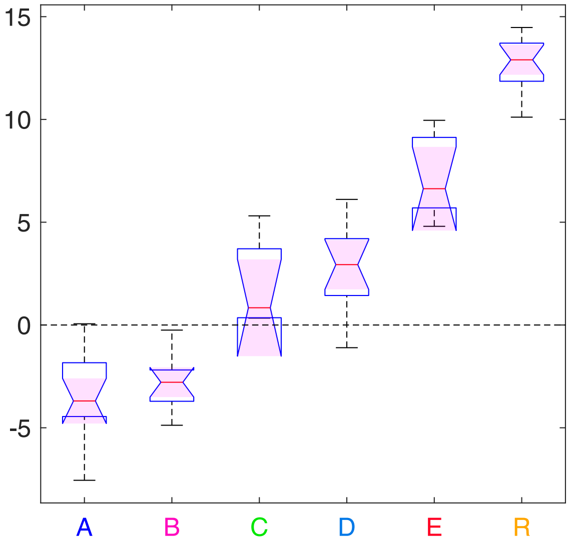

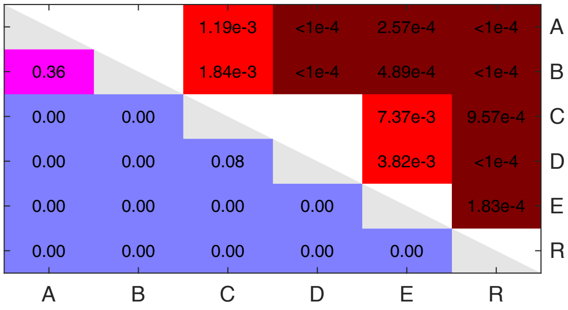

2.4 Case 4: Separating not E and R from E and R

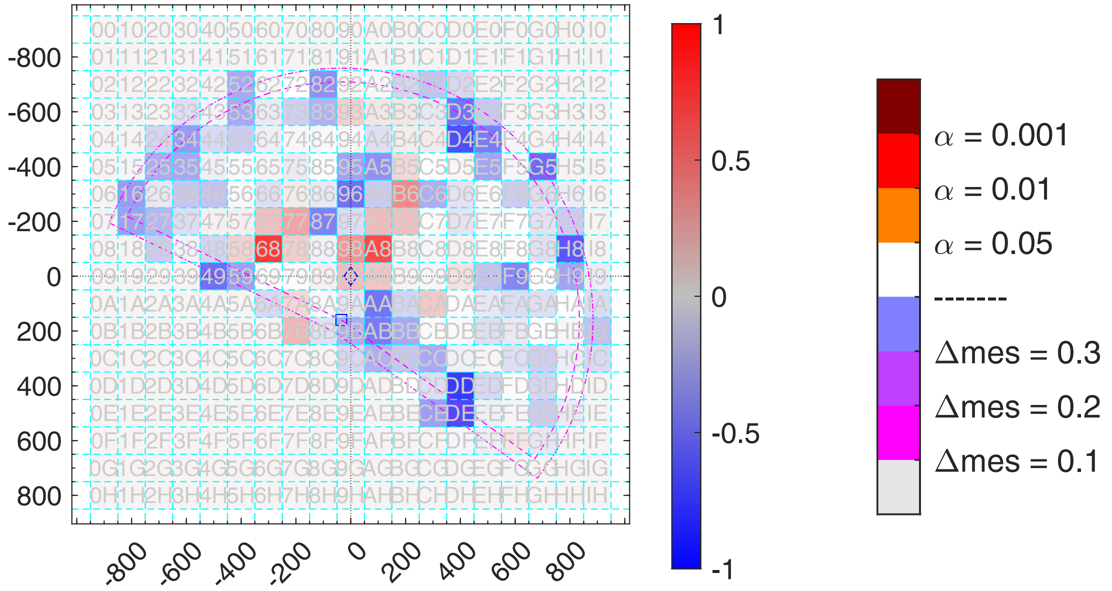

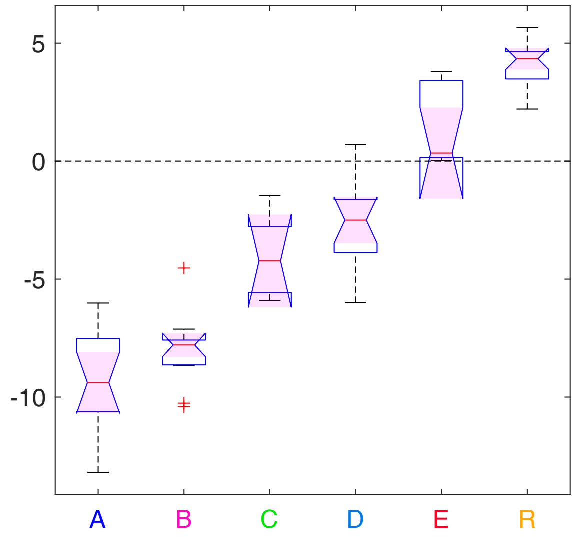

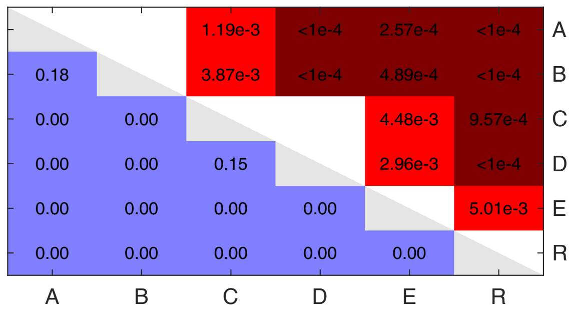

3 PLS Separatrices without R and S

3.1 Case 1: Separating A and B from C and D

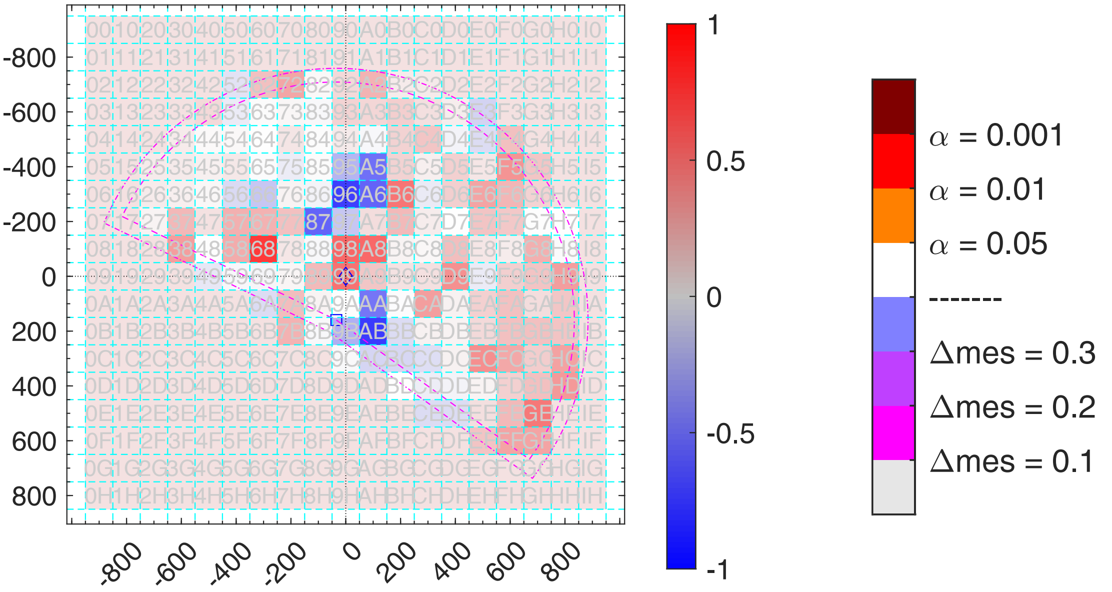

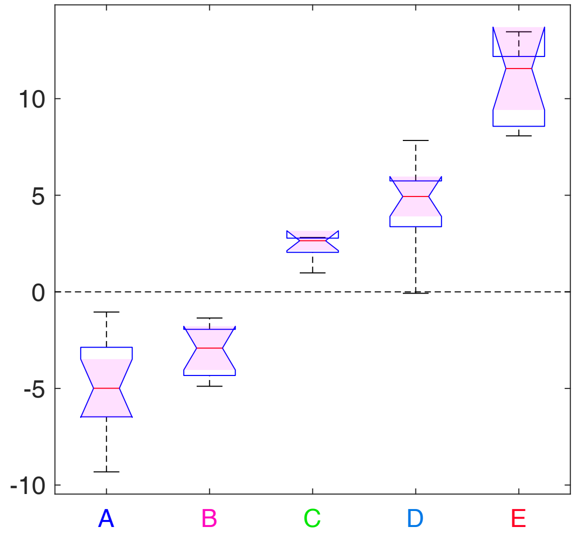

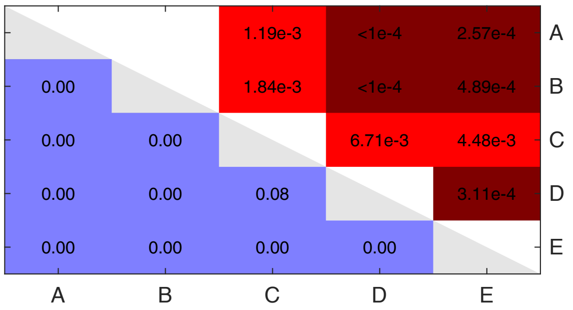

3.2 Case 2: Separating C and D from E

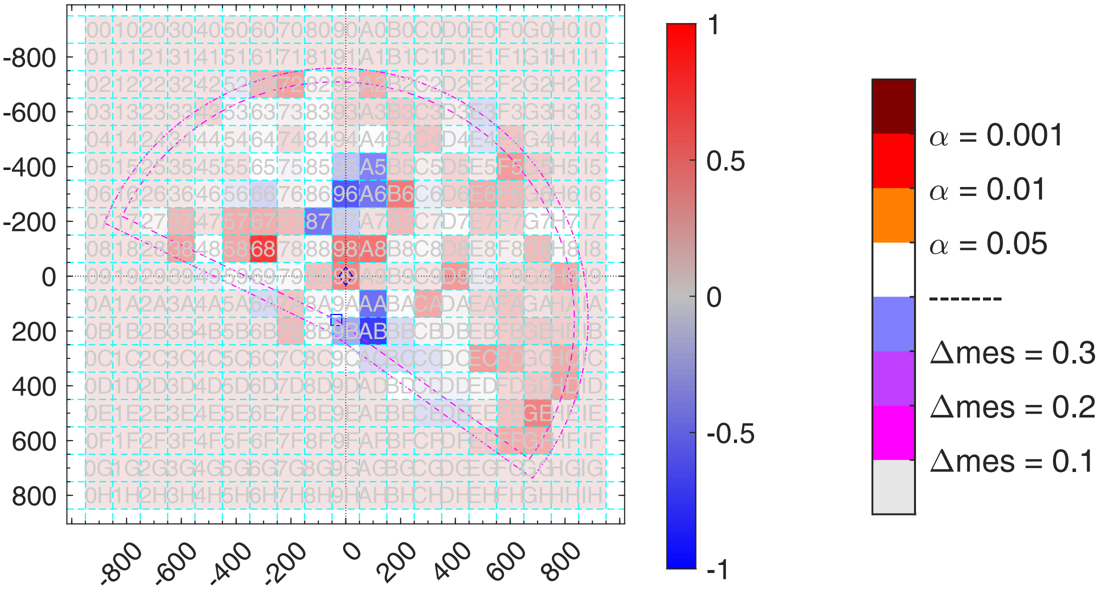

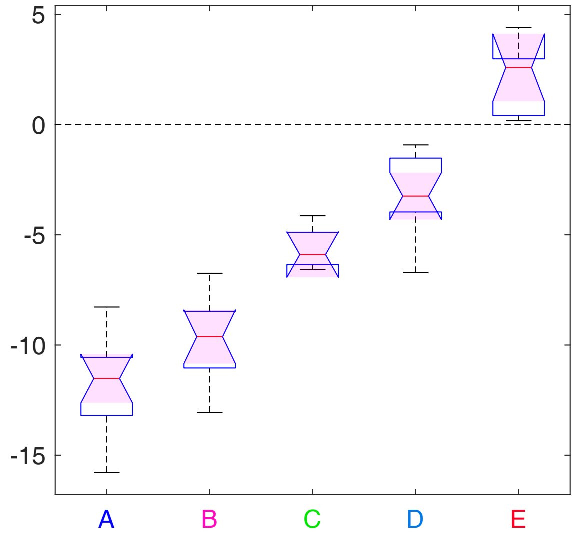

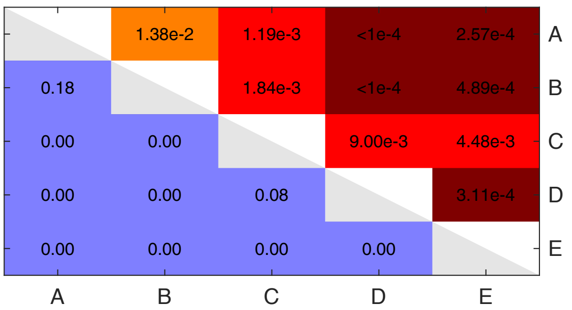

Supplement: Supplementary Data Sheet S10 — PLS separatices as heat maps. [file Data_Sheet_10.pdf]
