# Supplementary material for: Generalization of navigation memory in honeybees
Source: Front Behav Neurosci. 2023 Mar 6;17:1070957. doi: 10.3389/fnbeh.2023.1070957 (PMC10025308; doi:10.3389/fnbeh.2023.1070957)
Supplement: Supplementary Data Sheet S11 — Flight paths near edges. [file Data_Sheet_11.pdf]

Generalization of Navigation Memory in Honeybees

Supplement Data Sheet 11: Flight Paths near Edges

Eric Bullinger*
Otto-von-Guericke-Universität Magdeburg
Institut für Automatisierungstechnik
Universitätsplatz 2, 39106 Magdeburg, Germany
eric.bullinger@ovgu.de

Uwe Greggers & Randolf Menzel*
Freie Universität Berlin
Neurobiologie
Königin Luisestr. 1 -3, 14195 Berlin, Germany
menzel@neurobiologie.fu-berlin.de

14 February 2023

- Bee A01: p. 2
- Bee A02: p. 3
- Bee A03: p. 4
- Bee A04: p. 5
- Bee A05: p. 6
- Bee A06: p. 7
- Bee A07: p. 8
- Bee A08: p. 9
- Bee A09: p. 10
- Bee A10: p. 11
- Bee A11: p. 12
- Bee A12: p. 13
- Bee A13: p. 14
- Bee A14: p. 15
- Bee B01: p. 16
- Bee B02: p. 17
- Bee B03: p. 18
- Bee B04: p. 19
- Bee B05: p. 20
- Bee B06: p. 21
- Bee B07: p. 22
- Bee B08: p. 23
- Bee B09: p. 24
- Bee B10: p. 25
- Bee B11: p. 26
- Bee C01: p. 27
- Bee C02: p. 28
- Bee C03: p. 29
- Bee C04: p. 30
- Bee C05: p. 31
- Bee D01: p. 32
- Bee D02: p. 33
- Bee D03: p. 34
- Bee D04: p. 35
- Bee D05: p. 36
- Bee D06: p. 37
- Bee D07: p. 38
- Bee D08: p. 39
- Bee D09: p. 40
- Bee D10: p. 41
- Bee D11: p. 42
- Bee D12: p. 43
- Bee D13: p. 44
- Bee E01: p. 45
- Bee E02: p. 46
- Bee E03: p. 47
- Bee E04: p. 48
- Bee E05: p. 49
- Bee E06: p. 50
- Bee E07: p. 51
- Bee R01: p. 52
- Bee R02: p. 53
- Bee R03: p. 54
- Bee R04: p. 55
- Bee R05: p. 56
- Bee R06: p. 57
- Bee R07: p. 58
- Bee R08: p. 59
- Bee R09: p. 60
- Bee R10: p. 61
- Bee R11: p. 62
- Bee R12: p. 63
- Bee R13: p. 64
- Bee R14: p. 65
- Bee R15: p. 66
- Bee R16: p. 67

*corresponding author

Bee A01

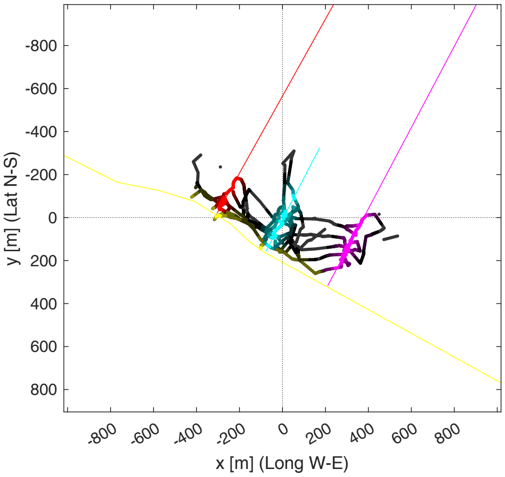
y [m] (Lat N-S)
x [m] (Long W-E)

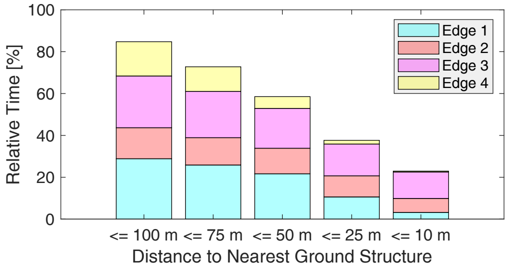
Relative Time [%]
Edge 1
Edge 2
Edge 3
Edge 4
<= 100 m <= 75 m <= 50 m <= 25 m <= 10 m
Distance to Nearest Ground Structure

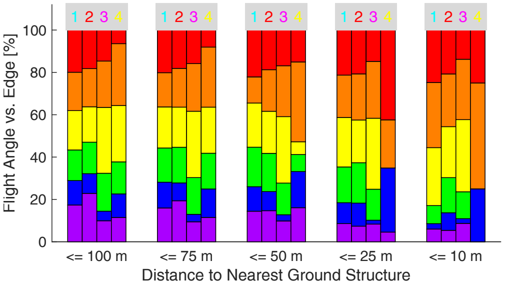
Flight Angle vs. Edge [%]
1 2 3 4
<= 100 m <= 75 m <= 50 m <= 25 m <= 10 m
Distance to Nearest Ground Structure

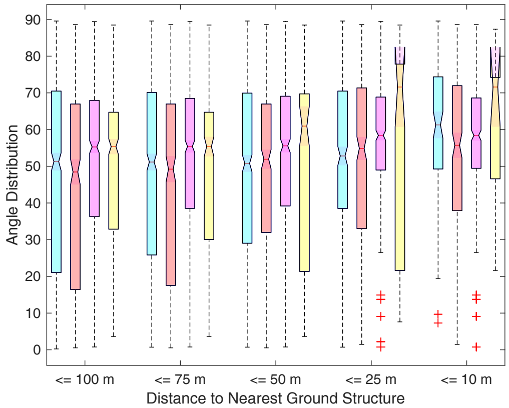
Angle Distribution
<= 100 m <= 75 m <= 50 m <= 25 m <= 10 m
Distance to Nearest Ground Structure

Bee A02

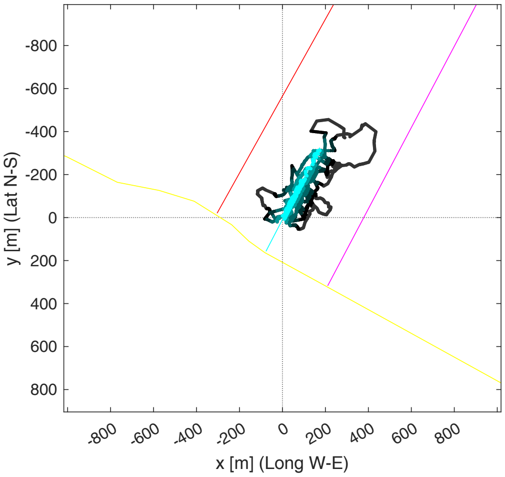
y [m] (Lat N-S)
x [m] (Long W-E)
-800
-600
-400
-200
0
200
400
600
800

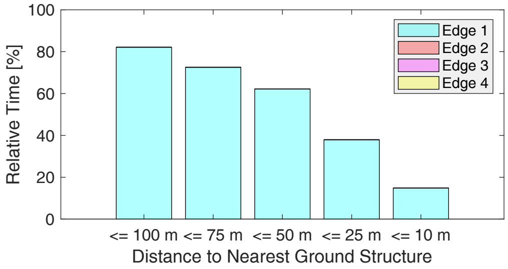
Relative Time [%]
Edge 1
Edge 2
Edge 3
Edge 4
<= 100 m
<= 75 m
<= 50 m
<= 25 m
<= 10 m
Distance to Nearest Ground Structure

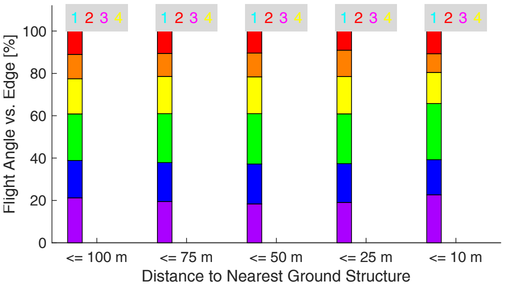
Flight Angle vs. Edge [%]
1 2 3 4
<= 100 m
<= 75 m
<= 50 m
<= 25 m
<= 10 m
Distance to Nearest Ground Structure

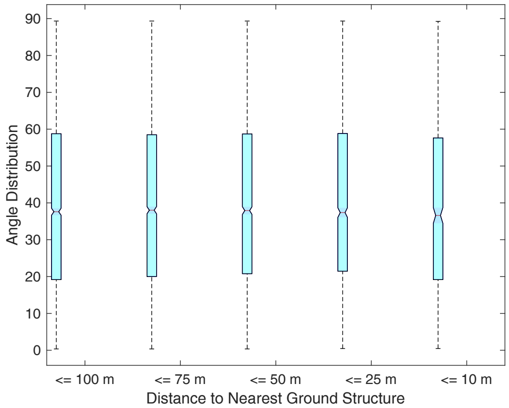
Angle Distribution
<= 100 m
<= 75 m
<= 50 m
<= 25 m
<= 10 m
Distance to Nearest Ground Structure

Bee A03

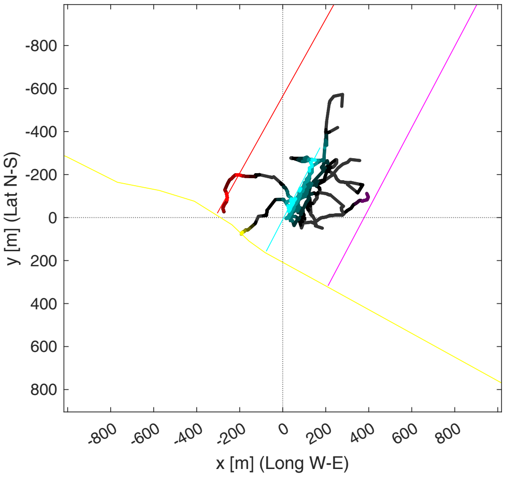
y [m] (Lat N-S)
x [m] (Long W-E)

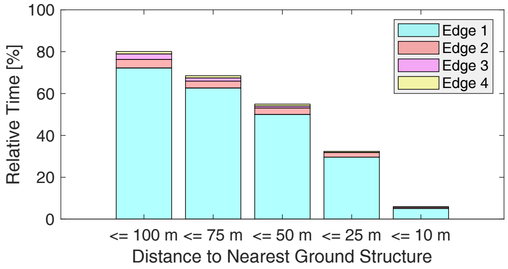
Edge 1
Edge 2
Edge 3
Edge 4
Relative Time [%]
<= 100 m
<= 75 m
<= 50 m
<= 25 m
<= 10 m
Distance to Nearest Ground Structure

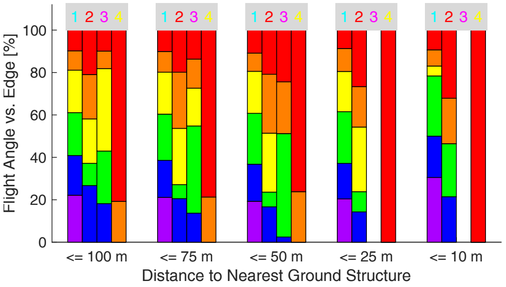
Flight Angle vs. Edge [%]
<= 100 m
<= 75 m
<= 50 m
<= 25 m
<= 10 m
Distance to Nearest Ground Structure

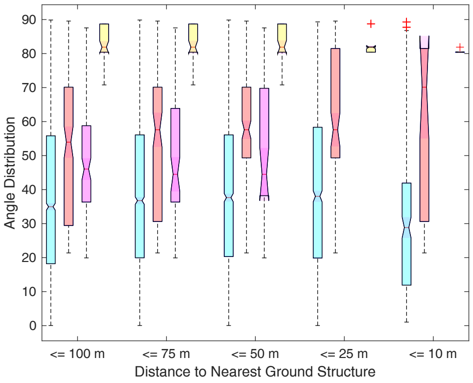
Angle Distribution
<= 100 m
<= 75 m
<= 50 m
<= 25 m
<= 10 m
Distance to Nearest Ground Structure

Bee A04

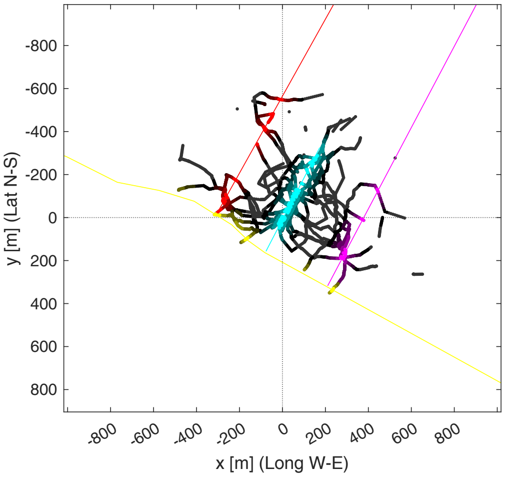
y [m] (Lat N-S)
x [m] (Long W-E)

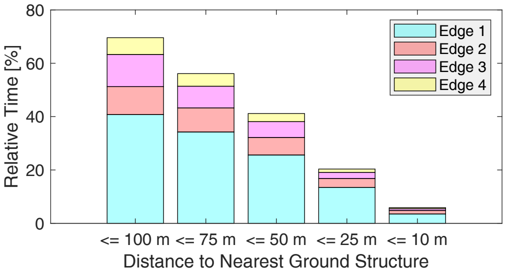
Relative Time [%]
Edge 1
Edge 2
Edge 3
Edge 4
<= 100 m <= 75 m <= 50 m <= 25 m <= 10 m
Distance to Nearest Ground Structure

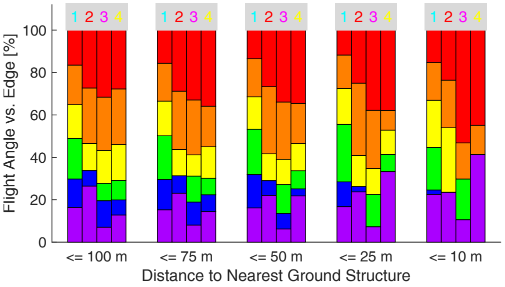
Flight Angle vs. Edge [%]
1 2 3 4
<= 100 m
<= 75 m
<= 50 m
<= 25 m
<= 10 m
Distance to Nearest Ground Structure

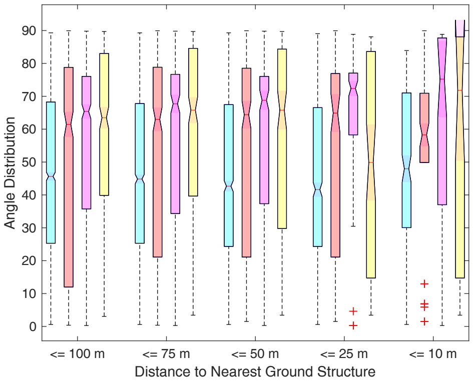
Angle Distribution
<= 100 m
<= 75 m
<= 50 m
<= 25 m
<= 10 m
Distance to Nearest Ground Structure

Bee A05

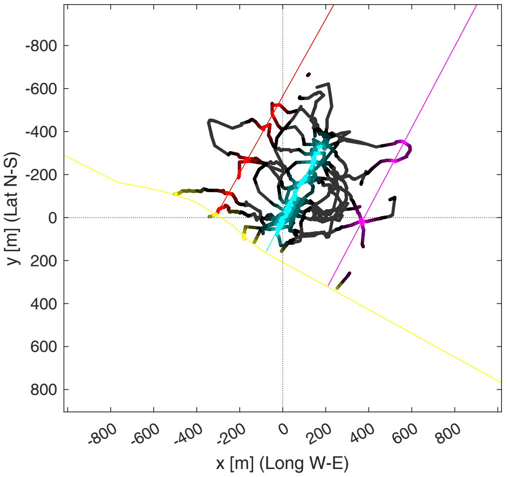

-800
-600
-400
-200
0
200
400
600
800
y [m] (Lat N-S)
-800 -600 -400 -200 0 200 400 600 800
x [m] (Long W-E)

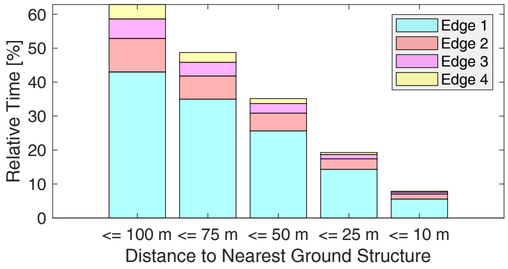

Edge 1
Edge 2
Edge 3
Edge 4
0 10 20 30 40 50 60
Relative Time [%]
<= 100 m <= 75 m <= 50 m <= 25 m <= 10 m
Distance to Nearest Ground Structure

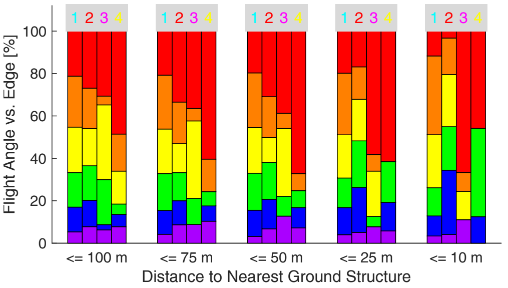

1 2 3 4
0 20 40 60 80 100
Flight Angle vs. Edge [%]
<= 100 m <= 75 m <= 50 m <= 25 m <= 10 m
Distance to Nearest Ground Structure

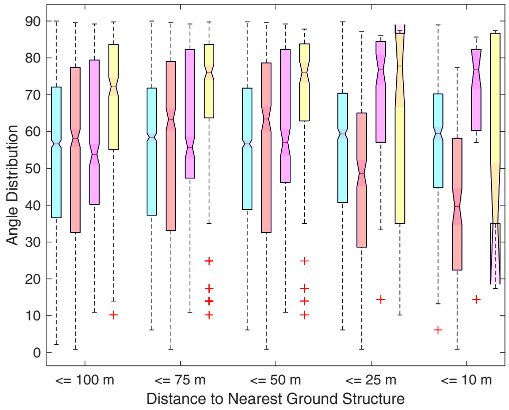

0 10 20 30 40 50 60 70 80 90
Angle Distribution
<= 100 m <= 75 m <= 50 m <= 25 m <= 10 m
Distance to Nearest Ground Structure

Bee A06

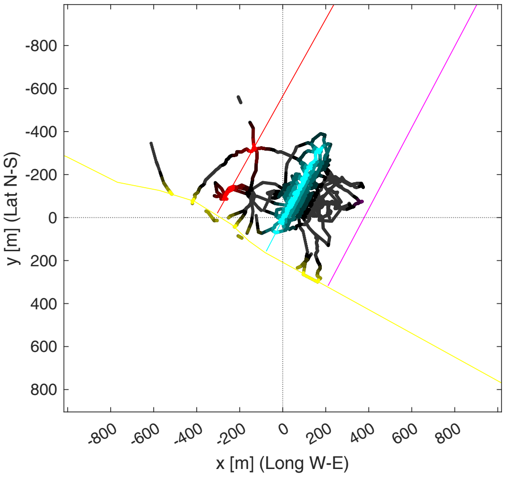
y [m] (Lat N-S)
x [m] (Long W-E)

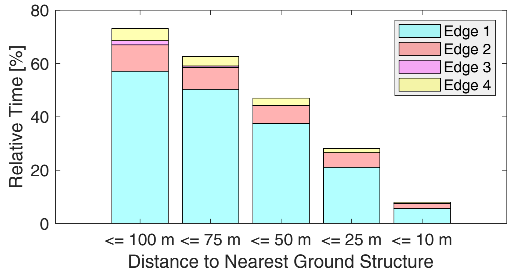
Edge 1
Edge 2
Edge 3
Edge 4
Relative Time [%]
<= 100 m
<= 75 m
<= 50 m
<= 25 m
<= 10 m
Distance to Nearest Ground Structure

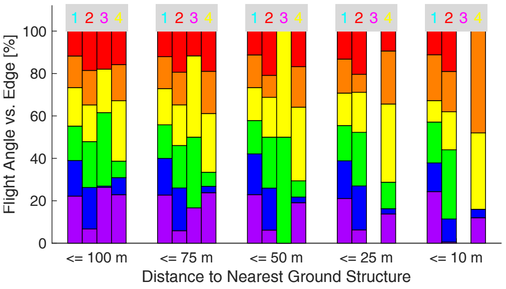
1 2 3 4
Flight Angle vs. Edge [%]
<= 100 m
<= 75 m
<= 50 m
<= 25 m
<= 10 m
Distance to Nearest Ground Structure

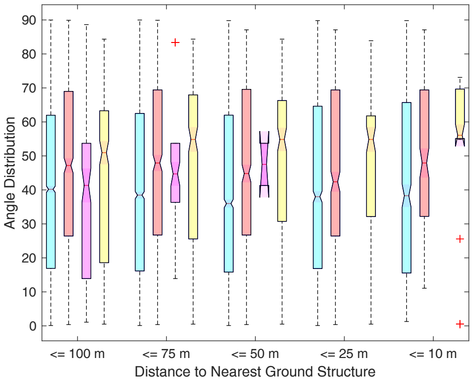
Angle Distribution
<= 100 m
<= 75 m
<= 50 m
<= 25 m
<= 10 m
Distance to Nearest Ground Structure

Bee A07

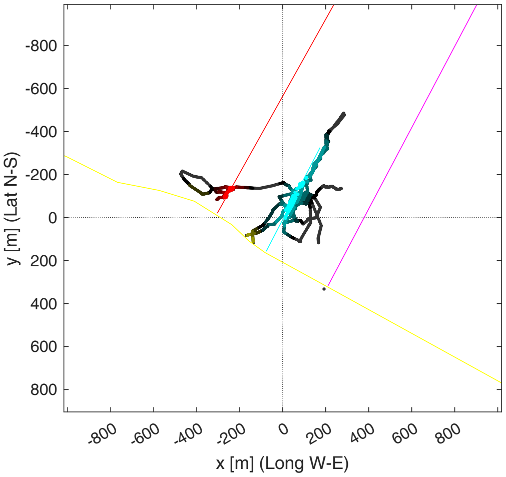
y [m] (Lat N-S)
x [m] (Long W-E)

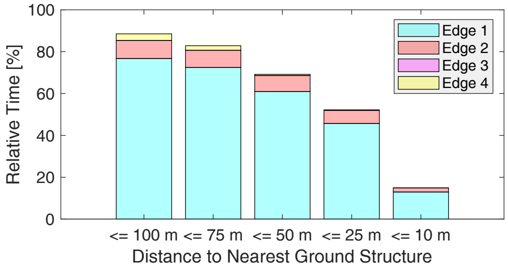
Edge 1
Edge 2
Edge 3
Edge 4
Relative Time [%]
<= 100 m
<= 75 m
<= 50 m
<= 25 m
<= 10 m
Distance to Nearest Ground Structure

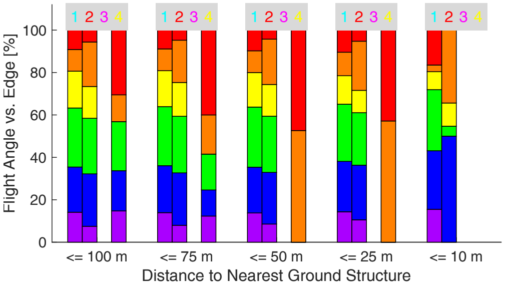
1 2 3 4
Flight Angle vs. Edge [%]
<= 100 m
<= 75 m
<= 50 m
<= 25 m
<= 10 m
Distance to Nearest Ground Structure

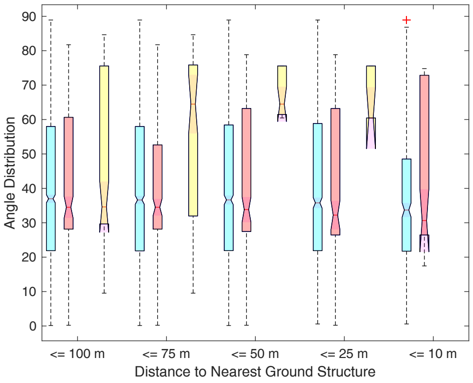
Angle Distribution
<= 100 m
<= 75 m
<= 50 m
<= 25 m
<= 10 m
Distance to Nearest Ground Structure

Bee A08

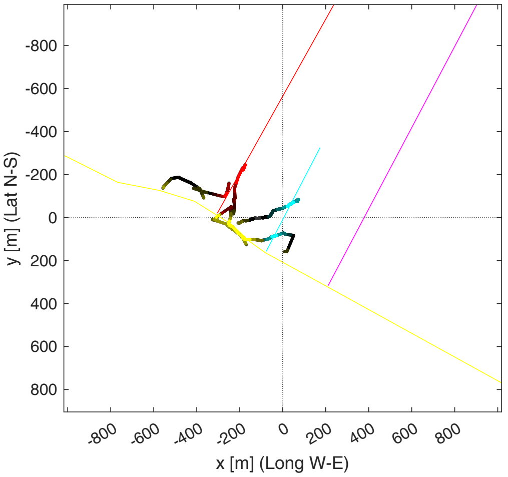
y [m] (Lat N-S)
x [m] (Long W-E)

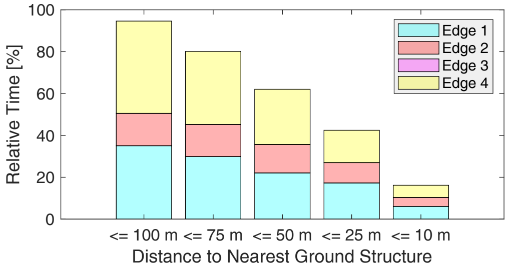
Edge 1
Edge 2
Edge 3
Edge 4
Relative Time [%]
<= 100 m
<= 75 m
<= 50 m
<= 25 m
<= 10 m
Distance to Nearest Ground Structure

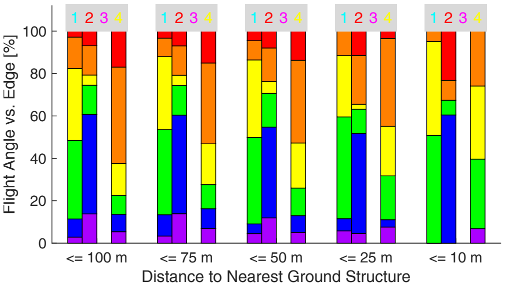
Flight Angle vs. Edge [%]
<= 100 m
<= 75 m
<= 50 m
<= 25 m
<= 10 m
Distance to Nearest Ground Structure

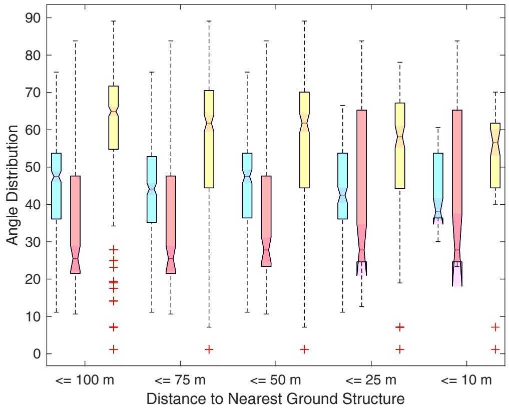
Angle Distribution
<= 100 m
<= 75 m
<= 50 m
<= 25 m
<= 10 m
Distance to Nearest Ground Structure

Bee A09

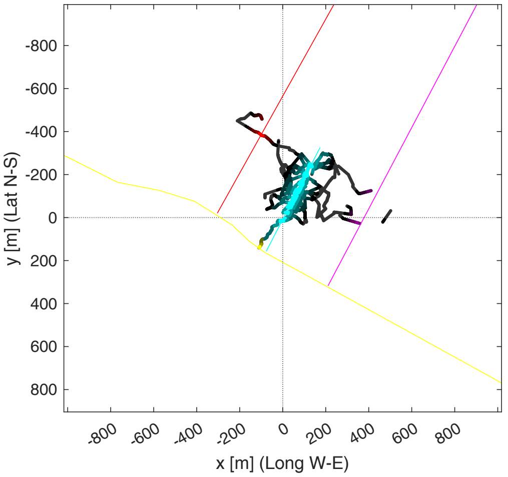
y [m] (Lat N-S)
x [m] (Long W-E)

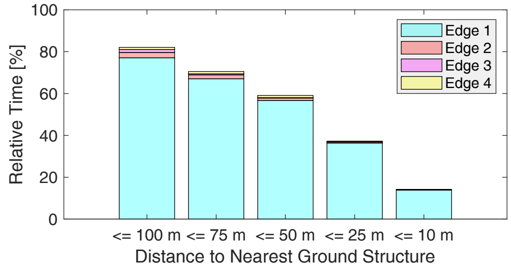
Edge 1
Edge 2
Edge 3
Edge 4
Relative Time [%]
<= 100 m
<= 75 m
<= 50 m
<= 25 m
<= 10 m
Distance to Nearest Ground Structure

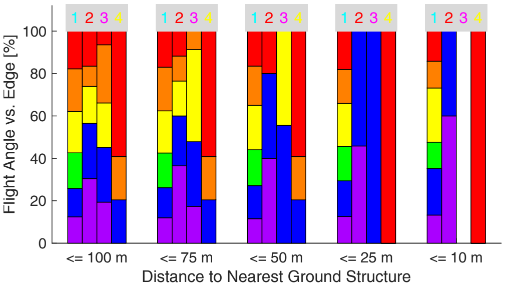
Flight Angle vs. Edge [%]
<= 100 m
<= 75 m
<= 50 m
<= 25 m
<= 10 m
Distance to Nearest Ground Structure

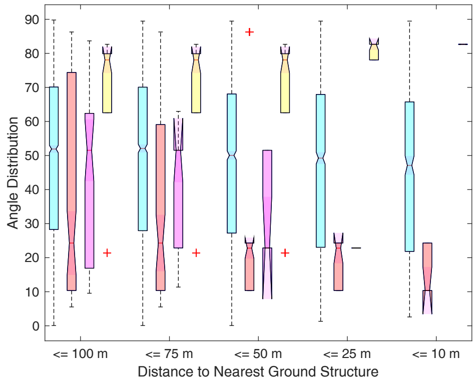
Angle Distribution
<= 100 m
<= 75 m
<= 50 m
<= 25 m
<= 10 m
Distance to Nearest Ground Structure

Bee A10

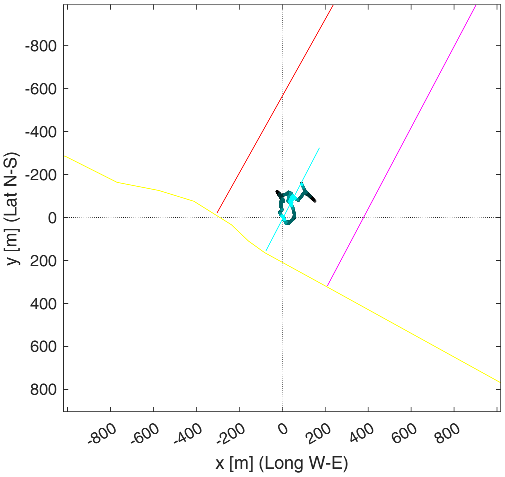
y [m] (Lat N-S)
x [m] (Long W-E)
-800
-600
-400
-200
0
200
400
600
800

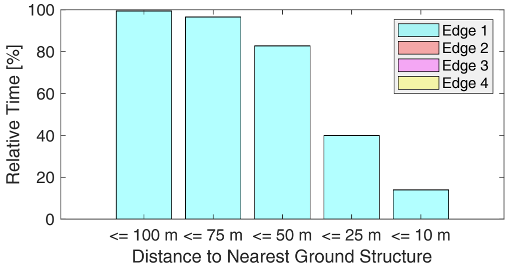
Relative Time [%]
Edge 1
Edge 2
Edge 3
Edge 4
<= 100 m
<= 75 m
<= 50 m
<= 25 m
<= 10 m
Distance to Nearest Ground Structure

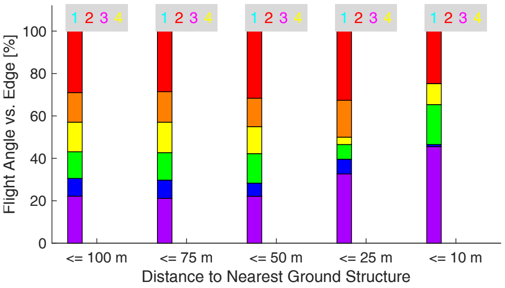
Flight Angle vs. Edge [%]
1 2 3 4
<= 100 m
<= 75 m
<= 50 m
<= 25 m
<= 10 m
Distance to Nearest Ground Structure

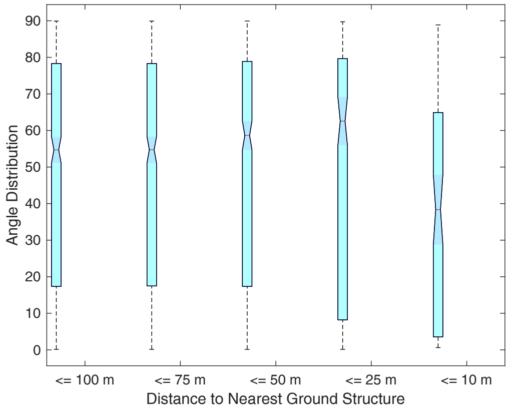
Angle Distribution
<= 100 m
<= 75 m
<= 50 m
<= 25 m
<= 10 m
Distance to Nearest Ground Structure

Bee A11

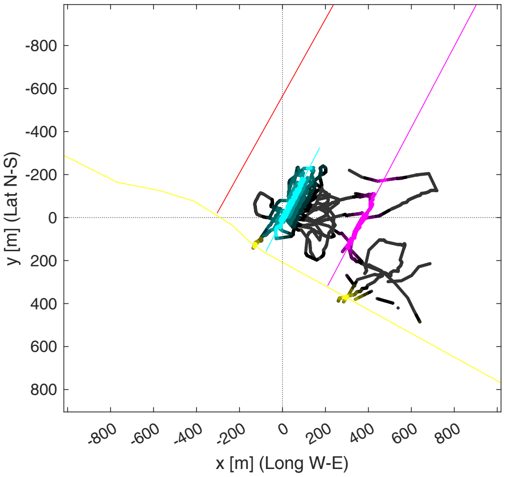
y [m] (Lat N-S)
x [m] (Long W-E)
-800
-600
-400
-200
0
200
400
600
800

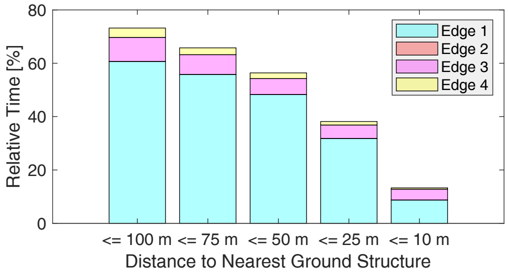
Relative Time [%]
Edge 1
Edge 2
Edge 3
Edge 4
<= 100 m
<= 75 m
<= 50 m
<= 25 m
<= 10 m
Distance to Nearest Ground Structure

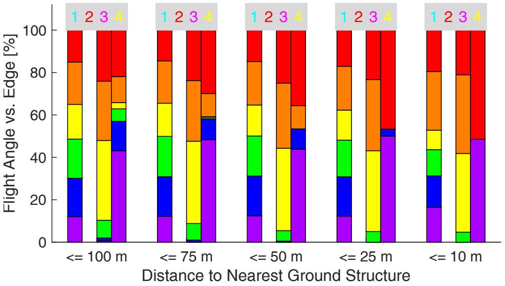
Flight Angle vs. Edge [%]
1 2 3 4
<= 100 m
<= 75 m
<= 50 m
<= 25 m
<= 10 m
Distance to Nearest Ground Structure

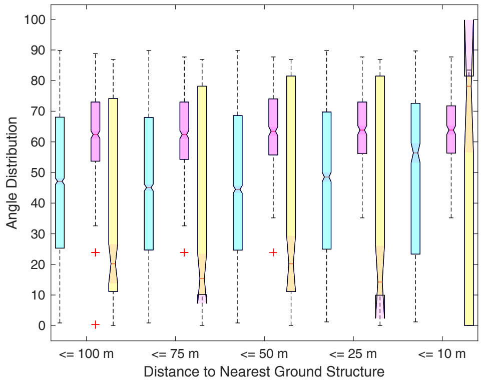
Angle Distribution
<= 100 m
<= 75 m
<= 50 m
<= 25 m
<= 10 m
Distance to Nearest Ground Structure

Bee A12

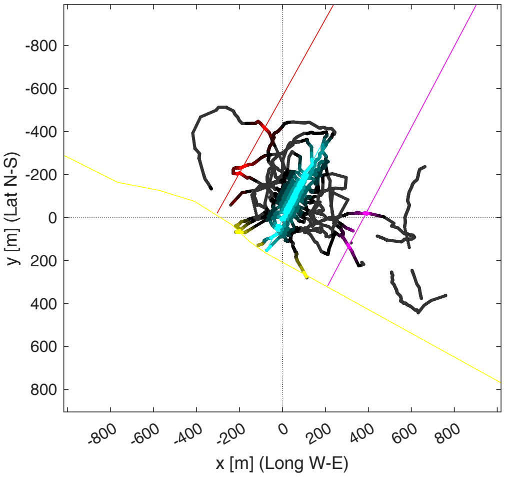
y [m] (Lat N-S)
x [m] (Long W-E)

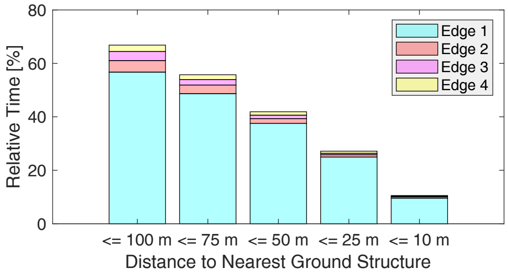
Edge 1
Edge 2
Edge 3
Edge 4
Relative Time [%]
<= 100 m
<= 75 m
<= 50 m
<= 25 m
<= 10 m
Distance to Nearest Ground Structure

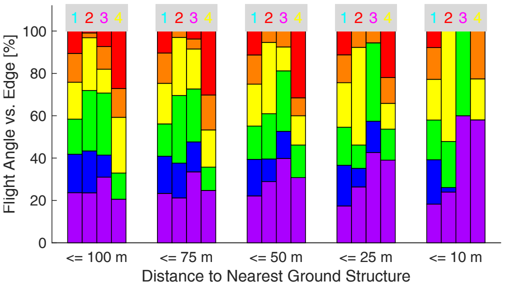
Flight Angle vs. Edge [%]
1 2 3 4
<= 100 m
<= 75 m
<= 50 m
<= 25 m
<= 10 m
Distance to Nearest Ground Structure

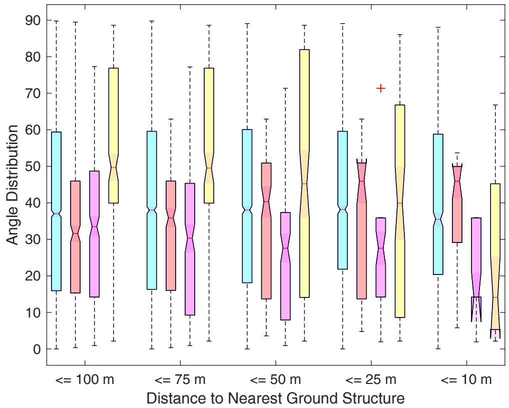
Angle Distribution
<= 100 m
<= 75 m
<= 50 m
<= 25 m
<= 10 m
Distance to Nearest Ground Structure

Bee A13

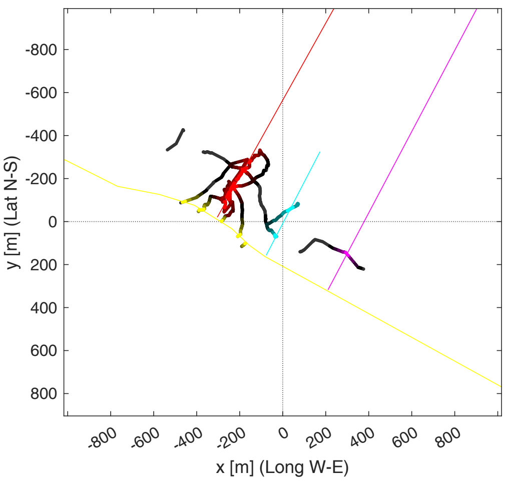

y [m] (Lat N-S)
x [m] (Long W-E)
-800
-600
-400
-200
0
200
400
600
800

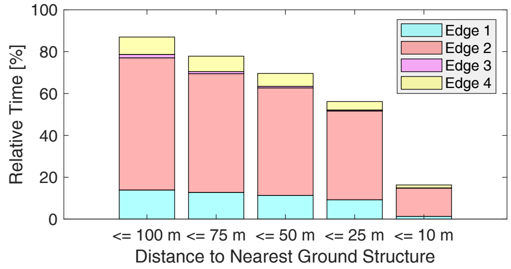

Relative Time [%]
Edge 1
Edge 2
Edge 3
Edge 4
<= 100 m
<= 75 m
<= 50 m
<= 25 m
<= 10 m
Distance to Nearest Ground Structure

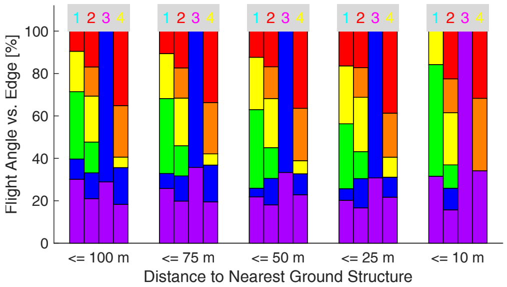

Flight Angle vs. Edge [%]
1 2 3 4
<= 100 m
<= 75 m
<= 50 m
<= 25 m
<= 10 m
Distance to Nearest Ground Structure

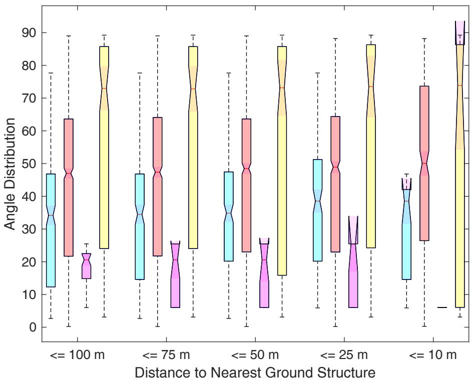

Angle Distribution
<= 100 m
<= 75 m
<= 50 m
<= 25 m
<= 10 m
Distance to Nearest Ground Structure

Bee A14

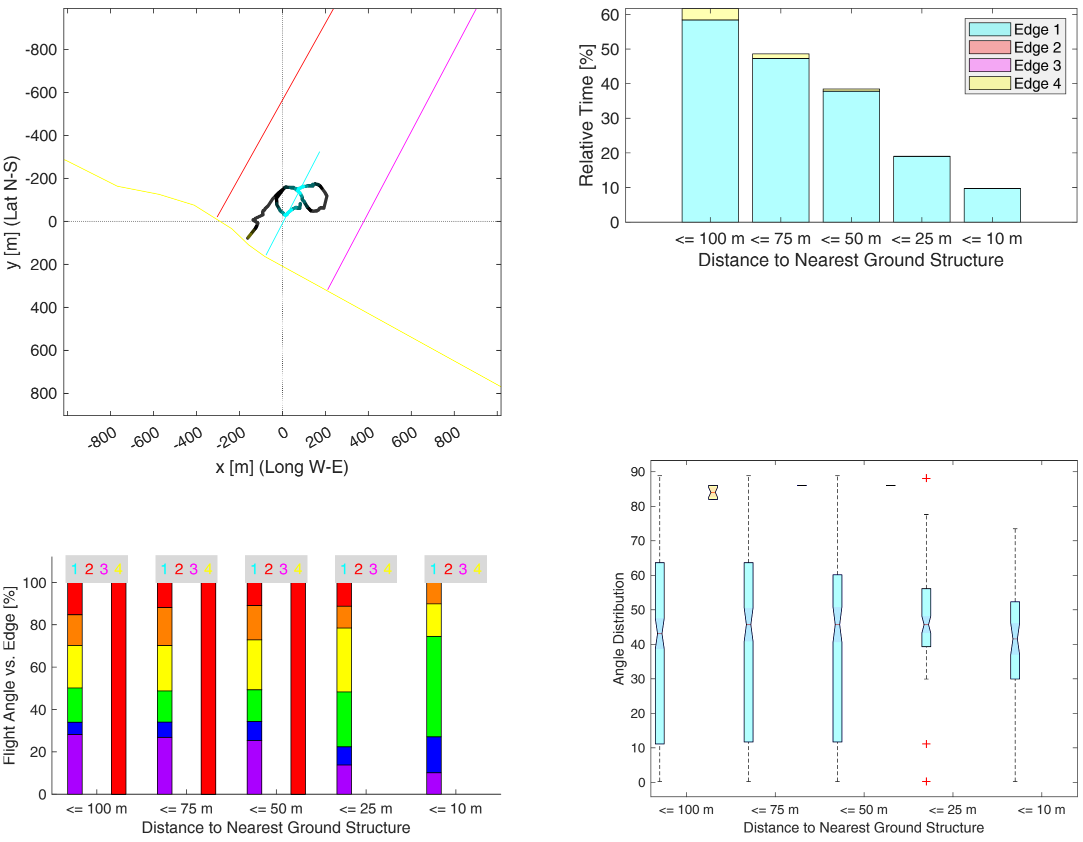
y [m] (Lat N-S)
x [m] (Long W-E)
Relative Time [%]
Edge 1
Edge 2
Edge 3
Edge 4
<= 100 m <= 75 m <= 50 m <= 25 m <= 10 m
Distance to Nearest Ground Structure
Flight Angle vs. Edge [%]
1 2 3 4
Angle Distribution

Bee B01

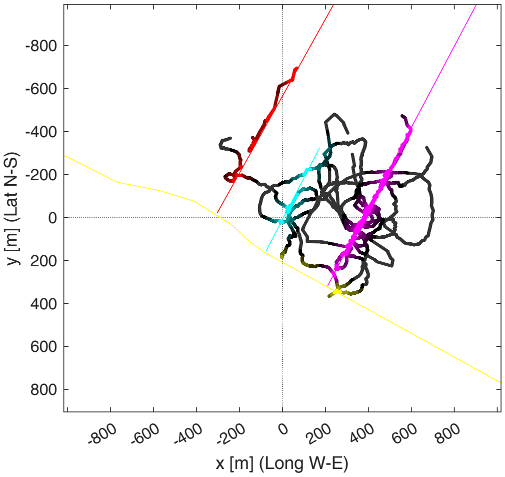
y [m] (Lat N-S)
x [m] (Long W-E)

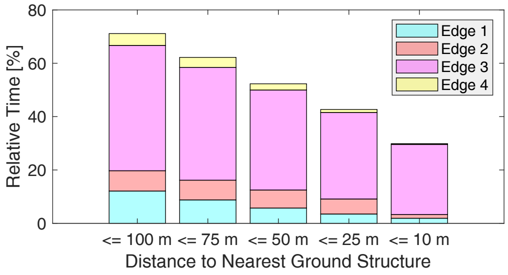
Relative Time [%]
Edge 1
Edge 2
Edge 3
Edge 4
<= 100 m
<= 75 m
<= 50 m
<= 25 m
<= 10 m
Distance to Nearest Ground Structure

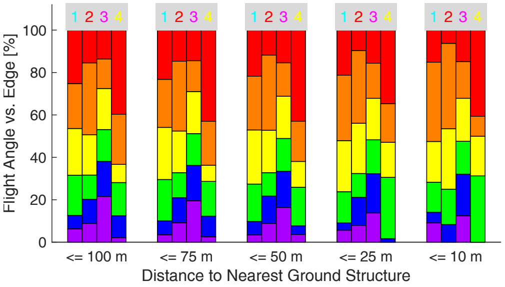
Flight Angle vs. Edge [%]
1 2 3 4
<= 100 m
<= 75 m
<= 50 m
<= 25 m
<= 10 m
Distance to Nearest Ground Structure

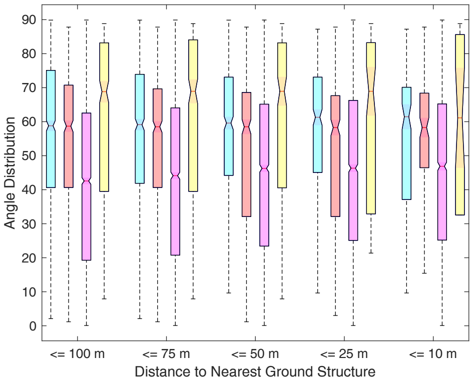
Angle Distribution
<= 100 m
<= 75 m
<= 50 m
<= 25 m
<= 10 m
Distance to Nearest Ground Structure

Bee B02

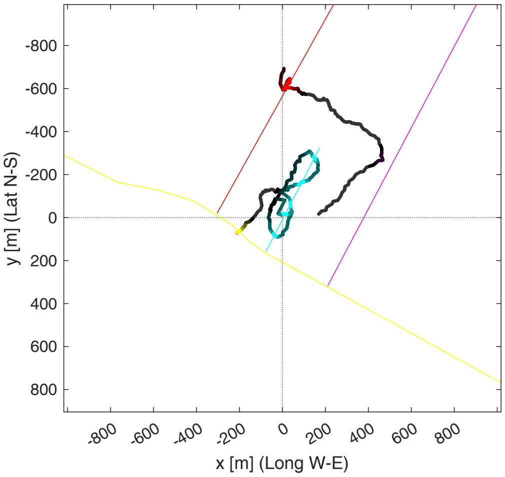
y [m] (Lat N-S)
x [m] (Long W-E)

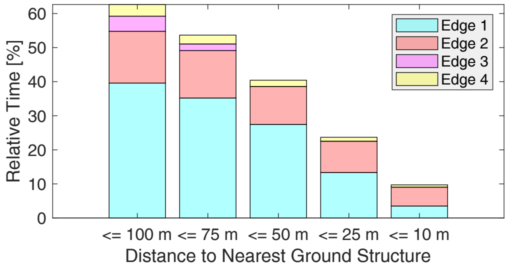
Edge 1
Edge 2
Edge 3
Edge 4
Relative Time [%]
<= 100 m
<= 75 m
<= 50 m
<= 25 m
<= 10 m
Distance to Nearest Ground Structure

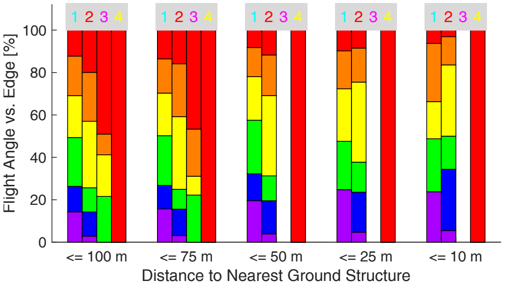
Flight Angle vs. Edge [%]
<= 100 m
<= 75 m
<= 50 m
<= 25 m
<= 10 m
Distance to Nearest Ground Structure

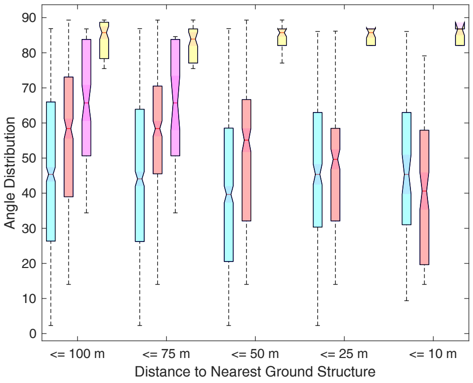
Angle Distribution
<= 100 m
<= 75 m
<= 50 m
<= 25 m
<= 10 m
Distance to Nearest Ground Structure

Bee B03

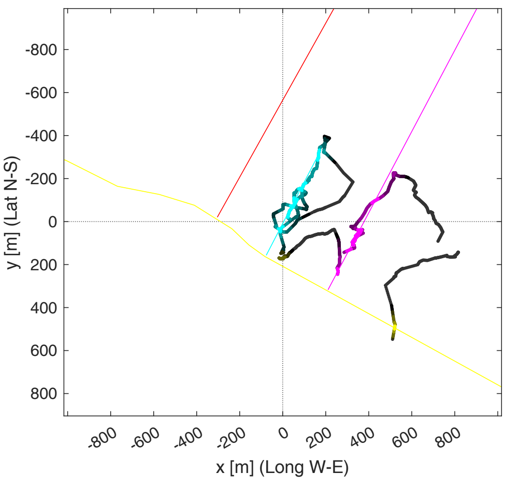
y [m] (Lat N-S)
x [m] (Long W-E)

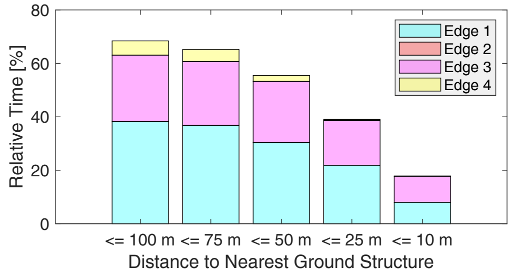
Edge 1
Edge 2
Edge 3
Edge 4
Relative Time [%]
<= 100 m
<= 75 m
<= 50 m
<= 25 m
<= 10 m
Distance to Nearest Ground Structure

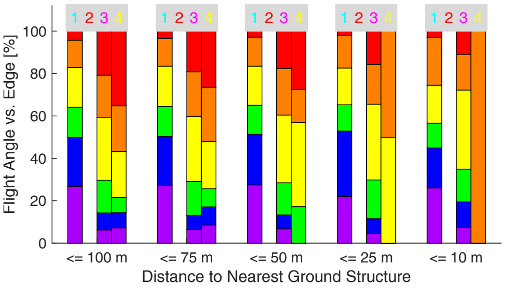
Flight Angle vs. Edge [%]
<= 100 m
<= 75 m
<= 50 m
<= 25 m
<= 10 m
Distance to Nearest Ground Structure

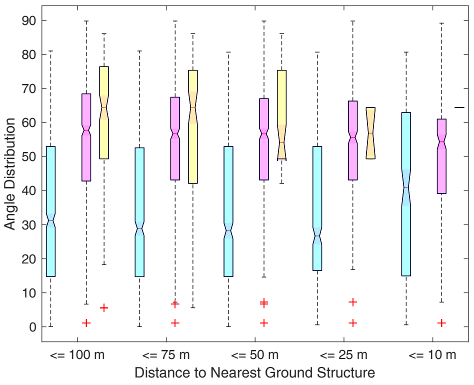
Angle Distribution
<= 100 m
<= 75 m
<= 50 m
<= 25 m
<= 10 m
Distance to Nearest Ground Structure

Bee B04

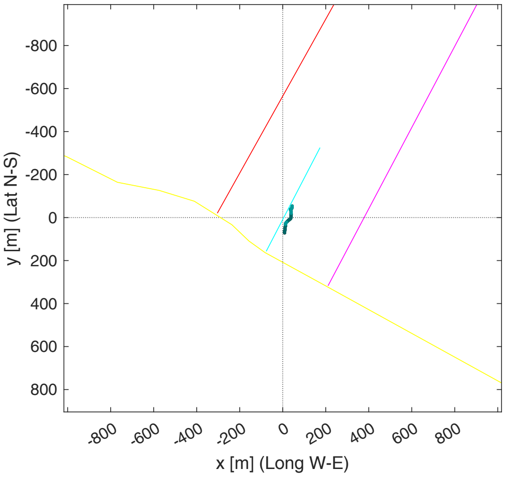
y [m] (Lat N-S)
x [m] (Long W-E)
-800
-600
-400
-200
0
200
400
600
800

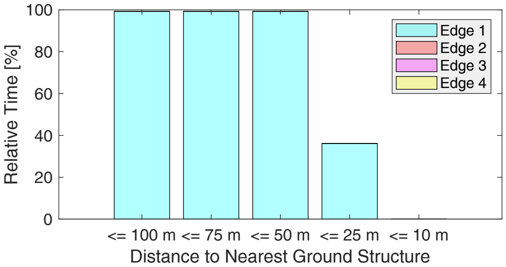
Relative Time [%]
Edge 1
Edge 2
Edge 3
Edge 4
<= 100 m
<= 75 m
<= 50 m
<= 25 m
<= 10 m
Distance to Nearest Ground Structure

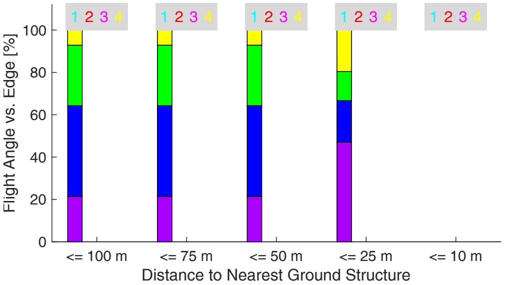
Flight Angle vs. Edge [%]
1 2 3 4
<= 100 m
<= 75 m
<= 50 m
<= 25 m
<= 10 m
Distance to Nearest Ground Structure

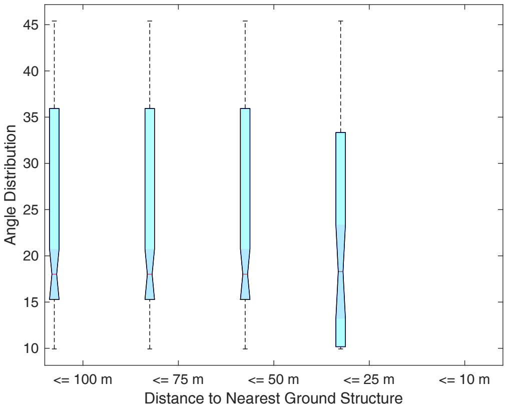
Angle Distribution
<= 100 m
<= 75 m
<= 50 m
<= 25 m
<= 10 m
Distance to Nearest Ground Structure

Bee B05

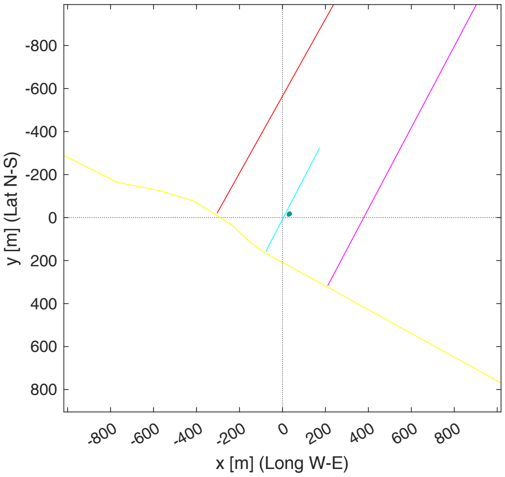
-800
-600
-400
-200
0
200
400
600
800
y [m] (Lat N-S)
-800
-600
-400
-200
0
200
400
600
800
x [m] (Long W-E)

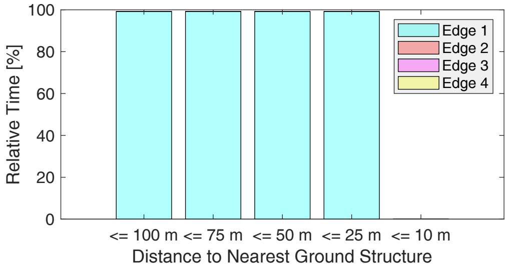
100
80
60
40
20
0
Relative Time [%]
Edge 1
Edge 2
Edge 3
Edge 4
<= 100 m
<= 75 m
<= 50 m
<= 25 m
<= 10 m
Distance to Nearest Ground Structure

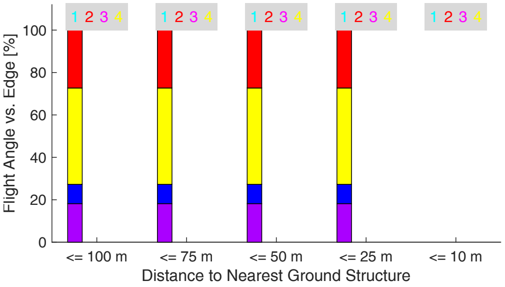
1 2 3 4
100
80
60
40
20
0
Flight Angle vs. Edge [%]
<= 100 m
<= 75 m
<= 50 m
<= 25 m
<= 10 m
Distance to Nearest Ground Structure

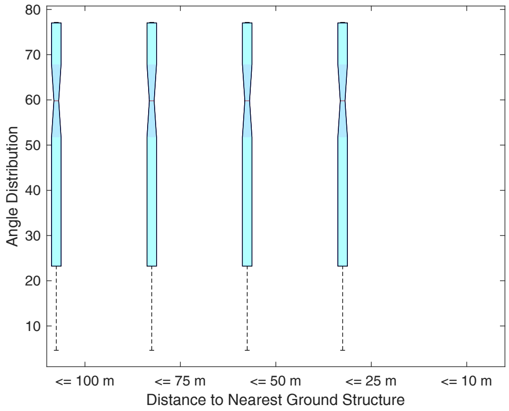
80
70
60
50
40
30
20
10
Angle Distribution
<= 100 m
<= 75 m
<= 50 m
<= 25 m
<= 10 m
Distance to Nearest Ground Structure

Bee B06

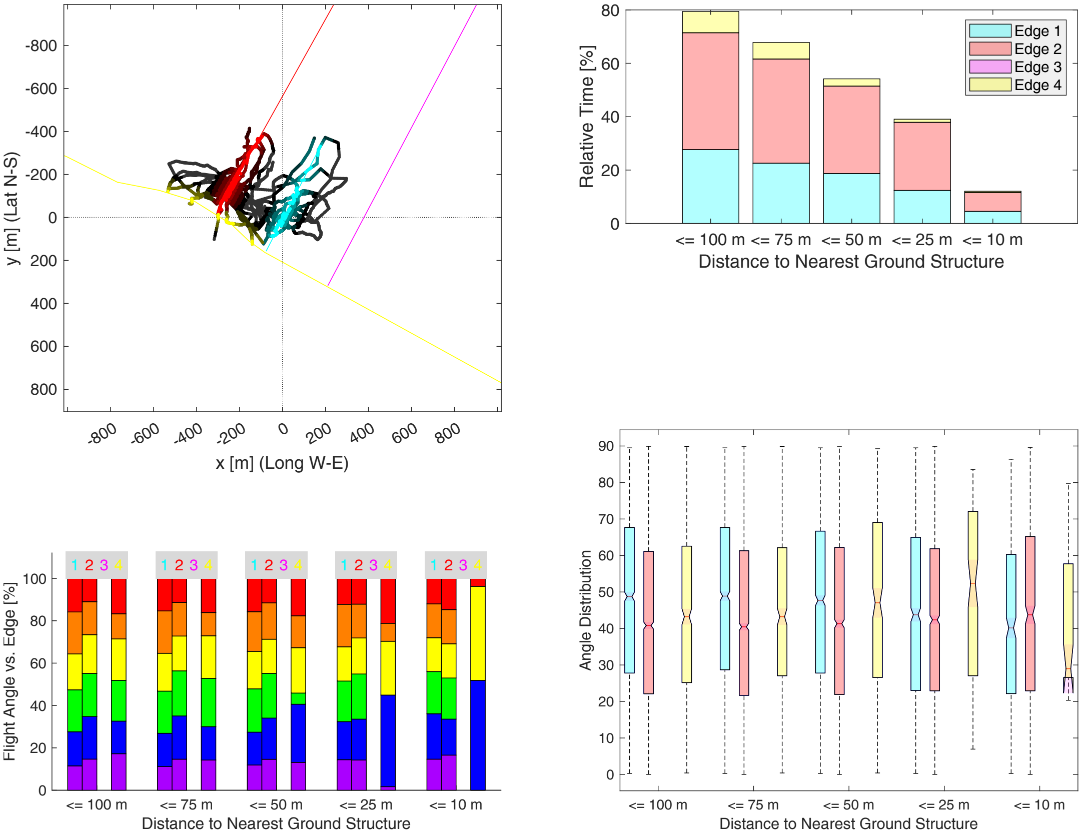
y [m] (Lat N-S)
x [m] (Long W-E)
Relative Time [%]
Edge 1
Edge 2
Edge 3
Edge 4
Distance to Nearest Ground Structure
Flight Angle vs. Edge [%]
Angle Distribution
<= 100 m
<= 75 m
<= 50 m
<= 25 m
<= 10 m

Bee B07

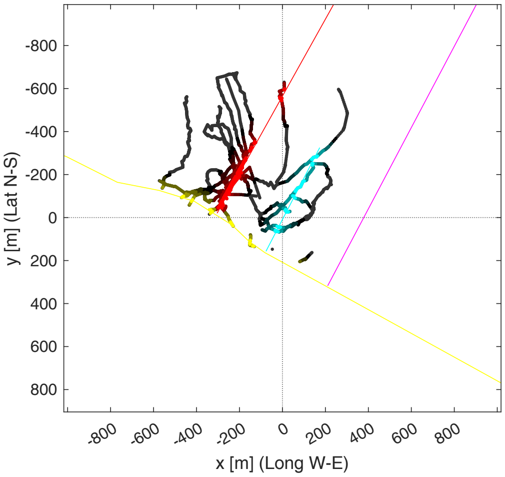
-800
-600
-400
-200
0
200
400
600
800
y [m] (Lat N-S)
x [m] (Long W-E)

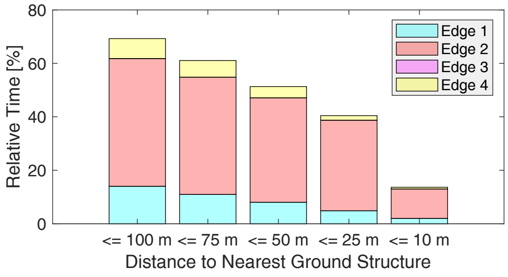
Edge 1
Edge 2
Edge 3
Edge 4
Relative Time [%]
<= 100 m
<= 75 m
<= 50 m
<= 25 m
<= 10 m
Distance to Nearest Ground Structure

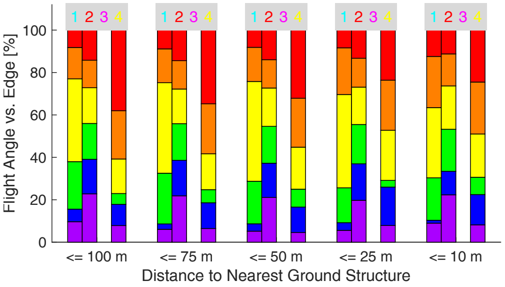
1 2 3 4
Flight Angle vs. Edge [%]
<= 100 m
<= 75 m
<= 50 m
<= 25 m
<= 10 m
Distance to Nearest Ground Structure

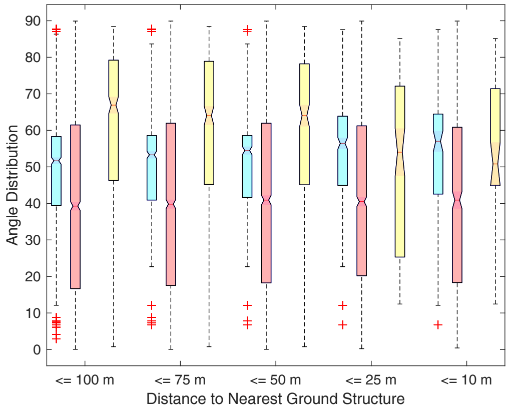
Angle Distribution
<= 100 m
<= 75 m
<= 50 m
<= 25 m
<= 10 m
Distance to Nearest Ground Structure

Bee B08

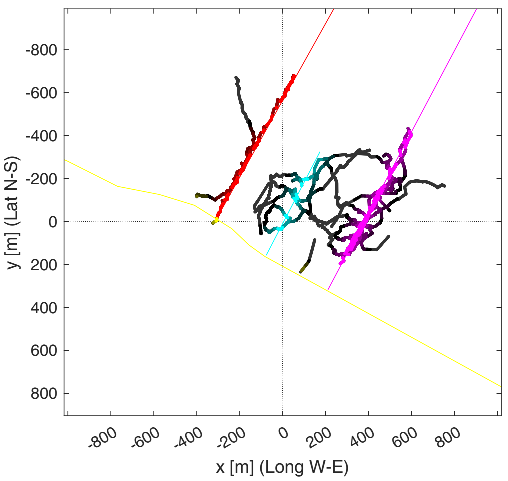
y [m] (Lat N-S)
x [m] (Long W-E)
-800
-600
-400
-200
0
200
400
600
800

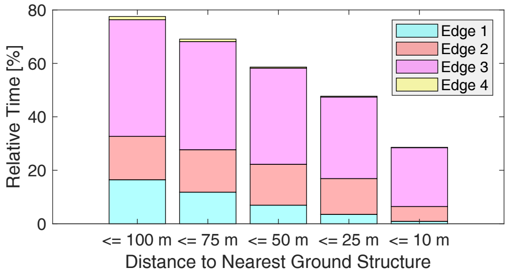
Relative Time [%]
Edge 1
Edge 2
Edge 3
Edge 4
<= 100 m
<= 75 m
<= 50 m
<= 25 m
<= 10 m
Distance to Nearest Ground Structure

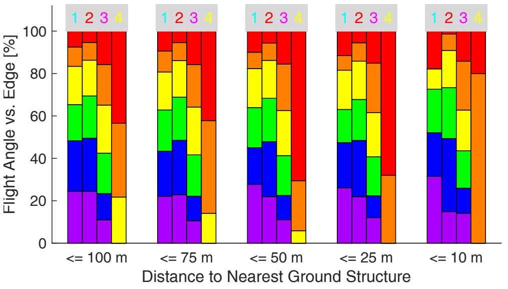
Flight Angle vs. Edge [%]
1 2 3 4
<= 100 m
<= 75 m
<= 50 m
<= 25 m
<= 10 m
Distance to Nearest Ground Structure

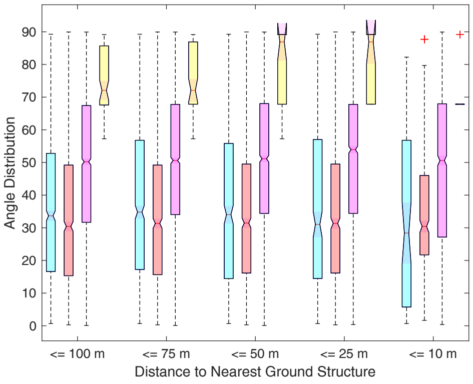
Angle Distribution
<= 100 m
<= 75 m
<= 50 m
<= 25 m
<= 10 m
Distance to Nearest Ground Structure

Bee B09

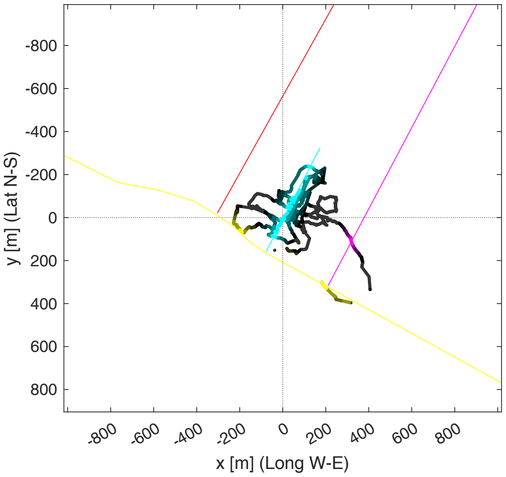
y [m] (Lat N-S)
x [m] (Long W-E)

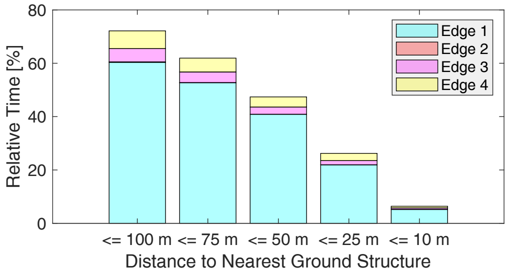
Edge 1
Edge 2
Edge 3
Edge 4
Relative Time [%]
<= 100 m <= 75 m <= 50 m <= 25 m <= 10 m
Distance to Nearest Ground Structure

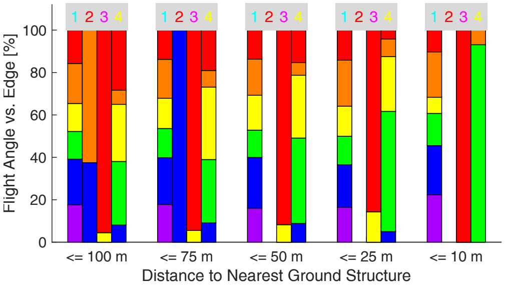
1 2 3 4
Flight Angle vs. Edge [%]
<= 100 m <= 75 m <= 50 m <= 25 m <= 10 m
Distance to Nearest Ground Structure

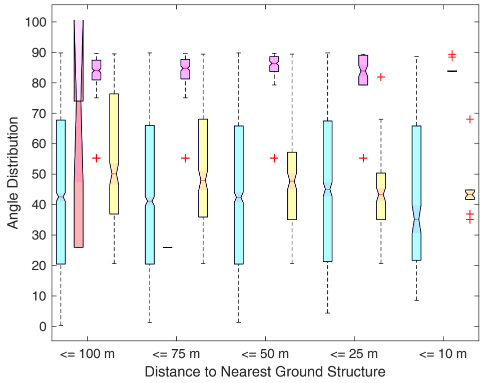
Angle Distribution
<= 100 m <= 75 m <= 50 m <= 25 m <= 10 m
Distance to Nearest Ground Structure

Bee B10

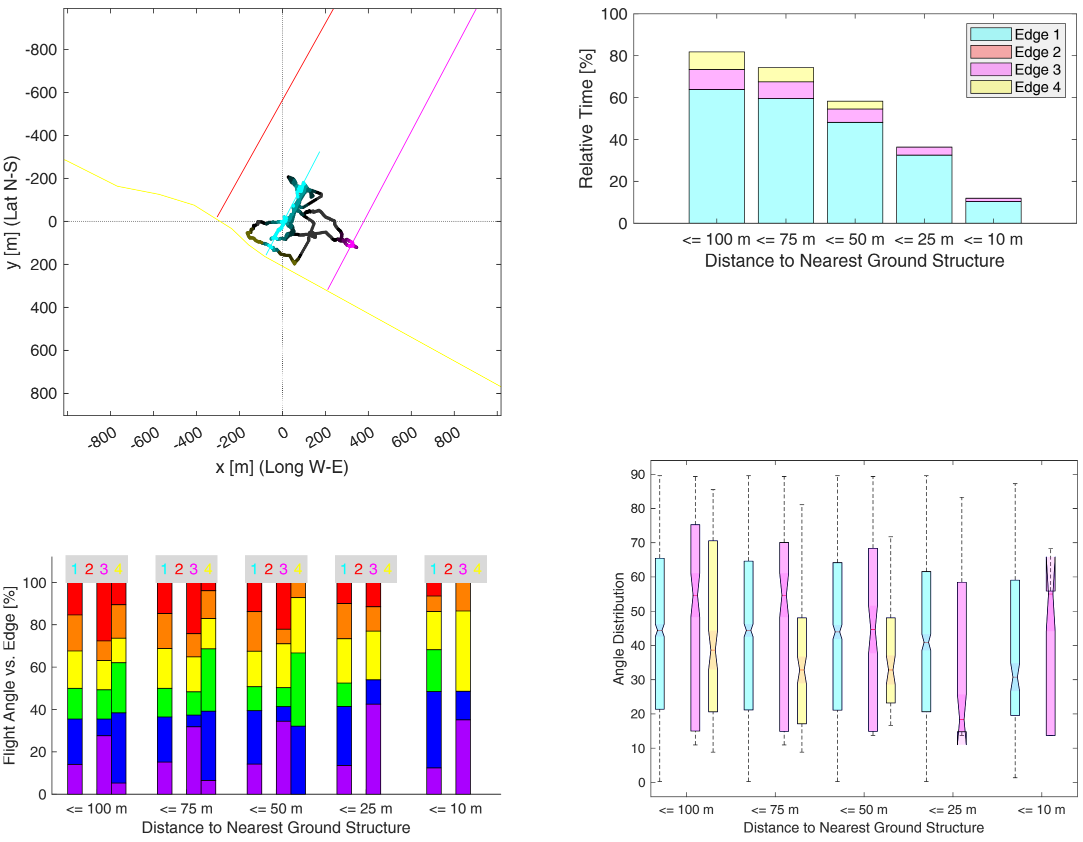
y [m] (Lat N-S)
x [m] (Long W-E)
Relative Time [%]
Edge 1
Edge 2
Edge 3
Edge 4
<= 100 m
<= 75 m
<= 50 m
<= 25 m
<= 10 m
Distance to Nearest Ground Structure
Flight Angle vs. Edge [%]
Angle Distribution

Bee B11

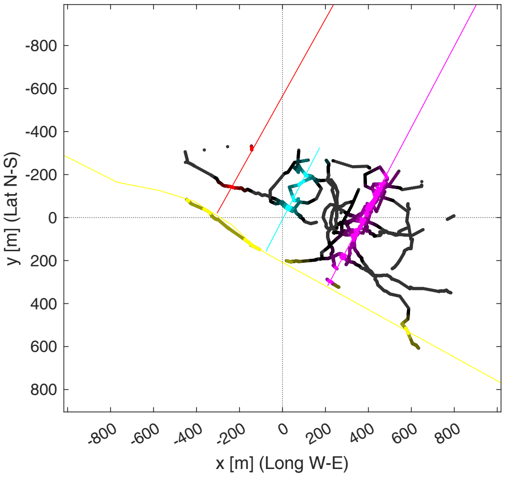
y [m] (Lat N-S)
x [m] (Long W-E)

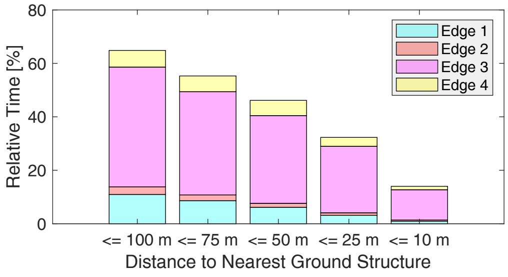
Edge 1
Edge 2
Edge 3
Edge 4
Relative Time [%]
<= 100 m
<= 75 m
<= 50 m
<= 25 m
<= 10 m
Distance to Nearest Ground Structure

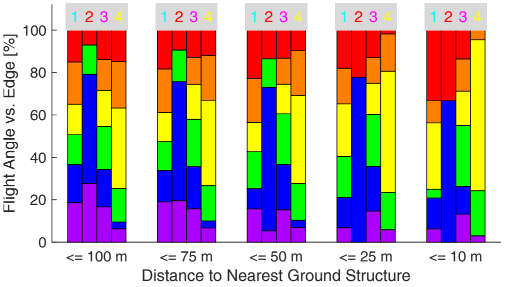
Flight Angle vs. Edge [%]
1 2 3 4
<= 100 m
<= 75 m
<= 50 m
<= 25 m
<= 10 m
Distance to Nearest Ground Structure

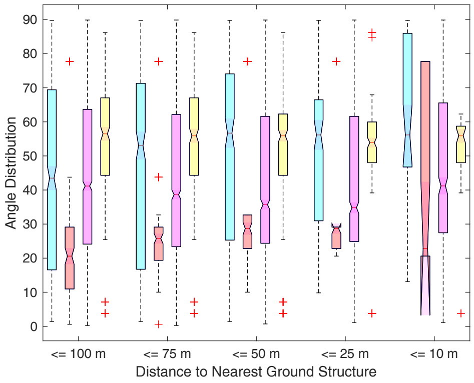
Angle Distribution
<= 100 m
<= 75 m
<= 50 m
<= 25 m
<= 10 m
Distance to Nearest Ground Structure

Bee C01

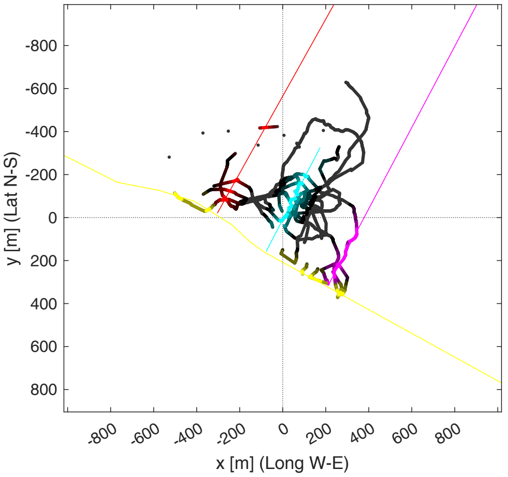
y [m] (Lat N-S)
x [m] (Long W-E)

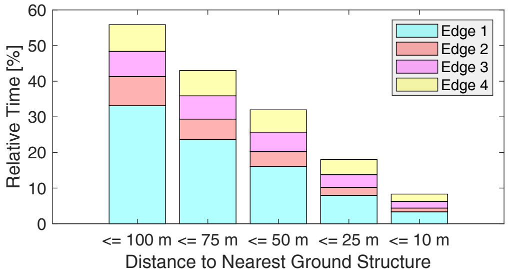
Edge 1
Edge 2
Edge 3
Edge 4
Relative Time [%]
<= 100 m
<= 75 m
<= 50 m
<= 25 m
<= 10 m
Distance to Nearest Ground Structure

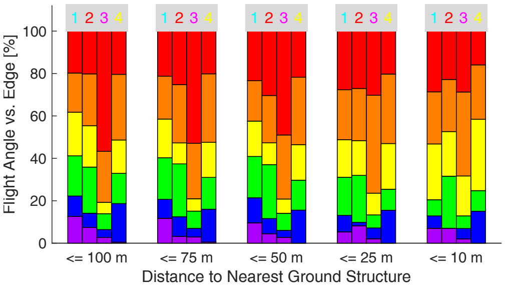
Flight Angle vs. Edge [%]
<= 100 m
<= 75 m
<= 50 m
<= 25 m
<= 10 m
Distance to Nearest Ground Structure

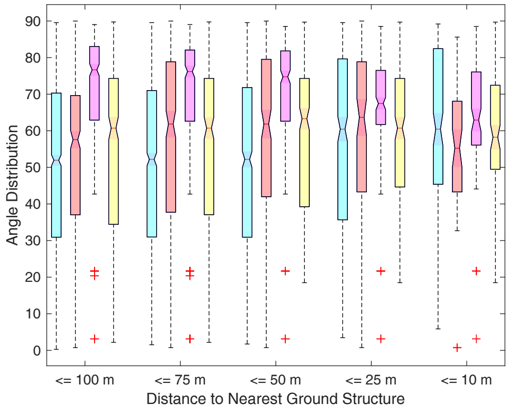
Angle Distribution
<= 100 m
<= 75 m
<= 50 m
<= 25 m
<= 10 m
Distance to Nearest Ground Structure

Bee C02

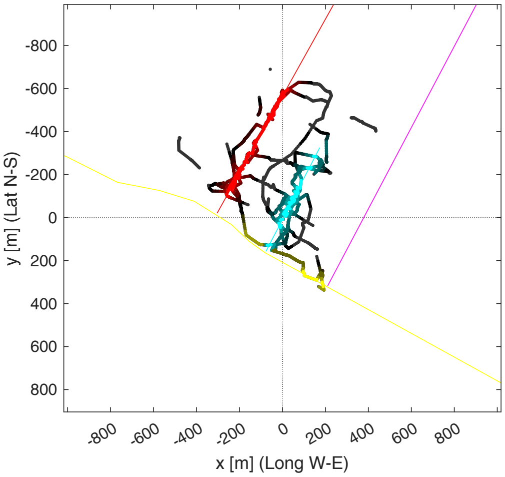
-800
-600
-400
-200
0
200
400
600
800
y [m] (Lat N-S)
-800
-600
-400
-200
0
200
400
600
800
x [m] (Long W-E)

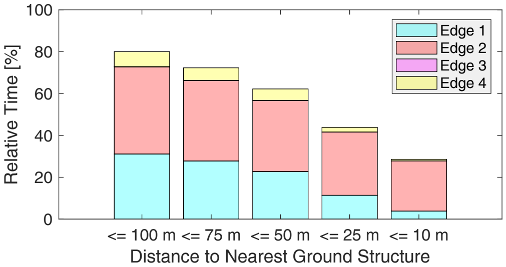
100
80
60
40
20
0
Relative Time [%]
Edge 1
Edge 2
Edge 3
Edge 4
<= 100 m
<= 75 m
<= 50 m
<= 25 m
<= 10 m
Distance to Nearest Ground Structure

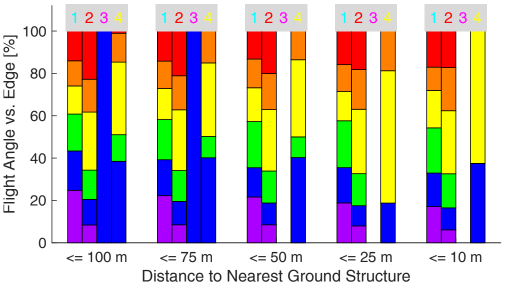
1 2 3 4
1 2 3 4
1 2 3 4
1 2 3 4
1 2 3 4
100
80
60
40
20
0
Flight Angle vs. Edge [%]
<= 100 m
<= 75 m
<= 50 m
<= 25 m
<= 10 m
Distance to Nearest Ground Structure

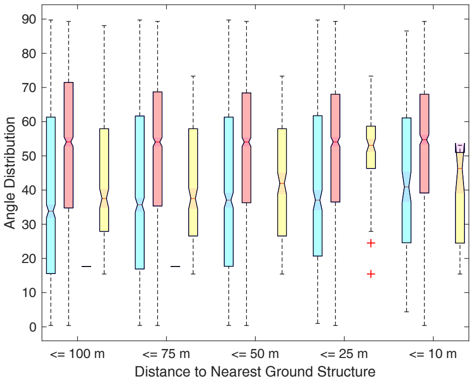
90
80
70
60
50
40
30
20
10
0
Angle Distribution
<= 100 m
<= 75 m
<= 50 m
<= 25 m
<= 10 m
Distance to Nearest Ground Structure

Bee C03

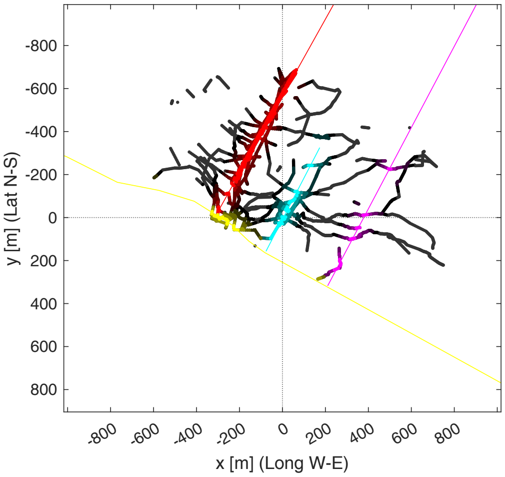

y [m] (Lat N-S)
x [m] (Long W-E)

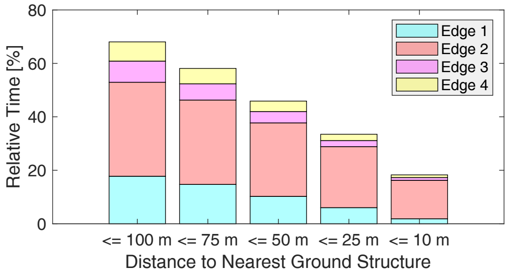

Relative Time [%]
Edge 1
Edge 2
Edge 3
Edge 4
<= 100 m
<= 75 m
<= 50 m
<= 25 m
<= 10 m
Distance to Nearest Ground Structure

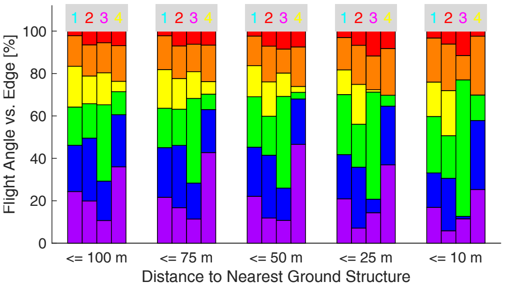

Flight Angle vs. Edge [%]
1 2 3 4
<= 100 m
<= 75 m
<= 50 m
<= 25 m
<= 10 m
Distance to Nearest Ground Structure

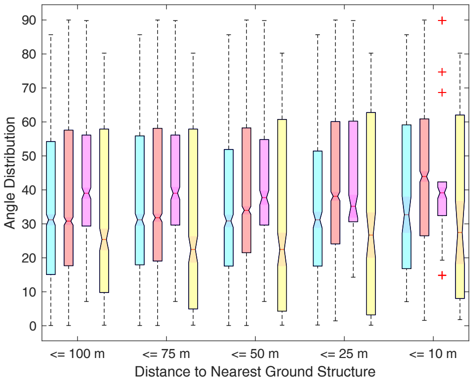

Angle Distribution
<= 100 m
<= 75 m
<= 50 m
<= 25 m
<= 10 m
Distance to Nearest Ground Structure

Bee C04

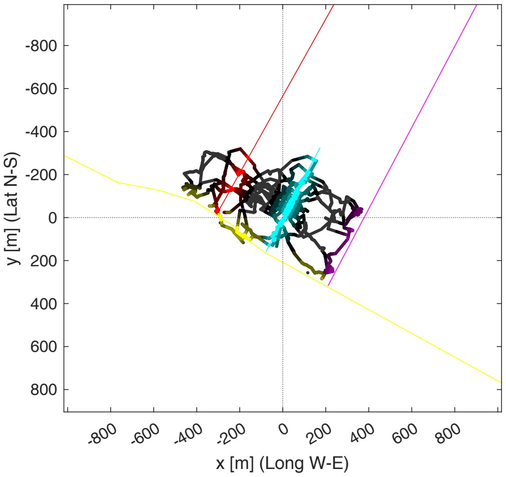
-800
-600
-400
-200
0
200
400
600
800
y [m] (Lat N-S)
-800
-600
-400
-200
0
200
400
600
800
x [m] (Long W-E)

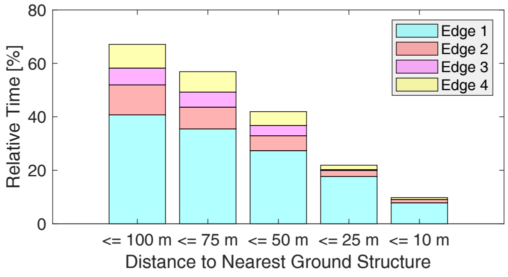
80
60
40
20
0
Relative Time [%]
Edge 1
Edge 2
Edge 3
Edge 4
<= 100 m
<= 75 m
<= 50 m
<= 25 m
<= 10 m
Distance to Nearest Ground Structure

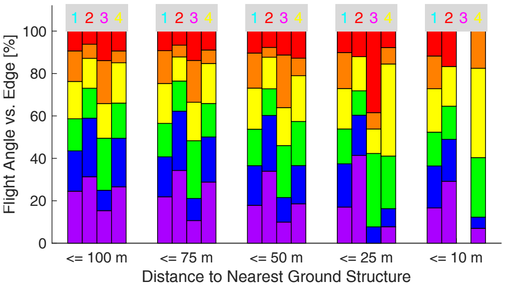
1 2 3 4
100
80
60
40
20
0
Flight Angle vs. Edge [%]
<= 100 m
<= 75 m
<= 50 m
<= 25 m
<= 10 m
Distance to Nearest Ground Structure

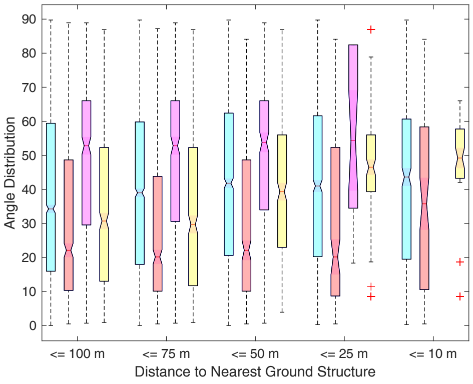
90
80
70
60
50
40
30
20
10
0
Angle Distribution
<= 100 m
<= 75 m
<= 50 m
<= 25 m
<= 10 m
Distance to Nearest Ground Structure

Bee C05

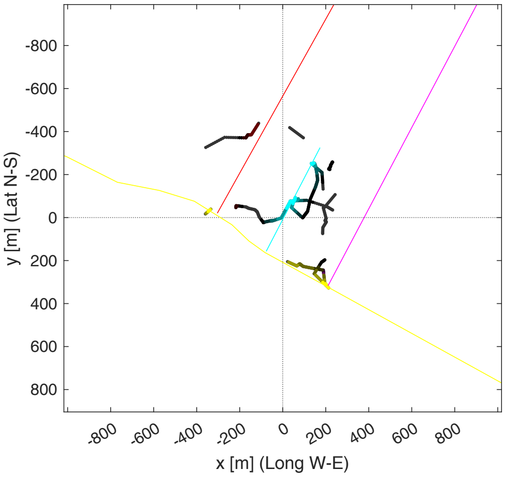
-800
-600
-400
-200
0
200
400
600
800
y [m] (Lat N-S)
-800
-600
-400
-200
0
200
400
600
800
x [m] (Long W-E)

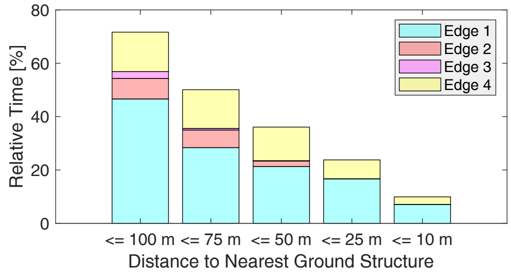
80
60
40
20
0
Relative Time [%]
Edge 1
Edge 2
Edge 3
Edge 4
<= 100 m
<= 75 m
<= 50 m
<= 25 m
<= 10 m
Distance to Nearest Ground Structure

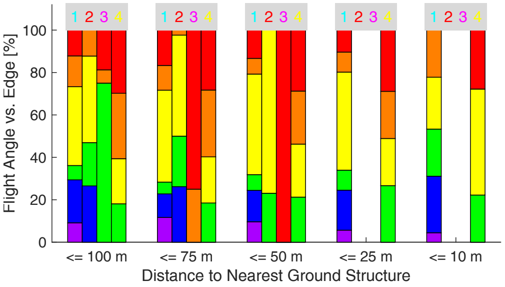
1 2 3 4
100
80
60
40
20
0
Flight Angle vs. Edge [%]
<= 100 m
<= 75 m
<= 50 m
<= 25 m
<= 10 m
Distance to Nearest Ground Structure

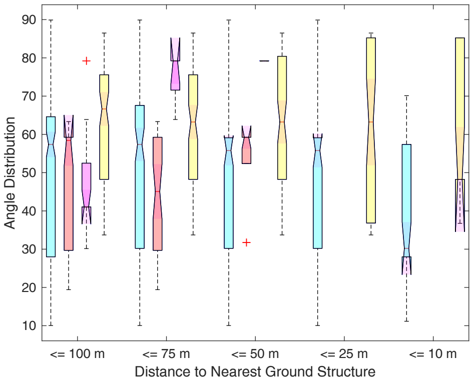
90
80
70
60
50
40
30
20
10
Angle Distribution
<= 100 m
<= 75 m
<= 50 m
<= 25 m
<= 10 m
Distance to Nearest Ground Structure

Bee D01

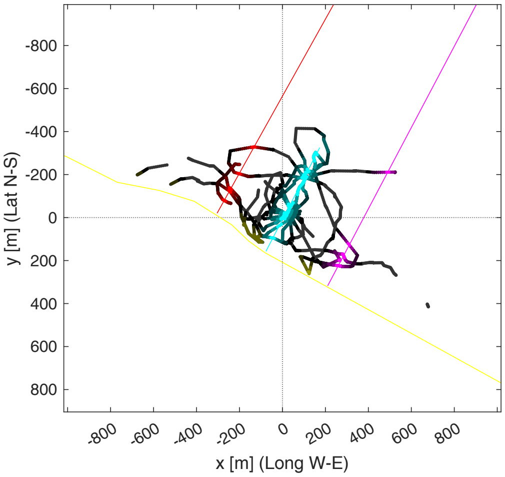

y [m] (Lat N-S)
x [m] (Long W-E)
-800
-600
-400
-200
0
200
400
600
800

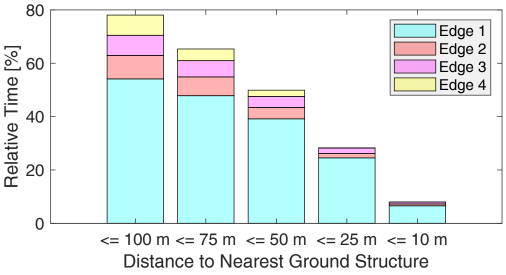

Relative Time [%]
Edge 1
Edge 2
Edge 3
Edge 4
<= 100 m
<= 75 m
<= 50 m
<= 25 m
<= 10 m
Distance to Nearest Ground Structure

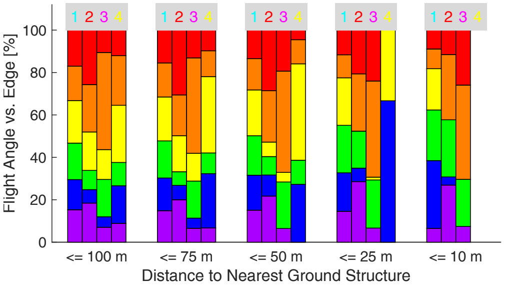

Flight Angle vs. Edge [%]
1 2 3 4
<= 100 m
<= 75 m
<= 50 m
<= 25 m
<= 10 m
Distance to Nearest Ground Structure

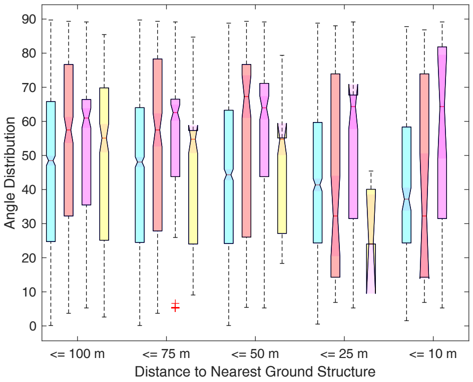

Angle Distribution
<= 100 m
<= 75 m
<= 50 m
<= 25 m
<= 10 m
Distance to Nearest Ground Structure

Bee D02

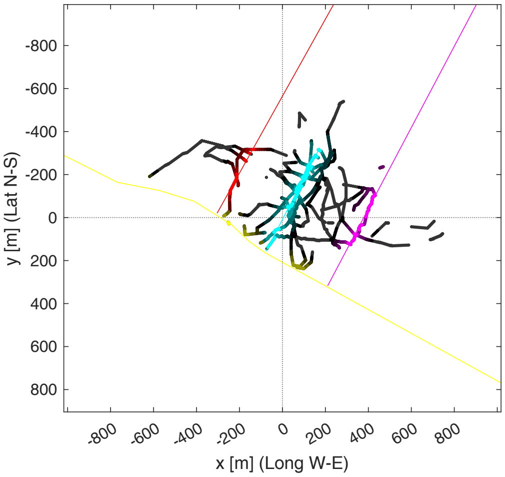
y [m] (Lat N-S)
x [m] (Long W-E)

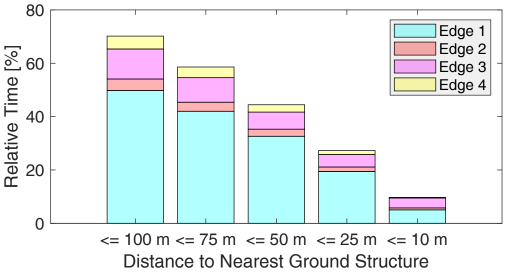
Relative Time [%]
Edge 1
Edge 2
Edge 3
Edge 4
<= 100 m
<= 75 m
<= 50 m
<= 25 m
<= 10 m
Distance to Nearest Ground Structure

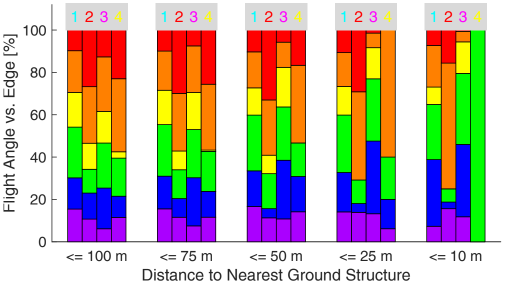
Flight Angle vs. Edge [%]
1 2 3 4
<= 100 m
<= 75 m
<= 50 m
<= 25 m
<= 10 m
Distance to Nearest Ground Structure

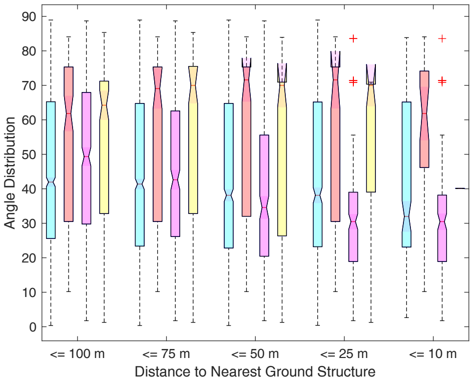
Angle Distribution
<= 100 m
<= 75 m
<= 50 m
<= 25 m
<= 10 m
Distance to Nearest Ground Structure

Bee D03

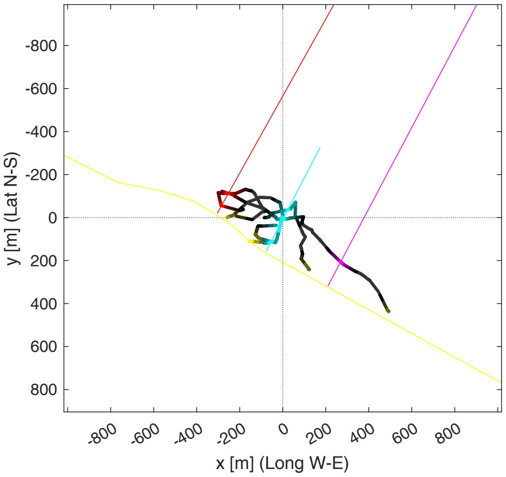
y [m] (Lat N-S)
x [m] (Long W-E)

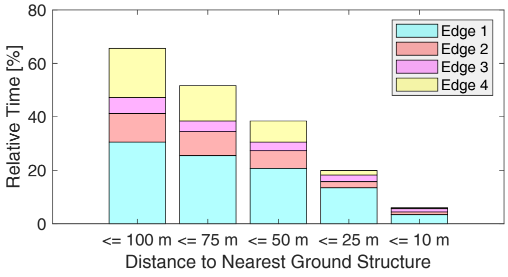
Edge 1
Edge 2
Edge 3
Edge 4
Relative Time [%]
<= 100 m
<= 75 m
<= 50 m
<= 25 m
<= 10 m
Distance to Nearest Ground Structure

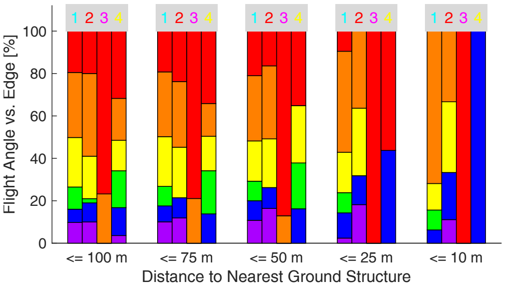
Flight Angle vs. Edge [%]
<= 100 m
<= 75 m
<= 50 m
<= 25 m
<= 10 m
Distance to Nearest Ground Structure

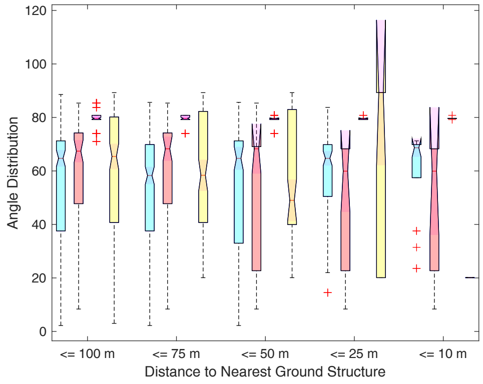
Angle Distribution
<= 100 m
<= 75 m
<= 50 m
<= 25 m
<= 10 m
Distance to Nearest Ground Structure

Bee D04

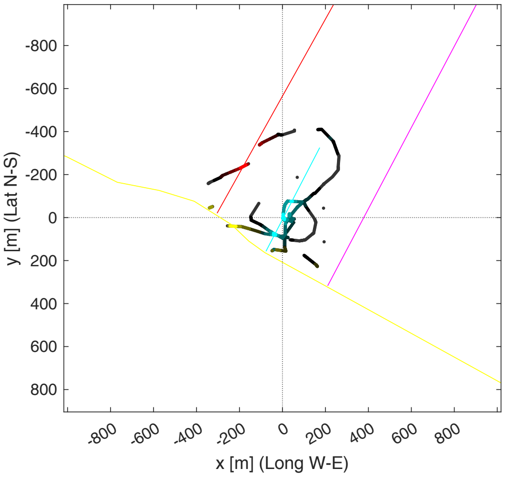
y [m] (Lat N-S)
x [m] (Long W-E)

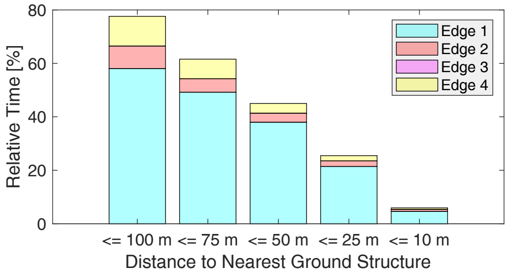
Edge 1
Edge 2
Edge 3
Edge 4
Relative Time [%]
Distance to Nearest Ground Structure

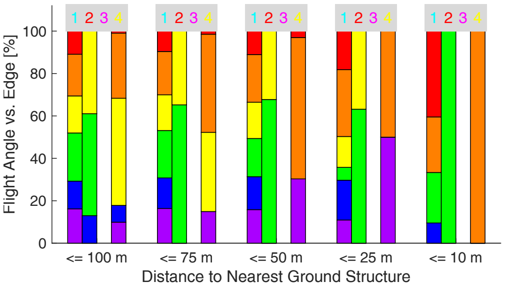
Flight Angle vs. Edge [%]
Distance to Nearest Ground Structure

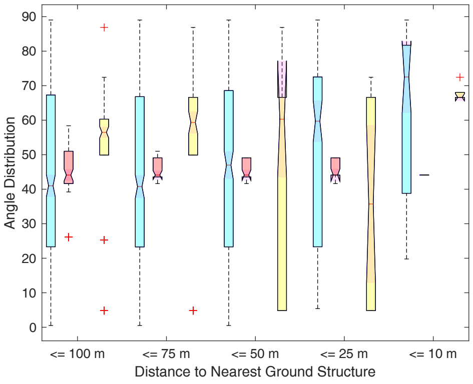
Angle Distribution
Distance to Nearest Ground Structure

Bee D05

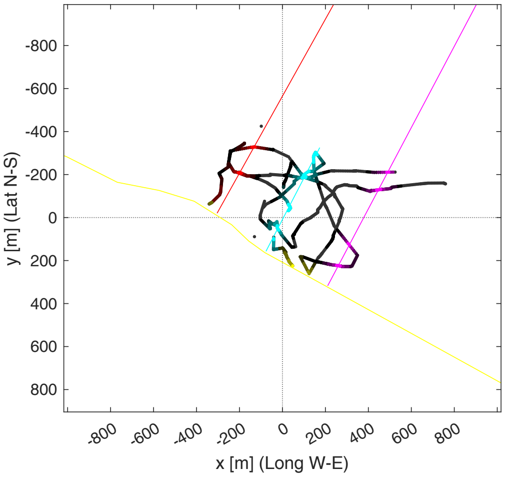
-800
-600
-400
-200
0
200
400
600
800
y [m] (Lat N-S)
-800
-600
-400
-200
0
200
400
600
800
x [m] (Long W-E)

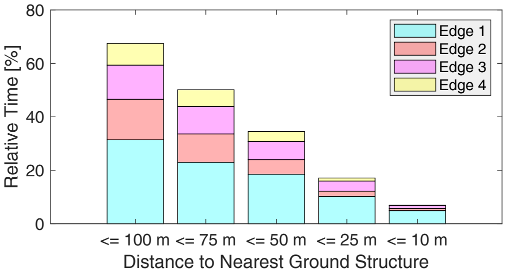
Edge 1
Edge 2
Edge 3
Edge 4
Relative Time [%]
80
60
40
20
0
<= 100 m
<= 75 m
<= 50 m
<= 25 m
<= 10 m
Distance to Nearest Ground Structure

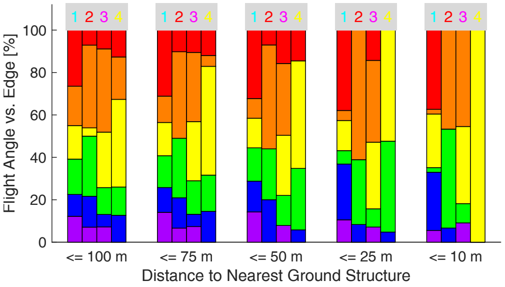
1 2 3 4
Flight Angle vs. Edge [%]
100
80
60
40
20
0
<= 100 m
<= 75 m
<= 50 m
<= 25 m
<= 10 m
Distance to Nearest Ground Structure

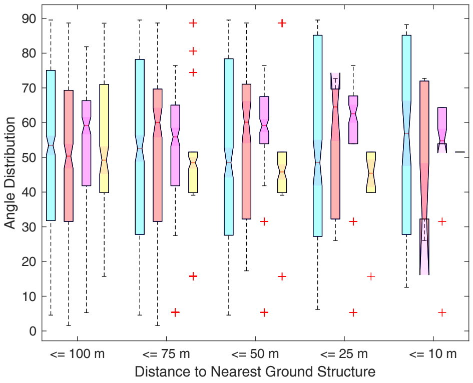
Angle Distribution
90
80
70
60
50
40
30
20
10
0
<= 100 m
<= 75 m
<= 50 m
<= 25 m
<= 10 m
Distance to Nearest Ground Structure

Bee D06

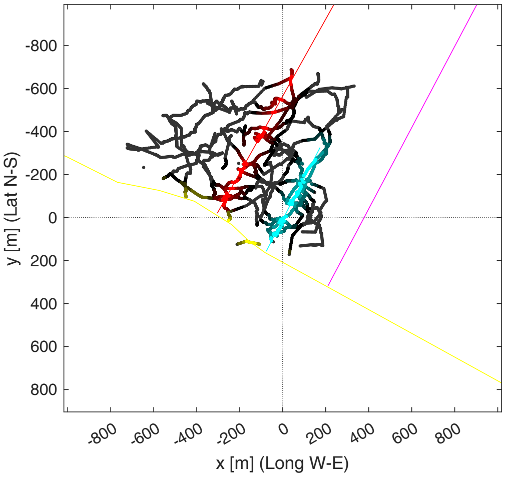
-800
-600
-400
-200
0
200
400
600
800
y [m] (Lat N-S)
-800 -600 -400 -200 0 200 400 600 800
x [m] (Long W-E)

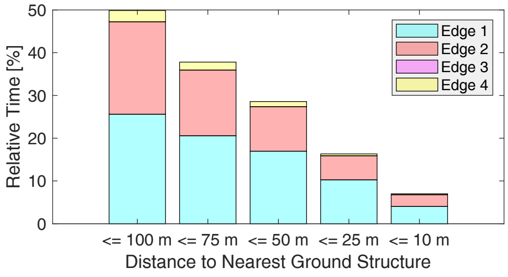
50
40
30
20
10
0
Relative Time [%]
Edge 1
Edge 2
Edge 3
Edge 4
<= 100 m <= 75 m <= 50 m <= 25 m <= 10 m
Distance to Nearest Ground Structure

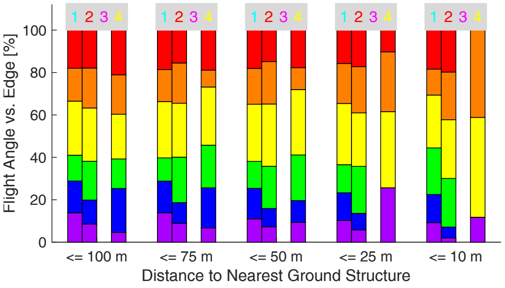
1 2 3 4
100
80
60
40
20
0
Flight Angle vs. Edge [%]
<= 100 m <= 75 m <= 50 m <= 25 m <= 10 m
Distance to Nearest Ground Structure

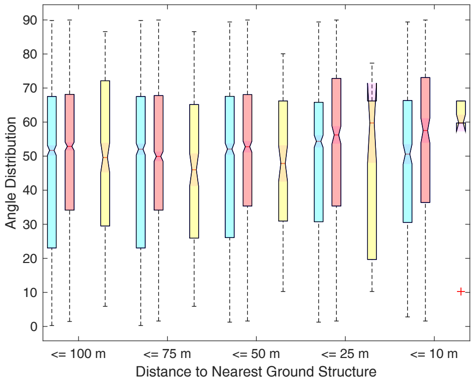
90
80
70
60
50
40
30
20
10
0
Angle Distribution
<= 100 m <= 75 m <= 50 m <= 25 m <= 10 m
Distance to Nearest Ground Structure

Bee D07

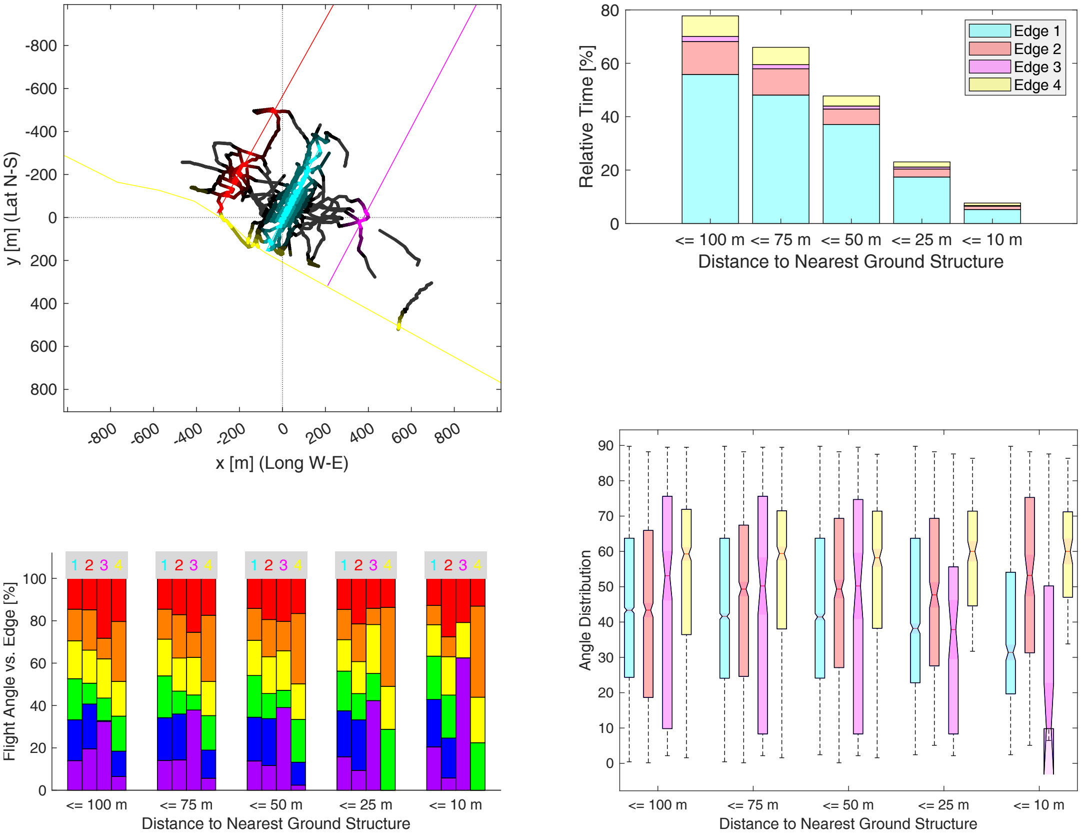
y [m] (Lat N-S)
x [m] (Long W-E)
Relative Time [%]
Edge 1
Edge 2
Edge 3
Edge 4
<= 100 m
<= 75 m
<= 50 m
<= 25 m
<= 10 m
Distance to Nearest Ground Structure
Flight Angle vs. Edge [%]
Angle Distribution

Bee D08

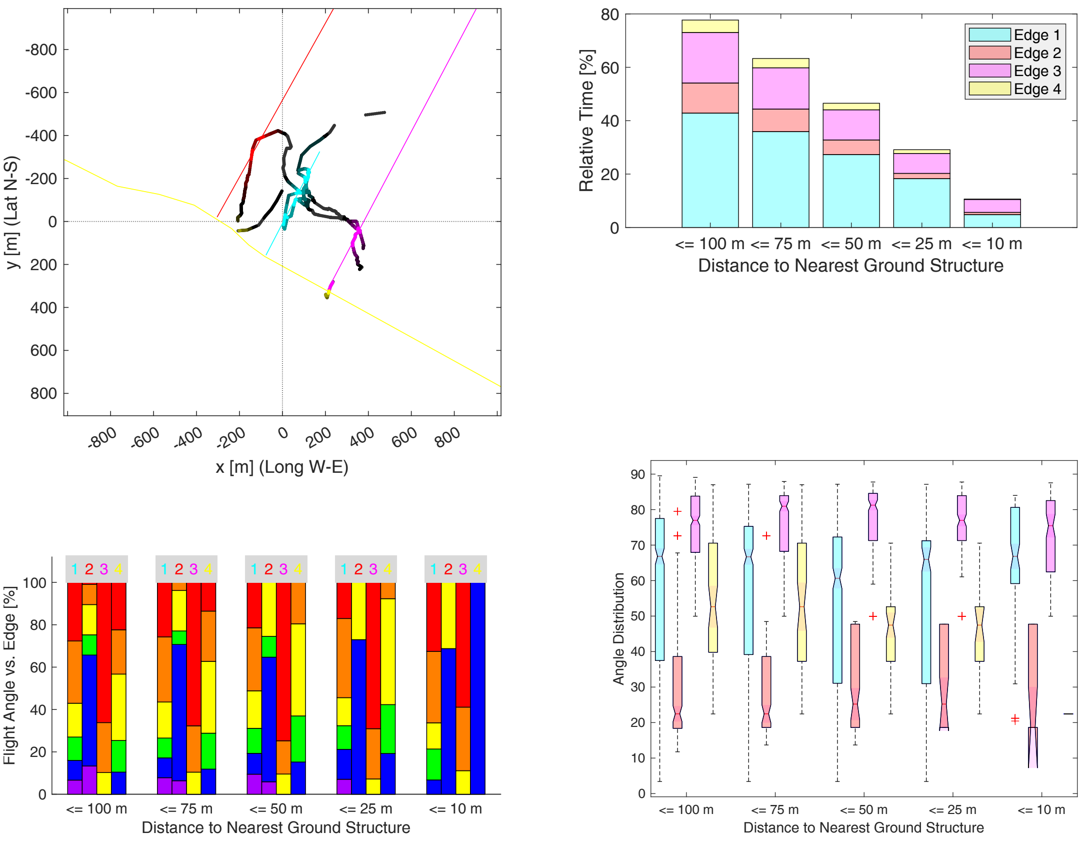
y [m] (Lat N-S)
x [m] (Long W-E)
Relative Time [%]
Edge 1
Edge 2
Edge 3
Edge 4
<= 100 m
<= 75 m
<= 50 m
<= 25 m
<= 10 m
Distance to Nearest Ground Structure
Flight Angle vs. Edge [%]
Angle Distribution

Bee D09

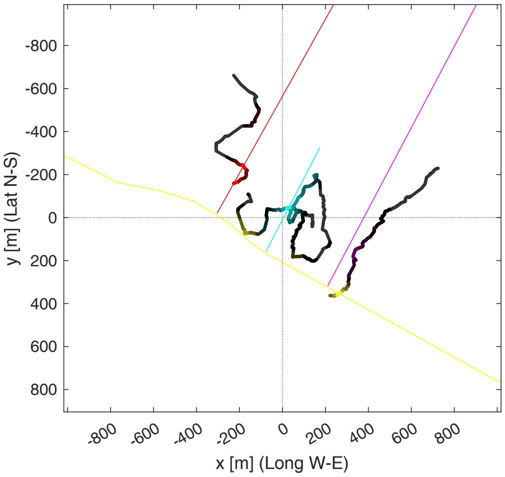
y [m] (Lat N-S)
x [m] (Long W-E)

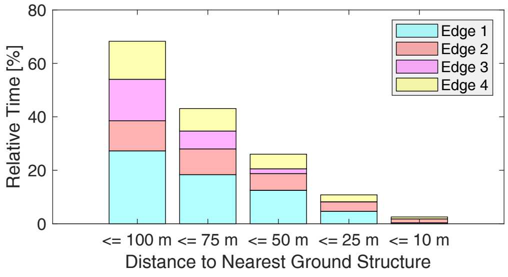
Edge 1
Edge 2
Edge 3
Edge 4
Relative Time [%]
<= 100 m
<= 75 m
<= 50 m
<= 25 m
<= 10 m
Distance to Nearest Ground Structure

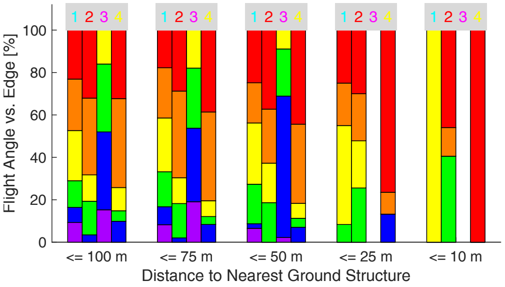
Flight Angle vs. Edge [%]
<= 100 m
<= 75 m
<= 50 m
<= 25 m
<= 10 m
Distance to Nearest Ground Structure

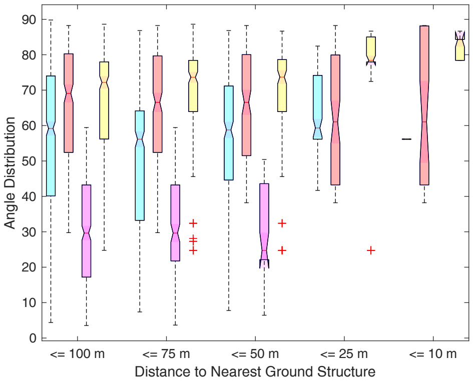
Angle Distribution
<= 100 m
<= 75 m
<= 50 m
<= 25 m
<= 10 m
Distance to Nearest Ground Structure

Bee D10

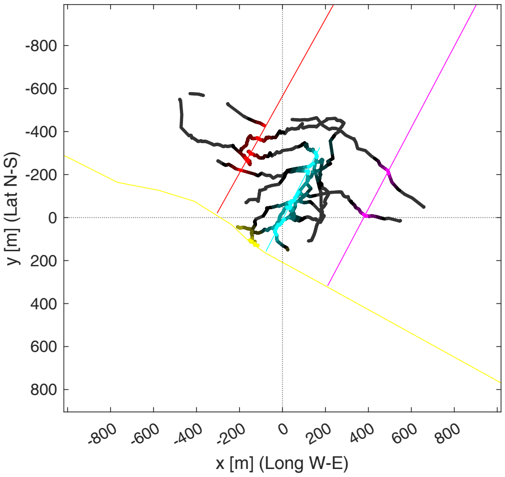

y [m] (Lat N-S)
x [m] (Long W-E)
-800
-600
-400
-200
0
200
400
600
800

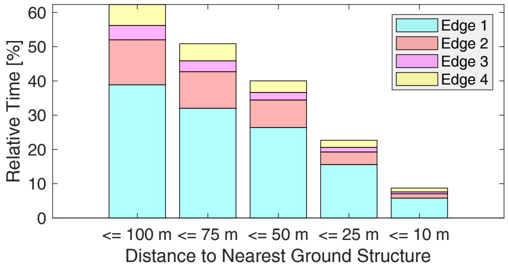

Relative Time [%]
Edge 1
Edge 2
Edge 3
Edge 4
0
10
20
30
40
50
60
<= 100 m
<= 75 m
<= 50 m
<= 25 m
<= 10 m
Distance to Nearest Ground Structure

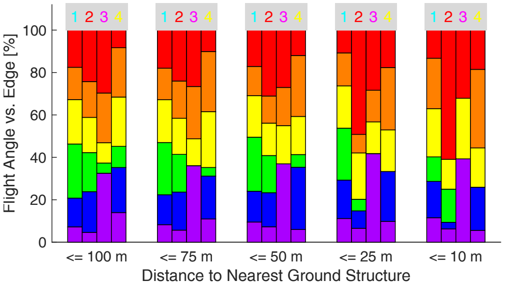

Flight Angle vs. Edge [%]
1 2 3 4
0
20
40
60
80
100
<= 100 m
<= 75 m
<= 50 m
<= 25 m
<= 10 m
Distance to Nearest Ground Structure

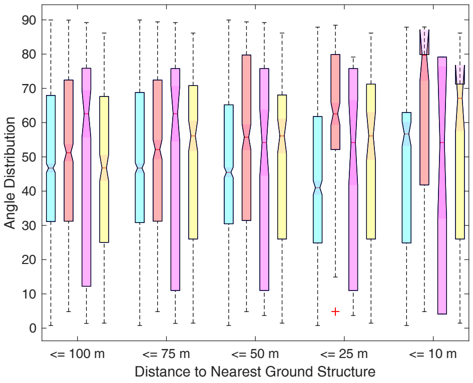

Angle Distribution
0
10
20
30
40
50
60
70
80
90
<= 100 m
<= 75 m
<= 50 m
<= 25 m
<= 10 m
Distance to Nearest Ground Structure

Bee D11

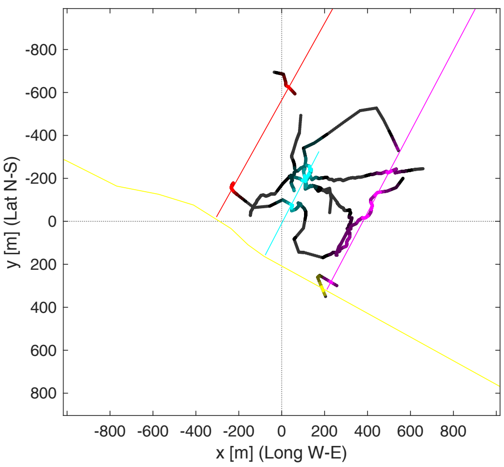
y [m] (Lat N-S)
x [m] (Long W-E)

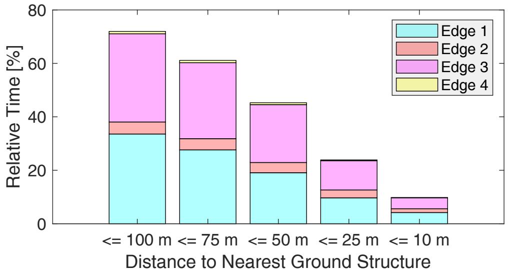
Edge 1
Edge 2
Edge 3
Edge 4
Relative Time [%]
<= 100 m <= 75 m <= 50 m <= 25 m <= 10 m
Distance to Nearest Ground Structure

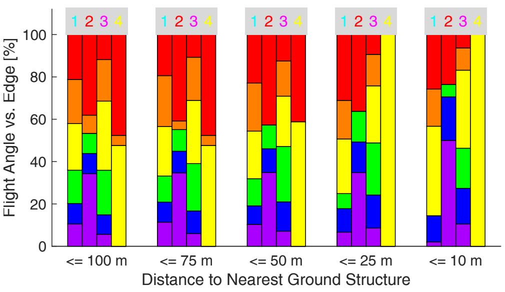
Flight Angle vs. Edge [%]
<= 100 m
<= 75 m
<= 50 m
<= 25 m
<= 10 m
Distance to Nearest Ground Structure

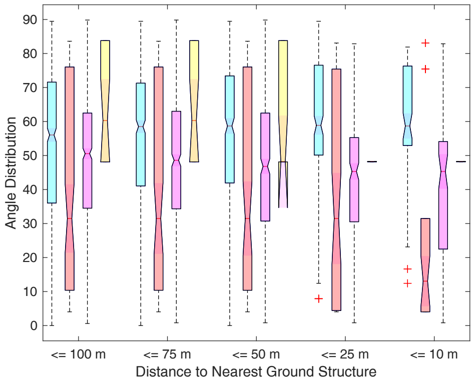
Angle Distribution
<= 100 m
<= 75 m
<= 50 m
<= 25 m
<= 10 m
Distance to Nearest Ground Structure

Bee D12

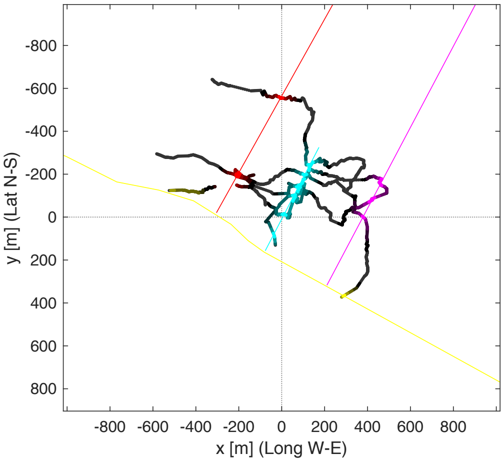
y [m] (Lat N-S)
x [m] (Long W-E)
-800
-600
-400
-200
0
200
400
600
800

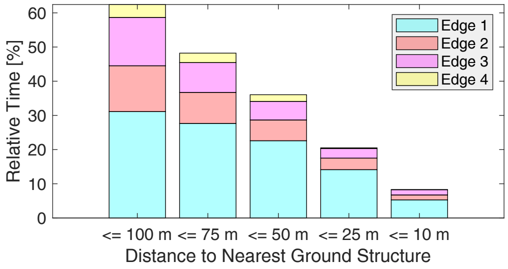
Relative Time [%]
Edge 1
Edge 2
Edge 3
Edge 4
<= 100 m
<= 75 m
<= 50 m
<= 25 m
<= 10 m
Distance to Nearest Ground Structure

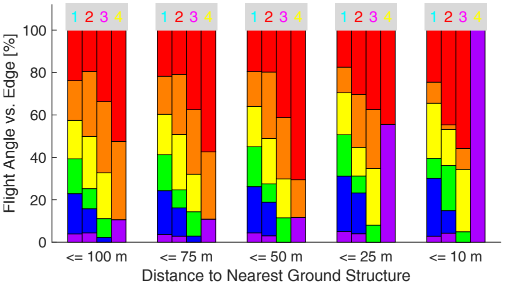
Flight Angle vs. Edge [%]
1 2 3 4
<= 100 m
<= 75 m
<= 50 m
<= 25 m
<= 10 m
Distance to Nearest Ground Structure

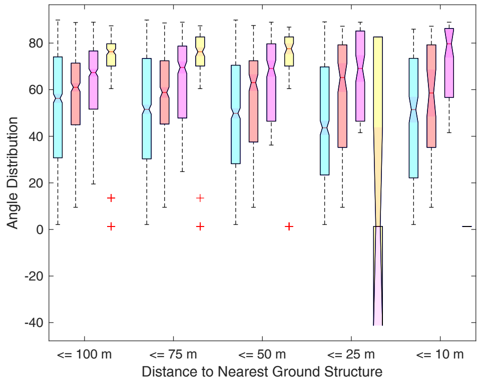
Angle Distribution
<= 100 m
<= 75 m
<= 50 m
<= 25 m
<= 10 m
Distance to Nearest Ground Structure

Bee D13

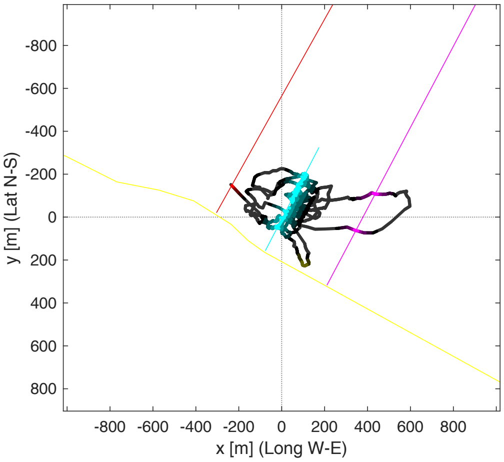
y [m] (Lat N-S)
x [m] (Long W-E)
-800
-600
-400
-200
0
200
400
600
800

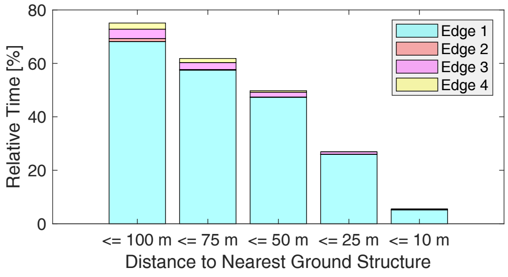
Edge 1
Edge 2
Edge 3
Edge 4
Relative Time [%]
<= 100 m
<= 75 m
<= 50 m
<= 25 m
<= 10 m
Distance to Nearest Ground Structure

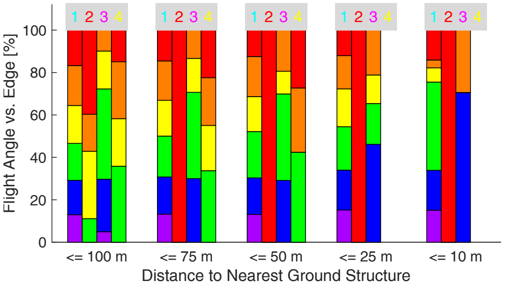
1 2 3 4
Flight Angle vs. Edge [%]
<= 100 m
<= 75 m
<= 50 m
<= 25 m
<= 10 m
Distance to Nearest Ground Structure

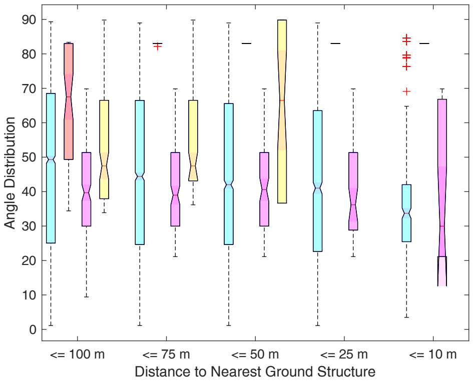
Angle Distribution
<= 100 m
<= 75 m
<= 50 m
<= 25 m
<= 10 m
Distance to Nearest Ground Structure

Bee E01

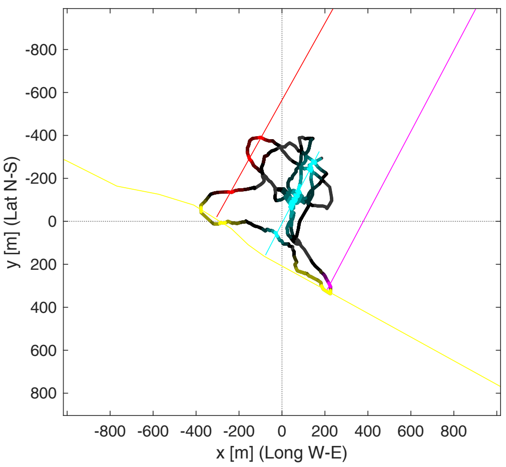
y [m] (Lat N-S)
x [m] (Long W-E)

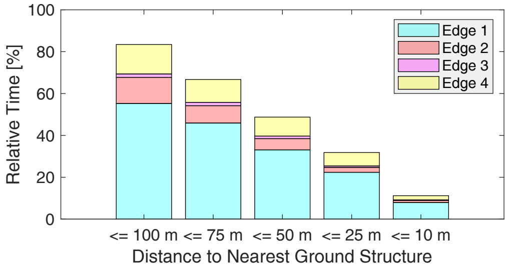
Edge 1
Edge 2
Edge 3
Edge 4
Relative Time [%]
<= 100 m <= 75 m <= 50 m <= 25 m <= 10 m
Distance to Nearest Ground Structure

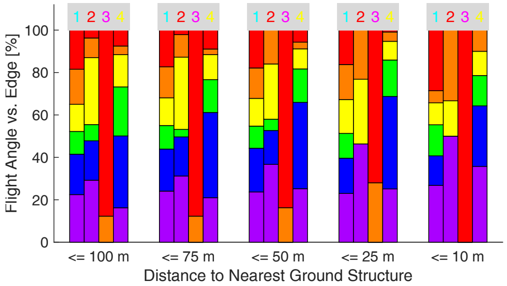
Flight Angle vs. Edge [%]
<= 100 m
<= 75 m
<= 50 m
<= 25 m
<= 10 m
Distance to Nearest Ground Structure

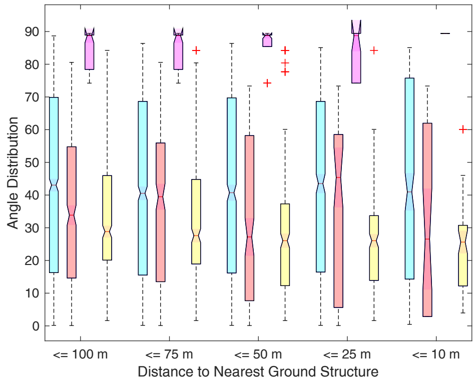
Angle Distribution
<= 100 m
<= 75 m
<= 50 m
<= 25 m
<= 10 m
Distance to Nearest Ground Structure

Bee E02

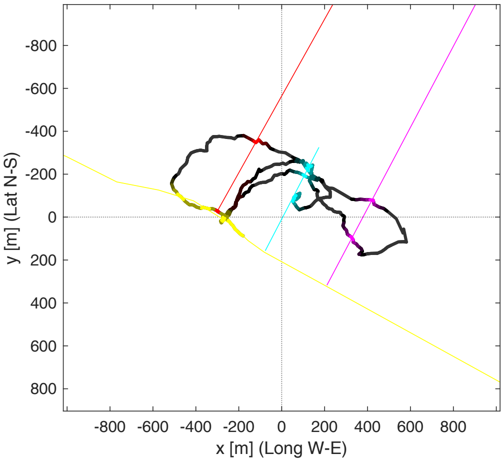
y [m] (Lat N-S)
x [m] (Long W-E)

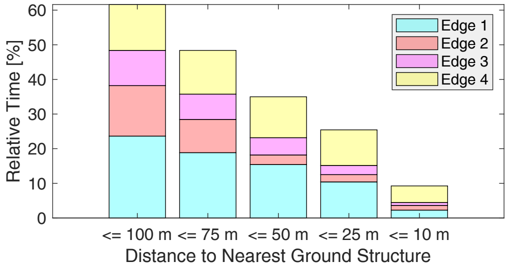
Relative Time [%]
Edge 1
Edge 2
Edge 3
Edge 4
<= 100 m
<= 75 m
<= 50 m
<= 25 m
<= 10 m
Distance to Nearest Ground Structure

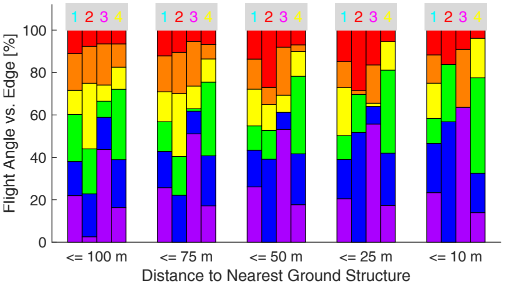
Flight Angle vs. Edge [%]
<= 100 m
<= 75 m
<= 50 m
<= 25 m
<= 10 m
Distance to Nearest Ground Structure

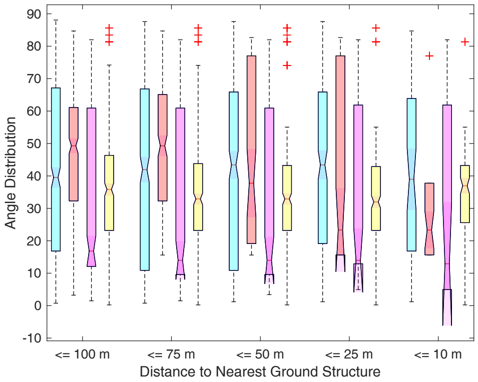
Angle Distribution
<= 100 m
<= 75 m
<= 50 m
<= 25 m
<= 10 m
Distance to Nearest Ground Structure

Bee E03

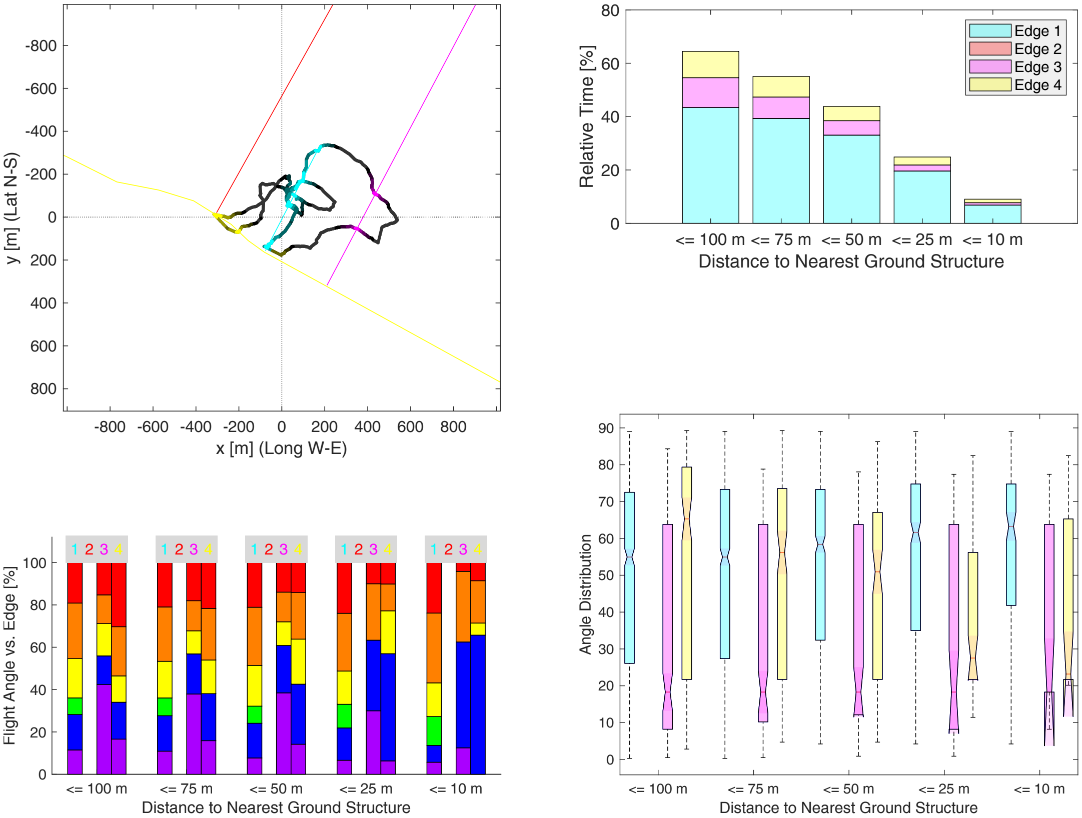
y [m] (Lat N-S)
x [m] (Long W-E)
Relative Time [%]
Edge 1
Edge 2
Edge 3
Edge 4
<= 100 m
<= 75 m
<= 50 m
<= 25 m
<= 10 m
Distance to Nearest Ground Structure
Flight Angle vs. Edge [%]
1 2 3 4
Angle Distribution

Bee E04

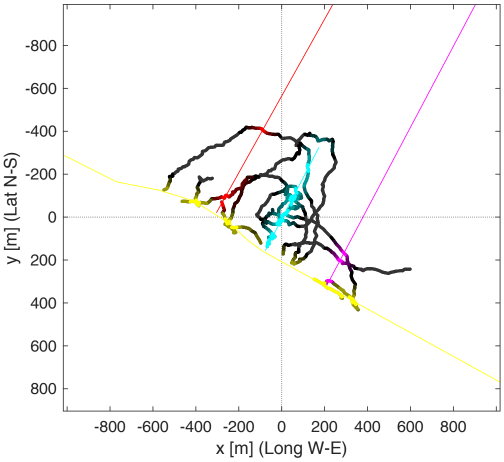
y [m] (Lat N-S)
x [m] (Long W-E)
-800
-600
-400
-200
0
200
400
600
800
-800 -600 -400 -200 0 200 400 600 800

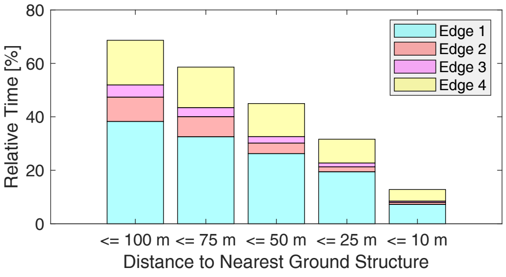
Relative Time [%]
Edge 1
Edge 2
Edge 3
Edge 4
0
20
40
60
80
<= 100 m <= 75 m <= 50 m <= 25 m <= 10 m
Distance to Nearest Ground Structure

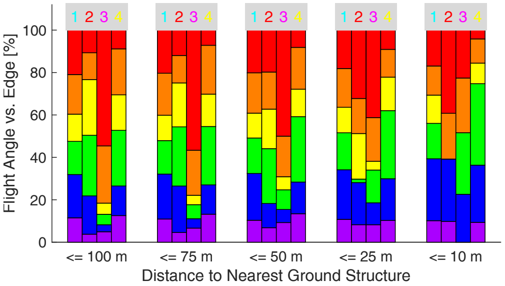
Flight Angle vs. Edge [%]
1 2 3 4
0
20
40
60
80
100
<= 100 m <= 75 m <= 50 m <= 25 m <= 10 m
Distance to Nearest Ground Structure

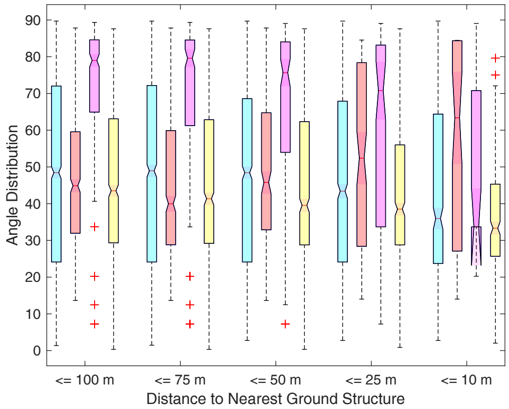
Angle Distribution
0
10
20
30
40
50
60
70
80
90
<= 100 m <= 75 m <= 50 m <= 25 m <= 10 m
Distance to Nearest Ground Structure

Bee E05

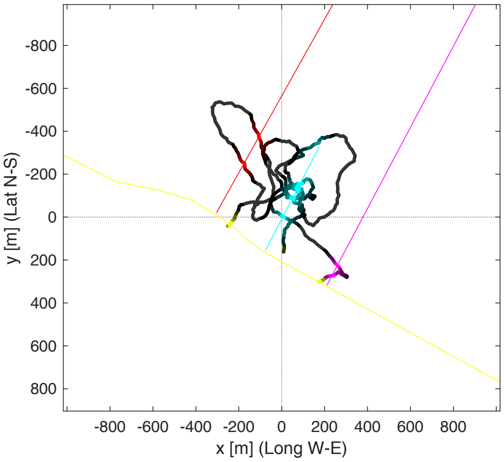
y [m] (Lat N-S)
x [m] (Long W-E)

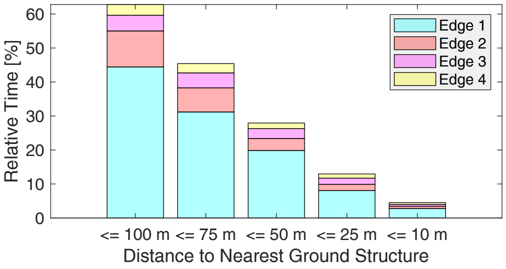
Relative Time [%]
Edge 1
Edge 2
Edge 3
Edge 4
<= 100 m
<= 75 m
<= 50 m
<= 25 m
<= 10 m
Distance to Nearest Ground Structure

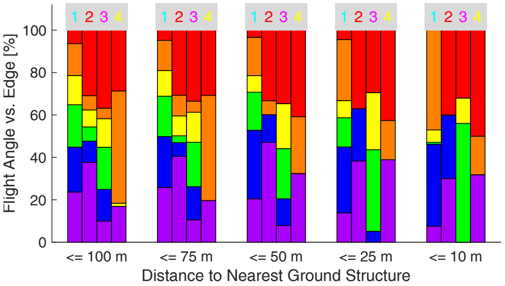
Flight Angle vs. Edge [%]
1 2 3 4
<= 100 m
<= 75 m
<= 50 m
<= 25 m
<= 10 m
Distance to Nearest Ground Structure

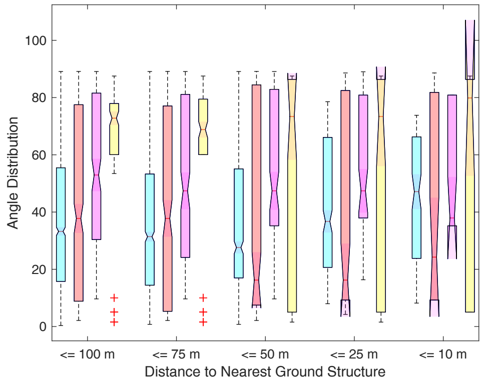
Angle Distribution
<= 100 m
<= 75 m
<= 50 m
<= 25 m
<= 10 m
Distance to Nearest Ground Structure

Bee E06

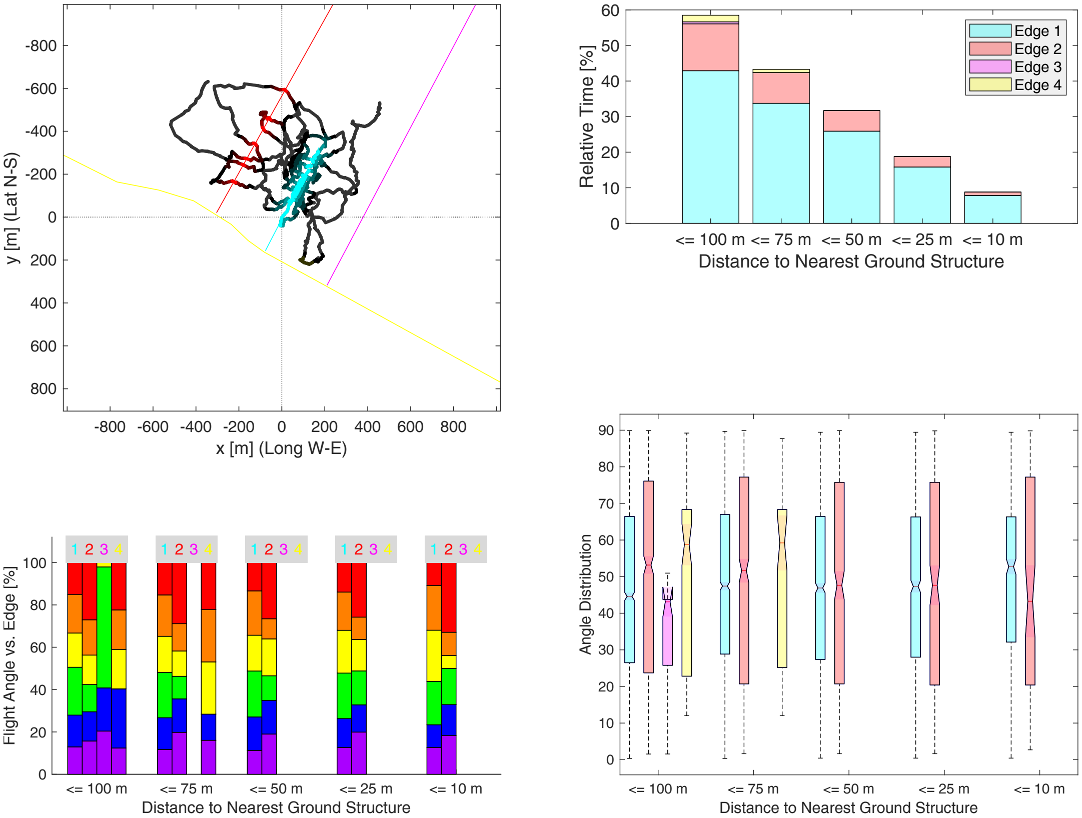
y [m] (Lat N-S)
x [m] (Long W-E)
Relative Time [%]
Edge 1
Edge 2
Edge 3
Edge 4
Distance to Nearest Ground Structure
Flight Angle vs. Edge [%]
Angle Distribution
<= 100 m
<= 75 m
<= 50 m
<= 25 m
<= 10 m

Bee E07

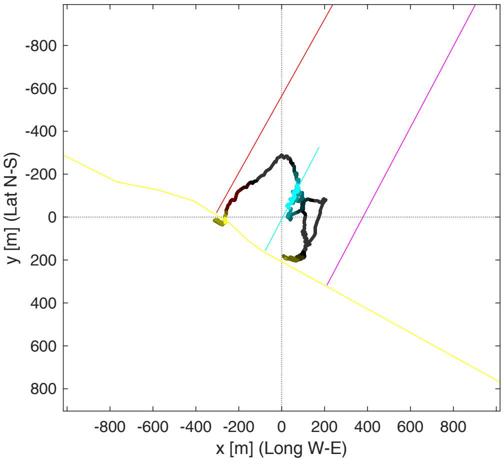
y [m] (Lat N-S)
x [m] (Long W-E)

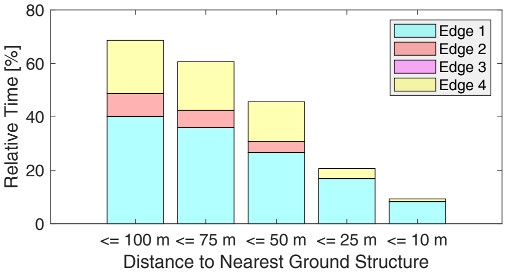
Edge 1
Edge 2
Edge 3
Edge 4
Relative Time [%]
<= 100 m <= 75 m <= 50 m <= 25 m <= 10 m
Distance to Nearest Ground Structure

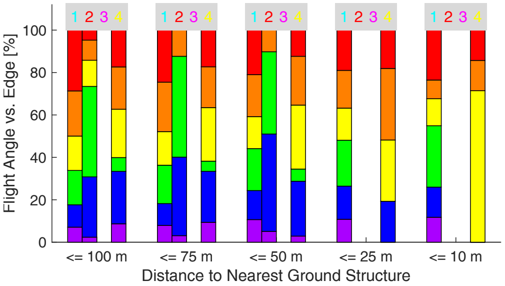
1 2 3 4
Flight Angle vs. Edge [%]
<= 100 m
<= 75 m
<= 50 m
<= 25 m
<= 10 m
Distance to Nearest Ground Structure

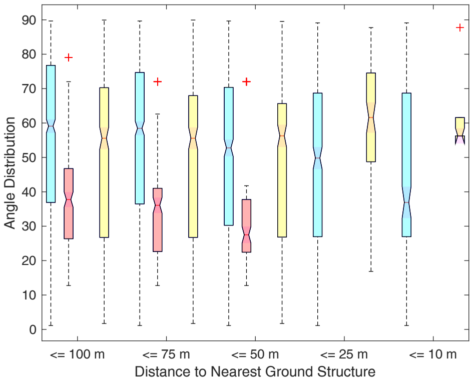
Angle Distribution
<= 100 m
<= 75 m
<= 50 m
<= 25 m
<= 10 m
Distance to Nearest Ground Structure

Bee R01

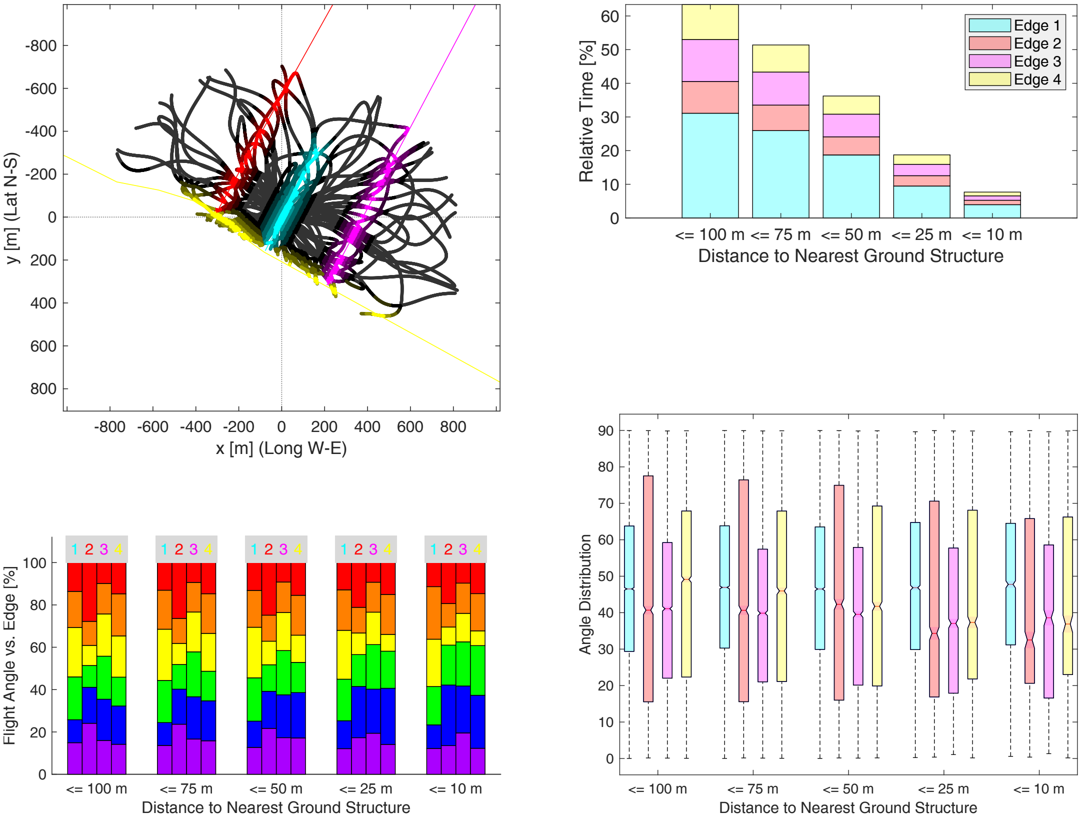
y [m] (Lat N-S)
x [m] (Long W-E)
Relative Time [%]
Edge 1
Edge 2
Edge 3
Edge 4
<= 100 m
<= 75 m
<= 50 m
<= 25 m
<= 10 m
Distance to Nearest Ground Structure
Flight Angle vs. Edge [%]
Angle Distribution

Bee R02

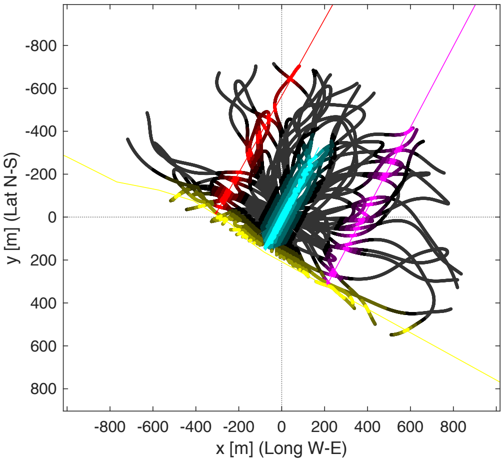
-800
-600
-400
-200
0
200
400
600
800
y [m] (Lat N-S)
-800 -600 -400 -200 0 200 400 600 800
x [m] (Long W-E)

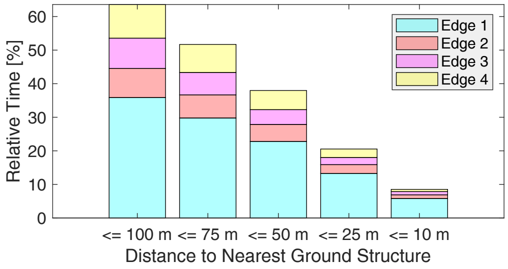
Edge 1
Edge 2
Edge 3
Edge 4
Relative Time [%]
0
10
20
30
40
50
60
<= 100 m <= 75 m <= 50 m <= 25 m <= 10 m
Distance to Nearest Ground Structure

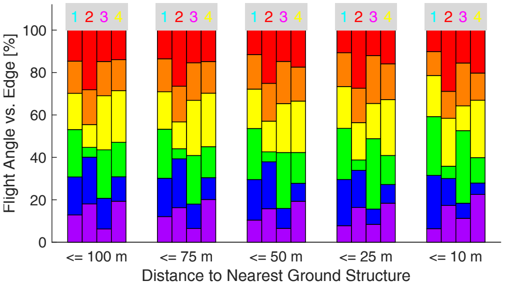
1 2 3 4
Flight Angle vs. Edge [%]
0
20
40
60
80
100
<= 100 m
<= 75 m
<= 50 m
<= 25 m
<= 10 m
Distance to Nearest Ground Structure

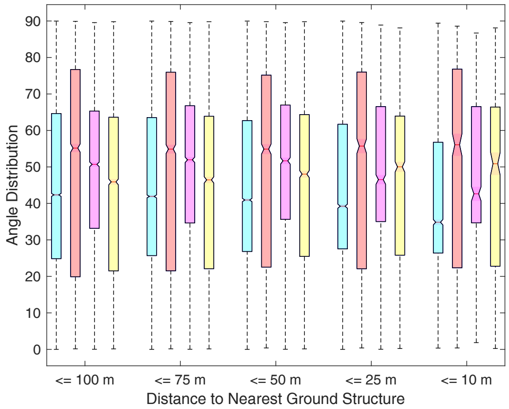
Angle Distribution
0
10
20
30
40
50
60
70
80
90
<= 100 m
<= 75 m
<= 50 m
<= 25 m
<= 10 m
Distance to Nearest Ground Structure

Bee R03

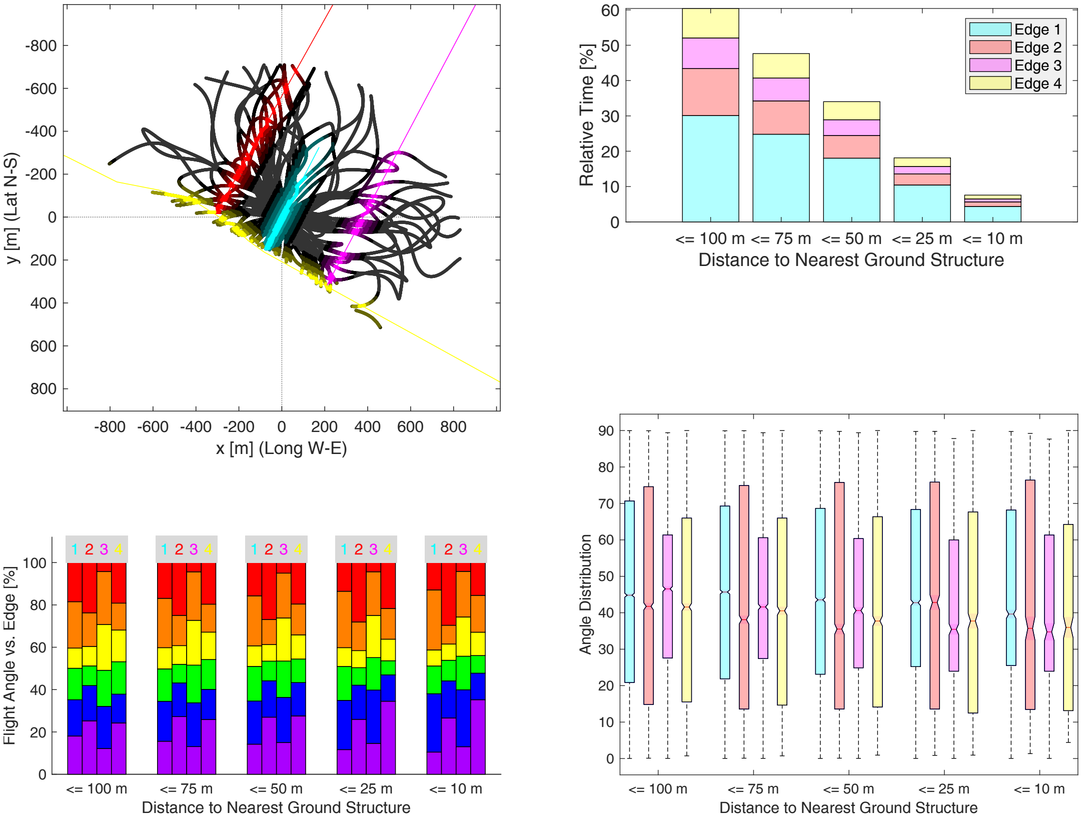
y [m] (Lat N-S)
x [m] (Long W-E)
-800
-600
-400
-200
0
200
400
600
800
Relative Time [%]
Edge 1
Edge 2
Edge 3
Edge 4
<= 100 m
<= 75 m
<= 50 m
<= 25 m
<= 10 m
Distance to Nearest Ground Structure
Flight Angle vs. Edge [%]
1 2 3 4
Angle Distribution

Bee R04

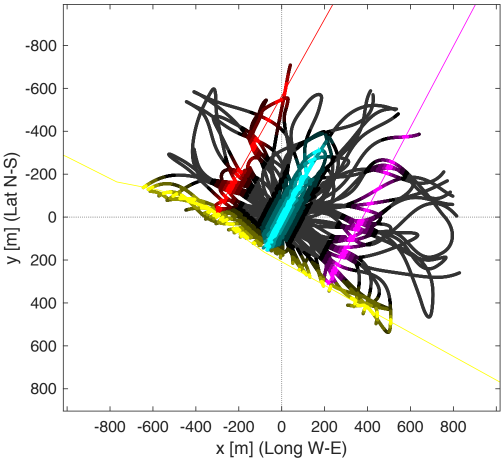
-800
-600
-400
-200
0
200
400
600
800
y [m] (Lat N-S)
-800 -600 -400 -200 0 200 400 600 800
x [m] (Long W-E)

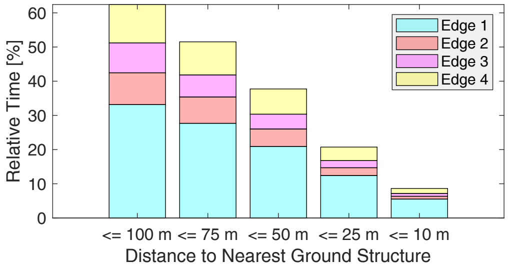
Relative Time [%]
0
10
20
30
40
50
60
Edge 1
Edge 2
Edge 3
Edge 4
<= 100 m <= 75 m <= 50 m <= 25 m <= 10 m
Distance to Nearest Ground Structure

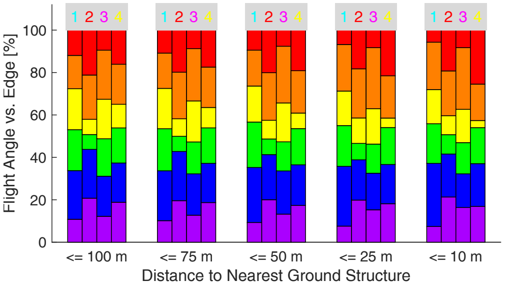
Flight Angle vs. Edge [%]
0
20
40
60
80
100
1 2 3 4
<= 100 m <= 75 m <= 50 m <= 25 m <= 10 m
Distance to Nearest Ground Structure

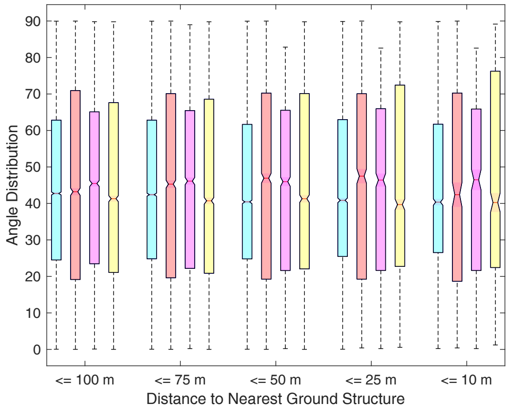
Angle Distribution
0
10
20
30
40
50
60
70
80
90
<= 100 m <= 75 m <= 50 m <= 25 m <= 10 m
Distance to Nearest Ground Structure

Bee R05

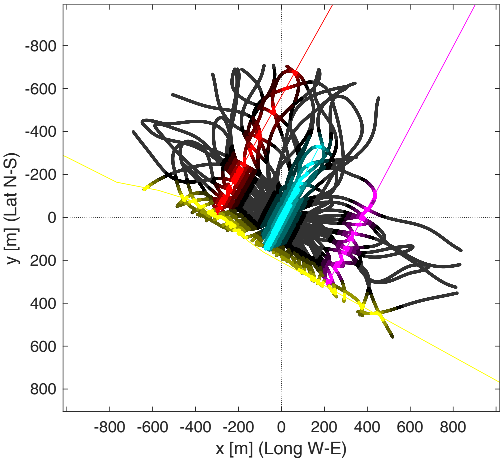
y [m] (Lat N-S)
x [m] (Long W-E)
-800
-600
-400
-200
0
200
400
600
800

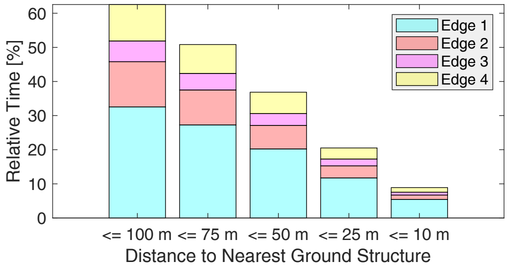
Relative Time [%]
Edge 1
Edge 2
Edge 3
Edge 4
<= 100 m
<= 75 m
<= 50 m
<= 25 m
<= 10 m
Distance to Nearest Ground Structure

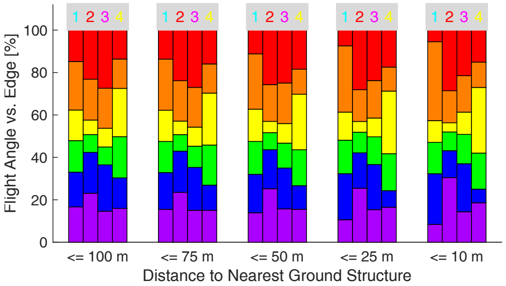
Flight Angle vs. Edge [%]
1 2 3 4
<= 100 m
<= 75 m
<= 50 m
<= 25 m
<= 10 m
Distance to Nearest Ground Structure

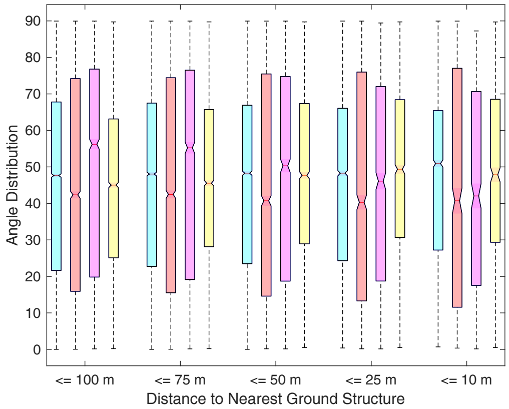
Angle Distribution
<= 100 m
<= 75 m
<= 50 m
<= 25 m
<= 10 m
Distance to Nearest Ground Structure

Bee R06

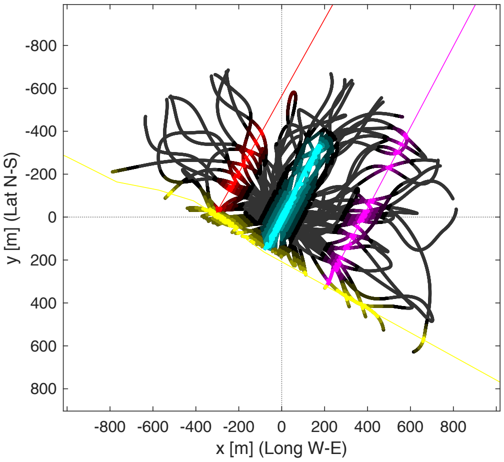
-800
-600
-400
-200
0
200
400
600
800
y [m] (Lat N-S)
-800 -600 -400 -200 0 200 400 600 800
x [m] (Long W-E)

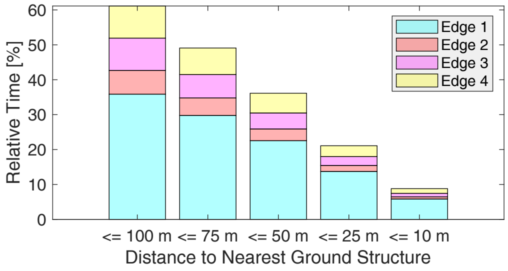
Edge 1
Edge 2
Edge 3
Edge 4
Relative Time [%]
0
10
20
30
40
50
60
<= 100 m
<= 75 m
<= 50 m
<= 25 m
<= 10 m
Distance to Nearest Ground Structure

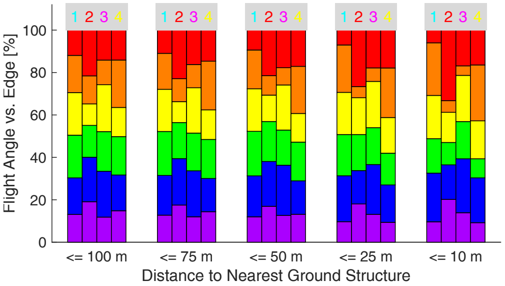
1 2 3 4
Flight Angle vs. Edge [%]
0
20
40
60
80
100
<= 100 m
<= 75 m
<= 50 m
<= 25 m
<= 10 m
Distance to Nearest Ground Structure

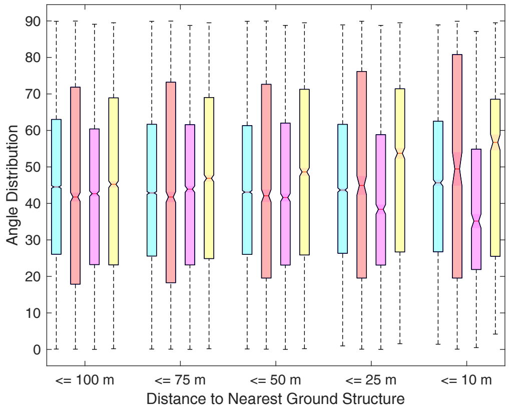
Angle Distribution
0
10
20
30
40
50
60
70
80
90
<= 100 m
<= 75 m
<= 50 m
<= 25 m
<= 10 m
Distance to Nearest Ground Structure

Bee R07

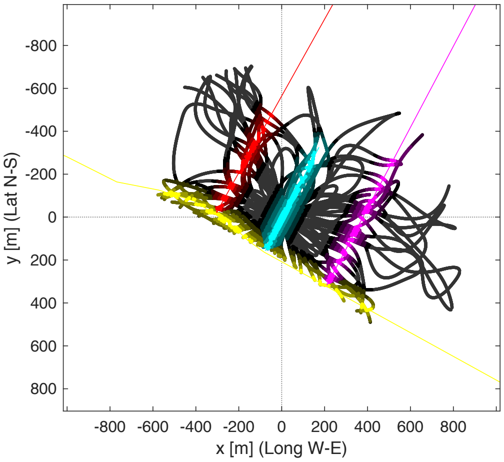
y [m] (Lat N-S)
x [m] (Long W-E)

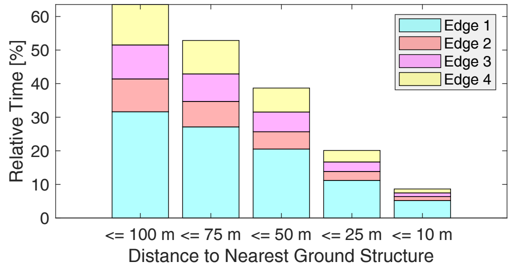
Relative Time [%]
Edge 1
Edge 2
Edge 3
Edge 4
<= 100 m
<= 75 m
<= 50 m
<= 25 m
<= 10 m
Distance to Nearest Ground Structure

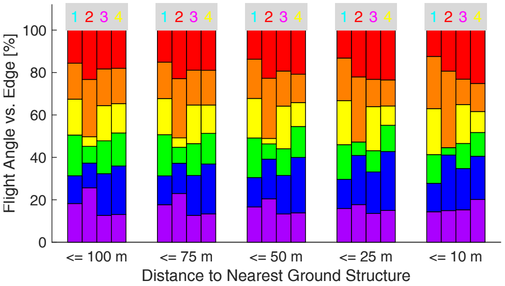
Flight Angle vs. Edge [%]
1 2 3 4
<= 100 m
<= 75 m
<= 50 m
<= 25 m
<= 10 m
Distance to Nearest Ground Structure

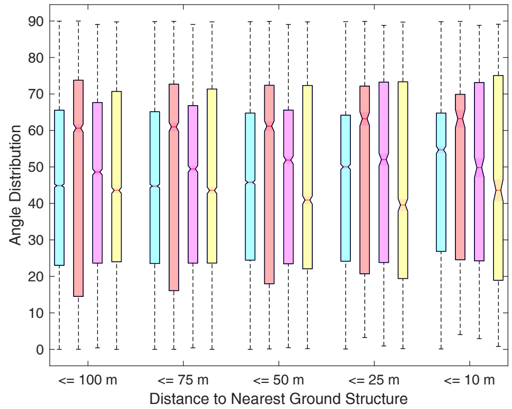
Angle Distribution
<= 100 m
<= 75 m
<= 50 m
<= 25 m
<= 10 m
Distance to Nearest Ground Structure

Bee R08

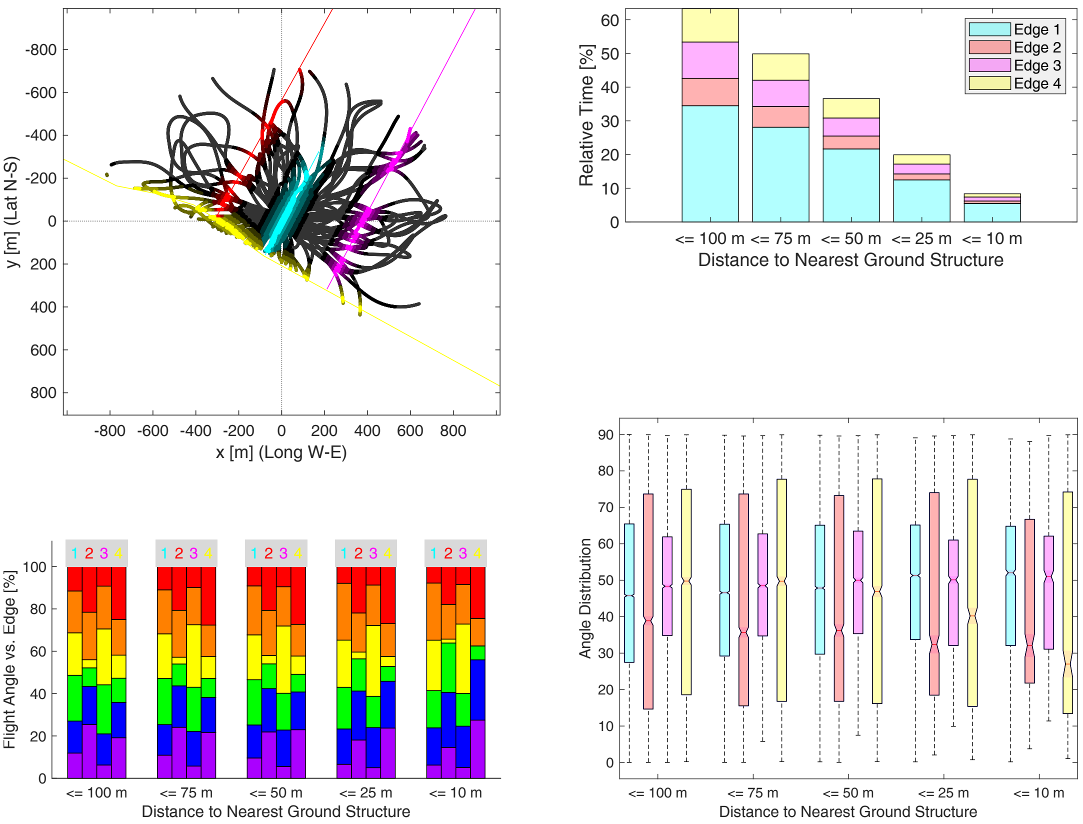
y [m] (Lat N-S)
x [m] (Long W-E)
-800
-600
-400
-200
0
200
400
600
800
Relative Time [%]
Edge 1
Edge 2
Edge 3
Edge 4
<= 100 m
<= 75 m
<= 50 m
<= 25 m
<= 10 m
Distance to Nearest Ground Structure
Flight Angle vs. Edge [%]
1 2 3 4
Angle Distribution

Bee R09

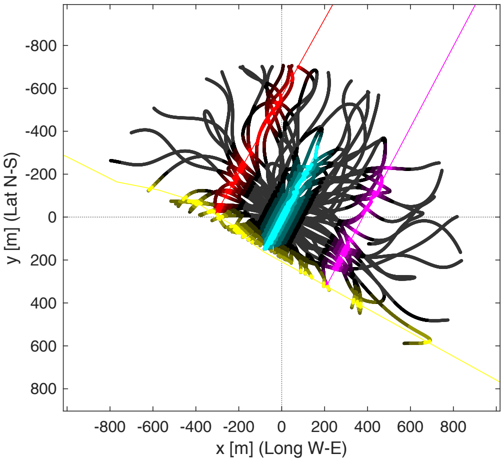
y [m] (Lat N-S)
x [m] (Long W-E)

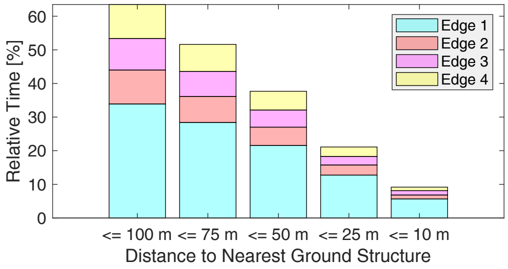
Relative Time [%]
Edge 1
Edge 2
Edge 3
Edge 4
<= 100 m <= 75 m <= 50 m <= 25 m <= 10 m
Distance to Nearest Ground Structure

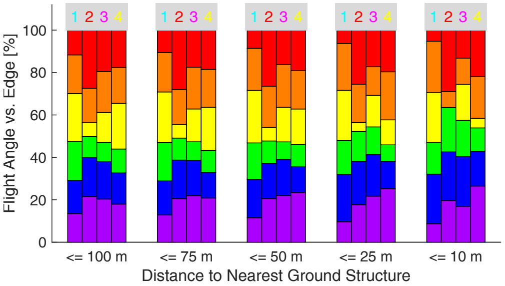
Flight Angle vs. Edge [%]
1 2 3 4
<= 100 m <= 75 m <= 50 m <= 25 m <= 10 m
Distance to Nearest Ground Structure

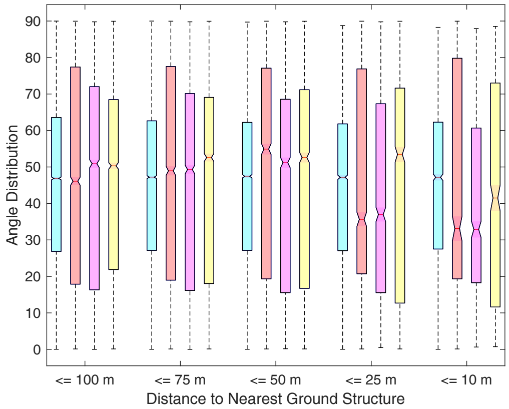
Angle Distribution
<= 100 m <= 75 m <= 50 m <= 25 m <= 10 m
Distance to Nearest Ground Structure

Bee R10

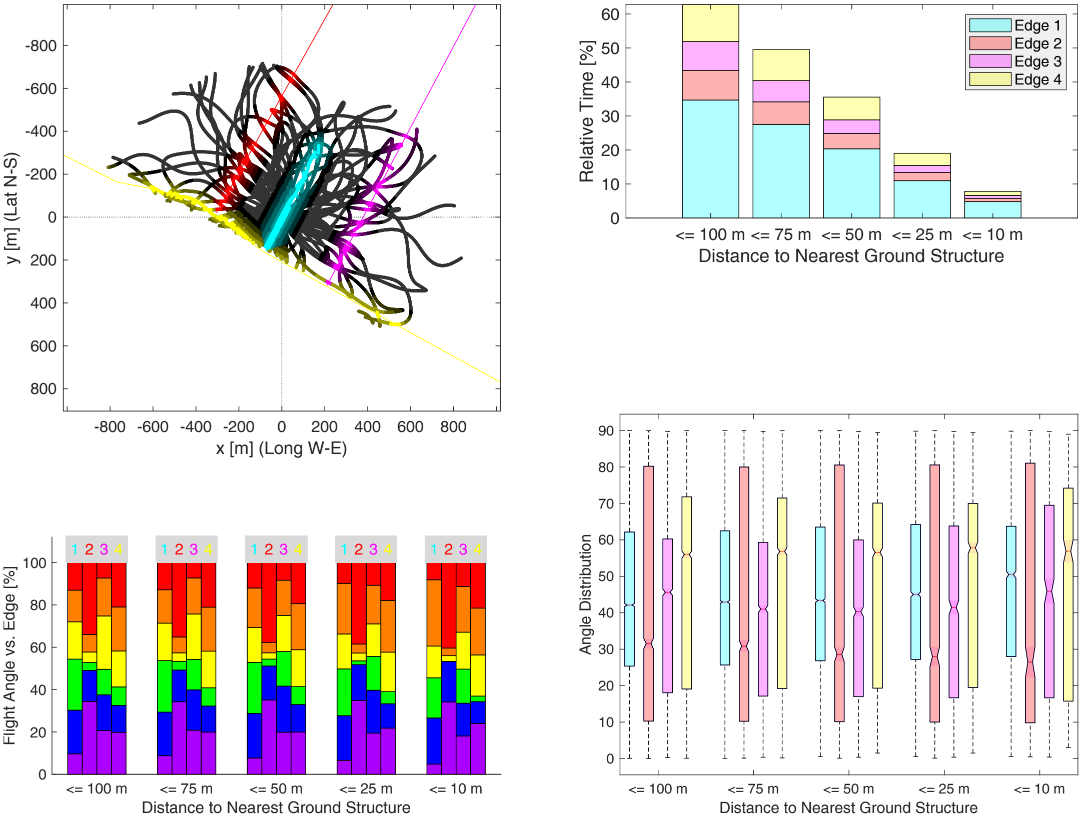
y [m] (Lat N-S)
x [m] (Long W-E)
-800
-600
-400
-200
0
200
400
600
800
Relative Time [%]
Edge 1
Edge 2
Edge 3
Edge 4
<= 100 m
<= 75 m
<= 50 m
<= 25 m
<= 10 m
Distance to Nearest Ground Structure
Flight Angle vs. Edge [%]
1 2 3 4
Angle Distribution

Bee R11

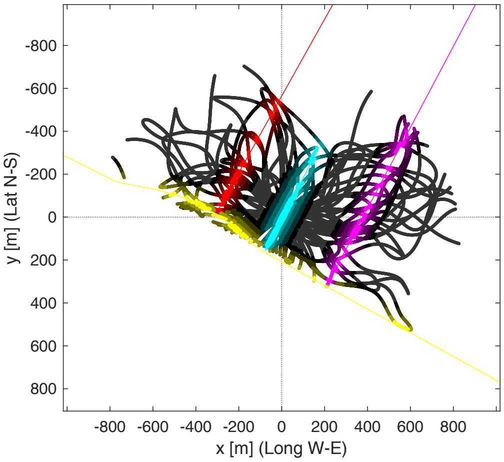
y [m] (Lat N-S)
x [m] (Long W-E)
-800
-600
-400
-200
0
200
400
600
800

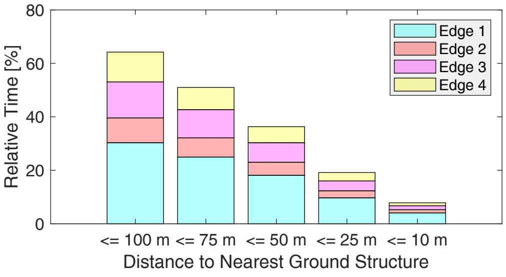
Edge 1
Edge 2
Edge 3
Edge 4
Relative Time [%]
<= 100 m
<= 75 m
<= 50 m
<= 25 m
<= 10 m
Distance to Nearest Ground Structure

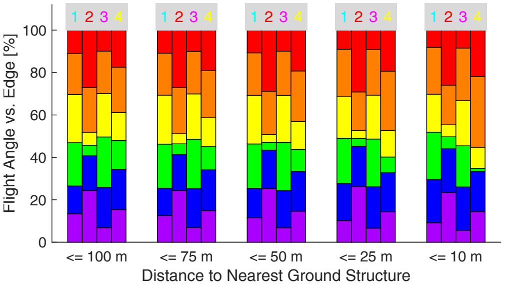
1 2 3 4
Flight Angle vs. Edge [%]
<= 100 m
<= 75 m
<= 50 m
<= 25 m
<= 10 m
Distance to Nearest Ground Structure

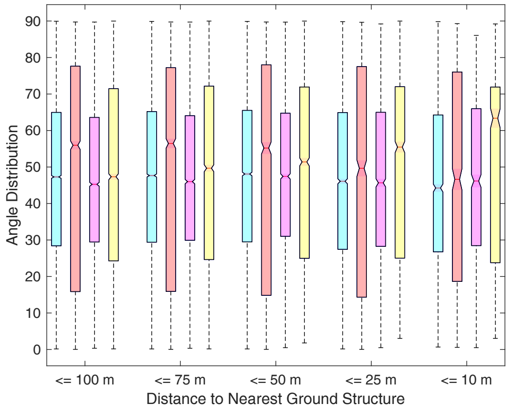
Angle Distribution
<= 100 m
<= 75 m
<= 50 m
<= 25 m
<= 10 m
Distance to Nearest Ground Structure

Bee R12

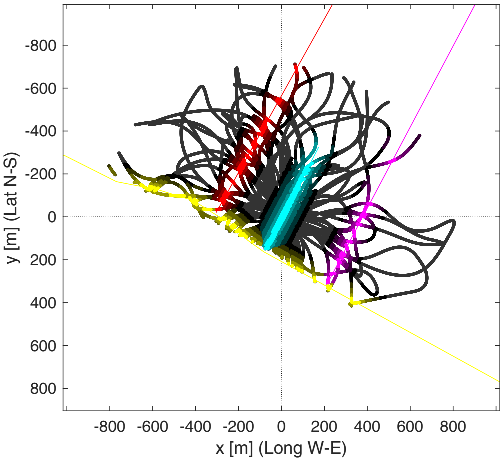
y [m] (Lat N-S)
x [m] (Long W-E)
-800
-600
-400
-200
0
200
400
600
800

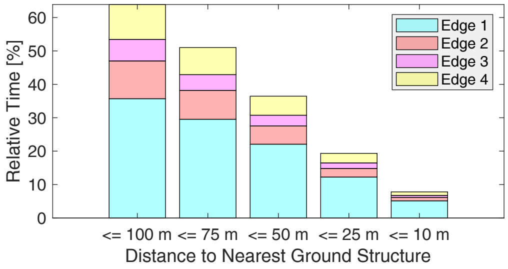
Relative Time [%]
Edge 1
Edge 2
Edge 3
Edge 4
<= 100 m
<= 75 m
<= 50 m
<= 25 m
<= 10 m
Distance to Nearest Ground Structure

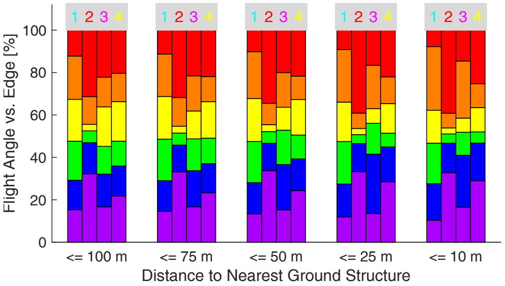
Flight Angle vs. Edge [%]
1 2 3 4
<= 100 m
<= 75 m
<= 50 m
<= 25 m
<= 10 m
Distance to Nearest Ground Structure

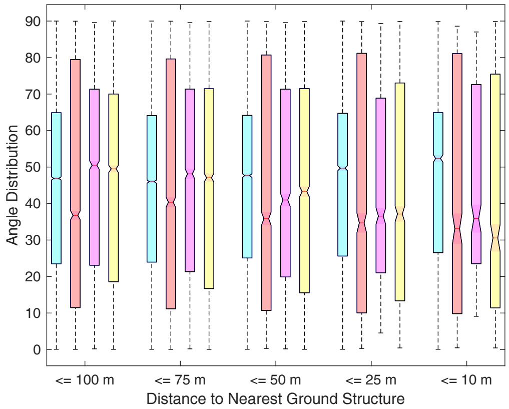
Angle Distribution
<= 100 m
<= 75 m
<= 50 m
<= 25 m
<= 10 m
Distance to Nearest Ground Structure

Bee R13

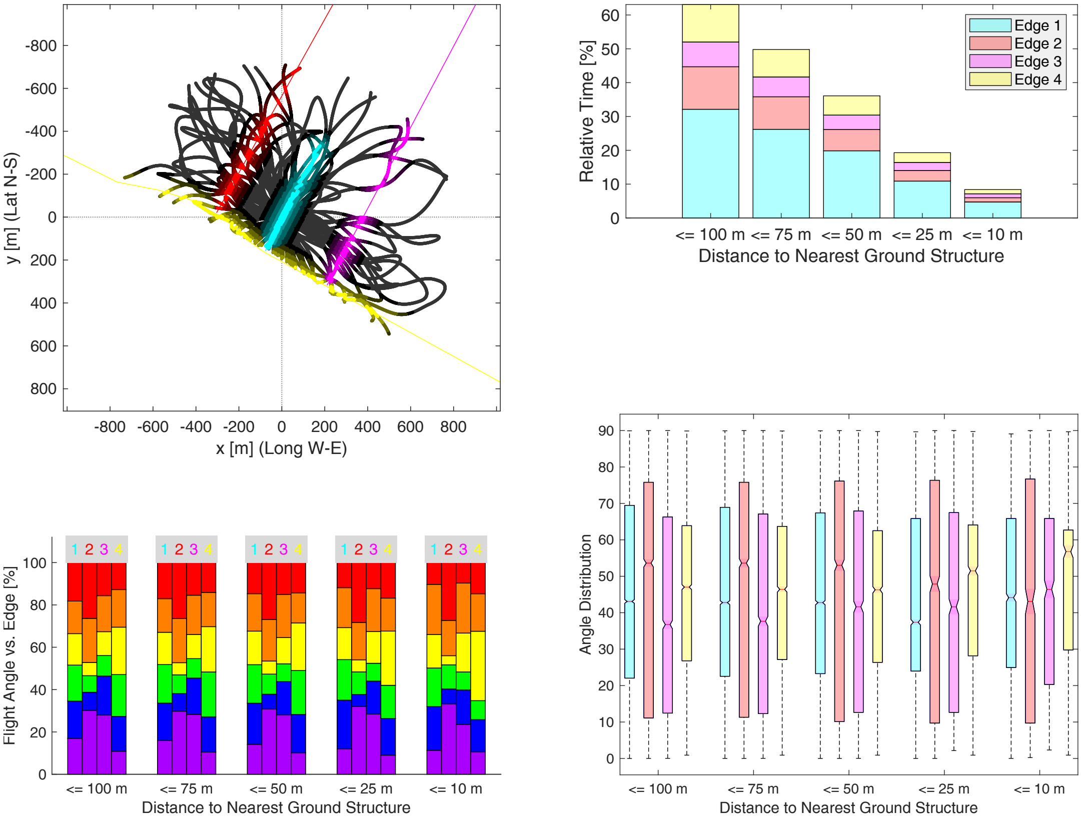
y [m] (Lat N-S)
x [m] (Long W-E)
Relative Time [%]
Edge 1
Edge 2
Edge 3
Edge 4
Distance to Nearest Ground Structure
<= 100 m
<= 75 m
<= 50 m
<= 25 m
<= 10 m
Flight Angle vs. Edge [%]
1 2 3 4
Angle Distribution

Bee R14

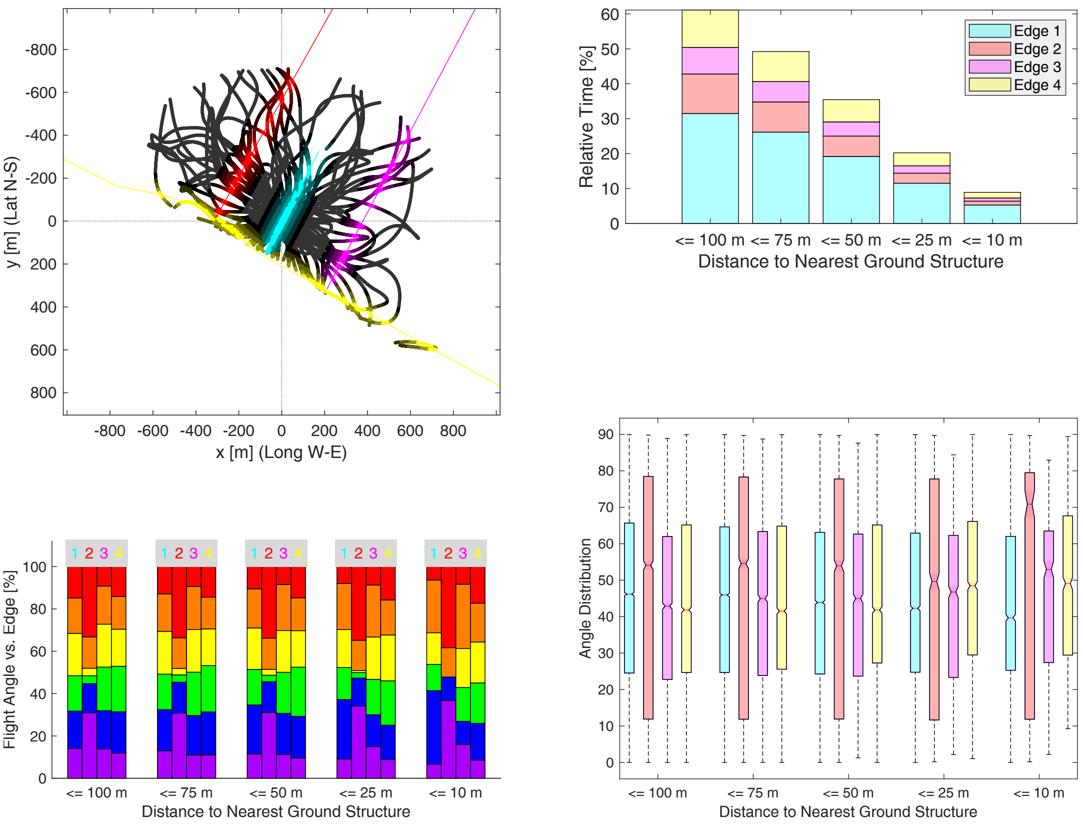

y [m] (Lat N-S)
x [m] (Long W-E)
-800
-600
-400
-200
0
200
400
600
800
Relative Time [%]
Edge 1
Edge 2
Edge 3
Edge 4
<= 100 m
<= 75 m
<= 50 m
<= 25 m
<= 10 m
Distance to Nearest Ground Structure
Flight Angle vs. Edge [%]
1 2 3 4
Angle Distribution

Bee R15

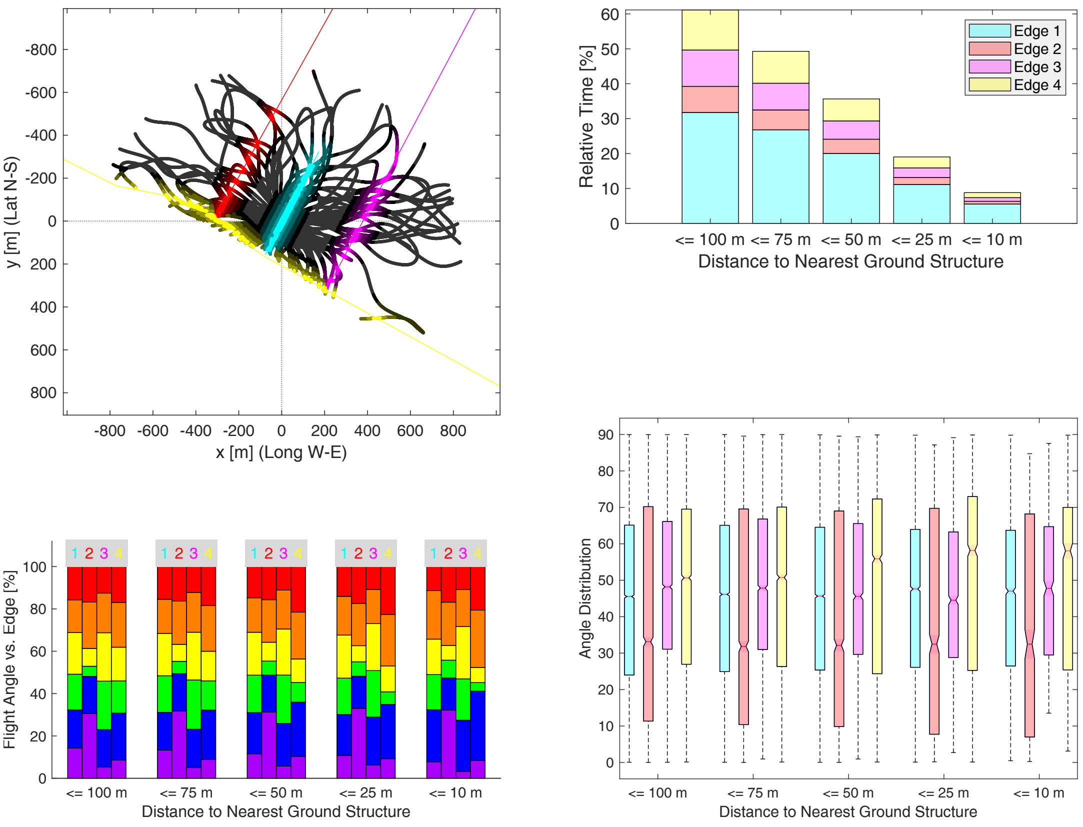
y [m] (Lat N-S)
x [m] (Long W-E)
-800 -600 -400 -200 0 200 400 600 800
Relative Time [%]
Edge 1
Edge 2
Edge 3
Edge 4
<= 100 m <= 75 m <= 50 m <= 25 m <= 10 m
Distance to Nearest Ground Structure
Flight Angle vs. Edge [%]
1 2 3 4
Angle Distribution

Bee R16

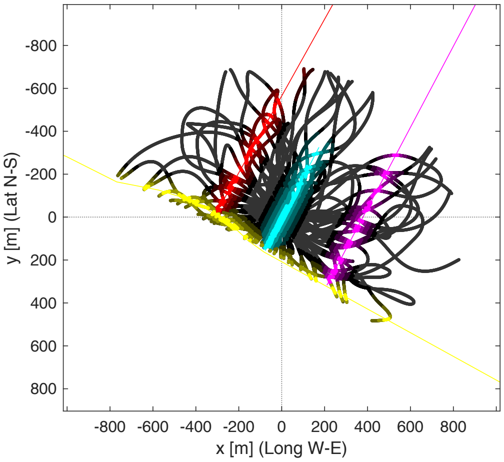
-800
-600
-400
-200
0
200
400
600
800
y [m] (Lat N-S)
-800 -600 -400 -200 0 200 400 600 800
x [m] (Long W-E)

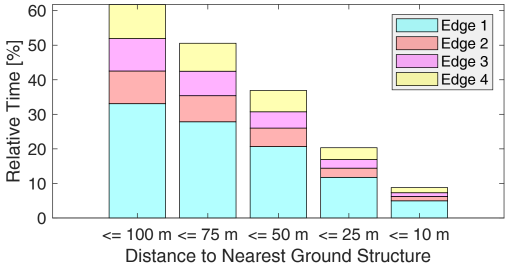
Edge 1
Edge 2
Edge 3
Edge 4
0 10 20 30 40 50 60
Relative Time [%]
<= 100 m <= 75 m <= 50 m <= 25 m <= 10 m
Distance to Nearest Ground Structure

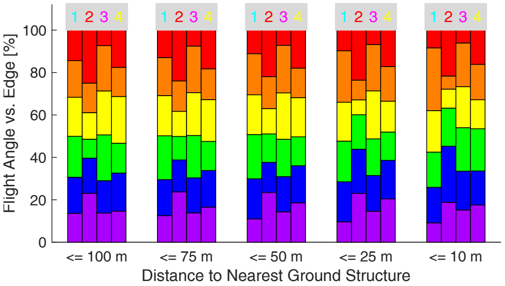
1 2 3 4
0 20 40 60 80 100
Flight Angle vs. Edge [%]
<= 100 m <= 75 m <= 50 m <= 25 m <= 10 m
Distance to Nearest Ground Structure

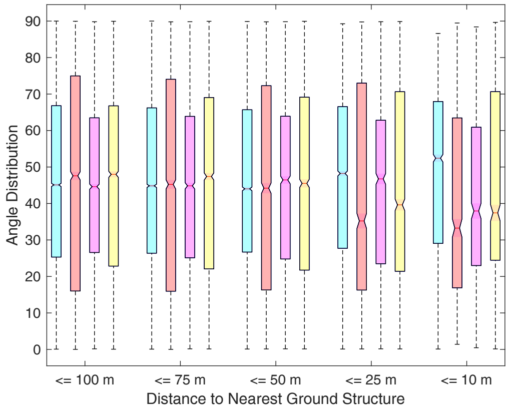
0 10 20 30 40 50 60 70 80 90
Angle Distribution
<= 100 m <= 75 m <= 50 m <= 25 m <= 10 m
Distance to Nearest Ground Structure
