# Supplementary material for: Generalization of navigation memory in honeybees
Source: Front Behav Neurosci. 2023 Mar 6;17:1070957. doi: 10.3389/fnbeh.2023.1070957 (PMC10025308; doi:10.3389/fnbeh.2023.1070957)
Supplement: Supplementary Data Sheet S12 — Significance analyses of time and flight angle near edges for all bee groups. [file Data_Sheet_12.pdf]

Generalization of Navigation Memory in Honeybees

Supplement Data Sheet 12: Significance Analyses of Time and Flight Angle near Edges for all Bee Groups

Eric Bullinger*
Otto-von-Guericke-Universität Magdeburg
Institut für Automatisierungstechnik
Universitätsplatz 2, 39106 Magdeburg, Germany
eric.bullinger@ovgu.de

Uwe Greggers & Randolf Menzel*
Freie Universität Berlin
Neurobiologie
Königin Luisestr. 1 -3, 14195 Berlin, Germany
menzel@neurobiologie.fu-berlin.de

14 February 2023

Contents

Edge 1 at $\leq 10\,\mathrm{m}$ **2**

Edge 1 at $\leq 25\,\mathrm{m}$ **4**

Edge 1 at $\leq 50\,\mathrm{m}$ **6**

Edge 1 at $\leq 75\,\mathrm{m}$ **8**

Edge 1 at $\leq 100\,\mathrm{m}$ **10**

Edge 2 at $\leq 10\,\mathrm{m}$ **12**

Edge 2 at $\leq 25\,\mathrm{m}$ **14**

Edge 2 at $\leq 50\,\mathrm{m}$ **16**

Edge 2 at $\leq 75\,\mathrm{m}$ **18**

Edge 2 at $\leq 100\,\mathrm{m}$ **20**

Edge 3 at $\leq 10\,\mathrm{m}$ **22**

Edge 3 at $\leq 25\,\mathrm{m}$ **24**

Edge 3 at $\leq 50\,\mathrm{m}$ **26**

Edge 3 at $\leq 75\,\mathrm{m}$ **28**

Edge 3 at $\leq 100\,\mathrm{m}$ **30**

Edge 4 at $\leq 10\,\mathrm{m}$ **32**

Edge 4 at $\leq 25\,\mathrm{m}$ **34**

Edge 4 at $\leq 50\,\mathrm{m}$ **36**

Edge 4 at $\leq 75\,\mathrm{m}$ **38**

Edge 4 at $\leq 100\,\mathrm{m}$ **40**

*corresponding author

Edge 1 at ≤10 m

Edge 1 at ≤10 m — Time near Edge

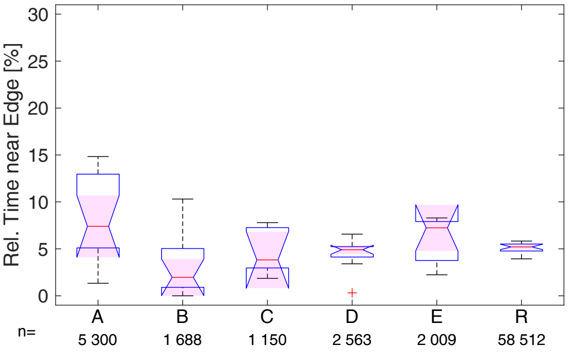

	A	B	C	D	E	R
A	-	.012	.529	.184	.999	.560
B	0.07	-	.964	.896	.170	.433
C	0.21	0.64	-	.999	.841	.994
D	0.21	0.73	0.60	-	.661	.972
E	0.50	0.82	0.80	1.00	-	.942
R	0.29	0.82	0.60	0.69	0.29	-

Edge 1 at ≤10 m — Angle in Range 0° to 15°

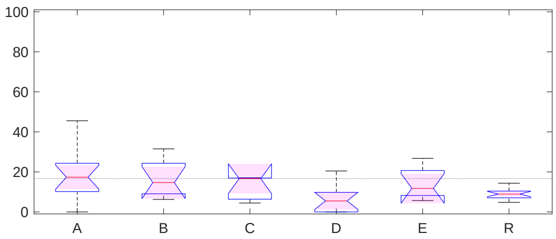

	A	B	C	D	E	R
A	-	.999	.968	.008	.989	.170
B	0.36	-	.979	.031	.993	.312
C	0.50	0.56	-	.524	.999	.952
D	0.14	0.00	0.20	-	.245	.833
E	0.29	0.33	0.40	0.85	-	.805
R	0.21	0.11	0.40	0.69	0.29	-

Edge 1 at ≤10 m — Angle in Range 15° to 30°

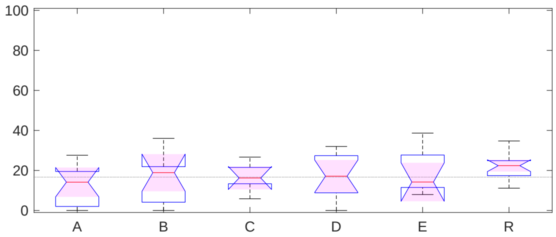

	A	B	C	D	E	R
A	-	.957	.969	.795	.737	.051
B	0.71	-	.999	.999	.994	.583
C	0.57	0.44	-	.999	.999	.823
D	0.71	0.44	0.60	-	.999	.686
E	0.50	0.33	0.20	0.46	-	.951
R	0.93	0.78	0.80	0.69	0.57	-

Edge 1 at ≤10 m — Angle in Range 30° to 45°

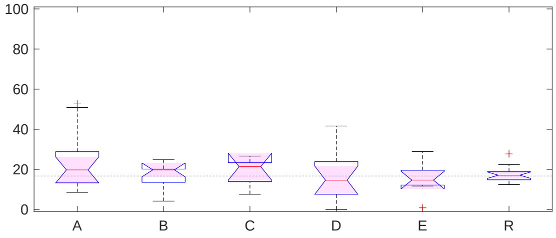

	A	B	C	D	E	R
A	-	.875	.999	.517	.637	.709
B	0.50	-	.996	.998	.997	.999
C	0.57	0.89	-	.949	.950	.988
D	0.29	0.33	0.20	-	.999	.999
E	0.29	0.33	0.20	0.54	-	.998
R	0.29	0.44	0.40	0.54	0.71	-

Edge 1 at ≤10 m — Angle in Range 45° to 60°

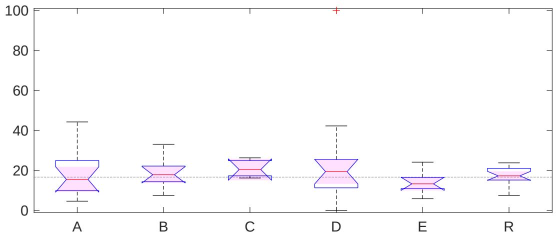

	A	B	C	D	E	R
A	-	.997	.752	.996	.955	.999
B	0.57	-	.946	.999	.839	.999
C	0.64	0.78	-	.927	.409	.866
D	0.64	0.78	0.40	-	.802	.999
E	0.36	0.22	0.00	0.38	-	.858
R	0.57	0.33	0.20	0.46	0.86	-

Edge 1 at ≤10 m — Angle in Range 60° to 75°

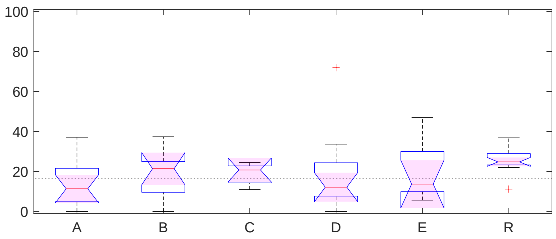

	A	B	C	D	E	R
A	-	.940	.979	.999	.955	.020
B	0.71	-	.999	.994	.999	.452
C	0.71	0.44	-	.998	.999	.656
D	0.50	0.33	0.20	-	.995	.081
E	0.57	0.33	0.20	0.54	-	.555
R	0.79	0.78	1.00	0.77	0.71	-

Edge 1 at ≤10 m — Angle in Range 75° to 90°

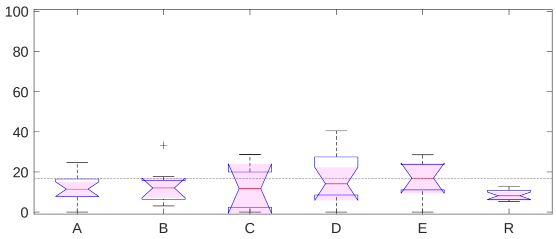

	A	B	C	D	E	R
A	-	.999	.999	.869	.941	.715
B	0.57	-	.999	.944	.973	.760
C	0.57	0.44	-	.945	.968	.938
D	0.57	0.67	0.60	-	.999	.108
E	0.79	0.78	0.60	0.54	-	.294
R	0.29	0.33	0.40	0.23	0.14	-

;

Edge 1 at ≤25 m

Edge 1 at ≤25 m — Time near Edge

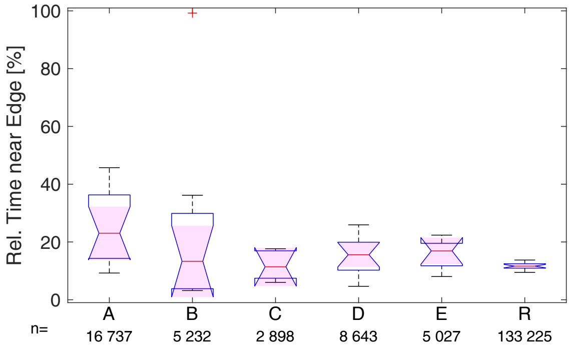

	A	B	C	D	E	R
A	-	.428	.175	.502	.818	.007
B	0.14	-	.951	.999	.999	.771
C	0.14	0.36	-	.902	.877	.999
D	0.29	0.55	0.60	-	.999	.601
E	0.29	0.55	0.80	0.54	-	.642
R	0.14	0.36	0.60	0.31	0.29	-

Edge 1 at ≤25 m — Angle in Range 0° to 15°

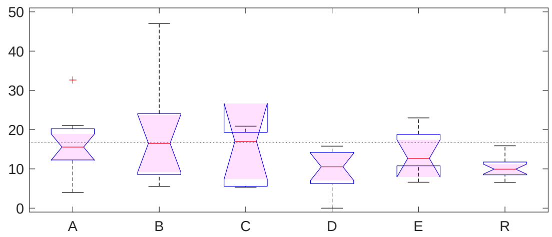

	A	B	C	D	E	R
A	-	.999	.988	.219	.998	.158
B	0.50	-	.982	.229	.996	.172
C	0.57	0.55	-	.915	.999	.896
D	0.21	0.27	0.40	-	.710	.999
E	0.36	0.27	0.40	0.69	-	.660
R	0.21	0.27	0.40	0.38	0.14	-

Edge 1 at ≤25 m — Angle in Range 15° to 30°

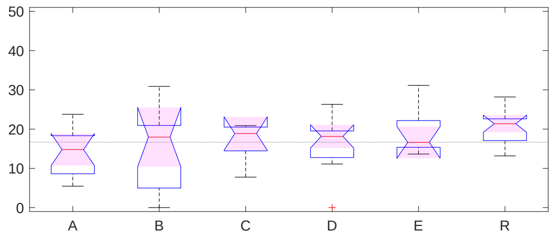

	A	B	C	D	E	R
A	-	.927	.888	.829	.733	.040
B	0.71	-	.999	.999	.995	.506
C	0.86	0.55	-	.999	.999	.921
D	0.71	0.55	0.40	-	.999	.587
E	0.50	0.45	0.20	0.38	-	.935
R	0.93	0.82	1.00	0.77	0.71	-

Edge 1 at ≤25 m — Angle in Range 30° to 45°

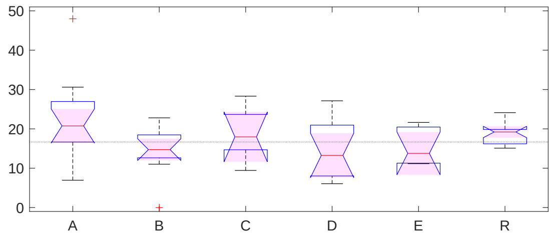

	A	B	C	D	E	R
A	-	.123	.991	.257	.419	.976
B	0.14	-	.788	.998	.999	.415
C	0.43	0.73	-	.921	.933	.999
D	0.07	0.27	0.20	-	.999	.665
E	0.07	0.45	0.20	0.54	-	.779
R	0.50	0.82	0.60	0.62	0.71	-

Edge 1 at $\leq$25 m — Angle in Range 45° to 60°

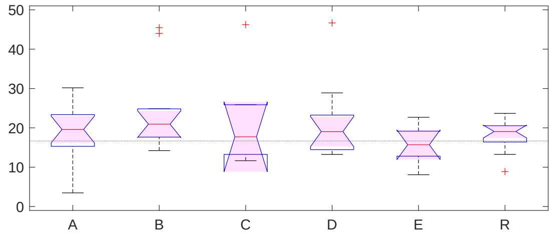

	A	B	C	D	E	R
A	-	.925	.983	.999	.713	.999
B	0.64	-	.731	.839	.248	.763
C	0.43	0.27	-	.996	.996	.998
D	0.50	0.36	0.60	-	.837	.999
E	0.29	0.09	0.40	0.38	-	.854
R	0.50	0.36	0.60	0.54	0.71	-

Edge 1 at $\leq$25 m — Angle in Range 60° to 75°

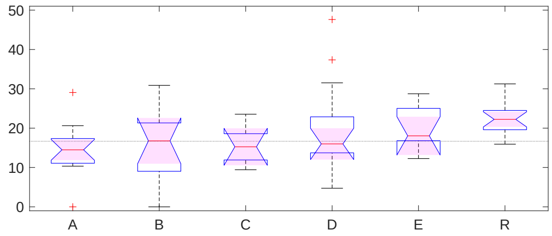

	A	B	C	D	E	R
A	-	.970	.999	.814	.580	.004
B	0.71	-	.998	.999	.946	.102
C	0.50	0.36	-	.977	.870	.152
D	0.57	0.36	0.60	-	.992	.206
E	0.79	0.64	0.80	0.54	-	.803
R	0.93	0.91	0.80	0.77	0.71	-

Edge 1 at $\leq$25 m — Angle in Range 75° to 90°

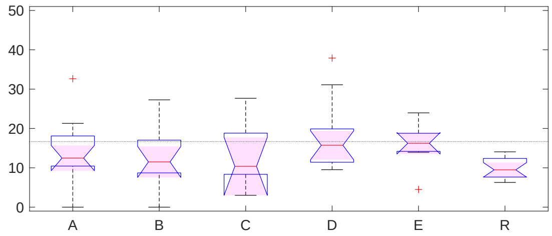

	A	B	C	D	E	R
A	-	.969	.996	.931	.968	.208
B	0.43	-	.999	.547	.713	.782
C	0.21	0.45	-	.838	.890	.878
D	0.57	0.73	0.60	-	.999	.017
E	0.57	0.73	0.80	0.54	-	.086
R	0.21	0.27	0.20	0.00	0.14	-

;

Edge 1 at $\leq 50\,\mathrm{m}$

Edge 1 at $\leq 50\,\mathrm{m}$ — Time near Edge

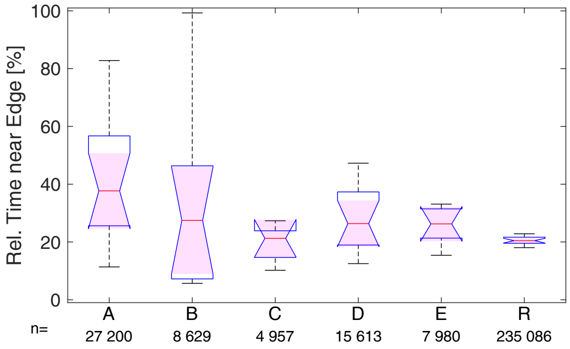

	A	B	C	D	E	R
A	-	.386	.166	.498	.698	.005
B	0.36	-	.958	.999	.999	.773
C	0.07	0.45	-	.895	.929	.999
D	0.36	0.45	0.80	-	.999	.559
E	0.36	0.45	0.80	0.46	-	.741
R	0.07	0.45	0.40	0.31	0.29	-

Edge 1 at $\leq 50\,\mathrm{m}$ — Angle in Range 0° to 15°

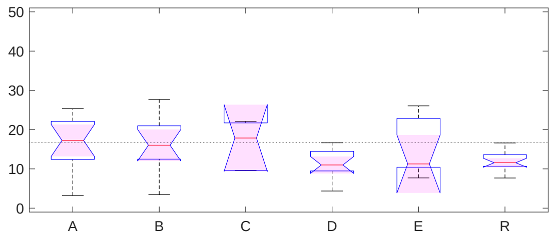

	A	B	C	D	E	R
A	-	.999	.999	.219	.974	.233
B	0.43	-	.999	.328	.986	.355
C	0.50	0.55	-	.807	.999	.843
D	0.14	0.18	0.40	-	.887	.999
E	0.14	0.18	0.40	0.54	-	.917
R	0.21	0.18	0.40	0.54	0.57	-

Edge 1 at $\leq 50\,\mathrm{m}$ — Angle in Range 15° to 30°

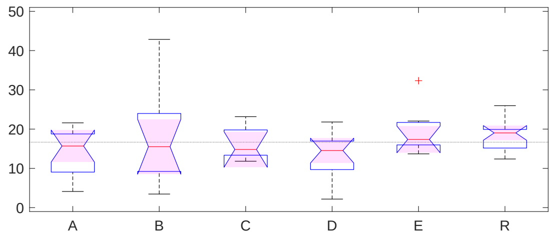

	A	B	C	D	E	R
A	-	.972	.995	.999	.497	.287
B	0.43	-	.999	.958	.908	.854
C	0.43	0.45	-	.991	.945	.933
D	0.43	0.45	0.40	-	.461	.259
E	0.64	0.64	0.60	0.85	-	.999
R	0.86	0.64	0.80	0.85	0.57	-

Edge 1 at $\leq 50\,\mathrm{m}$ — Angle in Range 30° to 45°

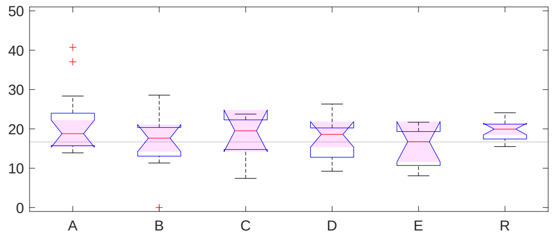

	A	B	C	D	E	R
A	-	.890	.999	.890	.476	.999
B	0.43	-	.998	.999	.966	.881
C	0.57	0.64	-	.998	.886	.999
D	0.43	0.55	0.40	-	.952	.880
E	0.29	0.36	0.20	0.38	-	.455
R	0.57	0.64	0.60	0.77	0.86	-

Edge 1 at ≤50 m — Angle in Range 45° to 60°

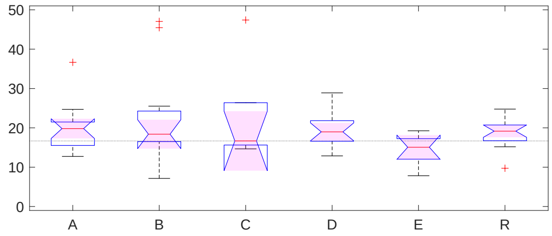

	A	B	C	D	E	R
A	-	.999	.997	.999	.244	.999
B	0.43	-	.999	.999	.365	.999
C	0.36	0.27	-	.998	.782	.995
D	0.43	0.64	0.60	-	.287	.999
E	0.14	0.18	0.20	0.15	-	.204
R	0.43	0.64	0.60	0.62	0.86	-

Edge 1 at ≤50 m — Angle in Range 60° to 75°

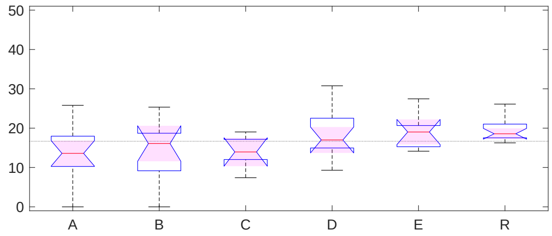

	A	B	C	D	E	R
A	-	.992	.999	.284	.256	.030
B	0.64	-	.998	.722	.608	.225
C	0.57	0.45	-	.630	.519	.251
D	0.71	0.55	0.80	-	.999	.969
E	0.86	0.82	0.80	0.69	-	.999
R	0.86	0.64	0.80	0.54	0.43	-

Edge 1 at ≤50 m — Angle in Range 75° to 90°

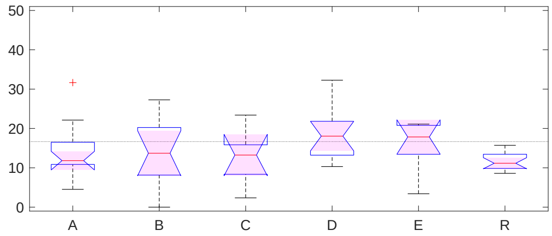

	A	B	C	D	E	R
A	-	.999	.999	.495	.938	.847
B	0.64	-	.999	.386	.871	.965
C	0.57	0.45	-	.564	.894	.997
D	0.79	0.73	0.80	-	.995	.036
E	0.79	0.73	0.80	0.46	-	.400
R	0.36	0.45	0.40	0.15	0.14	-

;

Edge 1 at ≤75 m

Edge 1 at ≤75 m — Time near Edge

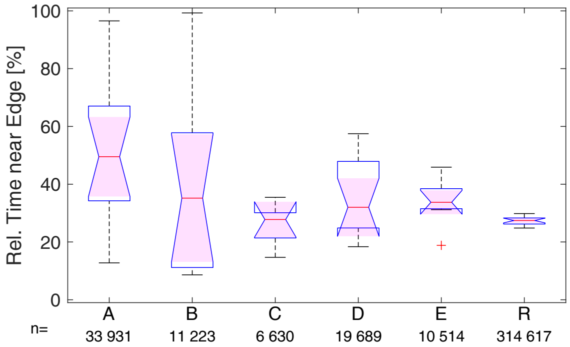

	A	B	C	D	E	R
A	-	.370	.167	.421	.878	.010
B	0.36	-	.962	.999	.993	.873
C	0.14	0.45	-	.926	.822	.999
D	0.21	0.45	0.80	-	.998	.754
E	0.21	0.45	0.80	0.54	-	.617
R	0.14	0.45	0.40	0.31	0.14	-

Edge 1 at ≤75 m — Angle in Range 0° to 15°

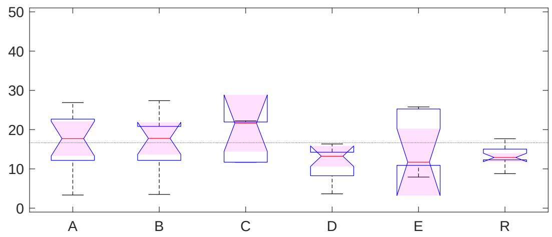

	A	B	C	D	E	R
A	-	.999	.999	.193	.981	.518
B	0.50	-	.999	.381	.997	.746
C	0.71	0.82	-	.510	.991	.797
D	0.29	0.27	0.40	-	.840	.983
E	0.14	0.27	0.40	0.46	-	.985
R	0.29	0.27	0.40	0.46	0.57	-

Edge 1 at ≤75 m — Angle in Range 15° to 30°

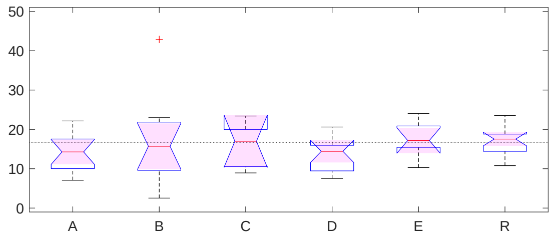

	A	B	C	D	E	R
A	-	.906	.981	.999	.625	.600
B	0.57	-	.999	.838	.989	.998
C	0.57	0.55	-	.960	.991	.998
D	0.57	0.36	0.40	-	.534	.491
E	0.57	0.55	0.60	0.77	-	.999
R	0.71	0.55	0.60	0.85	0.57	-

Edge 1 at ≤75 m — Angle in Range 30° to 45°

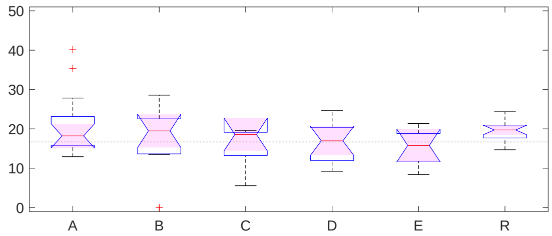

	A	B	C	D	E	R
A	-	.983	.893	.871	.593	.999
B	0.57	-	.995	.999	.930	.895
C	0.50	0.45	-	.999	.999	.751
D	0.43	0.45	0.40	-	.986	.643
E	0.21	0.45	0.20	0.38	-	.373
R	0.57	0.64	1.00	0.69	0.86	-

Edge 1 at ≤75 m — Angle in Range 45° to 60°

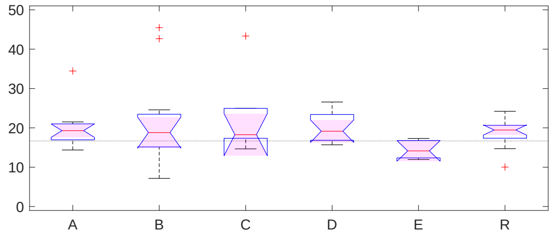

	A	B	C	D	E	R
A	-	.999	.999	.999	.087	.999
B	0.43	-	.999	.999	.129	.999
C	0.43	0.45	-	.999	.309	.999
D	0.43	0.64	0.80	-	.051	.999
E	0.00	0.18	0.00	0.00	-	.049
R	0.57	0.64	0.80	0.54	1.00	-

Edge 1 at ≤75 m — Angle in Range 60° to 75°

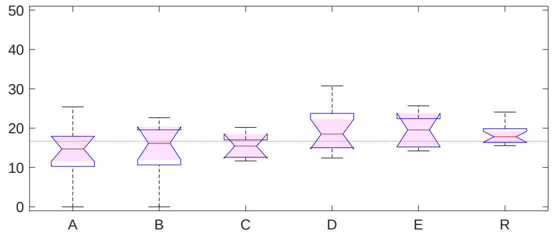

	A	B	C	D	E	R
A	-	.998	.999	.321	.518	.286
B	0.57	-	.998	.669	.791	.650
C	0.57	0.36	-	.601	.694	.592
D	0.79	0.73	0.80	-	.999	.999
E	0.79	0.73	0.80	0.62	-	.999
R	0.71	0.64	0.80	0.38	0.43	-

Edge 1 at ≤75 m — Angle in Range 75° to 90°

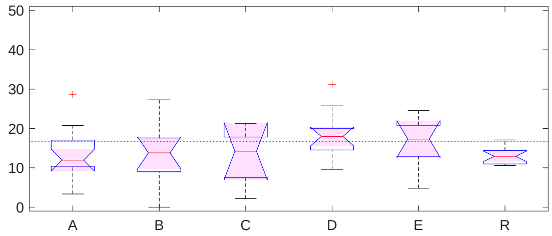

	A	B	C	D	E	R
A	-	.999	.999	.296	.728	.999
B	0.57	-	.999	.459	.831	.999
C	0.57	0.55	-	.642	.876	.999
D	0.79	0.73	0.80	-	.999	.179
E	0.79	0.73	0.80	0.38	-	.609
R	0.57	0.36	0.40	0.15	0.29	-

;

Edge 1 at ≤100 m

Edge 1 at ≤100 m — Time near Edge

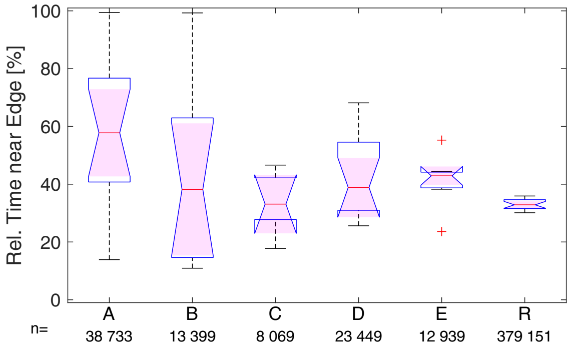

	A	B	C	D	E	R
A	-	.280	.338	.420	.911	.012
B	0.21	-	.999	.999	.970	.942
C	0.14	0.45	-	.990	.925	.999
D	0.21	0.55	0.60	-	.995	.777
E	0.29	0.64	0.80	0.62	-	.578
R	0.14	0.45	0.40	0.38	0.14	-

Edge 1 at ≤100 m — Angle in Range 0° to 15°

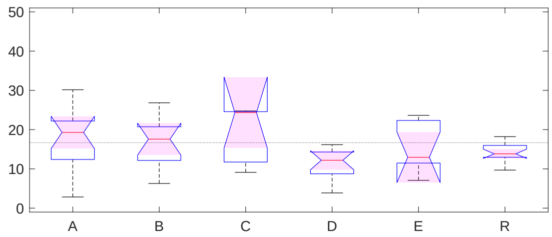

	A	B	C	D	E	R
A	-	.999	.999	.084	.952	.779
B	0.50	-	.998	.239	.992	.944
C	0.86	0.82	-	.248	.946	.849
D	0.21	0.27	0.20	-	.775	.692
E	0.29	0.27	0.40	0.54	-	.999
R	0.29	0.27	0.40	0.69	0.57	-

Edge 1 at ≤100 m — Angle in Range 15° to 30°

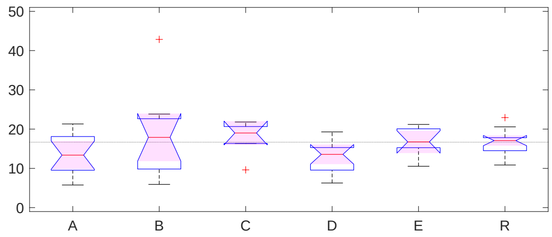

	A	B	C	D	E	R
A	-	.697	.442	.996	.747	.765
B	0.71	-	.983	.396	.999	.999
C	0.93	0.55	-	.239	.994	.943
D	0.57	0.36	0.20	-	.483	.439
E	0.57	0.45	0.20	0.85	-	.999
R	0.64	0.45	0.20	0.85	0.57	-

Edge 1 at ≤100 m — Angle in Range 30° to 45°

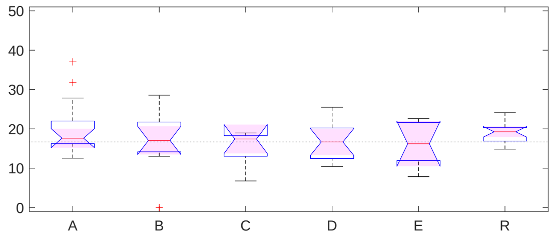

	A	B	C	D	E	R
A	-	.980	.868	.933	.961	.998
B	0.50	-	.994	.999	.999	.865
C	0.50	0.55	-	.998	.999	.690
D	0.43	0.45	0.40	-	.999	.725
E	0.21	0.45	0.40	0.38	-	.836
R	0.64	0.64	1.00	0.69	0.57	-

Edge 1 at ≤100 m — Angle in Range 45° to 60°

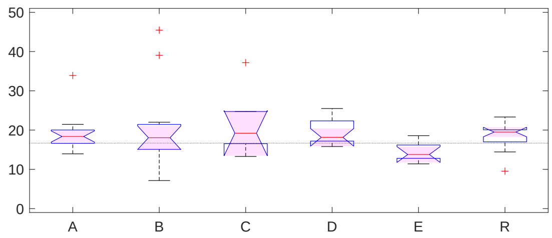

	A	B	C	D	E	R
A	-	.999	.998	.983	.158	.999
B	0.43	-	.997	.980	.222	.999
C	0.64	0.64	-	.999	.208	.999
D	0.50	0.55	0.40	-	.040	.999
E	0.00	0.18	0.20	0.00	-	.076
R	0.71	0.64	0.60	0.54	1.00	-

Edge 1 at ≤100 m — Angle in Range 60° to 75°

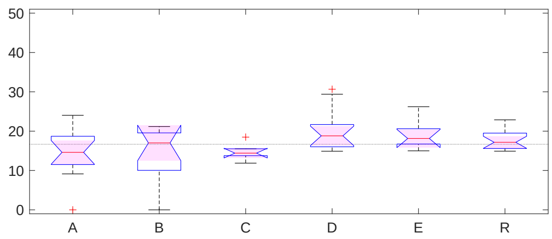

	A	B	C	D	E	R
A	-	.994	.985	.077	.409	.456
B	0.64	-	.896	.354	.761	.873
C	0.50	0.36	-	.090	.293	.355
D	0.79	0.64	1.00	-	.999	.920
E	0.71	0.55	0.80	0.31	-	.997
R	0.64	0.55	0.80	0.31	0.29	-

Edge 1 at ≤100 m — Angle in Range 75° to 90°

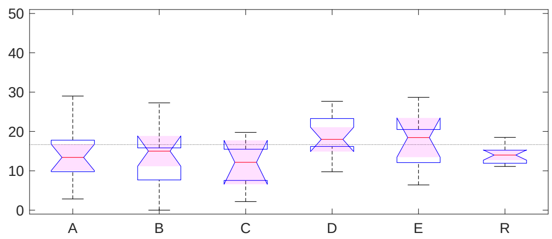

	A	B	C	D	E	R
A	-	.999	.983	.291	.878	.999
B	0.50	-	.992	.299	.858	.999
C	0.50	0.36	-	.229	.668	.984
D	0.79	0.82	0.80	-	.989	.239
E	0.79	0.82	0.80	0.54	-	.854
R	0.50	0.45	0.60	0.15	0.29	-

;

Edge 2 at ≤10 m

Edge 2 at ≤10 m — Time near Edge

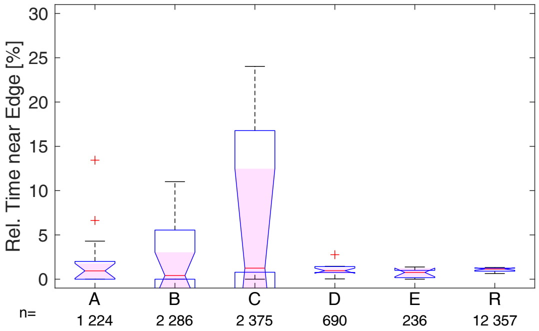

	A	B	C	D	E	R
A	-	.999	.935	.999	.889	.999
B	0.36	-	.866	.991	.971	.994
C	0.50	0.55	-	.982	.536	.971
D	0.50	0.55	0.20	-	.766	.999
E	0.50	0.55	0.20	0.23	-	.781
R	0.50	0.55	0.40	0.54	0.86	-

Edge 2 at ≤10 m — Angle in Range 0° to 15°

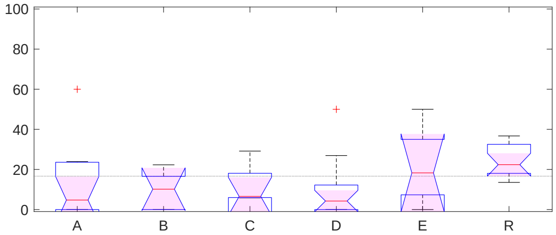

	A	B	C	D	E	R
A	-	.999	.999	.994	.883	.092
B	0.60	-	.995	.999	.853	.146
C	0.60	0.50	-	.964	.995	.688
D	0.50	0.33	0.00	-	.613	.007
E	0.70	0.83	0.75	0.85	-	.948
R	0.70	1.00	0.75	0.85	0.60	-

Edge 2 at ≤10 m — Angle in Range 15° to 30°

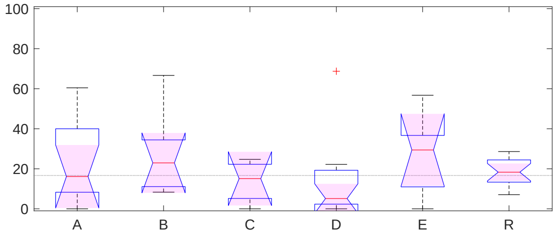

	A	B	C	D	E	R
A	-	.987	.982	.525	.994	.999
B	0.60	-	.855	.265	.999	.993
C	0.50	0.33	-	.995	.896	.956
D	0.20	0.00	0.25	-	.377	.281
E	0.60	0.67	1.00	0.92	-	.998
R	0.50	0.50	0.50	0.69	0.40	-

Edge 2 at ≤10 m — Angle in Range 30° to 45°

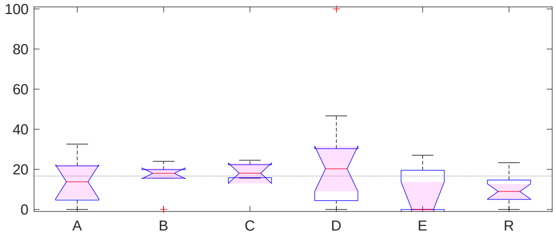

	A	B	C	D	E	R
A	-	.999	.967	.995	.935	.939
B	0.60	-	.997	.999	.873	.873
C	0.60	0.50	-	.998	.677	.651
D	0.60	0.83	0.75	-	.719	.593
E	0.10	0.08	0.00	0.12	-	.999
R	0.40	0.17	0.00	0.38	0.60	-

Edge 2 at $\leq 10\,\mathrm{m}$ — Angle in Range 45° to 60°

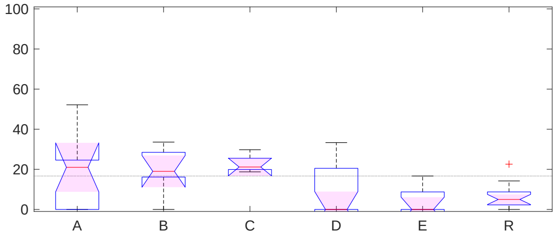

	A	B	C	D	E	R
A	-	.999	.907	.746	.389	.512
B	0.50	-	.989	.608	.298	.409
C	0.50	0.67	-	.301	.130	.178
D	0.15	0.08	0.00	-	.945	.999
E	0.15	0.08	0.00	0.50	-	.983
R	0.30	0.17	0.00	0.54	0.60	-

Edge 2 at $\leq 10\,\mathrm{m}$ — Angle in Range 60° to 75°

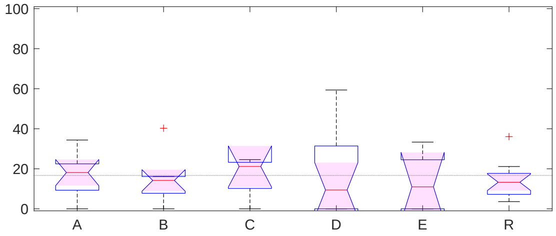

	A	B	C	D	E	R
A	-	.994	.999	.967	.976	.980
B	0.30	-	.993	.999	.999	.999
C	0.60	0.83	-	.976	.977	.984
D	0.30	0.33	0.25	-	.999	.999
E	0.30	0.33	0.25	0.54	-	.999
R	0.30	0.33	0.25	0.54	0.60	-

Edge 2 at $\leq 10\,\mathrm{m}$ — Angle in Range 75° to 90°

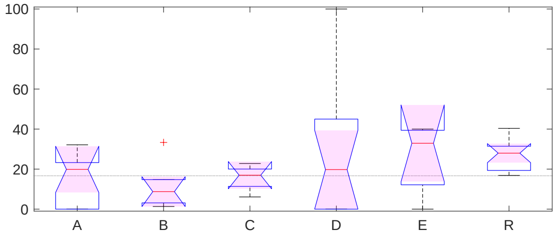

	A	B	C	D	E	R
A	-	.998	.999	.940	.787	.249
B	0.40	-	.999	.816	.637	.194
C	0.40	0.83	-	.976	.875	.574
D	0.50	0.83	0.75	-	.990	.782
E	1.00	0.83	1.00	0.69	-	.999
R	0.90	0.83	1.00	0.69	0.40	-

;

Edge 2 at $\leq 25\,\mathrm{m}$

Edge 2 at $\leq 25\,\mathrm{m}$ — Time near Edge

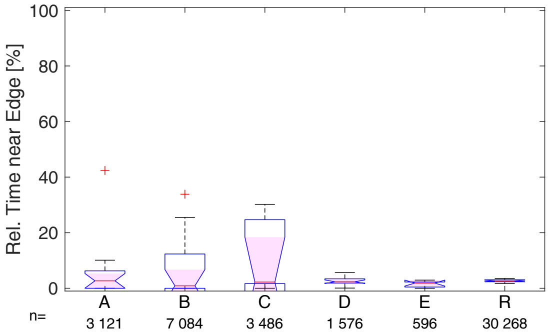

	A	B	C	D	E	R
A	-	.999	.999	.999	.773	.999
B	0.43	-	.993	.999	.903	.987
C	0.43	0.55	-	.999	.731	.999
D	0.50	0.55	0.60	-	.753	.999
E	0.43	0.55	0.20	0.23	-	.544
R	0.50	0.55	0.60	0.54	0.86	-

Edge 2 at $\leq 25\,\mathrm{m}$ — Angle in Range 0° to 15°

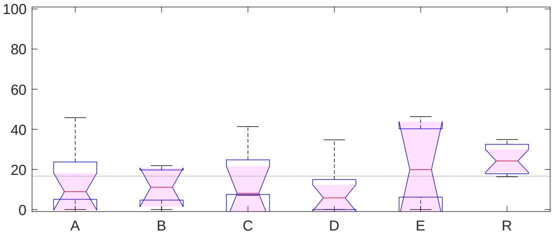

	A	B	C	D	E	R
A	-	.999	.999	.930	.896	.239
B	0.60	-	.997	.996	.830	.252
C	0.50	0.50	-	.928	.988	.776
D	0.30	0.33	0.00	-	.418	.007
E	0.70	0.83	0.75	0.85	-	.992
R	0.80	1.00	0.75	0.85	0.60	-

Edge 2 at $\leq 25\,\mathrm{m}$ — Angle in Range 15° to 30°

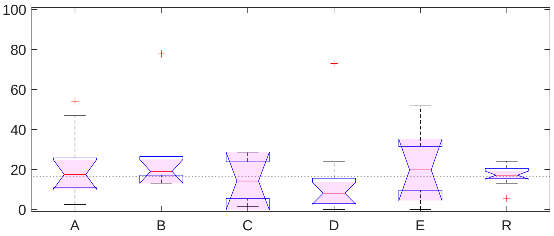

	A	B	C	D	E	R
A	-	.984	.993	.381	.999	.999
B	0.50	-	.891	.169	.991	.959
C	0.40	0.17	-	.963	.996	.995
D	0.10	0.00	0.25	-	.643	.317
E	0.50	0.67	0.75	0.85	-	.999
R	0.50	0.17	0.50	0.77	0.40	-

Edge 2 at $\leq 25\,\mathrm{m}$ — Angle in Range 30° to 45°

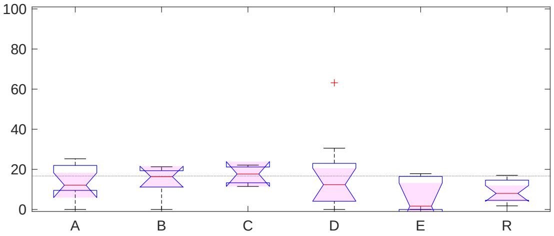

	A	B	C	D	E	R
A	-	.999	.939	.999	.794	.800
B	0.60	-	.987	.999	.744	.764
C	0.60	0.50	-	.900	.422	.402
D	0.50	0.33	0.25	-	.819	.820
E	0.20	0.17	0.00	0.23	-	.999
R	0.20	0.17	0.00	0.38	0.60	-

Edge 2 at ≤25 m — Angle in Range 45° to 60°

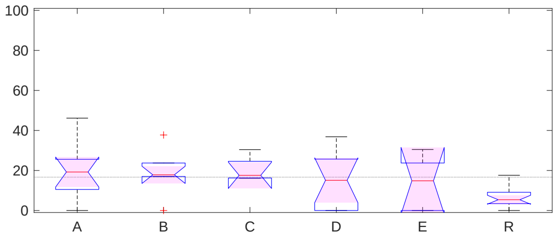

	A	B	C	D	E	R
A	-	.999	.999	.959	.954	.215
B	0.40	-	.999	.967	.958	.354
C	0.40	0.33	-	.930	.919	.359
D	0.40	0.17	0.00	-	.999	.677
E	0.40	0.17	0.00	0.46	-	.959
R	0.20	0.17	0.00	0.38	0.40	-

Edge 2 at ≤25 m — Angle in Range 60° to 75°

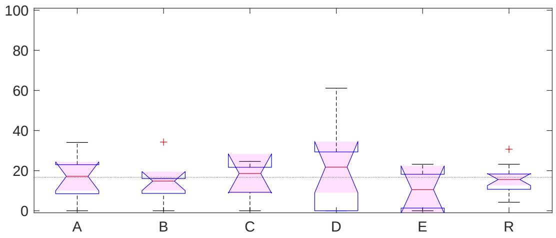

	A	B	C	D	E	R
A	-	.990	.999	.999	.934	.999
B	0.40	-	.991	.957	.999	.999
C	0.50	0.83	-	.999	.952	.999
D	0.60	0.83	0.75	-	.844	.983
E	0.30	0.33	0.25	0.38	-	.981
R	0.50	0.50	0.25	0.38	0.60	-

Edge 2 at ≤25 m — Angle in Range 75° to 90°

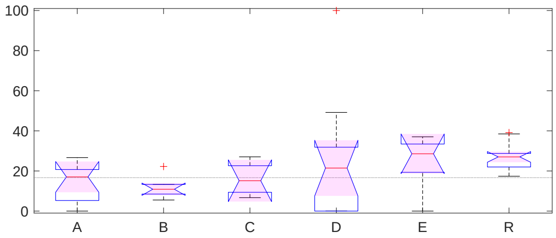

	A	B	C	D	E	R
A	-	.999	.999	.556	.330	.051
B	0.30	-	.996	.505	.292	.070
C	0.40	0.83	-	.934	.719	.489
D	0.80	0.83	0.75	-	.972	.840
E	1.00	1.00	1.00	0.54	-	.999
R	1.00	1.00	0.75	0.54	0.40	-

;

Edge 2 at ≤50 m

Edge 2 at ≤50 m — Time near Edge

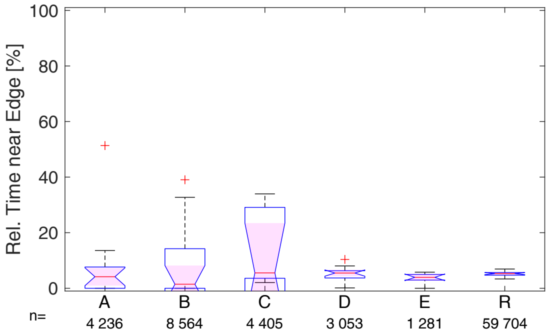

	A	B	C	D	E	R
A	-	.999	.908	.989	.965	.992
B	0.36	-	.891	.982	.983	.986
C	0.57	0.55	-	.994	.625	.989
D	0.57	0.55	0.40	-	.772	.999
E	0.50	0.55	0.20	0.31	-	.784
R	0.57	0.55	0.40	0.38	0.71	-

Edge 2 at ≤50 m — Angle in Range 0° to 15°

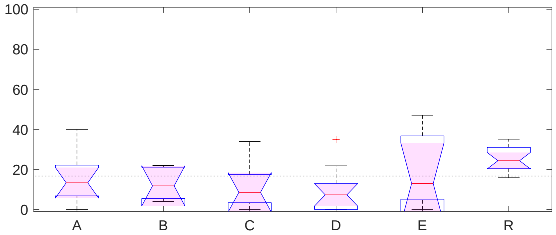

	A	B	C	D	E	R
A	-	.998	.983	.723	.999	.331
B	0.40	-	.999	.979	.996	.243
C	0.30	0.33	-	.999	.978	.181
D	0.30	0.33	0.40	-	.770	.002
E	0.50	0.50	0.80	0.77	-	.620
R	0.80	1.00	0.80	0.92	0.67	-

Edge 2 at ≤50 m — Angle in Range 15° to 30°

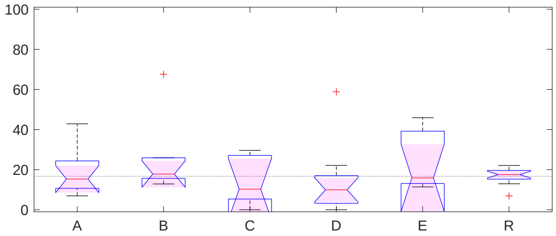

	A	B	C	D	E	R
A	-	.972	.995	.723	.999	.997
B	0.60	-	.861	.336	.999	.998
C	0.20	0.00	-	.993	.972	.937
D	0.20	0.00	0.40	-	.629	.298
E	0.50	0.33	0.60	0.69	-	.999
R	0.60	0.50	0.60	0.77	0.67	-

Edge 2 at ≤50 m — Angle in Range 30° to 45°

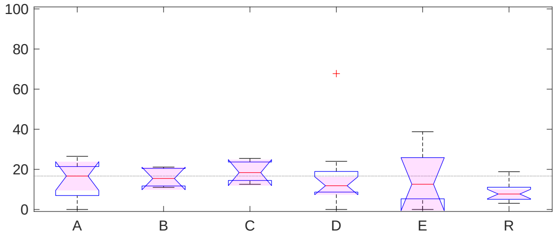

	A	B	C	D	E	R
A	-	.999	.900	.999	.999	.261
B	0.40	-	.990	.977	.990	.217
C	0.70	0.67	-	.729	.837	.055
D	0.30	0.33	0.00	-	.999	.403
E	0.30	0.33	0.20	0.54	-	.666
R	0.30	0.00	0.00	0.15	0.33	-

Edge 2 at ≤50 m — Angle in Range 45° to 60°

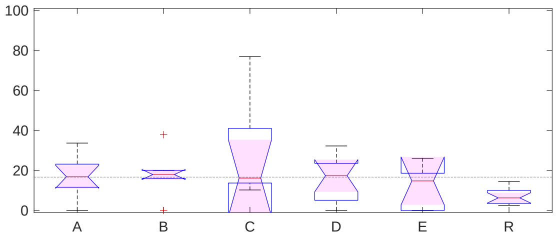

	A	B	C	D	E	R
A	-	.999	.993	.998	.955	.116
B	0.50	-	.999	.993	.936	.182
C	0.50	0.33	-	.934	.810	.097
D	0.50	0.33	0.60	-	.995	.223
E	0.40	0.17	0.20	0.38	-	.839
R	0.20	0.17	0.00	0.23	0.33	-

Edge 2 at ≤50 m — Angle in Range 60° to 75°

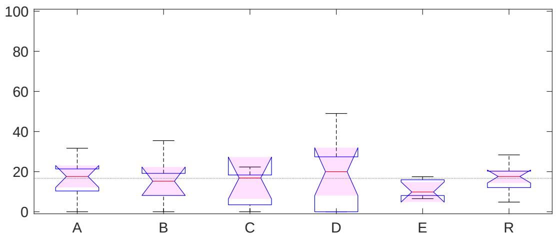

	A	B	C	D	E	R
A	-	.997	.958	.999	.754	.999
B	0.40	-	.999	.984	.968	.995
C	0.50	0.50	-	.892	.999	.939
D	0.70	0.83	0.80	-	.588	.999
E	0.20	0.33	0.40	0.31	-	.676
R	0.50	0.67	0.80	0.46	1.00	-

Edge 2 at ≤50 m — Angle in Range 75° to 90°

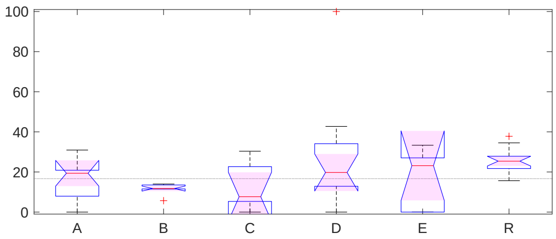

	A	B	C	D	E	R
A	-	.836	.997	.896	.999	.282
B	0.30	-	.991	.255	.706	.026
C	0.20	0.17	-	.760	.972	.252
D	0.50	1.00	0.60	-	.997	.889
E	0.80	1.00	0.80	0.54	-	.761
R	0.80	1.00	0.80	0.54	0.50	-

;

Edge 2 at ≤75 m

Edge 2 at ≤75 m — Time near Edge

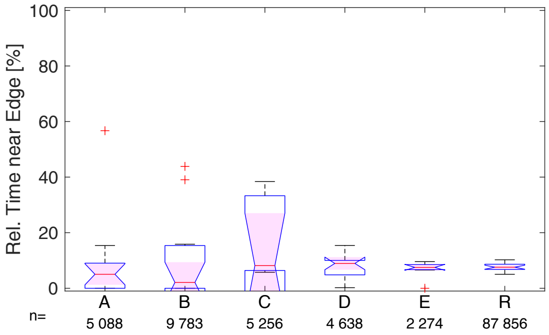

	A	B	C	D	E	R
A	-	.999	.747	.716	.998	.922
B	0.36	-	.831	.842	.999	.972
C	0.64	0.64	-	.999	.950	.984
D	0.71	0.64	0.60	-	.975	.996
E	0.57	0.64	0.40	0.38	-	.999
R	0.57	0.64	0.40	0.38	0.57	-

Edge 2 at ≤75 m — Angle in Range 0° to 15°

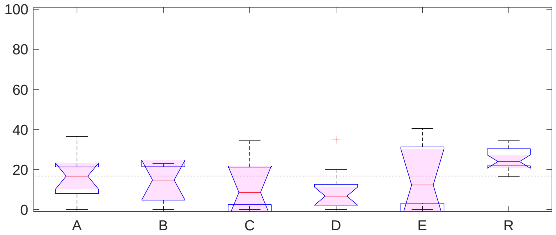

	A	B	C	D	E	R
A	-	.999	.996	.804	.999	.212
B	0.50	-	.999	.981	.999	.140
C	0.30	0.29	-	.997	.998	.187
D	0.20	0.29	0.40	-	.895	.002
E	0.40	0.43	0.60	0.77	-	.394
R	0.90	1.00	0.80	0.92	0.67	-

Edge 2 at ≤75 m — Angle in Range 15° to 30°

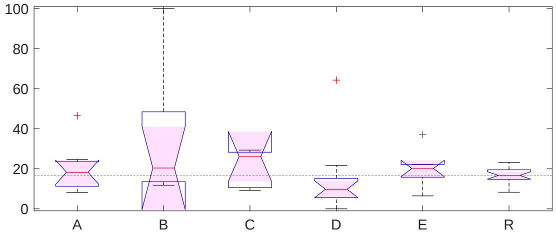

	A	B	C	D	E	R
A	-	.939	.992	.466	.997	.999
B	0.60	-	.999	.099	.999	.904
C	0.90	0.71	-	.317	.999	.987
D	0.20	0.00	0.20	-	.325	.348
E	0.60	0.43	0.40	0.85	-	.995
R	0.50	0.29	0.40	0.77	0.33	-

Edge 2 at ≤75 m — Angle in Range 30° to 45°

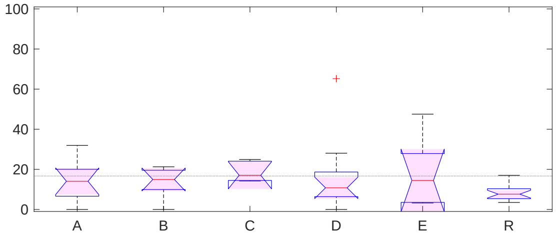

	A	B	C	D	E	R
A	-	.999	.815	.999	.999	.438
B	0.60	-	.918	.996	.999	.433
C	0.70	0.57	-	.631	.903	.061
D	0.30	0.29	0.00	-	.999	.580
E	0.60	0.43	0.20	0.62	-	.564
R	0.30	0.14	0.00	0.31	0.33	-

Edge 2 at ≤75 m — Angle in Range 45° to 60°

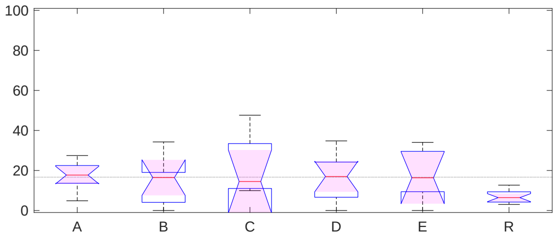

	A	B	C	D	E	R
A	-	.968	.999	.958	.998	.017
B	0.40	-	.982	.999	.999	.355
C	0.30	0.29	-	.980	.999	.110
D	0.50	0.57	0.60	-	.999	.126
E	0.40	0.29	0.60	0.46	-	.219
R	0.10	0.29	0.00	0.23	0.17	-

Edge 2 at ≤75 m — Angle in Range 60° to 75°

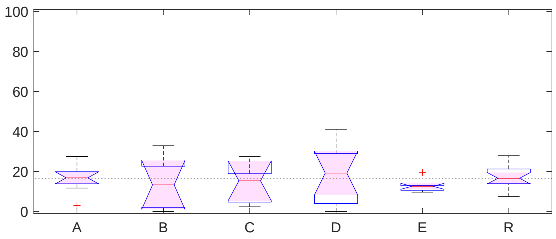

	A	B	C	D	E	R
A	-	.963	.982	.999	.788	.999
B	0.20	-	.999	.820	.997	.904
C	0.40	0.57	-	.897	.997	.951
D	0.60	0.71	0.80	-	.529	.999
E	0.20	0.43	0.40	0.31	-	.642
R	0.50	0.71	0.80	0.31	0.83	-

Edge 2 at ≤75 m — Angle in Range 75° to 90°

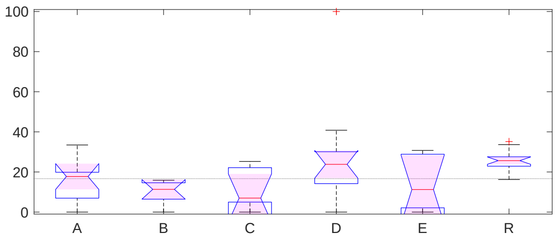

	A	B	C	D	E	R
A	-	.825	.993	.844	.999	.212
B	0.30	-	.996	.174	.922	.011
C	0.30	0.29	-	.640	.998	.162
D	0.80	1.00	0.80	-	.873	.881
E	0.30	0.43	0.60	0.23	-	.334
R	0.80	1.00	1.00	0.62	0.67	-

;

Edge 2 at ≤100 m

Edge 2 at ≤100 m — Time near Edge

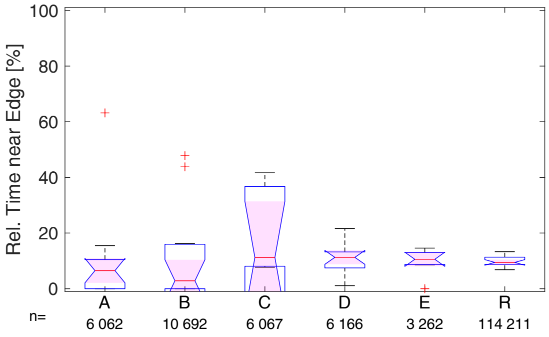

	A	B	C	D	E	R
A	-	.999	.664	.696	.911	.870
B	0.36	-	.754	.817	.953	.940
C	0.79	0.64	-	.998	.995	.980
D	0.79	0.64	0.60	-	.999	.999
E	0.79	0.64	0.40	0.38	-	.999
R	0.57	0.64	0.40	0.38	0.43	-

Edge 2 at ≤100 m — Angle in Range 0° to 15°

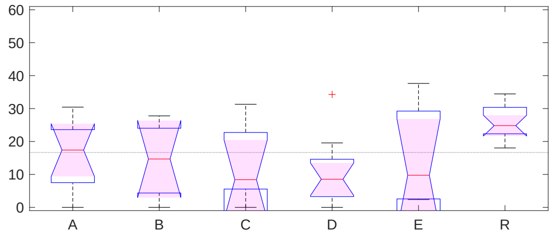

	A	B	C	D	E	R
A	-	.999	.999	.801	.999	.271
B	0.50	-	.999	.945	.999	.268
C	0.40	0.29	-	.991	.999	.284
D	0.40	0.29	0.60	-	.954	.003
E	0.40	0.43	0.60	0.54	-	.334
R	0.80	0.86	0.80	0.92	0.67	-

Edge 2 at ≤100 m — Angle in Range 15° to 30°

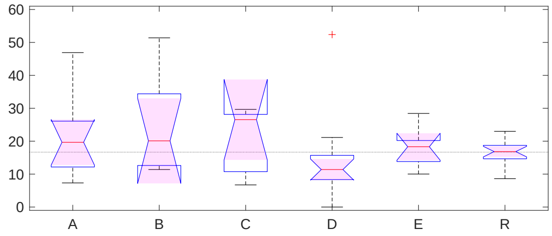

	A	B	C	D	E	R
A	-	.999	.999	.349	.999	.997
B	0.60	-	.999	.239	.999	.959
C	0.80	0.71	-	.446	.999	.990
D	0.20	0.00	0.20	-	.562	.508
E	0.40	0.43	0.40	0.77	-	.999
R	0.40	0.43	0.40	0.77	0.33	-

Edge 2 at ≤100 m — Angle in Range 30° to 45°

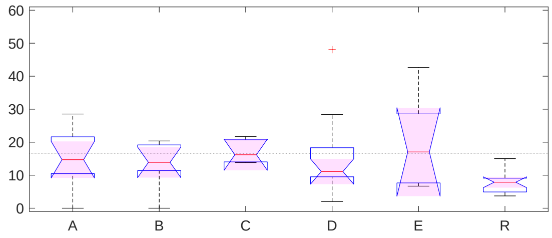

	A	B	C	D	E	R
A	-	.999	.943	.999	.999	.070
B	0.40	-	.951	.999	.999	.159
C	0.60	0.57	-	.834	.991	.022
D	0.30	0.14	0.00	-	.994	.101
E	0.70	0.71	0.60	0.69	-	.100
R	0.20	0.14	0.00	0.08	0.33	-

Edge 2 at ≤100 m — Angle in Range 45° to 60°

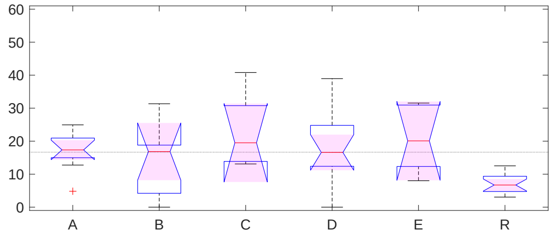

	A	B	C	D	E	R
A	-	.989	.985	.999	.999	.015
B	0.40	-	.857	.997	.974	.239
C	0.70	0.86	-	.957	.998	.014
D	0.30	0.29	0.40	-	.999	.013
E	0.70	0.86	0.60	0.69	-	.036
R	0.10	0.29	0.00	0.15	0.00	-

Edge 2 at ≤100 m — Angle in Range 60° to 75°

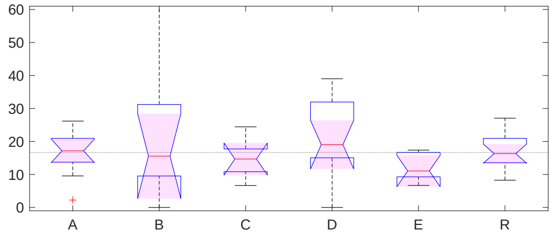

	A	B	C	D	E	R
A	-	.999	.985	.894	.750	.999
B	0.40	-	.978	.962	.744	.999
C	0.40	0.43	-	.630	.994	.946
D	0.60	0.57	0.80	-	.180	.933
E	0.20	0.29	0.20	0.23	-	.555
R	0.50	0.57	0.80	0.23	0.67	-

Edge 2 at ≤100 m — Angle in Range 75° to 90°

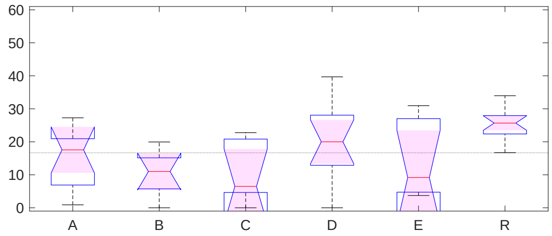

	A	B	C	D	E	R
A	-	.879	.979	.904	.999	.092
B	0.30	-	.999	.289	.950	.005
C	0.20	0.29	-	.607	.993	.051
D	0.70	1.00	0.60	-	.921	.558
E	0.30	0.43	0.60	0.23	-	.187
R	0.80	1.00	1.00	0.62	0.67	-

;

Edge 3 at ≤10 m

Edge 3 at ≤10 m — Time near Edge

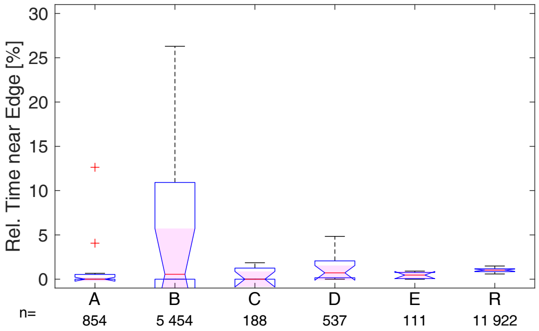

	A	B	C	D	E	R
A	-	.501	.998	.370	.999	.028
B	0.79	-	.951	.999	.877	.899
C	0.50	0.23	-	.919	.999	.507
D	0.86	0.55	0.60	-	.812	.925
E	0.71	0.45	0.60	0.38	-	.288
R	0.86	0.55	0.60	0.54	1.00	-

Edge 3 at ≤10 m — Angle in Range 0° to 15°

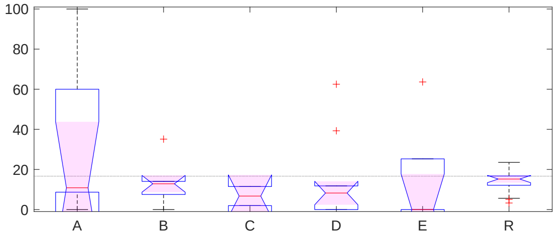

	A	B	C	D	E	R
A	-	.999	.968	.911	.927	.988
B	0.67	-	.983	.958	.963	.963
C	0.17	0.17	-	.999	.999	.799
D	0.17	0.33	0.50	-	.999	.305
E	0.08	0.08	0.00	0.20	-	.501
R	0.67	0.83	1.00	0.80	0.80	-

Edge 3 at ≤10 m — Angle in Range 15° to 30°

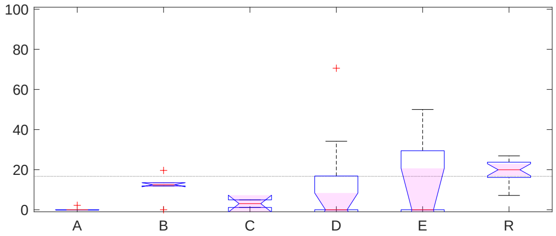

	A	B	C	D	E	R
A	-	.541	.975	.864	.717	.003
B	1.00	-	.998	.976	.999	.583
C	1.00	0.17	-	.999	.999	.656
D	0.50	0.08	0.00	-	.997	.046
E	0.50	0.08	0.00	0.50	-	.503
R	1.00	1.00	1.00	0.80	0.60	-

Edge 3 at ≤10 m — Angle in Range 30° to 45°

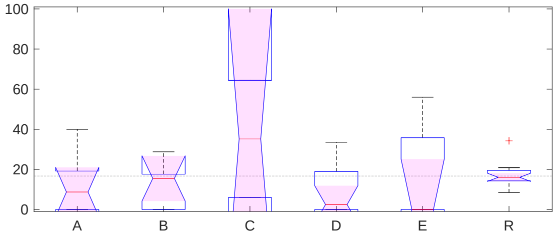

	A	B	C	D	E	R
A	-	.999	.944	.998	.999	.837
B	0.67	-	.970	.989	.999	.922
C	0.83	1.00	-	.814	.957	.999
D	0.33	0.33	0.00	-	.998	.356
E	0.17	0.17	0.00	0.25	-	.894
R	0.67	0.67	0.50	0.70	0.60	-

Edge 3 at ≤10 m — Angle in Range 45° to 60°

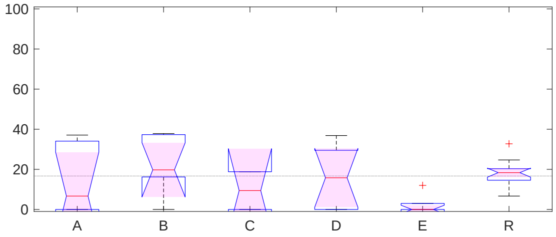

	A	B	C	D	E	R
A	-	.755	.999	.996	.691	.927
B	0.67	-	.869	.919	.066	.986
C	0.50	0.17	-	.995	.950	.966
D	0.67	0.17	0.50	-	.288	.997
E	0.25	0.08	0.25	0.15	-	.090
R	0.67	0.33	0.50	0.60	1.00	-

Edge 3 at ≤10 m — Angle in Range 60° to 75°

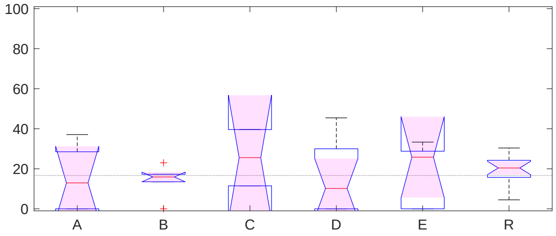

	A	B	C	D	E	R
A	-	.999	.966	.999	.997	.932
B	0.50	-	.948	.999	.992	.882
C	0.67	1.00	-	.983	.998	.999
D	0.50	0.17	0.00	-	.999	.963
E	0.67	1.00	0.50	0.60	-	.999
R	0.67	0.83	0.50	0.60	0.40	-

Edge 3 at ≤10 m — Angle in Range 75° to 90°

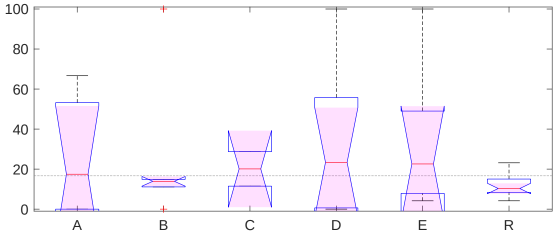

	A	B	C	D	E	R
A	-	.999	.998	.999	.998	.983
B	0.50	-	.997	.999	.997	.986
C	0.50	0.83	-	.999	.999	.938
D	0.67	0.83	0.50	-	.999	.891
E	0.67	0.83	0.50	0.50	-	.850
R	0.33	0.17	0.00	0.40	0.40	-

;

Edge 3 at ≤25 m

Edge 3 at ≤25 m — Time near Edge

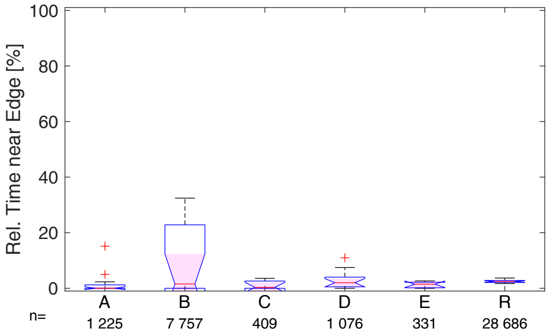

	A	B	C	D	E	R
A	-	.558	.999	.471	.996	.047
B	0.79	-	.953	.999	.948	.927
C	0.57	0.45	-	.938	.999	.559
D	0.79	0.55	0.60	-	.929	.930
E	0.79	0.45	0.60	0.46	-	.470
R	0.86	0.55	0.80	0.54	0.86	-

Edge 3 at ≤25 m — Angle in Range 0° to 15°

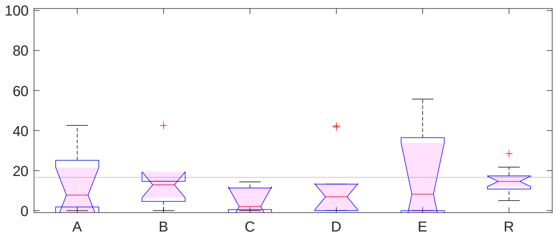

	A	B	C	D	E	R
A	-	.999	.978	.996	.999	.888
B	0.71	-	.931	.967	.999	.985
C	0.29	0.17	-	.999	.961	.607
D	0.29	0.33	0.67	-	.989	.451
E	0.57	0.33	0.67	0.60	-	.974
R	0.71	0.67	1.00	0.80	0.60	-

Edge 3 at ≤25 m — Angle in Range 15° to 30°

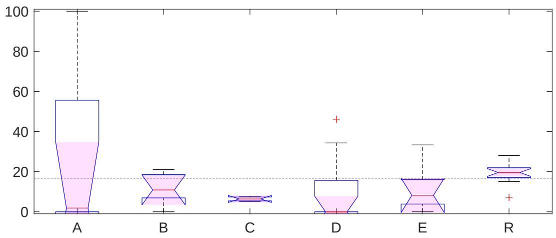

	A	B	C	D	E	R
A	-	.999	.996	.971	.999	.415
B	0.57	-	.990	.948	.999	.588
C	0.57	0.17	-	.999	.997	.389
D	0.21	0.08	0.00	-	.984	.027
E	0.57	0.33	1.00	0.70	-	.542
R	0.71	0.83	1.00	0.80	0.80	-

Edge 3 at ≤25 m — Angle in Range 30° to 45°

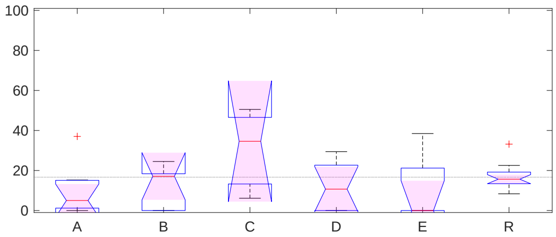

	A	B	C	D	E	R
A	-	.961	.495	.991	.999	.662
B	0.86	-	.900	.999	.968	.998
C	0.86	1.00	-	.731	.543	.960
D	0.57	0.33	0.33	-	.993	.930
E	0.14	0.17	0.00	0.15	-	.749
R	0.86	0.33	0.33	0.60	0.80	-

Edge 3 at ≤25 m — Angle in Range 45° to 60°

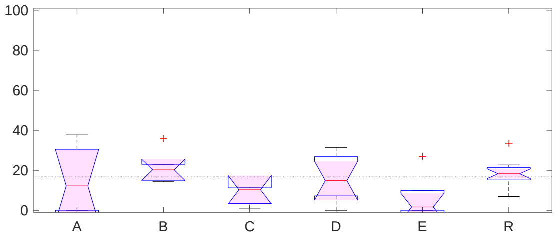

	A	B	C	D	E	R
A	-	.863	.906	.999	.902	.930
B	0.57	-	.396	.960	.308	.998
C	0.43	0.00	-	.732	.999	.448
D	0.57	0.33	1.00	-	.673	.992
E	0.43	0.00	0.33	0.20	-	.317
R	0.57	0.33	1.00	0.60	0.80	-

Edge 3 at ≤25 m — Angle in Range 60° to 75°

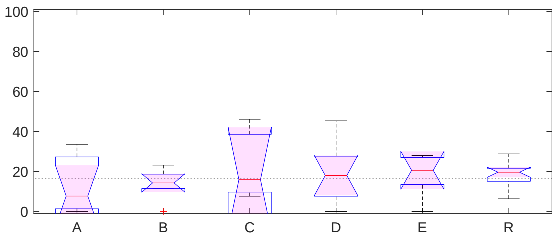

	A	B	C	D	E	R
A	-	.999	.998	.984	.973	.976
B	0.57	-	.979	.908	.895	.874
C	0.57	0.50	-	.999	.999	.999
D	0.57	0.67	0.67	-	.999	.999
E	0.57	0.83	0.67	0.50	-	.999
R	0.57	0.83	0.67	0.50	0.40	-

Edge 3 at ≤25 m — Angle in Range 75° to 90°

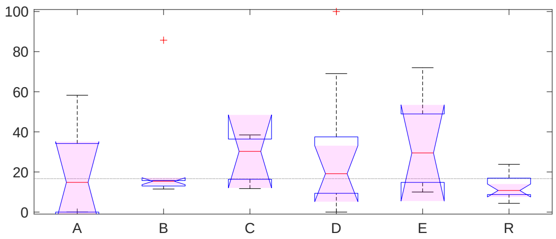

	A	B	C	D	E	R
A	-	.971	.842	.963	.657	.997
B	0.57	-	.994	.999	.976	.750
C	0.71	0.83	-	.989	.999	.562
D	0.57	0.83	0.33	-	.953	.629
E	0.71	0.83	0.33	0.70	-	.259
R	0.43	0.00	0.00	0.30	0.20	-

;

Edge 3 at ≤50 m

Edge 3 at ≤50 m — Time near Edge

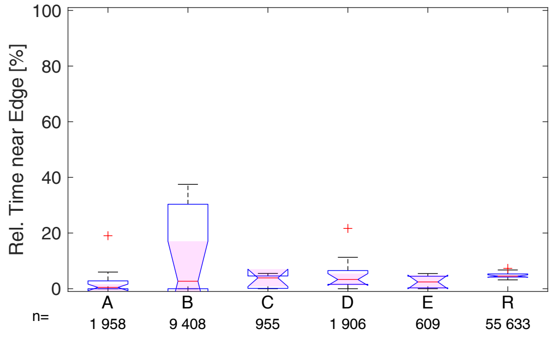

	A	B	C	D	E	R
A	-	.713	.992	.571	.999	.058
B	0.71	-	.996	.999	.961	.861
C	0.79	0.55	-	.989	.999	.726
D	0.79	0.55	0.40	-	.922	.902
E	0.71	0.45	0.40	0.46	-	.411
R	0.79	0.55	0.80	0.62	0.71	-

Edge 3 at ≤50 m — Angle in Range 0° to 15°

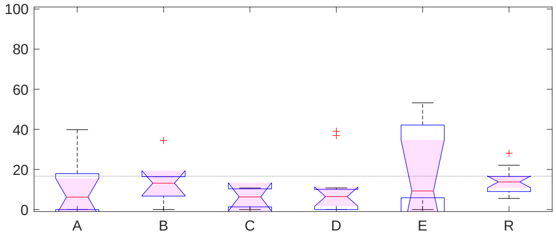

	A	B	C	D	E	R
A	-	.894	.999	.999	.887	.511
B	0.78	-	.826	.795	.999	.999
C	0.56	0.17	-	.999	.817	.536
D	0.56	0.17	0.50	-	.793	.307
E	0.56	0.33	0.50	0.73	-	.999
R	0.78	0.50	1.00	0.82	0.60	-

Edge 3 at ≤50 m — Angle in Range 15° to 30°

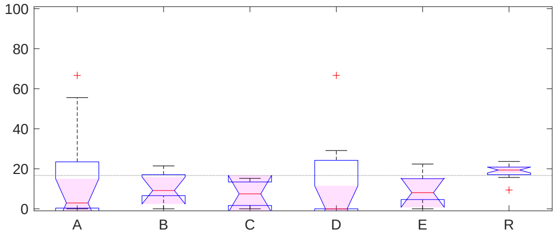

	A	B	C	D	E	R
A	-	.999	.998	.999	.999	.219
B	0.67	-	.995	.996	.999	.489
C	0.67	0.50	-	.999	.998	.281
D	0.11	0.08	0.13	-	.999	.056
E	0.67	0.50	0.50	0.64	-	.505
R	0.78	0.83	1.00	0.73	0.80	-

Edge 3 at ≤50 m — Angle in Range 30° to 45°

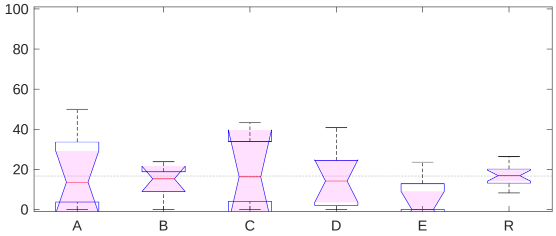

	A	B	C	D	E	R
A	-	.999	.999	.999	.753	.994
B	0.67	-	.999	.999	.867	.989
C	0.67	0.67	-	.999	.809	.999
D	0.56	0.33	0.50	-	.744	.989
E	0.11	0.08	0.13	0.14	-	.387
R	0.67	0.67	0.50	0.55	0.80	-

Edge 3 at $\leq 50\,\mathrm{m}$ — Angle in Range 45° to 60°

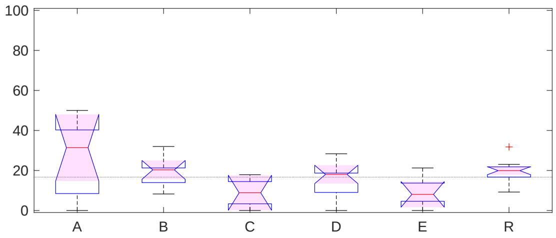

	A	B	C	D	E	R
A	-	.999	.278	.740	.296	.999
B	0.44	-	.422	.886	.461	.999
C	0.22	0.17	-	.874	.999	.272
D	0.44	0.33	1.00	-	.916	.744
E	0.22	0.00	0.50	0.18	-	.284
R	0.44	0.33	1.00	0.82	0.80	-

Edge 3 at $\leq 50\,\mathrm{m}$ — Angle in Range 60° to 75°

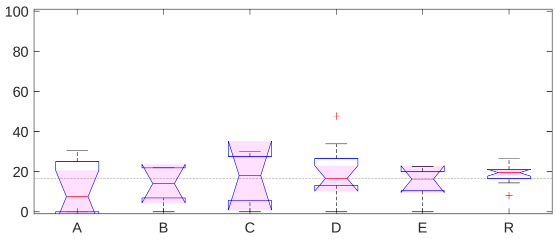

	A	B	C	D	E	R
A	-	.999	.999	.995	.999	.918
B	0.56	-	.984	.955	.999	.794
C	0.56	0.67	-	.999	.998	.999
D	0.56	0.67	0.50	-	.994	.998
E	0.56	0.67	0.50	0.45	-	.941
R	0.56	0.67	0.50	0.73	0.80	-

Edge 3 at $\leq 50\,\mathrm{m}$ — Angle in Range 75° to 90°

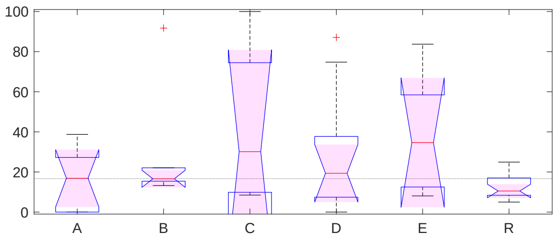

	A	B	C	D	E	R
A	-	.873	.887	.960	.753	.999
B	0.44	-	.999	.998	.999	.643
C	0.78	0.83	-	.997	.999	.713
D	0.56	0.67	0.50	-	.982	.768
E	0.89	0.83	0.50	0.73	-	.495
R	0.44	0.00	0.25	0.27	0.20	-

;

Edge 3 at $\leq 75\,\mathrm{m}$

Edge 3 at $\leq 75\,\mathrm{m}$ — Time near Edge

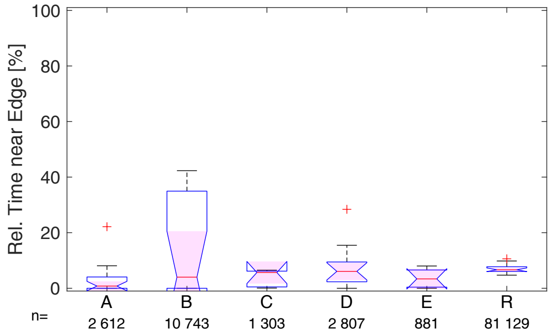

	A	B	C	D	E	R
A	-	.588	.994	.360	.997	.034
B	0.71	-	.985	.999	.949	.872
C	0.79	0.55	-	.949	.999	.624
D	0.79	0.55	0.80	-	.861	.949
E	0.71	0.45	0.40	0.38	-	.389
R	0.79	0.55	1.00	0.54	0.71	-

Edge 3 at $\leq 75\,\mathrm{m}$ — Angle in Range 0° to 15°

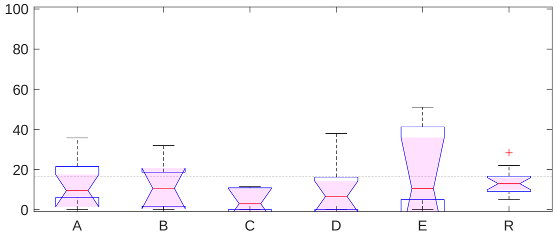

	A	B	C	D	E	R
A	-	.999	.676	.913	.999	.999
B	0.56	-	.895	.995	.993	.966
C	0.22	0.29	-	.984	.650	.382
D	0.22	0.43	0.60	-	.882	.610
E	0.56	0.43	0.60	0.73	-	.999
R	0.56	0.57	1.00	0.73	0.60	-

Edge 3 at $\leq 75\,\mathrm{m}$ — Angle in Range 15° to 30°

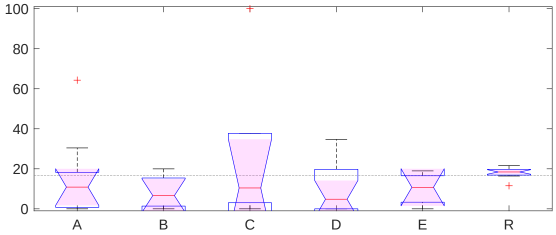

	A	B	C	D	E	R
A	-	.999	.999	.999	.999	.268
B	0.44	-	.998	.999	.999	.175
C	0.44	0.57	-	.998	.999	.627
D	0.44	0.29	0.40	-	.999	.091
E	0.44	0.57	0.60	0.73	-	.379
R	0.78	0.86	0.80	0.73	0.80	-

Edge 3 at $\leq 75\,\mathrm{m}$ — Angle in Range 30° to 45°

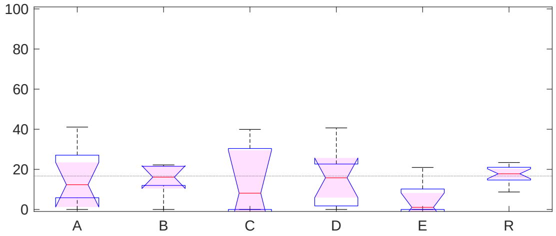

	A	B	C	D	E	R
A	-	.999	.999	.999	.643	.993
B	0.56	-	.999	.999	.694	.995
C	0.33	0.14	-	.999	.886	.970
D	0.56	0.43	0.60	-	.671	.978
E	0.22	0.14	0.40	0.27	-	.272
R	0.67	0.57	0.60	0.64	0.80	-

Edge 3 at ≤75 m — Angle in Range 45° to 60°

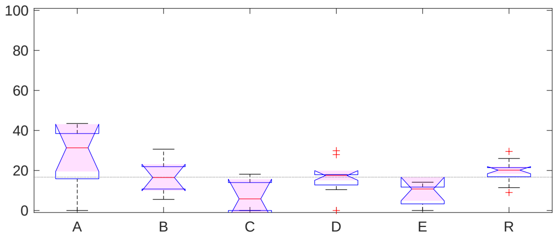

	A	B	C	D	E	R
A	-	.816	.048	.509	.037	.991
B	0.22	-	.559	.999	.500	.962
C	0.11	0.14	-	.653	.999	.090
D	0.22	0.57	0.80	-	.589	.756
E	0.22	0.29	0.60	0.18	-	.069
R	0.44	0.71	1.00	0.82	1.00	-

Edge 3 at ≤75 m — Angle in Range 60° to 75°

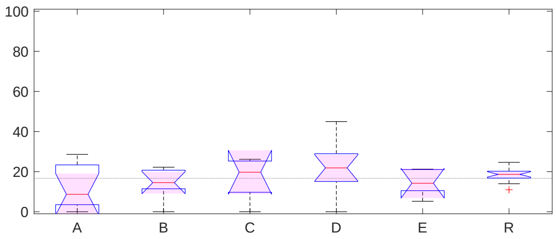

	A	B	C	D	E	R
A	-	.999	.938	.329	.999	.876
B	0.67	-	.982	.565	.999	.971
C	0.67	0.57	-	.979	.988	.999
D	0.67	0.86	0.60	-	.677	.866
E	0.67	0.43	0.40	0.27	-	.983
R	0.67	0.57	0.40	0.27	0.60	-

Edge 3 at ≤75 m — Angle in Range 75° to 90°

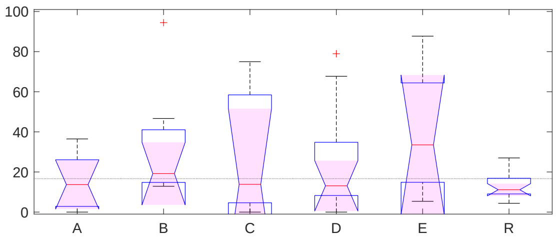

	A	B	C	D	E	R
A	-	.607	.998	.991	.614	.999
B	0.67	-	.927	.882	.999	.388
C	0.56	0.14	-	.999	.910	.992
D	0.44	0.14	0.40	-	.868	.954
E	0.89	0.71	0.60	0.73	-	.430
R	0.33	0.00	0.40	0.45	0.20	-

;

Edge 3 at $\leq 100\,\mathrm{m}$

Edge 3 at $\leq 100\,\mathrm{m}$ — Time near Edge

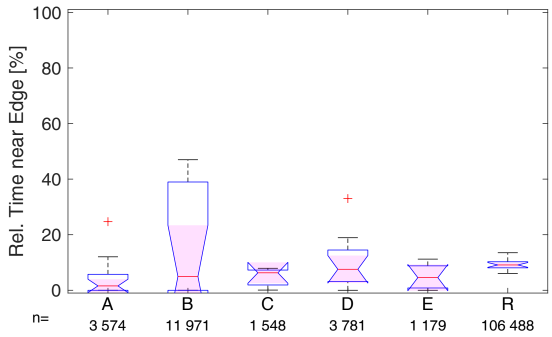

	A	B	C	D	E	R
A	-	.572	.995	.264	.991	.030
B	0.71	-	.980	.999	.969	.864
C	0.79	0.55	-	.900	.999	.579
D	0.79	0.55	0.80	-	.851	.975
E	0.71	0.45	0.40	0.38	-	.446
R	0.86	0.55	1.00	0.54	0.71	-

Edge 3 at $\leq 100\,\mathrm{m}$ — Angle in Range 0° to 15°

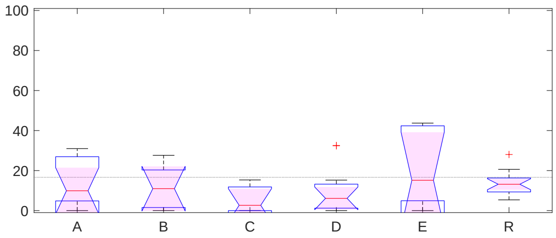

	A	B	C	D	E	R
A	-	.999	.690	.891	.999	.999
B	0.56	-	.904	.992	.988	.977
C	0.22	0.29	-	.990	.601	.439
D	0.22	0.29	0.60	-	.810	.621
E	0.56	0.57	0.80	0.73	-	.999
R	0.56	0.57	0.80	0.73	0.50	-

Edge 3 at $\leq 100\,\mathrm{m}$ — Angle in Range 15° to 30°

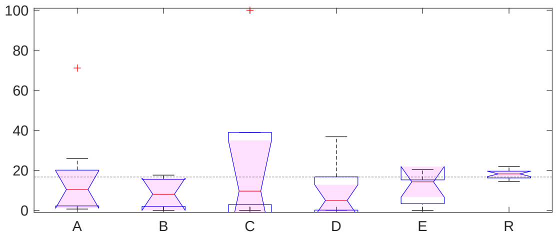

	A	B	C	D	E	R
A	-	.962	.999	.978	.999	.451
B	0.44	-	.971	.999	.993	.097
C	0.44	0.57	-	.984	.999	.722
D	0.44	0.29	0.40	-	.998	.060
E	0.67	0.71	0.60	0.73	-	.460
R	0.78	1.00	0.60	0.73	0.83	-

Edge 3 at $\leq 100\,\mathrm{m}$ — Angle in Range 30° to 45°

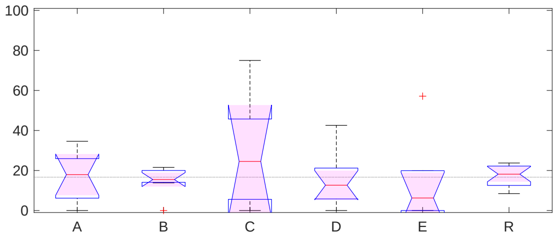

	A	B	C	D	E	R
A	-	.999	.988	.999	.922	.998
B	0.44	-	.972	.999	.973	.990
C	0.67	1.00	-	.937	.696	.999
D	0.44	0.14	0.40	-	.978	.957
E	0.22	0.14	0.20	0.27	-	.677
R	0.56	0.57	0.40	0.64	0.67	-

Edge 3 at ≤100 m — Angle in Range 45° to 60°

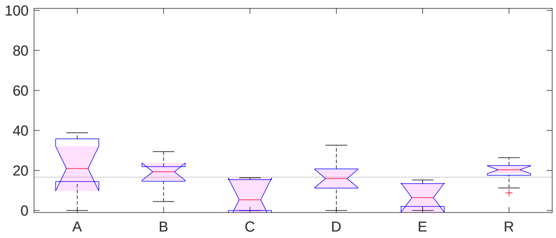

	A	B	C	D	E	R
A	-	.966	.070	.708	.028	.999
B	0.33	-	.401	.997	.258	.993
C	0.11	0.14	-	.581	.999	.080
D	0.33	0.29	0.80	-	.402	.808
E	0.11	0.14	0.60	0.09	-	.028
R	0.33	0.71	1.00	0.73	1.00	-

Edge 3 at ≤100 m — Angle in Range 60° to 75°

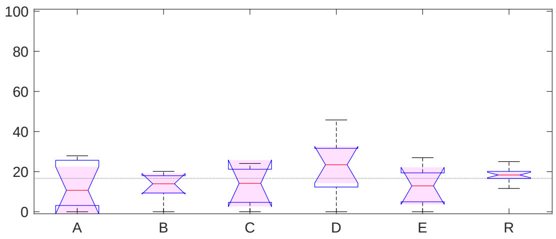

	A	B	C	D	E	R
A	-	.979	.999	.618	.997	.982
B	0.56	-	.998	.232	.999	.698
C	0.56	0.57	-	.641	.999	.966
D	0.67	1.00	0.80	-	.422	.898
E	0.56	0.43	0.40	0.27	-	.875
R	0.56	0.71	0.60	0.27	0.67	-

Edge 3 at ≤100 m — Angle in Range 75° to 90°

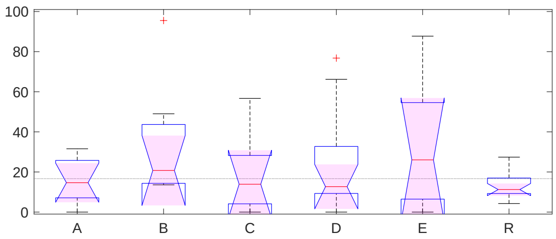

	A	B	C	D	E	R
A	-	.677	.999	.997	.974	.998
B	0.67	-	.714	.876	.989	.325
C	0.44	0.29	-	.994	.967	.999
D	0.44	0.00	0.40	-	.999	.926
E	0.78	0.57	0.80	0.55	-	.838
R	0.44	0.00	0.40	0.36	0.33	-

;

Edge 4 at ≤10 m

Edge 4 at ≤10 m — Time near Edge

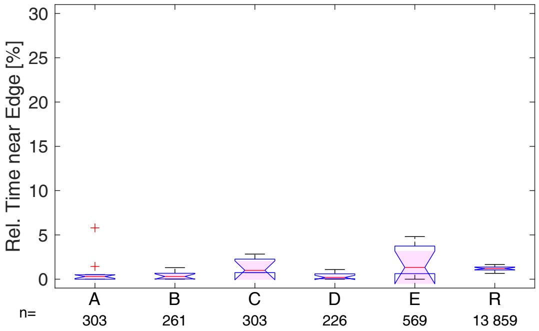

	A	B	C	D	E	R
A	-	.999	.143	.999	.140	.003
B	0.57	-	.137	.999	.137	.004
C	0.86	0.91	-	.093	.999	.999
D	0.36	0.45	0.00	-	.087	.001
E	0.86	1.00	0.60	1.00	-	.997
R	0.86	0.91	0.60	1.00	0.43	-

Edge 4 at ≤10 m — Angle in Range 0° to 15°

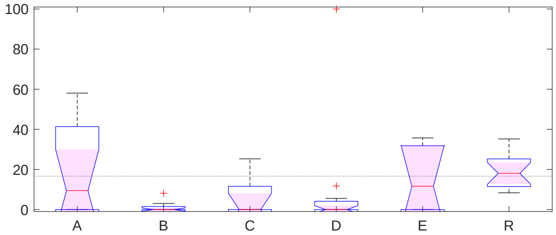

	A	B	C	D	E	R
A	-	.227	.828	.415	.999	.824
B	0.20	-	.984	.996	.353	.005
C	0.20	0.50	-	.999	.871	.208
D	0.20	0.50	0.50	-	.567	.010
E	0.50	1.00	0.80	0.82	-	.918
R	0.60	1.00	0.80	0.91	0.67	-

Edge 4 at ≤10 m — Angle in Range 15° to 30°

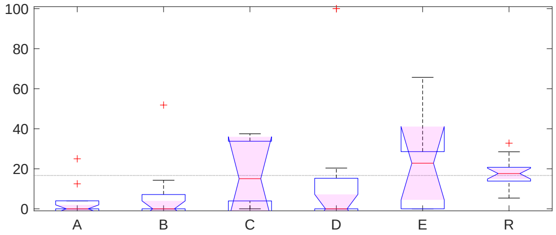

	A	B	C	D	E	R
A	-	.999	.427	.990	.291	.028
B	0.50	-	.567	.998	.432	.084
C	0.90	0.88	-	.736	.999	.998
D	0.50	0.50	0.10	-	.602	.141
E	0.90	0.88	0.60	0.82	-	.999
R	0.90	0.88	0.60	0.73	0.33	-

Edge 4 at ≤10 m — Angle in Range 30° to 45°

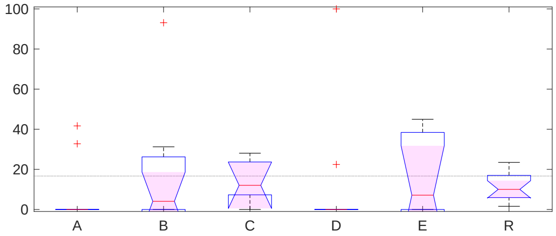

	A	B	C	D	E	R
A	-	.870	.447	.999	.813	.177
B	0.80	-	.964	.806	.999	.935
C	0.80	0.63	-	.370	.992	.999
D	0.50	0.25	0.10	-	.744	.112
E	0.80	0.50	0.20	0.82	-	.989
R	0.80	0.63	0.40	0.82	0.50	-

Edge 4 at ≤10 m — Angle in Range 45° to 60°

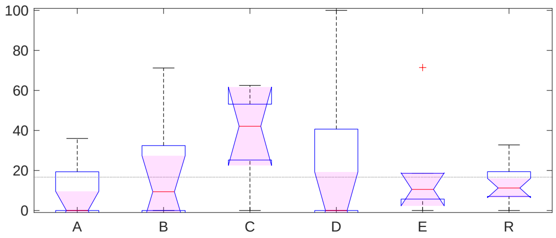

	A	B	C	D	E	R
A	-	.938	.160	.928	.869	.625
B	0.70	-	.652	.999	.999	.998
C	1.00	0.75	-	.570	.838	.770
D	0.50	0.25	0.10	-	.999	.995
E	0.70	0.50	0.20	0.55	-	.999
R	0.70	0.50	0.20	0.55	0.50	-

Edge 4 at ≤10 m — Angle in Range 60° to 75°

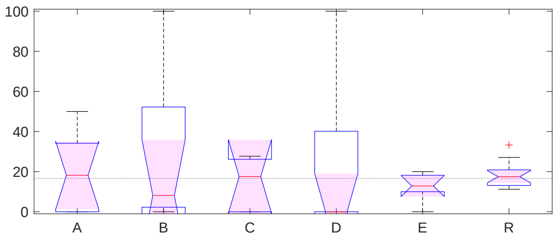

	A	B	C	D	E	R
A	-	.999	.999	.968	.995	.997
B	0.40	-	.999	.997	.999	.979
C	0.50	0.63	-	.999	.999	.987
D	0.20	0.13	0.20	-	.999	.744
E	0.40	0.63	0.40	0.64	-	.927
R	0.50	0.63	0.40	0.64	0.67	-

Edge 4 at ≤10 m — Angle in Range 75° to 90°

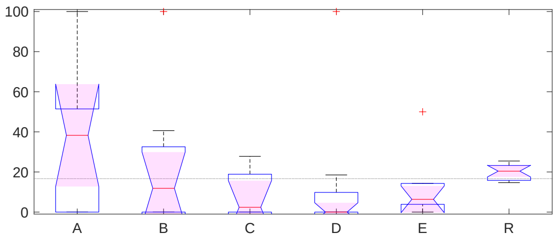

	A	B	C	D	E	R
A	-	.803	.499	.033	.613	.998
B	0.30	-	.989	.652	.999	.925
C	0.30	0.38	-	.987	.999	.649
D	0.15	0.19	0.20	-	.928	.045
E	0.30	0.50	0.60	0.73	-	.767
R	0.30	0.63	0.80	0.91	0.83	-

;

Edge 4 at ≤25 m

Edge 4 at ≤25 m — Time near Edge

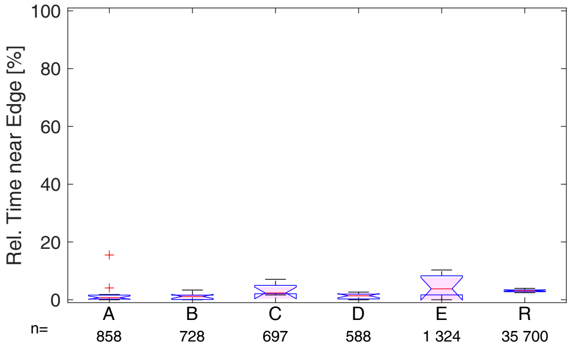

	A	B	C	D	E	R
A	-	.999	.225	.999	.097	.004
B	0.57	-	.171	.999	.071	.003
C	0.86	0.82	-	.226	.999	.999
D	0.71	0.73	0.00	-	.099	.004
E	0.86	1.00	0.60	1.00	-	.999
R	0.86	0.91	0.60	1.00	0.43	-

Edge 4 at ≤25 m — Angle in Range 0° to 15°

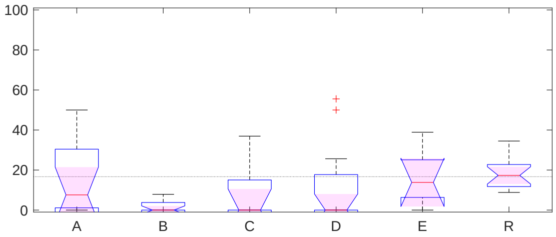

	A	B	C	D	E	R
A	-	.312	.918	.926	.997	.738
B	0.14	-	.979	.837	.229	.007
C	0.14	0.50	-	.999	.788	.282
D	0.14	0.50	0.50	-	.785	.129
E	0.64	1.00	0.80	0.75	-	.989
R	0.64	1.00	0.80	0.75	0.50	-

Edge 4 at ≤25 m — Angle in Range 15° to 30°

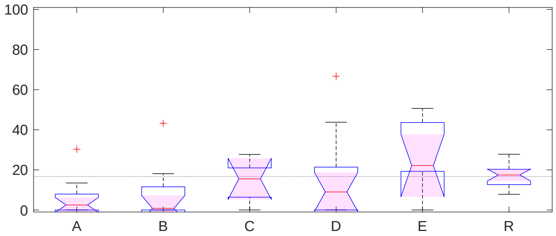

	A	B	C	D	E	R
A	-	.999	.725	.834	.055	.069
B	0.45	-	.855	.948	.133	.213
C	0.91	0.75	-	.997	.878	.990
D	0.73	0.75	0.40	-	.433	.674
E	0.91	0.88	0.80	0.75	-	.976
R	0.91	0.75	0.60	0.67	0.17	-

Edge 4 at ≤25 m — Angle in Range 30° to 45°

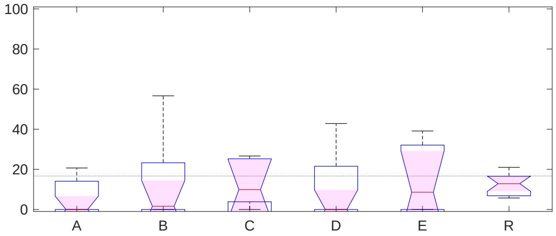

	A	B	C	D	E	R
A	-	.996	.872	.999	.966	.563
B	0.55	-	.987	.998	.999	.934
C	0.64	0.63	-	.892	.999	.999
D	0.50	0.25	0.10	-	.975	.593
E	0.64	0.63	0.40	0.67	-	.996
R	0.73	0.63	0.60	0.67	0.50	-

Edge 4 at ≤25 m — Angle in Range 45° to 60°

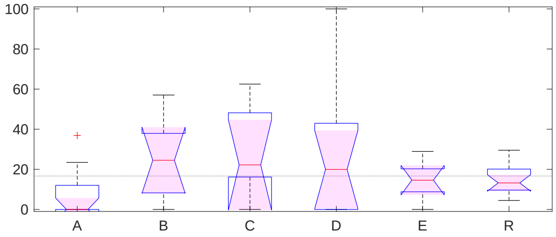

	A	B	C	D	E	R
A	-	.248	.231	.477	.890	.591
B	0.91	-	.999	.992	.956	.952
C	0.82	0.38	-	.959	.894	.881
D	0.82	0.38	0.20	-	.999	.999
E	0.82	0.25	0.20	0.42	-	.999
R	0.82	0.25	0.20	0.42	0.33	-

Edge 4 at ≤25 m — Angle in Range 60° to 75°

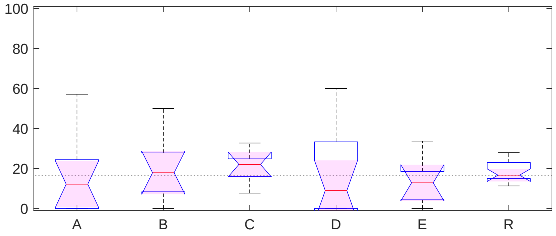

	A	B	C	D	E	R
A	-	.995	.943	.999	.999	.966
B	0.55	-	.998	.995	.986	.999
C	0.64	0.63	-	.944	.918	.999
D	0.36	0.38	0.20	-	.999	.966
E	0.55	0.38	0.20	0.58	-	.947
R	0.55	0.38	0.20	0.58	0.67	-

Edge 4 at ≤25 m — Angle in Range 75° to 90°

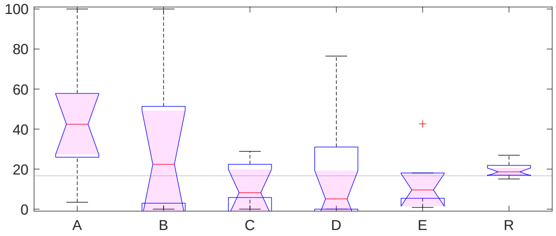

	A	B	C	D	E	R
A	-	.750	.198	.023	.162	.451
B	0.27	-	.898	.691	.894	.999
C	0.09	0.38	-	.999	.999	.912
D	0.09	0.38	0.20	-	.999	.645
E	0.18	0.38	0.60	0.50	-	.906
R	0.18	0.38	0.60	0.75	0.83	-

;

Edge 4 at $\leq 50\,\mathrm{m}$

Edge 4 at $\leq 50\,\mathrm{m}$ — Time near Edge

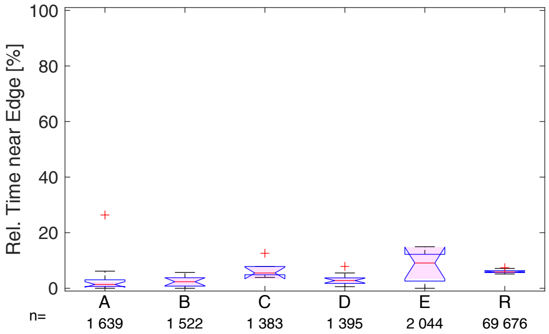

	A	B	C	D	E	R
A	-	.999	.163	.992	.124	.001
B	0.64	-	.195	.994	.159	.003
C	0.79	0.91	-	.382	.999	.999
D	0.71	0.64	0.00	-	.347	.014
E	0.93	1.00	0.80	1.00	-	.989
R	0.86	1.00	0.60	0.92	0.43	-

Edge 4 at $\leq 50\,\mathrm{m}$ — Angle in Range 0° to 15°

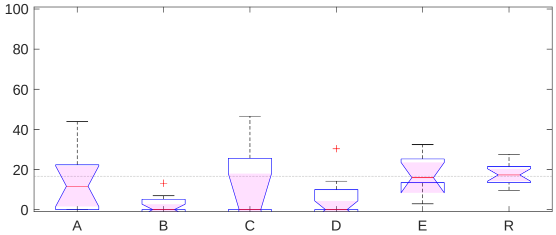

	A	B	C	D	E	R
A	-	.296	.988	.477	.915	.717
B	0.17	-	.906	.997	.075	.006
C	0.17	0.50	-	.981	.728	.517
D	0.17	0.50	0.50	-	.133	.010
E	0.50	1.00	0.60	0.92	-	.999
R	0.58	1.00	0.60	0.92	0.50	-

Edge 4 at $\leq 50\,\mathrm{m}$ — Angle in Range 15° to 30°

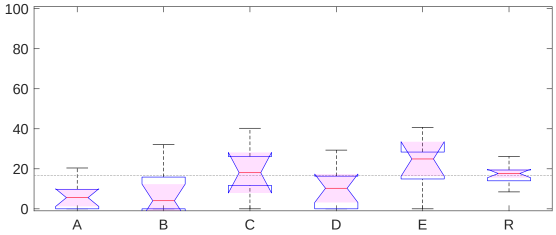

	A	B	C	D	E	R
A	-	.990	.316	.940	.077	.037
B	0.50	-	.686	.999	.323	.311
C	0.92	0.78	-	.748	.998	.999
D	0.83	0.67	0.20	-	.359	.326
E	1.00	0.78	0.80	0.85	-	.998
R	0.92	0.78	0.40	0.85	0.33	-

Edge 4 at $\leq 50\,\mathrm{m}$ — Angle in Range 30° to 45°

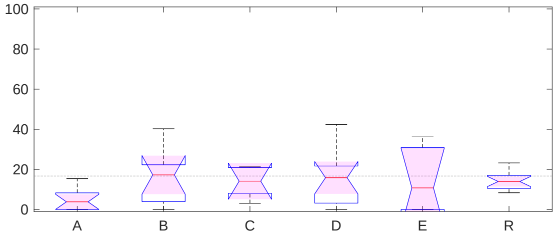

	A	B	C	D	E	R
A	-	.221	.342	.107	.565	.053
B	1.00	-	.999	.999	.999	.999
C	0.92	0.44	-	.999	.999	.999
D	1.00	0.44	0.60	-	.999	.999
E	0.83	0.44	0.40	0.38	-	.995
R	0.92	0.44	0.40	0.46	0.50	-

Edge 4 at ≤50 m — Angle in Range 45° to 60°

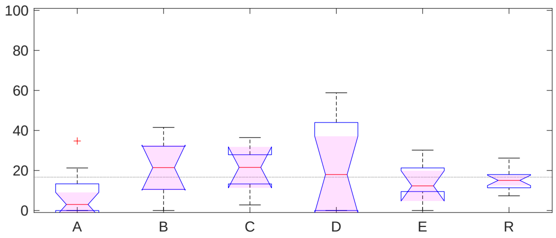

	A	B	C	D	E	R
A	-	.130	.280	.173	.856	.370
B	0.92	-	.999	.999	.926	.961
C	0.92	0.56	-	.999	.946	.977
D	0.83	0.33	0.40	-	.978	.995
E	0.67	0.33	0.20	0.38	-	.999
R	0.83	0.33	0.20	0.38	0.67	-

Edge 4 at ≤50 m — Angle in Range 60° to 75°

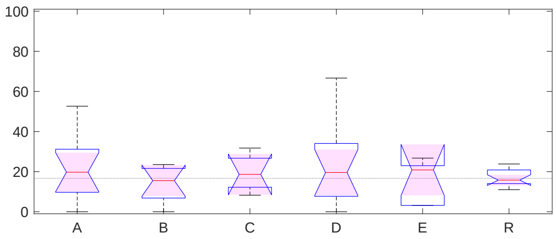

	A	B	C	D	E	R
A	-	.867	.999	.999	.998	.951
B	0.33	-	.981	.919	.995	.999
C	0.33	0.56	-	.999	.999	.998
D	0.50	0.67	0.60	-	.999	.980
E	0.58	0.67	0.60	0.54	-	.999
R	0.33	0.56	0.40	0.38	0.33	-

Edge 4 at ≤50 m — Angle in Range 75° to 90°

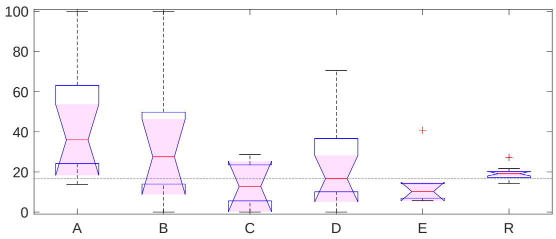

	A	B	C	D	E	R
A	-	.864	.143	.196	.024	.312
B	0.25	-	.712	.931	.348	.985
C	0.00	0.22	-	.980	.998	.917
D	0.17	0.33	0.60	-	.795	.999
E	0.00	0.22	0.40	0.23	-	.596
R	0.25	0.44	0.60	0.62	0.83	-

;

Edge 4 at ≤75 m

Edge 4 at ≤75 m — Time near Edge

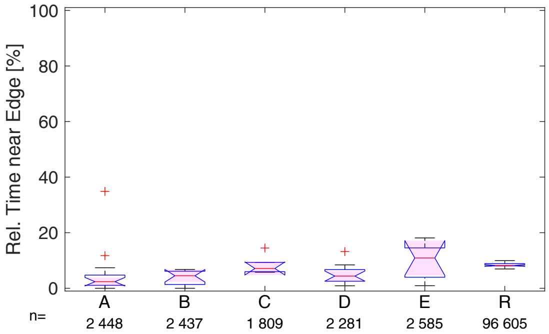

	A	B	C	D	E	R
A	-	.999	.426	.962	.094	.001
B	0.71	-	.435	.959	.108	.002
C	0.79	1.00	-	.820	.999	.940
D	0.71	0.45	0.00	-	.403	.031
E	0.86	1.00	0.80	0.92	-	.996
R	0.86	1.00	0.80	0.85	0.43	-

Edge 4 at ≤75 m — Angle in Range 0° to 15°

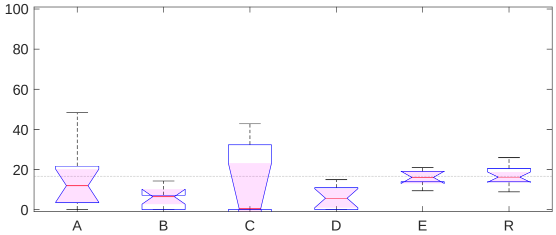

	A	B	C	D	E	R
A	-	.313	.999	.270	.882	.661
B	0.25	-	.796	.999	.052	.005
C	0.25	0.33	-	.807	.828	.668
D	0.25	0.44	0.60	-	.039	.002
E	0.67	1.00	0.60	1.00	-	.999
R	0.67	1.00	0.60	1.00	0.57	-

Edge 4 at ≤75 m — Angle in Range 15° to 30°

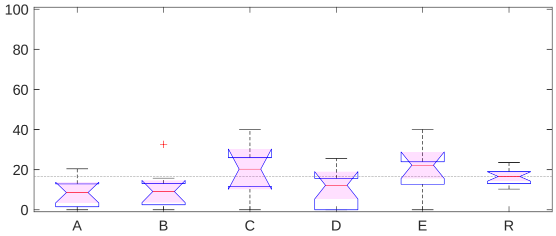

	A	B	C	D	E	R
A	-	.999	.264	.971	.112	.082
B	0.50	-	.412	.996	.229	.226
C	0.92	0.89	-	.612	.999	.999
D	0.67	0.78	0.20	-	.393	.406
E	1.00	0.89	0.80	0.92	-	.998
R	0.83	0.89	0.40	0.77	0.43	-

Edge 4 at ≤75 m — Angle in Range 30° to 45°

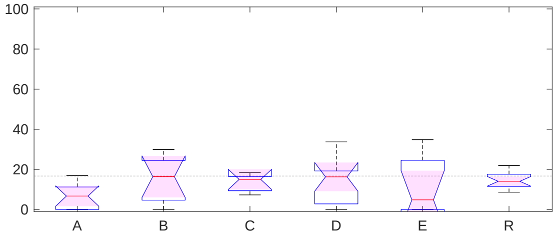

	A	B	C	D	E	R
A	-	.472	.673	.511	.960	.150
B	0.83	-	.999	.999	.972	.999
C	0.83	0.44	-	.999	.986	.999
D	0.83	0.44	0.80	-	.989	.990
E	0.42	0.22	0.00	0.38	-	.858
R	0.83	0.44	0.40	0.46	0.57	-

Edge 4 at ≤75 m — Angle in Range 45° to 60°

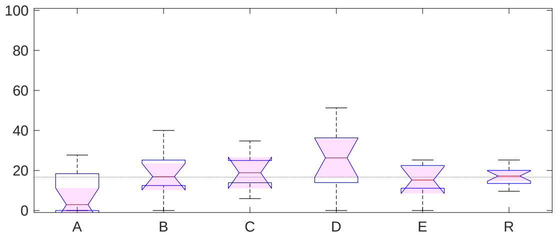

	A	B	C	D	E	R
A	-	.538	.532	.052	.953	.610
B	0.67	-	.999	.947	.987	.999
C	0.75	0.56	-	.998	.961	.992
D	0.92	0.78	0.80	-	.643	.716
E	0.67	0.44	0.20	0.23	-	.998
R	0.67	0.56	0.40	0.38	0.71	-

Edge 4 at ≤75 m — Angle in Range 60° to 75°

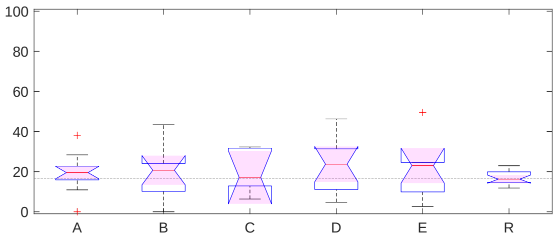

	A	B	C	D	E	R
A	-	.999	.999	.989	.999	.952
B	0.67	-	.999	.941	.989	.998
C	0.33	0.44	-	.999	.999	.971
D	0.75	0.78	0.60	-	.999	.615
E	0.75	0.67	0.60	0.46	-	.874
R	0.25	0.44	0.40	0.38	0.29	-

Edge 4 at ≤75 m — Angle in Range 75° to 90°

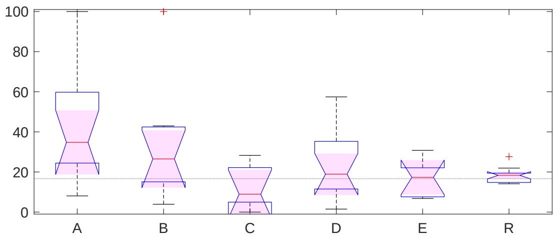

	A	B	C	D	E	R
A	-	.916	.063	.344	.131	.083
B	0.25	-	.438	.964	.693	.743
C	0.08	0.11	-	.797	.996	.949
D	0.17	0.33	0.60	-	.966	.992
E	0.17	0.33	0.60	0.38	-	.999
R	0.17	0.33	0.60	0.46	0.57	-

;

Edge 4 at ≤100 m

Edge 4 at ≤100 m — Time near Edge

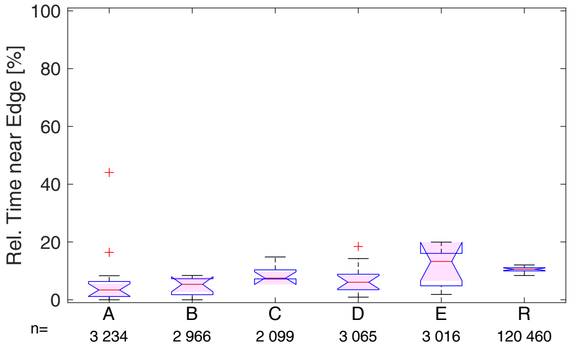

	A	B	C	D	E	R
A	-	.999	.562	.859	.113	.002
B	0.71	-	.550	.846	.120	.004
C	0.79	0.82	-	.969	.995	.911
D	0.71	0.55	0.00	-	.625	.119
E	0.86	1.00	0.80	0.85	-	.997
R	0.86	1.00	0.80	0.77	0.43	-

Edge 4 at ≤100 m — Angle in Range 0° to 15°

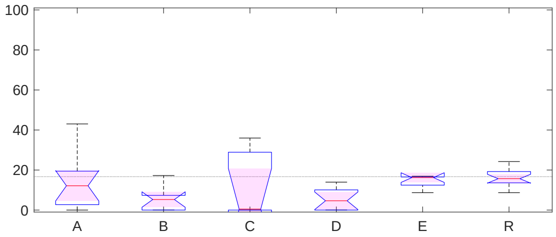

	A	B	C	D	E	R
A	-	.455	.999	.335	.965	.557
B	0.25	-	.881	.999	.170	.008
C	0.25	0.33	-	.849	.923	.596
D	0.25	0.44	0.60	-	.111	.002
E	0.67	0.89	0.60	1.00	-	.994
R	0.67	0.89	0.60	1.00	0.43	-

Edge 4 at ≤100 m — Angle in Range 15° to 30°

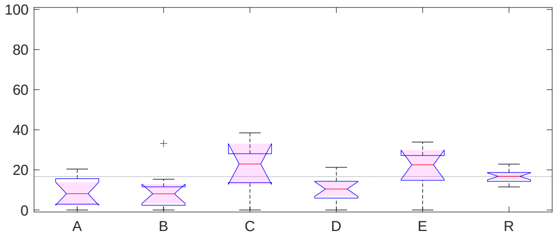

	A	B	C	D	E	R
A	-	.999	.166	.999	.105	.116
B	0.50	-	.166	.998	.112	.135
C	1.00	0.89	-	.260	.999	.992
D	0.58	0.78	0.20	-	.182	.219
E	1.00	0.89	0.40	1.00	-	.993
R	0.75	0.89	0.20	0.77	0.29	-

Edge 4 at ≤100 m — Angle in Range 30° to 45°

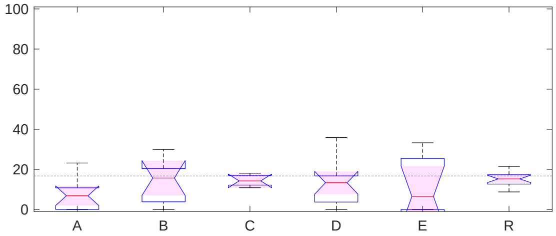

	A	B	C	D	E	R
A	-	.541	.459	.756	.836	.065
B	0.92	-	.999	.997	.999	.977
C	0.83	0.44	-	.967	.985	.999
D	0.83	0.44	0.40	-	.999	.745
E	0.50	0.33	0.00	0.31	-	.897
R	0.92	0.44	0.60	0.69	0.57	-

Edge 4 at $\leq 100\,\mathrm{m}$ **— Angle in Range 45° to 60°**

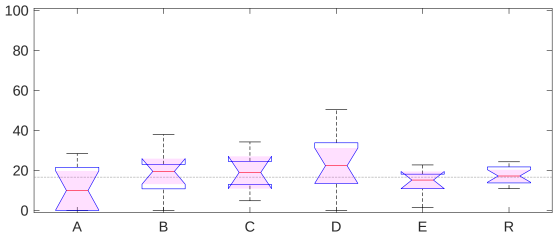

	A	B	C	D	E	R
A	-	.782	.833	.209	.999	.771
B	0.75	-	.999	.974	.952	.999
C	0.75	0.44	-	.996	.954	.999
D	0.75	0.78	0.80	-	.568	.892
E	0.58	0.33	0.20	0.31	-	.964
R	0.75	0.44	0.40	0.38	0.71	-

Edge 4 at $\leq 100\,\mathrm{m}$ **— Angle in Range 60° to 75°**

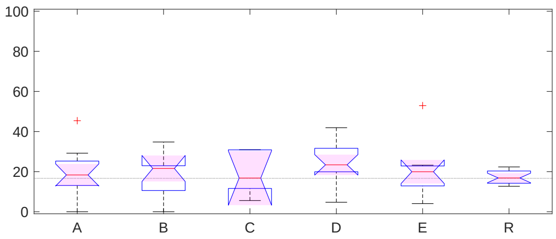

	A	B	C	D	E	R
A	-	.999	.999	.469	.999	.974
B	0.67	-	.999	.524	.999	.988
C	0.33	0.44	-	.758	.999	.992
D	0.67	0.78	0.60	-	.736	.075
E	0.58	0.44	0.60	0.23	-	.969
R	0.33	0.44	0.60	0.08	0.29	-

Edge 4 at $\leq 100\,\mathrm{m}$ **— Angle in Range 75° to 90°**

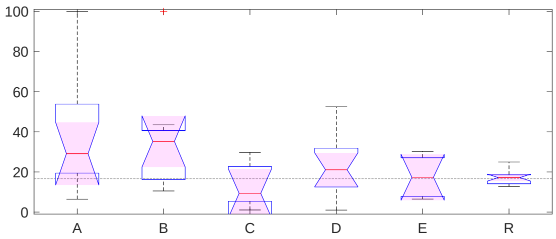

	A	B	C	D	E	R
A	-	.999	.158	.686	.330	.109
B	0.67	-	.240	.820	.462	.231
C	0.08	0.00	-	.781	.995	.992
D	0.25	0.33	0.80	-	.968	.905
E	0.25	0.33	0.60	0.38	-	.999
R	0.25	0.33	0.60	0.38	0.43	-

;
